# A non-targeted LC–MS metabolic profiling of pregnancy: longitudinal evidence from healthy and pre-eclamptic pregnancies

**DOI:** 10.1007/s11306-020-01752-5

**Published:** 2021-01-29

**Authors:** Tiina Jääskeläinen, Olli Kärkkäinen, Jenna Jokkala, Anton Klåvus, Seppo Heinonen, Seppo Auriola, Marko Lehtonen, The FINNPEC Core Investigator Group, Eero Kajantie, Juha Kere, Katja Kivinen, Anneli Pouta, Kati Hanhineva, Hannele Laivuori

**Affiliations:** 1 Medical and Clinical Genetics, University of Helsinki and Helsinki University Hospital, Helsinki, Finland; 2 Department of Food and Nutrition, University of Helsinki, Helsinki, Finland; 3 Institute of Public Health and Clinical Nutrition, University of Eastern Finland, Kuopio, Finland; 4 School of Pharmacy, University of Eastern Finland, Kuopio, Finland; 5 Obstetrics and Gynecology, University of Helsinki and Helsinki University Hospital, Helsinki, Finland; 6 Department of Biochemistry, Food Chemistry and Food Development Unit, University of Turku, Turku, Finland; 7 Institute for Molecular Medicine Finland (FIMM), Helsinki Institute of Life Science, University of Helsinki, Helsinki, Finland; 8 Department of Obstetrics and Gynecology, Faculty of Medicine and Health Technology, Tampere University Hospital and University of Tampere, Tampere, Finland

**Keywords:** Pregnancy, Preeclampsia, Metabolomics, LC–MS

## Abstract

**Introduction:**

Maternal metabolism changes substantially during pregnancy. However, few studies have used metabolomics technologies to characterize changes across gestation.

**Objectives and methods:**

We applied liquid chromatography–mass spectrometry (LC–MS) based non-targeted metabolomics to determine whether the metabolic profile of serum differs throughout the pregnancy between pre-eclamptic and healthy women in the FINNPEC (Finnish Genetics of Preeclampsia Consortium) Study. Serum samples were available from early and late pregnancy.

**Results:**

Progression of pregnancy had large-scale effects to the serum metabolite profile. Altogether 50 identified metabolites increased and 49 metabolites decreased when samples of early pregnancy were compared to samples of late pregnancy. The metabolic signatures of pregnancy were largely shared in pre-eclamptic and healthy women, only urea, monoacylglyceride 18:1 and glycerophosphocholine were identified to be increased in the pre-eclamptic women when compared to healthy controls.

**Conclusions:**

Our study highlights the need of large-scale longitudinal metabolomic studies in non-complicated pregnancies before more detailed understanding of metabolism in adverse outcomes could be provided. Our findings are one of the first steps for a broader metabolic understanding of the physiological changes caused by pregnancy per se.

**Supplementary Information:**

The online version of this article (10.1007/s11306-020-01752-5) contains supplementary material, which is available to authorized users.

## Introduction

Over the course of pregnancy, mothers face a unique physiological challenge that requires complex metabolic adaptations to support fetal growth and development (Lain and Catalano 2007). Profound changes in carbohydrate, fat, and protein metabolism have been reported (Butte 2000; Lain and Catalano 2007; Hadden and McLaughlin 2009). Changes in insulin sensitivity are a hallmark of pregnancy and contribute to the metabolic changes, while nutrient transfer to the fetus are thought to impact the maternal metabolite levels (Hadden and McLaughlin 2009; Catalano 2014). However, studies utilizing metabolomics technologies to characterize maternal metabolism during pregnancy are still relatively few (Lowe and Karban 2014; Zhao et al. 2019). New technologies allow more detailed characterization of metabolic profiles in uncomplicated pregnancies. This may also advance more comprehensive understanding of the pathophysiology of pre-eclampsia (PE), which is a common vascular pregnancy complication associated with exaggareted metabolic changes (Von Versen-Hoeynck and Powers 2007).

We applied liquid chromatography–mass spectrometry (LC–MS) based non-targeted metabolomics to determine whether the metabolic profile of serum differs throughout the pregnancy between women with and without PE in the FINNPEC (Finnish Genetics of Preeclampsia Consortium) study.

## Methods

### Study cohort and serum samples

FINNPEC is a cross-sectional case–control multicentre study with a nationwide clinical and DNA database on women with and without PE, including their partners and newborns. Data of the prospective arm was assembled in Finland between 2008 and 2011. Details of the study design, methods and procedures have been described elsewhere (Jääskeläinen et al. 2016). PE was defined as hypertension and proteinuria occurring after 20 weeks gestation according to the modified American College of Obstetricians and Gynecologists (ACOG) 2002 criteria. Hypertension was defined as systolic blood pressure ≥ 140 mm Hg or diastolic blood pressure ≥ 90 mm Hg. Proteinuria was defined as the urinary excretion of ≥ 0.3 g protein in a 24-h specimen, or 0.3 g/L, or two ≥ 1 + readings on a dipstick in a random urine determination with no evidence of a urinary tract infection. Women with PE superimposed on chronic hypertension were included in the cases. Exclusion criteria were multiple pregnancy, maternal age < 18 years and the inability to provide an informed consent based on information in Finnish or Swedish.

The FINNPEC study protocol was approved by the coordinating Ethics Committee of the Hospital District of Helsinki and Uusimaa. All participants provided written informed consent. All experiments were performed in accordance with relevant guidelines and regulations.

Early and late pregnancy serum samples were collected from a subcohort from the Hospital District of Helsinki and Uusimaa. Early pregnancy serum samples were obtained via first trimester biochemical screening for fetal chromosome abnormalities (range 10–15 weeks of gestation), and late pregnancy (range 23–41 weeks of gestation) serum samples were collected at hospitals. The samples were collected in the 10 ml serum tubes, centrifuged, the serum was removed and stored at − 80 °C.

For the current LC–MS metabolite profiling analysis, we selected samples of mothers who were non-smoking. Furthermore, all women included in the current study were Finnish in origin. In addition, pre-existing diseases (chronic hypertension, pregestational diabetes and gestational diabetes) were exclusion criteria for healthy control women. There were 47 and 53 samples available from early pregnancy and 57 and 14 samples from late pregnancy for PE and healthy women, respectively. Serum samples were available at both timepoints from 47 PE women and from 13 healthy women.

### Non-targeted LC–MS metabolite profiling

The sample preparation, instrument parameters and processing of data were performed in the LC–MS Metabolomics Center at Biocenter Kuopio (University of Eastern Finland). The detailed protocol has been published earlier (Jääskeläinen et al. 2018; Klåvus et al. 2020). Shortly, the serum samples were analyzed by the UHPLC-qTOF-MS system (Agilent Technologies, Waldbronn, Karlsruhe, Germany) that consisted of a 1290 LC system, a Jetstream electrospray ionization (ESI) source, and a 6540 UHD accurate-mass qTOF spectrometer.

The analysis order of the serum samples was randomized. 100 μL of each sample was mixed by pipette with 400 μL of acetonitrile (ACN, LC–MS grade), incubated on an ice bath for 15 min and centrifuged. The supernatant was filtered and collected on a 96-well plate. The samples were analyzed using two different chromatographic techniques, i.e. reversed phase (RP) and hydrophilic interaction (HILIC) chromatography. Data were acquired in both positive (+) and negative (−) polarity. The sample tray was kept at 4 °C during the analysis. The data acquisition software was MassHunter Acquisition B.04.00 (Agilent Technologies). The quality control and the blank samples were injected after every 12 samples and also in the beginning of the analysis. The quality control samples were composed by pooling together a small aliquot of all the study samples.

Data were collected with “Find by Molecular Feature” algorithm in MassHunter Qualitative Analysis B.07.00 software (Agilent Technologies, USA). The extraction algorithm was set to collect peaks with threshold at 200 counts for HILIC and 150 for RP chromatography, and the allowed ion species were [M + H]+, [M + Na]+, [M + K]+, [M + NH4]+, and [2 M + H]+ in ESI(+), and [M − H]−, [M + Cl]−, [M + HCOO]−, [M + CF3COO]−, and [2 M − H]− in ESI(−). Only signals over compound height threshold of 3000 counts containing at least two ions were included in the compound list. Peak spacing tolerance for isotope grouping was 0.0025 m/z plus 7 ppm, with isotope model for common organic molecules. Data files (.cef-format) were exported to Mass Profiler Professional (Agilent Technologies) for peak alignment. After the first initial alignment, the data were combined in one .cef file, against which the original raw data was reanalyzed. For this recursive analysis, compound mass tolerance was ± 15 ppm, retention time ± 0.150 min and symmetric expansion value for chromatograms ± 35.0 ppm. Resulting compounds were re-exported to Mass Profiler Professional software for peak alignment and data cleanup. The signals from the run using HILIC column and negative ionization were excluded from the analysis due to poor data quality. The number of removed potential compounds was 292 out of total 2547 potential compounds, resulting in 2255 potential compounds left for further analysis.

Moreover, we used MS-DIAL ver.2.90 for metabolite identification against exact mass, retention time and MSMS spectra found in our in-house standard library, internal database found in MS-DIAL and Metlin (https://metlin.scripps.edu). Identifications based on in-house library, in which commercial and synthetized compounds have been analyzed using same method and machinery, received identification level of 1. Identification against public libraries received identification level 2. Molecular features where we could identify the class of the compound, but not the exact metabolite, received identification level 3. Molecular features with significant differences between study groups and/or time points with MSMS data but no identification are marked with identification level 4 (unknown).

At the time of the study, the performance of the QCs was not assessed numerically, but the performance has been assessed post-hoc for the molecular features discussed or visualized in this paper. We used quality metrics defined by Broadhurst et al. (2018) to assess the performance of the QCs. In brief, relative standard deviation (RSD, also known as coefficient of variation) measures the spread in feature abundance in the QCs, while D-ratio measures the spread in QCs relative to the spread in the biological samples. RSD* and D-ratio* are robust alternatives for the measures, based on median instead of mean. The averages for each quality metric in the presented features are: RSD: 0.194, RSD*0.123, D-ratio: 0.286, D-ratio*: 0.269. Broadhurst et al. suggest a limit of 0.2 for RSD and a limit of 0.4 for D-ratio, so on average, the data presented here is of high quality, although some of the features do not satisfy these criteria. The following procedures were all conducted separately for each metabolite.

Inverse normal transformation was applied to approximate a normal distribution of metabolite concentrations. Discriminative features between healthy controls and PE women, as well as discriminative features between early and late pregnancy were discovered using linear mixed effects models. Metabolite levels were used as the dependent variable, predicted by early/late pregnancy, PE and the interaction between these two as fixed effects. Subject identifier was used as a random effect to account for inter-individual variation.

Multiple parameters showed statistically significant differences between the study groups. Thus, we investigated how the results on relevant metabolites change when the unmatched subject parameters are added as covariates to the linear mixed models. The covariates we added were parity, SGA, gestational weeks, mode of birth, pregestational diabetes mellitus, gestational diabetes mellitus, chronic hypertension, birth weight, relative birth weight, systolic blood pressure at first visit, highest systolic blood pressure, diastolic blood pressure at first visit, and highest diastolic blood pressure. False discovery rate (FDR) was used to adjust p-values for multiple comparison. The analyses were performed by using the R Project for Statistical Computing version 3.3.3 and speedglm R package version 0.3–1 (R Core Team 2015; Enea 2015).

The Pearson’s correlations were calculated with birth weight of newborn and main findings of the maternal metabolites.

## Results

### Clinical data

Maternal and fetal characteristics in PE and control groups are presented separately for early and late pregnancy in the Table 1. There were no differences in maternal age or body mass index (BMI) between the PE and control groups. PE women had higher systolic and diastolic blood pressure and proteinuria as expected according to the diagnostic criteria for PE. The proportion of nulliparous women was higher in the PE group compared with the control group. Some PE women also suffered from pre-existing diseases (e.g. chronic hypertension, pregestational diabetes) and gestational diabetes. PE women had also more caesarean deliveries than control women. The newborns of the PE pregnancies were born earlier and had smaller absolute and relative birth weights (Table 1). There was no difference in the sex distribution of newborns. When compared to whole FINNPEC population (n = 2515, Jääskeläinen et al. 2016), there were greater proportion of early-onset PE (18.0% vs. 30.4%), women with chronic hypertension (26.1% vs. 17.9%) and pregestational diabetes (8.7% vs. 3.2%) in the current study.

**Table 1:** Maternal and fetal characteristics in pre-eclamptic (PE) and control groups separately for early and late pregnancy

	Early pregnancy (10–15 weeks of gestation)			Late pregnancy (23–41 weeks of gestation)		
	PE	Control	*p*	PE	Control	*p*
n	46	53		57	14	
Gestational week at serum sampling	12 ± 1 (mean ± S.D)	12 ± 1	0.073	35 ± 4	39 ± 1	**0.006**
Age at delivery, year	31.4 ± 4.6	30.9 ± 4.5	0.997^a^	31.2 ± 4.5	32.0 ± 3.4	0.380
Nulliparous (%)	35 (76.1%)	23 (43.4%)	**0.001**	40 (70.2%)	5 (35.7%)	**0.028**
BMI, kg/m^2^ (self-reported, pre-pregnancy)	25.7 ± 4.5	25.4 ± 2.4	0.679	25.6 ± 4.4	24.0 ± 3.1	0.196
Systolic blood pressure at first antenatal visit, mm Hg	124 ± 15	117 ± 9	**0.010**	124 ± 15	117 ± 8	0.067
Diastolic blood pressure at first antenatal visit, mm Hg	80 ± 11	72 ± 8	** < 0.001**	81 ± 10	71 ± 7	**0.002**
Early onset of PE (delivery ≤ 34 + 0 weeks of gestation)	14 (30.4%)	–	–	42 (73.7%)	–	–
Preterm delivery (≤ 37 + 0 weeks of gestation	19 (41.3%)	0 (0.0%)	** < 0.001**	20 (35.1%)	0 (0.0%)	**0.007**^**a**^
Highest systolic blood pressure, mm Hg**	169 ± 15	124 ± 9	** < 0.001**^**a**^	169 ± 14	125 ± 9	** < 0.001**^**a**^
Highest diastolic blood pressure, mm Hg	112 ± 12	84 ± 6	** < 0.001**^**a**^	111 ± 11	82 ± 6	** < 0.001**^**a**^
Proteinuria (maximum), g/24 h	4.6 ± 4.1	–	–	4.2 ± 3.8	–	–
Chronic hypertension	12 (26.1%)	0 (0.0%)	** < 0.001**^b^	14 (24.6%)	0 (0.0%)	0.057^b^
Gestational diabetes mellitus	7 (15.2%)	0 (0.0%)	**0.004**^b^	11 (19.3%)	0 (0.0%)	0.106^b^
Pregestational diabetes mellitus	4 (8.7%)	0 (0.0%)	**0.043**^b^	5 (8.8%)	0 (0.0%)	0.575^b^
Mode of delivery			** < 0.001**			0.185
Vaginal	24 (52.2%)	47 (88,7%)		30 (52.6%)	9 (64.3%)	
Caesarean section	22 (47.8%)	6 (11.3%)		27 (47.4%)	5 (35.7%)	
Fetal characteristics						
Birth weight, g	2435 ± 1003	3627 ± 428	** < 0.001**^**a**^	2602 ± 1010	3644 ± 467	** < 0.001**^**a**^
Relative birth weight, SD	− 1.3 ± 1.4	0.1 ± 0.9	** < 0.001**	− 1.1 ± 1.5	0.2 ± 0.9	**0.002**
SGA	11 (23.9%)	0 (0.0%)	** < 0.001**	12 (21.1%)	0 (0.0%)	0.106
Gestational weeks	36 ± 4	40 ± 1.4	** < 0.001**^**a**^	36 ± 4	39 ± 1	**0.001**^**a**^
Sex			**0.035**			0.139
Girl	28 (60.9%)	21 (39.6%)		34 (59.6%)	5 (35.7%)	
Boy	18 (39.1%)	32 (60.4%)		23 (40.4%)	9 (64.3%)	

Level of significance for bolded values is *p* < 0.05

Chronic hypertension was defined as systolic blood pressure ≥ 140 mm Hg and/or diastolic blood pressure ≥ 90 mm Hg detected before 20 weeks of gestation. Gestational hypertension was defined as blood pressure ≥ 140/90 without proteinuria

*SD* birth weight and height converted to standard deviation scores, *SGA* small-for-gestational age

^a^Non-parametric test was used

^b^Fisher’s exact test

### Metabolite profiling

In the metabolite profiling analysis, we observed altogether 1288 molecular features with *p*-value < 0.05 when comparing samples from early to late pregnancy. After FDR correction for multiple testing, 1183 molecular features differed between samples from early to late pregnancy with a *p*-value < 0.05 (Supplementary Table 1).

**Fig. 1:**
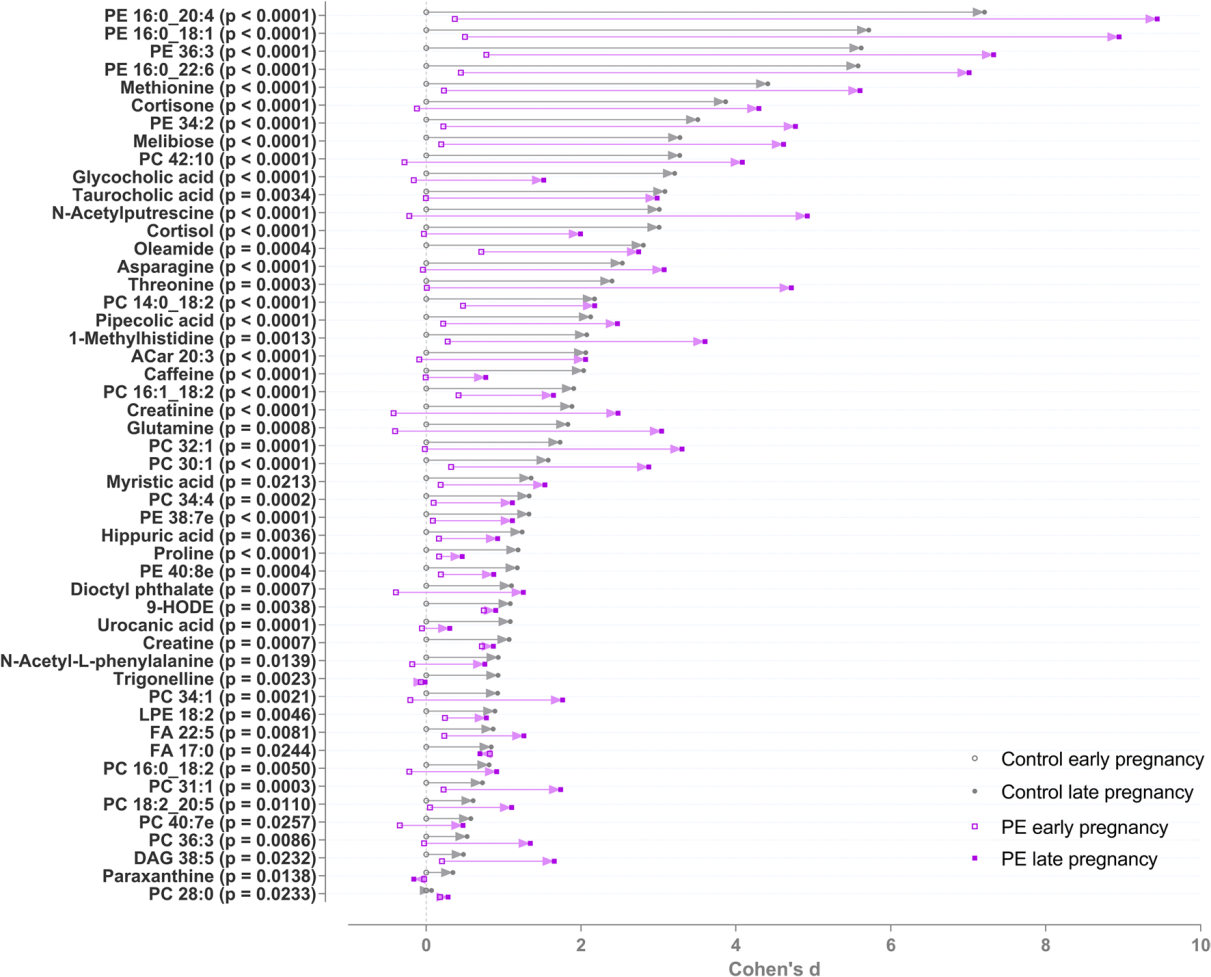
Metabolites increased in pre-eclamptic and control women from early to late pregnancy. All metabolites shown are significant after false discovery rate correction for multiple testing. Cohen’s d values when compared to early pregnancy control samples and raw p-values from linear mixed model analysis are shown. The d-value shows the mean difference divided by standard deviation compared to mean metabolite level in the control group 1st trimester samples. Positive d-value means that the increased, and negative that they were decreased, when compared to the 1st trimester samples from the control group. The level of identifications for each metabolite can be found in the Supplementary Table 1. *PE* phosphatidylethanolamine, *PC* phosphatidylcholine, *ACar* acylcarnitine, *LPE* lysophosphatidylethanolamine, *FA* fatty acid, *DAG* diacylglyceride

Figure 1 illustrates the identified metabolites that increased during pregnancy in the two groups. Particularly following metabolite groups were observed to increase: phosphatidylethanolamines and -cholines; amino acids (asparagine, threonine, proline, methionine) and bile acids (glycocholic acid and taurocholic acid). Furthermore, metabolites associated with caffeine metabolism (caffeine, trigonelline and paraxanthine) were detected to be elevated. Hippuric acid was also increased. The greatest increases of individual metabolites were detected in phosphatidylethanolamines, methionine and cortisone (Fig. 1).

**Fig. 2:**
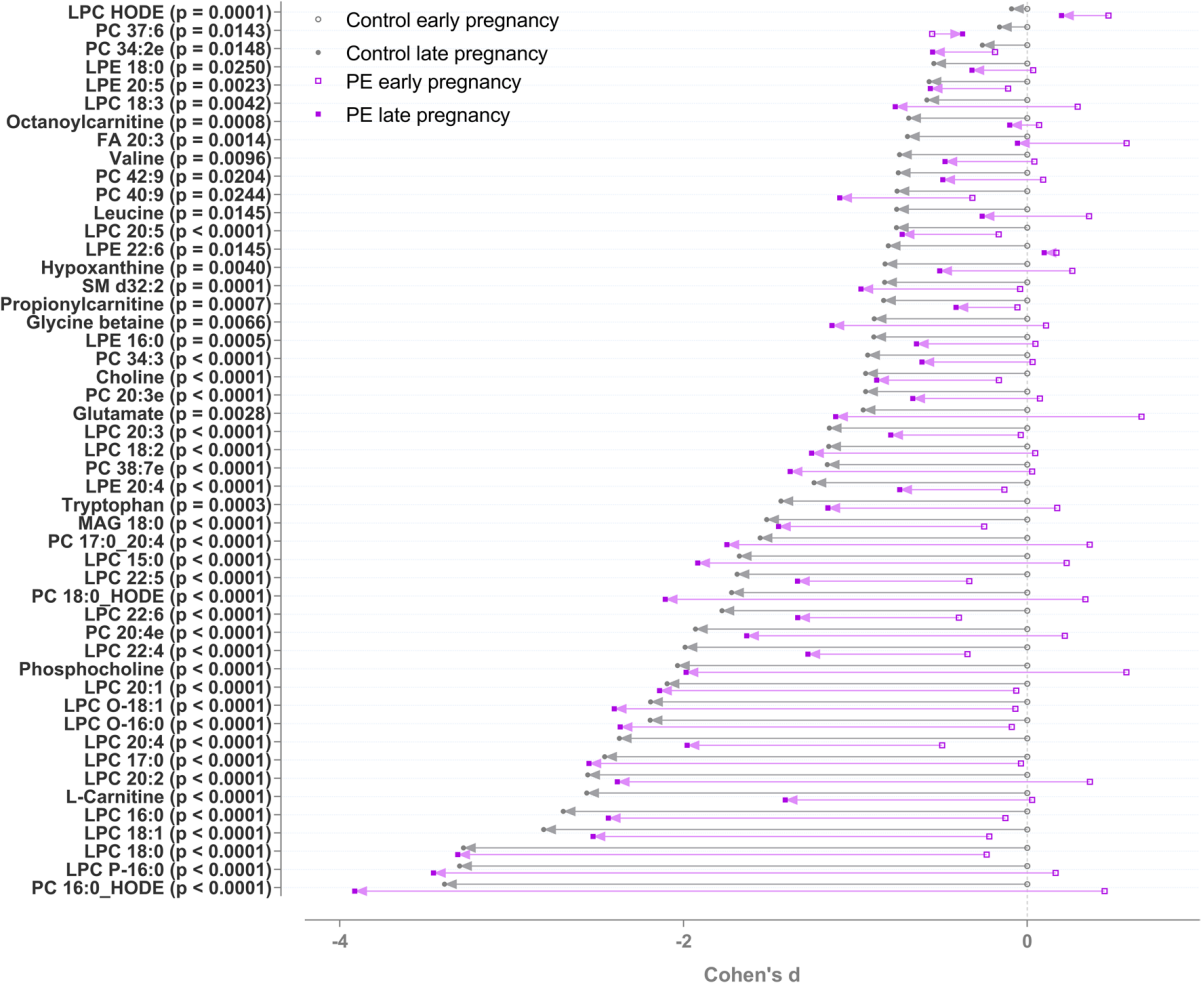
Metabolites decreased in pre-eclamptic and controls groups from early to late pregnancy. All metabolites shown are significant after false discovery rate correction for multiple testing. Cohen’s d values when compared to early pregnancy control samples and raw p-values from linear mixed model analysis are shown. The d-value shows the mean difference divided by standard deviation compared to mean metabolite level in the control group 1st trimester samples. Positive d-value means that the increased, and negative that they were decreased, when compared to the 1st trimester samples from the control group. The level of identifications for each metabolite can be found in the Supplementary Table 1. *PE* phosphatidylethanolamine, *PC* phosphatidylcholine, *ACar* acylcarnitine, *LPE* lysophosphatidylethanolamine, *LPC* lysophosphocholine, *FA* fatty acid

The identified metabolites that decreased during pregnancy in the two groups are depicted in Fig. 2. The metabolites included following metabolite groups: lysophosphatidylcholines (LPCs), lysophosphatidylethanolamines (LPEs), branched-chain amino acids (valine and leucine), trimethylated compounds, (choline, glycine betaine) and carnitines (octanoyl-carnitine and l-carnitine). In addition, tryptophan decreased in both groups.

**Fig. 3:**
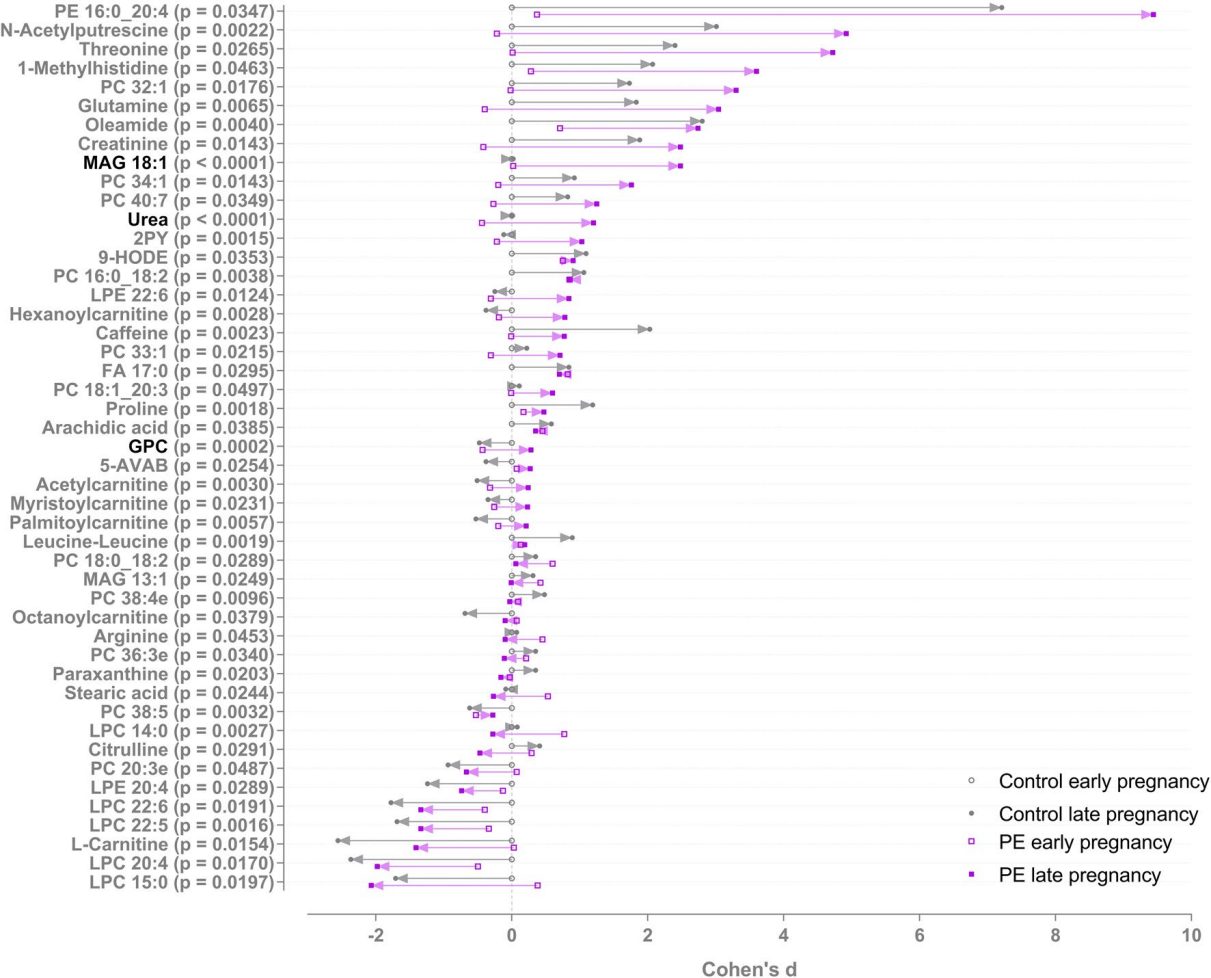
Metabolites in which changes differed in PE and controls groups from first to third trimester. Raw p-value is shown, metabolites with significant p-values after FDR correction for multiple testing are shown in black. Cohen’s d values when compared to 1st trimester control samples and raw p-values from linear mixed model analysis are shown. The d-value shows the mean difference divided by standard deviation compared to mean metabolite level in the control group 1st trimester samples. Positive d-value means that the increased, and negative that they were decreased, when compared to the 1st trimester samples from the control group. The level of identifications for each metabolite can be found in the Supplementary Table 1. *PE* phosphatidylethanolamine, *PC* phosphatidylcholine, *ACar* acylcarnitine, *LPE* lysophosphatidylethanolamine, *LPC* lysophosphocholine, *FA* fatty acid, *MAG* monoacylglyceride

**Fig. 4:**
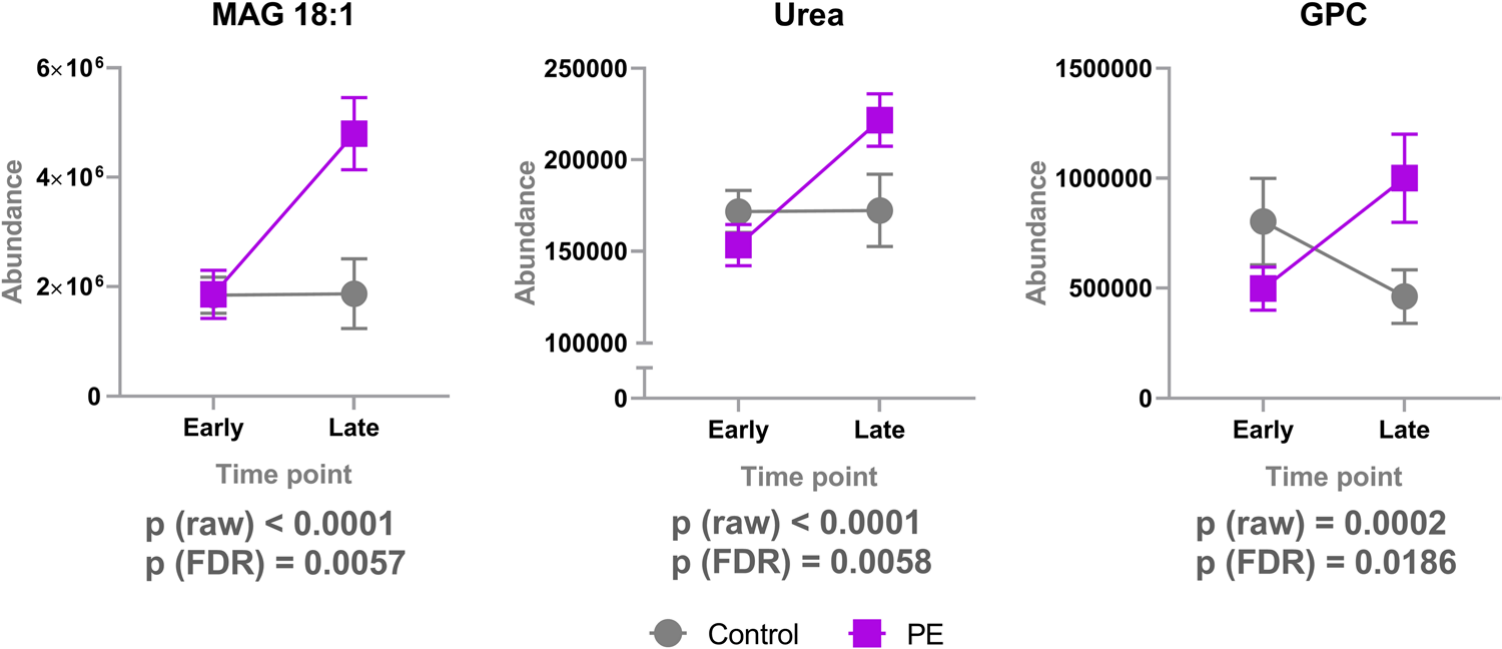
Metabolites in which changes differed significantly between PE and control groups from early to late pregnancy. Mean and 95% confidence intervals are shown. MAG 18:1, monoacylglyceride 18:1 (level of identification: 1); GPC, glycerophosphocholine (level of identification: 1); urea (level of identification: 2)

There were altogether 398 molecular features in which changes differed between PE and controls groups from early to late pregnancy with a p-value < 0.05. Of these, 47 were identified at level 1 and 2. 42 of the 398 features remained significant after FDR correction and three of these were identified with confidence level 1 and 2 (Fig. 3). These three significantly increased metabolites were monoacylglyceride 18:1 (MAG 18:1), urea and glycerophosphocholine (GPC) (Fig. 4). Levels of MAG 18:1 and urea increased in PE women, whereas there were no changes in the serum of control women. Furthermore, choline derivative, GPC levels decreased in control and increased in PE women.

We also investigated how the results on GPC, MAG and urea change when the unmatched subject parameters are added as covariates to the linear mixed models. The results are presented in Table 2. For GPC, the covariates explained approximately 13% of the effect, but the *p*-value of the regression coefficient remained below 0.001. For MAG and urea, adding covariates to the model did not change the results significantly.

**Table 2:** The proportion of the effect explained by the covariates (the difference between the unadjusted and adjusted regression coefficient, divided by the unadjusted regression coefficient)

Compound	Regression coefficient before adjustment	Regression coefficient after adjustment	Proportion of effect explained by covariates		*p*-value before adjustment	*p*-value after adjustment
GPC	1.348	1.173		0.129600	1.732E−04	9.515E−04
MAG	1.393	1.368		0.018240	7.61E−06	1.134E−05
Urea	1.3	1.294		0.004041	1.34E−05	2.00E−05

The strongest negative correlations with birth weight of newborn and maternal metabolite were detected with between following LPCs: LPC 20:4 (r = − 0.55, p < 0.001), LPC 20:3 (r = − 0.51, p < 0.001), LPC 22:4 (r = − 0.51, p < 0.001), LPC 22:3 (r = − 0.55, p < 0.001), LPC 22:6 (r = − 0.52, p < 0.001), LPC 20:4 (r = − 0.55, p < 0.001). In addition, there was negative correlation between birth weight and carnitine (r = − 0.62, p < 0.001) and palmitoylcarnitine (r = − 0.42, p < 0.001). The strongest positive correlations were observed between birth weight and cortisol (r = 0.58, p < 0.001) and cortisone (r = 0.59, p < 0.001).

## Discussion

Our work describes systemic metabolic changes PE and healthy pregnant women in early and late pregnancy. Progression of pregnancy from first to third trimester had large scale effects to the serum metabolite profile highlighting the remarkable dynamic and adaptive processes occurring in maternal metabolism. The differences in metabolic changes between PE women and healthy controls in early and in late pregnancy were relatively modest. Only urea, MAG 18:1 and GPC were identified to be increased in PE when compared with control pregnancies.

Particularly following metabolite groups were observed to be increased in both PE and control groups: phosphatidylethanolamines and –cholines, amino acids and bile acids. Metabolites associated with caffeine metabolism were elevated as well. Furthermore, hippuric acid was increased. The greatest increases of individual metabolites were detected in phosphatidylethanolamines, methionine and cortisone.

Phosphatidylethanolamines are the second most abundant phospholipids in mammalian cells (Patel and Witt 2017). They have critical role in energy metabolism, they are especially abundant in the inner mitochondrial membrane. Furthermore, they assists in the folding of certain membrane proteins, are required for the activity of several of the respiratory complexes. The changes in the levels of phosphatidylethanolamines and/or -cholines in various tissues are implicated in metabolic disorders, such as atherosclerosis, insulin resistance and obesity (van der Veen et al. 2017). To our knowledge, there are no previous data available describing their exact role in pregnancy. However, since the mitochondrial oxidation of fatty acids is known to be essential for meeting the fetal energy needs, the increased levels of phosphatidylethanolamines may indeed be critical over the course of pregnancy when physiological insulin resistance occurs. Previously, Colleoni et al. (2010) have shown that there is a progressive decrease of mitochondrial DNA content in blood of pregnant women across uncomplicated gestation compared to nonpregnant women. Thus, it could be speculated that the increase in phosphatidylethanolamines is not explained by the changed number of mitochondria.

The decreased maternal carnitine levels in the current study might reflect the enhanced fetal mitochondrial β-oxidation processes. This is in line with previous observations reported in uncomplicated pregnancies suggesting enhanced oxidative activity across gestation (Luan et al. 2014; Lindsay et al. 2015). Carnitine is also required by the fatty acid oxidation in the placental-fetal unit which is thought to be primarily met through placental carnitine uptake from the maternal circulation (Grube et al. 2005). This may further contribute to the reduction in maternal plasma carnitine levels throughout pregnancy.

Two bile acids, glycocholic and taurocholic acid were shown to be increased. A gradual increase in serum bile acid levels is a known feature of metabolic adaptations during pregnancy (McIlvride et al. 2017). Several studies have previously provided evidence that during normal pregnancy, women develop sub-clinical cholestasis (Lunzer et al. 1986, Pascual et al. 2002). A very recent paper demonstrated that conjugated bile acids including glycocholic and taurocholic acid eventually represent the majority of total bile acids as the pregnancy proceeds (Zhu et al. 2019). These changes are likely related to changes in energy metabolism and gut microbiota associated with normal physiology of pregnancy.

In the current study, metabolites associated with caffeine metabolism were also increased (caffeine, trigonelline and paraxanthine). It is known that half-life of caffeine is increased during pregnancy (Knutti 1982). Furthermore, maternal caffeine intake has been reported to be associated with a reduction in birth weight, however, the precise level of intake above which the risk is increased remains unknown (Sengpiel et al. 2013). To our knowledge, paraxanthine and trigonelline have not specifically been reported in pregnancy. However, it is known that paraxanthine is the primary metabolite of caffeine and trigonelline performs well as a marker of coffee intake (Midttun et al. 2018).

Hippuric acid is another interesting metabolite related to dietary intake. It is typically increased with increased consumption of phenolic compounds (tea, wine, fruit and vegetables) (Krupp et al. 2012). Furthermore, it is a well-known mammalian-microbial cometabolite as it is produced in liver from benzoic acid via glycine conjugation (Lees et al. 2013). Thus, the observed increase might also reflect changes in microbiome composition and/or liver metabolism that take place during gestation. To our knowledge, there are only few studies reporting hippuric acid levels in pregnancy. Austdal et al. (2015) have shown that reduced hippurate excretion in urine samples precede PE. In addition, there is one old study that has reported hippuric acid in pregnancy (Hirsheimer 1935). Serum hippuric acid has also been shown to correlate with fasting C‐peptide levels (Luo et al. 2016), and thus one could speculate that this might serve as a marker of enhanced insulin secretion in pregnancy as well.

Asparagine, threonine, proline, methionine were the amino acids that increased in both PE and healthy pregnancies. It is known that maternal amino acids are considered to be key determinants for fetal growth (Liu and Arany 2014). However, information on maternal amino acids during pregnancy is quite limited and comes from studies with only small numbers of participants (Cetin 2005; Rossary et al. 2014). Of the individual amino acids, methionine increased most in the both groups. Methionine is an essential amino acid and not only contribute to protein mass, but is also a key component in one carbon metabolism. (Dasarathy et al. 2010). Methionine provides the one carbon units for the numerous methyl transferase reactions participating in key metabolic reactions like DNA synthesis and thus, in pregnancy it could be expected the levels to be increased for the needs of growing fetus (Kalhan 2016).

The identified metabolites that decreased during pregnancy included LPCs, LPEs, branched-chain amino acids, trimethylated compounds and carnitines (octanoyl-carnitine and l-carnitine). In addition, tryptophan decreased in both groups.

LPCs are biologically active lipids that comprise a major class of lipids in human plasma. Previously, LPCs in cord blood have been shown to be positively associated with birth weight (Robinson et al. 2018; Hellmuth et al. 2017). The decreased maternal levels and negative correlations with birth weight observed in the current study might reflect the increased fetal need of LPCs. Interestingly, increased levels of LPCs have also been defined as indicators of metabolic health in obesity, as LPCs appear to have glucose-lowering and anti-inflammatory effects (Lehmann et al. 2013). However, their specific role in pregnancy remain to be elucidated.

Branched-chain amino acids, valine and leucine, decreased in both groups. There is indeed previous evidence to suggest that branched-chain amino acid metabolites, as well as the branched-chain amino acids themselves, contribute to insulin resistance and metabolic dysfunction (Lynch and Adams 2014). Furthermore, it is known that there is a large placental utilization of the branched-chain amino acids, some of which are transaminated to alpha ketoacids (Manta-Vogli et al. 2020).

The depleted maternal levels of trimethylated compounds, choline, glycine betaine may typically reflect generally the fetus’s high demand for methyl donors, as has been suggested earlier (Visentin et al. 2015). We have previously reported a clear increase in various trimethylated compounds particularly in cord plasma of the newborns of PE mothers (Jääskeläinen et al. 2018). Increased fetal choline level might also be associated with higher phospholipid demand for the fetus brain development during later periods of pregnancy (Zeisel 2006).

We identified three metabolites in which changes differed significantly between the PE and the control groups from early to late pregnancy. Increased levels of both MAG 18:1 and GPC may highlight the previous findings that there may be a characteristic lipid metabolism in women with PE (De Oliveira et al. 2012).

To our knowledge, the specific increase of MAG18:1 in PE mothers has not been reported before. Romanowicz and Bańkowski (2009) have presented the individual MAG-fractions in umbilical cord artery wall of newborns of PE mothers, and one of the most common unsaturated fatty acids occurring in MAG, was C18:1. It is well known that adaptation of a maternal lipid metabolism takes place throughout gestation. Villa et al. (2009) have observed that free fatty acids including oleic acid are higher in women with PE. Several characteristics of PE, such as increased insulin resistance, disturbed endothelial cell function and altered production of vasoactive substances, may be influenced by high concentrations of FFAs. However, the role of MAG 18:1 in PE remain to be elucidated.

GPC is a choline derivative and one of the two major forms of choline storage (along with phosphocholine) in the cytosol. GPC play an important role in the structural integrity of cell membranes (Klein 2000). Its increase may suggest a protective mechanism against cell damage which could also be a consequence of the oxidative stress present in PE (Aouache et al. 2018).

Increased levels of urea are known to be increased in women with PE (Roopnarinesingh and Morris 1971). Earlier, we have also observed increased levels of urea, creatine, creatinine, homocitrulline and guanidinopropionate in the cord plasma samples of the PE newborns (Jääskeläinen et al. 2018). This may indicate altered function of the urea cycle in both women with PE mothers and their newborns. It is possible that these changes reflect a derangement in the mechanism required for the elimination of these metabolites from the maternal and/or the fetal compartment.

Our study had several strengths. First, it is the one of the few studies to utilize non-targeted LC–MS method investigating healthy and PE pregnancy. Very recently Sovio et al. (2020) conducted a case–cohort study to analyse untargeted maternal serum metabolomics in samples from 12, 20, 28 and 36 weeks of gestational age in women with pre-eclampsia delivering at term and pre-term. Interestingly, they observed 4-Hydroxyglutamate and C-glycosyltryptophan to be independently predictive at 36 weeks of gestation of term PE. Furthermore, Sander et al. (2019) have performed non-targeted metabolomics on plasma samples from PE and healthy pregnant women but the samples were available only in the third trimester. Interestingly, they also observed similar metabolites to contribute to metabolic disturbances of PE women (e.g. bile and amino acids). Previous studies have mainly focused on targeted metabolites but not overall profiling of maternal serum or plasma. Luan et al. (2014) have investigated healthy women throughout pregnancy but there were no multiple collection of samples for the same subject, thus study reflected mostly inter-subject variability. Lindsay et al. (2015) have prospectively followed non-diabetic women from first trimester until the end of their pregnancy but they used targeted metobolomic technique only. In a very recent study, Zhao et al. (2019) performed a longitudinal (first to second trimester) non-targeted metabolomics evaluation of GDM women compared with healthy pregnant women. However, in future it would be valuable to obtain longitudinal data from all trimesters since there are significant alterations occurring in maternal metabolism in late pregnancy as well (Lain and Catalano 2007). One limitation of our study is that we had limited number of serum available and only very few samples from the second trimester. This was due to fact that serum samples were originally available only from a subset and have already been utilized in the previous FINNPEC studies. Furthermore, there were various exclusion criteria (e.g. smoking) for the current study. However, current samples were good representative of the whole FINNPEC population (Jääskeläinen et al. 2016) although there were greater proportion of early-onset PE, women with chronic hypertension and pregestational diabetes in the current study, thus probably representing more severe form of the disease. In addition, another limitation is the observational nature of the study, which complicates causal interpretation of the results. Furthermore, one could also speculate that difference in gestational weeks at late pregnancy serum sampling might have affected the results.

Another challenge with PE is that the condition is heterogeneous in origin, which results in considerable variation in the clinical presentation. Due to the mixed population, the results of this study are especially meaningful for the characterisation of the PE syndrome but not necessarily for the etiology of the disease. Furthermore, it might be that the participants of FINNPEC represent a more severe end of the disease since the recruitment occurred at university hospitals only.

It should also be noted that the changes described in maternal metabolism across pregnancy may reflect changes in placental metabolism (Dunn et al. 2012). Furthermore, the metabolome is affected by genetic, dietary and environmental contributors (Kadakia et al. 2019). We were not able to control for all of these confounding factors. In future, particularly sufficient dietary data would be useful since some of the metabolites we detected appeared to be related to dietary intake.

We conclude that LC–MS based non-targeted technology is crucial for gaining understanding of complexity of metabolic changes across gestation. In a cohort of women with PE and healthy pregnant women, we demonstrated that metabolic signatures from early to late pregnancy are largely shared. Our study highlights the need of large-scale longitudinal metabolomic studies in uncomplicated pregnancies before we could provide more detailed understanding of metabolism in adverse outcomes such as in PE. Our findings are one of the first steps for a broader metabolic understanding of the physiological changes caused by pregnancy per se.

## Electronic supplementary material

Below is the link to the electronic supplementary material.

Electronic supplementary material 1 (PDF 1556 kb)

## Abbreviations

FINNPEC: Finnish Genetics of Preeclampsia Consortium
GPC: Glycerophosphocholine
LC–MS: Liquid chromatography-mass spectrometry
LPC: Lysophosphatidylcholine
LPE: Lysophosphatidylethanolamines
MAG: Monoacylglyceride
MPP: Mass profiler professional
NO: Nitric oxide
PC: Phosphatidylcholine
PCA: Principal component analysis
PE: Pre-eclampsia
PLS-DA: Partial-least-squares discriminant-analysis
RP: Reversed phase
SD: Relative birth weight
VIP: Variable importance to the projection
