# Supplementary material for: A non-targeted LC–MS metabolic profiling of pregnancy: longitudinal evidence from healthy and pre-eclamptic pregnancies
Source: Metabolomics. 2021 Jan 29;17(2):20. doi: 10.1007/s11306-020-01752-5 (PMC7846510; doi:10.1007/s11306-020-01752-5)
Supplement: Supplementary file 1 — Electronic supplementary material 1 (PDF 1556 kb) [file 11306_2020_1752_MOESM1_ESM.pdf]

Supplementary information

A NON-TARGETED LC-MS METABOLIC PROFILING OF PREGNANCY: LONGITUDINAL EVIDENCE FROM HEALTHY AND PRE-ECLAMPTIC PREGNANCIES

Tiina Jääskeläinen[1], Olli Kärkkäinen[2,3], Jenna Jokkala[2], Anton Mattsson[2], Seppo Heinonen[4], Seppo Auriola[3], Marko Lehtonen[3], FINNPEC, Kati Hanhineva[2], Hannele Laivuori[1,5,6]

Supplementary table 1: Results of the metabolomics study (ordered according to molecular mass)

									1st trimester				3rd trimester									
									Control		Pre-eclampsia		Control		Pre-eclampsia		Time			Interaction		
Compound	Column	Ion_mode	Mass	Retention_	Identification	ID_level	MSMS_spectra	CompositeSpectrum	Mean	SD	Mean	SD	Mean	SD	Mean	SD	Value	raw p	FDR_p	Value	raw p	FDR_p
COMPOUND_60_0323a0_9495243	Hilic	+	60.0323	0.95	Urea	2	10 eV: 44.0133 (100), 61.0401 (70)	(121.0721, 15746.63)(122.0775, 606.07)(123.0784, 204.26)(83.0217, 642.82)(78.0672, 196.26)(61.0393, 41081.3)(62.0421, 691.72)(63.0436, 205.5)	171763.2	41675.37	153324.3	37866.51	172303.3	34207.08	221770.5	53922.98	0.01	0.981483	0.990277	1.30	1.34E-05	0.005768
COMPOUND_68_0622a4_7624702	Rp	+	68.0622	4.76				(69.0698, 5314.49)(70.0728, 412.9)	19392.46	5336.748	19921.72	4031.242	20473.7	4378.354	19455.22	5162.023	-0.34	0.2527	0.348701	0.26	0.468749	0.734042
COMPOUND_85_0529a0_8500329	Hilic	+	85.0529	0.85				(171.1134, 282.94)(86.06, 8444.94)(87.0622, 268.39)	10158.79	3372.965	10951.3	3501.109	13657.43	3464.032	14734.3	5109.426	0.79	0.002708	0.006529	0.09	0.763762	0.899577
COMPOUND_89_0477a5_551579	Hilic	+	89.0477	5.55				(112.0371, 1981.5)(113.0412, 134.81)(201.0921, 287.74)(90.055, 8697.23)(91.0569, 246.96)(92.0594, 96.86)	19087.38	2746.593	20243.37	3137.107	24277.5	4356.908	26045.23	5255.566	1.21	3.52E-07	1.99E-06	-0.05	0.852991	0.937499
COMPOUND_103_0634a5_112903	Hilic	+	103.0634	5.11				(126.0562, 273.43)(104.0707, 16203.7)(105.0738, 595.69)(106.0753, 186.77)	50989.17	8448.219	52663.89	9057.901	#DIV/0!	#DIV/0!	#DIV/0!	#DIV/0!	-1.41	1.54E-13	1.25E-11	-0.14	0.418222	0.69956
COMPOUND_103_0998a1_4269782	Hilic	+	103.0998	1.43	Choline	2	10 eV: 104.1075 (100), 60.0812 (25), 45.0338 (5)	(207.2069, 38011.88)(208.2103, 4932.08)(209.2105, 513.94)(104.107, 54639.89)(105.1102, 2944.98)(106.1127, 375.49)	62789.26	53520.99	53932.93	40914.87	12483.43	4889.389	15913.51	8131.051	-1.64	3.47E-10	4.44E-09	0.34	0.19379	0.505089
COMPOUND_103_0999a10_346933	Rp	+	103.0999	10.35				(207.2079, 235.55)(126.0914, 558.41)(104.1073, 5429.01)(105.1104, 432.79)	45150.07	16833.65	46777.05	15855.33	24937	10521.29	24046.35	7438.76	-0.65	0.019912	0.039301	-0.08	0.816152	0.92157
COMPOUND_108_0935a5_238501	Rp	+	108.0935	5.24				(109.1014, 5072.61)(110.1043, 663.33)(111.1165, 68.43)	17256.13	4139.061	17369.93	3360.317	18423.73	4656.411	18206.21	4359.348	0.26	0.351159	0.453281	-0.27	0.416625	0.699135
COMPOUND_109_064a6_711251	Hilic	+	109.0640	6.71				(110.0713, 10463.09)(111.0745, 1050.79)(112.087, 310.83)	#DIV/0!	#DIV/0!	#DIV/0!	#DIV/0!	#DIV/0!	#DIV/0!	#DIV/0!	#DIV/0!	0.62	0.019566	0.038754	0.01	0.968674	0.982722
COMPOUND_110_1094a4_7634273	Rp	+	110.1094	4.76				(111.1169, 9512.58)(112.1205, 1017.01)(113.1226, 104.3)	37439	10331.78	38187.13	7947.74	39293.77	8743.396	38010.06	11063.48	0.07	0.821818	0.873812	-0.07	0.845973	0.93343
COMPOUND_113_059a1_2121515	Hilic	+	113.0590	1.21	Creatinine	2	10 eV: 114.0671 (100), 44.0499 (85), 86.0718 (10)	(227.1257, 286196.84)(228.1286, 30190.36)(229.1304, 3118.65)(230.1317, 233.56)(136.0485, 21687.05)(137.0517, 585.62)(138.0558, 278.39)(249.1075, 4435.64)(250.1062, 461.47)(251.1038, 122.89)(114.0658, 1177171.5)(115.0694, 57221.78)(116.071, 4209.83)	13626415	1645758	12932336	1889497	16721429	2062925	17701754	2885152	1.00	2.57E-06	1.16E-05	0.56	0.014344	0.179521
COMPOUND_113_0842a2_9012837	Rp	+	113.0842	2.90				(227.1753, 581.27)(228.1775, 61.06)(136.0759, 250.35)(137.0805, 23.79)(249.1508, 565.74)(250.1447, 307.29)(114.0914, 30002.53)(115.0944, 2195.38)(116.0936, 32.87)	44958.97	22081.96	49336.73	32384.05	47082.11	34701.17	83024.5	69201.06	0.13	0.559671	0.656827	0.31	0.24631	0.555348
COMPOUND_113_0843a3_084201	Rp	+	113.0843	3.08				(227.1763, 47621.71)(228.1791, 6980.99)(229.1821, 612.46)(136.0734, 10454.63)(137.0757, 778.08)(138.0776, 114.49)(249.1582, 2492.78)(250.1598, 335.97)(251.1703, 36.52)(152.0431, 1220.94)(114.0912, 574859.8)(115.0946, 37249.12)(116.097, 2278.86)	4247166	448273	4159536	463146.2	4483274	548903.7	4209974	352462.8	0.46	0.129038	0.202363	-0.24	0.503519	0.754442
COMPOUND_115_0633a4_706444	Hilic	+	115.0633	4.71	Proline	2	10 eV: 70.0655 (100), 116.0707 (40)	(231.1344, 717.9)(232.1387, 196.85)(233.1284, 203.59)(138.0529, 39656.23)(139.0553, 2096.5)(253.1165, 1233.65)(254.1252, 238.3)(255.1202, 44.71)(116.0706, 952364.4)(117.0742, 52415.54)	4092242	896065.5	4242154	951260.7	5154669	1009436	4509167	1122848	1.10	2.03E-05	7.55E-05	-0.90	0.001827	0.061415
COMPOUND_115_0636a7_1022463	Hilic	+	115.0636	7.10	Ornithine (m/z 133.0974)	1	10 eV: 70.0651 (100), 116.0700 (55), 133.0961 (3)	(138.0527, 268.88)(133.0973, 58821.84)(134.1005, 3725.79)(135.1011, 478.01)(116.0705, 24649.64)(117.0735, 1389.89)	143719.2	64241.31	161749.9	68720.46	152658.4	62940.56	133701.9	55456.93	0.23	0.440248	0.545645	-0.60	0.095685	0.364606
COMPOUND_117_0535a5_6224656	Hilic	+	117.0535	5.62	Unknown betaine	4	20 eV: 59.0724 (100), 118.0863 (45), 58.0650 (40)	(140.0431, 2405.27)(141.0473, 76.76)(118.0609, 11678.5)(119.0652, 341.66)(120.0642, 139.41)	20275.04	3955.277	21921.3	4868.291	19188.92	4160.325	24255.68	8323.502	-0.38	0.146498	0.223821	0.68	0.030544	0.234758
COMPOUND_117_0787a3_4925504	Hilic	+	117.0787	3.49	Glycine betaine	1	10 eV: 118.0866 (100), 59.0733 (20), 58.0653 (15)	(235.1655, 5408.17)(236.1678, 606.03)(237.17, 626.53)(140.0684, 11153.54)(141.072, 1117.05)(142.0701, 119.74)(257.1477, 1285.88)(258.1521, 239.96)(259.152, 154.83)(118.0863, 961306.3)(119.0896, 46030.71)(120.0813, 41744.47)	13708736	2459475	13975706	3722627	11518323	1685340	10912432	2615521	-0.72	0.006574	0.014514	-0.23	0.451336	0.723875
COMPOUND_117_0795a4_6878123	Hilic	+	117.0795	4.69	Valine	2	20 eV: 55.0544 (100), 72.0810 (90), 118.0861 (5)	(140.0683, 4547.95)(141.0719, 355.39)(142.0829, 101.07)(257.1474, 420.83)(258.1561, 50.46)(118.0865, 395519.9)(119.0898, 21048.69)(120.0915, 1776.9)	963937.2	213159.6	972824.6	225804.5	805409.1	141052.8	861817.9	238901	-0.70	0.009622	0.020461	0.11	0.724823	0.880421
COMPOUND_119_0577a1_0233206	Hilic	+	119.0577	1.02				(239.1219, 272.34)(142.048, 2603.53)(143.0528, 93.71)(137.0949, 155.19)(120.0656, 35123.4)(121.0683, 2529.38)(122.0789, 418.6)(256.1539, 149.66)	47066.73	19694.44	39026.3	10585.3	51754.75	21862.45	63061.98	33551.39	0.47	0.05235	0.091176	0.36	0.205364	0.516497
COMPOUND_119_058a5_7017784	Hilic	+	119.0580	5.70	Threonine	2	10 eV: 74.0603 (100), 56.0493 (45), 102.0548 (15), 120.0663 (5)	(142.0474, 16760.56)(143.0509, 821.27)(144.0542, 187.1)(120.0659, 197510.62)(121.0686, 11543.4)(122.0799, 3614.87)(123.0743, 146.51)	165463.9	65594.6	166053.7	64612.81	322903.9	137878.9	474757.5	192154.1	0.87	0.00026	0.000757	0.61	0.026496	0.225166
COMPOUND_121_0741a5_1081805	Hilic	+	121.0741	5.11				(144.0634, 3840.4)(145.0653, 166.96)(146.0568, 250.57)(122.0813, 370562.62)(123.0846, 17166.64)(124.0859, 2836.67)	1143407	212425.7	1130988	258568.7	28918.18	18325.79	30239.62	24461.93	-1.52	1.93E-12	6.99E-11	-0.06	0.755951	0.895058
COMPOUND_121_9174a2_9088342	Hilic	+	121.9174	2.91				(144.9034, 86.61)(122.9245, 80129.39)(123.9283, 983.32)(124.9228, 10992.98)	129719.6	10141.67	131953.8	9374.259	154199.1	85382.39	150230	60821.11	0.57	0.032125	0.059938	-0.08	0.797761	0.913784
COMPOUND_125_0147a5_236711	Hilic	+	125.0147	5.24	Taurine	1	10 eV: 126.0217 (100), 44.049 (35)	(251.037, 1931.03)(252.0399, 150.04)(253.035, 247.28)(148.0035, 2204.68)(149.002, 105.18)(149.9993, 143.15)(273.0203, 452.84)(143.049, 930.55)(144.043, 37.72)(126.0219, 18291.66)(127.0259, 657.53)(128.0179, 1113.89)	47351.55	19360.23	51600.29	18004.49	45882.64	17275.37	47596.33	14539.52	-0.09	0.752429	0.820964	-0.08	0.818742	0.921903
COMPOUND_125_059a1_8322271	Hilic	+	125.0590	1.83				(126.0665, 5635.72)(127.0699, 285.49)(128.0708, 63.48)	15602.63	2253.826	16428.6	2838.101	14522.93	6013.422	14613.51	4998.777	-0.28	0.324841	0.425562	-0.22	0.520438	0.764408
COMPOUND_126_1157a1_6098809	Hilic	+	126.1157	1.61				(149.1024, 166.6)(127.123, 6422.11)(128.1265, 765.84)(129.1205, 65.51)	#DIV/0!	#DIV/0!	#DIV/0!	#DIV/0!	#DIV/0!	#DIV/0!	#DIV/0!	#DIV/0!	0.93	0.000491	0.001369	0.01	0.970943	0.984052
COMPOUND_128_0586a5_6120524	Hilic	+	128.0586	5.61	Unknown	4	20 eV: 83.0595 (100), 56.0495 (25), 129.0673 (5)	(151.0483, 3549.03)(152.05, 115.42)(279.1072, 639.62)(129.0659, 48212.21)(130.0688, 3422.31)(131.0701, 458.61)	70551.17	37482.76	75570.57	39786.96	60105.29	21826.27	104803.5	61818	-0.35	0.082337	0.136743	1.03	3.72E-05	0.007614
COMPOUND_129_0429a6_042503	Hilic	+	129.0429	6.04	Glutamine	1	10 eV: 130.0503 (100), 84.0443 (80), 147.0761 (20)	(152.032, 12190.3)(153.0357, 929.59)(154.0363, 162.36)(147.0765, 782716.06)(148.0799, 43904.18)(149.0813, 6153.97)(130.05, 175970.55)(131.0533, 10557.22)(132.0544, 1190.18)	1472919	384827.9	1318936	486148	2176402	676675.1	2643025	798302.3	0.78	0.000826	0.002186	0.74	0.006545	0.125256
COMPOUND_129_0433a4_067703	Hilic	+	129.0433	4.07				(259.0934, 486.05)(260.0955, 98.37)(152.0317, 3496.85)(153.0351, 184.1)(281.0756, 1300.58)(282.0784, 222.53)(283.0842, 23.12)(130.0503, 6502.85)(131.0537, 443.3)(132.0601, 139.59)(276.1226, 189.74)	37003.87	9565.398	36562.85	13972.86	22403.36	10173.96	26333.41	11226.59	-1.30	6.73E-06	2.78E-05	0.45	0.155998	0.466545
COMPOUND_129_0576a3_7972538	Hilic	+	129.0576	3.80				(130.065, 3119.85)(131.0697, 496.7)(132.0645, 72.76)	38505.15	7798.927	39437.98	7329.016	33601.07	8452.955	35000.23	8296.928	-0.55	0.047493	0.083952	-0.04	0.903147	0.956673
COMPOUND_129_0786a4_8300214	Hilic	+	129.0786	4.83	Pipecolic acid	1	20 eV: 84.0807 (100), 56.0497 (15), 130.0863 (5)	(152.0686, 3185.91)(153.0686, 458.1)(154.0779, 62.45)(130.0865, 40887.4)(131.0894, 2839.8)(132.1021, 750.97)	50224	18830.94	54354.54	35979.36	90187.93	33811.89	96746.26	37966.24	1.16	6.95E-07	3.64E-06	0.02	0.945172	0.974642
COMPOUND_129_0792a3_883771	Hilic	+	129.0792	3.88				(130.0864, 11382.58)(131.0886, 748.36)(132.0815, 846.27)	33515.98	13647.71	31502.42	10832.63	39632	16096.74	35067.28	14977.53	0.42	0.103188	0.16742	-0.16	0.591546	0.802507
COMPOUND_129_0795a3_5783715	Hilic	+	129.0795	3.58				(152.0686, 3804.12)(153.0716, 308.51)(130.0863, 442378.3)(131.0896, 29054.25)(132.0921, 2321.49)	1280243	1315689	989334	1022343	834595.2	650603.9	757126.3	871269.9	-0.47	0.072137	0.12187	0.02	0.942939	0.974529
COMPOUND_129_0796a7_045123	Hilic	+	129.0796	7.05	Lysine (m/z 147.1134)	1	10 eV: 84.0809 (100), 130.0860 (50), 147.1130 (5)	(147.1126, 524997.8)(148.116, 36577.5)(149.1181, 3297.37)(130.0864, 68245.74)(131.0898, 5096.79)(132.0916, 692.09)	810180.1	226187.8	898380.1	296455.4	933136.5	288666.4	914227.3	322274.8	0.46	0.131531	0.205699	-0.44	0.229395	0.535521
COMPOUND_129_0906a1_4882349	Hilic	+	129.0906	1.49				(259.1816, 112.71)(130.0973, 9021.15)(131.0995, 616.76)(132.1019, 721.66)	29561.58	10203.92	30292.24	8429.073	11896.46	5473.736	9951.25	5606.048	-1.23	3.73E-08	2.63E-07	-0.34	0.142265	0.448182
COMPOUND_130_1109a3_1116297	Hilic	+	130.1109	3.11	N-Acetylputrescine	1	10 eV: 72.0800 (100), 114.0905 (85), 131.1178 (80), 55.0529 (10)	(261.2328, 81.02)(131.118, 5716.52)(132.1204, 577.71)(133.1212, 75.87)	20709.25	5431.165	19526.27	5634.359	37056.57	9438.539	47440.58	14286.6	1.08	1.04E-07	6.43E-07	0.67	0.002198	0.067469
COMPOUND_131_0662a5_56081	Hilic	+	131.0662	5.56	Creatine	1	10 eV: 90.0548 (100), 132.0769 (95), 44.0490 (50), 114.0667 (4)	(154.0515, 6339.21)(155.0544, 336.91)(149.1001, 71.73)(132.0671, 83527.58)(133.0702, 5330.25)(134.0758, 884.65)	77208.74	36382.85	103340.5	42820.93	116118.6	47107.87	108806.5	37588.2	0.78	0.000666	0.001808	-0.50	0.055183	0.293093
COMPOUND_131_0697a5_482618	Hilic	+	131.0697	5.48	dublicate of creatine?			(263.1468, 45019.43)(264.1497, 4455.96)(265.15, 976.46)(266.1514, 91.97)(154.059, 117574.16)(155.0616, 5783.82)(156.0631, 725.34)(285.1287, 38911.15)(286.1315, 4264.06)(287.1343, 647.35)(288.1438, 73.12)(132.0766, 991723.94)(133.0801, 48276.17)(134.0814, 5881.67)	2045561	846157.2	2012231	655534.2	2117576	527902.1	3008951	1206934	0.16	0.515067	0.615348	0.74	0.010961	0.164558
COMPOUND_131_0704a5_6891522	Hilic	+	131.0704	5.69	dublicate of creatine?			(154.0581, 4167.23)(155.061, 316.51)(149.0982, 205.8)(132.068, 41749.95)(133.0723, 2381.34)(134.0792, 860.51)	34609.44	34475.21	21532.94	12604.37	18389.29	8791.356	35054.43	23091.85	-0.13	0.650863	0.738782	0.73	0.033525	0.247063
COMPOUND_131_0947a1_2622912	Rp	+	131.0947	1.26	Isoleucine/leucine	3	10eV:86.096(100), 30.0336(9), 132.0986(4)	(154.0835, 705.14)(155.0846, 51.47)(156.0753, 554.8)(132.1022, 400265.84)(133.1052, 28863.22)(134.1065, 2832.43)	2088195	1436837	1814852	1402946	1818966	1123822	1664952	1239844	-0.18	0.550101	0.647797	0.05	0.896495	0.954308
COMPOUND_131_0948a3_725444	Hilic	+	131.0948	3.73				(154.0843, 3224.47)(155.0843, 358.66)(156.0725, 208.28)(285.1792, 380.37)(286.1699, 110.23)(287.1695, 412.86)(132.1017, 868706.8)(133.1054, 57847.1)(134.1071, 5449.32)	3892125	707519	4065205	829590.4	3323981	646625.6	3618345	884666.9	-0.71	0.014891	0.030403	0.15	0.665018	0.844963
COMPOUND_131_0948a1_3254217	Rp	+	131.0948	1.33	duplicate of Isoleucine/leucine			(154.084, 1077.77)(155.0915, 22.18)(156.0765, 609.07)(170.057, 95.85)(132.1018, 777645.6)(133.1054, 50404.33)(134.1068, 4427.67)	3153043	994607.3	3083077	937868.9	2759434	840669.4	2842720	881897.9	-0.38	0.182871	0.270454	0.09	0.78827	0.909997
COMPOUND_131_0951a4_0280433	Hilic	+	131.0951	4.03	Leucine	1	10 eV: 86.0967 (100), 132.1021 (5)	(154.0844, 3430.3)(155.0857, 474.52)(156.0787, 87.11)(285.1768, 498.86)(286.1852, 82.63)(287.1697, 246.59)(132.1018, 605440.94)(133.1053, 39365.45)(134.1073, 3206.99)	1870090	436761.4	2027146	497141.6	1538220	534386.8	1755389	584071.9	-0.73	0.014509	0.029704	0.17	0.632037	0.828008

Compound	Column	Ion_mode	Mass	Retention_	Identification	ID_level	MSMS_spectra	CompositeSpectrum	1st trimester				3rd trimester									
									Control		Pre-eclampsia		Control		Pre-eclampsia		Time			Interaction		
									Mean	SD	Mean	SD	Mean	SD	Mean	SD	Value	raw p	FDR_p	Value	raw p	FDR_p
COMPOUND_132_0244a4_4539337	Hilic	+	132.0244	4.45	Methionine (m/z 150.0586)	1	20 eV: 56.0492 (100), 61.0108 (55), 74.0603 (15), 104.0515 (10), 45.9869 (10), 87.0254 (9)	(265.0652, 89.17)(150.0586, 49836.59)(151.0616, 3532.7)(152.0553, 2510.78)(133.0321, 5471.86)(134.0341, 337.57)(135.0287, 317.66)	34130.06	15607.68	37690.39	22493.29	102990.6	28559.31	121552.7	43618.18	1.38	2.11E-09	2.11E-08	0.15	0.515006	0.761025
COMPOUND_132_0536a6_15403	Hilic	+	132.0536	6.15	Asparagine	1	10 eV: 74.0241 (100), 87.0548 (95), 133.0610 (35), 116.0345 (25)	(155.043, 9908.62)(156.0457, 838.72)(133.0608, 31914.45)(134.0648, 1504.61)(135.0646, 538.52)	47191.83	11727.11	46687.17	13641.2	76886.07	27037.79	83233.25	24888.71	1.25	8.54E-08	5.46E-07	0.19	0.418065	0.69956
COMPOUND_135_0322a0_7497464	Hilic	+	135.0322	0.75	fragment of 2PY			(271.079, 1591.95)(153.066, 153165.66)(154.0691, 12821.9)(155.0721, 1656.51)(136.0395, 14648.16)(137.0429, 1051.18)(138.0531, 185.66)	336509.2	178787.1	296527.4	175827.6	311316.5	159380.3	513463.8	243751.8	-0.11	0.665042	0.752222	1.08	0.000834	0.045795
COMPOUND_136_0388a1_3568484	Hilic	+	136.0388	1.36	Hypoxanthine	2	10 eV: 137.0460 (100), 119.0356 (5), 110.0350 (4), 94.0399 (3)	(273.0848, 18746.45)(274.0869, 2460.75)(275.0922, 547.93)(159.0278, 121015.23)(160.0305, 8038.76)(161.0328, 611.76)(295.0668, 45767.58)(296.0694, 6148.97)(297.0724, 870.89)(298.0844, 95.27)(154.0715, 281.15)(137.0458, 403657.34)(138.0489, 23362.65)(139.0511, 1747.92)	1945179	450572.9	2063340	429922.7	1572090	477914.1	1715478	414438.3	-0.83	0.004046	0.009316	0.04	0.911712	0.959877
COMPOUND_136_0637a2_0840712	Hilic	+	136.0637	2.08	1-Methylnicotinamide	1	20 eV: 137.0699 (100), 94.0645 (60), 92.0490 (55)	(159.0535, 91.41)(137.0711, 98980.46)(138.0744, 8643.67)(139.0773, 770.57)	193383.8	88257.82	190411.7	93447.38	222314.9	90033.67	304174.7	169436	0.33	0.221768	0.315092	0.42	0.189123	0.504029
COMPOUND_137_0481a4_037293	Hilic	+	137.0481	4.04	Trigonelline	1	20 eV: 138.0555 (100), 94.0649 (70), 92.0500 (45), 78.0349 (10), 79.0409 (9)	(275.1028, 884.55)(276.1058, 170.14)(160.0368, 12125.84)(161.04, 1050.28)(162.0442, 163.15)(297.0847, 1034.85)(298.087, 185.12)(299.0939, 79.32)(138.0547, 765883.5)(139.0584, 62203.91)(140.061, 4981.41)(292.1277, 267.54)(293.1173, 96.15)	609770.2	829792.5	551571.2	952817	1378933	1350694	597695.9	876805.2	0.83	0.002301	0.005632	-0.56	0.069913	0.323961
COMPOUND_138_0419a1_0292504	Hilic	+	138.0419	1.03	Urocanic acid	1	10 eV: 139.0522 (100), 121.0378 (45), 95.0624 (5)	(139.0503, 26239.46)(140.0536, 2033.2)(141.0554, 301.66)	22182.6	14917.04	21356.02	12764.02	38387.36	19021.83	26717.4	11943.7	1.13	0.000119	0.000364	-0.61	0.064647	0.307286
COMPOUND_139_1365a0_727119	Hilic	+	139.1365	0.73				(279.2786, 263.66)(162.128, 339.24)(301.2533, 530.54)(157.17, 157.55)(140.1431, 6981.32)(141.146, 691.09)	17623.06	3270.431	18841.71	2686.747	22073.21	5172.411	20686.3	3052.885	1.12	7.12E-05	0.000227	-0.48	0.128746	0.425125
COMPOUND_140_0592a5_148521	Hilic	+	140.0592	5.15				(141.0662, 21732.08)(142.0698, 1634.69)(143.0828, 288.11)	20873.29	15773.61	17223.05	15510.56	22610.92	16281.26	27896.57	27127.15	0.17	0.514712	0.61525	0.46	0.128315	0.424949
COMPOUND_141_0794a1_2786746	Hilic	+	141.0794	1.28				(164.0684, 2417.25)(165.0722, 263.9)(166.078, 33.62)(305.1475, 118.36)(142.0866, 97285.31)(143.0898, 8370.35)(144.0926, 606.3)	5861	1854.936	8605.2	1683.105	105905	70117.49	189128.4	194126.2	0.93	2.28E-08	1.72E-07	0.46	0.009898	0.153413
COMPOUND_142_1105a1_4718053	Hilic	+	142.1105	1.47				(143.1181, 8665.2)(144.1209, 788.6)(145.1316, 115.68)	16575.87	6856.614	15862.15	4980.721	21772.5	4681.09	22980.18	6560.771	0.80	0.000953	0.002496	0.27	0.326359	0.633587
COMPOUND_143_0944a3_2846339	Hilic	+	143.0944	3.28	Proline betaine	1	20 eV: 144.1022 (100), 58.0656 (25), 84.0816 (24)	(287.1971, 2658.26)(288.1996, 283.24)(289.1945, 210.44)(166.0846, 5687.51)(167.0904, 1144.52)(168.0918, 299.66)(309.175, 324.63)(310.1771, 94.87)(144.1014, 1450867.0)(145.1051, 96817.09)(146.1077, 7669.81)	18121858	20325574	16665071	18431029	18796888	16581404	20866122	18635081	0.26	0.346151	0.447707	0.01	0.985314	0.992364
COMPOUND_143_0946a3_9029844	Hilic	+	143.0946	3.90				(144.102, 95526.16)(145.1053, 8282.14)(146.1086, 729.7)	84920.62	58767.59	89475.11	60524.12	101882.6	58280.58	224224.3	171331.9	0.31	0.133053	0.207504	0.80	0.001338	0.058548
COMPOUND_143_0948a4_9968605	Hilic	+	143.0948	5.00				(166.0856, 766.75)(144.1019, 5658.62)(145.1047, 646.35)(146.092, 539.5)	13241.63	3577.532	14448.92	4260.488	16062.25	3533.222	20616.81	5215.788	0.32	0.167358	0.250925	0.82	0.003321	0.082317
COMPOUND_145_053a4_673086	Rp	+	145.0530	4.67				(146.0603, 13883.18)(147.0634, 1509.62)(148.0552, 1409.93)	#DIV/0!	#DIV/0!	#DIV/0!	#DIV/0!	#DIV/0!	#DIV/0!	#DIV/0!	#DIV/0!	-1.11	4.56E-06	1.96E-05	-0.01	0.966437	0.982565
COMPOUND_145_0739a0_7994704	Hilic	+	145.0739	0.80				(291.1528, 624.7)(168.0636, 2169.81)(169.0683, 152.92)(170.0634, 82.51)(313.1338, 232.06)(146.0817, 155426.14)(147.0847, 10036.62)(148.0875, 999.92)(308.1867, 1425.62)	74456.79	76412.77	68000.98	74849.02	84655.36	60792.09	111955.9	114652.6	0.43	0.12291	0.194457	0.30	0.357049	0.665625
COMPOUND_145_074a0_78353864	Hilic	+	145.0740	0.78				(291.1528, 624.7)(168.0635, 2231.98)(169.0669, 127.44)(170.0595, 241.15)(313.1312, 239.43)(146.0816, 127334.28)(147.085, 8990.95)(148.0875, 1286.03)(308.1855, 1313.39)	67290.11	77161.07	65362.24	76290.01	71763.57	58440.02	92180.25	116548.3	0.51	0.07467	0.125584	0.15	0.659826	0.842895
COMPOUND_145_0848a3_482057	Hilic	+	145.0848	3.48				(146.0925, 10775.37)(147.095, 872.44)(148.096, 301.91)	54875.23	22329.65	53230.48	18976.35	19228.3	18683.94	20922.03	20082.07	-0.64	0.010617	0.022366	-0.37	0.203399	0.5149
COMPOUND_145_1106a2_9687293	Hilic	+	145.1106	2.97	dublicate of 4-aminobutyric acid betaine			(146.1178, 62740.04)(147.1211, 5312.56)(148.1232, 604.17)	174288.1	36939.47	189895.7	37498.38	150710.1	36769.53	164024.3	50375.38	-0.57	0.044081	0.078288	-0.06	0.861058	0.942428
COMPOUND_145_1109a3_0587182	Hilic	+	145.1109	3.06	4-Aminobutyric acid betaine	1	20 eV: 43.019 (100), 87.045 (76), 45.034 (42), 60.081 (23), 58.066 (20)	(146.1177, 24228.69)(147.1207, 1926.83)(148.1223, 289.17)	79084.85	15501.9	80085.07	28247.23	73538.21	23142.21	83596	39660.34	-0.46	0.123051	0.194464	0.36	0.313852	0.62054
COMPOUND_146_0942a5_80901	Rp	-	146.0942	5.81				(145.0868, 11390.1)(146.0903, 935.53)(147.0918, 159.81)	17924.58	8307.674	22270.27	9839.882	11764	1677.605	12633.13	2920.281	-1.09	7.25E-07	3.78E-06	-0.18	0.453647	0.725577
COMPOUND_147_0532a6_3011127	Hilic	+	147.0532	6.30	Glutamate	1	10 eV: 84.0438 (100), 148.0589 (25), 130.0499 (20), 102.0549 (5)	(170.0425, 4258.46)(171.0457, 401.01)(317.0898, 214.69)(148.0608, 165728.64)(149.0639, 10732.5)(150.0653, 1867.89)	143993.1	49687.64	177025	90122.76	96565.14	73286.66	88610.39	67704.38	-0.78	0.002768	0.006652	-0.43	0.154999	0.466404
COMPOUND_147_0895a3_9679167	Hilic	+	147.0895	3.97				(148.0969, 11739.22)(149.1003, 901.27)(150.11, 133.12)	16831.74	6799.892	17980.67	7843.962	25699.36	10443.44	28422.98	14522.97	0.97	7.95E-05	0.00025	-0.24	0.363561	0.668521
COMPOUND_148_0147a9_429351	Rp	+	148.0147	9.43				(297.0467, 192.78)(335.0046, 131.18)(171.0047, 162.42)(149.0234, 26552.31)(150.0265, 2354.88)	#DIV/0!	#DIV/0!	#DIV/0!	#DIV/0!	#DIV/0!	#DIV/0!	#DIV/0!	#DIV/0!	0.33	0.252432	0.348623	0.00	0.998689	0.999577
COMPOUND_148_0158a9_357669	Rp	+	148.0158	9.36				(335.0002, 155.89)(171.003, 165.6)(319.0306, 125.5)(149.0234, 31700.56)(150.0262, 2924.31)	108980.7	10317.62	109367.2	12775.93	109725.5	12099.41	108069.5	11782.21	0.05	0.869795	0.912333	-0.17	0.617807	0.816971
COMPOUND_148_0163a11_066134	Rp	+	148.0163	11.07				(186.9817, 12537.67)(187.9833, 1243.24)(149.0245, 19723.22)(150.0277, 1514.11)(151.0361, 381.97)	43074.17	12509.38	36821.28	13006.56	57888.43	14161.43	61056.93	16628.28	0.87	0.000701	0.00189	0.55	0.062799	0.307286
COMPOUND_149_0514a0_8602325	Hilic	+	149.0514	0.86				(172.0391, 386.81)(167.0917, 1189.51)(168.1002, 32.5)(150.0583, 40768.66)(151.0614, 3611.35)(152.0564, 1611.57)(316.1389, 617.56)	38407.23	10857.11	37577.63	9833.099	48986.07	12170.2	53896.67	16147.32	0.70	0.004252	0.00973	0.47	0.094988	0.36381
COMPOUND_150_1371a1_4405302	Hilic	+	150.1371	1.44	Trimethylamino N-oxide	2	10 eV: 76.0760 (100), 151.1442 (5)	(301.2922, 107.04)(151.1438, 988447.0)(152.1475, 64746.88)(153.149, 5133.22)(76.0755, 4782.93)(76.57, 48.71)	329913.2	1351085	275428.3	640911.3	257107.1	238474.1	318795.8	381445.9	0.53	0.067671	0.115803	-0.11	0.748228	0.891783
COMPOUND_152_0587a0_7483809	Hilic	+	152.0587	0.75	2PY (N-methyl-2-pyridone-5-carboxa	2	20 eV: 136.0383 (100), 92.0506 (80), 109.0772 (45), 80.0500 (10)	(305.1109, 476.23)(175.048, 4180.01)(176.0514, 301.04)(153.0662, 96785.63)(154.0694, 7426.83)(155.0723, 1060.04)(156.0705, 49.96)	317975.6	169556.9	280775.6	166017.6	296913.9	152901.9	492608.9	230300.9	-0.11	0.683975	0.768618	1.03	0.001507	0.06002
COMPOUND_152_0588a1_4972587	Rp	+	152.0588	1.50	Unknown	4	20 eV: 153.0671 (100), 110.0584 (88), 108.0452 (60), 78.0329 (33), 135.0518 (25), 70.0276 (22), 122.0228 (20)	(175.0475, 739.31)(176.0478, 38.53)(191.0166, 171.83)(153.066, 18654.11)(154.0691, 1508.13)(155.0695, 246.12)	75747.51	37421.05	61758.15	29974.59	50126.29	22210.45	84157.04	41750.7	-0.71	0.010762	0.022608	1.26	0.000201	0.018868
COMPOUND_152_1204a8_79496	Rp	+	152.1204	8.79				(327.2311, 1936.46)(328.2392, 628.12)(329.2227, 77.6)(153.1278, 16859.63)(154.1308, 1896.24)(155.1318, 5156.0)(156.1359, 408.07)	59261.6	33331.63	46612.43	30564.83	59836.54	33619.48	58130.82	33114.42	-0.06	0.841422	0.888787	0.43	0.225145	0.532591
COMPOUND_153_0195a4_71039	Hilic	+	153.0195	4.71				(307.0344, 323.75)(176.0088, 366.82)(154.0268, 6264.04)(155.0298, 373.27)(156.0258, 453.27)	15176.42	2976.822	14815.09	2494.096	17123.5	5133.696	15075.32	3146.734	0.51	0.067759	0.115864	-0.47	0.15288	0.465253
COMPOUND_155_0697a6_710648	Hilic	+	155.0697	6.71				(178.0586, 34093.75)(179.0615, 2211.69)(180.0624, 288.47)(333.1289, 7344.11)(334.1322, 1302.32)(335.1397, 116.37)(156.0769, 408987.12)(157.0801, 29212.24)(158.0819, 2546.38)	1786072	266127.5	1861860	287722.4	2181428	316421	2453531	386726.8	0.95	8.3E-05	0.000259	0.37	0.173683	0.488306
COMPOUND_157_1104a2_8601215	Hilic	+	157.1104	2.86	Pipecolic acid betaine	1	40 eV: 58.065 (100), 158.118 (33; [M + H]+), 70.065 (29), 98.095 (26), 56.049 (19), 44.049 (14), 43.041 (14), 42.034 (10)	(158.1175, 14689.48)(159.1207, 1248.7)(160.1304, 147.27)	26118.31	14862.19	32136.76	14610.08	27974.93	11688.81	42726.53	22235.13	0.28	0.257182	0.353802	0.49	0.087044	0.350026
COMPOUND_157_1471a1_1918651	Hilic	+	157.1471	1.19				(158.154, 10387.22)(159.1578, 1304.65)(160.1596, 137.15)	7944.075	1865.122	8076.935	1939.246	23824.21	11354.23	21778.35	13292.63	1.65	4.04E-11	7.76E-10	-0.21	0.397396	0.690408
COMPOUND_157_1473a7_533193	Rp	+	157.1473	7.53				(315.3019, 1890.5)(316.3056, 371.6)(317.3072, 60.4)(180.1365, 5407.75)(181.1383, 656.35)(182.137, 89.56)(337.2913, 534.66)(338.291, 103.3)(158.1546, 265100.47)(159.1578, 25891.21)(160.1603, 1801.69)	4809886	210293	4730664	162156.2	4761939	215247.2	4877709	241471	-0.19	0.517603	0.61805	0.94	0.00867	0.14883
COMPOUND_158_0581a4_680121	Rp	+	158.0581	4.68				(159.0656, 28730.7)(160.0695, 2148.3)(161.0749, 740.01)(162.0736, 38.05)	221368.8	84755.88	198982	60773.56	186885.1	47217.7	224875.5	89230.31	-0.31	0.307064	0.407969	0.63	0.083326	0.346217
COMPOUND_158_0693a6_293674	Hilic	+	158.0693	6.29	Citrulline	2	20 eV: 70.0650 (100), 43.0169 (10), 113.0712 (5)	(181.059, 820.86)(182.0618, 82.39)(176.1034, 157136.62)(177.1063, 12986.11)(178.109, 1580.96)(159.0768, 39215.78)(160.0796, 2576.78)(161.0839, 406.22)(334.1727, 159.99)	238766.6	69262.38	259104.5	91898.49	267458.6	127596.4	205973.4	92002.14	0.10	0.702493	0.783175	-0.72	0.029117	0.231966
COMPOUND_158_0941a6_5854783	Rp	-	158.0941	6.59				(157.0868, 8016.85)(158.0905, 708.24)(159.0957, 94.35)	15684.63	6120.196	12874.17	2889.073	27837.67	11716.3	22450.13	9212.804	1.41	4.35E-11	8.09E-10	-0.29	0.157538	0.468065
COMPOUND_159_0683a1_395856	Rp	+	159.0683	1.40				(177.1025, 5553.52)(178.1057, 738.71)(179.1058, 59.05)(160.0759, 6976.37)(161.0781, 764.49)(162.0798, 121.24)	45482.77	21064.18	43463.33	16081.27	38950.93	15289.36	34373.17	15620.88	-0.26	0.285932	0.384889	-0.40	0.151255	0.462181
COMPOUND_159_0689a1_5021456	Hilic	+	159.0689	1.50	Serotonin (mz 177.1022)	2	20 eV: 160.0761 (100), 132.0801 (45), 115.0547 (30)	(341.1356, 138.57)(177.1022, 84965.05)(178.1057, 10326.77)(179.1137, 945.45)(160.0759, 90335.98)(161.0791, 9965.81)(162.0827, 930.56)	390181.8	148527.9	397023.8	148667.8	323454	116303.4	276845.2	120555.2	-0.43	0.067957	0.116115	-0.46	0.087237	0.350026
COMPOUND_159_0898a4_4357047	Hilic	+	159.0898	4.44				(319.1868, 342.46)(320.1897, 92.43)(182.079, 75291.52)(183.0822, 5581.47)(184.0841, 796.14)(185.0922, 51.97)(341.169, 23823.1)(342.1724, 3559.99)(343.1739, 511.28)(344.1693, 69.88)(160.0969, 379444.0)(161.1004, 27521.81)(162.1022, 3261.7)	471302.8	648364.8	417895.1	666915.5	442486.3	506229	468704.1	672269.3	0.27	0.328462	0.429058	-0.06	0.862254	0.94262

									1st trimester				3rd trimester									
									Control		Pre-eclampsia		Control		Pre-eclampsia		Time			Interaction		
Compound	Column	Ion_mode	Mass	Retention_	Identification	ID_level	MSMS_spectra	CompositeSpectrum	Mean	SD	Mean	SD	Mean	SD	Mean	SD	Value	raw p	FDR_p	Value	raw p	FDR_p
COMPOUND_159_09a0_80897224	Hilic	+	159.0900	0.81				(319.2008, 176.35)(182.08, 1151.74)(183.0818, 218.36)(341.1727, 226.21)(160.0969, 30785.68)(161.1008, 2368.6)(162.1013, 271.59)(336.2175, 2922.77)(337.2204, 808.16)	31352.45	18330.25	32321.96	14313.38	39499.36	17907.66	57163.67	36350.72	0.54	0.027893	0.052875	0.32	0.253985	0.566051
COMPOUND_159_126a4_0211587	Rp	+	159.1260	4.02				(160.1336, 10226.85)(161.1363, 957.39)(162.1295, 135.22)	28743.08	16741.2	26197.58	10009.5	51337.21	85725.76	26752.34	14272.29	0.29	0.233347	0.328026	-0.36	0.208107	0.518913
COMPOUND_159_1264a1_9194915	Hilic	+	159.1264	1.92	5-Aminovaleric acid betaine	1	10 eV: 160.1334 (100), 60.0809 (30), 101.0609 (25)	(341.2543, 438.82)(342.2452, 119.3)(177.1633, 56.41)(160.1333, 181574.97)(161.1364, 17252.03)(162.1381, 1744.43)(163.1407, 96.94)	749199	211637.9	765049.2	253183.9	669165.7	163231.8	806496.6	268627.4	-0.50	0.041889	0.075047	0.63	0.025387	0.219937
COMPOUND_160_1096a6_332935	Rp	-	160.1096	6.33				(159.1024, 9165.55)(160.106, 725.64)(161.1084, 134.08)	17084.84	9378.355	16574.57	5177.725	13749.4	1336.556	13995.66	3716.272	-0.70	0.005746	0.012838	0.16	0.574884	0.79068
COMPOUND_160_1098a6_856494	Rp	-	160.1098	6.86				(159.1024, 19108.61)(160.1061, 1882.38)(161.1072, 239.18)	20932.62	10954.83	18802.02	4009.22	22218.14	12731.54	19129.67	5716.444	0.11	0.709556	0.787658	-0.15	0.678187	0.851748
COMPOUND_160_1213a6_7415733	Hilic	+	160.1213	6.74				(183.1109, 595.51)(161.1288, 75614.12)(162.132, 6045.43)(163.1338, 787.01)	47896.77	28829.72	55566.24	35238.38	54003.79	40480.67	79282.91	58850.65	0.04	0.818224	0.872049	0.45	0.032582	0.244873
COMPOUND_161_1052a4_6681848	Hilic	+	161.1052	4.67	L-Carnitine	1	10 eV: 162.1130 (100), 103.0391 (15), 60.0811 (15)	(323.2164, 694.27)(324.2295, 254.54)(325.206, 273.48)(184.0946, 28661.07)(185.0988, 2437.93)(186.0981, 430.18)(345.201, 678.05)(346.1981, 271.11)(347.1853, 225.2)(162.1128, 1180400.0)(163.1165, 84337.64)(164.1182, 11723.16)(165.1204, 627.14)	15620755	2296013	15686948	2771683	9735898	1715885	12387019	4095732	-1.53	2.58E-08	1.89E-07	0.69	0.015419	0.185682
COMPOUND_161_1055a5_3846574	Hilic	+	161.1055	5.38				(184.0946, 596.14)(162.1126, 8253.83)(163.116, 712.81)(164.1243, 86.26)	11269.83	5101.885	11362.29	4482.939	9010.5	3328.291	8929.061	4287.766	-0.62	0.028978	0.054747	-0.14	0.681296	0.853001
COMPOUND_162_0674a7_166878	Rp	+	162.0674	7.17				(163.0758, 22142.65)(164.079, 2339.11)(165.0853, 322.73)	60017.23	24612.94	54521.98	21525.12	74084.14	31695.71	59465.66	21709.75	0.47	0.117742	0.187521	-0.26	0.462826	0.731428
COMPOUND_162_0677a5_95999	Rp	+	162.0677	5.96				(185.0578, 607.24)(186.0624, 38.16)(180.102, 944.06)(181.1057, 146.39)(163.0753, 14377.15)(164.0788, 1666.86)(165.0923, 289.49)	37579.1	11163.32	38401.39	11998.57	36641.33	13697.4	37532.1	12940.22	-0.12	0.696862	0.77844	-0.08	0.820648	0.922833
COMPOUND_162_0682a7_1922607	Rp	+	162.0682	7.19				(163.0758, 25239.6)(164.0792, 2974.47)(165.0891, 585.97)	62121.33	25516.99	63412.45	22240.73	79000	31692.96	62447.17	22599.83	0.55	0.055634	0.096598	-0.23	0.492686	0.748671
COMPOUND_162_0684a8_399502	Rp	+	162.0684	8.40				(363.0926, 144.05)(163.0758, 22784.43)(164.0791, 2964.67)(165.0894, 320.91)	46408.44	17770.79	36990.73	20098.19	51437.57	21782.33	43506.26	19047.61	0.38	0.201753	0.292749	0.02	0.951884	0.980085
COMPOUND_162_0688a8_3108425	Rp	+	162.0688	8.31				(363.1095, 141.81)(163.0756, 36728.94)(164.0792, 4041.89)(165.0839, 371.92)	67031.43	29380.55	54381.62	32425.66	77139.93	35848.18	66790.84	31094.85	0.29	0.323658	0.424754	0.17	0.629931	0.827657
COMPOUND_162_1051a6_3720818	Rp	+	162.1051	6.37				(163.1121, 26504.05)(164.1157, 3107.88)(165.1227, 332.44)	37231.89	17771.51	35429.87	14838.65	32069	3701.655	32497.44	13884.15	-1.24	3.86E-08	2.72E-07	-0.03	0.914331	0.961085
COMPOUND_162_1262a5_6244516	Rp	+	162.1262	5.62				(185.1168, 1229.52)(186.1173, 148.13)(187.1083, 84.44)(180.1601, 1887.49)(181.1615, 197.99)(182.1629, 24.7)(163.1333, 11266.86)(164.1372, 1075.71)(165.1305, 190.24)	66818.94	8352.836	62576.74	6310.16	61503.36	7713.786	64478.75	8104.658	-0.70	0.021584	0.042131	0.90	0.013152	0.173893
COMPOUND_163_0225a5_6991754	Hilic	+	163.0225	5.70				(181.059, 1007.55)(164.0296, 10013.45)(165.0338, 613.42)(166.0357, 143.67)(344.0802, 157.63)	34791.3	7220.134	38165.76	8950.592	42577.5	10286.61	49504.35	10446.24	0.76	0.002601	0.006305	0.27	0.346002	0.651869
COMPOUND_164_0476a4_975613	Hilic	+	164.0476	4.98	Tyrosine (m/z 182.0811)	1	10 eV: 136.0759 (100), 165.0551 (70), 123.0442 (25), 147.0444 (15), 182.0821 (10)	(329.1157, 343.96)(182.0816, 67949.21)(183.0849, 7110.25)(184.0898, 1279.85)(185.0851, 80.13)(165.0546, 13304.5)(166.0584, 1347.35)(167.0601, 163.57)	293362.4	56807.66	330078.2	80870.94	322662.1	52006.98	316936.7	82490.42	0.49	0.103933	0.168387	-0.68	0.061113	0.302474
COMPOUND_164_0834a5_493956	Rp	+	164.0834	5.49				(329.1779, 2813.53)(187.0758, 581.16)(165.0914, 13299.32)(166.0938, 1588.53)	19711.59	6711.891	21950.55	10424.1	29925.25	13406.95	27887.27	13279.19	-0.53	0.028711	0.054288	-0.39	0.177227	0.491826
COMPOUND_164_1203a8_410994	Rp	+	164.1203	8.41				(165.1279, 27714.15)(166.1313, 3592.68)	35214.58	15199.91	34954.75	12772.32	34411.33	4260.767	39523.29	10381.88	-1.14	5.09E-07	2.74E-06	-0.10	0.673133	0.848765
COMPOUND_165_0462a6_005415	Hilic	+	165.0462	6.01				(188.0354, 18877.94)(189.0387, 1348.49)(190.0325, 903.43)(353.0813, 1087.1)(166.0535, 15283.03)(167.0559, 907.31)(168.0499, 835.69)(169.0509, 34.21)	111816.4	29835.73	118104.6	40105.43	34860.79	33283.22	31573.8	37192.45	-1.17	1.43E-06	6.96E-06	-0.34	0.192796	0.505089
COMPOUND_165_0654a1_4190111	Hilic	+	165.0654	1.42				(331.1397, 413.73)(332.1487, 73.14)(188.0552, 708.89)(166.0723, 24192.33)(167.0755, 2107.92)(168.0779, 253.98)	60919.34	12610.77	62054.35	14436.27	129166.1	25643.78	121897.4	24490.95	1.66	1.58E-12	6.22E-11	-0.12	0.599429	0.805439
COMPOUND_165_0789a1_9692475	Rp	+	165.0789	1.97	Normetanephrine	1	10eV: 120.081(100), 103.0549(5), 166.093(4)	(331.1524, 60.73)(204.0338, 252.77)(166.0863, 664502.0)(167.0899, 62677.99)(168.0921, 5057.16)	5064084	925003.3	5061087	1008721	4985224	1080000	5081637	1151667	-0.03	0.924058	0.950653	0.01	0.97179	0.984467
COMPOUND_165_0792a3_5999737	Hilic	+	165.0792	3.60	Phenylalanine	2	10 eV: 120.0814 (100), 166.0876 (5)	(331.1578, 151.94)(188.0682, 8406.81)(189.0706, 970.98)(190.0715, 114.71)(353.1625, 272.18)(166.0861, 795099.6)(167.0897, 75220.42)(168.0921, 6656.49)(348.1968, 220.12)	3202594	665554.3	3317060	666370	3149198	823712.1	3219243	838379.3	0.04	0.897854	0.933072	-0.23	0.486477	0.747178
COMPOUND_166_0629a4_513383	Rp	-	166.0629	4.51				(201.025, 110.89)(165.0555, 10954.59)(166.0582, 1218.29)(167.0706, 154.95)(211.0615, 685.14)(331.1223, 747.58)	20162.82	10549	20692.4	11681.36	22361.38	7649.277	18871.29	9605.785	0.30	0.287381	0.386378	-0.59	0.082054	0.343638
COMPOUND_166_1364a8_912118	Rp	+	166.1364	8.91				(371.2352, 3531.2)(372.2383, 966.78)(373.2376, 207.53)(167.1436, 44370.44)(168.147, 5615.04)(169.1533, 796.94)	106125.5	35473.87	108805.2	40774.07	65125.5	35708.67	67663.27	35743.13	-1.29	2.76E-09	2.64E-08	-0.10	0.654348	0.841269
COMPOUND_168_9786a5_236611	Hilic	+	168.9786	5.24				(169.986, 6457.63)(170.9883, 262.78)(171.982, 337.98)	25426.04	4296.223	27767.33	4234.026	25487.38	2870.982	25942.09	3112.925	-0.14	0.621305	0.713139	-0.41	0.238046	0.543692
COMPOUND_169_0852a6_19915	Hilic	+	169.0852	6.20	1-Methylhistidine	2	20 eV: 124.0871 (100), 83.0600 (45), 96.0673 (15), 170.0909 (5)	(339.1825, 181.29)(192.0745, 4364.2)(193.078, 400.18)(194.0772, 75.56)(170.0925, 124629.09)(171.0955, 10290.07)(172.0974, 1233.98)	118778.4	35522.58	128596.5	40947.36	192434.4	123944.9	246632.4	186773.7	0.71	0.001309	0.003335	0.50	0.046305	0.273966
COMPOUND_169_0852a6_288577	Hilic	+	169.0852	6.29				(339.1848, 58.04)(192.0745, 1128.6)(193.0759, 39.77)(194.078, 37.63)(170.0926, 105897.48)(171.0956, 8616.52)(172.098, 1152.79)	72131.91	20648.94	80276.02	27092.84	111485.6	134476.3	142686.2	204807.6	0.06	0.820856	0.873202	0.44	0.165508	0.47785
COMPOUND_169_0852a6_804594	Hilic	+	169.0852	6.80	3-Methylhistidine	2	20 eV: 96.0684 (100), 109.0756 (25), 68.0489 (15), 126.1045 (12), 125.0698 (9), 82.0641 (8), 170.0949 (5)	(192.0744, 342.53)(361.1589, 202.93)(170.0925, 280469.06)(171.0957, 21349.58)(172.0984, 2053.88)	206835.9	273606.1	193946.9	288146.5	247750.1	273858.6	223370.4	230947.8	0.02	0.935699	0.957815	0.22	0.498762	0.753329
COMPOUND_171_1136a1_2169836	Hilic	+	171.1136	1.22				(172.1197, 4131.97)(173.1219, 492.03)(174.1246, 81.87)	10785.59	2533.737	12527.24	4071.462	13016.86	3552.247	13092.75	3402.5	0.66	0.019837	0.039186	-0.39	0.228651	0.535521
COMPOUND_173_1163a1_8126739	Hilic	+	173.1163	1.81				(174.124, 10491.71)(175.1277, 1113.12)(176.1333, 137.12)	17933.83	9247.958	19511.69	10177.22	13361.1	9403.219	14880.53	13247.99	-0.78	0.001992	0.004957	-0.07	0.796948	0.91372
COMPOUND_173_1165a1_712097	Hilic	+	173.1165	1.71				(174.1237, 14110.3)(175.1264, 1197.38)(176.1342, 312.36)	22227.04	9475.665	24122.67	10805.14	20145.09	11458.35	18593.25	19163.19	-0.61	0.02561	0.048959	-0.12	0.70792	0.868783
COMPOUND_173_142a0_751595	Hilic	+	173.1420	0.75				(196.1269, 2968.49)(197.1284, 737.46)(369.2828, 769.4)(191.177, 85.18)(174.1491, 15507.66)(175.1524, 1962.37)(176.1573, 288.09)	68494.89	9542.531	72112.59	9278.417	75200.57	11813.8	76447.28	11499.35	0.63	0.033006	0.061346	-0.25	0.471471	0.734155
COMPOUND_173_9946a1_3492537	Hilic	+	173.9946	1.35				(196.9829, 405.13)(175.0016, 11625.43)(176.0049, 829.52)(176.9997, 933.86)	32786.55	8278.383	35416.2	7580.258	25612.43	9477.203	27606.46	7531.162	-0.85	0.002636	0.006377	-0.09	0.78835	0.909997
COMPOUND_174_1119a7_036021	Hilic	+	174.1119	7.04	Arginine	2	20 eV: 70.0653 (100), 60.0557 (18), 116.0705 (10), 130.0972 (5)	(197.1018, 326.55)(175.1191, 15334.04)(176.1221, 1289.46)(177.1236, 201.35)	56353.7	19961.62	65345.74	21906.9	57756.57	16232.47	54302.29	20706.56	0.24	0.41251	0.516958	-0.72	0.04533	0.273371
COMPOUND_174_1125a6_8896704	Hilic	+	174.1125	6.89				(349.2314, 33523.76)(350.2338, 5251.39)(351.237, 687.63)(352.2402, 82.54)(197.101, 10971.2)(198.1051, 836.7)(199.1048, 157.74)(371.213, 9891.42)(372.2154, 1824.33)(373.2167, 372.71)(374.2247, 42.28)(175.119, 543532.8)(176.1221, 38576.66)(177.1238, 2989.03)	1593384	356307.3	1779197	393144	1594931	372088.5	1484319	445845.4	0.11	0.716727	0.792741	-0.82	0.021875	0.209625
COMPOUND_174_1371a6_616072	Hilic	+	174.1371	6.62				(175.1443, 13620.85)(176.1468, 1318.16)(177.1487, 136.28)	22222.81	9234.161	24380.15	9559.682	25391.57	9248.966	32413.74	17151.06	0.29	0.134725	0.209386	0.28	0.208884	0.518913
COMPOUND_174_1405a10_760002	Rp	+	174.1405	10.76				(349.2971, 1613.9)(175.1485, 27381.64)(176.1515, 4145.46)(177.1568, 343.11)	24859.03	8694.448	25726.78	6867.569	32415.82	21379.89	31609.07	15366.13	0.57	0.030894	0.057833	-0.20	0.522184	0.765284
COMPOUND_174_1421a10_785122	Rp	+	174.1421	10.79				(349.2939, 5801.83)(175.1481, 20268.54)(176.1504, 2560.58)(177.1512, 320.7)	44591.88	15253.67	38973.75	16900.73	44303.73	20611.09	44990.19	18982.11	0.64	0.010111	0.02144	-0.48	0.098678	0.368908
COMPOUND_175_0589a3_7198732	Hilic	+	175.0589	3.72				(351.1146, 406.37)(176.0662, 3448.67)(177.0687, 280.42)(178.0824, 77.13)(368.1551, 298.8)	19247.15	3154.693	21700.73	4287.611	17544.43	2932.234	18404.49	2895.63	-0.46	0.117689	0.187521	-0.21	0.539277	0.776408
COMPOUND_175_0637a5_027729	Rp	+	175.0637	5.03	3-Indoleacetic acid	1	10eV: 130.0655(100), 176.0727(15), 125.9833(10)	(389.0817, 434.68)(198.0535, 2368.45)(199.0559, 542.27)(200.0572, 30.32)(214.0204, 931.63)(215.0264, 218.22)(193.0963, 834.76)(194.0908, 66.18)(195.0897, 769.59)(176.0711, 89578.04)(177.0744, 11368.85)(178.0775, 1006.62)	187765.8	86945.17	170070.2	56397.64	185779.6	45760.3	164957	64541.38	0.16	0.597043	0.691669	-0.29	0.410581	0.694731
COMPOUND_176_0175a6_157084	Hilic	+	176.0175	6.16				(375.0165, 438.59)(177.0249, 5799.82)(178.0273, 319.22)(179.0291, 156.75)	17849.04	3149.731	19297.52	4381.403	20570.79	4435.395	22263.25	4981.187	0.56	0.041003	0.073753	0.02	0.957257	0.980661
COMPOUND_176_0804a6_238077	Hilic	+	176.0804	6.24				(199.069, 4621.42)(200.0722, 496.08)(201.0692, 58.8)(375.1469, 202.48)(177.0868, 13354.3)(178.0901, 915.6)(179.0886, 202.36)	6143.6	2252.667	5760.75	1537.906	26748.23	8120.599	26275.15	10311.31	1.37	4.83E-14	5.44E-12	0.04	0.788025	0.909997
COMPOUND_178_0995a9_289115	Rp	+	178.0995	9.29				(357.2006, 11768.55)(358.2056, 2802.74)(379.1928, 754.06)(380.1835, 243.88)(179.1069, 10635.52)(180.1098, 1195.87)(181.1063, 474.64)	59466.66	21699.02	55114.91	29793.44	25307	12076.38	36222.22	17314.02	-1.50	8.1E-12	2.12E-10	0.25	0.238465	0.543832
COMPOUND_179_0583a3_296708	Rp	+	179.0583	3.30	Hippuric acid	1	10eV: 105.0331(100), 77.0392(10), 138.9585(2)	(397.0716, 8184.73)(202.048, 13885.39)(203.0508, 1508.34)(204.0526, 192.34)(381.1031, 2244.18)(180.0657, 30250.06)(181.0695, 3349.55)(182.0739, 612.1)(183.0828, 27.35)	156544.2	127649.1	177360.8	165769.6	314622.3	204266.5	274183.8	441698.2	0.82	0.003576	0.008353	-0.47	0.148381	0.456493
COMPOUND_180_0461a1_5157193	Hilic	+	180.0461	1.52				(361.1115, 1875.23)(203.035, 4299.3)(204.0381, 262.21)(205.0317, 248.91)(383.0924, 385.3)(181.0532, 12688.92)(182.0556, 809.33)(183.0512, 641.09)	32068.22	6235.164	31415.23	6717.745	37190.36	7895.035	45073.42	11216.84	0.67	0.003709	0.008647	0.64	0.018085	0.195632
COMPOUND_180_0648a2_92785	Rp	+	180.0648	2.93				(203.0546, 1865.59)(204.0574, 270.3)(205.0645, 27.46)(181.0723, 53859.27)(182.0757, 5763.91)(183.0756, 528.46)	41371.31	35293.16	39998.05	21305.85	98953.07	81084.71	100412.6	80513.61	1.05	1.76E-05	6.65E-05	-0.35	0.182275	0.495754
COMPOUND_180_0649a1_7921726	Rp	+	180.0649	1.79				(399.0847, 507.43)(203.0541, 6643.8)(204.0581, 666.15)(205.0614, 93.0)(198.0977, 3313.13)(199.1014, 330.46)(181.0722, 57252.61)(182.0753, 5447.42)(183.0775, 597.62)	167288	168685	197493	180322	352400.2	197942	277510.4	485912.5	1.17	2.06E-05	7.62E-05	-0.87	0.004731	0.106402

									1st trimester				3rd trimester									
									Control		Pre-eclampsia		Control		Pre-eclampsia		Time			Interaction		
Compound	Column	Ion_mode	Mass	Retention_	Identification	ID_level	MSMS_spectra	CompositeSpectrum	Mean	SD	Mean	SD	Mean	SD	Mean	SD	Value	raw p	FDR_p	Value	raw p	FDR_p
COMPOUND_180_0649a2_8124936	Rp	+	180.0649	2.81	Paraxanthine	1	10eV: 181.0709(100), 124.0497(15), 77.967(5), 42.0308(1.5)	(203.054, 1957.89)(204.0553, 141.35)(181.0726, 68232.86)(182.0756, 6317.74)(183.0763, 538.56)	175012.5	181781.8	170105.1	144207.9	237832.9	210050.5	145755.3	142679.6	0.69	0.0138	0.028459	-0.76	0.020252	0.199159
COMPOUND_180_0652a2_3989115	Rp	+	180.0652	2.40				(399.0836, 899.77)(203.0539, 4027.42)(204.0571, 359.5)(181.0723, 275140.12)(182.0752, 23901.78)(183.0772, 2138.58)	139607.8	117585.6	156233	122502.8	269870.4	164228.6	183419.7	303147.7	1.06	0.000187	0.000559	-0.96	0.003096	0.079218
COMPOUND_180_1157a6_7009764	Rp	+	180.1157	6.70				(203.1059, 1030.19)(204.1044, 199.55)(181.1232, 16216.1)(182.1263, 1944.2)(183.1357, 482.16)	39885.42	15054.65	39544.72	16717.44	25155.8	15609.17	26444	13759.8	-1.24	3.7E-08	2.62E-07	-0.10	0.667565	0.846185
COMPOUND_181_0854a6_0349765	Hilic	+	181.0854	6.03				(363.1674, 153.21)(204.0703, 71.17)(182.0925, 26466.98)(183.0957, 2407.42)(184.0983, 373.77)	23672.94	26202.79	26664.71	23211	18175	13612.53	31905.27	21895.04	-1.11	3.13E-06	1.39E-05	-0.06	0.822181	0.922833
COMPOUND_182_0736a7_964954	Rp	+	182.0736	7.96				(183.0809, 35081.98)(184.084, 5103.46)(185.0884, 894.72)	71385.23	29963.26	56383.52	29387.98	82802.14	40825.25	70528.19	29706.34	0.39	0.192379	0.281323	0.03	0.9233	0.965307
COMPOUND_182_0948a5_4959054	Rp	+	182.0948	5.50				(205.0851, 2071.04)(206.0851, 245.2)(200.1295, 2048.45)(183.1021, 9117.99)(184.1043, 1013.72)(185.0898, 292.87)	19014.48	7326.411	22982.15	12335.54	36693.67	8835.101	30383.64	13761.5	-0.71	0.004231	0.009712	-0.23	0.424466	0.704939
COMPOUND_182_1054a0_7720168	Hilic	+	182.1054	0.77	no msms			(365.229, 585.7)(387.188, 522.05)(183.1132, 9611.53)(184.1136, 1433.97)(185.103, 499.13)(382.2559, 473.75)	10701.5	3708.421	11099.53	4922.505	12996.8	5306.559	22699	11123.12	-0.23	0.260733	0.357595	1.50	5.58E-08	0.000126
COMPOUND_183_0659a10_258124	Rp	+	183.0659	10.26				(222.0197, 127.15)(184.0741, 18951.51)(185.0776, 1220.49)(186.0831, 241.15)(384.1897, 305.28)	89557.02	8099.762	90011.67	8785.237	69119.07	8495.189	71241.86	10676.73	-1.52	4.7E-10	5.74E-09	0.09	0.7188	0.876415
COMPOUND_183_0662a10_140341	Rp	+	183.0662	10.14				(367.143, 204.38)(184.0739, 35737.41)(185.0771, 2288.61)(186.0781, 530.2)	154455.3	18862.58	149769.2	30214.37	99520.36	19738.19	103132.4	23331.83	-1.55	1.69E-09	1.75E-08	0.22	0.400334	0.690408
COMPOUND_183_0663a10_559524	Rp	+	183.0663	10.56				(184.074, 11477.26)(185.0767, 908.39)(186.0797, 175.05)	32578.89	6903.901	33013.33	7630.033	13323.88	2364.051	14646.74	2986.511	-1.55	5.33E-13	2.86E-11	-0.03	0.896441	0.954308
COMPOUND_183_0663a1_0600661	Hilic	+	183.0663	1.06	Phosphocholine	2	10 eV: 184.0733 (100), 86.0966 (15)	(184.0735, 18062.77)(185.0777, 1208.19)(186.0771, 273.62)	89152.09	21334.42	101458.3	18719.13	45740.57	11775.54	46801.2	12877.07	-1.34	7.52E-10	8.73E-09	-0.43	0.051292	0.283822
COMPOUND_183_0666a10_631687	Rp	+	183.0666	10.63				(184.074, 10788.05)(185.0768, 767.26)(186.0786, 249.14)	29950.69	5942.219	30377.71	5798.389	14882.43	2852.957	15953.35	3254.281	-1.42	2.96E-11	6.17E-10	-0.12	0.556281	0.781554
COMPOUND_183_1623a0_7821191	Hilic	+	183.1623	0.78		4	10 eV: 102.0916 (100), 184.1686 (60), 128.1077 (12), 84.081 (10)	(367.3288, 270.57)(184.1697, 25207.48)(185.1732, 3375.01)(186.1853, 381.75)	74998.64	7757.117	79859.09	10052.65	78103.86	8856.204	77706.93	8401.267	0.38	0.192173	0.281205	-0.60	0.087933	0.350486
COMPOUND_184_1212a2_6085315	Rp	+	184.1212	2.61	no msms			(185.1289, 11922.88)(186.1321, 1202.92)(187.143, 279.18)	28702.53	12291.72	24245.24	8456.348	20498.21	7503.059	37059.5	15507.18	-0.64	0.01662	0.033477	1.59	2.87E-06	0.003234
COMPOUND_184_1468a8_186916	Rp	+	184.1468	8.19				(207.1385, 5686.95)(208.1382, 1278.36)(202.1809, 2051.52)(203.1819, 492.89)(204.1739, 228.37)(185.154, 8878.81)(186.1573, 1070.65)(187.1489, 903.53)	41938.34	6148.01	38990.43	7299.021	38580.79	4099.841	38893.86	6958.641	-0.47	0.09962	0.162099	0.41	0.225105	0.532591
COMPOUND_185_1054a3_7405825	Hilic	+	185.1054	3.74				(208.0957, 1410.36)(203.1479, 152.79)(186.1128, 41647.26)(187.1161, 4803.88)	86605.83	49263.37	84119.35	21538.66	80433.64	29727.96	86149.86	30528.06	-0.27	0.262339	0.359141	0.21	0.460656	0.729797
COMPOUND_186_051a2_254241	Hilic	+	186.0510	2.25				(373.1106, 211.45)(187.0581, 14172.18)(188.0614, 981.26)(189.0595, 197.9)	46713.3	17963.78	43215.89	17790.28	29594.14	10243.98	33474.91	12173.18	-0.95	1.89E-06	8.84E-06	0.51	0.016467	0.189627
COMPOUND_186_1251a7_807181	Rp	-	186.1251	7.81				(185.1182, 24697.62)(186.1213, 3026.49)(187.1254, 535.53)(231.1317, 57.95)(371.2418, 927.72)(372.2434, 203.41)	27946.72	19136.21	30396.17	16556.16	24120.5	10452.43	21013.63	11335.31	0.00	0.999441	0.999965	-0.73	0.026284	0.224785
COMPOUND_187_0278a6_014426	Hilic	+	187.0278	6.01				(375.0652, 1656.56)(376.0667, 188.57)(377.0624, 127.47)(210.0172, 3206.02)(211.0203, 214.52)(212.0138, 372.38)(397.0456, 1181.82)(398.0476, 217.04)(399.041, 165.99)(188.0354, 16087.78)(189.0387, 1161.15)(190.0325, 739.17)	65316.09	17010.83	70001	23596.26	24494.43	17270.8	22751.89	19528.13	-1.17	1.46E-06	7.08E-06	-0.37	0.164398	0.47785
COMPOUND_187_0636a3_785936	Hilic	+	187.0636	3.79	Tryptophan (mz 205.0976)	2	10 eV: 146.0598 (100), 188.0721 (50), 118.0650 (30), 144.0804 (30), 170.0587 (10)	(375.1458, 153.85)(210.0532, 604.23)(205.0974, 440130.62)(206.1008, 50505.11)(207.1033, 4483.31)(188.071, 143724.73)(189.0742, 18563.2)(190.0773, 1653.67)(392.1586, 154.64)	2796544	343687.4	2856336	506354.3	2304024	344382.6	2397651	566363.4	-1.01	0.000303	0.000873	0.12	0.709479	0.869758
COMPOUND_187_0639a2_564372	Rp	+	187.0639	2.56	Tryptophan (205.0976)			(205.0978, 644452.5)(206.1013, 73979.02)(207.1039, 6671.9)(208.1058, 514.37)(188.0714, 229185.61)(189.0744, 27156.44)(190.0769, 2432.75)	5645896	781598	5616848	970268.5	4738035	482281	4753862	1123508	-0.92	0.000768	0.002052	0.05	0.87163	0.945212
COMPOUND_187_0642a2_0999832	Rp	+	187.0642	2.10				(205.0979, 272103.06)(206.1011, 32757.86)(207.1034, 3051.91)(208.1057, 201.16)(188.0711, 86773.01)(189.0744, 10498.27)(190.0766, 1152.76)(191.0757, 50.31)	4048746	598970.6	4027903	760220.3	3415298	402566.7	3415608	822209.1	-0.88	0.002184	0.005381	0.08	0.80458	0.917422
COMPOUND_187_1684a6_1041994	Hilic	+	187.1684	6.10				(397.3411, 113.69)(188.1761, 7794.79)(189.179, 834.88)(190.1801, 87.63)	22008.17	7993.375	25931.63	8320.817	18393	4450.978	22011.86	9155.733	-0.42	0.142551	0.218998	-0.08	0.807437	0.917894
COMPOUND_188_0661a1_5741142	Hilic	+	188.0661	1.57				(399.1185, 346.57)(189.0737, 22285.49)(190.0772, 1622.76)(191.0777, 369.77)	19875.6	16482.35	16207.86	13173.93	13803	8140.424	18056.88	11109.48	-0.17	0.548497	0.646371	0.54	0.112637	0.398247
COMPOUND_188_1048a5_7491193	Rp	-	188.1048	5.75				(223.0679, 66.07)(187.0974, 9655.9)(188.0993, 893.74)(189.1033, 226.63)	15972.28	5530.807	17060	4220.602	14023.31	2446.397	13002.18	2389.997	-0.52	0.04712	0.083422	-0.70	0.024607	0.219303
COMPOUND_188_1163a5_572104	Hilic	+	188.1163	5.57				(211.1057, 7095.35)(212.1078, 841.69)(213.115, 384.93)(399.2307, 268.83)(189.1238, 7122.42)(190.1253, 665.93)(191.1268, 450.9)	23157.81	4357.623	23312.13	4207.297	28977.29	6818.023	28041.68	5771.653	0.96	0.000443	0.001243	-0.12	0.699099	0.862515
COMPOUND_188_1277a6_7667017	Hilic	+	188.1277	6.77				(377.2644, 214.88)(211.1173, 1501.54)(212.1215, 282.18)(213.1257, 141.95)(399.2462, 270.41)(189.135, 65637.16)(190.1379, 5876.29)(191.1374, 601.48)	98236.75	40298.06	109128.7	52353.61	139994.6	48366.49	132408.1	53187.17	0.76	0.002569	0.00624	-0.27	0.329775	0.635357
COMPOUND_188_1409a8_30797	Rp	-	188.1409	8.31				(301.1164, 815.94)(187.134, 94640.9)(188.1373, 10561.01)(189.1388, 1271.74)	45764.64	58193.98	40460.11	23449.42	39513.86	15578.5	29371.04	15736.63	0.11	0.682415	0.767632	-0.76	0.015249	0.184632
COMPOUND_188_141a7_952267	Rp	-	188.1410	7.95				(301.1203, 259.06)(223.1003, 239.99)(187.1338, 13015.2)(188.1371, 1241.73)(189.139, 183.72)	19161.58	8303.133	21655.78	10566.78	16487.45	6056.366	21786.98	10219.07	-0.25	0.317489	0.419347	0.34	0.252964	0.565176
COMPOUND_188_1525a6_6988297	Hilic	+	188.1525	6.70	Trimethyllysine	1	10 eV: 189.10600 (100), 130.0856 (70), 60.0808 (30), 84.0815 (25), 144.1380 (5)	(211.1415, 339.59)(189.16, 68067.77)(190.1632, 7664.21)(191.1657, 782.71)	116726.1	38221.96	115199.7	39349.35	147466.7	77155.9	143304.2	42579.45	0.33	0.220994	0.3145	0.36	0.256712	0.56678
COMPOUND_189_0793a5_7895746	Rp	+	189.0793	5.79	Indole-3-methyl acetate	1	10eV: 130.065(100), 172.0743(15), 190.0879(10)	(379.1624, 155.27)(380.1534, 61.17)(417.1138, 6758.56)(212.0693, 8102.07)(213.0714, 1225.37)(214.0667, 93.18)(228.0361, 7693.6)(229.0383, 1141.48)(190.0873, 346608.97)(191.0905, 40858.31)(192.0931, 3674.0)	252617.4	247863.2	230082.2	247501.8	245447.6	136249.3	219368	252676.5	0.04	0.848787	0.894514	-0.14	0.613647	0.816343
COMPOUND_189_1369a0_8433127	Hilic	+	189.1369	0.84				(379.2851, 1498.86)(401.2595, 800.22)(207.1758, 172.65)(190.1433, 3616.2)(191.1467, 297.5)(192.1381, 6439.36)(193.1426, 1215.66)(396.2964, 158.83)	24836.41	9753.565	24525.68	8856.062	23579.45	6332.437	23932.21	9442.937	0.30	0.263111	0.35998	-0.05	0.886316	0.952737
COMPOUND_190_1003a6_4029007	Rp	+	190.1003	6.40				(381.2096, 659.74)(382.215, 233.88)(213.0898, 828.89)(191.1073, 9197.58)(192.1112, 1700.9)(193.121, 199.53)	18614.3	6089.544	21579.78	8679.977	21820.27	8751.073	17735.61	7503.137	0.47	0.080935	0.135011	-0.29	0.368076	0.670094
COMPOUND_190_1205a5_4844217	Rp	-	190.1205	5.48				(225.0897, 207.2)(189.1132, 14013.18)(190.1163, 1099.68)(191.1188, 159.16)(493.2249, 177.35)(494.2333, 96.06)	18694.91	9682.859	22275.94	8238.36	15065	1915.122	14523.5	4271.273	-0.90	5.71E-05	0.000187	-0.24	0.336549	0.640666
COMPOUND_190_1575a7_339925	Rp	+	190.1575	7.34				(213.1484, 1943.52)(214.1522, 378.97)(208.1904, 652.92)(209.196, 51.47)(210.1927, 21.57)(191.1646, 12108.44)(192.1674, 1743.39)(193.1704, 187.02)	109031.7	15965.69	101528.5	13163.7	105503.9	11245.67	104651.8	14631.32	-0.09	0.733424	0.806204	0.32	0.325885	0.633587
COMPOUND_191_0172a6_30662	Hilic	+	191.0172	6.31				(383.0415, 3096.68)(384.0452, 406.83)(385.0513, 76.7)(214.0067, 1595.82)(215.0109, 223.91)(405.0238, 651.85)(406.0368, 134.43)(192.0244, 5809.78)(193.0254, 138.51)(194.0306, 79.84)	28784.04	7629.609	34510.39	9692.385	19943.54	8479.519	19842.12	9287.351	-0.86	0.000623	0.001698	-0.49	0.084838	0.346484
COMPOUND_191_0584a1_7337179	Rp	+	191.0584	1.73	N-Acetyl-L-phenylalanine (m/z 209.0	1	10eV: 136.758(100), 46.0282(85), 192.0659(60), 94.0655(60), 209.1328(20)	(383.119, 1035.68)(209.0925, 14068.61)(210.0961, 1656.15)(211.1011, 269.39)(192.0662, 2100.69)(193.0703, 268.89)(194.065, 64.25)	72895.11	15214.45	70143.3	15955.22	87038.29	19514.54	84399.23	22214.62	0.69	0.01391	0.028633	0.00	0.995562	0.998139
COMPOUND_191_0587a3_8032203	Hilic	+	191.0587	3.80				(383.1367, 179.03)(214.0496, 96.43)(209.0924, 18522.14)(210.0953, 2186.21)(211.0973, 333.72)(192.0655, 2310.36)(193.0677, 302.55)(400.143, 314.67)	65094.36	13635.97	66329.39	14153.77	77873.93	19148.12	73741.74	20469.62	0.69	0.016131	0.032698	-0.38	0.247962	0.557845
COMPOUND_191_1519a1_013443	Hilic	+	191.1519	1.01	Unknown	4	10 eV: 192.1585 (100), 174.1489 (15), 156.1373 (12)	(214.1412, 322.18)(405.279, 252.29)(192.1594, 30315.79)(193.1622, 2971.49)(194.1631, 452.83)(400.331, 201.59)	67291.72	14180.81	74487.89	19424.13	75765.57	21005.14	76602.25	20569.01	0.45	0.13381	0.208252	-0.39	0.273715	0.583098
COMPOUND_192_1519a10_810863	Rp	+	192.1519	10.81				(407.3004, 1067.14)(210.1863, 5913.94)(193.1588, 11953.92)(194.162, 2144.33)(195.1693, 226.83)	23993.24	9858.893	23738.96	8664.13	35402.15	22868	35738.66	20131.08	0.44	0.124099	0.195708	0.28	0.407648	0.693371
COMPOUND_192_1519a10_759333	Rp	+	192.1519	10.76				(407.3047, 1861.71)(210.1854, 11737.93)(193.1592, 35757.35)(194.162, 5746.61)(195.1735, 386.44)	36574.25	16858.24	40208.26	14579.61	50743.77	34115.4	49742.52	26587.81	0.35	0.235067	0.329826	-0.04	0.917987	0.962217
COMPOUND_194_0815a3_5553122	Rp	+	194.0815	3.56	Caffeine	1	10eV:195.088(100), 138.0659(32), 110.0703(2)	(217.0701, 2604.5)(218.0743, 273.64)(219.0717, 86.59)(411.1495, 256.4)(195.0882, 522888.78)(196.0911, 46580.71)(197.0931, 4064.95)(198.0947, 283.58)	1372987	1579508	1362041	1354875	4582641	2795734	2586271	2301011	1.42	7E-08	4.61E-07	-0.86	0.002269	0.067469
COMPOUND_194_094a7_493821	Rp	-	194.0940	7.49				(307.0781, 866.52)(308.0761, 88.98)(309.0792, 33.53)(193.0868, 7139.57)(194.0907, 1012.04)(195.0915, 99.99)	45943.17	6130.843	45363.8	5232.348	46986.43	6889.907	46201.72	4697.548	0.21	0.481824	0.583999	-0.10	0.774532	0.905077
COMPOUND_194_1156a1_906152	Rp	+	194.1156	1.91				(217.1051, 8450.37)(218.11, 836.96)(219.1096, 108.85)(233.0747, 369.99)(212.1495, 5498.14)(213.1532, 548.95)(214.1551, 86.24)(195.1229, 42147.06)(196.1265, 3981.09)(197.1268, 644.17)	237766.7	127507.1	220456.4	112926.7	249252.3	117495.4	220528.1	101204.2	0.21	0.489092	0.589698	-0.11	0.77048	0.902789
COMPOUND_194_1156a2_470362	Rp	+	194.1156	2.47				(217.1048, 5788.85)(218.1081, 565.25)(219.1047, 104.41)(233.0713, 409.97)(212.1495, 4658.94)(213.153, 654.79)(214.1541, 80.77)(195.123, 44458.14)(196.1264, 4118.5)(197.1279, 1035.68)	134126.8	73674.15	124895.2	62245.17	140061.4	67438.92	119981.4	57587.08	0.07	0.822888	0.874125	-0.25	0.490328	0.748114

									1st trimester				3rd trimester									
									Control		Pre-eclampsia		Control		Pre-eclampsia		Time			Interaction		
Compound	Column	Ion_mode	Mass	Retention_	Identification	ID_level	MSMS_spectra	CompositeSpectrum	Mean	SD	Mean	SD	Mean	SD	Mean	SD	Value	raw p	FDR_p	Value	raw p	FDR_p
COMPOUND_196_1218a3_4818578	Rp	+	196.1218	3.48				(197.1285, 5524.47)(198.1225, 350.72)(199.1293, 1962.19)(200.1308, 221.28)(201.1175, 59.15)	44164.96	19348.61	52275.45	27531.74	55071.92	18064.55	60161.95	32005.54	0.40	0.140349	0.216188	-0.38	0.22375	0.531523
COMPOUND_197_1785a7_8260665	Rp	+	197.1785	7.83				(417.3393, 546.16)(418.3407, 235.3)(198.1858, 10339.55)(199.1886, 1738.27)(200.1981, 291.19)	95589.66	10274.99	96440.11	16402.26	99446.14	9602.834	102138.1	15835.03	0.34	0.252206	0.348623	0.18	0.605599	0.810674
COMPOUND_198_1488a1_2070646	Hilic	+	198.1488	1.21				(199.1554, 39502.11)(200.1586, 4107.96)(201.1602, 387.32)	31817.68	29847.44	16084.15	7103.036	29744.21	9869.836	29297.49	8705.993	0.52	0.056679	0.098302	0.47	0.154094	0.466404
COMPOUND_198_1626a8_514762	Rp	+	198.1626	8.51				(221.1537, 2978.61)(222.1555, 846.98)(199.1696, 11368.71)(200.1721, 1573.4)(201.1644, 1738.21)	#DIV/0!	#DIV/0!	#DIV/0!	#DIV/0!	#DIV/0!	#DIV/0!	#DIV/0!	#DIV/0!	0.05	0.876335	0.91791	0.01	0.985161	0.992364
COMPOUND_199_0336a6_71391	Hilic	+	199.0336	6.71				(200.0408, 6186.79)(201.0435, 474.91)(202.0457, 70.02)	42924.42	8576.53	47888.11	9994.617	47243.43	9501.217	51126.58	9924.724	0.42	0.136389	0.21168	-0.10	0.755242	0.895058
COMPOUND_201_1114a3_757579	Hilic	+	201.1114	3.76				(224.0965, 1043.36)(202.119, 10009.02)(203.1216, 1118.41)(204.1227, 601.22)	9797.667	2663.113	29712.5	32786.42	54921.75	28397.35	60411.42	32895.89	1.19	1.99E-11	4.53E-10	0.22	0.193823	0.505089
COMPOUND_201_1738a1_2995349	Hilic	+	201.1738	1.30				(219.2116, 91.82)(202.1806, 270025.62)(203.1838, 33622.65)(204.1862, 3332.73)(205.1872, 248.96)	133086.8	66366.87	151740	64650.66	383847.5	301078.8	370124.2	431863.4	1.22	2.47E-06	1.12E-05	-0.29	0.295865	0.606268
COMPOUND_202_1325a4_476957	Hilic	+	202.1325	4.48	Unknown	4	20 eV: 100.0754 (100), 86.0966 (15)	(225.1246, 1079.81)(226.1256, 188.12)(203.1395, 24550.99)(204.1418, 2799.69)(205.1418, 404.01)	35662.23	9317.126	35808.58	8855.047	61502.57	12276.24	75492.86	23734.45	1.17	5.08E-08	3.49E-07	0.46	0.041687	0.265944
COMPOUND_202_1366a7_922454	Rp	+	202.1366	7.92				(203.1441, 9232.91)(204.1466, 1400.58)(205.1501, 254.86)	23281.29	11055.98	23427	8172.656	25462	3270.533	25215.67	10743.85	-1.05	6.56E-06	2.72E-05	-0.08	0.756797	0.895589
COMPOUND_202_1431a6_3173676	Hilic	+	202.1431	6.32				(405.3021, 142.28)(203.1508, 123688.27)(204.1535, 10960.09)(205.1551, 1076.42)	169664	31966.13	172448.8	33906.5	222707	39267.03	273778.6	80293.16	0.96	7.17E-05	0.000228	0.44	0.101722	0.376156
COMPOUND_202_1431a6_3645186	Hilic	+	202.1431	6.36				(225.132, 1563.5)(226.1349, 289.57)(203.1507, 76011.93)(204.1536, 7332.54)(205.1554, 850.7)	115756.5	46942.36	115951.8	45002.59	138366.6	42205.39	167589.9	89370.95	0.68	0.014958	0.030457	0.21	0.519042	0.764125
COMPOUND_202_1724a9_02613	Rp	+	202.1724	9.03				(203.1799, 33231.36)(204.1835, 5486.97)(205.1893, 753.03)	90164.08	36817.81	98856.59	40407.67	100633.3	58787.92	111196.4	49219.72	-0.96	3.32E-05	0.000117	-0.31	0.231616	0.537875
COMPOUND_203_0352a3_598715	Hilic	+	203.0352	3.60				(226.0243, 1501.22)(227.0281, 153.89)(228.0236, 202.36)(429.0699, 120.72)(204.0426, 5548.88)(205.0456, 593.13)(206.0415, 342.04)	38663.21	6236.437	38293.26	4906.1	39751.86	6542.313	41718.26	7494.739	0.19	0.523273	0.622181	0.30	0.388212	0.68827
COMPOUND_203_1159a2_7040265	Hilic	+	203.1159	2.70	Acetylcarnitine	2	10 eV: 204.1238 (100), 85.0284 (78), 60.0804 (15), 145.0489 (12)	(407.2396, 195.05)(408.2317, 93.84)(226.1061, 1454.5)(227.1084, 305.43)(228.1006, 188.13)(204.1229, 875433.2)(205.1266, 85177.6)(206.1286, 10555.83)	5285352	2243314	4575600	1874066	4134002	2075062	5826471	2732539	-0.56	0.059845	0.103591	1.07	0.003038	0.07865
COMPOUND_204_0611a4_753548	Hilic	+	204.0611	4.75	Unknown	4	20 eV: 74.2873 (100), 160.0403 (25)	(205.0686, 26837.9)(206.0721, 1840.48)(207.0735, 438.82)(208.0659, 27.02)	77264.53	53628.76	99596.63	90811.21	182968	115053.2	142912.8	131002.1	1.15	7.06E-05	0.000225	-0.53	0.098351	0.36878
COMPOUND_204_0794a9_358511	Rp	+	204.0794	9.36				(447.1203, 635.39)(448.1103, 186.75)(449.1173, 150.66)(431.1356, 79.88)(205.0865, 13093.85)(206.0906, 1774.75)(207.0996, 354.12)(426.171, 187.08)	40297.02	9500.348	39652.78	5304.036	39415.64	4274.172	38727.25	5517.05	0.05	0.862898	0.907212	-0.25	0.48588	0.747178
COMPOUND_204_0797a9_424371	Rp	+	204.0797	9.42				(447.1181, 742.48)(448.1178, 322.78)(449.1242, 184.3)(431.1605, 244.32)(243.0514, 173.88)(205.0865, 13947.2)(206.0897, 1481.36)(207.0956, 190.31)(426.1926, 67.17)	#DIV/0!	#DIV/0!	#DIV/0!	#DIV/0!	#DIV/0!	#DIV/0!	#DIV/0!	#DIV/0!	0.36	0.214283	0.30678	0.01	0.978182	0.987903
COMPOUND_204_1518a8_383157	Rp	+	204.1518	8.38				(205.1596, 23483.3)(206.163, 3702.5)(207.1683, 501.64)	46912.25	17271.52	45851.59	18103.43	48730.33	2837.461	39687.3	21028.62	-1.17	1.56E-07	9.47E-07	-0.13	0.591235	0.802507
COMPOUND_204_1519a9_384745	Rp	+	204.1519	9.38				(227.1442, 880.63)(228.1477, 253.91)(205.1596, 36057.78)(206.1626, 5697.08)	42955.91	20921.95	39964.87	15450.71	37790.33	5080.406	35680.4	15359.2	-1.26	3.2E-08	2.31E-07	0.02	0.937353	0.97184
COMPOUND_205_0739a4_526644	Rp	-	205.0739	4.53	Unknown	4	10 eV: 204.0667(100), 158.0613(25), 116.0507(14), 27.6129(10)	(240.045, 168.31)(204.0665, 23686.2)(205.0699, 3107.31)(206.073, 471.9)(409.1276, 159.9)	#DIV/0!	#DIV/0!	#DIV/0!	#DIV/0!	#DIV/0!	#DIV/0!	#DIV/0!	#DIV/0!	0.51	0.043958	0.078211	-0.10	0.73032	0.885165
COMPOUND_205_0744a4_5384536	Rp	+	205.0744	4.54				(449.1018, 157.49)(228.0637, 693.21)(229.0664, 194.54)(244.0312, 796.7)(206.0818, 21274.49)(207.0856, 2508.93)(208.0882, 378.94)	43577.3	11512.23	45657.35	10555.66	48284.29	12065.21	53920.74	18325.94	0.41	0.120537	0.191566	0.07	0.808674	0.918373
COMPOUND_206_1678a9_777095	Rp	+	206.1678	9.78				(207.175, 62121.49)(208.1784, 10285.92)(209.1876, 1610.09)	94147.09	34610.71	81530	32399.38	56405.75	27108.47	54871.64	29812.21	-1.41	1.27E-10	1.85E-09	0.13	0.552056	0.780002
COMPOUND_208_095a2_2298753	Hilic	+	208.0950	2.23				(231.0843, 19083.01)(232.0891, 1679.07)(233.0898, 498.95)(226.1288, 1800.64)(227.12, 123.94)(228.1228, 107.88)(209.1023, 6220.43)(210.1076, 854.73)(211.1069, 184.3)	30008.87	7136.29	33994.15	20766.82	32756.64	8040.056	30676.47	8726.052	0.36	0.240039	0.335131	-0.51	0.158662	0.468876
COMPOUND_208_1109a8_7118635	Rp	+	208.1109	8.71				(209.1179, 14987.42)(210.1239, 2244.3)(211.1157, 205.63)	22204	10001.72	20989.6	8395.582	25285.38	6222.381	20535.84	8284.538	0.40	0.133638	0.208128	-0.52	0.098113	0.36878
COMPOUND_208_1829a8_799443	Rp	+	208.1829	8.80				(226.2175, 3787.94)(227.2113, 1002.9)(209.1908, 47145.8)(210.1943, 8179.21)(211.1967, 1112.09)(212.2046, 83.61)(434.3808, 389.34)	102516.3	60169.04	81766.76	58382.41	100340.6	60739.44	103030.9	63303.71	-0.03	0.933397	0.95693	0.39	0.284285	0.593016
COMPOUND_208_1833a8_707669	Rp	+	208.1833	8.71				(226.2176, 2997.67)(227.2164, 549.22)(209.1909, 52982.06)(210.1942, 8484.25)(211.198, 1163.98)	100420.5	51290.34	80984.61	49846.15	97933.57	54208.87	100602.4	53778.55	-0.11	0.709661	0.787658	0.50	0.165005	0.47785
COMPOUND_210_0899a6_264288	Rp	+	210.0899	6.26				(233.0785, 5534.96)(234.0825, 725.78)(211.0974, 197923.23)(212.1004, 23620.89)(213.1024, 3223.7)	53450.25	141907.3	29324.17	28924.41	14806.18	4469.422	32062.66	86492.98	-0.82	0.001454	0.003676	0.19	0.517784	0.763621
COMPOUND_210_1371a4_4528456	Rp	+	210.1371	4.45				(211.1445, 6251.51)(212.1471, 918.79)(213.1489, 136.44)	22776.46	8749.653	25236.05	9816.744	27104.93	7192.029	26497.25	9480.413	0.53	0.029351	0.05536	-0.37	0.185635	0.499463
COMPOUND_210_1378a4_631759	Rp	+	210.1378	4.63				(233.1258, 323.56)(211.1447, 19244.79)(212.1474, 2658.44)(213.1478, 264.21)	19130.41	7434.319	20913.76	10089.57	33932.86	10144.24	34045.75	18212.9	1.25	6.96E-08	4.59E-07	-0.34	0.14739	0.456493
COMPOUND_210_1628a10_757662	Rp	+	210.1628	10.76				(228.1961, 691234.5)(229.1997, 91315.52)(230.2026, 8996.67)(231.2057, 795.98)(211.1697, 13483.61)(212.1722, 2036.12)(213.1651, 149.82)(438.3615, 1414.61)	#DIV/0!	#DIV/0!	#DIV/0!	#DIV/0!	#DIV/0!	#DIV/0!	#DIV/0!	#DIV/0!	0.43	0.148742	0.226482	0.00	0.989031	0.99433
COMPOUND_210_1634a10_823721	Rp	+	210.1634	10.82				(228.1965, 34502.48)(229.1993, 4859.17)(230.2015, 678.57)(231.2061, 53.69)(211.1697, 16842.99)(212.1725, 2773.57)	558828	320898.2	616496.6	301125.8	911815.9	743482.4	958270.2	598787.1	0.61	0.034532	0.063534	0.07	0.8446	0.93246
COMPOUND_211_9926a4_6164865	Hilic	+	211.9926	4.62				(425.0033, 589.5)(425.9954, 63.55)(234.9817, 1192.8)(235.981, 102.92)(212.9997, 8758.35)(214.0017, 586.84)(215.0006, 106.37)	31978.68	5096.043	35463.57	5384.612	38835.93	4683.826	40725.12	5175.268	1.11	1.75E-05	6.62E-05	-0.30	0.29141	0.601884
COMPOUND_213_0834a6_825285	Rp	+	213.0834	6.83				(236.0724, 9728.57)(237.077, 1563.31)(238.0708, 558.09)(449.1617, 299.79)(450.1556, 99.95)(451.1612, 126.0)(252.0467, 1094.06)(253.05, 196.62)(254.0429, 137.97)(231.117, 13415.17)(232.1201, 1815.67)(233.1157, 740.68)(214.0905, 131749.89)(215.0935, 15556.05)(216.088, 6466.89)(217.0885, 132.95)	2942396	248993.7	2791447	272032.4	2837409	380809.9	2908859	322321.4	-0.34	0.247443	0.342707	0.92	0.009991	0.153413
COMPOUND_214_0297a5_508314	Rp	-	214.0297	5.51				(213.0225, 5038.46)(214.0258, 533.61)(215.0225, 321.66)(216.0265, 22.7)	197243.7	241174.3	225083.9	402624.6	127142.6	107312.5	92865.52	94557.09	-0.08	0.762655	0.828509	-0.47	0.15886	0.468876
COMPOUND_214_0298a5_316028	Rp	-	214.0298	5.32				(213.0226, 12748.66)(214.0257, 1913.2)(215.0204, 902.33)(216.0214, 46.68)(259.0274, 950.89)	41493.11	42070.9	35313.86	29930.31	70983.54	56766.53	37459.08	25500.37	1.01	0.000216	0.000637	-0.55	0.075457	0.329961
COMPOUND_216_1003a4_53358	Rp	+	216.1003	4.53				(239.0857, 1003.14)(240.0864, 148.75)(234.1342, 4040.43)(235.1379, 352.61)(236.1334, 420.21)(217.1073, 7682.23)(218.1119, 982.71)(219.113, 167.58)	31748.45	11036.75	26609.49	11292.65	30014.57	9994.498	32134.47	10333.27	-0.13	0.649163	0.737859	0.72	0.039217	0.261543
COMPOUND_216_1222a5_5747	Hilic	+	216.1222	5.57				(239.1059, 611.42)(217.1302, 13485.79)(218.1329, 1607.34)(219.1398, 258.14)	13782.08	4578.161	13959.15	3984.626	15561.86	3740.4	16798.84	6691.569	0.32	0.203527	0.294704	0.12	0.67593	0.851339
COMPOUND_217_1319a1_9099891	Hilic	+	217.1319	1.91	Propionylcarnitine	2	20 eV: 85.0282 (100), 218.1386 (5)	(240.1204, 445.44)(241.1221, 115.7)(218.1389, 162789.23)(219.1422, 17372.75)(220.1438, 2667.97)	471165.4	146504.8	462786.9	147122.7	348666.2	69888.89	410359.2	156989.1	-0.94	0.000704	0.001896	0.54	0.086217	0.348584
COMPOUND_218_0197a5_1376905	Hilic	+	218.0197	5.14				(219.0268, 21622.8)(220.0305, 1547.2)(221.0262, 1846.4)(222.0293, 101.87)	300561.5	25985.96	300541	52494.01	315524.8	86096.5	326846	48893.47	0.17	0.559284	0.656827	0.55	0.11828	0.408539
COMPOUND_218_0801a4_6811094	Rp	+	218.0801	4.68				(241.069, 17881.5)(242.0723, 1731.95)(243.0726, 328.65)(459.1426, 216.47)(257.0425, 1839.87)(258.0443, 216.07)(259.0301, 315.67)(219.0863, 1957.3)(220.0905, 179.12)(221.0929, 2230.02)	45764.89	19395.75	43257.74	14107.82	39544.36	10637.4	47028.89	20293.63	-0.21	0.487622	0.588806	0.36	0.321259	0.628561
COMPOUND_218_1267a5_7108383	Hilic	+	218.1267	5.71				(241.1163, 1359.55)(242.1176, 312.36)(219.1343, 31927.72)(220.1374, 3118.68)(221.1385, 657.99)(454.2804, 379.17)	14883.71	8019.96	16165.45	7316.78	21219.57	21548.88	14370.16	14278.33	-0.58	0.034157	0.062999	-0.18	0.56939	0.788601
COMPOUND_218_1313a7_728156	Rp	+	218.1313	7.73				(219.1387, 8035.45)(220.1458, 1405.32)(221.1479, 304.19)	52100	19019.1	55740.27	14927.93	45157.29	12640.54	47591.61	16726.8	-0.20	0.477413	0.580686	-0.18	0.591504	0.802507
COMPOUND_218_1674a9_106586	Rp	+	218.1674	9.11				(437.3462, 1138.41)(438.3511, 560.28)(219.175, 15437.29)(220.1781, 2924.75)(221.1867, 373.08)	21346.77	6560.936	17971.28	4897.148	38801	6876.626	41275.16	8187.183	1.09	9.86E-07	4.91E-06	0.50	0.038482	0.259981
COMPOUND_219_1109a0_9964019	Hilic	+	219.1109	1.00				(242.1005, 5639.11)(243.105, 791.79)(244.1043, 113.3)(220.1181, 22917.11)(221.1221, 2313.88)(222.1173, 655.1)	20775.94	13175.33	18664.8	5578.769	36064.93	17610.88	35293.89	9070.087	1.13	1.31E-08	1.05E-07	0.21	0.288684	0.597533
COMPOUND_220_1104a8_320629	Rp	+	220.1104	8.32				(441.2319, 571.59)(442.2386, 256.62)(443.2186, 41.64)(243.0995, 3979.95)(244.1076, 470.32)(245.1104, 129.65)(221.118, 36184.02)(222.1211, 5958.21)(223.1244, 725.03)(458.2541, 402.35)(459.2574, 243.83)	76127.1	31343.45	63919.27	32466.56	82416.86	38495.48	76019.04	32018.66	0.22	0.464523	0.569464	0.18	0.616816	0.816971
COMPOUND_220_1107a8_403094	Rp	+	220.1107	8.40				(441.228, 763.52)(442.2311, 169.52)(443.2343, 96.05)(243.0999, 8418.75)(244.1037, 1162.63)(245.106, 275.28)(221.1181, 77108.98)(222.1213, 12165.01)(223.1247, 1355.37)(458.2596, 526.81)(459.2603, 277.52)	163556.6	69571.34	131987.7	78475.12	187980.6	80800.8	163620	70129.91	0.28	0.342622	0.443695	0.15	0.664294	0.844813
COMPOUND_222_0896a7_6437235	Rp	+	222.0896	7.64				(245.0706, 233.24)(246.0713, 42.99)(223.0975, 172507.17)(224.1008, 22828.02)(225.1032, 3022.51)(226.1071, 252.58)	#DIV/0!	#DIV/0!	#DIV/0!	#DIV/0!	#DIV/0!	#DIV/0!	#DIV/0!	#DIV/0!	-0.07	0.801677	0.857257	-0.01	0.972469	0.984712

Compound	Column	Ion_mode	Mass	Retention_	Identification	ID_level	MSMS_spectra	CompositeSpectrum	1st trimester				3rd trimester				Time			Interaction		
									Control		Pre-eclampsia		Control		Pre-eclampsia							
									Mean	SD	Mean	SD	Mean	SD	Mean	SD	Value	raw p	FDR_p	Value	raw p	FDR_p
COMPOUND_222_0899a7_359233	Rp	+	222.0899	7.36				(483.1288, 265.78)(245.0794, 6331.47)(246.0827, 1159.75)(247.0879, 267.05)(467.1531, 388.6)(468.1493, 61.28)(261.0534, 425.05)(223.0972, 34325.86)(224.1006, 5241.66)(225.1031, 786.73)(226.1017, 65.99)	171402.7	46982.07	161423.4	42163.67	166109.8	44359.36	175510.5	52546.31	-0.05	0.864789	0.908351	0.37	0.308275	0.614305
COMPOUND_222_1255a7_407982	Rp	-	222.1255	7.41	no msms			(479.2274, 3004.66)(480.2313, 786.62)(257.092, 100.54)(335.1001, 330.41)(221.1179, 8826.59)(222.1211, 1274.56)(223.1307, 260.53)(267.123, 211.44)(268.1198, 60.67)	44747.7	5763.16	47022.74	5109.124	55508.07	8886.364	49382.35	6447.042	1.44	1.42E-06	6.9E-06	-1.11	0.000863	0.046284
COMPOUND_222_1618a9_278769	Rp	-	222.1618	9.28				(257.1305, 563.07)(258.1318, 33.61)(259.1245, 58.28)(221.1545, 4316.78)(222.1584, 931.3)(223.1643, 94.44)	14841.21	2007.405	16279.54	2462.534	14128.5	1844.519	14954.65	1888.398	-0.33	0.256184	0.352716	-0.24	0.485576	0.747178
COMPOUND_223_1215a5_385399	Hilic	+	223.1215	5.39				(246.1109, 545.71)(247.1141, 102.24)(248.1086, 98.45)(224.1282, 25508.55)(225.1313, 3910.16)(226.1342, 260.25)	#DIV/0!	#DIV/0!	#DIV/0!	#DIV/0!	#DIV/0!	#DIV/0!	#DIV/0!	#DIV/0!	-0.27	0.323939	0.424795	-0.02	0.96165	0.981922
COMPOUND_224_0274a3_056523	Hilic	+	224.0274	3.06				(242.0513, 120.02)(225.0345, 3888.39)(226.0367, 622.47)(227.0315, 1657.29)(228.0345, 242.78)(229.0284, 61.65)	63163.66	14362.03	64172.98	10595.91	60981.07	14960.23	61865.07	11970.63	-0.19	0.526947	0.625888	-0.02	0.947411	0.976462
COMPOUND_224_0278a3_0012653	Hilic	+	224.0278	3.00				(225.0352, 4365.48)(226.0377, 670.5)(227.0322, 1479.25)(228.0343, 162.89)(229.0334, 112.77)	41238.19	28840.59	35293.93	26610.72	31557.14	25184.92	40000.91	26685.17	-0.69	0.024811	0.047634	0.65	0.073642	0.326462
COMPOUND_224_1408a7_7266345	Rp	-	224.1408	7.73				(483.2587, 658.53)(484.2601, 249.65)(259.1092, 335.76)(260.1109, 22.9)(261.1065, 219.98)(223.1337, 3376.55)(224.1367, 513.89)(225.1444, 111.55)	16742.49	2951.656	16433.02	2633.073	18174.93	3932.025	17063.6	2744.504	0.48	0.113146	0.181482	-0.29	0.42549	0.705315
COMPOUND_224_1416a7_737181	Rp	+	224.1416	7.74				(247.1284, 715.1)(248.1295, 49.63)(249.1132, 524.11)(225.1493, 12541.87)(226.1531, 1822.57)(227.1594, 559.82)(466.3167, 5600.15)(467.3214, 1773.92)(468.3213, 378.67)	64269.09	6806.237	63514.52	6561.001	66566.71	6218.738	63473.33	7454.876	0.30	0.315065	0.417123	-0.34	0.338127	0.642187
COMPOUND_225_0755a1_6014915	Hilic	+	225.0755	1.60				(451.1576, 849.23)(452.1607, 198.86)(248.0646, 4156.81)(249.0688, 478.5)(250.0714, 54.1)(473.1433, 331.92)(243.1123, 105.04)(226.0829, 7290.93)(227.0868, 929.84)(228.1002, 119.09)	15988.37	5891.935	17521.51	6517.536	22702.5	6889.267	27230.49	9170.856	0.86	0.000512	0.001422	0.23	0.410974	0.694731
COMPOUND_225_1735a10_758327	Rp	+	225.1735	10.76				(248.1632, 1114.71)(226.1811, 123859.27)(227.1841, 19428.05)	74394.1	43147.25	82613	39429.19	94825.23	83920.07	90098.29	67451.82	0.06	0.839285	0.887774	-0.07	0.843605	0.93246
COMPOUND_225_9443a5_387598	Hilic	+	225.9443	5.39				(226.9517, 6660.35)(227.9555, 358.97)(228.956, 106.4)(469.9257, 811.68)(470.9291, 125.22)	30162.45	6586.453	32041.85	4593.612	29360.86	4514.274	30643.75	5274.754	-0.04	0.882494	0.922216	-0.18	0.54664	0.77815
COMPOUND_225_9447a4_214626	Hilic	+	225.9447	4.21				(226.9519, 145803.81)(227.9551, 5159.22)(228.956, 1990.64)(229.9587, 73.14)	1388692	89471.64	1348519	96128.94	1371412	112780.3	1400539	111171.8	-0.16	0.595204	0.690571	0.77	0.030186	0.234557
COMPOUND_226_1069a6_7047267	Hilic	+	226.1069	6.70				(249.0966, 3139.18)(250.0986, 432.4)(227.1142, 71632.97)(228.1172, 8533.14)(229.1195, 917.38)	79950.34	52233.45	95496.11	67407.35	41059.82	47373.27	30638.16	43494.76	-1.02	3.96E-05	0.000135	-0.26	0.344143	0.650723
COMPOUND_227_1892a7_6200376	Rp	+	227.1892	7.62				(250.1787, 7834.54)(251.1819, 1271.48)(252.1825, 267.93)(266.1508, 662.81)(267.1565, 218.87)(228.1969, 122100.52)(229.2, 16783.48)(230.2039, 1837.83)(231.197, 146.05)	356780.1	61471.7	392346.3	81801.15	383115.4	119746.5	386315	110476	0.17	0.568433	0.665338	-0.31	0.389299	0.689231
COMPOUND_227_1896a8_620347	Rp	+	227.1896	8.62				(493.3424, 1255.72)(494.3465, 792.03)(228.1966, 9905.08)(229.1979, 1543.51)(230.2047, 179.48)	#DIV/0!	#DIV/0!	#DIV/0!	#DIV/0!	#DIV/0!	#DIV/0!	#DIV/0!	#DIV/0!	0.22	0.434067	0.539172	0.01	0.987781	0.994046
COMPOUND_227_1899a10_823332	Rp	+	227.1899	10.82	Myristic acid	1	10eV: 175.1501(100), 95.085(80), 228.1965(40), 151.469(25)	(250.1786, 61277.63)(251.1821, 8946.83)(252.1751, 645.06)(228.1966, 322520.44)(229.1998, 46013.67)(230.2026, 4519.31)(231.2027, 390.74)(472.4291, 809.22)	570268	287944	624098.5	302245.3	960393.6	780495.1	1011783	616985.6	0.66	0.021347	0.041766	0.10	0.771624	0.903639
COMPOUND_227_1904a10_4461565	Rp	+	227.1904	10.45				(245.2274, 22876.42)(228.1966, 11734.01)(229.1997, 1560.72)(230.2055, 315.92)	33678.04	14908.51	33999.57	19908.98	39968.31	12879.19	50570.15	26367.26	0.29	0.292926	0.392194	0.33	0.29801	0.60748
COMPOUND_227_2255a6_6411934	Rp	+	227.2255	6.64				(228.233, 27619.0)(229.2361, 4260.13)(230.24, 324.58)	75925	65304.77	52430.08	58068.72	64390.43	42181.94	77979.93	64244.22	-0.02	0.939737	0.960639	0.81	0.015808	0.188356
COMPOUND_228_1477a4_747693	Hilic	+	228.1477	4.75				(229.155, 56956.08)(230.1582, 7218.09)(231.1604, 886.59)	116595.4	67984.89	110218.3	70894.66	95927.43	64885.25	202111.7	267520.6	-0.35	0.219751	0.31355	1.06	0.002155	0.067469
COMPOUND_228_1478a3_9028063	Hilic	+	228.1478	3.90				(246.1732, 103.69)(229.155, 82398.07)(230.1581, 10938.51)(231.1608, 1199.66)	173543.8	57433.42	180197.4	47317.02	141666.9	48038.54	284149.3	290264.2	-0.57	0.034999	0.064288	1.06	0.001131	0.055356
COMPOUND_228_2086a10_465194	Rp	-	228.2086	10.47	Myristic acid	1	10 eV: 227.2014(100), 96.4889(4), 33.9118(4)	(263.1721, 149.02)(227.2014, 75895.78)(228.2048, 12894.7)(229.2077, 1206.46)(230.2081, 104.05)(455.4067, 307.09)(273.2065, 3535.68)(274.2082, 661.38)(275.2028, 157.07)	133361.8	43393.27	158022.9	50240.61	169039.1	47911.96	184268.1	49940.91	0.74	0.009882	0.020995	-0.25	0.458371	0.727963
COMPOUND_229_1678a0_78063387	Hilic	+	229.1678	0.78				(459.3477, 469.85)(460.3518, 133.49)(252.1588, 694.82)(230.1753, 6655.6)(231.1779, 1049.15)(232.1721, 351.47)	16458.9	4723.804	15889.21	4328.206	9633.667	3588.65	10182.86	6410.829	-1.31	5E-09	4.38E-08	0.21	0.354928	0.664974
COMPOUND_230_1641a2_3929374	Rp	+	230.1641	2.39				(231.1708, 28351.87)(232.1741, 3529.18)(233.1743, 495.72)	39662.09	35016.9	46205.29	35228.47	59384.63	44463.76	36835.69	26015.71	0.04	0.859924	0.90493	-0.59	0.027581	0.226687
COMPOUND_231_1247a5_709998	Hilic	+	231.1247	5.71				(463.2527, 375.7)(254.1119, 54514.46)(255.1151, 6524.67)(256.1173, 785.93)(485.2338, 3643.82)(486.2361, 851.37)(232.1299, 13924.15)(233.1335, 1677.26)(234.1374, 336.42)	8835.706	5493.491	9998.615	4653.913	78955.31	49379.08	67358.22	41579.93	1.46	7.19E-13	3.45E-11	-0.08	0.689659	0.856181
COMPOUND_231_1474a1_4667246	Hilic	+	231.1474	1.47				(463.2917, 636.5)(254.1375, 414.67)(232.1545, 29015.84)(233.1578, 3550.21)(234.1596, 648.67)(235.1531, 51.88)	283293.7	126583	272225.8	130276.6	266461.6	96731.53	319448.8	139063.2	-0.17	0.509196	0.609952	0.55	0.063734	0.307286
COMPOUND_231_1477a1_9933995	Rp	+	231.1477	1.99	Butylcarnitine	1	10 eV: 85.0271(100), 232.1545(55), 190.9435(15), 33.8396(1)	(232.1549, 11476.8)(233.1574, 1570.67)(234.1557, 221.37)	41597.28	19858.71	37997.17	16529.98	35354.86	13203.47	41363.16	17851.73	-0.30	0.273716	0.372453	0.60	0.0609	0.302474
COMPOUND_232_053a3_463621	Rp	+	232.0530	3.46				(233.0607, 15935.77)(234.064, 1527.49)(235.0636, 291.2)	28555.76	11479.8	26626.61	9691.315	38534.36	13234.01	53308.89	24514.45	0.71	0.003068	0.007297	0.66	0.02096	0.204049
COMPOUND_232_1825a9_655233	Rp	+	232.1825	9.66				(503.3413, 826.96)(504.3472, 375.81)(255.1787, 683.84)(256.1791, 181.62)(250.2168, 411.97)(233.191, 7604.74)(234.1941, 1273.62)	38251.35	7147.191	39500.8	6394.399	36832.07	8248.719	39349.7	8873.649	-0.09	0.755834	0.82302	0.12	0.73964	0.888787
COMPOUND_232_1836a8_81827	Rp	+	232.1836	8.82				(233.191, 23468.72)(234.1942, 4209.95)(235.1963, 488.55)	67783.36	8995.027	69881.7	7555.289	67320.29	9551.525	67785.26	9739.637	0.03	0.906763	0.938328	-0.19	0.594167	0.804125
COMPOUND_234_1136a1_7280852	Hilic	+	234.1136	1.73				(257.1039, 1337.05)(258.1062, 414.47)(235.1197, 17727.24)(236.1229, 2568.19)(237.125, 280.05)	28974.6	11796.77	32656.96	17894.02	22421.54	19742.79	15591.78	7777.838	-0.64	0.005479	0.01229	-0.79	0.004654	0.105867
COMPOUND_234_1616a9_010874	Rp	-	234.1616	9.01				(233.1543, 18522.82)(234.1578, 3197.47)(235.1612, 452.36)	111617.7	10018.94	116976.1	10414.55	99213.93	16874.01	104887.7	10233.3	-0.90	0.001068	0.002768	-0.12	0.703108	0.86442
COMPOUND_234_1633a9_016191	Rp	+	234.1633	9.02				(257.154, 941.24)(258.1577, 312.5)(259.1488, 2236.97)(235.1703, 99879.56)(236.1735, 16473.6)(237.1802, 2775.31)(238.1872, 233.49)	750743.9	35994.43	740895.4	42311.05	768819.6	50156.12	782928.2	41059.28	0.44	0.117728	0.187521	0.49	0.149506	0.458703
COMPOUND_235_1064a4_680067	Rp	+	235.1064	4.68				(236.1137, 68388.15)(237.1169, 6933.62)(238.1189, 1441.67)(239.1205, 125.8)	441492.4	166401.6	389442.7	118674.8	378917.4	102897.4	447350	172549.5	-0.28	0.358843	0.461252	0.66	0.071672	0.325554
COMPOUND_236_1048a7_6399374	Rp	-	236.1048	7.64				(349.0893, 143.39)(235.0973, 3751.64)(236.1007, 581.42)(237.108, 144.45)	16699.32	1511.494	17279.61	1707.026	17278.64	1227.966	16630.32	1608.843	0.36	0.229012	0.324051	-0.78	0.032634	0.244873
COMPOUND_236_1413a8_4762125	Rp	-	236.1413	8.48				(271.1118, 349.58)(272.1139, 79.22)(273.1043, 182.28)(235.1339, 2915.71)(236.1371, 442.74)(237.138, 79.96)	16137.98	1641.301	16260.8	1778.533	16414	1540.539	16144	1140.245	0.09	0.770635	0.833358	-0.14	0.693459	0.85806
COMPOUND_236_215a10_315211	Rp	+	236.2150	10.32				(259.207, 5427.4)(260.2107, 1138.89)(261.2099, 131.19)(237.2219, 47272.77)(238.225, 8694.57)(239.232, 1239.26)	48044.26	13947.82	45108.48	11145.01	32432.92	14861.39	52065.26	34735.11	-1.04	0.000663	0.001803	1.15	0.001567	0.06002
COMPOUND_238_0841a5_3442845	Rp	+	238.0841	5.34				(239.0919, 13874.54)(240.0954, 1745.54)(241.0969, 461.98)(242.1059, 38.01)	112707	104492.5	75978.46	49176.71	58862.86	44678.67	45760.2	25498.54	-0.76	5.7E-07	3.02E-06	0.07	0.65767	0.842058
COMPOUND_238_1209a7_1961994	Rp	+	238.1209	7.20				(261.1107, 8044.04)(262.1136, 1164.99)(263.1153, 94.87)(277.0839, 499.45)(239.1287, 39316.63)(240.1322, 5600.42)(241.1381, 872.92)	106378.3	51806.51	95078.91	50940.75	103596.9	46526.41	105841.5	53117.74	0.00	0.999713	0.999965	0.24	0.502047	0.75374
COMPOUND_238_1213a7_156719	Rp	+	238.1213	7.16				(261.1106, 5288.05)(262.1159, 784.06)(263.1205, 183.6)(277.0835, 251.56)(278.0843, 50.67)(239.1286, 17091.99)(240.1318, 2766.61)(241.1391, 643.56)	69244.75	26783.71	73138.91	34644	61588.71	20637.05	70948.26	31931.15	-0.23	0.44578	0.551591	0.25	0.489564	0.748114
COMPOUND_238_1575a9_549709	Rp	+	238.1575	9.55				(261.1467, 1417.3)(262.1502, 392.12)(263.144, 35.93)(277.1079, 366.67)(239.1651, 8015.87)(240.1684, 1471.9)(241.1699, 541.66)(242.1673, 39.53)	65584.92	32609.55	64939.07	26250.23	52826.57	11993.48	49805.28	24099.88	-0.32	0.228051	0.323	-0.37	0.223204	0.531262
COMPOUND_238_2312a10_599689	Rp	+	238.2312	10.60				(256.2642, 75783.02)(257.2668, 13428.76)(258.2697, 1906.41)(239.2376, 47352.92)(240.2412, 8943.25)(241.2413, 1194.47)(494.5107, 217.46)	106910.3	62708.84	105487.5	60175.73	87560.23	43212.05	104182.6	92973.01	-0.27	0.349959	0.452156	0.35	0.30232	0.610641
COMPOUND_240_135a8_091674	Rp	-	240.1350	8.09	CMPF	1	10 eV: 195.1018(100), 239.0909(40), 151.1115(18), 109.9354(3)	(525.268, 1628.35)(526.2708, 601.3)(527.2651, 281.02)(239.1274, 6224.4)(240.132, 684.62)(241.1462, 62.6)	25037.53	3454.357	25315.63	3262.732	26661.21	5361.465	25675.14	3943.076	0.59	0.053147	0.092494	-0.50	0.165477	0.47785
COMPOUND_241_2046a10_758661	Rp	+	241.2046	10.76				(483.4049, 422.88)(484.3993, 241.8)(485.3984, 81.88)(521.3752, 1780.87)(505.378, 961.66)(242.212, 57815.29)(243.2155, 8564.65)(244.2259, 716.9)	213678.7	102949.5	234546.2	94092.6	294750.7	220304.8	302269.7	177516.9	0.45	0.131362	0.205578	-0.05	0.884919	0.952599
COMPOUND_241_2048a10_826314	Rp	+	241.2048	10.83				(483.4065, 386.29)(521.3646, 1673.37)(242.2125, 45202.02)(243.2159, 7182.39)(244.2215, 844.04)(500.4279, 288.05)	125088.4	57432.42	135507.2	59109.71	227888.8	161687.4	220728.5	133163.2	0.76	0.00692	0.015218	0.03	0.925763	0.966461
COMPOUND_241_2781a7_499523	Rp	+	241.2781	7.50				(242.2851, 35938.54)(243.2886, 6022.31)(244.2919, 450.87)	277469.8	29900.68	261941.6	29005.54	273159.4	24694.65	282499.8	29718.31	-0.14	0.630019	0.720571	0.85	0.012981	0.173893
COMPOUND_242_0461a3_7858174	Hilic	+	242.0461	3.79				(485.0884, 368.96)(486.0905, 113.45)(487.0882, 358.27)(265.0356, 2306.21)(266.0403, 335.44)(267.0281, 75.71)(507.08, 152.2)(243.0535, 22398.21)(244.0572, 3239.35)(245.0531, 1912.19)(246.0538, 182.36)	145165.2	17191.97	147646.4	22722.26	132327.5	26554.74	135073.8	33574.95	-0.54	0.063983	0.110161	0.04	0.907219	0.958319

									1st trimester				3rd trimester									
									Control		Pre-eclampsia		Control		Pre-eclampsia		Time			Interaction		
Compound	Column	Ion_mode	Mass	Retention_	Identification	ID_level	MSMS_spectra	CompositeSpectrum	Mean	SD	Mean	SD	Mean	SD	Mean	SD	Value	raw p	FDR_p	Value	raw p	FDR_p
COMPOUND_242_177a1_4351301	Hilic	+	242.1770	1.44				(485.3592, 6696.9)(486.3615, 2377.64)(487.366, 602.24)(243.184, 9873.84)(244.1877, 1242.55)(245.1816, 1288.6)(246.1846, 117.6)(247.1741, 105.19)	62230.72	20823.96	64129.8	24688.84	55997.14	37794.89	51955.89	27372.35	-0.51	0.085593	0.141726	-0.08	0.825709	0.923744
COMPOUND_242_2238a10_637414	Rp	-	242.2238	10.64				(519.4035, 1180.89)(241.217, 10650.08)(242.2198, 1700.01)(243.2234, 193.15)(287.2214, 438.86)(288.2158, 53.21)	16368.69	4650.828	17925.86	4920.446	21119.71	8688.515	18290.82	6179.047	0.81	0.005723	0.0128	-0.73	0.033931	0.248347
COMPOUND_242_2244a10_659308	Rp	-	242.2244	10.66				(519.408, 263.11)(241.2171, 8995.49)(242.2204, 1509.47)(243.2222, 130.85)(287.2223, 377.79)(288.2296, 73.66)	16740.88	4877.989	19970.64	7336.978	22466.5	8386.649	21151.85	8472.495	0.88	0.003116	0.007394	-0.62	0.07284	0.326093
COMPOUND_243_1491a1_3397642	Hilic	+	243.1491	1.34				(266.1394, 33308.27)(509.284, 441.47)(244.1546, 4608.02)(245.1581, 787.69)(246.1536, 94.93)	33639.02	11584.61	34800.25	10260.69	29085.5	11541.89	41957.63	32961.91	-0.62	0.023572	0.045487	0.54	0.091532	0.359093
COMPOUND_243_1826a10_816747	Rp	-	243.1826	10.82				(521.3172, 305.79)(278.1524, 2669.09)(279.1574, 486.25)(280.1501, 963.75)(281.1411, 127.68)(531.3635, 334.43)(242.176, 16197.13)(243.1795, 2595.44)(244.1812, 239.45)(485.3457, 473.24)(486.3545, 166.15)	19569.71	8143.41	21713.09	9393.096	23525.36	12071.95	25841.8	12784.98	0.37	0.21352	0.306078	-0.09	0.802917	0.916921
COMPOUND_243_1833a7_493578	Rp	-	243.1833	7.49				(356.1556, 138.93)(278.1525, 373.78)(279.1567, 99.66)(280.148, 173.38)(242.176, 18290.99)(243.1797, 2605.52)(244.18, 297.58)(599.3318, 167.22)	26533.57	7657.591	28821.87	9401.18	28545.14	11275.37	28158.75	11803.91	0.13	0.657101	0.745111	-0.27	0.456918	0.727709
COMPOUND_243_184a10_81224	Rp	+	243.1840	10.81				(487.373, 2189.69)(488.3759, 827.46)(489.3674, 936.32)(490.3673, 311.33)(525.315, 295.64)(266.173, 26197.24)(267.1768, 4069.96)(268.1775, 411.72)(282.1393, 1100.4)(244.1916, 268717.47)(245.1947, 36081.71)(246.1967, 4622.3)(504.3784, 719.11)(505.3679, 1130.86)	148776.5	86794.64	153981.1	100993.1	273891.5	248302.7	266464.9	141941.4	0.72	0.01068	0.022457	0.10	0.749224	0.891783
COMPOUND_243_1841a7_5022793	Rp	+	243.1841	7.50				(266.1736, 1968.93)(267.1762, 356.4)(282.1397, 672.96)(244.1916, 12350.64)(245.1946, 1943.43)(246.1959, 313.58)(247.1888, 29.12)	44051.7	13583.2	46111.91	15930.63	47457.43	20356.88	46669.65	20073.93	0.14	0.638801	0.728395	-0.19	0.611766	0.814985
COMPOUND_243_1843a10_825046	Rp	+	243.1843	10.83				(487.3751, 2729.14)(488.3835, 1113.25)(489.3872, 939.29)(525.3134, 164.43)(266.1735, 32028.64)(267.1771, 5505.4)(268.1776, 520.1)(282.1373, 1400.01)(244.192, 326917.2)(245.1952, 45091.02)(246.1977, 5778.93)(504.3965, 450.56)(505.4133, 261.29)	165023.3	84958.01	185720.1	84705.65	309346.5	241401.8	317046.8	208582.2	0.83	0.003403	0.007991	-0.06	0.851615	0.936901
COMPOUND_244_0889a0_90353024	Hilic	+	244.0889	0.90				(489.1976, 155.02)(267.0795, 435.89)(511.1814, 160.77)(245.096, 8626.18)(246.0981, 870.23)(247.094, 334.23)(506.2238, 444.11)	13939.04	3106.176	13051.91	2911.712	12119.36	2868.04	12617.65	3801.831	-0.55	0.068207	0.116454	0.36	0.310295	0.616211
COMPOUND_244_1789a1_7912574	Hilic	+	244.1789	1.79	Leucine-Leucine	2	10 eV: 86.0976 (100), 245.1856 (25), 132.1023 (20)	(489.3649, 324.21)(490.3677, 90.97)(267.1685, 7228.13)(268.1717, 1035.25)(269.1777, 147.8)(511.3446, 592.47)(245.1865, 44845.3)(246.19, 6275.68)(247.192, 574.9)(506.4027, 95.67)	30088.48	45157.59	36054.28	34802.36	70143	78696.13	38660.92	62747.22	0.38	0.180836	0.2681	-1.08	0.001858	0.061536
COMPOUND_244_1789a3_7724419	Rp	+	244.1789	3.77				(489.3658, 55.51)(245.1869, 34080.21)(246.1904, 4928.89)(247.1921, 695.66)(248.1853, 63.93)	145576.1	225175.5	174184.7	188830.9	351590.8	410817.2	156263.3	254395.4	0.36	0.193183	0.282004	-1.08	0.001717	0.06041
COMPOUND_244_2201a10_460588	Rp	+	244.2201	10.46				(511.42, 504.43)(245.2268, 5997.58)(246.2317, 1075.22)(247.2267, 88.46)	22704.15	11388.55	25140.63	18762.9	27893.9	7745.131	42587.54	22538.24	0.02	0.934308	0.95693	0.95	0.002348	0.067469
COMPOUND_245_1632a2_8370006	Rp	+	245.1632	2.84	Isovalerylcarnitine	1	10 eV: 246.1697(100), 85.0267(90), 179.9112(15), 60.0795(10)	(268.1553, 52329.32)(246.1709, 261319.62)(247.1742, 33896.7)(248.176, 4330.24)	67315.36	25639.23	140665.6	484357.6	55352.14	20128.7	71293.7	88994.74	-0.43	0.144209	0.221225	0.05	0.881806	0.950612
COMPOUND_246_1333a5_5420938	Hilic	+	246.1333	5.54				(493.2812, 208.49)(269.1233, 597.01)(270.1292, 135.03)(247.1407, 52542.5)(248.1439, 5190.8)(249.1464, 784.95)	38741.92	15012.74	46761.7	18291.17	19555.18	23832.47	18473.54	26963.03	-0.98	0.000116	0.000355	-0.37	0.201102	0.513471
COMPOUND_246_1372a2_7683043	Rp	+	246.1372	2.77				(493.2878, 59.19)(269.1292, 179.66)(247.1451, 120905.54)(248.148, 18546.61)(249.1505, 1924.61)	137921.9	246547.7	95156.17	85476.75	157320.4	232764.9	95340.36	94455.65	-0.27	0.18999	0.279097	0.23	0.331907	0.636996
COMPOUND_246_1375a1_3290383	Hilic	+	246.1375	1.33	Tryptophan betaine	1	20 eV: 146.061 (100), 188.072 (54), 60.082 (48)	(493.2818, 4203.65)(494.2843, 1235.48)(495.282, 196.74)(269.1264, 13027.6)(270.1292, 2130.36)(271.1314, 250.29)(515.264, 577.41)(516.2686, 138.2)(247.1442, 266633.2)(248.1476, 41785.43)(249.1506, 4658.8)(250.1483, 421.84)(510.297, 1775.38)	434327.2	846558.7	307627.8	295149.6	471698.4	827318.2	284060	334351.1	-0.31	0.12447	0.196155	0.18	0.433947	0.712127
COMPOUND_246_2348a10_693114	Rp	+	246.2348	10.69	no msms			(247.2426, 16749.08)(248.2463, 3264.52)(249.25, 308.2)	18251.9	7366.659	16849.1	8589.885	16448	3937.029	29617.36	13166.24	0.04	0.863638	0.907566	1.14	6.28E-05	0.010097
COMPOUND_247_142a3_9591537	Hilic	+	247.1420	3.96				(248.1495, 21818.15)(249.1532, 2821.55)(250.1532, 559.79)	26616.48	15860.18	27099.66	11164.12	26789.5	15261.06	41813.77	29833.32	0.00	0.998069	0.999401	0.69	0.017798	0.195199
COMPOUND_248_1267a4_403865	Rp	+	248.1267	4.40				(271.116, 1409.77)(272.1178, 168.81)(273.1057, 89.28)(266.1606, 6450.47)(267.1731, 1156.19)(268.1673, 343.85)(287.0944, 152.06)(249.1342, 7763.65)(250.1391, 1371.07)(251.1401, 221.63)	53461.3	10795.5	50974.98	9437.95	51859.38	8166.845	53876.65	9728.551	-0.12	0.691203	0.774808	0.40	0.263417	0.574821
COMPOUND_248_1782a9_118431	Rp	+	248.1782	9.12				(497.3697, 3031.8)(498.3583, 1133.68)(499.3584, 547.36)(271.17, 4519.69)(272.1742, 1096.24)(273.1708, 486.1)(519.3432, 507.24)(520.3498, 888.37)(249.1857, 48631.65)(250.1892, 8771.26)(251.1965, 1912.65)(252.2006, 123.04)	242039.9	27546.13	251239.5	28649.25	269103.4	47518.92	247797.3	29180.71	0.75	0.011555	0.02405	-0.89	0.012435	0.169858
COMPOUND_248_1989a8_150036	Rp	+	248.1989	8.15				(271.1915, 3786.38)(272.1885, 541.32)(273.1863, 576.24)(519.3833, 2724.02)(520.3852, 917.74)(521.3825, 254.12)(266.2331, 5137.54)(267.2368, 782.04)(268.2299, 233.67)(249.2066, 34341.03)(250.2101, 5085.53)(251.2097, 740.2)	138164.8	48968.61	128899.6	43658.61	139609.3	22706.27	138803	61659.15	0.29	0.323091	0.424408	-0.24	0.490013	0.748114
COMPOUND_249_0978a1_3501186	Hilic	+	249.0978	1.35				(272.0891, 779.21)(250.1052, 16080.58)(251.1084, 1797.74)(252.108, 195.53)	59730.91	12552.48	59180.26	9719.796	53796.93	12649.34	61783.46	13117.93	-0.45	0.12197	0.193297	0.62	0.07453	0.328457
COMPOUND_250_1211a8_072027	Rp	+	250.1211	8.07				(273.1108, 571.96)(274.1224, 132.33)(275.1207, 371.49)(251.1284, 10775.26)(252.134, 1447.77)(253.1393, 455.92)	46344.68	8576.673	42190.54	8741.787	46780.86	6231.903	47569.88	8196.722	0.09	0.754316	0.821829	0.57	0.094989	0.36381
COMPOUND_250_1215a8_709986	Rp	+	250.1215	8.71	Furan fatty acid 268.131	3	20 eV: 209.1195(100), 153.0548(30), 57.0699(20)	(251.1286, 63420.49)(252.1316, 10166.53)(253.1359, 1714.61)	77844.51	42200.03	76906.8	33618.85	99497.79	21759.37	76799.65	34930.69	0.67	0.010227	0.021626	-0.73	0.017232	0.194029
COMPOUND_251_152a9_54126	Rp	-	251.1520	9.54				(250.1446, 13312.14)(251.1477, 1958.07)(252.1488, 201.28)(501.2917, 17223.5)	59233.68	8139.395	64172.54	7507.028	68091.93	36740.31	62042.49	13945.79	0.10	0.734624	0.807011	-0.49	0.158543	0.468876
COMPOUND_251_1658a8_569771	Rp	+	251.1658	8.57				(274.1556, 86.23)(275.164, 330.32)(252.1728, 16653.78)(253.1774, 3535.15)(254.1769, 1187.84)	99214.46	26305.94	114239.8	31468.13	78894.89	30970.15	88641.42	33192.69	-0.42	0.103786	0.16827	-0.25	0.40295	0.691103
COMPOUND_252_0994a7_5606976	Rp	-	252.0994	7.56				(251.0923, 4562.52)(252.0965, 903.83)(253.1019, 299.18)	37760.89	4361.199	39311.11	3612.031	39601.79	4394.04	37507.25	3899.352	0.48	0.110578	0.177746	-0.92	0.011672	0.167839
COMPOUND_252_0996a7_1789227	Rp	-	252.0996	7.18				(365.0866, 585.93)(366.0921, 87.62)(251.092, 5683.06)(252.0965, 937.46)(253.1014, 295.97)	25502.45	2339.001	25649.78	2751.427	24925.29	2124.006	25323.49	2716.978	-0.19	0.520644	0.620038	0.08	0.832401	0.926681
COMPOUND_252_2085a9_942671	Rp	+	252.2085	9.94				(543.3886, 2345.58)(275.204, 1045.3)(276.2122, 174.71)(253.2173, 9889.61)(254.2198, 1906.85)	23305.79	5179.465	23577.83	6125.322	20786.46	5042.886	18282.76	4275.008	-0.06	0.836989	0.886862	-0.43	0.209467	0.518944
COMPOUND_252_2093a9_446959	Rp	+	252.2093	9.45				(275.2012, 2517.97)(276.2133, 860.34)(253.2169, 11511.54)(254.22, 2286.27)(255.2179, 132.01)	99942.19	20767.47	99035.26	22119.21	99098.79	14488.84	92499.53	20656.28	-0.03	0.919742	0.94911	-0.30	0.390762	0.690408
COMPOUND_254_2241a10_578774	Rp	-	254.2241	10.58	FA 16:1	1	10 eV: 253.2168(100), 134.3113(2)	(543.4109, 153.41)(289.1929, 648.78)(253.2172, 167518.45)(254.2204, 31055.0)(255.2257, 4244.29)(256.2298, 405.76)(507.4411, 222.07)(508.4426, 111.17)(299.2224, 3881.99)(300.2258, 852.12)(301.2168, 1500.21)(302.2192, 120.91)(621.4129, 347.06)	171265.7	147139.1	201916.5	200636.5	200185.6	154174.5	341040.6	197814.5	0.25	0.336217	0.43716	0.55	0.07281	0.326093
COMPOUND_254_2245a9_524177	Rp	+	254.2245	9.52				(277.2172, 41858.41)(278.2206, 8670.12)(272.2575, 1477.19)(273.2541, 434.23)(255.233, 7562.47)(256.2347, 1422.79)(257.2423, 204.92)	36029.47	17770.98	37693.57	28270.2	21470.64	6631.786	28965.09	19352.38	-1.10	0.000274	0.000791	0.66	0.058209	0.298605
COMPOUND_254_2248a9_955129	Rp	+	254.2248	9.96				(509.4721, 63.84)(510.4674, 40.85)(277.2164, 1244.27)(278.2155, 355.54)(272.2591, 2525.02)(273.2603, 644.32)(255.2326, 6055.49)(256.2354, 914.97)(257.2259, 818.09)(258.2313, 62.26)	61332.22	10237.26	65614.77	14180.46	56867.83	12476.63	56325.63	13479.73	0.10	0.717685	0.793046	-0.15	0.665614	0.844963
COMPOUND_255_2186a11_0484915	Rp	+	255.2186	11.05				(549.4074, 5248.76)(550.4033, 3000.61)(551.4234, 10208.4)(552.417, 6830.43)(278.2082, 541.02)(279.2097, 106.75)(533.4492, 857.31)(534.4502, 1312.98)(256.2275, 17871.9)(257.2307, 3310.59)(258.2365, 676.68)(528.4714, 412.04)	44402.77	21693.58	43678.18	21193.62	69435.73	20797.75	63346	25273.65	0.82	0.001377	0.003501	0.04	0.901421	0.955487
COMPOUND_255_22a10_746781	Rp	+	255.2200	10.75				(511.4401, 554.27)(549.389, 2409.67)(278.2111, 1418.28)(279.2066, 232.06)(533.4524, 379.7)(534.4624, 49.54)(294.1697, 1723.17)(273.2574, 3366.91)(274.2654, 875.39)(256.2278, 37909.12)(257.2311, 6615.48)(258.232, 894.69)(528.4587, 287.15)	23812.6	10205.72	28563.27	11175.42	39424.17	32660.17	37691.63	21804.51	0.47	0.10866	0.175037	-0.20	0.557658	0.781971
COMPOUND_255_2207a10_799834	Rp	+	255.2207	10.80				(511.4401, 408.05)(549.3963, 2788.83)(278.2097, 3191.31)(279.2188, 913.37)(533.4511, 897.32)(534.4577, 570.13)(535.4468, 450.43)(294.1699, 902.49)(273.2593, 629.74)(274.251, 55.27)(256.2278, 50172.44)(257.2314, 7844.86)(258.2325, 733.28)(528.462, 753.19)	24858.55	14793.3	30033.17	12037.58	45230	37478.49	41457.98	26167.63	0.82	0.004078	0.00938	-0.25	0.442916	0.718105

									1st trimester				3rd trimester									
									Control		Pre-eclampsia		Control		Pre-eclampsia		Time			Interaction		
Compound	Column	Ion_mode	Mass	Retention_	Identification	ID_level	MSMS_spectra	CompositeSpectrum	Mean	SD	Mean	SD	Mean	SD	Mean	SD	Value	raw p	FDR_p	Value	raw p	FDR_p
COMPOUND_255_2212a11_035777	Rp	+	255.2212	11.04				(549.4059, 5116.41)(550.4025, 3567.41)(551.4217, 8515.47)(552.4157, 4380.33)(278.2111, 545.8)(279.206, 47.96)(533.4238, 1341.06)(534.4347, 860.51)(256.228, 17997.06)(257.2305, 3213.3)(258.237, 496.12)(528.497, 606.07)	44372.8	34197.27	40165.38	22782.62	84947	30658.91	67099.08	30110.78	1.02	0.000171	0.000514	-0.30	0.31798	0.624317
COMPOUND_255_2569a10_489768	Rp	+	255.2569	10.49	Unknown	4	10eV: 256.2643(100), 88.0762(6)	(511.5197, 2870.62)(512.524, 1216.42)(513.5244, 339.27)(278.2467, 2138.05)(279.261, 241.71)(533.506, 457.01)(256.2644, 213531.2)(257.2674, 37654.84)(258.2706, 3834.53)(259.2733, 301.63)	#DIV/0!	#DIV/0!	#DIV/0!	#DIV/0!	#DIV/0!	#DIV/0!	#DIV/0!	#DIV/0!	1.04	0.000509	0.001416	-1.01	0.003908	0.094399
COMPOUND_255_2573a7_6793904	Rp	+	255.2573	7.68				(278.2488, 88.65)(256.2647, 174374.66)(257.2679, 29581.27)(258.2704, 2781.63)(259.2712, 243.74)	499622.4	458577.5	310813.3	389057	422698.3	282442	518434.1	443547.2	-0.01	0.958774	0.97523	0.82	0.016125	0.189073
COMPOUND_255_2941a7_561388	Rp	+	255.2941	7.56				(549.5707, 58.6)(256.3006, 321390.84)(257.3041, 58558.19)(258.3072, 5181.29)(259.31, 291.86)	3133687	294996.3	2960513	301013.6	3129539	211662.3	3163727	298409.7	0.00	0.99453	0.998768	0.82	0.014429	0.179521
COMPOUND_256_0944a5_3326526	Rp	-	256.0944	5.33				(255.0874, 109273.94)(256.0906, 13964.63)(257.0929, 2349.25)(258.0946, 198.04)(301.1004, 357.97)	92870.65	83370.2	67347.41	41595.17	51162.15	33703.32	39014.51	21711.87	-0.89	1.32E-09	1.41E-08	0.17	0.23743	0.542834
COMPOUND_256_1062a1_2656062	Hilic	+	256.1062	1.27				(279.0919, 289.38)(257.1139, 3778.88)(258.1162, 659.2)(259.1176, 205.45)(530.2454, 378.69)	10274.96	1702.144	10517.3	1807.567	12318.43	2448.64	13523.91	2388.223	0.84	0.000965	0.002523	0.38	0.197478	0.508252
COMPOUND_256_2201a9_888487	Rp	+	256.2201	9.89				(513.4352, 115.17)(257.2272, 45261.22)(258.2313, 9131.62)(259.2346, 790.33)	24901.05	13069.03	25037.03	10819.87	45210.93	15630.64	37441.86	25550.01	1.41	5.66E-07	3E-06	-0.93	0.002652	0.07283
COMPOUND_256_2206a8_639969	Rp	+	256.2206	8.64				(257.2272, 36086.58)(258.2303, 7905.09)(259.2329, 940.01)	16432.51	4908.077	16689.06	6783.403	40414.36	12329.76	37395.06	24712.49	1.51	2.25E-09	2.24E-08	-0.33	0.198737	0.510852
COMPOUND_256_2397a10_264209	Rp	-	256.2397	10.26				(547.4378, 59.82)(255.2328, 6331.71)(256.2361, 1163.75)(257.2379, 128.8)	28477.36	3742.09	30976.89	4333.508	21496.36	4681.613	22788	5000.771	-1.17	5.4E-06	2.28E-05	-0.26	0.362918	0.668267
COMPOUND_256_2398a10_160457	Rp	-	256.2398	10.16				(255.2327, 5437.9)(256.2361, 1107.43)(257.2358, 116.79)	15834.75	2572.764	16936.67	2662.926	15479.92	4286.132	14027.6	3214.798	-0.25	0.359286	0.461559	-0.74	0.029407	0.231966
COMPOUND_256_2398a10_853131	Rp	-	256.2398	10.85	FA 16:0	1	10 eV: 255.2333(100)	(291.2091, 2730.24)(292.2125, 367.13)(293.2054, 929.37)(294.2052, 74.64)(295.1913, 42.71)(557.464, 206.91)(255.2323, 847006.1)(256.2361, 142204.56)(257.2389, 14248.2)(258.2412, 1059.03)(511.4733, 1365.01)(512.4768, 584.89)(513.474, 35.86)(301.238, 15199.55)(302.2414, 2519.85)(303.2371, 918.41)(304.2405, 82.68)(625.4577, 3502.73)(626.4543, 1796.57)(627.4384, 692.39)	1689717	628838.2	2057245	684419.7	1680787	484636.3	1746359	622036.4	0.11	0.71199	0.788624	-0.50	0.155272	0.466404
COMPOUND_257_1012a1_4381056	Hilic	+	257.1012	1.44				(515.2297, 1065.77)(516.2355, 367.78)(280.0924, 1132.84)(281.0959, 357.32)(282.1034, 205.66)(258.1089, 7081.81)(259.1113, 819.18)(260.1094, 175.57)	13829.58	6033.565	12739.25	7229.818	21468.85	5033.676	22530.6	5939.553	0.96	2.2E-05	8.12E-05	0.26	0.298361	0.60748
COMPOUND_257_1035a6_1399736	Hilic	+	257.1035	6.14	Glycerophosphocholine	1	10 ev: 104.1075 (100), 258.1101 (60), 184.0692 (5)	(515.2132, 15051.87)(516.2167, 2881.31)(517.2181, 709.66)(518.2208, 115.63)(280.0928, 216027.34)(281.0957, 18748.97)(282.0974, 3125.41)(537.1953, 142999.12)(538.1983, 25458.69)(539.2004, 5243.96)(540.2017, 730.75)(275.1275, 163.35)(258.1107, 244179.05)(259.1138, 21217.03)(260.1152, 3687.9)(261.1188, 335.85)	802530.8	713954.1	497622.7	329782.2	461676.3	211280.6	1000174	754852.5	-0.43	0.128833	0.202182	1.35	0.000173	0.018576
COMPOUND_258_1986a10_330836	Rp	+	258.1986	10.33				(276.2304, 304.81)(259.2064, 5250.45)(260.2101, 1102.8)(261.2169, 488.86)	31873.4	11233.49	28645	9092.699	23610.75	15589.8	27730.75	14405.64	-1.19	6.3E-07	3.3E-06	0.17	0.500472	0.753664
COMPOUND_259_1783a0_9986215	Hilic	+	259.1783	1.00				(282.1674, 279.71)(541.3483, 914.65)(260.1861, 77558.66)(261.1894, 10986.93)(262.1918, 1666.97)	49776.7	26219.1	51667.59	29477.26	47822.14	22552.18	77853.54	43588.39	-0.04	0.879614	0.920296	0.79	0.014572	0.179825
COMPOUND_259_1794a3_9611745	Rp	+	259.1794	3.96	Hexanoylcarnitine	1	10eV: 260.1852(100), 85.0277(75), 201.1116(40), 60.0814(18)	(260.1868, 15609.43)(261.1903, 2352.62)(262.1901, 414.4)	21282.21	9827.492	19464.03	9358.527	17579.92	9641.404	28981.83	15013.49	-0.28	0.291732	0.390827	1.00	0.002803	0.074044
COMPOUND_260_1379a3_1481564	Rp	+	260.1379	3.15				(261.1454, 18073.75)(262.1486, 2307.65)(263.1504, 368.61)	76063.49	24796.31	73380.22	26785.41	59017.17	56724.15	39637.76	39961.56	-1.17	4.33E-06	1.86E-05	0.05	0.844236	0.93246
COMPOUND_261_1579a3_1249304	Hilic	+	261.1579	3.12				(262.1653, 6772.12)(263.1685, 1208.35)(264.1685, 287.97)	30201.87	11010.6	32091.48	8480.484	20906.29	4539.232	31177.19	9254.666	-1.04	3.7E-05	0.000128	0.93	0.000997	0.051018
COMPOUND_262_194a9_176586	Rp	+	262.1940	9.18				(285.1852, 598.58)(286.1915, 162.35)(263.2012, 29168.34)(264.2042, 5755.56)(265.2034, 1495.53)(266.214, 150.35)	49497.88	28368.05	63261.23	57396.74	59580.69	58508.15	84611.54	58373.6	0.52	0.019506	0.038669	-0.07	0.796985	0.91372
COMPOUND_262_2302a10_459587	Rp	+	262.2302	10.46				(285.2185, 1797.91)(280.2646, 11440.07)(263.2381, 156543.6)(264.2413, 31193.8)	116749	59452.16	134132.3	85572.8	126471.1	60819.97	223484	114122.6	0.24	0.367628	0.469863	0.68	0.033571	0.247063
COMPOUND_262_2303a10_443133	Rp	+	262.2303	10.44				(525.4533, 217.91)(285.2124, 836.66)(280.2646, 24112.17)(263.2384, 91457.25)(264.2418, 18173.09)	98994.72	55238.48	121054.8	60093.76	100580.6	60292.73	134179.5	100553.9	0.03	0.920775	0.94911	-0.08	0.82958	0.926681
COMPOUND_264_1112a1_7612511	Hilic	+	264.1112	1.76				(287.1007, 44198.33)(288.1035, 6859.44)(289.1033, 1044.91)(290.0988, 118.59)(551.2114, 7184.15)(552.2151, 2237.54)(553.2168, 627.92)(554.218, 120.39)(265.1183, 12423.71)(266.1214, 1975.37)(267.1242, 455.84)(268.1315, 45.56)(546.2571, 252.66)	251726.7	131978.3	233564.1	164354.2	364337.3	222328.9	296789.4	212138.8	0.50	0.064597	0.111133	-0.16	0.616247	0.816971
COMPOUND_264_112a3_427942	Rp	+	264.1120	3.43				(287.0941, 1775.38)(288.0975, 208.56)(303.0675, 824.22)(265.1189, 20162.09)(266.122, 2800.05)(267.1209, 274.03)	92358.4	50601.29	83204.49	57841.36	132338.5	73676.05	110052.8	75387.07	0.52	0.059929	0.103656	-0.11	0.737621	0.888668
COMPOUND_264_1371a7_0381002	Rp	+	264.1371	7.04				(287.1257, 1431.96)(288.1276, 330.97)(289.135, 233.44)(303.0996, 3189.27)(265.1444, 11729.56)(266.1484, 1946.25)(267.153, 404.84)	37186	10143.3	36726.13	11755.86	38799.29	14371.85	34309.43	8337.977	0.07	0.815834	0.870326	-0.42	0.234775	0.54116
COMPOUND_264_2447a10_448037	Rp	+	264.2447	10.45				(567.4494, 113.66)(287.2374, 6469.49)(288.2413, 1563.61)(282.2786, 602.31)(265.2518, 6542.12)(266.2567, 1165.61)	30436.25	6844.763	31057.16	10193.93	34260	6351.317	40024.77	9602.071	0.50	0.056703	0.098302	0.63	0.04438	0.272788
COMPOUND_264_2459a10_622422	Rp	+	264.2459	10.62				(529.5023, 309.48)(567.4587, 197.83)(287.2356, 23784.35)(288.2411, 4271.11)(289.2484, 702.66)(265.2533, 105627.15)(266.2563, 20294.19)(267.264, 3419.45)	66596.92	50349.61	65340	65157.41	131421.1	52859.17	140618	62300.45	1.05	7.8E-05	0.000246	0.19	0.52806	0.7696
COMPOUND_264_2463a10_692466	Rp	+	264.2463	10.69	no msms			(529.5249, 466.56)(567.4615, 551.93)(287.2371, 3199.52)(288.241, 941.72)(289.2466, 236.4)(265.2536, 33804.48)(266.257, 6165.94)(267.2642, 1381.93)(268.266, 136.79)	103348	54628.75	102701	58946.85	100482	42521.38	212343.8	95217.85	-0.03	0.89068	0.926471	1.27	5.53E-05	0.009576
COMPOUND_265_1662a10_830807	Rp	+	265.1662	10.83				(266.1733, 25593.86)(267.1772, 4422.21)(268.1796, 537.33)	14714.83	5482.054	16241.68	4680.879	41780.11	23229.75	36014.23	20089.33	0.99	1.88E-06	8.8E-06	0.17	0.446283	0.72091
COMPOUND_266_0517a3_329751	Hilic	+	266.0517	3.33				(289.0427, 238.28)(290.044, 61.8)(555.0963, 113.28)(267.0594, 4299.35)(268.0622, 398.62)(269.0631, 82.52)	21430.57	2973.34	22455.67	4085.338	25816.38	3359.709	28513.46	5921.824	0.63	0.009172	0.019596	0.52	0.064677	0.307286
COMPOUND_266_1367a0_7758269	Hilic	+	266.1367	0.78				(533.2753, 226.49)(289.1264, 1791.15)(290.1164, 515.55)(291.1095, 35.03)(284.1707, 46862.45)(285.1753, 6699.67)(286.1791, 1312.68)(267.1441, 30950.32)(268.1472, 4152.77)(269.1486, 679.73)	#DIV/0!	#DIV/0!	#DIV/0!	#DIV/0!	#DIV/0!	#DIV/0!	#DIV/0!	#DIV/0!	0.22	0.470078	0.573399	-0.01	0.987866	0.994046
COMPOUND_266_1566a10_47078	Rp	+	266.1566	10.47				(555.3131, 1165.99)(267.1642, 11201.08)(268.166, 2196.57)(269.1705, 410.1)	22265.38	6659.349	24568.95	5946.83	28633.09	7258.67	27775.96	8018.915	0.65	0.021669	0.042214	-0.34	0.301363	0.609767
COMPOUND_266_2034a9_926917	Rp	+	266.2034	9.93				(571.3667, 614.91)(572.34, 254.09)(267.2112, 20710.61)(268.2154, 4465.75)(269.2093, 1338.09)	41838.92	15605.74	46019.67	16424.86	52127	4344.554	49496.38	14007.43	-0.92	5.57E-05	0.000183	-0.28	0.276003	0.585272
COMPOUND_266_2623a10_916413	Rp	+	266.2623	10.92				(533.5286, 1667.57)(555.5371, 563.82)(556.5392, 1558.0)(557.5308, 620.55)(284.2964, 134186.98)(267.2691, 28069.38)(268.2726, 6265.38)(269.2741, 737.48)(550.5365, 976.76)	83421.42	39956.36	70757.72	36540.45	116919.2	110538.6	110206	74923.22	-0.41	0.162349	0.244554	0.64	0.069255	0.32157
COMPOUND_266_9886a3_6063783	Rp	+	266.9886	3.61				(534.9777, 127.52)(556.9574, 121.36)(267.996, 6261.71)(268.9983, 852.46)(269.9939, 327.18)(271.0004, 29.2)	45564.73	22136.23	38053.18	21462.14	40339.31	17470.2	39211.66	21318.32	0.02	0.931007	0.956096	-0.15	0.616309	0.816971
COMPOUND_267_0934a9_219599	Rp	+	267.0934	9.22				(306.0525, 193.92)(268.1, 5282.46)(269.105, 967.53)(270.1001, 1617.85)(271.1022, 346.63)(272.0954, 515.34)(552.2084, 70.77)(553.2166, 2081.55)	26257.67	6870.67	26394.56	5461.257	32009.07	5558.012	33411.25	19019.3	0.84	0.003368	0.007917	-0.38	0.240376	0.545142
COMPOUND_267_1482a7_526463	Rp	+	267.1482	7.53				(268.1552, 16608.89)(269.1586, 2703.73)(270.1604, 398.09)(552.3283, 274.02)	59110.79	16478.58	50894.02	15752.87	61656.07	11853.58	62318.88	15523.59	0.16	0.562347	0.659586	0.65	0.056164	0.29488
COMPOUND_268_0813a2_3771577	Hilic	+	268.0813	2.38	Inosine	2	10 eV: 137.0456 (100)	(537.1691, 1726.33)(538.1707, 443.73)(539.1715, 68.77)(291.0704, 111602.04)(292.0733, 13531.96)(293.0755, 1997.05)(294.0768, 214.54)(559.1511, 14656.6)(560.1541, 3730.91)(561.1564, 780.52)(562.1562, 125.17)(269.0886, 20190.17)(270.0918, 2829.3)(271.0936, 439.82)	158119.2	248594.7	164767.1	217412.4	163123.2	129236.2	117571.6	109772.8	0.36	0.221072	0.3145	-0.24	0.49784	0.752946
COMPOUND_268_131a8_702773	Rp	-	268.1310	8.70	Furan fatty acid 268.131	3	10 eV: 223.1334(100), 267.1235(90), 179.1442(14), 41.7169(2)	(267.1236, 60219.71)(268.1268, 8973.98)(269.1301, 1410.36)(270.1319, 123.41)	86452.58	46642.51	89938.37	39554.97	107489.4	32101.35	82314.68	38697.05	0.52	0.042008	0.0752	-0.80	0.007662	0.138042
COMPOUND_268_1327a8_711904	Rp	+	268.1327	8.71				(575.2197, 875.66)(576.2186, 583.32)(291.1208, 5935.01)(292.1245, 994.15)(293.1211, 279.05)(307.0888, 942.19)(308.096, 199.59)(309.0873, 53.12)(269.1392, 5656.09)(270.1421, 1133.47)(271.1309, 169.96)(135.0791, 532.04)(135.5744, 26.15)(136.0765, 615.87)	17531.83	8087.169	17559.18	6968.238	20991.43	5142.325	17616.06	6810.85	0.82	0.002215	0.005445	-0.79	0.010926	0.164558
COMPOUND_268_2201a10_614021	Rp	+	268.2201	10.61				(575.398, 1745.98)(269.2278, 195819.66)(270.2307, 40503.34)(271.2276, 5107.43)(272.2238, 526.75)	#DIV/0!	#DIV/0!	#DIV/0!	#DIV/0!	#DIV/0!	#DIV/0!	#DIV/0!	#DIV/0!	1.17	4.2E-06	1.81E-05	-0.01	0.97693	0.987453
COMPOUND_268_2385a10_768955	Rp	-	268.2385	10.77				(571.4342, 662.58)(381.2303, 1735.94)(382.2327, 441.83)(267.2325, 12332.23)(268.2362, 2313.51)(269.2414, 315.25)(313.2376, 1091.37)(314.2407, 248.66)(649.4412, 1082.66)	24036.76	15252.24	35055.84	20930.93	26650.67	13195.86	33186.77	17599.29	0.27	0.232746	0.327385	0.27	0.306804	0.613089

									1st trimester				3rd trimester									
									Control		Pre-eclampsia		Control		Pre-eclampsia		Time			Interaction		
Compound	Column	Ion_mode	Mass	Retention_	Identification	ID_level	MSMS_spectra	CompositeSpectrum	Mean	SD	Mean	SD	Mean	SD	Mean	SD	Value	raw p	FDR_p	Value	raw p	FDR_p
COMPOUND_269_2738a10_468469	Rp	+	269.2738	10.47				(539.5505, 8488.55)(540.5562, 4108.45)(541.5635, 1093.02)(542.5582, 129.05)(292.2623, 32462.6)(293.2656, 5718.31)(294.2705, 750.73)(561.5331, 2003.01)(562.5355, 838.48)(563.5336, 400.07)(308.2326, 820.92)(309.2261, 195.31)(310.237, 1909.04)(287.3057, 1217.78)(270.28, 296042.6)(271.2834, 53707.76)(272.2862, 5496.5)(273.2876, 447.06)(556.562, 1126.98)	1236474	147074.4	1217178	130153.6	1471131	68317.29	1493665	78685.69	1.33	3.35E-09	3.12E-08	0.31	0.180301	0.493854
COMPOUND_270_0855a1_6402763	Hilic	+	270.0855	1.64				(541.187, 259.76)(293.075, 5495.54)(294.0794, 889.51)(295.0777, 97.07)(288.1229, 440.21)(271.0928, 4251.63)(272.0971, 738.79)(273.0974, 120.5)	24602.32	5919.543	24824.67	5803.834	22425.71	6821.126	29586.7	7513.987	-0.34	0.203623	0.294704	1.03	0.001501	0.06002
COMPOUND_270_1994a7_383529	Rp	+	270.1994	7.38				(293.191, 550.08)(294.2053, 163.03)(288.233, 4369.72)(289.2355, 934.41)(290.2381, 161.93)(271.2064, 30096.73)(272.21, 6370.14)(273.2139, 917.15)(274.2261, 96.51)	149276.6	62584.26	155229.8	54974.03	65747.14	31549.7	76335.86	43219.41	-1.05	1.08E-06	5.32E-06	-0.31	0.171894	0.487538
COMPOUND_270_2189a9_938386	Rp	-	270.2189	9.94				(305.1873, 187.44)(383.1971, 1019.37)(384.1965, 136.3)(269.2119, 5951.67)(270.2157, 1277.0)	18946.9	4740.146	20645.37	5127.818	15194.46	3386.858	17087.16	5050.785	-0.94	0.0008	0.002124	0.18	0.571729	0.789098
COMPOUND_270_2195a10_099287	Rp	-	270.2195	10.10				(305.1865, 103.8)(269.2118, 6290.51)(270.2155, 1255.34)	14024.14	3634.286	14637.15	3546.203	11548.8	975.6852	11453.8	1467.394	-0.68	0.003261	0.00769	-0.30	0.253353	0.565462
COMPOUND_270_2195a9_591034	Rp	+	270.2195	9.59				(579.4014, 1257.27)(293.2071, 1720.05)(294.2109, 580.6)(295.192, 685.87)(288.2542, 2438.58)(289.2551, 210.15)(290.2639, 463.09)(271.227, 5817.47)(272.2294, 1185.58)(273.2351, 224.72)	25727.74	6384.093	27786.11	9691.093	27011.17	4209.345	27512.2	7510.919	0.21	0.455949	0.561091	-0.13	0.700573	0.863364
COMPOUND_270_2217a9_94457	Rp	+	270.2217	9.94				(541.4432, 127.4)(579.4145, 3745.43)(293.2097, 2151.68)(294.2095, 766.71)(295.2203, 1757.58)(288.2481, 1205.96)(289.241, 336.3)(290.236, 381.22)(271.227, 9646.67)(272.2298, 1602.74)(273.2337, 858.51)	29988.76	9285.899	26994	8361.541	25753.3	4731.681	27674.64	6972.791	-0.32	0.212508	0.304821	0.50	0.099107	0.368908
COMPOUND_270_2555a10_969355	Rp	-	270.2555	10.97				(305.2212, 163.75)(269.2483, 41730.93)(270.2515, 8595.62)(271.2542, 876.15)(315.2532, 1104.94)(316.2544, 235.95)(653.4723, 4190.38)	53713.02	18206.82	73328.02	40287.28	78133.29	48884.88	69175.35	32805.84	0.82	0.006314	0.013967	-0.86	0.016057	0.189073
COMPOUND_270_2555a11_014253	Rp	-	270.2555	11.01	FA 17:0	1	10 eV: 69.2486(100), 228.1497(10), 75.2181(2)	(305.2191, 174.22)(269.2482, 65445.16)(270.2514, 13027.23)(271.2545, 1594.61)(315.2539, 1405.5)(316.2578, 355.08)(317.2546, 41.8)(653.4716, 4713.61)	74462.62	31068.39	100038.1	45619.55	100583.6	43970.21	96059.8	46019.23	0.67	0.024428	0.046938	-0.78	0.029459	0.231966
COMPOUND_272_215a6_9161153	Rp	+	272.2150	6.92				(273.2223, 10168.73)(274.2252, 2180.95)(275.2296, 244.62)	23283.43	9706.782	27113.76	11629.08	13363	#DIV/0!	14114.45	3649.723	-1.28	2.67E-10	3.62E-09	-0.12	0.55836	0.781981
COMPOUND_272_2343a9_512525	Rp	-	272.2343	9.51				(271.2276, 5872.44)(272.2304, 1097.04)(273.2216, 139.72)	20718.04	5848.43	21961.17	7182.554	19773.64	3683.737	20699.02	7801.899	-0.09	0.763308	0.828819	-0.20	0.543746	0.776977
COMPOUND_272_2347a9_940352	Rp	-	272.2347	9.94				(307.1949, 813.03)(271.2274, 11261.97)(272.2312, 2271.09)(273.2312, 188.55)(317.2316, 78.12)	17231.14	4510.008	18370.47	6358.174	13240.25	3005.769	16419.67	7470.796	-0.73	0.009216	0.019673	-0.05	0.867701	0.9447
COMPOUND_272_2348a10_4309225	Rp	-	272.2348	10.43				(307.2036, 448.54)(308.2068, 125.7)(309.2024, 348.5)(271.2275, 38872.18)(272.231, 6466.56)(273.2324, 740.86)(274.234, 66.86)(543.4386, 147.66)	92223.83	17582.95	94674	20056.32	67128	12855.33	72694.16	16223.74	-1.45	3.53E-08	2.51E-07	0.34	0.212665	0.520001
COMPOUND_273_1803a6_5916677	Hilic	+	273.1803	6.59				(296.1692, 1247.85)(297.1717, 201.59)(274.1881, 23034.67)(275.1909, 2974.71)(276.1944, 486.76)	12493.7	7646.577	9913.52	6742.453	42797.92	24710.83	27864.81	16920.59	1.42	2.66E-09	2.58E-08	-0.38	0.12007	0.410243
COMPOUND_273_1932a7_613201	Rp	-	273.1932	7.61				(308.1466, 66.94)(386.1813, 469.89)(272.1865, 3886.84)(273.1897, 806.8)(274.186, 274.78)	13492.55	2285.842	14491.42	2763.823	12272.46	1701.964	12848.66	2845.422	-0.23	0.395936	0.500048	-0.61	0.058069	0.298567
COMPOUND_276_0826a5_3917756	Hilic	+	276.0826	5.39				(277.0898, 14265.17)(278.0931, 1474.74)(279.0944, 321.01)	11130.25	5429.336	12452.35	7905.177	12911.07	5845.862	11873.17	7146.562	0.61	0.027116	0.051575	-0.37	0.246355	0.555348
COMPOUND_276_172a8_981627	Rp	-	276.1720	8.98				(275.165, 6989.59)(276.169, 1450.35)(277.1732, 178.93)	26897.3	6409.271	29818.48	5573.992	26327.29	5078.233	25445.98	6619.767	-0.12	0.670847	0.758407	-0.66	0.052846	0.286771
COMPOUND_276_1736a8_209234	Rp	+	276.1736	8.21				(299.1628, 509.73)(300.1607, 116.06)(277.1807, 8912.24)(278.1843, 2114.25)(279.1879, 486.67)	16258.18	5458.582	15915.64	6696.543	14859.31	3435.505	15752.38	4875.189	0.12	0.661057	0.748467	0.33	0.306224	0.613089
COMPOUND_276_1738a9_035188	Rp	+	276.1738	9.04				(299.1625, 1821.02)(300.1649, 397.68)(301.1547, 22.63)(294.2072, 4355.49)(295.2203, 1520.62)(315.1426, 384.34)(316.1349, 58.09)(317.1473, 917.26)(277.1806, 16748.33)(278.1852, 3463.44)(279.1925, 1055.04)(280.1886, 90.07)	85567.63	33342.6	82586.7	37769.87	83560.5	27597.45	94013.46	37318.99	0.22	0.462527	0.567635	0.10	0.776863	0.905535
COMPOUND_276_174a9_034545	Rp	+	276.1740	9.03				(299.1624, 1583.63)(300.1651, 488.56)(301.159, 52.85)(315.1481, 394.78)(316.1394, 59.14)(317.1496, 852.86)(294.2078, 4071.77)(295.2208, 1588.92)(277.1811, 13403.19)(278.186, 3074.46)(279.1922, 996.68)(280.1857, 82.28)	88869.56	38652.18	89892.48	33036.13	86407.36	30208.2	85864.47	33162.02	0.09	0.768629	0.83299	-0.35	0.332081	0.636996
COMPOUND_276_209a9_656247	Rp	+	276.2090	9.66				(277.2177, 41620.87)(278.2208, 8530.68)	43102.68	20908.03	49645.33	29331.94	81196.5	65795.87	99307.92	83485.08	-0.84	0.000691	0.001869	-0.21	0.452895	0.72489
COMPOUND_276_2096a9_609907	Rp	+	276.2096	9.61				(591.4012, 925.15)(592.4058, 393.74)(294.2423, 945.0)(277.2176, 57559.31)(278.2208, 12572.54)	69931.81	31001.43	93837.89	51530.45	115072	83103.19	131255.7	66113	-0.74	0.001728	0.004348	-0.49	0.077782	0.333235
COMPOUND_277_169a6_334597	Rp	+	277.1690	6.33				(278.1759, 14308.44)(279.1783, 2821.84)(280.1832, 395.59)	26688.51	12316.05	28919.07	14200.83	13926.67	2486.093	15668	8475.036	-1.24	2.5E-09	2.46E-08	-0.18	0.399626	0.690408
COMPOUND_278_0874a7_708572	Rp	+	278.0874	7.71				(301.0752, 411.59)(302.0821, 144.11)(303.0802, 178.86)(317.063, 97.43)(279.0941, 13901.83)(280.0974, 2803.41)(281.1002, 299.69)	99128.43	10733.43	93866.67	9717.729	95452.07	7540.242	97055.72	9484.616	-0.31	0.301177	0.401807	0.66	0.06715	0.315046
COMPOUND_278_1529a9_435037	Rp	+	278.1529	9.44				(301.1423, 5902.41)(302.1439, 1255.97)(303.1493, 226.84)(317.1179, 314.31)(318.1116, 109.36)(319.1176, 179.13)(279.1599, 45370.24)(280.1637, 8312.96)(281.1688, 1636.57)(574.3443, 314.24)(575.3396, 115.53)(576.3328, 126.12)	149246.4	26236.08	141325.2	27295.52	150805.1	24673.54	152400.7	25271.8	0.11	0.700775	0.782034	0.31	0.373319	0.675816
COMPOUND_278_1531a9_357016	Rp	+	278.1531	9.36	Phthalic acid Mono-2-ethylhexyl Este	2	20 eV: 149.0234(100), 57.0695(30), 183.0147(4), 279.0904(3), 101.0984(2)	(301.1421, 14436.1)(302.1444, 2794.49)(303.1485, 413.9)(579.2999, 608.73)(580.3093, 277.27)(581.2925, 76.26)(317.1151, 802.26)(318.1191, 253.49)(319.1125, 172.09)(279.1605, 96397.19)(280.1637, 16241.28)(281.1688, 2456.58)	330958	31120.92	324196.2	36972.98	331464	28564.13	325619.2	34782.24	0.10	0.736774	0.808584	-0.09	0.806795	0.917894
COMPOUND_278_1637a4_23307	Rp	+	278.1637	4.23				(279.1712, 84385.31)(280.1743, 14206.5)(281.1771, 1749.35)(282.1797, 184.44)(574.3615, 247.91)	146416.6	63134.57	169284.8	95899.41	277181	115287.1	275772.8	137782.3	0.90	0.000799	0.002124	-0.14	0.631835	0.828008
COMPOUND_278_1637a1_5383792	Hilic	+	278.1637	1.54				(557.3272, 64.49)(558.3184, 2646.74)(301.1529, 2624.56)(302.1558, 564.54)(303.1603, 27.4)(579.3411, 373.32)(279.1709, 46646.07)(280.174, 8146.26)(281.1782, 1024.97)(574.3316, 142.79)	55249.6	22549.88	67739.67	37764.27	97442.31	38773.45	101134.1	53641.72	0.81	0.003453	0.008091	-0.22	0.490219	0.748114
COMPOUND_278_1637a4_3144116	Rp	+	278.1637	4.31				(579.3094, 72.99)(279.1714, 61657.52)(280.1745, 10260.07)(281.1753, 1362.33)(282.1662, 139.67)(574.3576, 68.36)	144375	185366.5	220372	277420.3	184321.2	135390.4	151441.3	133729	0.21	0.468045	0.572535	-0.53	0.127595	0.424221
COMPOUND_278_188a9_038569	Rp	-	278.1880	9.04				(277.1808, 13204.08)(278.1843, 2705.09)(279.1859, 409.01)(280.1864, 39.51)(323.1861, 663.59)(324.1876, 55.49)	66540.28	10071.55	71887.78	10393.17	61019.29	8454.916	62493.63	9830.477	-0.50	0.067211	0.115136	-0.40	0.205727	0.516497
COMPOUND_278_2244a10_500361	Rp	-	278.2244	10.50	FA 18:3	2	10 eV: 277.2163(100), 178.6658(1),	(313.1939, 893.79)(314.1977, 81.43)(315.1907, 469.74)(277.2171, 67969.73)(278.2204, 13554.81)(279.2239, 1934.21)(280.2219, 168.1)(555.4215, 239.5)(323.2168, 541.67)	148401.9	102480.5	194620.9	137249.8	189299.9	110898.2	227376	130507	0.42	0.121906	0.193297	-0.06	0.841703	0.93246
COMPOUND_278_2254a9_403822	Rp	+	278.2254	9.40				(296.2569, 813.09)(279.2326, 24512.28)(280.2358, 4864.62)(281.2434, 1682.18)(282.2343, 106.84)	46078.06	24828.76	59851.76	31577.75	63536.67	46629.42	95073.09	50079.81	-0.90	0.000238	0.000701	-0.19	0.494012	0.750179
COMPOUND_278_2255a10_500704	Rp	+	278.2255	10.50				(557.4462, 300.66)(595.4161, 2612.56)(596.4126, 1705.44)(597.4296, 6439.21)(301.2077, 9565.57)(302.2085, 3240.42)(303.2, 757.85)(296.259, 32994.05)(297.2634, 7305.86)(298.2723, 1601.44)(279.2331, 39816.4)(280.2366, 7864.84)	99773.92	41017.21	104859.3	49724.57	139912.9	48338.49	161357.2	69892.96	0.71	0.00886	0.019026	0.18	0.565203	0.785799
COMPOUND_278_2263a9_637026	Rp	+	278.2263	9.64				(557.4574, 359.86)(301.2178, 286633.75)(302.2211, 56658.69)(279.2331, 495819.8)(280.2369, 90665.09)(281.2406, 10835.35)(282.245, 953.62)	788745.7	412973.1	1036400	542822.9	384938.5	689475.7	367026.7	711868.6	-0.65	0.011236	0.023452	-0.66	0.030413	0.234557
COMPOUND_279_1481a1_2006848	Hilic	+	279.1481	1.20				(559.2983, 927.87)(280.155, 8786.51)(281.158, 1658.22)(282.1618, 272.37)(576.3282, 2097.79)	24921.78	16395.19	22427.58	17499.57	15509.83	8521.489	20739.37	10247.7	-0.74	0.003234	0.007643	0.24	0.399987	0.690408
COMPOUND_280_1059a2_741435	Rp	+	280.1059	2.74				(281.1145, 7840.72)(282.1166, 1260.82)(283.1207, 243.81)	19035.72	7540.722	20681.79	7100.589	21509.77	6018.814	24593.32	9601.749	0.56	0.025944	0.049514	-0.05	0.874238	0.946531
COMPOUND_280_2405a10_7077675	Rp	-	280.2405	10.71	FA 18:2	1	10 eV: 279.2321(100), 201.5305(1), 80.2731(1)	(595.4468, 233.49)(315.2093, 4364.02)(316.2127, 935.55)(317.207, 1409.89)(318.2093, 354.01)(279.2324, 597695.0)(280.2361, 103159.23)(281.2394, 11879.25)(282.2423, 1246.29)(559.4758, 217.2)(327.2328, 304587.3)	1030057	507266.5	1295928	680996.6	924758.5	438717	1240135	633049.7	-0.09	0.740229	0.811585	0.06	0.854121	0.937825
COMPOUND_280_2413a10_715105	Rp	+	280.2413	10.72				(561.4837, 826.65)(562.4736, 86.21)(298.2747, 40154.15)(299.2768, 8376.51)(300.2835, 1634.54)(281.2488, 23116.74)(282.2528, 4766.0)	75605.89	26168.52	79721.89	35700.59	91812.57	30155.88	114336.9	44622.15	0.44	0.097989	0.160007	0.44	0.162723	0.476531
COMPOUND_280_2416a9_988901	Rp	+	280.2416	9.99				(303.2328, 799857.44)(298.2781, 364.21)(299.2888, 42.09)(281.2482, 51955.9)(282.2517, 9838.2)	546353.7	618655.3	651124.4	767981	243370.4	62145.9	312395.4	414566.3	-0.44	0.136851	0.21225	-0.04	0.909966	0.958935

									1st trimester				3rd trimester									
									Control		Pre-eclampsia		Control		Pre-eclampsia		Time			Interaction		
Compound	Column	Ion_mode	Mass	Retention_	Identification	ID_level	MSMS_spectra	CompositeSpectrum	Mean	SD	Mean	SD	Mean	SD	Mean	SD	Value	raw p	FDR_p	Value	raw p	FDR_p
COMPOUND_280_2416a10_028688	Rp	+	280.2416	10.03				(303.2327, 810438.75)(298.2754, 361.29)(299.2772, 77.87)(281.2481, 50366.52)(282.2514, 9954.42)	556628.3	887871.7	405069.1	676846.9	223828.1	143090.1	298760.6	470194.2	-0.03	0.912153	0.942711	0.05	0.883915	0.951974
COMPOUND_280_2417a10_084801	Rp	+	280.2417	10.08				(599.4343, 4429.37)(600.4358, 1640.4)(601.4286, 2120.54)(303.233, 7284.5)(298.2739, 1714.24)(281.2483, 33277.5)(282.2518, 6706.07)	84006.18	74959.96	76587.14	32828.53	80594.38	48901.03	94145.62	59730.82	0.11	0.70151	0.782467	0.04	0.90827	0.958319
COMPOUND_280_243a10_235375	Rp	+	280.2430	10.24				(599.4398, 62135.09)(600.4438, 20635.38)(601.4437, 7315.62)(298.2753, 949.87)(281.2481, 20838.52)(282.2517, 3941.23)	147067.4	84063.69	109918.9	54050.74	219756.4	34969.19	215317.9	55139.57	0.54	0.037037	0.0677	0.59	0.055509	0.294133
COMPOUND_281_1132a4_2414556	Hilic	+	281.1132	4.24				(304.1024, 2565.79)(305.1045, 362.55)(282.1205, 4524.84)(283.1239, 689.92)(284.128, 119.91)	25092.7	4256.56	25365.83	4704.075	40629.93	7683.702	43117.25	7053.76	1.44	2.49E-10	3.42E-09	0.13	0.556593	0.781554
COMPOUND_281_2718a10_601354	Rp	+	281.2718	10.60	Oleamide	1	10 eV: 282.2817(100), 57.0699(82), 148.5324(78), 95.0881(50)	(563.5522, 16343.6)(564.5557, 6575.87)(565.557, 1517.28)(304.2622, 35179.18)(305.2637, 7783.41)(306.2707, 1176.51)(320.2363, 1760.85)(282.2806, 382545.72)(283.2835, 73064.03)(284.2894, 10231.9)(580.5751, 1057.19)	234871.6	116440.3	317577.1	189581.7	561195.8	462092.8	554393.1	493933	1.02	0.000414	0.001165	-0.98	0.003982	0.094399
COMPOUND_282_0257a3_3187468	Hilic	+	282.0257	3.32				(305.016, 111.06)(283.0332, 5345.54)(284.0364, 547.66)(285.0332, 429.22)	22630.91	4468.321	24758.24	5322.115	26547	5031.69	30793.49	6791.788	0.61	0.004549	0.010337	0.27	0.261476	0.572854
COMPOUND_282_1299a10_470434	Rp	+	282.1299	10.47				(587.2442, 1495.4)(283.137, 6062.18)(284.1388, 1194.66)(285.137, 4274.02)(286.1398, 757.53)(287.132, 1786.83)(288.1252, 113.66)	31913.51	6894.494	30381.22	5791.589	40060.93	6817.313	39568.77	6879.379	1.19	6.85E-06	2.82E-05	-0.02	0.932826	0.970312
COMPOUND_282_2359a8_031316	Rp	+	282.2359	8.03				(321.195, 721.39)(322.1983, 289.87)(283.2433, 38151.75)(284.2463, 8597.07)(285.2549, 1540.88)	56633.33	24872.14	62171.51	38578.4	31266.71	9469.803	29683.94	12751.36	-0.91	9.29E-05	0.000288	-0.43	0.09223	0.359968
COMPOUND_282_2557a10_931676	Rp	-	282.2557	10.93	FA 18:1	1	10 eV: 281.2481(100)	(599.4821, 292.61)(317.2251, 7794.2)(318.2285, 1677.57)(319.2218, 2904.11)(320.2253, 595.48)(321.2181, 1086.27)(322.2187, 348.51)(281.248, 936368.7)(282.2519, 169994.42)(283.2553, 19343.86)(284.2597, 2150.62)(563.503, 496.53)(564.5011, 183.47)(565.5137, 109.49)(327.2533, 7541.66)(328.2562, 1413.36)(329.2539, 608.03)(330.2464, 79.47)(677.4872, 1741.84)	1319349	936930.5	1676081	1385607	1548106	751639.3	2075641	1197834	0.34	0.215327	0.30808	0.20	0.527781	0.7696
COMPOUND_282_2567a9_993883	Rp	+	282.2567	9.99				(305.2472, 67281.16)(306.2509, 13076.31)(283.2634, 17377.28)(284.2668, 3384.6)(285.2748, 620.8)	71551.79	31914.91	114060.3	71940.13	62421.08	31237.76	56740.84	17777.17	0.78	0.001758	0.004409	-0.22	0.446162	0.72091
COMPOUND_282_2567a10_931937	Rp	+	282.2567	10.93				(565.5281, 885.97)(566.5413, 2128.71)(567.5302, 1249.7)(300.2898, 13859.76)(301.2932, 2934.85)(302.2962, 469.21)(283.2643, 28274.93)(284.2687, 5724.94)(582.5613, 3935.71)	45561.92	19521.48	46580.74	24675.01	59060.29	22312.55	82953.75	29924.45	0.54	0.019645	0.038876	0.66	0.015558	0.186363
COMPOUND_282_2569a10_455742	Rp	+	282.2569	10.46				(565.5372, 318.85)(305.248, 4903.83)(306.2516, 1081.42)(300.2902, 5840.85)(301.2932, 1120.36)(302.3008, 250.71)(283.2641, 23714.24)(284.2669, 4218.66)(285.2765, 372.13)(582.5467, 1905.24)(583.5558, 996.6)(584.5574, 270.7)	193104.9	36839.71	180195.8	28102.86	225207.2	19960.02	231258.2	32788.77	0.60	0.014273	0.029303	0.53	0.065785	0.309933
COMPOUND_283_2879a10_857864	Rp	+	283.2879	10.86				(567.5745, 3570.57)(568.5716, 1719.39)(605.5587, 1491.19)(306.2779, 80197.7)(307.2803, 17428.75)(308.2835, 2331.24)(589.5563, 2291.85)(590.5748, 1527.63)(322.2497, 2432.92)(323.2497, 287.17)(284.2962, 203213.31)(285.2991, 39619.43)(286.301, 4641.87)(584.614, 2782.73)(585.609, 275.84)	85998.35	39804.15	107681.2	44622.33	212802.5	134678.3	178523.1	128597.6	1.42	8.06E-07	4.12E-06	-0.78	0.01404	0.179521
COMPOUND_283_2885a8_449652	Rp	+	283.2885	8.45				(284.2957, 21663.46)(285.299, 4141.85)(286.3024, 475.99)	64256.02	54266.08	43199.83	48345.68	50488.36	32042.07	62586.54	53008.75	-0.01	0.970706	0.983369	0.70	0.039287	0.261543
COMPOUND_284_2156a9_926902	Rp	+	284.2156	9.93				(307.2054, 777.82)(308.1951, 142.55)(309.2111, 1932.13)(323.1641, 242.05)(285.2223, 27562.32)(286.2288, 6847.53)(287.2274, 3221.76)(288.2209, 312.73)	144926.3	50050.12	152718.6	51732.33	169677.3	9763.875	152783.2	69066.04	-0.96	4.8E-05	0.00016	-0.18	0.487395	0.747693
COMPOUND_284_2349a10_180572	Rp	+	284.2349	10.18				(569.4548, 185.31)(607.4257, 4840.64)(302.267, 755.46)(285.2433, 10436.88)(286.2459, 2195.29)(287.2322, 1675.22)(288.2266, 158.93)	36171.1	13184.6	38600.82	16643.16	45595.78	15406.8	62790.02	32989.82	0.15	0.547952	0.646067	0.62	0.038469	0.259981
COMPOUND_284_251a8_235709	Rp	+	284.2510	8.24				(591.514, 85.39)(302.2696, 300.19)(285.2589, 17787.68)(286.2619, 3178.9)(287.2656, 643.62)	24289.96	9212.57	24212.92	9759.082	57567.07	19678.94	48495.84	22353.3	1.40	1.52E-08	1.19E-07	-0.15	0.551989	0.780002
COMPOUND_284_2515a8_853771	Rp	+	284.2515	8.85				(569.5082, 132.59)(285.259, 15904.64)(286.2622, 4074.5)(287.2657, 563.88)(288.2718, 46.63)	#DIV/0!	#DIV/0!	#DIV/0!	#DIV/0!	#DIV/0!	#DIV/0!	#DIV/0!	#DIV/0!	1.55	3.21E-11	6.39E-10	0.01	0.962737	0.981922
COMPOUND_284_2709a11_185121	Rp	-	284.2709	11.19	Stearic acid	1	10 eV: 283.2632(100)	(319.2401, 6441.77)(320.2438, 1500.33)(321.2253, 3119.46)(322.2248, 674.78)(323.2199, 419.66)(613.5219, 225.21)(283.2635, 1024397.5)(284.2675, 185898.58)(285.2703, 20044.0)(286.2735, 1841.71)(567.5338, 525.16)(568.5371, 282.08)(569.5459, 71.43)(329.2692, 13727.62)(330.2727, 2702.82)(331.2725, 216.08)	3440636	1492739	4229792	1531885	3300295	1115388	3037785	1640165	-0.06	0.819864	0.872719	-0.78	0.024411	0.219303
COMPOUND_284_2834a10_460715	Rp	+	284.2834	10.46				(569.5884, 212.06)(570.6038, 741.37)(307.2666, 1005.98)(591.5772, 341.1)(323.2609, 1086.97)(302.325, 755.6)(285.2919, 11029.5)(286.294, 2351.01)(287.3018, 563.34)(586.5878, 541.23)	38489.85	9882.296	38605.28	7831.822	41628.77	10998.76	40080.13	9298.171	0.07	0.785767	0.844249	0.05	0.87172	0.945212
COMPOUND_285_1378a8_28072	Rp	+	285.1378	8.28				(571.2818, 1712.07)(572.2862, 664.74)(573.2905, 236.46)(308.1276, 3842.56)(309.1292, 932.36)(310.1311, 83.22)(593.264, 1126.98)(594.2674, 513.12)(595.266, 94.61)(324.0893, 790.12)(286.145, 407326.3)(287.1485, 70386.09)(288.1514, 8704.53)(289.1533, 953.59)	471219	493905.6	508790.1	407741.6	433283.9	486602.2	238356.8	241053.8	-0.02	0.948055	0.966948	-0.79	0.022532	0.213113
COMPOUND_285_1946a4_9806747	Rp	+	285.1946	4.98	ACar 8:1	2	10 eV: 286.2037(100), 85.0288(40), 227.131(12), 125.0951(10), 55.0554(10)	(286.2022, 42941.66)(287.2058, 6716.69)(288.2071, 966.29)(289.2052, 99.52)	110844.1	55860.03	115731.6	52731.15	114492.9	29859.63	133431.2	54660.65	0.15	0.57166	0.668263	0.21	0.491695	0.748114
COMPOUND_285_195a0_9093032	Hilic	+	285.1950	0.91				(308.1862, 4675.11)(309.1897, 1031.25)(593.396, 356.33)(303.2251, 244.2)(286.2021, 66181.64)(287.2054, 11494.48)(288.2138, 2629.8)(289.2201, 289.66)(588.4375, 3263.6)(589.4406, 1046.1)	315227.8	159611.9	334997.7	129512.3	310107.4	93165.84	349069.8	123547.8	-0.01	0.96489	0.980123	0.15	0.613707	0.816343
COMPOUND_285_1953a5_092399	Rp	+	285.1953	5.09				(593.3834, 55.9)(286.2022, 11203.94)(287.2051, 1990.93)(288.2073, 229.47)	28260.08	11756.49	29218.29	15386.37	30460.5	23160.98	26753.5	14803.63	-0.12	0.674282	0.760763	-0.07	0.822082	0.922833
COMPOUND_285_2223a10_613533	Rp	+	285.2223	10.61				(593.441, 6672.21)(286.2304, 258041.5)(287.235, 71499.95)(288.2388, 10544.11)	149906.8	35595.69	113983.8	26188.2	497062.9	184808.1	424602	149449.2	1.60	8.48E-16	3.82E-13	-0.30	0.091687	0.359093
COMPOUND_286_2156a9_812983	Rp	+	286.2156	9.81	MAG 13:1	2	10 eV: 111.1163(100), 199.1695(35), 69.0693(10), 304.2394(1)	(611.4137, 668.63)(309.2054, 96129.01)(310.2101, 16229.86)(311.2152, 2648.57)(312.2194, 301.6)(595.4231, 10389.75)(596.4252, 3063.37)(325.1786, 3398.51)(326.1799, 677.99)(327.1776, 479.9)(328.1727, 36.25)(304.2493, 304911.03)(305.2525, 53063.19)(306.2549, 7277.05)(307.2557, 858.37)(287.2227, 198150.12)(288.2259, 33629.24)(289.2288, 4439.72)	1436713	318036	1569943	307009	1536870	352737.3	1434123	348395.2	0.44	0.131945	0.206061	-0.79	0.024906	0.219303
COMPOUND_286_2373a5_8505173	Hilic	+	286.2373	5.85	Unknown	4	20 eV: 100.0759 (100), 171.1502 (50), 269.2285 (20)	(287.2447, 33835.09)(288.248, 5802.66)(289.2509, 606.69)(144.1262, 2355.21)(144.6276, 409.2)(145.13, 235.41)	11542.09	3110.856	10750.57	4722.911	46531.86	20969.72	64140.72	29987.5	1.11	1.28E-08	1.03E-07	0.71	0.000671	0.04444
COMPOUND_287_2105a5_8078117	Rp	+	287.2105	5.81	Octanoylcarnitine	1	10eV: 288.2179(100), 85.0285(70), 60.0808(9), 127.1072(7)	(310.2027, 11664.4)(311.2055, 2280.0)(288.2181, 55420.46)(289.2215, 9434.36)(290.2236, 1317.2)(291.2223, 123.63)	129823.4	67635.8	134504.3	72098.91	83141.43	46994.23	122922.7	67540.92	-0.95	0.00078	0.002078	0.67	0.037876	0.259981
COMPOUND_287_2119a0_82994664	Hilic	+	287.2119	0.83				(310.2022, 78371.57)(311.2053, 14953.76)(312.2147, 5116.81)(305.2392, 187.16)(288.2178, 178350.81)(289.2209, 30152.17)(290.2236, 3958.52)(291.226, 407.85)	339342.8	171981	367946.6	193333.6	385711.2	173826.2	462993.7	180146	0.29	0.290711	0.390388	0.25	0.434803	0.712127
COMPOUND_288_04a1_2144005	Hilic	+	288.0400	1.21				(311.0305, 133.78)(289.0474, 27046.46)(290.0497, 3024.69)(291.0458, 12457.2)(292.0482, 1161.85)(293.0503, 102.33)	138500.9	9894.797	139249.8	12502.59	152734.4	8910.556	155652.1	13612.88	0.99	4.74E-05	0.000158	0.17	0.522258	0.765284
COMPOUND_289_1118a9_219045	Rp	+	289.1118	9.22				(328.0842, 168.4)(290.1186, 6407.95)(291.1218, 1134.53)(292.121, 303.05)	14294.58	2961.012	15417.69	3004.001	15618.77	1988.923	15275.12	2996.098	0.50	0.079326	0.132426	-0.67	0.045764	0.273966
COMPOUND_289_1531a4_1546154	Hilic	+	289.1531	4.15				(307.1875, 173.67)(290.1603, 11177.58)(291.1644, 1574.66)(292.1638, 311.53)	6049.943	1957.066	6541.821	2158.297	10644.42	5658.853	13894.81	7724.834	0.77	0.000416	0.001169	0.48	0.050509	0.283798
COMPOUND_290_191a8_975247	Rp	+	290.1910	8.98				(581.4065, 3190.06)(582.4112, 742.24)(308.2223, 700.66)(291.1962, 28964.38)(292.1995, 6451.57)(293.2049, 1348.49)	159515.3	19208.99	156815.4	14075.07	161273.8	20832.18	168879.3	13053	0.11	0.708662	0.787324	0.62	0.077521	0.333235
COMPOUND_294_1828a8_201307	Rp	-	294.1828	8.20				(623.3436, 456.74)(624.3426, 187.02)(407.1684, 2362.71)(408.1697, 639.49)(409.1623, 1110.93)(329.1528, 2040.59)(330.1549, 341.31)(331.1505, 794.04)(332.1564, 150.97)(293.1767, 9044.12)(294.1795, 1617.02)(295.1801, 459.8)(296.183, 48.19)	87703.49	32792.4	80161.61	38002.88	86288.43	20990.24	84019.67	31972.82	0.11	0.724253	0.798344	0.21	0.564606	0.785799
COMPOUND_294_2188a9_64962	Rp	-	294.2188	9.65				(329.1875, 1284.42)(330.1915, 259.34)(331.1981, 1720.87)(293.212, 71733.31)(294.2155, 13656.34)	76485.23	61489.45	101869.5	99689.38	183190.8	208024.8	68128.18	84841.53	-0.86	7.9E-05	0.000249	-0.52	0.03612	0.252742
COMPOUND_294_2189a9_60272	Rp	-	294.2189	9.60				(329.1892, 677.49)(330.1905, 104.97)(331.1998, 2172.88)(293.2122, 147995.31)(294.2156, 26620.56)	178905.6	86652.67	239840.8	141786.7	248198.8	191715.9	308602	170111.1	-0.82	0.00052	0.00144	-0.43	0.113973	0.400254

									1st trimester				3rd trimester									
									Control		Pre-eclampsia		Control		Pre-eclampsia		Time			Interaction		
Compound	Column	Ion_mode	Mass	Retention_	Identification	ID_level	MSMS_spectra	CompositeSpectrum	Mean	SD	Mean	SD	Mean	SD	Mean	SD	Value	raw p	FDR_p	Value	raw p	FDR_p
COMPOUND_294_2189a9_363246	Rp	-	294.2189	9.36				(329.1829, 264.4)(330.1856, 81.17)(331.1924, 826.81)(293.2116, 7027.77)(294.2153, 1445.98)(295.2078, 202.87)	29125.19	13891.22	39734.75	23142.32	37928.5	23916.97	45024.16	45622.66	-0.58	0.024898	0.04772	-0.38	0.217401	0.524815
COMPOUND_294_2194a9_665863	Rp	+	294.2194	9.67				(589.42, 1171.07)(590.4107, 402.01)(627.4108, 208.44)(317.2107, 38613.58)(318.2144, 8471.55)(613.4417, 1160.63)(614.4563, 367.32)(615.4236, 298.04)(312.2497, 605.52)(313.239, 817.74)(333.1786, 2408.92)(334.1823, 558.27)(295.228, 41658.87)(296.2299, 10299.57)(297.2397, 3515.94)(298.2427, 328.09)	180416.3	121446.2	242658.8	170503.1	136073.5	97986.6	325372	264807.5	-1.02	1.32E-05	5.15E-05	-0.26	0.309896	0.615962
COMPOUND_294_2201a9_608477	Rp	+	294.2201	9.61				(589.4665, 132.79)(590.4634, 39.12)(627.3984, 720.64)(317.2102, 41690.54)(318.2142, 8954.08)(611.4297, 1808.53)(612.4319, 846.93)(613.4466, 2264.96)(614.4477, 823.84)(615.4483, 762.17)(333.1657, 4551.2)(334.1674, 1053.74)(312.2541, 22110.41)(313.2562, 4924.68)(314.2635, 986.78)(295.2286, 230221.64)(296.2316, 45408.76)(297.2353, 7160.15)(298.239, 840.99)	317478.7	160793.1	437294	259861.9	492203.6	525447.5	461920	332631.7	-0.68	0.003767	0.008747	-0.58	0.035328	0.250187
COMPOUND_295_0591a6_145353	Hilic	+	295.0591	6.15				(613.1192, 231.38)(296.0663, 73142.34)(297.0699, 7300.86)(298.0657, 6310.11)(299.0687, 549.46)	107745.8	63583.65	80204.83	40729.69	88536.21	34847.74	147609.6	62964.82	-0.19	0.489146	0.589698	1.30	0.000171	0.018576
COMPOUND_295_1249a9_949218	Rp	+	295.1249	9.95				(591.26, 1973.23)(592.2587, 759.35)(318.1065, 181.02)(334.0973, 261.74)(296.1319, 13993.38)(297.1342, 2701.93)(298.1318, 6074.33)(299.135, 1316.0)(300.1279, 2684.84)	34435.88	9040.053	37935.44	10552.67	36391.07	8525.546	40401.26	11538.17	0.20	0.480134	0.582891	0.06	0.853745	0.937825
COMPOUND_295_2162a9_045663	Rp	+	295.2162	9.05				(591.4234, 1530.37)(296.2232, 65593.04)(297.2275, 12653.97)(298.2308, 1849.63)	304401	17437.3	292451.6	20559.16	320060	21152.69	323942.1	19600.04	0.68	0.00862	0.018594	0.69	0.024515	0.219303
COMPOUND_296_1958a10_465157	Rp	-	296.1958	10.47				(627.3484, 513.95)(637.367, 701.62)(295.189, 10510.62)(296.1923, 1653.15)(297.1941, 331.57)(705.3638, 2124.82)	20059.4	5715.129	25147.07	7814.097	18400.93	5911.309	20974.06	6911.772	-0.17	0.550283	0.647797	-0.42	0.204697	0.516497
COMPOUND_296_2347a9_623438	Rp	-	296.2347	9.62	9-HODE	2	10 eV: 295.2276(100), 171.1023(15), 226.9689(3), 42.3797(2)	(331.2033, 8581.69)(332.2074, 1663.72)(333.2041, 3923.0)(334.2066, 674.36)(295.2276, 184656.31)(296.2308, 36121.0)(297.234, 4973.55)(298.2349, 561.62)(341.2294, 411.13)	252869.2	123282.4	381278.2	233952.7	155666.5	237139.1	168937.2	295785.2	-0.75	0.003359	0.007905	-0.56	0.06042	0.302235
COMPOUND_297_1077a1_8546703	Hilic	+	297.1077	1.85				(320.0969, 3571.81)(321.1002, 545.77)(322.1125, 88.22)(315.1366, 292.93)(298.1149, 1412.08)(299.1159, 336.31)(300.1248, 44.5)	18944.08	3460.183	20030.63	2758.775	23309.14	3344.942	24975.32	4059.803	1.00	4.34E-05	0.000146	0.09	0.751095	0.893065
COMPOUND_298_1332a11_366976	Rp	+	298.1332	11.37				(597.2823, 4418.58)(321.1207, 888.63)(322.1223, 253.72)(337.0881, 286.62)(299.1397, 28132.09)(300.1431, 6066.03)(301.143, 1050.85)	39803.34	29586.78	32203	19135.25	54946.79	40619.56	39342.4	27894.47	0.57	0.033534	0.062104	-0.07	0.825382	0.923744
COMPOUND_298_1938a8_965505	Rp	+	298.1938	8.97				(299.2019, 114866.73)(300.2051, 25114.96)	105792.9	97895.34	90602.69	44341.4	74404.5	43603.65	69041.1	33260.11	-1.19	7.9E-08	5.08E-07	-0.08	0.741725	0.888965
COMPOUND_298_2501a10_544218	Rp	-	298.2501	10.54				(333.2122, 322.04)(297.243, 7799.13)(298.2467, 1776.8)(709.467, 624.5)	19063.49	4802.049	18678.82	4105.84	17472.92	3498.514	18518.41	4461.42	0.14	0.625671	0.717054	-0.33	0.319997	0.626638
COMPOUND_298_2502a10_0630665	Rp	-	298.2502	10.06				(411.2202, 139.78)(333.2181, 544.57)(334.2208, 199.13)(335.2216, 290.5)(297.2431, 21109.53)(298.2465, 4395.11)	92538.79	46738.52	91528.48	44377.42	100006.7	38908.51	86757.65	32211.48	0.24	0.430239	0.535303	-0.31	0.398003	0.690408
COMPOUND_298_2503a10_080804	Rp	-	298.2503	10.08				(411.2223, 157.64)(333.2186, 508.1)(334.2209, 36.75)(335.2269, 456.28)(297.2434, 22417.84)(298.247, 4757.11)	85221.05	43220.54	78823.8	34912.59	97565.83	28940.71	84969.76	41603.08	0.41	0.154811	0.234297	-0.11	0.749216	0.891783
COMPOUND_298_2503a10_408115	Rp	-	298.2503	10.41				(411.2159, 173.94)(333.2189, 439.27)(334.2217, 58.95)(297.243, 15900.74)(298.2465, 3425.91)	31380.43	11888.73	33885.87	14518.21	31348.43	6392.347	31992.39	14706.37	0.01	0.976507	0.987116	-0.13	0.718474	0.876415
COMPOUND_298_2506a9_992452	Rp	-	298.2506	9.99				(333.2184, 439.96)(334.223, 94.46)(335.2238, 492.09)(297.2432, 21913.41)(298.2468, 4945.98)	95203.02	50776.03	85217.26	48605.31	92108.71	31335.95	92664.81	42492.02	0.33	0.269942	0.367762	0.16	0.648306	0.838541
COMPOUND_298_2506a10_0638	Rp	-	298.2506	10.06				(333.2187, 420.57)(334.2225, 102.56)(335.2277, 393.77)(297.243, 17054.93)(298.2465, 3582.67)	98375.94	45248.95	107176.6	44362.99	100658.2	29741.27	97021.89	33874.92	0.20	0.505128	0.605401	-0.24	0.511015	0.757607
COMPOUND_298_2513a10_031149	Rp	+	298.2513	10.03				(321.2423, 26508.17)(322.2458, 5437.27)(323.2538, 2712.1)(619.478, 160.1)(337.2209, 1441.29)(338.2277, 428.23)(339.2228, 379.4)(316.286, 5539.53)(317.2882, 1111.67)(318.2695, 2532.39)(299.2588, 48532.73)(300.2621, 9784.09)(301.2641, 1735.07)(614.5651, 120.13)	336030	142219.9	290182.2	122332.8	282246	98681.68	278867.9	119489.3	-0.32	0.254482	0.350945	0.29	0.387193	0.68827
COMPOUND_298_2513a10_417089	Rp	+	298.2513	10.42				(321.2415, 1896.74)(322.2435, 487.82)(323.2374, 700.6)(324.2438, 154.79)(337.2228, 342.09)(316.2849, 1622.56)(317.2918, 65.69)(299.2587, 23506.19)(300.2626, 5144.49)(301.2653, 866.37)	41030.19	13388.36	40157	16702.32	55035.57	7397.68	57631.93	18795.38	0.07	0.773524	0.833547	0.62	0.048385	0.279822
COMPOUND_298_2515a10_02615	Rp	+	298.2515	10.03				(321.2421, 18010.96)(322.2455, 3628.69)(323.2512, 2297.41)(619.4819, 129.23)(337.2113, 1726.07)(338.2198, 568.77)(339.215, 350.91)(316.2857, 6678.19)(317.2891, 1518.62)(318.2963, 1667.92)(299.2589, 55097.09)(300.2626, 11180.39)(301.2655, 1670.21)(614.5582, 103.81)	321097.9	130784.9	290928.6	105360.6	232320.6	90860.95	280030.2	116411.5	-0.82	0.004579	0.010385	0.86	0.011274	0.166978
COMPOUND_298_2515a10_214904	Rp	+	298.2515	10.21				(321.2423, 2705.5)(322.2418, 592.84)(323.2515, 878.85)(324.2562, 146.32)(337.2054, 159.04)(316.2858, 7097.62)(317.2879, 1577.2)(318.2975, 863.71)(299.2589, 18013.81)(300.2625, 3686.05)(301.2666, 642.11)	74443.05	15939.61	66861.9	14901.54	85345.36	25738.16	85413.63	27245.93	0.67	0.01434	0.029412	-0.43	0.178881	0.493677
COMPOUND_298_2517a10_217733	Rp	+	298.2517	10.22				(321.2412, 2215.86)(322.2439, 670.21)(323.2494, 1151.13)(324.252, 211.25)(337.2161, 350.65)(338.2073, 109.0)(316.2855, 6882.04)(317.2891, 1360.73)(318.2973, 856.84)(299.2585, 22961.75)(300.2623, 4379.28)(301.262, 753.01)	71749.63	20303.69	69236.24	15142.56	83036.08	30971.92	91737.65	33910.64	0.15	0.5769	0.673499	0.48	0.135104	0.435893
COMPOUND_298_2519a10_314493	Rp	+	298.2519	10.31				(321.2423, 2687.59)(322.2476, 397.26)(323.2536, 944.54)(324.2559, 219.87)(337.2075, 150.12)(338.2135, 49.82)(316.2841, 3019.11)(317.2883, 674.19)(299.2584, 23506.22)(300.262, 5372.82)(301.2645, 829.33)	73850.87	17039.24	69134.48	14942.28	112299.6	16251.75	103084.2	21719.7	1.50	1.81E-09	1.85E-08	-0.12	0.633123	0.828355
COMPOUND_298_2522a10_054714	Rp	+	298.2522	10.05				(321.2415, 11385.57)(322.2452, 2173.46)(323.2517, 847.74)(619.4738, 126.15)(337.2197, 1235.35)(338.2289, 329.74)(339.2264, 395.67)(316.2857, 13176.39)(317.2883, 2708.11)(318.297, 2188.95)(299.2589, 42885.49)(300.2624, 8795.28)(301.2648, 1465.71)(614.5687, 106.32)	302903.4	153821	293717.5	148722.1	260159.6	140098.4	306976.3	180186.7	-0.33	0.280871	0.38012	0.39	0.278153	0.585315
COMPOUND_298_286a11_37146	Rp	-	298.2860	11.37				(411.2601, 1170.0)(297.2796, 13446.6)(298.2832, 3114.99)(299.2848, 480.55)(343.2844, 233.69)	15284.71	5092.552	16835.23	4767.27	19510.92	6188.541	18867.78	7615.956	0.79	0.003627	0.008465	-0.71	0.024004	0.219303
COMPOUND_299_2811a10_44985	Rp	+	299.2811	10.45				(322.2719, 3440.48)(323.2763, 1259.44)(621.5594, 266.6)(300.2914, 21752.65)(301.2949, 4320.17)(302.2951, 627.45)	26260.04	8750.21	28415.49	12886.82	31047.79	12399.63	33031.11	10854.28	0.51	0.060425	0.104274	-0.13	0.677337	0.851683
COMPOUND_299_2834a8_822751	Rp	+	299.2834	8.82				(300.2905, 20197.8)(301.2933, 4544.01)(302.2963, 636.54)(303.3035, 72.33)	34724.49	24482.44	35368.19	18277.23	43539	25516.95	65379.7	50093.03	0.42	0.115492	0.184853	0.47	0.144657	0.451201
COMPOUND_300_2097a9_011984	Rp	+	300.2097	9.01				(323.2018, 6437.12)(324.2074, 1884.33)(623.4093, 576.41)(624.4188, 192.58)(625.421, 525.39)(301.2171, 37552.09)(302.2205, 8310.45)(303.227, 1784.23)(304.2272, 199.09)	269563.5	146263.2	280868.3	141898.6	359042.5	220751.5	253872.7	176117.8	-0.95	3.01E-05	0.000107	-0.32	0.210454	0.519294
COMPOUND_300_2097a9_649985	Rp	+	300.2097	9.65				(601.4198, 660.4)(602.427, 3211.22)(323.199, 10020.75)(324.2027, 2561.86)(339.1703, 1082.99)(340.1696, 357.18)(341.1547, 107.51)(318.242, 5670.63)(301.2178, 375473.66)(302.2211, 73935.51)(303.2303, 22280.84)	255296.6	225640.4	247278	166473.9	317489	69819.04	267402.6	117141.4	-0.92	4.5E-05	0.000151	-0.25	0.314333	0.620945
COMPOUND_300_2099a9_305988	Rp	+	300.2099	9.31				(323.2024, 1576.17)(324.212, 670.26)(623.4247, 477.72)(301.2175, 437941.94)(302.2209, 88838.86)(303.2253, 11999.76)(304.2325, 1421.78)	1001118	385324.6	851999.6	342940.5	515043.5	291357.3	300522.7	286547.6	-1.55	1.44E-12	5.91E-11	0.24	0.246047	0.555348
COMPOUND_300_21a9_024943	Rp	+	300.2100	9.02				(323.2018, 5591.31)(324.2074, 1546.69)(623.4153, 428.72)(301.2173, 87786.15)(302.2203, 18931.8)(303.2274, 3084.4)(304.2227, 289.36)	296688.9	128422.3	316293.8	155191	360308.8	230777.3	253095.1	140259.6	-0.94	2.31E-05	8.45E-05	-0.40	0.105055	0.385317
COMPOUND_300_2462a8_750348	Rp	+	300.2462	8.75				(323.235, 1949.52)(324.2401, 333.29)(325.246, 821.67)(623.4793, 362.57)(624.4852, 147.33)(625.4872, 38.02)(339.199, 329.39)(340.2104, 120.2)(341.2105, 120.19)(318.279, 447.32)(301.2534, 18007.09)(302.2568, 3916.45)(303.2582, 478.05)	11420	#DIV/0!	14586	#DIV/0!	49098.69	30777.5	47476.94	44708.4	1.43	4.98E-15	1.12E-12	-0.14	0.386079	0.687856
COMPOUND_300_2465a8_813984	Rp	+	300.2465	8.81				(323.235, 3350.6)(324.2377, 1026.75)(325.2364, 269.15)(623.4795, 750.36)(624.4843, 413.94)(625.4843, 93.31)(339.2127, 615.08)(340.2036, 262.93)(341.2085, 283.38)(318.2815, 965.78)(319.2734, 259.79)(301.2536, 17044.53)(302.257, 4410.68)(303.2658, 1264.38)	#DIV/0!	#DIV/0!	#DIV/0!	#DIV/0!	84039.5	55203.1	57606.55	47440.43	1.47	1.6E-13	1.25E-11	-0.38	0.042589	0.268638
COMPOUND_300_2465a8_023449	Rp	+	300.2465	8.02				(301.254, 17670.78)(302.2554, 4211.59)(303.259, 568.25)	33366.37	14594.58	38079.32	20962.88	19292.85	5186.274	20527.12	7005.148	-0.75	0.002873	0.006875	-0.17	0.560898	0.783425
COMPOUND_300_265a10_052652	Rp	-	300.2650	10.05				(335.2237, 370.5)(299.2585, 8476.62)(300.2618, 1615.11)(301.264, 213.83)	40597.14	17208.28	43781.87	15966.42	42410.85	18720.06	39163.75	18486.63	0.05	0.867465	0.910313	-0.23	0.530842	0.771657

									1st trimester				3rd trimester									
									Control		Pre-eclampsia		Control		Pre-eclampsia		Time			Interaction		
Compound	Column	Ion_mode	Mass	Retention_	Identification	ID_level	MSMS_spectra	CompositeSpectrum	Mean	SD	Mean	SD	Mean	SD	Mean	SD	Value	raw p	FDR_p	Value	raw p	FDR_p
COMPOUND_300_2653a10_016575	Rp	-	300.2653	10.02				(335.2258, 463.49)(336.222, 70.0)(299.2585, 9229.74)(300.2621, 1862.54)(301.2663, 121.93)	46720.53	7512.53	47459.93	6727.716	49599.5	10478.07	50936.11	11289.55	0.34	0.230509	0.325662	-0.03	0.918208	0.962217
COMPOUND_300_2658a10_777741	Rp	-	300.2658	10.78				(413.2471, 319.68)(335.22, 1424.97)(299.2587, 12930.3)(300.2628, 2331.15)(301.2662, 321.17)	28472.88	9862.398	30997.22	9361.214	20622.07	5906.423	22622.02	5507.416	-0.82	0.003966	0.00917	-0.04	0.901603	0.955487
COMPOUND_300_266a10_737138	Rp	-	300.2660	10.74				(335.2245, 499.67)(413.2444, 282.69)(299.2587, 12981.21)(300.2622, 2615.14)(301.2663, 546.54)	23106.94	9298.916	21551.78	9582.629	19328.86	6385.69	16869.81	4417.715	-0.24	0.398019	0.501869	-0.31	0.374287	0.677023
COMPOUND_301_0754a9_211498	Rp	-	301.0754	9.21				(414.0469, 161.79)(300.0665, 4906.87)(301.0678, 969.53)(302.0673, 234.12)(601.1602, 871.25)(602.156, 552.24)(715.1528, 620.48)(716.1366, 385.08)	17171.35	4785.724	17375.12	4913.292	17333.79	3224.751	17161.4	4397.404	0.33	0.274953	0.37391	-0.29	0.411174	0.694731
COMPOUND_301_1895a1_4800488	Hilic	+	301.1895	1.48				(319.226, 358.91)(302.197, 32908.91)(303.2006, 5333.69)(304.2087, 1386.27)(305.2113, 134.29)	10507.58	8066.564	11537.29	7184.235	90132.64	86269.97	74224.75	102865.4	1.53	2.51E-10	3.43E-09	-0.16	0.497755	0.752946
COMPOUND_301_1896a1_1891912	Hilic	+	301.1896	1.19				(603.394, 193.71)(302.1964, 32380.04)(303.1996, 5512.14)(304.2019, 957.74)(305.2052, 105.9)	9666.022	6510.729	10607.38	8623.651	48679.21	35820.56	42752.39	48830.18	1.59	7.03E-10	8.21E-09	-0.39	0.133839	0.434929
COMPOUND_301_2095a8_116018	Rp	+	301.2095	8.12				(302.2161, 33821.04)(303.2193, 6370.2)(304.2159, 2184.49)	51398.34	23249.6	53920.2	22327.59	60613.29	36547.65	74267.96	30891.9	0.28	0.285599	0.384886	0.38	0.223404	0.531262
COMPOUND_301_2243a0_7576121	Hilic	+	301.2243	0.76				(603.4524, 244.01)(324.218, 396.32)(625.3986, 688.69)(302.2314, 4953.26)(303.2358, 1201.73)(304.2313, 1689.24)(305.2355, 230.99)	78136.27	35618.59	83101.07	53745.09	64733.73	30618.74	93882.34	52640.29	-0.44	0.039295	0.071135	0.06	0.804088	0.917422
COMPOUND_301_2263a6_172385	Rp	+	301.2263	6.17	ACar 9:0	2	10 eV: 302.2299(100), 60.0787(50), 243.1611(50)	(641.4048, 99.94)(302.2335, 8830.36)(303.2367, 1424.59)(304.2296, 570.49)(305.2402, 48.6)	35284	18898.93	34447.47	22800.87	27814.62	12320.84	33745.32	20504.91	-0.22	0.331741	0.43284	-0.09	0.721809	0.877707
COMPOUND_302_2149a10_7173195	Rp	+	302.2149	10.72				(605.4288, 4122.97)(606.4326, 2397.37)(607.433, 2564.14)(643.3741, 609.16)(303.2213, 10436.01)(304.2253, 3334.6)(305.2176, 1552.05)(306.2138, 165.77)	47172	23105.7	45342.89	17627.2	49255.73	16822.28	56515.33	32255.78	0.63	0.016812	0.033834	-0.21	0.502969	0.754119
COMPOUND_302_2238a10_482109	Rp	-	302.2238	10.48				(337.1881, 4480.89)(338.1942, 994.07)(301.2172, 308249.2)(302.2205, 64439.22)(303.2235, 7671.46)(304.2262, 722.07)(347.2055, 586.88)(717.4192, 1150.96)(718.4268, 1726.53)	234529.5	126446.8	288820.6	212010.5	143504.1	101115.3	159071.1	105620.8	-0.95	7.24E-05	0.00023	0.03	0.896249	0.954308
COMPOUND_302_2242a9_920079	Rp	-	302.2242	9.92				(301.2167, 23776.09)(302.22, 5315.76)(303.2273, 977.96)(304.2283, 115.98)	51702.28	22511.67	56231.57	22815.6	45729.25	22831.01	61351	19387.57	-1.00	1.49E-05	5.75E-05	-0.27	0.285137	0.593016
COMPOUND_302_2265a9_926412	Rp	+	302.2265	9.93				(605.4299, 2312.06)(606.4519, 651.87)(607.4187, 1376.29)(608.4274, 968.26)(325.2142, 16271.79)(326.2178, 4277.21)(627.4378, 3820.83)(628.4264, 625.01)(629.4319, 1235.03)(320.2591, 333.71)(341.1945, 2491.54)(342.1853, 642.6)(303.2328, 799857.44)(304.2371, 160367.83)(305.2456, 41993.32)	1527532	604844.5	1608513	657697	1551175	823001.4	1538975	877524.9	-1.00	1.87E-05	7.06E-05	-0.23	0.369536	0.671661
COMPOUND_302_2455a10_181607	Rp	+	302.2455	10.18				(643.4433, 3080.63)(644.4504, 1966.68)(645.4515, 2046.95)(325.2359, 14558.6)(326.2403, 2736.97)(327.2373, 1309.85)(328.2277, 98.86)(320.2803, 27284.57)(321.2829, 5549.4)(322.2845, 1108.03)(341.208, 2421.62)(342.2195, 472.88)(343.2133, 1780.04)(344.2223, 631.26)(303.2542, 69987.6)(304.2567, 13633.22)(305.2493, 21330.05)(306.2518, 4520.6)	104262.5	54884.37	122670	70582.07	122026.2	66649.37	232681.3	154848.3	0.31	0.235478	0.329992	0.63	0.041652	0.265944
COMPOUND_303_2047a4_3564334	Rp	+	303.2047	4.36				(304.2133, 16046.99)(305.2161, 3218.25)(306.221, 364.86)	25641.98	11254.91	26422.1	13972.91	22016.5	10960.49	22784.48	10379.37	-0.68	0.012136	0.02519	0.53	0.092922	0.361416
COMPOUND_303_2047a1_400974	Hilic	+	303.2047	1.40				(607.4056, 118.77)(326.2024, 288.79)(327.1932, 95.52)(328.2001, 1259.54)(329.2064, 293.32)(629.408, 122.64)(304.2126, 64195.16)(305.2161, 10908.52)(306.2176, 2056.38)(307.2205, 238.79)(624.416, 130.08)	74771.26	37446.02	79485.54	50824.84	58048.36	31849.75	72216.89	35347.75	-0.65	0.022029	0.042878	0.57	0.083064	0.345768
COMPOUND_304_2404a10_686863	Rp	-	304.2404	10.69				(643.442, 146.61)(644.4632, 73.41)(645.4362, 301.34)(339.2087, 5722.79)(340.2126, 1313.31)(341.2074, 2110.0)(342.2099, 660.22)(343.1981, 67.0)(303.2323, 895668.94)(304.2363, 176600.42)(305.2392, 21101.61)(306.2422, 1689.53)(349.2398, 1313.2)	1151397	320914.4	1387655	520570.1	929458.4	401620.1	991457.4	418179.3	-0.66	0.00767	0.016689	-0.21	0.451618	0.723875
COMPOUND_304_2409a9_84599	Rp	+	304.2409	9.85				(327.2332, 94353.87)(328.2368, 22543.75)(322.2747, 90.09)(305.249, 44715.91)(306.2522, 9165.92)(307.2585, 1884.37)(308.2508, 199.66)	186207.6	85042.96	213703.8	128493.6	217213.6	79399.85	189883.8	98112.94	-0.69	0.01389	0.028618	0.05	0.876224	0.948226
COMPOUND_304_2414a10_6913805	Rp	+	304.2414	10.69				(609.4705, 508.08)(610.4696, 274.02)(647.4316, 4916.36)(648.426, 9276.05)(327.2254, 8334.75)(328.2276, 2377.4)(329.2163, 160.53)(322.2746, 38234.28)(323.2776, 10910.18)(324.2818, 1638.64)(305.2487, 55528.38)(306.2523, 11958.46)(307.255, 1783.95)	175741	38990.68	192850.8	67377.02	154431.9	73752.42	147742.6	59613.55	-0.27	0.299675	0.400041	-0.28	0.348674	0.65489
COMPOUND_304_2421a9_799166	Rp	+	304.2421	9.80				(609.515, 125.07)(327.2336, 128710.77)(328.2366, 31097.42)(322.2888, 446.96)(305.2527, 31098.41)(306.2546, 4180.3)(307.2543, 446.5)	240005.5	78103.32	261694.5	144432.8	247791.8	28103.73	223710.1	156709	-1.09	2.08E-05	7.7E-05	0.09	0.73781	0.888668
COMPOUND_306_2552a10_840969	Rp	-	306.2552	10.84	FA 20:3	2	10 eV: 305.2491(100), 88.5665(1)	(341.2244, 1166.55)(342.2268, 326.15)(343.2238, 848.42)(344.2274, 243.98)(657.5035, 1020.12)(658.5071, 328.14)(305.2485, 115133.92)(306.2517, 26220.29)(307.2546, 3396.8)(308.2566, 364.41)(611.4864, 1972.96)(725.4824, 2655.55)(726.4705, 1652.19)(727.4809, 1513.2)	173843.4	68879.97	213644.7	106812.9	125830.3	43849.76	169883.6	70977.18	-0.85	0.00139	0.003524	0.37	0.21743	0.524815
COMPOUND_308_2702a11_02445	Rp	-	308.2702	11.02				(651.5141, 153.44)(421.2449, 1073.37)(422.2456, 344.73)(343.2367, 396.56)(661.533, 763.45)(662.5358, 381.06)(307.2638, 27679.15)(308.2673, 5773.44)(309.2703, 752.59)(310.2775, 85.59)	27875.69	15124.26	37860.41	26251.42	34469.07	15926.41	50161.44	26433.51	0.53	0.041252	0.074142	0.07	0.80705	0.917894
COMPOUND_309_1952a5_795485	Rp	+	309.1952	5.80				(310.2026, 25178.2)(311.2056, 4910.04)(312.2065, 714.13)	31382.25	14776.53	33460.27	14119.08	49530.64	18609.53	53427.93	24237.3	0.94	0.000921	0.002423	-0.08	0.80289	0.916921
COMPOUND_309_1952a8_7701235	Rp	+	309.1952	8.77				(310.2022, 8801.47)(311.2039, 2011.08)(312.2039, 664.42)	33648.44	3014.259	32794.61	3046.738	33413.64	2837.681	33685.91	3076.03	-0.05	0.874192	0.874192	0.29	0.428185	0.522902
COMPOUND_309_1953a5_7616744	Rp	+	309.1953	5.76				(310.2026, 25178.2)(311.2056, 4910.04)(312.2065, 714.13)	36509.04	15069.72	37215.33	14553.46	53733.31	18921.37	53888.09	23931.06	0.76	0.007196	0.01578	0.05	0.869108	0.945066
COMPOUND_309_1956a5_458934	Rp	+	309.1956	5.46				(310.2022, 6419.38)(311.2063, 1229.53)(312.2118, 619.54)	17383.04	6799.966	15058.14	4597.563	18030.29	5830.675	21316.7	8588.062	0.83	0.000823	0.002181	0.14	0.62463	0.823156
COMPOUND_309_232a9_70118	Rp	+	309.2320	9.70				(332.2244, 325.27)(310.2386, 52590.45)(311.2422, 10502.6)(312.2476, 1756.43)	172594.2	16422.75	172351.2	12219.02	164072.4	13837.94	171745.3	15289.71	-0.52	0.082866	0.137519	0.40	0.258793	0.570391
COMPOUND_309_3044a10_923578	Rp	+	309.3044	10.92				(619.6188, 15729.51)(620.633, 1130.92)(621.6004, 19031.22)(657.5949, 3001.48)(332.2938, 13425.47)(333.2941, 3354.42)(334.3035, 784.93)(335.3104, 94.73)(348.265, 3727.91)(349.2693, 1427.2)(350.2701, 528.75)(310.3113, 106478.91)(311.3143, 25399.17)(312.3194, 4297.0)(313.3204, 528.97)	236833.8	84850.9	256742.9	97034.51	418875.1	214655.9	376515.5	186107.8	1.16	2.96E-05	0.000105	-0.40	0.193678	0.505089
COMPOUND_310_1608a10_859484	Rp	+	310.1608	10.86				(621.3033, 1539.92)(622.3011, 562.46)(623.3112, 1386.7)(659.3096, 4065.11)(660.2861, 2819.45)(643.2917, 1483.66)(311.168, 21192.77)(312.171, 4183.96)(313.1683, 11957.45)(314.1718, 2360.0)(315.1642, 4549.44)(316.167, 1184.59)(317.1636, 970.27)	92492.19	24648.96	93848.77	24504.42	143636.7	37709.68	143065.8	31435.42	1.25	1.66E-06	7.97E-06	0.05	0.847077	0.934191
COMPOUND_310_1774a8_144192	Rp	-	310.1774	8.14				(345.1471, 542.31)(346.1465, 71.26)(347.1439, 150.58)(665.3593, 130.69)(666.3511, 75.56)(309.1703, 10155.27)(310.1741, 1851.15)(311.1721, 649.87)(312.1776, 66.75)	54137.17	7364.808	54849.7	4750.575	55952.86	5476.129	54530.75	6455.589	0.25	0.416482	0.521355	-0.23	0.519483	0.764125
COMPOUND_310_1786a8_1496525	Rp	+	310.1786	8.15		4	10eV: 237.1136(100), 255.1226(60), 293.178(35), 209.1185(22), 57.0699(15)	(333.1679, 2989.32)(334.1711, 658.56)(335.173, 164.03)(349.1384, 655.59)(350.135, 154.38)(351.1419, 235.96)(311.1863, 34763.54)(312.1897, 6635.11)(313.1884, 1506.14)(314.1873, 162.86)	198899.7	12443.4	195701.9	13858.35	211780.9	11112.8	202961.1	11285.56	1.10	0.000118	0.000359	-0.53	0.097031	0.367252
COMPOUND_310_2519a10_315594	Rp	+	310.2519	10.32				(659.4696, 5887.23)(333.246, 517.27)(334.2516, 188.51)(335.2546, 271.84)(349.2155, 28913.74)(350.2188, 7185.95)(328.2842, 656.68)(311.2552, 1496.82)(312.2575, 413.81)(313.2385, 6744.33)	67209.47	23027.05	65908.61	26016.55	68022.46	21286.95	97569.65	65313.81	-0.15	0.600893	0.694311	0.76	0.025079	0.219303
COMPOUND_310_2862a11_246996	Rp	-	310.2862	11.25				(423.2714, 6611.27)(424.273, 1677.72)(425.2616, 724.32)(345.2565, 548.73)(309.2794, 55482.91)(310.2829, 12439.38)(355.2846, 48601.75)(356.2882, 10333.07)	49807.27	55852.25	42264.12	35796.6	67823.93	47654.46	72819.65	47440.01	0.75	0.004751	0.010742	0.15	0.614446	0.816844
COMPOUND_311_1234a1_5998819	Hilic	+	311.1234	1.60				(623.2541, 440.03)(334.1134, 3457.72)(335.1143, 128.82)(645.2358, 276.49)(312.132, 3270.41)(313.135, 486.86)(314.1356, 205.23)	18164.84	3915.592	16674.83	3386.698	22849.5	5475.456	26069.38	4882.165	0.46	0.036292	0.066394	0.56	0.032758	0.244873
COMPOUND_311_21a0_7971924	Hilic	+	311.2100	0.80				(334.2112, 643.84)(329.2521, 4206.51)(312.2176, 99409.11)(313.2209, 17600.68)(314.2232, 3150.05)(315.2247, 260.67)(640.4329, 271.34)	75171.94	46815.97	75535.19	47338.1	79264.73	21741.11	75223.59	64122.78	-0.08	0.792366	0.849314	0.02	0.958453	0.980661
COMPOUND_311_3204a11_173452	Rp	+	311.3204	11.17				(661.6222, 2036.92)(334.3095, 13609.21)(335.3113, 2858.9)(336.3214, 1052.31)(645.6344, 9988.13)(646.6198, 3923.77)(350.2805, 1623.78)(329.353, 3098.05)(330.3547, 592.03)(312.3269, 62798.49)(313.3301, 13517.3)(314.3327, 1508.89)	39391.5	24758.11	39992.07	33944.4	126403	69425.48	120944.3	47970.45	1.30	6.75E-08	4.47E-07	-0.05	0.838657	0.930372

									1st trimester				3rd trimester									
									Control		Pre-eclampsia		Control		Pre-eclampsia		Time			Interaction		
Compound	Column	Ion_mode	Mass	Retention_	Identification	ID_level	MSMS_spectra	CompositeSpectrum	Mean	SD	Mean	SD	Mean	SD	Mean	SD	Value	raw p	FDR_p	Value	raw p	FDR_p
COMPOUND_312_1376a9_385394	Rp	+	312.1376	9.39				(335.1257, 2514.72)(336.13, 589.73)(337.1326, 144.33)(351.0956, 643.44)(313.1441, 21264.02)(314.1475, 4388.67)(315.1494, 736.76)	18311.57	8979.078	21230.24	15133.18	24888.58	10720.18	20803.82	16293.51	0.42	0.144655	0.221608	-0.74	0.034807	0.249266
COMPOUND_312_1471a4_4788327	Rp	-	312.1471	4.48				(425.1289, 3350.27)(426.1302, 751.56)(347.1164, 7311.91)(348.1192, 1433.04)(349.1138, 2487.95)(350.1177, 548.67)(351.1202, 111.78)(311.14, 206103.8)(312.1432, 40379.48)(313.1462, 5059.0)(314.1484, 510.66)(623.2869, 25252.98)(624.2899, 10428.23)(625.2931, 2179.95)(626.2932, 448.67)	439659.6	169693.1	477717.7	206422.2	479290.9	186283.8	487591.7	222195.6	0.08	0.765971	0.831309	-0.12	0.708297	0.868783
COMPOUND_312_1482a4_495519	Rp	+	312.1482	4.50				(625.3042, 2364.66)(626.3043, 1085.69)(627.3064, 316.35)(628.3106, 65.38)(663.2586, 176.26)(664.2524, 82.62)(335.1346, 727.73)(336.1389, 247.76)(337.1223, 43.83)(647.2801, 61.57)(648.2765, 31.76)(649.2611, 67.24)(650.2743, 31.5)(330.181, 222.65)(351.1038, 1162.74)(313.1554, 951894.6)(314.159, 180538.81)(315.1617, 21652.04)(316.1643, 2471.78)	#DIV/0!	#DIV/0!	#DIV/0!	#DIV/0!	#DIV/0!	#DIV/0!	#DIV/0!	#DIV/0!	0.10	0.736163	0.808307	-0.01	0.967305	0.982576
COMPOUND_312_1483a1_6320281	Hilic	+	312.1483	1.63				(625.3026, 66313.67)(626.3056, 27334.73)(627.3081, 5858.52)(628.3109, 1065.64)(629.3176, 202.13)(335.137, 31933.41)(336.1409, 7429.08)(337.1435, 1761.03)(338.1584, 211.3)(647.2844, 4404.27)(648.2878, 1703.59)(649.2896, 843.34)(650.2894, 179.09)(651.288, 23.99)(313.1551, 680675.94)(314.1588, 128304.59)(315.1613, 16688.75)(316.1636, 1465.31)(642.3341, 268.7)(643.3398, 80.54)	1249131	525330.3	1357152	660578.8	1317223	563553.1	1398477	741556	-0.02	0.934409	0.95693	-0.02	0.939927	0.972308
COMPOUND_312_2295a9_604098	Rp	-	312.2295	9.60				(347.1992, 5286.9)(348.2017, 1143.67)(349.1965, 1617.63)(350.2009, 398.47)(351.208, 202.29)(311.2223, 46507.95)(312.2256, 8637.88)(313.2296, 1651.18)(314.233, 191.09)(357.2176, 268.93)	90553.15	44813.06	115815.4	64122.6	107925.3	89164.77	138279.1	75889.07	-0.89	0.000148	0.000447	-0.37	0.161943	0.475484
COMPOUND_312_2312a10_211772	Rp	+	312.2312	10.21				(335.2208, 2104.9)(336.2239, 547.43)(337.2315, 645.03)(330.272, 5487.44)(313.2385, 16278.71)(314.2419, 3119.21)(315.2489, 1063.79)	83715.98	8415.888	79787.24	9428.159	101704.1	14391.46	105687.8	14608.5	1.01	2.27E-05	8.33E-05	0.58	0.029257	0.231966
COMPOUND_312_2644a10_172118	Rp	+	312.2644	10.17				(335.2563, 770.03)(330.3012, 1407.24)(331.2999, 274.34)(313.2728, 6213.93)(314.2758, 1508.06)(315.2679, 213.96)	#DIV/0!	#DIV/0!	#DIV/0!	#DIV/0!	#DIV/0!	#DIV/0!	#DIV/0!	#DIV/0!	-0.32	0.244081	0.339722	0.02	0.963262	0.981972
COMPOUND_312_2671a9_99728	Rp	+	312.2671	10.00				(335.2468, 787.08)(647.5463, 106.24)(330.3008, 3135.38)(331.3034, 882.73)(332.307, 52.01)(313.2741, 7559.96)(314.276, 2494.37)(315.2752, 100.79)	55907.3	16398.31	50864.79	14885.3	67171.5	21307.77	67195.84	21163.98	0.75	0.007306	0.01599	-0.09	0.785234	0.909997
COMPOUND_312_2675a10_563439	Rp	+	312.2675	10.56				(625.5199, 580.19)(335.2517, 1281.82)(336.2569, 454.04)(330.2976, 1387.81)(313.275, 217949.97)(314.278, 42423.14)(315.2812, 5822.7)(642.6, 671.47)	290222.5	187814	239311.2	149641.2	168231.6	136961	208085.5	173479.5	-0.63	0.020253	0.039938	0.41	0.192716	0.505089
COMPOUND_312_2681a10_60154	Rp	+	312.2681	10.60				(625.523, 650.85)(335.2498, 900.6)(336.2475, 381.87)(330.3008, 1119.43)(313.2748, 242493.42)(314.2779, 51107.61)(315.2812, 6801.54)(642.5963, 719.83)	380801.8	140574.2	364895.5	121626.8	269276.5	94403.05	381827	138227.9	-0.85	0.004239	0.009721	0.92	0.008531	0.14883
COMPOUND_312_3024a11_580105	Rp	-	312.3024	11.58	Arachidic acid	1	10 eV: 311.2956(100), 51.5024(2), 135.1534(2)	(347.2713, 479.03)(348.2729, 94.41)(349.2678, 277.57)(311.2953, 45630.87)(312.2985, 9585.06)(313.3011, 1382.97)(314.3046, 171.96)(357.3004, 860.73)(358.3033, 96.62)	98359.89	46493.09	119438	45350.61	125459.5	47007.97	114622	58925.72	0.63	0.034925	0.064204	-0.74	0.038547	0.259981
COMPOUND_312_3026a11_240537	Rp	-	312.3026	11.24				(347.2673, 249.09)(311.2947, 11124.19)(312.2983, 2032.0)(313.2993, 208.64)(357.2913, 1376.6)	15335.59	4808.622	16922.06	5169.478	19222.83	7445.196	19706.31	6838.353	0.61	0.022233	0.043237	-0.08	0.790192	0.91097
COMPOUND_313_2258a0_7485032	Hilic	+	313.2258	0.75				(336.2181, 2137.4)(337.2198, 579.46)(331.2508, 844.31)(314.2334, 158928.25)(315.2366, 30503.12)	181607.6	98029.23	189389.6	108177.4	171554.5	110925.9	193681.3	89004.74	-0.21	0.477545	0.580686	0.27	0.439915	0.715816
COMPOUND_313_2259a6_604511	Rp	+	313.2259	6.60	ACar 10:1	2	10 eV: 314.2328(100), 85.0277(25), 281.9817(13)	(314.2335, 23465.77)(315.2369, 4546.92)(316.2402, 790.63)(317.2408, 74.01)	65391.3	35461.1	67383.24	40328.84	53483.57	33840.01	68896.32	33596.75	-0.46	0.122139	0.19343	0.51	0.143066	0.448726
COMPOUND_313_2264a6_763148	Rp	+	313.2264	6.76				(314.2339, 12049.53)(315.236, 2687.19)(316.2426, 499.67)	28875.3	12707.83	25688.83	10519.21	23009.18	13258.1	21013.77	8696.694	-0.58	0.038168	0.069543	0.11	0.742694	0.889653
COMPOUND_314_1263a7_063569	Rp	-	314.1263	7.06				(427.1116, 1239.62)(428.1154, 321.05)(429.1252, 99.96)(349.0964, 1659.68)(350.101, 336.11)(351.092, 451.97)(313.1189, 6188.6)(314.1218, 999.77)(315.1265, 266.93)(316.1326, 33.44)	28825.23	17051.22	24465.07	14248.55	20865.8	9513.077	20068.05	13517.39	-0.64	0.005291	0.011914	-0.37	0.155252	0.466404
COMPOUND_314_1274a7_0736856	Rp	+	314.1274	7.07				(337.1198, 411.02)(315.1354, 22027.99)(316.1388, 4280.43)(317.1402, 735.75)(318.1316, 91.75)	42477.62	27302.54	34537.96	19916.9	27555.92	15103.98	23006.18	21610.1	-0.74	0.002233	0.005483	-0.32	0.242461	0.549318
COMPOUND_314_1275a6_8774543	Rp	+	314.1275	6.88				(315.1356, 14190.64)(316.138, 2663.9)(317.1426, 513.73)	32214.86	19391.83	26802.98	14060.95	23888.45	12288.55	19671.2	11418.05	-0.61	0.008787	0.0189	-0.36	0.179345	0.493746
COMPOUND_314_2261a8_915458	Rp	+	314.2261	8.92				(629.4547, 857.71)(630.4579, 602.3)(631.4508, 186.23)(337.2155, 17411.31)(338.2184, 3947.59)(339.2234, 791.46)(651.4393, 577.22)(652.44, 388.7)(332.2638, 4014.0)(333.2645, 1096.0)(334.259, 172.33)(353.1822, 380.1)(354.1884, 156.58)(355.1895, 1937.51)(315.2327, 562747.7)(316.2365, 117971.78)(317.2404, 15980.05)(318.2434, 1835.48)	154259.4	41216.14	151100.6	50570	757768.7	198892.7	938244.2	398024.4	1.28	2.1E-09	2.11E-08	0.45	0.039475	0.261543
COMPOUND_314_2452a10_503139	Rp	-	314.2452	10.50				(663.4815, 228.56)(427.2337, 5110.19)(428.236, 1868.46)(429.2337, 722.78)(673.4655, 124.44)(313.2382, 13394.39)(314.2414, 2484.68)(315.2464, 418.9)	24598.67	11000.64	31038.26	15467.17	37007.86	17941.87	34035.68	18697.16	0.91	0.002633	0.006377	-0.81	0.022617	0.213113
COMPOUND_314_284a10_898516	Rp	+	314.2840	10.90				(629.5921, 7323.49)(337.2723, 21309.5)(338.2754, 4222.19)(339.2804, 1876.24)(651.5379, 848.61)(332.3293, 236.64)(315.2905, 32958.99)(316.2936, 7158.85)(317.3009, 1521.44)(646.6002, 9952.63)(647.5947, 2263.22)	122214.2	33451.57	119489.4	31599.03	66387.5	28017.7	68778.88	23244.23	-1.36	1.29E-09	1.39E-08	0.10	0.657718	0.842058
COMPOUND_314_3306a10_467288	Rp	+	314.3306	10.47				(629.6558, 659.25)(353.2908, 260.78)(354.2711, 2554.04)(315.3378, 60538.92)(316.341, 12285.39)(317.3439, 1459.72)(318.3443, 108.93)(646.7039, 1589.6)(647.6951, 713.65)(648.7, 523.65)	165802.6	99547	124976.8	90994.29	178291.8	64020.64	206704.5	111457.6	0.38	0.158537	0.239133	0.67	0.03471	0.249266
COMPOUND_315_2416a7_087783	Rp	+	315.2416	7.09	Decanoylcarnitine	1	10eV: 316,247(100), 85.0283(25), 257.1774(17), 155.143(9)	(316.2493, 93208.73)(317.2524, 17671.54)(318.255, 2543.31)(319.2608, 309.43)	200916.7	145342.6	202770.2	151500.7	158562.1	122047.3	227608.8	153862.1	-0.49	0.093722	0.15406	0.62	0.073562	0.326462
COMPOUND_315_2419a0_71815705	Hilic	+	315.2419	0.72				(631.4768, 328.13)(338.2331, 8867.36)(339.2358, 1983.02)(653.4468, 774.06)(316.2485, 383654.78)(317.252, 67376.16)(318.2547, 8559.69)(319.2566, 846.77)(648.5366, 349.17)	318828.9	235801.4	348235.8	291974	290275.4	230174.4	404946	270638.7	-0.25	0.384707	0.487541	0.45	0.185058	0.499463
COMPOUND_316_1725a10_502323	Rp	+	316.1725	10.50				(633.3567, 1201.33)(634.3658, 652.92)(635.3568, 833.6)(339.1553, 1581.94)(317.1799, 18871.35)(318.1833, 3934.98)(319.1846, 730.55)(159.0944, 1067.99)(159.5951, 182.39)(160.0929, 116.43)	20283.52	10114.6	22995.97	10768.43	30264.77	12372.28	33321.31	17143.91	0.88	0.00045	0.00126	0.03	0.926733	0.966461
COMPOUND_316_2051a9_232506	Rp	+	316.2051	9.23				(339.1946, 5200.35)(340.1974, 1619.56)(334.2391, 14574.35)(317.2126, 47269.07)(318.2172, 11170.89)	45637.63	27872.2	43885.84	27438.97	19025	#DIV/0!	14740	3729.281	-1.25	5.76E-09	4.99E-08	0.09	0.676559	0.851655
COMPOUND_316_2051a9_221732	Rp	+	316.2051	9.22				(339.1946, 5200.35)(340.1974, 1619.56)(334.2391, 14574.35)(317.2126, 47269.07)(318.2172, 11170.89)	59644.52	39240.49	47425.08	29531.84	34666	25810.81	11165	#DIV/0!	-1.16	4.76E-08	3.29E-07	0.07	0.738115	0.888668
COMPOUND_316_206a8_972682	Rp	+	316.2060	8.97				(633.4437, 942.48)(634.4296, 273.87)(339.1942, 9836.98)(340.1981, 2295.87)(355.1847, 2409.61)(334.2377, 4128.26)(317.2125, 34606.7)(318.2167, 8944.28)	72047.11	52080.14	73456.09	46341.44	31701	8865.055	27965.33	11091.05	-1.43	2.37E-12	7.99E-11	-0.04	0.816899	0.92157
COMPOUND_316_2405a8_828208	Rp	+	316.2405	8.83				(671.4434, 155.7)(339.2311, 8305.79)(340.2337, 2114.43)(341.2371, 356.35)(355.2046, 489.26)(356.2069, 169.66)(357.2063, 2785.12)(317.2488, 64193.72)(318.2521, 13960.59)(319.2579, 2122.42)(320.2561, 249.87)(650.5152, 117.63)	40887.26	16854.47	38507.28	13966.72	150451.4	60260.15	243690.4	112040.3	1.09	1.84E-07	1.09E-06	0.55	0.01426	0.179521
COMPOUND_317_142a9_949789	Rp	+	317.1420	9.95				(356.0947, 280.63)(318.1499, 14636.25)(319.1534, 3020.7)(320.1526, 696.51)	18413.87	5277.742	19783.59	5880.536	22746.21	5866.51	26538.61	8738.549	0.75	0.005951	0.013217	0.14	0.655328	0.841269
COMPOUND_318_1218a5_6670127	Rp	-	318.1218	5.67				(671.2271, 309.17)(353.0896, 364.38)(317.1139, 9438.74)(318.1175, 1771.81)(319.1208, 203.13)	23413.67	11986.74	18882.58	10023.89	23693.64	11055.72	17243.03	8534.42	0.06	0.803332	0.858616	-0.40	0.134636	0.43508
COMPOUND_318_1585a1_2901298	Hilic	+	318.1585	1.29				(319.1659, 11993.71)(320.1689, 2443.53)(321.1707, 366.62)	15683.04	9319.123	13843.17	6672.822	8075.444	5132.722	9760.459	4021.377	-1.10	4.86E-07	2.65E-06	0.33	0.139951	0.444883
COMPOUND_318_1889a10_716282	Rp	+	318.1889	10.72				(637.3925, 5100.7)(638.3959, 2473.1)(639.3939, 3909.4)(640.4094, 1240.55)(641.391, 1795.77)(675.366, 1141.91)(676.3633, 570.99)(677.3668, 601.5)(319.1957, 32571.33)(320.1993, 6856.79)(321.2034, 1288.76)(160.1003, 1111.3)(160.6043, 65.02)(161.0938, 420.03)(654.423, 900.26)(655.4336, 436.56)(656.4037, 1206.1)	59662.3	21411.96	62857.61	25802.03	78255.5	28366.61	84655.32	34013.54	0.82	0.001483	0.003745	0.17	0.556703	0.781554

									1st trimester				3rd trimester									
									Control		Pre-eclampsia		Control		Pre-eclampsia		Time			Interaction		
Compound	Column	Ion_mode	Mass	Retention_	Identification	ID_level	MSMS_spectra	CompositeSpectrum	Mean	SD	Mean	SD	Mean	SD	Mean	SD	Value	raw p	FDR_p	Value	raw p	FDR_p
COMPOUND_318_2184a9_643887	Rp	+	318.2184	9.64				(637.4417, 1638.82)(341.2101, 42343.65)(342.2143, 9982.0)(659.4298, 601.46)(357.1782, 5749.5)(358.1807, 1319.61)(359.1799, 313.53)(336.2538, 2140.03)(337.2536, 953.69)(338.2387, 240.47)(319.2255, 49471.52)(320.229, 10267.3)(321.2377, 1946.17)	113955.8	68159.27	132743.3	86071.51	76515	67458.18	107209.3	113793.2	-0.79	0.000989	0.002581	-0.43	0.119188	0.408824
COMPOUND_318_2189a9_642285	Rp	-	318.2189	9.64				(431.1952, 2007.82)(432.1937, 580.46)(433.1821, 1576.77)(353.1882, 4577.98)(354.1916, 1102.48)(355.1923, 2770.7)(356.1966, 632.24)(357.2027, 522.08)(317.2119, 235526.89)(318.2152, 51423.41)(319.2242, 12901.96)(635.4278, 1039.89)(636.4307, 491.44)(637.416, 221.7)(363.2145, 7779.45)	210967.5	172638.5	244000	148299.9	231199.3	134957.3	243616.6	125562.5	-0.93	4.97E-05	0.000165	-0.35	0.171369	0.487277
COMPOUND_318_2194a10_001665	Rp	-	318.2194	10.00				(671.4004, 123.14)(431.2007, 780.79)(432.2009, 195.64)(433.2091, 870.3)(434.202, 372.43)(353.1892, 1297.42)(354.1906, 309.02)(355.1978, 1060.8)(317.2118, 65463.47)(318.2153, 14828.56)	76520.54	37687.39	83846.68	38813.64	104168	14969.78	95396.18	47945.14	-0.88	0.000238	0.000701	-0.24	0.366411	0.669574
COMPOUND_318_2201a9_668321	Rp	+	318.2201	9.67				(637.4406, 1269.25)(341.2101, 22405.57)(342.2143, 5570.8)(659.4385, 395.21)(357.1763, 389.26)(336.2544, 2951.58)(337.2525, 1316.28)(338.265, 1193.27)(319.2258, 15781.44)(320.2305, 3648.91)(321.238, 1036.91)(322.2323, 105.27)	140225.5	111377.4	148844	92656.6	142215	130553.7	196171.4	140941.3	-0.43	0.069643	0.118456	-0.49	0.08388	0.346484
COMPOUND_318_2203a9_186296	Rp	+	318.2203	9.19				(341.2104, 27237.8)(342.2145, 6123.58)(659.4285, 471.4)(660.4217, 200.55)(336.2539, 20055.08)(337.255, 4855.35)(338.2552, 860.52)(319.2278, 28253.81)(320.2316, 6441.59)(321.2408, 1998.3)	204934.3	63108.61	196290.1	69447.45	131009	166092.3	28022.33	13429.53	-1.19	2.46E-08	1.83E-07	0.08	0.703777	0.86442
COMPOUND_318_2209a9_3014145	Rp	+	318.2209	9.30				(675.399, 1683.0)(341.2097, 55581.71)(342.2135, 11938.27)(659.4314, 1716.56)(660.4322, 596.58)(661.4425, 559.91)(357.1833, 527.89)(358.1837, 180.09)(336.2538, 5300.2)(319.2282, 6399.66)(320.2343, 1277.33)(321.2395, 837.98)(322.2489, 103.97)	214461.2	77424.57	195802.3	80030.68	69061.5	72805.84	45899.5	26232.95	-1.44	1.36E-12	5.69E-11	0.12	0.540422	0.776663
COMPOUND_318_2209a9_026607	Rp	+	318.2209	9.03				(341.2119, 28827.84)(342.2154, 6893.08)(659.4048, 61.42)(336.2461, 114.85)(337.2499, 39.31)(319.228, 30999.19)(320.2325, 7343.67)(321.2407, 2265.02)(322.2383, 175.79)	189680.6	83873.16	211243.6	96409.51	213719.3	132954.3	241408.5	98379.58	-0.92	6.53E-05	0.000211	-0.33	0.203202	0.5149
COMPOUND_318_221a10_008217	Rp	+	318.2210	10.01				(637.4457, 9691.73)(638.4527, 2220.06)(639.434, 1260.72)(675.3839, 947.52)(341.2096, 15990.59)(342.2139, 3877.41)(357.1864, 1781.44)(358.1814, 370.23)(359.1822, 442.28)(319.2276, 89472.64)(320.2308, 19090.47)	100779.4	43617.74	103557.1	47268.22	102222.6	82649.71	113172.5	72808.29	-0.85	0.000315	0.000903	-0.28	0.285185	0.593016
COMPOUND_319_1642a6_1318603	Rp	+	319.1642	6.13				(320.1711, 13290.75)(321.1746, 2195.29)(322.1772, 373.99)	34131.14	9582.073	31880.51	7100.308	30702.25	9899.278	34840.41	8405.245	-0.15	0.600729	0.694311	0.47	0.179336	0.493746
COMPOUND_320_233a9_90998	Rp	+	320.2330	9.91				(343.2257, 62111.8)(344.2302, 12634.09)(359.193, 9019.89)(338.2694, 3015.63)(321.2438, 29304.41)(322.2471, 6857.75)(323.2562, 1291.55)	251206.8	123997	274162.9	140785.8	144158.6	122170.6	139870.9	112420.9	-1.16	2.9E-07	1.66E-06	-0.18	0.449073	0.722351
COMPOUND_320_2345a9_747945	Rp	-	320.2345	9.75				(355.2037, 1459.71)(356.2073, 276.17)(357.2018, 537.47)(358.2054, 73.14)(359.1872, 92.09)(319.2277, 50484.45)(320.2309, 10657.04)(321.2369, 2053.39)(322.2439, 253.31)(365.2301, 992.82)(366.2326, 291.13)(367.23, 55.5)	74586.55	38271.28	110228.6	79097.48	74878.45	53983.24	107659.3	113581	-0.52	0.072619	0.122501	0.20	0.560211	0.783425
COMPOUND_320_2346a9_793816	Rp	-	320.2346	9.79				(355.2037, 1459.71)(356.2073, 276.17)(357.2018, 537.47)(358.2054, 73.14)(359.1872, 92.09)(319.2276, 54780.79)(320.2308, 10638.41)(321.237, 1889.52)(322.2385, 237.64)(365.2296, 709.75)(366.232, 205.36)(367.2386, 86.82)	123800.9	82532.52	160388.6	109891.4	97164	104949.5	148115.2	185789.9	-0.68	0.017588	0.035176	0.09	0.79397	0.913187
COMPOUND_320_2347a9_8436165	Rp	-	320.2347	9.84				(355.2045, 16006.14)(356.2078, 3791.46)(357.2023, 4804.69)(358.2047, 1245.22)(359.2134, 299.01)(319.2274, 31163.08)(320.2308, 6099.9)(321.237, 1252.65)(322.2431, 138.56)(365.2304, 391.24)(366.2312, 115.06)(367.2246, 50.08)	465993.2	485766.5	528021.7	555941.1	110860.8	85706.56	168316.5	252784.1	-0.97	0.00061	0.001672	0.04	0.908511	0.958319
COMPOUND_320_2356a9_889301	Rp	-	320.2356	9.89				(355.2042, 20506.49)(356.2079, 4309.65)(357.2028, 6996.43)(358.2043, 1436.12)(359.215, 445.36)(319.2272, 559744.56)(320.2308, 109073.77)(321.2358, 17005.97)(322.2411, 2015.56)(639.4609, 595.66)(640.4664, 266.17)	1160962	528494.8	1290259	550275.2	472631	602448.4	358447.5	576553.1	-1.19	3.64E-06	1.59E-05	-0.15	0.584204	0.797833
COMPOUND_320_3097a11_230653	Rp	+	320.3097	11.23				(679.5785, 444.05)(680.6003, 310.97)(663.6252, 2315.57)(321.3159, 21855.95)(322.3196, 5189.78)(323.3245, 785.74)(658.6339, 2151.54)	30878.09	12074.3	37590.02	14906.47	54348.08	29630.3	49255.95	22467.85	1.02	0.000308	0.000886	-0.42	0.190574	0.505078
COMPOUND_321_1805a6_089787	Hilic	+	321.1805	6.09				(643.3679, 203.25)(344.1697, 1605.7)(345.1738, 324.16)(322.1882, 166721.77)(323.1912, 29754.04)(324.1936, 3615.59)	63772.62	77154.48	127905.3	129623.6	11671.55	16115.89	12858.58	8423.894	-1.25	2.6E-07	1.49E-06	-0.17	0.519271	0.764125
COMPOUND_322_2118a10_580965	Rp	-	322.2118	10.58				(679.4191, 653.31)(689.4226, 964.09)(690.4306, 3395.01)(321.2047, 7380.83)(322.2079, 1261.34)(323.2158, 532.29)(324.2171, 68.72)	36395.4	15629.72	40523.3	26443.8	27174.57	12441.16	30389.16	17027.32	-0.56	0.038214	0.06957	0.48	0.130191	0.425921
COMPOUND_322_2502a10_194824	Rp	-	322.2502	10.19				(357.2204, 1158.0)(358.2232, 283.33)(359.2181, 666.51)(321.2431, 34531.41)(322.2462, 7896.34)(323.2502, 847.25)	29936.29	17652.89	30305.64	15934.26	40390.75	19502.42	63896	34255.2	-0.85	0.000745	0.001998	-0.04	0.891784	0.953702
COMPOUND_322_2502a9_863129	Rp	-	322.2502	9.86				(357.2152, 2082.01)(358.2167, 444.25)(359.2218, 3341.35)(360.2256, 822.9)(321.2431, 36695.09)(322.2464, 7863.83)(323.2517, 1144.07)(324.2564, 110.02)	41633.13	16034.23	55662.89	33431.24	64978.75	38588.24	105362.1	21268.73	-0.29	0.206946	0.297771	-0.55	0.046292	0.273966
COMPOUND_323_156a10_47007	Rp	+	323.1560	10.47				(685.284, 951.61)(686.2829, 706.84)(324.1633, 31519.52)(325.1663, 6642.0)(326.164, 19313.54)(327.1676, 4011.06)(328.1591, 8270.65)(329.1618, 1808.81)(330.1573, 1413.66)	112687.2	23909.14	112839.9	21514.64	166076.4	27758.03	166066.5	29145.9	1.44	1.38E-08	1.1E-07	-0.05	0.836432	0.929001
COMPOUND_323_2289a10_958062	Rp	+	323.2289	10.96				(685.4168, 549.78)(346.2157, 894.4)(347.2173, 309.15)(324.2356, 11116.46)(325.2392, 3132.78)(326.2354, 1238.77)	#DIV/0!	#DIV/0!	#DIV/0!	#DIV/0!	#DIV/0!	#DIV/0!	#DIV/0!	#DIV/0!	1.27	1.16E-08	9.44E-08	-0.01	0.981333	0.990571
COMPOUND_324_1804a11_034648	Rp	+	324.1804	11.03				(687.3088, 6598.04)(688.3214, 3699.79)(325.1837, 4704.62)(326.1856, 1098.23)(327.1843, 1932.72)(328.1917, 525.72)(329.1807, 764.34)(666.3685, 27699.63)	94377.69	25919.34	75429.38	32571.68	97268.27	52990.28	114331.6	61499.81	0.41	0.116712	0.186408	0.24	0.447894	0.721588
COMPOUND_324_2094a9_208561	Rp	+	324.2094	9.21				(687.3782, 244.51)(347.1941, 347.98)(348.1962, 118.1)(349.1825, 408.64)(342.2382, 411.79)(325.217, 17147.14)(326.2207, 4846.8)(327.2297, 1132.46)(328.2231, 145.65)	99930.78	28340.27	100479.6	59828.76	125104	35632.92	147043.1	73255.49	-0.83	0.000719	0.00193	-0.15	0.590563	0.802507
COMPOUND_324_2104a9_4589205	Rp	+	324.2104	9.46				(649.4136, 269.73)(650.4136, 180.95)(347.1946, 280.97)(348.2012, 83.21)(349.2008, 256.97)(342.2388, 800.32)(325.2175, 40774.29)(326.2209, 10439.91)(327.2273, 2013.98)(328.2219, 241.52)	123252.9	54573.86	118335.4	46134.5	51527.5	51135.84	48670	41636.27	-1.41	1.29E-11	3.06E-10	0.04	0.84468	0.93246
COMPOUND_324_2104a8_915277	Rp	+	324.2104	8.92				(325.2177, 32672.07)(326.2213, 8466.97)(327.2301, 3317.83)	148577.9	61870.55	161241.4	94798.96	147801	91196.07	134594.6	72942.05	-1.06	3.74E-06	1.63E-05	-0.21	0.407532	0.693371
COMPOUND_324_286a10_215273	Rp	+	324.2860	10.22				(325.2927, 16887.74)(326.2965, 4974.06)(327.2875, 1472.99)	31621.87	13824.27	26886.97	13458.37	27887.91	9209.25	37028.63	16609.63	-0.26	0.358757	0.461252	0.65	0.063626	0.307286
COMPOUND_326_0017a8_006655	Rp	+	326.0017	8.01				(348.9904, 676.91)(349.9948, 129.94)(350.9871, 761.24)(351.9908, 71.78)(352.9846, 312.78)(344.0253, 81.05)(327.0087, 12211.81)(328.0118, 1355.07)(329.0058, 12040.13)(330.0092, 1341.88)(331.0027, 3717.74)(332.0066, 307.88)(333.0006, 644.97)(334.0031, 71.49)(335.0117, 145.92)	198698.6	11207.72	193762.6	9540.042	199993.1	7894.956	201308.4	11826.31	0.20	0.490013	0.590427	0.48	0.173343	0.487961
COMPOUND_326_0718a8_964087	Rp	+	326.0718	8.96				(349.0607, 1646.08)(350.0629, 390.83)(351.0591, 32.03)(365.0269, 241.86)(344.1045, 968.93)(345.1132, 211.78)(346.099, 97.09)(327.0791, 18939.0)(328.0827, 3550.51)(329.087, 716.89)(330.083, 84.66)	45737.9	23843.65	61281.22	25817.2	43181.36	20675.23	42354.84	23775.43	0.06	0.820015	0.872719	-0.77	0.019027	0.196411
COMPOUND_326_1433a9_950292	Rp	+	326.1433	9.95				(349.1478, 400.62)(327.1502, 14173.48)(328.1554, 4293.73)(329.1595, 665.32)	20307.58	5591.677	21080.57	6559.315	23606.07	5025.68	28087.34	9079.107	0.57	0.03511	0.06444	0.23	0.465398	0.732912
COMPOUND_326_2258a9_978272	Rp	+	326.2258	9.98				(349.214, 11801.47)(350.2165, 2982.29)(351.214, 368.07)(365.1974, 1372.94)(366.1879, 482.12)(344.2592, 58688.59)(345.2614, 15467.23)(346.2701, 3099.92)(327.233, 344461.66)(328.2364, 79914.65)(329.2404, 11789.69)(330.2466, 1464.11)	411261.7	285468.2	377784.6	362613	212512.5	86345.01	428838.5	497882.9	-1.07	9.55E-06	3.84E-05	0.11	0.685169	0.853695
COMPOUND_326_2259a10_325211	Rp	+	326.2259	10.33				(653.4554, 2131.94)(654.4473, 1445.73)(655.4554, 1840.49)(691.3843, 2242.36)(349.2146, 27416.51)(350.2178, 6709.36)(351.2194, 469.84)(365.1899, 3543.67)(366.1934, 939.64)(367.1886, 394.68)(344.2602, 100502.52)(345.2624, 26286.33)(346.2652, 4564.88)(347.264, 452.79)(327.2331, 127521.34)(328.2361, 30347.03)(329.2379, 1633.28)	434984.2	220484.5	365369.4	210824	184884.3	103741.4	183444.9	102407.9	-1.43	8.22E-11	1.29E-09	0.17	0.425172	0.705315
COMPOUND_326_226a9_745729	Rp	+	326.2260	9.75				(349.2086, 293.22)(350.2199, 136.67)(351.1986, 190.99)(344.2557, 3913.48)(345.2561, 477.97)(327.2336, 125374.38)(328.2367, 30189.58)(329.2425, 5303.17)	237342	100680.5	311199.4	245427.9	182747.5	104209.9	253081	246501.6	-1.03	1.73E-05	6.58E-05	-0.08	0.773566	0.904971

Compound	Column	Ion_mode	Mass	Retention_	Identification	ID_level	MSMS_spectra	CompositeSpectrum	1st trimester Control Mean	1st trimester Control SD	1st trimester Pre-eclampsia Mean	1st trimester Pre-eclampsia SD	3rd trimester Control Mean	3rd trimester Control SD	3rd trimester Pre-eclampsia Mean	3rd trimester Pre-eclampsia SD	Time Value	Time raw p	Time FDR_p	Interaction Value	Interaction raw p	Interaction FDR_p
COMPOUND_326_2263a9_990872	Rp	+	326.2263	9.99				(349.2143, 13569.49)(350.2179, 2969.74)(351.2174, 491.84)(365.1934, 1665.19)(366.202, 520.71)(344.2594, 59759.23)(345.2622, 16007.0)(346.272, 3700.87)(327.2332, 405357.06)(328.2367, 94508.63)(329.2412, 13549.51)(330.2456, 1603.01)	415769.2	314535.2	509316.6	367142.1	896416	414227.7	817109.5	575176.2	-0.74	0.002279	0.005586	-0.29	0.287665	0.59707
COMPOUND_327_2412a0_71427613	Hilic	+	327.2412	0.71				(655.4752, 97.27)(350.2319, 326.88)(328.2488, 29775.33)(329.2519, 5955.84)(330.2603, 4216.41)(331.2504, 460.74)	97634.98	42210.89	96538.54	37959.49	114120.8	61157.01	135699.8	64422.54	0.24	0.354925	0.45726	0.45	0.148187	0.456493
COMPOUND_327_2421a7_0652266	Rp	+	327.2421	7.07	ACar 11:1	2	10 eV: 328.2476(100), 85.0306(15), 210.8617(6), 279.8413(6)	(328.2492, 19541.63)(329.2526, 4116.41)(330.2537, 573.12)(331.255, 65.35)	65621.58	29330.94	59899.26	23227.18	66271.57	32886.75	81154.02	37511.16	-0.01	0.978324	0.987833	0.60	0.059765	0.300425
COMPOUND_328_1348a10_465241	Rp	-	328.1348	10.47				(691.2495, 561.73)(441.1186, 2976.86)(442.1177, 957.33)(443.1171, 3645.39)(444.1231, 2033.7)(445.1164, 1598.4)(446.12, 355.06)(447.1076, 81.01)(363.1016, 2131.98)(364.1045, 572.37)(365.1015, 2739.95)(366.1051, 373.88)(367.1001, 1384.6)(368.1014, 344.95)(369.0964, 413.21)(701.258, 965.4)(327.1265, 30333.52)(328.1303, 9246.19)(329.1296, 3597.93)(655.2805, 2480.9)(656.2918, 1576.48)(657.2926, 329.57)(373.1314, 8465.15)(374.1337, 2094.52)(375.1323, 16149.03)(376.1355, 2970.5)(377.1379, 15988.25)(378.1423, 5275.61)(379.1272, 716.88)(769.2434, 783.63)(770.2477, 553.27)(771.2498, 795.91)	297959.7	46773.45	311739.3	54817.62	339764.9	42947.39	373146.8	46768.53	0.76	0.004106	0.009434	0.26	0.387593	0.68827
COMPOUND_328_2404a10_66481	Rp	-	328.2404	10.66	FA 22:6	1	10 eV: 327.2328(100), 283.2418(60), 59.0143(15), 229.1964(9)	(691.4745, 2548.66)(692.4788, 1315.84)(363.2087, 12558.54)(364.2121, 2748.17)(365.2064, 4022.82)(366.2095, 942.92)(367.2105, 131.2)(701.4681, 272.38)(702.4332, 935.34)(327.2324, 832931.1)(328.2363, 172401.89)(329.2395, 24450.0)(330.2421, 2039.8)(655.4584, 1491.13)(769.478, 1096.21)	1035360	330775.8	1262496	717304.1	969328.8	495142.9	1224065	591887.4	-0.36	0.167904	0.25141	0.36	0.23679	0.542473
COMPOUND_328_2422a10_646862	Rp	+	328.2422	10.65	FA 22:5	2	10eV: 329.2456(100), 60.0821(75), 121.0977(25)	(695.4662, 2325.41)(696.4553, 3265.55)(351.2301, 58683.23)(352.2337, 14193.41)(353.2341, 1511.49)(679.4629, 3855.41)(680.452, 15225.11)(681.4566, 6137.9)(367.1982, 16337.65)(346.2756, 463141.9)(347.279, 106350.41)(348.2824, 13741.96)(349.2854, 1593.59)(329.2488, 36936.77)(330.2519, 9544.97)(331.255, 1284.46)(332.2476, 133.95)	326242.6	230901.5	380123.1	235298.3	526153.6	249128.3	617653.9	345329.4	0.71	0.00814	0.017643	0.20	0.514369	0.761025
COMPOUND_328_2614a10_473845	Rp	-	328.2614	10.47				(691.4716, 350.14)(441.2597, 1152.68)(442.2603, 507.39)(363.2261, 465.88)(364.2315, 100.96)(365.236, 934.3)(366.235, 148.33)(367.2441, 396.11)(327.2539, 9935.59)(328.2573, 2284.48)(329.2612, 337.53)	89981.4	15046.59	95667.89	14823.5	100830.5	14394.23	101101.3	16329.98	0.69	0.018559	0.03702	-0.38	0.267283	0.575669
COMPOUND_328_2627a10_30045	Rp	+	328.2627	10.30	no msms			(657.5435, 143.4)(695.4789, 1355.59)(696.4984, 2467.11)(697.4932, 1256.85)(351.2516, 52671.52)(352.2551, 11685.91)(353.2591, 1945.36)(354.2543, 258.13)(367.2246, 8512.11)(368.2282, 1731.04)(369.2286, 932.92)(346.296, 27332.45)(347.2995, 6172.97)(348.294, 2653.93)(349.2954, 219.49)(329.2692, 441415.53)(330.2728, 87962.29)(331.2756, 12719.81)	142961.2	72888.16	147656.4	106326.2	159563.2	206134.9	548195.4	641414.1	-0.59	0.035488	0.065081	1.35	0.000119	0.015703
COMPOUND_328_2631a10_299084	Rp	+	328.2631	10.30				(657.5294, 199.77)(695.4847, 1716.68)(696.4694, 164.2)(697.5009, 2008.78)(351.2521, 40346.0)(352.255, 8791.2)(353.2614, 1782.57)(354.2533, 234.36)(367.2258, 6151.53)(368.2278, 1360.17)(369.2313, 913.28)(346.2961, 33972.29)(347.2995, 7664.61)(348.2978, 1962.19)(349.2926, 259.37)(329.2699, 291157.66)(330.273, 59831.69)(331.2755, 7933.39)	136551.4	73253.58	138557.3	90022.42	208458.8	160248.4	400855.3	372605	0.02	0.931907	0.956543	0.81	0.019158	0.196411
COMPOUND_328_2635a10_486962	Rp	+	328.2635	10.49				(695.4855, 2411.25)(351.2516, 10714.91)(352.2559, 2448.03)(353.2552, 463.82)(346.2961, 21192.6)(347.2992, 4720.22)(348.2982, 1221.53)(329.2694, 4264.09)(330.2699, 1049.13)(331.2725, 282.79)	187894.7	43783.86	178371.6	35586.04	285327.3	25201.86	277779.4	59270.83	1.35	1.13E-07	6.95E-07	0.09	0.744228	0.889952
COMPOUND_328_3464a10_468469	Rp	+	328.3464	10.47				(657.7128, 1463.77)(658.7027, 338.23)(367.3014, 1479.14)(329.3539, 40406.95)(330.357, 9323.95)(331.3601, 1231.26)(332.361, 125.79)	169396.2	19122.38	175495.9	19943.85	218133.2	17186.9	217855.6	15811.99	1.64	2.34E-11	5.11E-10	-0.25	0.292525	0.602481
COMPOUND_329_1057a9_944288	Rp	-	329.1057	9.94				(442.0994, 698.37)(328.098, 6477.46)(329.1014, 1784.05)(330.1001, 551.0)(331.1015, 66.59)(771.2005, 363.45)(772.1998, 600.22)(773.1878, 363.08)	29526.89	6407.85	32338.09	8173.33	32058.07	8189.155	36068.77	9004.274	0.29	0.30234	0.40312	0.16	0.631881	0.828008
COMPOUND_329_2423a8_626431	Rp	+	329.2423	8.63				(330.2474, 7596.75)(331.2506, 1851.05)(332.2462, 615.87)	27528.89	10657.92	30633.55	9962.957	28273.57	11237.95	35646.09	13577.58	0.23	0.406733	0.510438	0.26	0.436658	0.714128
COMPOUND_330_16a7_132373	Rp	+	330.1600	7.13				(661.3323, 152.7)(662.3285, 112.77)(348.193, 30657.21)(349.1968, 7401.41)(350.1998, 1215.1)(331.1675, 8818.06)(332.1724, 1819.4)(333.1708, 381.89)	60077.43	30901.59	62603.84	32275	39326.67	9886.799	19711.4	5497.313	-1.31	6.77E-11	1.1E-09	-0.14	0.46221	0.730967
COMPOUND_330_2188a7_4728866	Rp	+	330.2188	7.47				(661.4374, 713.74)(353.2125, 597.2)(354.2147, 272.03)(355.2029, 65.23)(683.4011, 275.03)(331.2279, 11083.08)(332.231, 2920.31)(333.2398, 664.09)	#DIV/0!	#DIV/0!	#DIV/0!	#DIV/0!	29888.64	6628.875	25699.65	10255.29	1.66	5.3E-19	1.19E-15	-0.33	0.030023	0.234557
COMPOUND_330_2202a8_229498	Rp	+	330.2202	8.23				(353.2103, 1258.05)(354.2173, 385.19)(331.2274, 33445.22)(332.231, 7684.6)	11743.17	1278.26	12716.67	3395.199	28777.93	7862.018	25573.21	17730.57	1.70	9.19E-17	6.9E-14	-0.42	0.019479	0.198488
COMPOUND_330_2552a10_778889	Rp	-	330.2552	10.78	FA 22:5	1	10 eV: 329.2473(100), 100.9339(7), 257.2244(4)	(695.491, 544.96)(696.5149, 81.0)(443.2297, 3012.66)(444.2272, 1278.61)(445.2319, 629.5)(365.2246, 2718.29)(366.2273, 509.02)(367.2228, 912.72)(329.2485, 191991.06)(330.2516, 46224.21)(331.2548, 6339.31)(332.258, 725.28)(375.2562, 57.97)(773.489, 873.67)(774.4812, 578.83)(775.4951, 1008.24)	122271.6	58339.82	161964.2	129441.4	108907.9	57745.48	163061.8	123172.5	-0.34	0.204496	0.295398	0.41	0.192957	0.505089
COMPOUND_330_2554a10_841849	Rp	-	330.2554	10.84				(695.458, 932.49)(696.4206, 1160.93)(443.2554, 257.93)(444.2612, 37.81)(445.2382, 68.09)(365.213, 677.51)(366.2057, 141.02)(329.2484, 114466.55)(330.2516, 26597.34)(331.2551, 3369.38)(332.258, 282.08)(375.2378, 683.47)(773.4846, 1778.81)(774.4814, 933.5)(775.4966, 2290.53)	93605.75	40031.59	105687.1	63170.14	89229.21	49082.63	112930.6	52363.54	-0.24	0.374469	0.476411	0.44	0.155166	0.466404
COMPOUND_330_257a10_784361	Rp	+	330.2570	10.78				(661.5419, 250.21)(699.4825, 3637.88)(353.2455, 13882.07)(354.2469, 4133.94)(355.2482, 1150.9)(369.2121, 7062.76)(348.2909, 15267.15)(349.2946, 3681.82)(350.3031, 1094.61)(351.2934, 131.96)(331.2644, 20381.43)(332.2689, 5601.08)(333.2748, 1252.46)	71523.94	33394.25	79120.04	46756.59	87486	35837.28	116633.7	76381.9	0.01	0.978302	0.987833	0.66	0.04635	0.273966
COMPOUND_330_2784a10_585497	Rp	+	330.2784	10.59				(661.5951, 127.49)(662.5915, 529.72)(353.2672, 633002.06)(354.271, 120611.75)(369.2408, 14092.14)(370.2431, 3075.29)(371.2489, 2236.48)(331.2847, 870523.25)(332.2887, 175353.5)(333.2912, 23645.0)	1685094	1222032	1695435	1228679	1738036	665609.7	2298982	1178028	0.04	0.881504	0.921609	0.46	0.183448	0.497141
COMPOUND_330_2787a10_601486	Rp	+	330.2787	10.60				(353.2672, 633002.06)(354.271, 120611.75)(355.2768, 17909.29)(356.2874, 2378.3)(683.5189, 921.13)(369.2403, 10165.97)(370.2434, 2382.29)(371.2463, 1371.61)(372.2476, 147.97)(348.3122, 101686.39)(349.3149, 21985.63)(350.3173, 3769.61)(351.3206, 512.68)(331.2851, 810365.75)(332.2891, 163279.95)(333.2917, 22492.22)(334.2942, 2342.13)	2530304	983201.3	2468270	888107.2	1951994	742822.9	2847866	1075323	-0.61	0.037845	0.06901	0.95	0.007607	0.138042
COMPOUND_331_2365a6_1036196	Rp	+	331.2365	6.10				(332.2444, 13004.21)(333.2479, 2207.45)(334.2504, 402.67)	21518.2	9365.92	22490.88	10632.78	17439.78	8475.423	17992.95	6986.355	-0.52	0.049279	0.086362	0.22	0.47484	0.737985
COMPOUND_331_2365a1_0914434	Hilic	+	331.2365	1.09				(332.2437, 9466.25)(333.2473, 2069.95)(334.2517, 530.23)(335.2504, 48.63)	33717.02	20628.76	36886.04	24937.56	34800	25122.23	37553.25	20526.73	-0.16	0.571673	0.668263	0.26	0.42952	0.707514
COMPOUND_332_1376a6_4594316	Rp	+	332.1376	6.46				(333.1457, 11674.3)(334.1492, 2376.75)(335.1537, 428.44)	15618.32	5073.064	14632.13	3374.047	24245.73	11521.35	18655.52	9951.862	0.90	5.76E-05	0.000189	-0.31	0.209792	0.519177
COMPOUND_332_138a6_158398	Rp	+	332.1380	6.16				(333.145, 5196.45)(334.15, 1037.49)(335.154, 213.96)(336.1492, 26.25)	21407.62	10657.55	18907.94	7468.096	29219	15616.04	22564.43	13873.25	0.74	0.005922	0.013179	-0.26	0.394674	0.690408
COMPOUND_332_1384a5_941389	Rp	+	332.1384	5.94				(333.1458, 15842.48)(334.1484, 3020.11)(335.1502, 429.94)	18454.21	7440.537	16846.88	6139.437	29580.15	16824.95	22513.61	13611.73	0.93	4.84E-05	0.000161	-0.11	0.65598	0.841269
COMPOUND_332_1449a10_500881	Rp	+	332.1449	10.50				(665.2964, 2264.32)(666.2925, 1455.15)(371.1019, 7817.71)(372.1041, 3259.63)(373.1012, 1605.21)(333.1522, 30621.4)(334.1551, 6971.49)(335.1531, 22609.42)(336.1563, 5107.1)(337.149, 8233.98)(338.1601, 2838.45)(339.1506, 1355.62)(682.3447, 1568.71)	70026.19	33114.72	74321	34003.77	104895.8	45501.48	128473.2	58878.34	0.80	0.003032	0.007218	0.21	0.496234	0.751527
COMPOUND_332_271a10_952698	Rp	-	332.2710	10.95				(367.241, 1831.34)(368.2443, 595.75)(369.2333, 461.93)(331.2638, 77675.48)(332.2673, 16812.75)(333.2695, 2453.15)(334.2717, 272.19)(663.5276, 200.82)(664.5186, 74.23)	47159.6	22764.81	61428.5	44869.3	50301.5	26984.62	74283.11	35335.16	0.10	0.694853	0.777738	0.39	0.207928	0.518913
COMPOUND_333_304a10_851194	Rp	+	333.3040	10.85				(667.6151, 2414.8)(668.5998, 188.91)(356.2938, 36561.77)(357.298, 10463.53)(358.2998, 2033.19)(689.5786, 787.18)(372.2641, 1795.51)(373.2686, 568.43)(334.3112, 70196.4)(335.3148, 16537.56)(336.319, 2355.36)(684.6409, 3038.73)	65200.22	27366.09	82907.21	53112.25	170672.8	140919.6	146347.1	101858.5	1.14	2.92E-05	0.000104	-0.63	0.04014	0.264661

									1st trimester				3rd trimester									
									Control		Pre-eclampsia		Control		Pre-eclampsia		Time			Interaction		
Compound	Column	Ion_mode	Mass	Retention_	Identification	ID_level	MSMS_spectra	CompositeSpectrum	Mean	SD	Mean	SD	Mean	SD	Mean	SD	Value	raw p	FDR_p	Value	raw p	FDR_p
COMPOUND_334_1599a10_715213	Rp	+	334.1599	10.72				(669.3059, 536.85)(670.2815, 833.39)(707.3075, 2154.45)(691.295, 1550.05)(692.2785, 970.66)(373.1428, 619.79)(335.1683, 98335.97)(336.1709, 21641.6)(337.1688, 69390.33)(338.1711, 15596.69)(339.1649, 26384.77)(340.1673, 6700.04)(341.1638, 3828.16)	260892.3	102437.3	269029.2	99240.7	380858.9	129394.9	437567.7	155420.6	0.90	0.000716	0.001923	0.22	0.468524	0.734042
COMPOUND_334_2125a9_609749	Rp	+	334.2125	9.61				(352.2495, 3526.84)(353.2563, 1274.01)(354.2609, 296.35)(373.1813, 1174.43)(335.2192, 22605.87)(336.2237, 4522.14)(337.2314, 1403.33)(686.4655, 746.07)(687.4652, 116.41)(688.4785, 601.7)	41025.13	19104.43	43302.8	21770.31	55550.67	22724.75	53176.13	14062.76	-0.63	0.005665	0.012681	-0.51	0.056372	0.295234
COMPOUND_334_2137a8_671635	Rp	-	334.2137	8.67				(369.1824, 2926.96)(370.1853, 859.81)(371.188, 1690.91)(333.2069, 68517.8)(334.2101, 14278.03)(379.2111, 657.19)(380.215, 119.41)	72759.72	59551.8	83076.27	48904.59	143484.3	6358.146	112613.9	46800.27	-0.61	0.01136	0.023687	-0.30	0.290319	0.600365
COMPOUND_334_2139a8_443624	Rp	-	334.2139	8.44				(369.1745, 5061.22)(370.1778, 1158.14)(333.2069, 16869.01)(334.21, 3390.53)(335.2189, 1056.97)(336.2222, 120.11)(379.2119, 2761.8)(380.2155, 698.49)(381.1943, 840.62)	72070.27	49660.84	83000.1	39455.76	63647	36480.39	101577	54722.82	-0.95	5.2E-05	0.000172	-0.20	0.440247	0.715839
COMPOUND_334_2139a8_490905	Rp	-	334.2139	8.49				(369.1747, 2413.52)(370.1787, 538.62)(333.2069, 30792.12)(334.2102, 7016.31)(335.2169, 1727.85)(379.2116, 2431.2)(380.215, 389.84)(381.2214, 340.46)(667.4421, 66.85)	41741.59	23131.61	52590.94	30746.35	44812.5	7265.522	54546.43	12635.53	-0.79	0.000709	0.001908	-0.10	0.717702	0.876415
COMPOUND_334_2144a9_279192	Rp	+	334.2144	9.28				(357.202, 18915.63)(358.2052, 3544.04)(691.3867, 286.18)(692.3939, 210.29)(373.1734, 1736.1)(374.1773, 438.52)(375.1699, 767.38)(335.2227, 23491.77)(336.227, 5252.62)	42305.08	20214.02	37107.43	19958.35	38909	4587.709	22874	6012.625	-1.35	1.84E-10	2.6E-09	0.15	0.46706	0.733998
COMPOUND_334_2157a8_971936	Rp	+	334.2157	8.97				(669.4412, 477.85)(357.2052, 10837.61)(358.2096, 2627.43)(352.2471, 7850.87)(353.2462, 2913.64)(373.1971, 3274.68)(335.2227, 18726.45)(336.228, 4358.08)(337.2257, 4596.27)(338.2209, 629.47)	163023	47795	160469.8	63890.07	86432.33	22945.8	125610.9	84752.12	-1.40	2.36E-10	3.28E-09	0.06	0.800923	0.916038
COMPOUND_335_2143a10_856005	Rp	+	335.2143	10.86				(671.4119, 2950.41)(672.4219, 2185.94)(353.2563, 6680.47)(354.2618, 3069.42)(336.2221, 15113.39)(337.2266, 4131.02)(338.2303, 766.63)	41678.4	11214.76	42780.55	10259.89	71188.77	19493.12	77867.85	19881.21	1.10	1.4E-05	5.43E-05	0.08	0.78454	0.909997
COMPOUND_335_3194a11_008178	Rp	+	335.3194	11.01				(671.6248, 5452.98)(672.6448, 15925.84)(673.6294, 11347.73)(709.5785, 519.4)(358.3086, 14336.21)(359.3118, 4367.66)(360.3174, 1516.24)(361.3193, 142.69)(693.6559, 3543.42)(374.2852, 4810.98)(375.2828, 1710.17)(376.2817, 707.25)(336.3268, 490341.75)(337.3304, 111133.24)(338.3354, 15958.64)(688.6461, 6039.47)	343413	221952	548252.4	458553.8	635781.3	588465	510976.3	368020.4	0.74	0.010977	0.022973	-0.76	0.026674	0.225828
COMPOUND_335_3195a10_225195	Rp	+	335.3195	10.23				(336.3274, 17208.64)(337.3311, 4032.77)(338.3389, 1463.28)(339.3288, 141.73)	35166.18	20038.69	53557.87	39788.03	67036.29	62717.61	58181.72	37575.59	0.71	0.016359	0.033041	-0.67	0.054984	0.292726
COMPOUND_336_176a10_932189	Rp	+	336.1760	10.93				(695.3668, 7060.56)(696.3648, 4769.61)(337.1833, 61087.06)(338.1861, 14960.71)(339.184, 22536.58)(340.1863, 4725.32)(341.1797, 8536.91)	124725.5	54128.38	124791.4	54792	222960	66472.41	271293.4	86319.62	1.05	4E-06	1.73E-05	0.47	0.061477	0.303349
COMPOUND_336_2294a8_728184	Rp	-	336.2294	8.73				(371.1976, 2251.26)(372.1999, 457.98)(373.2008, 2264.0)(374.2036, 490.41)(717.4341, 104.6)(335.2226, 57958.09)(336.2258, 12289.72)(337.2337, 2703.87)(338.2391, 353.79)(381.2277, 83.88)	96914.77	44889.42	118241.8	64343.28	143243.8	69267.33	185759.5	89458.2	-0.80	0.00092	0.002423	-0.33	0.232654	0.538289
COMPOUND_336_2297a9_005535	Rp	-	336.2297	9.01				(449.2019, 517.03)(450.2021, 229.27)(371.1982, 9025.99)(372.2024, 1974.21)(373.1993, 5069.26)(374.2029, 1214.13)(335.2226, 210764.53)(336.2258, 44206.97)(337.2309, 7877.56)(338.2353, 878.82)(381.2274, 868.23)(382.2306, 158.3)(383.2385, 52.22)	432433.2	220768.8	465870.6	282720.3	523572.3	323963.6	480645.8	294649.6	-0.96	3.33E-05	0.000117	-0.29	0.261321	0.572854
COMPOUND_336_2297a8_870703	Rp	-	336.2297	8.87				(371.1978, 4281.2)(372.2006, 951.48)(373.1998, 2520.51)(374.2025, 588.41)(335.2226, 82616.15)(336.2259, 16440.93)(337.2322, 3011.42)(338.2356, 319.88)(381.2226, 609.55)(382.2282, 101.46)	179076.3	83442.79	213900.4	103063.9	189643.5	93171.89	220316.4	114552.1	-0.96	3.51E-05	0.000122	-0.35	0.18055	0.493854
COMPOUND_336_2297a8_816782	Rp	-	336.2297	8.82				(371.193, 3515.2)(372.1986, 806.83)(373.1994, 1976.44)(374.2032, 522.95)(717.4313, 201.76)(335.2224, 87589.66)(336.2256, 17968.46)(337.23, 2797.67)(338.2337, 346.07)(381.2185, 375.65)	143594.5	71996	172334.9	85955.66	197020	99437.92	221289.5	114717.2	-0.87	0.000245	0.000717	-0.32	0.233159	0.538538
COMPOUND_336_2298a9_017025	Rp	-	336.2298	9.02				(449.2029, 664.59)(450.1983, 370.94)(371.1984, 10768.02)(372.2021, 2313.56)(373.1995, 6311.86)(374.201, 1425.61)(335.2225, 107723.05)(336.2258, 21758.61)(337.2331, 6408.64)(338.2396, 834.95)(339.2436, 55.01)(381.227, 1574.4)(382.23, 302.91)(383.2308, 166.79)	484156.6	187973.1	574721.8	258307	570669.5	348778	592524.6	280309.4	-0.86	0.000202	0.000602	-0.44	0.097023	0.367252
COMPOUND_336_2659a10_459847	Rp	+	336.2659	10.46				(711.4829, 1581.01)(359.2611, 568.94)(337.2741, 11905.78)(338.2778, 2865.04)(339.2735, 1829.75)(340.2767, 239.61)	56922.84	32330.73	62700.67	50008.69	65222.1	22414.71	111922.3	62242.18	0.02	0.952289	0.96951	0.81	0.011419	0.166978
COMPOUND_337_2254a0_73094666	Hilic	+	337.2254	0.73				(360.2258, 284.23)(697.4418, 1623.73)(355.2629, 292.4)(338.2335, 2082.73)(339.2367, 620.59)(340.2397, 64.12)(692.4759, 4008.0)(693.4797, 1664.17)	33028.18	11534.05	39246.63	13054.19	21230.5	9625.75	19798.44	6464.353	0.30	0.242013	0.33726	-0.15	0.635299	0.829869
COMPOUND_337_2278a9_358261	Rp	+	337.2278	9.36				(713.4113, 612.84)(360.2274, 954.46)(697.4735, 446.69)(355.2671, 4983.6)(356.2684, 1460.26)(338.2336, 10066.61)(339.2426, 2466.98)(340.2386, 535.55)	37704.18	7499.648	37006.56	8339.877	40982.92	7893.023	36094.2	9685.519	0.29	0.329905	0.430693	-0.40	0.267896	0.575669
COMPOUND_337_3328a10_780164	Rp	+	337.3328	10.78				(675.6592, 2877.41)(676.6451, 914.21)(677.6683, 854.29)(713.6498, 2423.03)(360.3172, 2751.09)(361.3189, 683.9)(697.6551, 2221.76)(338.3419, 12093.73)(339.3433, 3828.76)(340.3479, 260.34)(692.6703, 1790.15)	33054.71	12918.58	33426.47	16201.95	29892.13	10514.84	31338.11	8903.429	-0.30	0.265596	0.362498	0.17	0.597178	0.805251
COMPOUND_337_335a3_067285	Hilic	+	337.3350	3.07				(338.3424, 8158.28)(339.3455, 2037.42)(340.3525, 294.2)	#DIV/0!	#DIV/0!	#DIV/0!	#DIV/0!	#DIV/0!	#DIV/0!	#DIV/0!	#DIV/0!	-0.20	0.503275	0.603823	-0.01	0.978253	0.987903
COMPOUND_337_3354a11_230882	Rp	+	337.3354	11.23				(675.6797, 112361.09)(676.6825, 54307.33)(677.685, 15284.2)(678.6948, 2898.58)(679.6945, 521.49)(360.3243, 655626.2)(361.3279, 150329.92)(362.3315, 18951.5)(363.332, 2181.75)(697.6896, 9608.27)(698.6931, 5489.5)(699.6672, 2930.03)(376.2979, 24922.86)(377.3007, 6922.48)(378.2987, 2748.38)(338.3429, 1450368.6)(339.3471, 318514.56)(340.3499, 38001.98)(341.3537, 3358.56)	7926947	4396743	10476107	5422527	16736362	12008959	14703697	8510187	1.06	0.000264	0.000767	-0.58	0.079787	0.339484
COMPOUND_338_1928a11_190645	Rp	+	338.1928	11.19				(677.3872, 442.75)(715.3168, 4861.2)(699.3874, 422.92)(700.3449, 1935.34)(339.199, 25061.0)(340.2023, 5854.74)(341.1995, 13331.37)(342.2041, 2486.07)(343.1957, 4437.66)(344.1994, 1311.97)(345.1931, 1021.4)(694.4127, 1012.46)	80461.94	16367.71	74448.98	18341.84	173169	35780.25	162624.5	42714.27	1.01	3.58E-05	0.000124	0.43	0.118746	0.408824
COMPOUND_338_2421a11_008429	Rp	-	338.2421	11.01				(711.4811, 847.99)(721.4636, 1295.0)(722.4669, 451.03)(723.4451, 843.0)(337.235, 5607.53)(338.2383, 1037.12)(339.2324, 1887.13)(340.2352, 698.55)(675.4651, 1070.86)(676.4661, 665.69)	25781.77	10407.99	30668.87	13344.06	22737.38	10038.05	23874.38	10116.76	-0.17	0.544752	0.642631	-0.65	0.056929	0.296083
COMPOUND_338_283a10_689177	Rp	+	338.2830	10.69	no msms			(677.5378, 486.11)(356.3074, 4824.68)(339.2892, 10011.94)(340.2928, 2353.05)(341.2895, 7580.87)(342.2937, 2108.48)(694.6295, 780.23)	220443.5	133578.9	192652.6	97903.8	265972.4	77887.8	465206.5	264485.7	-0.12	0.583477	0.679416	1.22	1.89E-05	0.005768
COMPOUND_338_2833a10_692525	Rp	+	338.2833	10.69	Dublicate of 338.2830@10.69			(677.6076, 643.81)(356.3067, 3769.3)(339.2885, 6123.14)(340.2883, 1464.99)(341.2905, 6739.98)(342.2946, 2175.47)(694.6244, 517.18)	240622.3	146680.3	235205.3	159439.2	266551.2	75860.69	509619.4	257459.6	-0.11	0.637418	0.727186	1.28	2.05E-05	0.005768
COMPOUND_339_1632a10_581099	Rp	+	339.1632	10.58				(679.3473, 1288.52)(717.2992, 1459.5)(718.2857, 600.54)(340.1703, 7941.97)(341.1743, 1671.21)(342.1829, 615.13)(343.1726, 78.67)(696.3813, 1141.85)	41885.14	26867.21	41918.91	29993.73	75172.36	42403.52	120909.2	55429.87	0.73	0.001752	0.004399	0.74	0.006835	0.128278
COMPOUND_339_3517a11_553691	Rp	+	339.3517	11.55				(679.7089, 4997.76)(680.7136, 2517.56)(681.7086, 875.78)(682.714, 232.99)(717.6923, 2274.53)(362.3404, 19799.67)(363.3438, 5120.45)(364.3461, 750.74)(701.6884, 2006.16)(702.6991, 1308.09)(703.7036, 514.91)(378.3139, 1946.41)(379.3145, 794.84)(380.309, 580.84)(340.3585, 480928.53)(341.3619, 111112.28)(342.365, 13106.06)(696.752, 1090.45)(697.7728, 819.91)	284657.7	153740.1	407603.4	236566.5	501956.1	504389.6	432588.6	318525.3	0.63	0.035691	0.0654	-0.67	0.060202	0.301949
COMPOUND_340_2983a10_928763	Rp	+	340.2983	10.93	MAG 18:0 (M+NH4)+	2	10 eV: 341.3075(100), 267.2664(35), 55.0539(15)	(681.6243, 7118.8)(363.2838, 1105.35)(703.5643, 1519.1)(358.3332, 1262.6)(341.306, 140035.0)(342.3092, 31429.55)(343.307, 2274.71)(698.6635, 5260.48)	270718.6	108218.8	243588.4	89023.28	106634.1	29197.86	114116.2	39114.78	-1.46	3.5E-10	4.46E-09	0.15	0.527777	0.7696
COMPOUND_340_2986a10_907662	Rp	+	340.2986	10.91				(681.6404, 4983.89)(719.5713, 1033.69)(363.2918, 3627.42)(364.2942, 1388.51)(365.2836, 6386.27)(366.2822, 1906.83)(703.596, 1022.29)(358.3329, 1898.9)(341.306, 140035.0)(342.3092, 31429.55)(343.307, 2274.71)(698.6388, 5179.63)	194147.3	136604.5	153629.5	112576.4	101225	34699.99	100093.3	37982.87	-0.60	0.039987	0.072213	0.27	0.428188	0.707279

									1st trimester				3rd trimester									
									Control		Pre-eclampsia		Control		Pre-eclampsia		Time			Interaction		
Compound	Column	Ion_mode	Mass	Retention_	Identification	ID_level	MSMS_spectra	CompositeSpectrum	Mean	SD	Mean	SD	Mean	SD	Mean	SD	Value	raw p	FDR_p	Value	raw p	FDR_p
COMPOUND_340_3338a12_078226	Rp	-	340.3338	12.08				(339.3262, 14134.37)(340.3295, 3278.42)(341.3333, 326.49)(385.3298, 136.48)	19943.98	8747.392	22982.83	8224.438	19220	6771.418	18391.67	8229.71	-0.06	0.841427	0.888787	-0.61	0.071697	0.325554
COMPOUND_341_149a1_812361	Rp	+	341.1490	1.81				(342.1571, 30752.99)(343.1601, 6500.67)(344.1621, 800.5)(171.5827, 757.26)(172.0835, 188.84)	32585.12	40762.34	34160.29	29353.79	66098.91	73681.67	51902.73	65968.2	0.36	0.183634	0.271353	-0.58	0.074229	0.327774
COMPOUND_341_1494a5_4751654	Hilic	+	341.1494	5.48	His-Trp	3	20 eV: 110.0712 (100), 324.1403 (10), 156.0774 (8), 342.1556 (5)	(364.1386, 3103.88)(365.1416, 742.7)(366.1413, 103.54)(342.1563, 40614.25)(343.1595, 7907.64)(344.1622, 1142.25)	29062.77	37588.9	34836.42	31567.78	58872.17	60872.08	34668.45	46245.77	0.28	0.334384	0.435531	-0.77	0.027308	0.226687
COMPOUND_341_2574a7_6879916	Rp	+	341.2574	7.69				(364.2469, 50.93)(365.237, 30.73)(342.2649, 11355.71)(343.268, 2415.62)(344.2676, 436.73)	23166.63	10536.65	21764.11	7419.721	20242.58	9302.552	23101.44	8504.696	-0.47	0.1164	0.186174	0.44	0.216632	0.524744
COMPOUND_341_258a7_5795503	Rp	+	341.2580	7.58	ACar 12:1	2	20 eV: 85.0297(100), 342.2704(30), 283.1982(10)	(364.2491, 716.93)(365.2479, 286.26)(342.2649, 6259.26)(343.2699, 1259.93)(344.2694, 226.1)(345.2661, 30.16)	49436.73	33717.39	44883.41	26675.92	38490.64	34858.66	50579	31721.04	-0.48	0.096858	0.158406	0.67	0.051045	0.283822
COMPOUND_342_1496a10_620397	Rp	-	342.1496	10.62				(455.1372, 6203.33)(377.1293, 693.67)(378.1278, 169.21)(379.1161, 569.86)(729.2758, 982.55)(341.1421, 3543.86)(342.145, 1252.77)(343.1476, 885.3)(344.1505, 46.19)(683.3217, 2275.32)(387.1485, 1256.14)(388.1551, 210.04)(389.1462, 3649.41)(390.148, 813.59)(391.1299, 2667.41)(392.1255, 709.58)(393.117, 931.23)(797.2481, 380.15)(798.2358, 585.34)	37071.04	7819.579	38807.18	7705.813	43949.14	10426.4	49441.23	8227.825	0.77	0.003088	0.007337	0.32	0.28643	0.595057
COMPOUND_342_1883a10_69024	Rp	+	342.1883	10.69				(685.3863, 1473.14)(686.3979, 909.53)(687.3878, 1005.42)(707.3884, 1153.96)(708.3816, 662.9)(343.1958, 8954.92)(344.1998, 1856.52)(345.2041, 345.07)	15457.98	3185.944	16700.34	5123.762	21557	7080.465	19865.76	7031.958	1.11	8.09E-05	0.000254	-0.71	0.025042	0.219303
COMPOUND_342_2187a9_899821	Rp	+	342.2187	9.90				(685.4604, 343.05)(686.4627, 374.85)(365.2104, 4789.96)(366.2156, 1384.62)(381.1582, 1674.67)(382.159, 803.73)(383.1654, 615.88)(360.2544, 10280.12)(361.2571, 2662.79)(362.2669, 1853.26)(343.2254, 100182.21)(344.2289, 20968.43)(345.2385, 7098.06)(702.4769, 1427.96)(703.4782, 1163.29)	205905.1	79197.9	215808.7	81159.25	92506.44	102630.4	78124.32	97255.67	-1.19	2.79E-06	1.25E-05	-0.11	0.700812	0.863364
COMPOUND_342_2201a9_458428	Rp	+	342.2201	9.46				(365.2097, 15821.84)(366.2135, 4183.66)(367.2152, 952.56)(368.2077, 102.38)(381.1932, 2654.83)(382.1955, 911.93)(383.1798, 263.46)(360.2542, 29026.35)(361.2587, 7504.62)(362.2638, 2193.44)(363.262, 244.93)(343.228, 10023.1)(344.2313, 2493.59)(345.2383, 763.16)	162033.8	75768.64	151050.2	71381.06	78356.5	77199.8	81066.88	46953.03	-1.47	5.46E-12	1.53E-10	0.13	0.538628	0.776408
COMPOUND_342_2203a8_997395	Rp	+	342.2203	9.00				(365.2093, 1304.36)(366.2162, 423.83)(343.2278, 34154.21)(344.2313, 8135.04)(345.2406, 2436.75)	86697.53	35569.32	92583.64	51233.87	105857.8	70933.08	155555.1	86684.59	-0.69	0.005508	0.012342	-0.14	0.616421	0.816971
COMPOUND_342_2692a10_860364	Rp	+	342.2692	10.86				(685.5502, 623.55)(723.5022, 4119.33)(343.2728, 14544.52)(344.2745, 5347.65)(345.2709, 2866.83)(702.55, 323.53)(703.5644, 499.22)	30269.63	10109.22	33230.57	10615.18	56515.8	18718.22	51479.16	19436.82	0.20	0.434694	0.539654	-0.08	0.785698	0.909997
COMPOUND_342_2896a8_817643	Rp	+	342.2896	8.82				(365.2853, 672.53)(366.2954, 71.59)(367.271, 119.15)(707.5562, 166.33)(343.297, 35503.36)(344.2999, 7860.94)(345.3017, 1092.98)	32952.38	21093.28	32878.35	13404.2	48645.79	39748.81	29724.86	15678.94	0.48	0.112636	0.180924	-0.76	0.039191	0.261543
COMPOUND_343_273a8_000297	Rp	+	343.2730	8.00	ACar 12:0	2	10 eV: 344.2795(100), 60.0806(17), 144.0984(7)	(366.2624, 734.76)(367.2634, 333.61)(344.2807, 38715.42)(345.2844, 8059.8)(346.2859, 1185.47)(347.2907, 116.87)	73683.94	43034.71	75345.33	40460.47	56941.29	42700.5	76654.89	40617.87	-0.64	0.029771	0.055984	0.65	0.063073	0.307286
COMPOUND_344_2109a8_284668	Rp	+	344.2109	8.28				(345.2184, 5841.06)(346.2209, 1382.44)(347.2222, 315.51)(348.2173, 35.48)	44821.49	31781.63	44137.32	27629.44	36996.42	37541.33	24311.11	13766.75	-0.02	0.927006	0.95325	-0.81	0.012231	0.168989
COMPOUND_344_2345a9_795691	Rp	-	344.2345	9.80				(379.203, 1221.55)(380.2077, 288.08)(381.2033, 681.88)(382.203, 153.78)(343.2274, 10338.44)(344.2309, 2525.46)(345.2353, 489.43)(346.2432, 64.78)(687.4548, 225.04)(389.2296, 1518.25)(390.2316, 312.81)(391.2382, 40.37)	162689.2	98979.54	236956.2	167470.2	145670.2	165381.1	123109.2	242907.2	-0.97	0.000127	0.000385	-0.15	0.589394	0.801519
COMPOUND_344_2347a9_777399	Rp	-	344.2347	9.78				(379.2036, 3954.09)(380.2058, 1125.53)(381.2039, 1492.8)(382.2059, 334.29)(343.2277, 116116.12)(344.231, 28715.71)(345.238, 4803.11)(389.228, 530.11)(390.2341, 97.32)(391.2399, 42.26)	194868	81880.33	252006.4	155541.6	244786.6	214352.2	138822.9	244709.8	-0.95	0.000258	0.000751	-0.12	0.670713	0.847138
COMPOUND_344_2351a9_914334	Rp	+	344.2351	9.91				(367.2251, 20507.59)(368.2285, 5034.69)(383.1952, 2591.15)(384.1965, 835.15)(385.2032, 241.53)(362.269, 2639.17)(363.2699, 977.03)(364.2814, 525.57)(345.2429, 16397.88)(346.2506, 3541.79)(347.2567, 1025.26)	67403.73	30937.38	89514.74	60890.45	123980.3	34100.35	130536.3	68404.74	-0.77	0.001201	0.003073	-0.40	0.141226	0.447724
COMPOUND_344_2352a10_011725	Rp	-	344.2352	10.01				(457.2119, 2300.25)(458.2063, 1053.61)(379.204, 15299.33)(380.2071, 3618.16)(381.2021, 4717.38)(382.2033, 1241.77)(383.2087, 384.85)(343.2273, 527995.4)(344.231, 120745.44)(345.2344, 16295.12)(346.2389, 1804.29)(687.464, 204.02)	549147.3	264526.7	633578.7	329961.4	747185.5	325238	921542	433222.1	-0.85	0.000493	0.001375	-0.27	0.32981	0.635357
COMPOUND_345_1746a10_4704075	Rp	+	345.1746	10.47				(729.303, 492.63)(730.2649, 881.48)(368.1561, 260.58)(384.1246, 533.63)(346.1811, 31368.7)(347.1839, 6236.94)(348.1876, 1095.94)	63276.46	10532.18	60273.7	10679	95226.07	14845.85	96485.61	13057.11	1.44	6.77E-10	7.94E-09	0.19	0.427114	0.706212
COMPOUND_345_2679a9_974165	Rp	+	345.2679	9.97				(729.5099, 667.57)(730.5157, 420.62)(346.275, 25480.29)(347.278, 6364.51)(348.2811, 1119.46)	27790.67	15538.99	27192.3	18483.72	36485.67	8385.786	28278.17	14979.03	-0.75	0.000766	0.002049	0.28	0.266821	0.575669
COMPOUND_345_2889a10_488772	Rp	+	345.2889	10.49				(713.5804, 236.22)(346.2958, 16734.89)(347.2986, 3964.32)(348.2946, 1095.66)	100875.5	20854.37	95284.16	21348.99	137031.1	13342.03	138879.2	21362.83	1.18	1.77E-06	8.36E-06	0.32	0.226076	0.533671
COMPOUND_346_2119a10_500157	Rp	-	346.2119	10.50				(737.4525, 813.82)(345.2041, 10911.34)(346.207, 2362.5)(347.209, 365.14)(805.4421, 711.44)	17988.13	6810.057	21637.5	9904.18	14984.7	5649.86	18072.94	7701.39	0.12	0.631808	0.721883	-0.23	0.43427	0.712127
COMPOUND_346_215a7_3962293	Rp	+	346.2150	7.40				(693.4428, 223.53)(369.2038, 287.19)(370.2023, 135.03)(364.2499, 490.5)(365.2506, 219.01)(347.223, 13075.08)(348.2253, 3426.35)(349.2303, 529.71)	15524.35	4347.437	14205.57	3117.73	26532.43	15184.64	26310.96	15883.78	1.27	1.22E-07	7.53E-07	-0.18	0.469217	0.734042
COMPOUND_346_2499a9_872088	Rp	-	346.2499	9.87				(381.2298, 1888.74)(382.2336, 412.52)(383.226, 210.62)(345.2428, 22292.36)(346.2464, 5372.86)(347.2511, 793.11)	31458.66	15036.11	43314.91	32445.94	50384	6017.296	66718.42	37515.82	-0.88	0.000246	0.000718	-0.25	0.351315	0.658752
COMPOUND_346_2504a10_245456	Rp	-	346.2504	10.25				(381.2307, 1508.06)(345.2431, 21978.94)(346.2463, 5000.31)(347.2496, 723.73)	#DIV/0!	#DIV/0!	#DIV/0!	#DIV/0!	#DIV/0!	#DIV/0!	#DIV/0!	#DIV/0!	-0.61	0.019304	0.03837	-0.01	0.968555	0.982722
COMPOUND_347_1073a10_465133	Rp	-	347.1073	10.47				(729.2069, 458.79)(730.2048, 328.98)(731.2227, 254.12)(460.0977, 1882.32)(739.2512, 682.14)(346.1005, 36948.72)(347.1029, 9801.37)(348.098, 12617.7)(349.1014, 3346.6)(350.1008, 818.05)(693.1562, 416.75)(694.1758, 313.19)(807.203, 865.73)(808.228, 616.88)(809.2216, 351.89)	58254.43	14224.76	61096.46	15818.71	88004.79	17190.81	98431.49	17754.5	1.24	1.03E-07	6.38E-07	0.26	0.298648	0.60748
COMPOUND_347_1839a7_123609	Rp	-	347.1839	7.12				(460.1687, 452.35)(461.172, 175.82)(462.164, 26.81)(382.1534, 6189.61)(383.1571, 1228.42)(384.1516, 2105.02)(385.156, 628.18)(386.1524, 87.96)(346.1768, 17935.71)(347.1803, 3794.73)(348.1835, 579.03)(392.183, 492.51)(393.188, 173.19)	51955.94	27605.16	54656.02	29064.62	25898.75	8539.067	18554.57	6088.545	-1.30	5.62E-11	9.89E-10	-0.24	0.222124	0.531023
COMPOUND_349_1708a10_581104	Rp	+	349.1708	10.58				(737.2865, 1212.29)(738.2707, 1046.12)(739.3003, 560.87)(367.1964, 4340.84)(350.1789, 30947.1)(351.1815, 6798.02)(352.1795, 34969.13)(353.183, 7652.95)(354.1752, 14114.86)(355.1781, 4051.85)(356.177, 2658.39)	68574.33	48024.39	67864.11	49678.98	109524.2	65122.11	174515.8	84271.55	0.76	0.002115	0.005235	0.53	0.065033	0.308265
COMPOUND_350_1185a6_711507	Rp	-	350.1185	6.71	no msms			(349.1112, 28613.2)(350.1144, 5821.85)(351.1117, 1849.52)(352.1111, 221.39)	28321.86	13523.5	41305.65	33270.7	351472.1	173612.1	174082	187817.7	1.88	3.32E-14	4.32E-12	-0.81	0.000504	0.035763
COMPOUND_350_2105a7_7050176	Rp	+	350.2105	7.71				(373.201, 5735.91)(374.2046, 1383.48)(368.2446, 6068.37)(369.2473, 1506.25)(370.2576, 885.92)(351.2173, 6585.25)(352.2214, 1717.49)(353.23, 541.41)	65587.47	33699.58	58358.54	40353.27	29866	12162.24	28243	20308.05	-1.29	3.22E-09	3.01E-08	0.23	0.31237	0.618152
COMPOUND_350_2109a7_7852817	Rp	+	350.2109	7.79				(373.201, 6224.37)(374.206, 1564.69)(368.2439, 12015.31)(369.2476, 3293.81)(351.2174, 11871.1)(352.2214, 2469.23)(353.2296, 938.1)	79043.04	41072.32	81231.59	39233.27	47060	14417.24	33979	22045.05	-1.27	2.56E-09	2.5E-08	-0.13	0.551771	0.780002
COMPOUND_351_1585a1_6661193	Hilic	+	351.1585	1.67				(703.3013, 277.2)(374.1475, 5190.09)(375.1509, 1260.45)(376.1533, 345.07)(369.1792, 131.11)(352.166, 9553.64)(353.1692, 2191.23)(354.1733, 469.86)(355.1835, 65.4)	64002.51	19588.35	68426.67	28216.23	51456.79	18667.67	61180.75	27449.06	-0.52	0.072372	0.122176	0.20	0.557013	0.781554
COMPOUND_351_1585a4_397621	Rp	-	351.1585	4.40				(386.1273, 556.52)(387.1244, 52.26)(388.1234, 80.05)(747.3014, 114.24)(350.1505, 10965.09)(351.1533, 2662.25)(352.1557, 269.4)(701.311, 176.93)	23151.1	7584.526	24882.73	8922.166	21819.75	3669.832	22602	7746.807	-0.31	0.260494	0.357485	-0.06	0.85013	0.936641
COMPOUND_351_1592a4_416396	Rp	+	351.1592	4.42				(725.3101, 74.03)(390.1125, 130.24)(352.1662, 33864.54)(353.1693, 8112.35)(354.1735, 1218.43)(355.1734, 158.89)	121363	35691.92	123323.7	44500.71	99187.57	36976.5	113020.4	46874.14	-0.51	0.070685	0.119866	0.22	0.50007	0.753664
COMPOUND_351_1862a10_858264	Rp	+	351.1862	10.86				(741.3372, 4857.48)(742.3228, 4092.67)(743.3282, 3141.93)(390.1525, 182.82)(369.2057, 14795.3)(352.1946, 75284.05)(353.1973, 16566.22)(354.1949, 36046.04)(355.1982, 7614.7)(356.1906, 13880.08)(357.1932, 3515.65)(358.1902, 2097.94)(720.3887, 3552.42)(721.3699, 1244.02)(722.3791, 1950.42)	#DIV/0!	#DIV/0!	#DIV/0!	#DIV/0!	#DIV/0!	#DIV/0!	#DIV/0!	#DIV/0!	1.34	2.03E-07	1.19E-06	0.00	0.990899	0.995763
COMPOUND_351_2404a8_487484	Rp	+	351.2404	8.49				(703.4849, 552.87)(704.4826, 267.58)(369.274, 1201.93)(352.2492, 10249.21)(353.2511, 2977.04)(354.2619, 1757.66)(720.5102, 2802.92)(721.5137, 1097.86)(722.516, 315.2)	77590.65	22672.4	64024.38	22196.71	33978.5	5618.389	32745.83	10135.79	-1.32	1.87E-07	1.11E-06	0.51	0.062079	0.305246
COMPOUND_351_2408a8_812827	Rp	+	351.2408	8.81				(369.2733, 1269.2)(352.2496, 8083.84)(353.2501, 2409.57)(354.2621, 1419.9)(720.5116, 2224.27)(721.5126, 960.39)	68841.91	26698.77	56410.24	22285.31	28037.6	7772.177	34574.29	10869.06	-1.28	8.79E-08	5.59E-07	0.36	0.151144	0.462181

									1st trimester				3rd trimester									
									Control		Pre-eclampsia		Control		Pre-eclampsia		Time			Interaction		
Compound	Column	Ion_mode	Mass	Retention_	Identification	ID_level	MSMS_spectra	CompositeSpectrum	Mean	SD	Mean	SD	Mean	SD	Mean	SD	Value	raw p	FDR_p	Value	raw p	FDR_p
COMPOUND_352_2244a8_018731	Rp	-	352.2244	8.02				(465.1933, 1527.05)(466.194, 289.71)(467.1887, 327.77)(387.1902, 1413.54)(388.1932, 327.48)(389.1965, 5225.99)(390.2002, 1279.59)(351.2173, 24918.39)(352.2205, 5553.19)(397.2139, 1142.28)(398.2078, 114.16)(399.2207, 6597.28)(400.224, 1584.82)	51515.07	52797.31	55531.93	49245.45	78812.67	78041.66	57886.4	29761.03	-0.72	0.002344	0.005731	-0.30	0.267796	0.575669
COMPOUND_352_2246a8_980364	Rp	-	352.2246	8.98				(387.1938, 2033.28)(388.1966, 496.78)(389.1949, 834.39)(390.1955, 355.41)(351.2175, 10547.32)(352.2222, 2498.97)(353.2309, 1165.29)(354.2339, 143.13)	59671.4	30869.54	62722.93	31179.95	54704.33	21010.04	52479.56	13676.02	-1.16	1.67E-07	1.01E-06	-0.18	0.438803	0.715597
COMPOUND_352_2246a8_884727	Rp	-	352.2246	8.88				(387.1926, 1698.78)(388.1979, 324.06)(389.1941, 876.96)(390.1965, 262.22)(351.2174, 7625.38)(352.2205, 1713.2)(353.2304, 863.55)(354.2355, 103.51)	43765.33	22858.72	53213.5	32346.22	29995	4887.84	34277.29	17058.5	-0.92	3.1E-05	0.000109	-0.27	0.263302	0.574821
COMPOUND_352_2334a10_849869	Rp	-	352.2334	10.85				(739.4663, 3002.52)(465.2222, 1630.8)(466.2255, 501.87)(467.2075, 800.24)(387.2034, 323.5)(388.2105, 35.31)(389.2085, 916.54)(749.4806, 2065.21)(750.4746, 1354.58)(751.4845, 1145.47)(351.2289, 8405.42)(352.2322, 1410.73)(353.2292, 312.62)(703.4403, 1587.33)(397.2351, 7927.48)(398.2385, 2239.26)(399.2392, 468.21)(817.4799, 413.61)	48732.02	12924.96	54949.84	18132.94	32393	12925.8	39754.7	11754.63	-0.55	0.040667	0.073265	0.10	0.758569	0.896744
COMPOUND_352_2625a10_223319	Rp	+	352.2625	10.22				(743.507, 835.97)(375.2523, 21943.56)(376.2555, 5380.07)(377.2672, 1578.56)(727.4951, 1299.93)(728.4879, 492.61)(729.5051, 1837.07)(730.5035, 463.9)(391.2261, 1241.94)(392.235, 566.23)(393.2176, 536.65)(370.297, 38760.49)(371.2994, 10064.24)(372.3062, 1957.65)(373.3093, 254.16)(353.2699, 35681.61)(354.2736, 7504.13)(355.2796, 2428.41)(722.5358, 410.27)	53595.8	35891.06	60830.04	45845.41	62079.42	32760.96	96460.53	55226.01	0.11	0.694125	0.777309	0.63	0.053571	0.288052
COMPOUND_353_3313a10_579868	Rp	+	353.3313	10.58				(707.6758, 1040.11)(376.32, 13016.33)(377.3241, 3434.67)(378.3314, 919.95)(392.2931, 927.1)(371.364, 5782.72)(354.3373, 11227.82)(355.3399, 3130.68)(356.3454, 752.64)(357.3427, 67.2)	31194.92	13265.66	38340.11	18508.8	49381.36	35074.49	47829.36	25510.29	0.80	0.006189	0.013717	-0.47	0.166902	0.480028
COMPOUND_354_1033a6_715043	Hilic	+	354.1033	6.72				(377.0918, 5976.57)(378.0951, 886.11)(379.0963, 129.88)(731.1942, 847.25)(732.2007, 350.63)(733.1929, 87.09)(355.1111, 1851.2)(356.1121, 183.4)	19164.21	4606.613	21307.8	5518.484	25797.57	7116.442	29638.04	7896.351	0.91	0.000516	0.001432	0.11	0.712766	0.872838
COMPOUND_354_1497a10_579311	Rp	-	354.1497	10.58				(743.2595, 270.39)(744.2707, 787.14)(467.1343, 5053.6)(468.1383, 666.06)(469.1306, 5409.23)(470.1259, 1653.7)(471.1311, 2192.56)(472.1321, 889.55)(473.1361, 742.44)(389.1155, 3490.72)(390.1179, 746.76)(391.1177, 3797.93)(392.1214, 796.95)(393.1159, 2175.43)(394.1252, 598.96)(395.1108, 547.94)(753.2907, 977.73)(754.3068, 602.46)(755.2983, 1110.79)(756.3058, 865.94)(757.2945, 732.41)(353.1422, 17526.06)(354.1461, 6464.64)(355.1502, 1967.09)(356.1638, 275.67)(707.2914, 972.23)(708.2786, 1580.04)(709.2856, 1127.81)(399.1465, 16942.69)(400.1502, 4288.83)(401.1474, 22152.98)(402.1514, 4481.55)(403.1437, 8626.87)(404.1483, 2625.21)(405.1464, 1708.06)(821.3058, 667.5)(822.2967, 499.51)(823.283, 726.02)	201231.3	108120.4	209723.3	127643.9	238470.8	134219.9	361772.1	146516	0.32	0.215762	0.308505	0.75	0.017118	0.19372
COMPOUND_354_1746a10_470297	Rp	+	354.1746	10.47				(747.3631, 1004.49)(748.3756, 155.43)(749.3469, 1273.51)(393.1238, 691.45)(372.1942, 1217.78)(355.1811, 35468.58)(356.1851, 8962.54)(357.1907, 1957.68)(726.3601, 955.59)(727.3787, 559.09)(728.3477, 931.0)	65228.49	13390.02	61691.07	14134.94	100116.3	17830.99	100843.4	17812.68	1.36	2.73E-09	2.62E-08	0.19	0.413522	0.696003
COMPOUND_354_2399a8_686489	Rp	-	354.2399	8.69				(389.2047, 695.07)(390.1975, 100.05)(391.2114, 1180.92)(392.2158, 266.17)(393.2232, 313.04)(353.2328, 14459.58)(354.2364, 3455.2)(355.2396, 590.08)(356.2428, 84.63)	39033.92	21351.03	45866.15	22832.95	78488	33286.01	59721.2	21393.48	-0.77	0.001028	0.002672	-0.43	0.114755	0.401909
COMPOUND_354_278a10_459615	Rp	+	354.2780	10.46	no msms			(709.5937, 313.49)(377.2673, 587964.06)(378.271, 124014.46)(379.2805, 32846.94)(393.2408, 12969.22)(394.2449, 3718.19)(395.2433, 1642.85)(372.3119, 925775.5)(373.3158, 200988.2)(374.3189, 30050.97)(375.3215, 3470.26)(355.2853, 583538.9)(356.2893, 119046.83)(357.2911, 19798.4)(358.2953, 2820.99)	978542.8	600135.2	1100807	944968.9	1100027	652355.7	2292947	1289399	0.00	0.996256	0.999363	1.10	0.000508	0.035763
COMPOUND_354_2794a10_43812	Rp	+	354.2794	10.44				(709.5937, 313.49)(377.2673, 587964.06)(378.271, 124014.46)(393.2408, 12969.22)(394.2449, 3718.19)(372.3119, 925775.5)(373.3158, 200988.2)(374.3189, 30050.97)(355.2853, 583538.9)(356.2893, 119046.83)(357.2911, 19798.4)	1003242	679554.3	671806.3	544988.9	795936.9	865848	1181000	1362139	-0.39	0.197329	0.286885	0.85	0.01996	0.199116
COMPOUND_355_2369a8_151008	Rp	+	355.2369	8.15				(356.2437, 9425.17)(357.2464, 2206.88)(358.2483, 593.65)	26843.08	13246.41	21023.12	12181.61	27081.43	9457.592	29459.65	13805.08	0.17	0.55931	0.656827	0.42	0.220442	0.53038
COMPOUND_355_273a7_978727	Rp	+	355.2730	7.98	ACar 13:1	2	10 eV: 356.2832(100), 163.1108(12), 278.4125(10), 44.1736(3)	(378.2645, 1994.92)(379.2665, 595.75)(380.2745, 247.06)(356.2804, 10711.06)(357.2843, 2509.35)(358.2863, 530.92)(359.2759, 71.16)	36620.04	15851.6	33462.72	13243.7	32586.46	14302.57	33351.69	16220.04	-0.40	0.153809	0.232938	0.29	0.38436	0.686423
COMPOUND_356_144a9_927284	Rp	+	356.1440	9.93				(395.1274, 898.48)(374.166, 1369.41)(357.1521, 9657.59)(358.1567, 2643.34)(359.1538, 3289.6)(360.1585, 877.44)(361.1511, 1313.53)	28660.65	10278.86	27837.96	9894.783	33343.67	1307.866	33935.11	10310.34	-1.07	5.63E-06	2.37E-05	-0.03	0.892679	0.953702
COMPOUND_356_2713a10_901843	Rp	-	356.2713	10.90				(747.5002, 555.83)(355.2638, 34137.99)(356.2676, 9337.53)(357.2707, 1088.91)(358.2727, 139.79)(825.5267, 1709.25)	21226.82	9170.031	31634	47118.16	23423.86	13320.34	36194.74	18501.76	0.50	0.034352	0.063307	0.31	0.250218	0.56013
COMPOUND_356_2723a9_733602	Rp	+	356.2723	9.73				(713.5725, 145.14)(374.2927, 484.01)(357.2799, 108157.39)(358.2833, 27669.47)(359.2869, 3946.38)(360.2832, 560.57)	79201.7	64569.95	87085.6	126026.7	64288.9	83119.62	91954.33	94833.81	-0.54	0.029797	0.055984	0.60	0.038288	0.259981
COMPOUND_356_2936a10_660318	Rp	+	356.2936	10.66				(379.283, 745789.94)(380.2869, 158695.0)(395.2567, 16036.79)(396.2583, 4549.44)(374.3275, 608008.44)(375.3313, 132881.06)(357.3009, 862338.8)(358.3049, 188872.89)(359.3076, 25796.81)(730.6216, 650.47)	1043549	1023525	916011.9	847512.2	642301.4	815802.4	2290047	2422353	-0.20	0.489057	0.589698	0.93	0.008713	0.14883
COMPOUND_356_2946a10_693461	Rp	+	356.2946	10.69	MAG 18:1	1	10eV: 265.2548(100), 339.2913(90), 247.2426(35), 109.1034(22), 357.3053(15)	(379.2834, 772951.25)(380.2875, 163999.9)(381.2901, 23961.18)(382.2947, 3337.33)(395.2567, 11830.38)(396.2593, 2815.79)(397.2607, 1649.75)(398.2596, 205.52)(374.3281, 347925.16)(375.3312, 78793.88)(376.3332, 11797.05)(377.3358, 1740.35)(378.3355, 185.76)(357.3016, 543354.8)(358.3053, 115006.61)(359.3077, 16585.91)(360.3115, 2372.08)	1841413	1194453	1859414	1480080	1868554	1102725	4798112	2492278	-0.11	0.642878	0.732302	1.39	7.61E-06	0.005716
COMPOUND_357_1368a10_464027	Rp	-	357.1368	10.46				(749.2597, 619.73)(750.2573, 483.13)(751.2592, 608.1)(759.2915, 1070.04)(760.3028, 375.75)(761.2903, 881.29)(356.1293, 49132.06)(357.1325, 12701.0)(358.1335, 3287.28)(359.1352, 401.11)(713.2769, 1002.11)(714.278, 5218.61)(715.2816, 1880.44)(716.2794, 2937.59)(827.2409, 608.36)(828.2522, 471.03)	#DIV/0!	#DIV/0!	#DIV/0!	#DIV/0!	#DIV/0!	#DIV/0!	#DIV/0!	#DIV/0!	-0.74	0.0082	0.017755	0.01	0.975386	0.986465
COMPOUND_357_1827a10_86012	Rp	+	357.1827	10.86				(715.3863, 6189.11)(716.391, 3248.64)(753.3467, 892.54)(754.3418, 3921.81)(737.3269, 1980.83)(738.3416, 1259.02)(396.1456, 519.85)(397.1434, 1149.01)(375.2154, 4228.63)(376.2184, 662.68)(377.2078, 75.11)(358.1892, 1565.61)(359.1939, 340.92)(360.1882, 2900.07)(361.1857, 1353.03)(362.1868, 1662.62)(732.3941, 3149.68)	80490.89	19484.04	82801.56	19331.21	148404.8	43782.51	149869.9	30836.54	1.44	8.64E-08	5.51E-07	-0.43	0.129455	0.425745
COMPOUND_357_2894a8_258104	Rp	+	357.2894	8.26	ACar 13:0	2	10 eV: 358.3049(100), 165.099(55), 294.3479(27), 85.1828(5,5)	(380.2803, 5525.88)(358.2961, 10211.54)(359.3004, 2156.46)(360.3048, 411.29)(361.2934, 58.97)	48013.74	42062.01	38529.48	26836.23	50598.15	47862.49	43430.7	32577.1	-0.17	0.537618	0.636056	0.22	0.479188	0.742181
COMPOUND_358_1615a10_691497	Rp	+	358.1615	10.69				(717.3439, 1632.95)(718.3458, 1130.17)(719.349, 780.82)(755.2959, 1658.2)(739.3341, 1319.6)(376.1836, 1970.85)(359.1672, 24423.85)(360.1759, 6328.9)(361.1685, 15351.34)(362.1739, 3822.03)(363.1644, 5679.1)(364.1675, 1103.73)(365.1669, 722.45)(734.3701, 2556.17)(735.3772, 1375.71)	87279.6	22716.81	91532.67	25997.22	110454.6	33372.41	116032.2	31712.79	0.70	0.015305	0.031136	-0.12	0.720405	0.876947
COMPOUND_358_1623a10_467041	Rp	-	358.1623	10.47				(751.2632, 853.37)(471.1609, 1400.68)(393.1486, 3183.81)(357.1552, 14321.77)(358.1594, 2976.09)(359.1696, 537.6)(715.3244, 621.72)(403.1494, 1825.64)(829.3143, 289.9)(830.3163, 532.44)	23903	10108.27	29191.36	8227.041	37746.7	7781.894	36850.02	11911	0.95	3.91E-05	0.000133	-0.16	0.528331	0.7696
COMPOUND_358_1848a9_927043	Rp	+	358.1848	9.93				(717.3757, 499.28)(718.3608, 385.77)(719.3813, 308.57)(381.1586, 1123.23)(382.1675, 289.45)(383.1629, 889.9)(397.1335, 339.76)(359.1926, 10815.23)(360.1952, 2501.99)(361.204, 1305.18)(362.1951, 155.38)	38166.87	13820.79	41830	13907.55	42455.33	1730.932	41519	15817.61	-1.03	1.04E-05	4.14E-05	-0.22	0.391523	0.690408

									1st trimester				3rd trimester									
									Control		Pre-eclampsia		Control		Pre-eclampsia		Time			Interaction		
Compound	Column	Ion_mode	Mass	Retention_	Identification	ID_level	MSMS_spectra	CompositeSpectrum	Mean	SD	Mean	SD	Mean	SD	Mean	SD	Value	raw p	FDR_p	Value	raw p	FDR_p
COMPOUND_358_214a9_015355	Rp	-	358.2140	9.02				(751.378, 220.27)(752.3767, 55.67)(753.3983, 72.45)(471.1953, 1100.45)(472.1962, 244.56)(473.1883, 395.37)(474.1766, 27.51)(393.1803, 1878.89)(394.184, 439.46)(395.1933, 1273.32)(761.4113, 177.04)(762.3977, 40.7)(357.2068, 13829.12)(358.2097, 3212.07)(403.21, 4581.49)(404.2123, 1180.44)(405.217, 624.2)(406.228, 78.02)	109223.5	52603.95	109024.4	56727.94	122863.7	22636	115057	53843.82	-1.11	1.93E-06	8.98E-06	-0.10	0.70283	0.86442
COMPOUND_358_214a9_025965	Rp	+	358.2140	9.03				(381.2044, 7756.14)(382.208, 2303.53)(376.2496, 2285.35)(377.2404, 1009.76)(359.2204, 15331.95)(360.2249, 3440.15)(361.2312, 1110.41)	78080.96	41770.17	81779.38	33972.59	119631	71990.3	88278.63	27892.38	-0.94	3.39E-05	0.000119	-0.31	0.213356	0.520562
COMPOUND_358_2141a9_018258	Rp	-	358.2141	9.02				(751.3814, 147.34)(752.3782, 81.36)(753.3816, 146.14)(471.1965, 722.84)(472.1987, 199.26)(473.1936, 560.06)(474.2031, 99.66)(393.1806, 1806.82)(394.1835, 384.46)(395.1923, 947.47)(396.1977, 224.23)(761.4127, 172.75)(762.4118, 60.14)(357.2068, 12038.38)(358.2099, 2837.5)(403.2092, 6285.25)(404.2124, 1498.88)(405.2196, 608.23)(406.2246, 78.41)	113952.6	70161.7	118153	66378.49	133003.7	26304.15	106309.5	58804.05	-1.09	2.65E-06	1.2E-05	-0.16	0.531456	0.771657
COMPOUND_358_217a9_217477	Rp	+	358.2170	9.22				(381.2046, 7053.69)(382.2073, 1726.77)(397.1941, 1739.65)(398.2006, 534.86)(399.1939, 3305.37)(376.2498, 6117.01)(377.2553, 1327.02)(359.2223, 13347.84)(360.2263, 3323.02)(361.2359, 1184.0)	100701.7	37802.12	90850.48	44344.01	20332.5	856.3063	29331.71	14938.33	-1.47	2.41E-12	7.99E-11	0.13	0.500992	0.753664
COMPOUND_358_3094a10_909369	Rp	+	358.3094	10.91				(717.6525, 1593.16)(718.644, 554.23)(381.2981, 527484.06)(382.3019, 112025.93)(383.3046, 17468.8)(384.3086, 2559.93)(739.6043, 2565.71)(740.5953, 1153.63)(376.343, 64121.32)(377.3463, 15937.28)(378.3476, 2713.44)(397.2726, 12545.59)(398.2706, 3542.43)(399.2687, 1839.87)(359.3166, 208336.77)(360.3201, 49735.46)(361.323, 7298.81)(362.3221, 1045.22)(734.6606, 2029.67)(735.6565, 947.44)	1315781	907638.4	1005062	793438.7	720776.3	455060.1	705666.6	569134.9	-0.59	0.04875	0.085503	0.35	0.319119	0.625463
COMPOUND_358_3101a10_927794	Rp	+	358.3101	10.93				(717.6395, 1969.1)(718.6271, 898.7)(381.2993, 290370.47)(382.3021, 65212.25)(383.3047, 10515.27)(384.3064, 1488.53)(385.2994, 154.6)(739.6034, 2288.99)(740.6256, 1303.55)(397.2718, 18155.11)(398.2749, 5383.89)(399.2713, 2554.13)(400.2832, 395.91)(376.3433, 169977.31)(377.3464, 40414.08)(378.3492, 7071.73)(379.3531, 1083.22)(359.3159, 948949.94)(360.3199, 203503.94)(361.3223, 28491.7)(362.3254, 3292.28)(734.6464, 4045.43)(735.6486, 2751.25)	2159549	1057308	1947626	896862.4	984292.6	579064	1218670	777447.6	-1.27	5.55E-06	2.33E-05	0.41	0.174909	0.48931
COMPOUND_359_2471a9_459599	Rp	+	359.2471	9.46				(360.2539, 16419.92)(361.256, 5176.36)(362.2644, 2030.85)(363.2691, 303.13)	126425.5	50172.55	116968.2	43226.92	65490.5	38969.36	70405.86	29550.43	-1.41	2.65E-11	5.63E-10	0.10	0.615926	0.816971
COMPOUND_359_2709a0_918676	Hilic	+	359.2709	0.92				(382.2591, 1437.25)(741.5306, 6538.96)(360.2751, 7538.6)(361.2787, 1653.6)(362.2795, 245.05)(736.5734, 1083.01)	40121.39	20191.7	41666.85	16105.48	53904.44	27127.6	34271.95	18834.62	0.00	0.997735	0.999401	-0.57	0.087615	0.350433
COMPOUND_360_1944a6_5683947	Rp	+	360.1944	6.57	Cortisone	1	10eV: 361.2009(100), 322.2927(11), 121.0607(6)	(383.1844, 2612.5)(384.1878, 608.54)(385.1801, 35.81)(399.1564, 401.44)(400.1621, 78.36)(361.2023, 53727.71)(362.2051, 12594.35)(363.2088, 2200.11)(364.2099, 311.35)	58121.53	16089.87	56191.28	14533.52	120306.6	24446.04	127249.2	48217.68	1.24	7.44E-06	3.04E-05	-0.17	0.569133	0.788601
COMPOUND_360_2294a8_915713	Rp	-	360.2294	8.92				(395.1975, 747.65)(396.2048, 120.92)(397.2051, 4023.11)(359.2222, 12385.22)(360.2254, 2720.37)(361.2352, 853.27)(362.2394, 105.66)(405.2176, 691.37)(406.2086, 65.84)	128962.3	82121.36	162227.7	110607.1	183766	133515.6	226349	202293.2	-0.86	0.000268	0.000778	-0.34	0.20259	0.5149
COMPOUND_360_2294a9_128733	Rp	-	360.2294	9.13				(395.1985, 1880.95)(396.2002, 389.02)(397.2053, 11452.22)(398.2086, 2985.29)(359.2222, 20340.73)(360.2255, 4662.58)(361.2309, 1170.1)(362.2359, 160.1)(405.2276, 11813.23)(406.2308, 2946.76)(407.2328, 553.21)	424630.1	257530	518678	356820.5	3256000	4970357	1859806	1887534	-0.87	0.000536	0.00148	-0.10	0.721237	0.877485
COMPOUND_360_2296a9_092889	Rp	-	360.2296	9.09				(395.1991, 1851.65)(396.203, 382.58)(397.207, 4751.86)(398.2109, 1185.44)(359.2225, 25264.85)(360.2258, 6007.6)(361.2316, 1399.45)(362.2416, 187.37)(405.2276, 15773.11)(406.2312, 4095.32)(407.2339, 789.66)	235982.4	215181.7	208658.4	199614.8	2548341	4323235	762832.5	1434714	-0.55	0.021563	0.042131	-0.29	0.306297	0.613089
COMPOUND_360_2311a9_514435	Rp	+	360.2311	9.51				(383.2205, 9600.42)(384.2237, 2744.68)(385.2331, 1305.29)(399.1962, 1712.09)(400.2044, 467.53)(401.2013, 426.74)(378.2656, 74119.06)(379.2686, 18790.51)(380.2792, 11170.71)(361.2386, 8960.84)(362.2421, 2734.25)(363.2465, 797.67)(181.1274, 591.37)(181.6179, 22.96)	124988.2	72005.61	128229.2	67799.07	112618.3	64513.66	153040.8	94950.71	-1.05	9.9E-06	3.97E-05	-0.14	0.598574	0.805251
COMPOUND_361_2273a8_595282	Rp	+	361.2273	8.60				(362.2341, 5005.1)(363.2376, 1154.3)(364.2364, 235.18)	29444.33	7041.994	27537.8	6672.233	28871.64	5214.131	27041.7	5055.614	0.05	0.866998	0.910247	-0.11	0.759726	0.896853
COMPOUND_361_2616a8_963542	Rp	+	361.2616	8.96				(379.3, 472.99)(362.2699, 21321.05)(363.2729, 5299.95)(364.2806, 2355.2)	52505.35	32464.63	44803.28	30061.28	46898.67	10859.17	35966	22688.28	-0.94	2.67E-05	9.62E-05	0.29	0.237365	0.542834
COMPOUND_361_3202a10_602238	Rp	+	361.3202	10.60				(723.6681, 447.61)(384.3108, 99.91)(385.3102, 1564.35)(379.3452, 486.16)(362.327, 19203.13)(363.3301, 4405.7)(364.3317, 1078.06)	20629.47	8560.757	16610.91	7494.388	15189.2	3799.416	21445.24	9747.781	-0.24	0.357123	0.459566	0.85	0.007117	0.131884
COMPOUND_362_2103a6_80662	Rp	+	362.2103	6.81	Cortisol	1	20 eV: 363.2204(100), 43.0158(75), 147.0816(75), 267.1743(65), 97.0611(55), 201.1308(55)	(725.4251, 136.36)(385.1994, 7870.78)(386.203, 2012.11)(387.2028, 316.51)(747.4063, 324.09)(748.4084, 159.39)(749.4092, 71.46)(401.1723, 876.57)(402.1782, 148.27)(403.1685, 86.55)(363.2178, 300556.06)(364.221, 67719.42)(365.2242, 10478.79)(366.2278, 1258.98)	290636.7	97627.4	287770.5	92798.21	584199.9	164292.5	485252.7	269459.4	1.41	1.55E-06	7.5E-06	-0.62	0.052324	0.286125
COMPOUND_363_1912a6_3340254	Rp	+	363.1912	6.33				(727.4054, 118.0)(364.1978, 10924.07)(365.2019, 2040.9)(366.2045, 530.48)	28398.27	7131.223	27166.5	5911.209	25804.64	7406.407	27580.94	7227.745	-0.23	0.430728	0.535615	0.31	0.375003	0.67723
COMPOUND_363_2784a9_301902	Rp	+	363.2784	9.30				(364.2855, 118077.09)(365.2887, 29192.21)(366.291, 4205.25)(367.2937, 493.0)	237533	151323.8	166915.1	139195	160388.3	98588.55	115359.3	86957.02	-1.33	9.07E-10	1.01E-08	0.18	0.420066	0.700732
COMPOUND_363_2784a9_190326	Rp	+	363.2784	9.19				(364.2855, 53081.75)(365.289, 13053.08)(366.2854, 2417.37)(367.2921, 330.56)	158121.8	98483.2	117556	89570.39	109611.5	60844.04	89896.2	64809.52	-1.31	2.24E-09	2.24E-08	0.15	0.510716	0.757607
COMPOUND_364_0983a6_384417	Hilic	+	364.0983	6.38				(729.2054, 120.73)(365.1061, 107234.94)(366.1097, 14646.54)(367.1111, 3460.11)(368.113, 412.79)	11430	6212.934	12007.22	11979.33	30266.14	13553.71	41818.96	62155.56	1.40	1.61E-11	3.7E-10	0.02	0.926182	0.966461
COMPOUND_364_0985a6_3290606	Hilic	+	364.0985	6.33	Melibiose	1	10 eV: 365.1044 (100), 203.0512 (5)	(387.0943, 66.69)(365.1061, 81302.7)(366.1095, 10603.95)(367.1111, 2667.58)(368.1144, 313.12)	9745.382	6587.447	11021.72	14120.5	31323.64	12434.57	40147.67	46957.48	1.35	7.91E-10	9.09E-09	0.15	0.489757	0.748114
COMPOUND_364_1822a10_469843	Rp	+	364.1822	10.47				(729.4031, 788.2)(730.3915, 582.86)(767.3036, 1475.06)(768.3271, 948.18)(769.3263, 810.45)(751.3701, 2064.68)(752.3848, 1178.21)(753.3693, 966.95)(403.1363, 805.82)(365.1884, 20820.28)(366.1927, 4975.55)(367.1906, 25866.97)(368.1942, 5481.98)(369.187, 10240.26)(370.1918, 2665.88)(371.1849, 1903.56)(746.398, 1946.1)(747.4001, 1307.15)	122016.2	20817.01	126057	25213.26	166196.4	27686.23	170861.1	28964.57	1.30	5.6E-07	2.98E-06	-0.03	0.91076	0.959323
COMPOUND_364_2216a9_625648	Rp	-	364.2216	9.63				(363.2146, 11519.83)(364.218, 2555.95)(365.2169, 473.62)	16303.55	5167.889	20828.97	10485.65	25145	6469.416	33710.67	9830.983	-0.28	0.230332	0.325617	-0.56	0.048511	0.279822
COMPOUND_365_1795a10_715941	Rp	+	365.1795	10.72				(731.3713, 1278.28)(732.3707, 4534.16)(733.3752, 1951.09)(404.1571, 931.2)(366.1862, 73347.06)(367.1899, 17991.74)(368.1924, 3259.01)(748.4006, 3331.92)	110127.5	38251.28	109080	39102.61	176181.2	46842.49	203705.4	67411.07	1.03	6.95E-05	0.000222	0.24	0.399441	0.690408
COMPOUND_365_2025a11_020949	Rp	+	365.2025	11.02				(731.3864, 2546.55)(769.3513, 3095.47)(753.3866, 1711.63)(366.2097, 14554.79)(367.212, 4010.52)(368.2092, 6715.3)(369.2119, 1874.68)(370.2078, 2335.83)(371.2106, 1119.23)(372.2069, 515.51)(748.4538, 2322.35)	40681.68	11460.39	41141.83	10304.93	66820.69	16364.64	68617.36	18469.3	1.11	5.88E-05	0.000192	-0.39	0.211821	0.519471
COMPOUND_365_3672a11_613963	Rp	+	365.3672	11.61				(731.7407, 5964.0)(732.7417, 3898.71)(733.7575, 1157.91)(769.6651, 1318.56)(388.3558, 19000.78)(389.3598, 5431.6)(390.3606, 970.93)(753.686, 879.89)(404.3289, 2428.87)(405.3352, 609.48)(406.3321, 614.36)(383.4, 8595.5)(384.4021, 2541.91)(366.3744, 290079.97)(367.3774, 77877.8)(368.3805, 9985.26)(369.3807, 1355.84)	190914.5	103076.1	266899.5	174485.9	336292.6	307287.3	272797.5	211957.1	0.58	0.051013	0.089124	-0.65	0.063803	0.307286
COMPOUND_366_2142a10_882574	Rp	-	366.2142	10.88				(767.4431, 695.96)(479.1926, 3668.34)(480.2018, 1407.07)(365.2092, 7905.21)(366.213, 1966.87)(367.2223, 540.96)	70434.58	9676	72623.72	10086.41	52897.56	23207.99	58516.78	24601.08	-0.93	0.000842	0.002226	0.21	0.502397	0.753763
COMPOUND_366_218a10_01806	Rp	+	366.2180	10.02				(389.204, 843.41)(390.2121, 227.2)(391.2092, 357.42)(755.4289, 205.57)(756.4394, 237.52)(367.2255, 15369.11)(368.2297, 3830.59)(369.238, 1284.3)(370.2344, 174.05)	70170.72	28927.51	81251.84	38995.77	108132.8	44117.85	124706.5	53405.5	-0.80	0.00118	0.003023	-0.26	0.365472	0.669574
COMPOUND_366_2525a10_537385	Rp	-	366.2525	10.54				(767.4605, 492.16)(768.472, 1323.71)(365.2459, 12621.24)(366.2498, 2562.11)(367.2435, 4672.22)(368.2467, 971.46)(369.2531, 145.32)(845.5222, 1495.85)	19455.53	5678.738	23019.33	10813.92	15529.92	3900.514	17952.11	5992.827	-0.69	0.019242	0.038302	0.15	0.661814	0.844422

									1st trimester				3rd trimester									
									Control		Pre-eclampsia		Control		Pre-eclampsia		Time			Interaction		
Compound	Column	Ion_mode	Mass	Retention_	Identification	ID_level	MSMS_spectra	CompositeSpectrum	Mean	SD	Mean	SD	Mean	SD	Mean	SD	Value	raw p	FDR_p	Value	raw p	FDR_p
COMPOUND_366_2527a10_596336	Rp	-	366.2527	10.60				(767.4744, 1097.78)(768.476, 1937.8)(365.2458, 15282.5)(366.2491, 3234.2)(367.2444, 5366.06)(368.2469, 1348.08)(369.2447, 213.73)(845.5135, 1675.86)	36842.9	14170.5	41372.76	14480.27	21817.23	8503.286	28787.77	11942.53	-1.16	7.39E-05	0.000234	0.37	0.266975	0.575669
COMPOUND_366_3294a11_815989	Rp	+	366.3294	11.82				(771.5941, 800.98)(772.6088, 74.12)(755.6515, 1024.22)(367.337, 11070.7)(368.3402, 3163.9)	38816	18776.25	34520.57	14943.25	29322.67	15231.19	16360.86	5198.457	-1.26	2.72E-09	2.62E-08	0.02	0.929228	0.967463
COMPOUND_366_3322a11_066952	Rp	+	366.3322	11.07				(733.6999, 3352.61)(734.6811, 1987.11)(735.6757, 1332.67)(367.3376, 16327.86)(368.3408, 5422.02)(369.3492, 2065.76)(370.349, 210.44)(750.7122, 4888.27)(751.6955, 1786.86)(752.6895, 1378.67)	206300	46818.42	199944.7	44509.76	237686	141575.5	199417.9	106355.2	-0.91	0.00016	0.000482	0.22	0.413326	0.696003
COMPOUND_367_1953a10_932911	Rp	+	367.1953	10.93				(735.3932, 3121.81)(773.3658, 2925.3)(774.3454, 10489.79)(757.4067, 2711.84)(368.2035, 17385.8)(369.2064, 5223.75)(370.2117, 2036.29)(371.2067, 214.06)(752.4371, 1322.08)(753.4367, 1024.8)(754.4205, 1506.64)	145677.8	56694.1	149706.9	60223.07	281086.7	77896.02	359892.9	116188.6	1.01	1.22E-05	4.82E-05	0.53	0.040193	0.264661
COMPOUND_367_2726a7_9651318	Rp	+	367.2726	7.97	ACar 14:2	2	10 eV: 368.282(100), 55.0523(6)	(390.2654, 2164.76)(391.2713, 796.64)(368.2806, 5379.46)(369.283, 1463.58)(370.2784, 431.55)(371.2944, 44.34)	39913.33	19550.89	34554.79	16977.71	26485.85	19509	34766.76	16019.87	-0.54	0.063448	0.109323	0.38	0.26442	0.575338
COMPOUND_367_3816a12_037113	Rp	+	367.3816	12.04				(390.369, 364.9)(391.3648, 85.84)(392.3553, 120.83)(406.3468, 381.96)(407.3502, 187.27)(368.3892, 12168.6)(369.3915, 3377.33)(370.3955, 482.5)(752.8073, 706.45)(753.8089, 209.33)(754.8289, 147.45)	15760.76	5135.549	18485.36	7080.267	22791.63	15345.63	17222.91	7219.606	0.23	0.402158	0.506238	-0.48	0.139042	0.444794
COMPOUND_368_1654a8_309799	Rp	-	368.1654	8.31				(367.1579, 28028.77)(368.1611, 6217.03)	43747.35	29664.97	45829.14	16779.89	21133.62	4244.779	25049.64	10467.81	-0.73	0.000219	0.000646	-0.55	0.012228	0.168989
COMPOUND_368_1658a7_434831	Rp	-	368.1658	7.43	steroid sulfate	3	10 eV: 367.158(100), 96.9599(6)	(367.1579, 794406.7)(368.1617, 164919.44)(369.1591, 45765.38)(735.3236, 3361.99)(736.326, 1470.59)(737.3237, 623.94)(738.322, 192.1)(739.3292, 38.76)	2698883	1264167	2957206	1183172	983266.3	540257.5	1136107	747830.9	-1.10	1.68E-07	1.01E-06	-0.35	0.111675	0.397302
COMPOUND_368_2195a7_7771025	Rp	-	368.2195	7.78				(403.1873, 941.47)(404.189, 295.33)(405.1923, 2109.41)(781.42, 107.15)(367.2122, 14592.46)(368.2155, 2874.96)	43320.1	25127.78	47511.78	25989.84	30652.33	826.84	35168.89	14887.82	-1.22	2.2E-08	1.67E-07	-0.19	0.396591	0.690408
COMPOUND_368_2195a8_789687	Rp	-	368.2195	8.79				(367.2122, 11017.15)(368.2149, 2836.24)(369.223, 1038.01)(370.2284, 116.71)	24667.6	11475.76	27210.69	14564.25	22280.33	7511.632	21893.56	11737.14	-1.18	9.12E-08	5.79E-07	-0.16	0.483346	0.746055
COMPOUND_368_2196a8_819054	Rp	-	368.2196	8.82				(367.2121, 16287.92)(368.2146, 3459.92)(369.2232, 870.44)(370.2241, 124.17)	30600.02	14200.86	34018.16	15900.48	29994.33	8590.299	25953.7	8841.671	-1.15	1.87E-07	1.11E-06	-0.21	0.360172	0.666906
COMPOUND_368_2236a11_193171	Rp	+	368.2236	11.19				(775.3974, 4066.44)(776.4029, 2246.48)(391.218, 819.69)(392.2166, 1103.61)(393.2162, 463.63)(407.1861, 8658.43)(408.1889, 1680.06)(409.1869, 3360.76)(410.2026, 1135.43)(411.1849, 1342.67)(369.2326, 9309.32)(754.4863, 1742.64)(755.489, 1232.28)(756.489, 597.52)	33567.14	11055.77	27933.41	9601.435	127115.1	34982.28	126216.7	32445.49	1.04	1.4E-05	5.43E-05	0.22	0.400154	0.690408
COMPOUND_368_3451a12_545252	Rp	+	368.3451	12.55				(386.3809, 12841.38)(387.383, 3874.06)(388.3791, 1170.35)(369.3528, 154639.45)(370.356, 44504.38)(371.3588, 6637.71)	965742.9	109890.1	983616.9	118447.9	1025173	112841.3	983773.6	105902.5	0.53	0.078219	0.130869	-0.55	0.13026	0.425921
COMPOUND_368_3771a11_22971	Rp	+	368.3771	11.23				(737.7837, 1092.51)(775.7181, 2068.85)(759.7361, 1949.0)(760.7473, 280.41)(761.7262, 1308.71)(369.3843, 107556.59)(370.3868, 29125.66)(371.3875, 3860.65)(754.8012, 1401.72)	76631.26	45143.27	80975.36	42998.21	177902.4	102696.6	186781	115896.5	0.77	0.001202	0.003074	0.41	0.129501	0.425745
COMPOUND_369_2103a11_190524	Rp	+	369.2103	11.19				(777.3737, 4751.84)(761.4096, 1485.86)(762.4034, 2000.44)(370.2182, 31300.52)(371.2227, 13159.18)(372.2258, 2694.53)	85975.14	18227.38	73506.46	16774.74	168229.3	56990.51	183992.6	46510.05	1.12	1.45E-06	7.03E-06	0.23	0.359636	0.666906
COMPOUND_369_2882a8_350857	Rp	+	369.2882	8.35	ACar 14:1	2	10 eV: 370.2965(100), 115.0756(11), 329.1844(7)	(370.2966, 55892.55)(371.2996, 12926.31)(372.2998, 2197.24)(373.309, 272.64)	83991.21	58146.69	72548.09	42449.7	55106	45857.51	70551.33	42600.98	-0.65	0.027428	0.052082	0.51	0.145118	0.452012
COMPOUND_370_1808a6_951668	Rp	-	370.1808	6.95				(369.1737, 14897.16)(370.1764, 3297.32)(371.1744, 1145.48)(372.1729, 156.08)	84836.45	38960.74	103393.3	45698.6	16526.15	9852.475	26555.73	16378.29	-1.41	7.54E-12	2E-10	-0.25	0.193744	0.505089
COMPOUND_370_1808a7_689697	Rp	-	370.1808	7.69				(369.1736, 81939.73)(370.1768, 18554.74)(371.1749, 5743.57)(372.1761, 828.57)(739.3438, 219.94)(740.3426, 81.67)(741.3311, 85.49)	290450.1	158121	261097.9	139489	122167.8	52710.75	113066.1	102557.6	-0.95	7.18E-05	0.000228	-0.18	0.495275	0.751527
COMPOUND_370_1808a10_995727	Rp	-	370.1808	11.00				(775.3317, 378.41)(776.3485, 325.99)(777.3457, 250.66)(483.1661, 2614.65)(484.1628, 1556.13)(485.1632, 2493.17)(486.1601, 267.9)(487.1554, 1213.3)(405.1544, 3677.12)(406.1551, 1143.5)(407.1502, 1627.4)(408.1611, 601.29)(409.1537, 1195.77)(785.3667, 840.31)(786.3603, 563.78)(787.3821, 821.78)(369.1732, 7477.53)(370.1781, 3174.11)(371.1763, 1323.46)(372.188, 195.76)(739.3594, 326.71)(740.3762, 148.4)(741.3566, 246.16)(415.1777, 9742.01)(416.1807, 2079.0)(417.1784, 18548.08)(418.1833, 4074.03)(419.1734, 6733.81)(420.1769, 1778.36)(421.1691, 1370.53)(853.3595, 666.44)(854.3756, 432.13)(855.3484, 317.82)	146168.1	59668.79	146632	55388	160096.6	35066.3	151602.7	59222.01	0.53	0.082325	0.136743	-0.35	0.338594	0.642388
COMPOUND_370_1809a11_009078	Rp	-	370.1809	11.01				(775.3339, 373.68)(776.3391, 229.2)(777.3207, 90.3)(483.1673, 3050.98)(484.1624, 1684.23)(485.149, 1856.42)(486.1546, 503.91)(487.1492, 1717.34)(405.1535, 4226.26)(406.1557, 1184.91)(407.1489, 2771.95)(408.1521, 668.61)(409.1516, 1629.97)(785.3445, 1086.33)(786.3431, 547.34)(787.3411, 559.55)(369.1735, 7491.59)(370.1777, 3406.01)(371.1753, 1817.25)(372.1776, 249.58)(739.3278, 503.94)(740.3372, 410.11)(741.3472, 854.45)(415.1777, 11122.68)(416.1818, 2177.43)(417.1785, 21615.24)(418.1835, 5033.43)(419.1744, 8099.67)(420.177, 2130.62)(421.1732, 1555.68)(853.367, 618.83)(854.3825, 462.19)(855.3755, 839.33)	153225.6	42949.18	170997.1	53845.72	218894.5	88695.16	178357.2	51359.03	1.15	0.000177	0.000529	-0.93	0.008722	0.14883
COMPOUND_370_1809a8_225458	Rp	-	370.1809	8.23				(483.156, 892.06)(484.1548, 321.65)(485.156, 202.98)(405.1452, 191.25)(369.1738, 30616.29)(370.1768, 6588.29)(371.1747, 1792.25)(372.1748, 259.59)(739.3546, 4148.24)(740.3581, 1700.82)(741.3561, 827.82)(742.358, 176.87)	911180.1	603976.8	913132.7	579794	538050.6	369605.3	517494.5	461508.4	-0.62	0.003752	0.008719	-0.22	0.360698	0.666906
COMPOUND_370_1809a8_846407	Rp	-	370.1809	8.85				(369.1738, 42963.97)(370.177, 9358.95)(371.1849, 3585.33)(372.1756, 391.01)	93647.21	54770.41	105170.8	56444.53	123419.1	59392.55	84023.51	49773.25	0.56	0.022781	0.044113	-0.94	0.001212	0.056309
COMPOUND_370_181a8_184061	Rp	-	370.1810	8.18				(405.1576, 205.69)(483.1542, 936.24)(484.1572, 394.88)(485.1534, 201.41)(369.1738, 30616.29)(370.1768, 6588.29)(371.1747, 1792.25)(372.1748, 259.59)(739.3549, 3252.59)(740.3575, 1441.43)(741.3571, 644.08)(742.3623, 139.07)	482512.5	500263.9	323358.2	490818.8	143700.6	208198.7	280464.5	410490.3	-1.05	0.000611	0.001672	1.00	0.0056	0.118614
COMPOUND_370_2113a10_48221	Rp	-	370.2113	10.48				(369.2038, 10587.05)(370.2074, 2095.68)(371.2137, 452.36)(372.2084, 59.85)(739.4372, 697.89)(740.4237, 1408.31)	20747.23	9104.689	24803.28	14676.04	19137.75	7428.21	19523.5	8082.126	-0.54	0.033843	0.062626	-0.04	0.901497	0.955487
COMPOUND_370_2346a7_587578	Rp	-	370.2346	7.59				(405.1972, 1459.94)(406.201, 334.72)(407.2069, 5148.65)(408.2102, 1163.2)(369.2277, 16681.71)(370.2313, 3760.93)(371.2377, 914.15)(372.2414, 134.22)	97422.73	48039.02	108388.9	64554.81	85582.5	59088.1	57642.94	26544.34	-1.02	4E-05	0.000136	-0.17	0.532184	0.772216
COMPOUND_370_3081a11_103045	Rp	+	370.3081	11.10				(741.6005, 81.64)(779.5524, 2761.57)(393.2927, 5467.37)(394.2933, 1194.59)(395.2944, 187.74)(409.2794, 114.39)(410.2652, 907.23)(371.3069, 8001.81)(372.3096, 2601.89)(373.3028, 1339.3)(374.3059, 170.56)	47389.69	14820.81	43510.78	12494.55	69146.8	22347.71	70059.66	21825.53	-0.42	0.121496	0.192954	0.41	0.193636	0.505089
COMPOUND_371_2101a10_859324	Rp	+	371.2101	10.86				(743.4027, 2264.21)(744.3952, 1820.98)(745.3963, 1444.04)(765.4172, 2179.02)(766.4098, 1585.76)(410.1734, 9852.89)(411.1792, 2488.37)(412.1586, 926.41)(374.2127, 53617.54)	131998.4	24265.87	127313	27932.68	255848	57951.44	266920.9	52084.24	1.12	4.08E-06	1.77E-05	0.07	0.793501	0.913114
COMPOUND_371_2839a10_325151	Rp	+	371.2839	10.33				(781.5294, 1246.78)(394.2812, 844.32)(389.311, 694.75)(372.2906, 141129.3)(373.2938, 36617.66)(374.2969, 5667.38)(375.2997, 718.32)	118826.7	71978.83	78890.3	69359.52	64975.25	43018.77	53305.46	39448.19	-1.32	6.52E-09	5.56E-08	0.28	0.23193	0.537875
COMPOUND_371_3029a8_677095	Rp	+	371.3029	8.68	Myristoylcarnitine	1	10eV: 372.3108(100), 283.2021(11), 197.1189(9), 71.0879(8)	(394.2938, 2791.41)(372.3116, 10401.84)(373.3148, 2540.58)(374.3158, 642.42)(375.3325, 87.57)	47069	16998.22	42688.91	15183.15	41170.29	21445.49	50919.49	17613.53	-0.37	0.205825	0.296557	0.80	0.023073	0.215607
COMPOUND_372_0615a6_709951	Hilic	+	372.0615	6.71				(745.1332, 191.54)(395.064, 272.64)(767.0788, 141.75)(390.0944, 63.66)(373.0683, 7410.32)(374.0714, 1067.74)(375.0668, 3292.66)(376.0695, 807.44)	31466.77	6826.037	34503.61	7998.725	40289.93	7263.73	43075.95	8868.108	0.93	0.000668	0.00181	-0.02	0.958145	0.980661
COMPOUND_372_2273a10_686917	Rp	-	372.2273	10.69				(789.4774, 635.19)(371.2199, 14881.29)(372.2226, 3689.41)(373.2254, 712.42)(374.2225, 101.21)(743.4414, 1925.99)	75946.11	19596.95	89143.24	25406.9	63883.71	21070.39	70616.68	24099.28	-0.59	0.014847	0.030341	-0.19	0.50176	0.75374
COMPOUND_373_1242a10_579026	Rp	-	373.1242	10.58	no msms			(781.1948, 292.33)(486.1, 1405.57)(372.1161, 61956.9)(373.1185, 17579.65)(374.1142, 22771.04)(375.117, 5399.17)(376.1182, 1438.05)(745.2652, 332.51)(746.2641, 664.86)(859.2761, 342.96)(860.287, 578.16)	44977.6	25464.56	49841.29	27400.57	69732.13	25814.43	91250.17	49031.2	0.27	0.212488	0.304821	0.91	0.000793	0.045795
COMPOUND_373_2051a10_858647	Rp	+	373.2051	10.86				(747.4034, 8355.35)(785.3685, 1537.95)(786.3545, 2115.64)(787.3601, 1167.78)(769.4173, 14457.95)(770.4123, 4569.43)(374.2122, 53773.7)(375.2157, 11972.12)(376.2184, 1993.04)(764.4536, 1851.15)(765.4521, 1052.76)	133140.7	26193.3	123967.4	24255.14	250838.4	88043.75	238470.5	47719.26	1.53	7.96E-11	1.26E-09	0.11	0.644574	0.836645

									1st trimester				3rd trimester									
									Control		Pre-eclampsia		Control		Pre-eclampsia		Time			Interaction		
Compound	Column	Ion_mode	Mass	Retention_	Identification	ID_level	MSMS_spectra	CompositeSpectrum	Mean	SD	Mean	SD	Mean	SD	Mean	SD	Value	raw p	FDR_p	Value	raw p	FDR_p
COMPOUND_374_155a9_926652	Rp	+	374.1550	9.93				(749.3241, 490.65)(750.3135, 324.43)(751.3256, 98.33)(375.1623, 7830.94)(376.1659, 2141.68)(377.163, 8916.04)(378.1651, 2211.74)(379.159, 3703.49)(380.1614, 1140.49)(381.1623, 971.1)	41897.98	16072.15	44833.13	16985.5	39794.25	19809.52	46868	19782.68	-1.02	1.52E-05	5.83E-05	-0.16	0.530411	0.771657
COMPOUND_374_2088a8_513025	Rp	-	374.2088	8.51				(409.1779, 1673.12)(410.1647, 22.58)(373.2015, 10517.18)(374.2045, 2273.66)(419.2061, 1705.03)(420.2101, 452.14)	25143.91	8895.997	24428.16	10488.63	19788	9397.833	20491.67	7208.453	-1.30	6.27E-09	5.39E-08	0.06	0.781445	0.909465
COMPOUND_376_282a10_598473	Rp	-	376.2820	10.60				(375.2748, 12562.37)(376.2788, 2832.81)(377.2786, 744.69)(378.2787, 109.42)(421.2876, 345.15)	32275.23	11732.97	38471.02	14086.61	20347.69	7877.843	26202.89	10749.57	-1.06	0.000207	0.000613	0.20	0.539935	0.776458
COMPOUND_376_2821a10_541127	Rp	-	376.2821	10.54				(375.2746, 9198.58)(376.2785, 1974.94)(377.2789, 812.09)(378.2689, 102.6)(421.2973, 176.74)	17501	6271.961	20619.2	7027.135	15675.83	4761.182	16407.16	3827.597	-0.53	0.069492	0.118289	-0.06	0.86619	0.944172
COMPOUND_377_2031a10_93219	Rp	+	377.2031	10.93				(755.4004, 6096.55)(756.3913, 3568.4)(793.3695, 1595.29)(794.3718, 1444.56)(795.3547, 5072.46)(796.3471, 2034.65)(777.4115, 3655.03)(416.1628, 305.29)(378.21, 77984.02)(379.2131, 18987.94)(380.2107, 68449.18)(381.2143, 16042.37)(382.2066, 26306.85)(383.2095, 9597.82)(384.2078, 5318.0)(772.3564, 1132.04)(773.3673, 1316.35)(774.3935, 359.61)	265397.5	111993	264966.9	112979.8	478941.9	141888.7	582769	183244.2	1.06	8.29E-06	3.36E-05	0.43	0.103948	0.381877
COMPOUND_377_2572a9_505563	Rp	+	377.2572	9.51				(777.5297, 388.09)(378.2651, 18732.24)(379.2681, 5561.11)(380.2723, 1059.63)(381.262, 160.22)	74348.4	36527.41	75194.89	34359.53	73136.5	40326.05	93538	48334.25	-1.01	1.72E-05	6.56E-05	-0.17	0.518147	0.763656
COMPOUND_377_258a9_251448	Rp	+	377.2580	9.25				(378.2661, 200588.36)(379.2688, 48509.59)(380.2757, 11460.14)	206398.3	105216.3	207524.4	100286.4	207391.8	118772.9	243370.5	143958.2	-0.99	2.77E-05	9.96E-05	-0.18	0.491728	0.748114
COMPOUND_377_2929a9_299879	Rp	+	377.2929	9.30				(378.3001, 50116.64)(379.3039, 12914.79)(380.2835, 9658.69)	116829.9	40854.53	101968.1	38568.97	48183	23280.46	51004	21454.55	-1.49	1.1E-12	4.87E-11	0.14	0.486021	0.747178
COMPOUND_378_1465a10_50011	Rp	-	378.1465	10.50				(791.2838, 773.33)(491.1371, 2591.51)(492.1398, 1423.51)(493.1297, 3242.85)(494.1243, 948.63)(495.1282, 1178.35)(496.1364, 606.46)(497.1297, 492.02)(413.0988, 2814.46)(801.2774, 414.39)(802.292, 260.91)(803.2803, 795.94)(377.1336, 4498.06)(378.1388, 1202.18)(379.1288, 539.54)(380.117, 76.75)(755.2841, 810.05)(756.2796, 654.99)(757.2634, 673.29)(423.1469, 6936.83)(424.1476, 1755.85)(425.1467, 12138.69)(426.1505, 2670.52)(427.1431, 4090.0)(428.1455, 1415.65)(429.1416, 970.24)(869.2781, 348.45)(870.2788, 270.44)(871.2641, 369.79)	131118.1	52531.1	143905.9	59050.81	163781	53077.11	189990.9	59910.9	0.30	0.264734	0.361761	0.21	0.500683	0.753664
COMPOUND_378_1938a9_921415	Rp	-	378.1938	9.92				(377.1865, 6909.99)(378.1894, 1415.49)(379.1868, 2342.62)(380.1876, 606.24)(381.1993, 79.33)(755.3501, 247.99)(756.3562, 195.19)	16082.2	6301.635	17731.08	5331.25	17833.5	236.8808	19234	4800.668	-0.94	4.65E-05	0.000155	-0.16	0.52231	0.765284
COMPOUND_378_2397a8_97858	Rp	-	378.2397	8.98				(413.2076, 1258.25)(414.2119, 466.49)(415.1953, 973.36)(416.1885, 82.25)(417.1811, 1121.09)(377.2327, 21186.54)(378.2359, 5453.32)(379.2201, 3018.27)(380.216, 462.74)(755.4569, 54.17)(423.2381, 2474.94)(424.2409, 676.11)	34366.3	21421.75	38747.56	21833.19	56824	13040.94	70271.9	26900.73	-0.90	0.000275	0.000794	-0.14	0.619017	0.817823
COMPOUND_378_2774a10_447278	Rp	+	378.2774	10.45				(795.5212, 2803.14)(401.2766, 12881.11)(402.2796, 3978.07)(403.2861, 2414.99)(417.24, 1323.51)(418.2473, 607.26)(419.2358, 248.81)(396.3128, 85309.33)(397.3155, 21644.83)(398.3195, 3752.3)(379.284, 12176.12)(380.2853, 3806.79)(381.2884, 1333.58)(382.2869, 189.45)(774.5746, 496.05)(775.5868, 173.3)(776.5517, 1534.34)	106328.8	58453.75	120333.6	122483.6	98136.92	55316.22	170162.7	93384.76	0.07	0.773955	0.833547	0.72	0.020102	0.199116
COMPOUND_378_2925a10_860721	Rp	+	378.2925	10.86				(795.5388, 21262.57)(379.2956, 8735.38)(380.3016, 2420.43)(381.2989, 42268.68)(382.3018, 9692.21)	86278.7	39238.88	74955.83	36999.67	94752.9	46046.77	93385.45	47789.32	0.28	0.279518	0.379202	-0.10	0.741084	0.888965
COMPOUND_379_2187a11_189893	Rp	+	379.2187	11.19				(759.4155, 1667.31)(797.3736, 4870.98)(798.363, 1208.37)(799.3926, 5055.43)(418.1813, 399.31)(380.2257, 82343.21)(381.2285, 19925.35)(382.2262, 35955.13)(383.2291, 9027.3)(384.2216, 15115.97)(385.224, 4168.74)(386.2212, 2194.37)(776.4692, 696.24)(777.4716, 615.37)(778.4787, 152.52)	247264.8	50334.47	225822.5	51837.61	516646.2	173232.3	512284.3	149477.2	1.26	4.25E-07	2.37E-06	0.22	0.401307	0.690408
COMPOUND_379_2723a9_27655	Rp	+	379.2723	9.28				(380.2805, 27882.69)(381.2835, 6664.5)(382.2911, 1996.5)	53461.77	23179.89	45561.49	18184.66	66092.33	21889.73	47643.83	9648.144	-1.17	9.32E-08	5.88E-07	0.06	0.786035	0.909997
COMPOUND_380_378a11_01279	Rp	+	380.3780	11.01				(761.7973, 1670.6)(799.7292, 2605.38)(800.7174, 1925.64)(403.3787, 6057.42)(783.7488, 2734.44)(784.7294, 534.28)(785.7499, 2610.2)(381.3846, 100125.17)(382.3875, 27101.95)(383.3928, 4308.76)(384.3965, 565.79)(778.7644, 950.73)	86710.07	36686	91328.74	40991.57	173480.8	115852.3	184249	160997.4	1.06	0.000155	0.000466	-0.28	0.382371	0.685589
COMPOUND_382_1098a6_195838	Hilic	+	382.1098	6.20				(383.1167, 10591.75)(384.1208, 1397.02)(385.1187, 176.59)	7840.531	3125.66	6833.784	3378.778	9310.769	2106.677	10123.8	4702.338	0.39	0.142692	0.219047	0.55	0.085236	0.346484
COMPOUND_382_1615a10_667491	Rp	+	382.1615	10.67				(765.353, 838.85)(383.1677, 31722.55)(384.1705, 7959.84)(385.1688, 17956.05)(386.1721, 4130.82)(387.1648, 6792.01)(388.1672, 1650.99)(389.1632, 883.23)(782.3612, 859.41)	47576.15	16479.23	47937.35	19105.54	68336.29	27626.3	82394.84	38842.85	0.76	0.002877	0.006877	0.34	0.238609	0.543832
COMPOUND_382_182a10_312435	Rp	+	382.1820	10.31	no msms			(405.1598, 726.95)(421.1638, 1389.22)(383.1884, 14999.54)(384.1917, 3844.52)(385.1893, 6336.07)(386.1928, 1619.36)(387.1851, 3015.4)(388.1866, 695.02)	12076.63	2125.312	16702.4	6333.529	19387	5676.229	31238.69	19188.14	0.32	0.074901	0.125878	0.85	0.000189	0.018868
COMPOUND_382_2054a10_859109	Rp	+	382.2054	10.86				(765.4067, 3012.73)(803.345, 1888.33)(787.4163, 3693.73)(788.4213, 3996.25)(421.1788, 792.72)(400.2242, 1690.1)(383.2126, 61485.65)(384.2163, 16752.33)(385.2214, 3147.93)(782.418, 2786.44)	150378.2	37426.69	137980.8	34614.5	250271.3	48672.64	277472.8	61868.7	1.15	5.26E-07	2.81E-06	0.52	0.035269	0.250187
COMPOUND_382_2122a9_251608	Rp	+	382.2122	9.25				(383.2208, 9424.69)(384.2242, 2521.73)(385.2347, 787.28)(386.2223, 114.19)	51004.57	22619.75	56499.35	25401.13	54704.25	29942.41	75066.7	31885.56	-0.91	0.000139	0.000422	-0.26	0.330998	0.636557
COMPOUND_382_324a10_396373	Rp	+	382.3240	10.40				(383.3318, 9150.22)(384.3351, 2761.41)(385.3375, 621.95)	14658.76	2585.681	13087.87	2571.217	19555.64	7435.435	18298.6	5452.75	1.09	1.35E-05	5.25E-05	-0.20	0.461105	0.729797
COMPOUND_382_3934a11_232425	Rp	+	382.3934	11.23				(765.754, 1247.91)(766.7343, 656.96)(787.7518, 1345.26)(383.4005, 41244.66)(384.4034, 11019.19)(385.4068, 1825.2)(386.4019, 220.26)	351609.3	335382.7	283506.2	237286.3	869156.1	590360.9	1147434	1061041	1.03	9.13E-05	0.000284	0.22	0.45469	0.725699
COMPOUND_383_1524a10_576748	Rp	-	383.1524	10.58				(801.2459, 353.27)(802.258, 566.55)(803.2735, 491.22)(811.3231, 732.65)(812.3284, 426.18)(382.1448, 38867.64)(383.1474, 12789.82)(384.1497, 2980.16)(385.1474, 124.27)(765.2843, 454.76)(766.3046, 1534.02)(767.297, 1299.8)(879.3069, 453.84)	183532.7	76179	199255.8	88403.68	214685	75312.17	254104.5	70800.73	-0.05	0.848256	0.894514	0.65	0.054162	0.289719
COMPOUND_383_3399a10_777776	Rp	+	383.3399	10.78				(767.7037, 1058.59)(406.3286, 6237.85)(407.3314, 1702.96)(422.2987, 976.3)(423.3047, 533.65)(384.3477, 11602.22)(385.3507, 3252.5)(386.3401, 189.87)(784.726, 1324.59)(785.7288, 1219.56)	83765.26	19352.27	77950.8	21722.75	105442.6	33350.31	118967.8	19314.81	0.70	0.00899	0.019226	0.40	0.196835	0.507758
COMPOUND_384_1601a5_617394	Rp	-	384.1601	5.62				(383.1529, 14388.69)(384.1565, 3483.78)	18035.67	11693.39	18655.2	9784.407	40936.31	12567.61	30713.35	14319.51	1.52	3.1E-11	6.3E-10	-0.72	0.001632	0.06002
COMPOUND_384_1604a6_282825	Rp	-	384.1604	6.28				(383.1527, 44977.66)(384.1559, 10121.08)(385.1539, 2928.43)(386.1546, 437.55)(387.153, 32.0)(767.3124, 1501.4)(768.317, 721.72)(769.316, 327.62)	181923.5	257969.2	172255.9	171457	541721.7	214533.2	370133.9	239933.1	1.55	9.76E-09	8.14E-08	-0.73	0.009235	0.153413
COMPOUND_384_1759a10_784266	Rp	+	384.1759	10.78				(769.3392, 1583.23)(770.3633, 1083.64)(807.3449, 1596.07)(791.3563, 1034.14)(423.1527, 176.79)(402.2216, 1071.06)(385.1822, 14920.04)(386.1869, 3505.01)(387.183, 6945.3)(388.186, 1702.57)(389.18, 2406.77)(390.1824, 1084.35)(391.1635, 784.9)	28251.8	17763.11	28049.53	15558.99	24331.5	10224.39	38132.21	27354.59	0.02	0.944912	0.964615	0.83	0.002216	0.067469
COMPOUND_384_3401a12_557961	Rp	+	384.3401	12.56				(407.3289, 6672.67)(408.3328, 1934.9)(409.3376, 394.69)(423.3018, 1025.33)(424.3065, 157.57)(385.3475, 20350.11)(386.3521, 5645.93)(387.3563, 1180.36)(388.3527, 179.36)	75600.56	21221.81	65075.67	20079.2	194089.6	38875.11	177605	64531.89	1.48	5.8E-10	6.93E-09	0.17	0.486727	0.747178
COMPOUND_384_3405a11_051774	Rp	+	384.3405	11.05				(769.72, 1637.11)(770.7134, 1990.92)(771.6936, 432.1)(402.3767, 16478.32)(403.3787, 5186.42)(404.3733, 1837.38)(385.3473, 31149.26)(386.353, 8770.79)(387.364, 1694.14)	141217.1	53907.97	119588.4	44943.28	113467.6	58746.67	147543.6	144625.1	-0.96	0.000904	0.002384	0.63	0.057963	0.298567
COMPOUND_384_3407a11_521464	Rp	+	384.3407	11.52				(791.6922, 1405.94)(385.3476, 38259.66)(386.3524, 11185.64)(387.3533, 1338.98)(786.7284, 1826.01)	72745.6	23245.33	72061.98	23606.04	63673.5	12418.73	56670.67	20805.15	-1.16	2.73E-07	1.57E-06	-0.11	0.645837	0.836838
COMPOUND_384_4081a11_554472	Rp	+	384.4081	11.55				(807.742, 672.61)(385.4163, 23145.84)(386.418, 6386.07)(387.4193, 864.92)(388.4182, 90.42)(786.8912, 1051.65)(787.8705, 876.09)	39387.98	22042.35	42600.36	23360.01	55807	53940.25	58206.13	55260.46	0.36	0.221294	0.314618	-0.13	0.718486	0.876415
COMPOUND_385_0998a10_467474	Rp	-	385.0998	10.47				(498.0724, 932.05)(815.2061, 461.04)(816.2038, 634.17)(817.2088, 500.24)(384.0918, 8032.6)(385.0963, 1524.98)(386.089, 5576.61)(387.0942, 982.23)(388.0856, 1048.35)(389.0938, 199.46)(430.0916, 555.88)	20498.67	7111.427	20329.79	6962.297	35230.5	6048.813	37434.43	7289.99	1.33	1.49E-08	1.18E-07	0.10	0.669208	0.846185

									1st trimester				3rd trimester									
									Control		Pre-eclampsia		Control		Pre-eclampsia		Time			Interaction		
Compound	Column	Ion_mode	Mass	Retention_	Identification	ID_level	MSMS_spectra	CompositeSpectrum	Mean	SD	Mean	SD	Mean	SD	Mean	SD	Value	raw p	FDR_p	Value	raw p	FDR_p
COMPOUND_385_1673a10_868931	Rp	-	385.1673	10.87				(805.335, 1344.03)(384.1606, 100249.88)(385.1655, 31212.05)(386.1646, 7995.63)(387.1673, 1174.14)(769.3231, 1809.42)(770.3383, 1199.81)(771.3452, 1154.3)(883.3443, 747.97)	642139.5	69252.43	614268.4	72882.92	494991.3	162513.4	505132.6	105494.4	-1.44	4.94E-08	3.4E-07	0.37	0.183835	0.497593
COMPOUND_385_3199a10_459403	Rp	+	385.3199	10.46				(403.3541, 17754.96)(404.3567, 6803.23)(386.3272, 11257.54)(387.3248, 4131.02)(388.3302, 189.53)	56222.95	28204.55	42550.16	20641.11	64252.67	20376.43	101929.5	55430.48	0.32	0.214051	0.306643	0.63	0.037529	0.259604
COMPOUND_386_1706a7_9313602	Rp	+	386.1706	7.93				(409.1628, 3140.6)(410.1684, 814.88)(411.1717, 218.01)(404.2077, 50154.91)(405.2108, 12894.66)(387.1812, 71342.19)(388.1843, 17480.46)(389.1886, 3839.24)	124098.1	125022.5	72445.42	88761.32	60986	26048.11	48761.57	43092.16	-1.23	1.08E-06	5.33E-06	0.42	0.127718	0.424221
COMPOUND_386_1743a8_2173395	Rp	+	386.1743	8.22				(409.1651, 1401.51)(410.1627, 412.14)(411.1735, 130.57)(404.2086, 435.9)(405.2192, 232.75)(387.1817, 8364.5)(388.1853, 1797.99)(389.1926, 1213.37)	25620.02	11284.57	23013.56	10018.04	10337	#DIV/0!	13825.15	3610.265	-1.37	7.73E-10	8.93E-09	0.16	0.471118	0.734155
COMPOUND_386_1749a7_864254	Rp	+	386.1749	7.86				(409.162, 3794.5)(410.1653, 995.6)(411.1694, 214.41)(425.1363, 972.18)(426.1396, 196.99)(427.1318, 174.51)(404.2075, 16760.91)(405.2107, 4535.62)(406.2152, 885.28)(407.2123, 130.28)(387.1812, 71342.19)(388.1843, 17480.46)(389.1886, 3839.24)(390.191, 516.63)	231740.6	121617.8	199405.2	58936.45	43424.79	20240.69	51031.58	28797.09	-1.77	1.32E-12	5.62E-11	0.40	0.096451	0.366905
COMPOUND_386_1755a6_473585	Rp	-	386.1755	6.47				(385.1683, 6262.82)(386.1718, 1399.13)(387.1693, 613.43)(388.169, 68.38)	17159.05	6476.573	17023.43	5268.542	26232.31	10544.05	25202.11	18344.26	1.16	9.05E-07	4.54E-06	-0.23	0.37013	0.671661
COMPOUND_386_1755a5_6170444	Rp	-	386.1755	5.62				(385.1683, 20628.79)(386.1721, 4523.12)(387.1698, 1437.56)(388.1732, 217.97)	51040.15	21429.29	48735.87	15888.48	79661.07	54382.13	59109	44415.25	0.46	0.129254	0.20256	-0.50	0.170552	0.485567
COMPOUND_386_1756a7_296122	Rp	-	386.1756	7.30				(385.1684, 13197.5)(386.1714, 3064.78)(387.1747, 1207.7)(388.1808, 144.76)	24541.52	11408.51	24006.68	11677.58	57356.57	23443.14	47265.76	26738.99	1.51	3.44E-10	4.42E-09	-0.54	0.024998	0.219303
COMPOUND_386_1756a5_6674857	Rp	-	386.1756	5.67				(385.1686, 34036.33)(386.1718, 7196.43)(387.1696, 2200.47)(388.1702, 329.46)	58891.49	36097.24	55044.2	26589.45	138773.4	61236	104818.4	64203.22	1.32	3.61E-06	1.58E-05	-0.50	0.109048	0.391669
COMPOUND_386_1878a8_722836	Rp	+	386.1878	8.72				(409.1687, 1003.14)(410.1697, 428.28)(411.1691, 374.72)(425.1518, 272.75)(426.1458, 116.98)(387.1939, 6318.58)(388.1989, 1404.22)(389.1972, 678.11)	22611.27	4521.374	21098.2	6236.652	21481.71	6389.985	20522.98	4377.833	-0.03	0.923778	0.950653	0.06	0.864182	0.943353
COMPOUND_387_1618a9_927632	Rp	+	387.1618	9.93				(775.3248, 350.69)(776.3316, 259.0)(777.3111, 450.8)(813.2828, 212.58)(410.141, 362.63)(426.1093, 505.72)(388.1693, 6239.36)(389.1724, 2183.64)(390.1623, 71.14)	17945.94	5710.612	18979.23	5899.716	20149	1013.468	22399.38	5960.526	-1.00	2.6E-05	9.41E-05	-0.02	0.94411	0.974642
COMPOUND_387_2201a11_016309	Rp	+	387.2201	11.02				(813.3963, 1102.48)(426.1719, 469.25)(388.2286, 7689.77)(389.2306, 2124.01)(390.2188, 272.41)	22942.92	6167.906	21631.08	5708.548	33947.83	8421.467	31020.63	9169.111	0.74	0.00748	0.016338	0.10	0.753439	0.895058
COMPOUND_387_298a0_86685723	Hilic	+	387.2980	0.87				(410.2912, 879.03)(411.2931, 332.76)(412.3041, 1998.15)(797.5844, 327.85)(798.5887, 529.61)(405.3304, 987.13)(406.3405, 265.87)(388.3064, 11198.64)(389.3095, 2343.02)(390.3104, 584.32)	24670.76	9564.171	26868.81	8141.631	17269.92	7099.418	21673.8	9370.668	-0.43	0.122961	0.194457	0.03	0.939042	0.97184
COMPOUND_387_3392a10_695449	Rp	+	387.3392	10.70	no msms			(775.6829, 1593.89)(776.6869, 995.01)(777.6592, 738.69)(410.316, 3609.51)(426.2906, 1012.44)(388.3468, 26433.55)(389.3511, 6993.47)(390.3494, 1418.95)	28836.85	18540.75	23233.91	9595.818	26997.56	7839.107	52698.11	33069.44	0.18	0.367564	0.469863	1.12	1.93E-05	0.005768
COMPOUND_388_1912a6_901253	Rp	-	388.1912	6.90				(387.1842, 15891.48)(388.1876, 3318.07)(389.1844, 1338.96)(390.1862, 207.74)	28688.11	16267.13	29555.22	20055.4	69457.5	31273.67	57350.28	34559.55	1.23	1.07E-08	8.73E-08	-0.33	0.125664	0.423013
COMPOUND_388_222a9_917744	Rp	-	388.2220	9.92				(811.3723, 765.89)(812.3618, 238.78)(813.3624, 184.88)(387.2147, 29127.75)(388.2183, 6660.35)(389.2228, 1476.04)(390.2309, 228.27)(775.4201, 590.92)(776.4247, 404.86)(777.4269, 273.43)	66366.66	24892.88	74988.5	25421.89	60463.5	28805.16	61274.93	35157.31	-0.99	1.93E-05	7.24E-05	-0.32	0.214103	0.521709
COMPOUND_389_0504a10_468301	Rp	-	389.0504	10.47				(502.0273, 347.72)(388.0424, 6739.26)(389.0456, 1163.06)(390.0399, 6132.37)(391.0422, 1216.86)(392.0381, 1629.94)(393.0393, 469.62)(394.0357, 360.96)	12579.65	2468.974	12756	1670.589	34310.93	9061.815	35200.67	10354.62	1.42	2.98E-14	4.19E-12	0.14	0.403246	0.691103
COMPOUND_389_1546a11_008361	Rp	-	389.1546	11.01				(813.2873, 279.49)(814.2831, 200.62)(823.3465, 451.65)(388.1471, 8416.09)(389.1486, 2507.41)(390.1457, 2983.78)(391.1455, 1360.72)(392.1404, 374.27)(434.1755, 708.82)	17159.78	4952.057	17489.76	5362.747	25178.57	9688.534	24935.79	10015.73	1.09	8.72E-05	0.000272	-0.22	0.476608	0.739711
COMPOUND_389_1785a10_689982	Rp	+	389.1785	10.69				(779.3549, 1218.36)(817.3465, 511.56)(818.3201, 767.26)(801.3606, 1191.17)(390.1858, 12629.55)(391.1889, 3149.91)(392.19, 724.38)(393.1829, 70.28)(796.3904, 852.36)(797.3911, 526.87)(798.3749, 1098.57)	36227.38	9746.806	38683.85	11836.77	48193.85	21557.93	46939.73	14596.47	0.71	0.010122	0.021443	-0.32	0.312026	0.618015
COMPOUND_390_2523a10_461271	Rp	-	390.2523	10.46				(389.246, 26606.49)(390.2495, 5401.29)(391.2443, 8015.63)(392.2477, 2164.92)(393.2525, 487.1)(893.4742, 945.19)	25342.96	11319.43	32555.86	30864.46	34266.57	8927.378	44743.38	23568.89	-0.21	0.420184	0.52424	0.89	0.005531	0.118614
COMPOUND_390_2786a11_066194	Rp	+	390.2786	11.07	Dioctyl phthalate	1	10eV: 149.0246(100), 71.0866(65), 391.2837(6), 300.2127(6)	(413.2669, 15431.88)(414.2707, 4545.84)(415.273, 1040.81)(416.2818, 159.86)(803.5612, 21886.83)(429.2415, 2753.92)(430.2474, 699.25)(431.2422, 604.36)(408.3095, 1994.32)(391.2861, 244614.92)(392.289, 62437.43)(393.2925, 10804.38)(394.297, 1621.18)	382561.1	101590.8	342829.3	103657.6	494518.7	86099.23	509875.6	126687.4	0.90	0.000668	0.00181	0.39	0.199395	0.510852
COMPOUND_391_1863a6_4711413	Rp	+	391.1863	6.47				(392.1933, 7559.01)(393.1974, 1632.21)(394.1955, 405.32)(395.2015, 65.59)	27016.14	6999.314	26030.21	5389.238	24530.75	7283.514	27698.06	6607.932	-0.14	0.623392	0.714806	0.38	0.277939	0.585315
COMPOUND_392_2134a10_858345	Rp	+	392.2134	10.86				(785.4129, 6182.08)(823.3888, 221.5)(824.3764, 2089.5)(825.3764, 1003.0)(807.4295, 4304.0)(808.428, 2797.96)(431.1728, 724.09)(432.1761, 369.37)(433.1675, 343.78)(393.221, 47912.39)(394.2241, 11897.58)(395.2218, 64421.94)(396.2247, 15254.26)(397.2181, 24621.74)(398.2204, 6819.95)(399.2188, 4486.6)(400.2214, 1607.6)(802.4344, 6098.8)	331555.4	58716.53	328028.7	55442.78	507687	104647.9	518643.2	91305.72	1.24	4.03E-07	2.25E-06	0.23	0.38686	0.68827
COMPOUND_392_2684a10_663915	Rp	-	392.2684	10.66				(819.4982, 1213.6)(505.2591, 7566.41)(506.2584, 2181.31)(427.2334, 6716.1)(428.2355, 2717.74)(429.235, 1164.25)(391.2614, 53506.73)(392.2646, 12973.89)(393.2592, 18344.23)(394.2618, 4082.74)(395.2653, 298.9)(897.5324, 7865.03)	60054.76	43015.47	58141.91	53006.3	49661.83	38467.87	71650.09	53552.61	-0.39	0.185627	0.273763	0.85	0.017643	0.194764
COMPOUND_392_2687a10_679073	Rp	-	392.2687	10.68				(819.4992, 1268.09)(505.2589, 7016.16)(506.2585, 1898.61)(427.233, 5485.76)(428.236, 2289.27)(429.2351, 871.65)(391.2614, 31052.39)(392.265, 7504.9)(393.2595, 11677.25)(394.262, 2751.81)(395.263, 261.74)(897.5348, 7163.79)	127663.8	73078.6	117664.7	67825.1	46036.44	26016.66	71113.38	58838.36	-1.41	7.7E-07	3.94E-06	0.76	0.014408	0.179521
COMPOUND_392_2919a9_723376	Rp	-	392.2919	9.72	Deoxycholic acid or Chenodeoxycholi	3	10 eV: 391.2849(100), 345.9474(18), 250.8475(13)	(427.2613, 18713.16)(428.2645, 5249.25)(429.2596, 6336.83)(430.2617, 1813.09)(431.2646, 289.06)(391.2851, 73106.61)(392.2885, 18248.94)(393.2913, 2903.4)(394.294, 392.43)(395.2786, 45.44)(783.5769, 13154.33)(784.5798, 7056.26)(785.5824, 2315.24)(786.5847, 562.27)(437.2905, 26327.6)(438.2937, 6603.56)(439.2959, 1277.73)	100569.4	92104.56	119699.3	189750.7	70549.92	101710.6	118604.9	115114	-0.49	0.050228	0.08789	0.48	0.100196	0.372347
COMPOUND_392_292a9_117318	Rp	-	392.2920	9.12				(505.2707, 473.49)(427.2588, 1121.26)(428.2636, 193.07)(429.2613, 411.83)(391.2847, 13153.65)(392.2879, 3668.99)(393.2896, 665.09)(783.5787, 341.66)(784.574, 139.54)(437.2903, 19254.2)(438.2936, 5194.18)(439.2967, 838.83)	27097.67	21589.5	29856.68	22761.88	38922.5	39153.21	19190.5	7119.272	-0.68	0.002855	0.006839	-0.13	0.608586	0.812181
COMPOUND_393_2523a8_269238	Rp	+	393.2523	8.27				(394.2597, 33789.83)(395.2626, 8494.94)(396.2724, 3243.12)(397.2732, 422.93)	152173.9	70955.11	144443.7	63370.42	139818	3719.63	126130.3	60931.17	-1.26	2.44E-08	1.83E-07	-0.03	0.900134	0.955487
COMPOUND_393_2525a8_486106	Rp	+	393.2525	8.49				(394.2602, 13688.92)(395.2634, 3311.94)(396.2728, 1729.74)(397.276, 252.68)	76549.02	37584.49	71738.68	34917.5	67992	6437.8	68849.22	26213.23	-1.13	8.39E-07	4.24E-06	-0.07	0.782587	0.909997
COMPOUND_393_2552a11_232046	Rp	+	393.2552	11.23				(825.4686, 1030.03)(826.4629, 499.77)(827.4622, 663.83)(416.2515, 1290.6)(809.4943, 828.03)(411.2902, 685.6)(394.2618, 19314.84)(395.2657, 5038.08)(396.2576, 7129.24)(397.2582, 2535.31)(398.2539, 1919.74)	28453.45	12023.39	33669.13	13967.35	52694.75	33908.01	52719.32	22764.78	0.64	0.023563	0.045487	-0.58	0.080086	0.339484
COMPOUND_393_2874a9_277704	Rp	+	393.2874	9.28				(394.2957, 12427.04)(395.2985, 3307.82)(396.3015, 911.98)	27401.69	10944.63	27541.98	16361.43	32785.67	17445.02	26169.43	13302.66	-1.11	3.82E-07	2.15E-06	-0.18	0.435602	0.712919
COMPOUND_394_2349a8_000831	Rp	-	394.2349	8.00				(393.2279, 3892.15)(394.2304, 1108.93)(395.2366, 347.43)(396.2405, 46.54)(439.2251, 635.93)(440.2356, 148.59)	32561.22	28448.98	39197.74	29420.13	99222.33	30065.31	103009.1	35255	-0.50	0.042862	0.076425	-0.31	0.292947	0.602481
COMPOUND_394_2472a11_216245	Rp	-	394.2472	11.22				(393.2397, 13368.62)(394.2427, 2260.07)(395.2464, 724.41)(396.2451, 103.74)(787.4602, 3207.5)(901.5157, 938.23)	91718.23	12322.42	94909.7	15121.65	74605	19723.21	72457.43	18663.71	-0.99	1.1E-05	4.36E-05	-0.49	0.048656	0.279822
COMPOUND_394_2833a10_927651	Rp	-	394.2833	10.93				(823.5289, 2928.91)(824.5276, 3483.25)(825.5261, 1814.14)(393.2772, 43818.92)(394.2805, 8673.21)(395.275, 14430.09)(396.278, 3357.82)(397.2808, 455.11)(398.2785, 147.0)(901.5478, 631.64)(902.5502, 505.09)	100760.2	51148.07	108006.6	48068.56	37353.23	20217.88	43042.09	24932.68	-1.39	1.99E-07	1.17E-06	0.16	0.568831	0.788601
COMPOUND_395_1284a10_473548	Rp	-	395.1284	10.47				(825.2125, 577.39)(826.2114, 612.33)(508.0994, 691.35)(430.0923, 339.25)(835.2273, 470.73)(394.1227, 4335.32)(395.1234, 1015.72)(396.1164, 7546.66)(397.1202, 1926.34)(398.1146, 2588.2)(399.1268, 1141.03)(789.2739, 585.79)(790.28, 942.65)(791.286, 546.07)(440.1232, 753.26)	61533.3	20401.11	62305.93	22842.48	66061.36	17062.26	72587.26	21621.83	0.28	0.335484	0.436459	0.12	0.73513	0.888627

									1st trimester				3rd trimester				Time			Interaction		
									Control		Pre-eclampsia		Control		Pre-eclampsia							
Compound	Column	Ion_mode	Mass	Retention_	Identification	ID_level	MSMS_spectra	CompositeSpectrum	Mean	SD	Mean	SD	Mean	SD	Mean	SD	Value	raw p	FDR_p	Value	raw p	FDR_p
COMPOUND_395_1556a1_2353802	Hilic	+	395.1556	1.24				(418.1462, 336.68)(813.3099, 83.12)(396.1632, 4844.23)(397.1664, 866.81)(398.168, 105.93)	12572.66	1914.18	12427.43	2254.02	18221.93	2741.019	16941.23	3912.285	1.41	5.07E-07	2.73E-06	-0.31	0.298424	0.60748
COMPOUND_395_187a10_686862	Rp	-	395.1870	10.69				(825.3766, 734.93)(432.1606, 27067.83)(835.3424, 635.98)(836.3493, 575.67)(837.344, 625.99)(394.1814, 5265.3)(395.1829, 1587.66)(396.1833, 506.25)(903.3247, 469.12)(904.3465, 423.58)	137612.9	16439.48	138501	24609.81	118175.4	41568.61	115186.5	31931.4	-0.74	0.003183	0.00753	-0.28	0.325093	0.632765
COMPOUND_395_2218a10_000543	Rp	+	395.2218	10.00				(396.2308, 13398.48)(397.2339, 3638.85)(398.2289, 5168.3)(399.2332, 1275.65)(400.2391, 354.27)(808.4717, 1417.35)(809.4604, 769.94)	41958.12	15534.23	38076.62	12925.54	31773	4760.846	33468.25	11828.97	-1.26	2.55E-08	1.88E-07	0.10	0.666092	0.845092
COMPOUND_396_1699a6_5587883	Rp	-	396.1699	6.56				(395.1628, 12616.18)(396.1662, 2926.81)(397.1606, 4173.73)(398.1626, 982.1)(399.165, 192.73)	16829.27	4331.436	18032.44	4529.683	32404	6834.184	35274.36	11439.02	1.27	3.77E-06	1.64E-05	-0.27	0.365652	0.669574
COMPOUND_396_1967a8_501419	Rp	-	396.1967	8.50	steroid sulfate	3	10 eV: 395.1874 (100), 96.9594 (25)	(509.168, 465.53)(395.1894, 34890.1)(396.1928, 8550.42)(397.1938, 3006.78)(398.1961, 458.97)	165602.4	94476.1	193519.9	150129.3	304681	118993.6	188203.1	101332.5	1.30	7.88E-06	3.21E-05	-1.34	6.74E-05	0.010116
COMPOUND_396_2179a11_01659	Rp	+	396.2179	11.02				(793.4441, 336.89)(831.4018, 3865.22)(832.3984, 1048.51)(815.424, 1074.29)(397.2279, 8604.16)(398.232, 2707.32)(399.2372, 650.71)	42031.05	12397.44	37312.21	9950.868	55784.08	34860.52	57551.6	20788.7	0.57	0.031669	0.059136	0.34	0.270725	0.579536
COMPOUND_396_2201a11_018905	Rp	+	396.2201	11.02				(793.4243, 351.33)(831.3905, 1975.22)(832.4076, 799.49)(815.4231, 981.5)(397.2288, 7288.21)(398.2333, 2757.4)(399.2389, 719.31)	38686.84	9992.376	35815.95	8254.826	44830.46	14768.18	49212.08	14166.06	0.69	0.006252	0.013844	0.34	0.249199	0.558959
COMPOUND_396_2269a10_665085	Rp	-	396.2269	10.67				(827.4016, 997.84)(395.2197, 30907.36)(396.223, 7504.79)(397.2266, 1332.83)(398.2271, 123.94)(791.4443, 1416.21)(792.4428, 1262.19)(793.4432, 611.56)	66087.11	17829.53	76138.61	30669.24	65232.21	26489.25	81254.39	31850.23	-0.19	0.478577	0.581628	0.39	0.208994	0.518913
COMPOUND_397_1213a10_497002	Rp	-	397.1213	10.50				(510.105, 943.69)(839.2209, 525.25)(840.2096, 493.6)(841.22, 423.2)(396.1164, 11989.19)(397.1203, 3465.99)(398.1146, 4070.99)(399.1178, 988.15)(400.1148, 291.42)(793.1998, 593.58)(794.2106, 528.37)(907.2064, 351.63)(908.2108, 291.09)	44947	12716.25	50838	13063.43	52951.2	11846.56	62937.45	20142.59	0.49	0.040544	0.073162	0.30	0.284409	0.593016
COMPOUND_397_2053a10_917028	Rp	-	397.2053	10.92				(829.4048, 794.18)(432.181, 33369.58)(433.1837, 8655.95)(434.1815, 4062.16)(839.3774, 525.06)(396.1986, 6814.79)(397.2, 2833.87)(398.2016, 434.13)(793.3661, 1357.64)(794.3793, 903.82)(907.4035, 290.07)(908.3953, 245.35)(909.3908, 369.64)	115897	79768.53	91216.26	50077.41	122615.3	82257.99	94015.4	53599.67	-0.72	0.0025	0.006086	-0.09	0.755385	0.895058
COMPOUND_397_3199a8_86249	Rp	+	397.3199	8.86	ACar 16:1	2	10eV: 398.3277(100), 85.0287(6), 237.2237(4)	(420.3024, 1819.21)(421.3057, 376.56)(422.2914, 3252.86)(423.3053, 1547.02)(398.3276, 9511.78)(399.3313, 2604.08)(400.331, 468.5)(401.3415, 73.95)	54508.08	24006.26	49437.89	19748.34	55194.57	29596.69	64438.79	26578.52	-0.01	0.966729	0.980664	0.53	0.138817	0.444794
COMPOUND_398_2119a9_107574	Rp	-	398.2119	9.11	Unknown	4	10 eV: 397.2047 (100), 243.9077 (5)	(511.1857, 1467.0)(512.1866, 537.88)(513.1862, 434.17)(397.2046, 763105.3)(398.2085, 164885.31)(399.2154, 88588.8)(795.416, 1786.83)(796.4203, 960.17)(797.429, 1221.94)(798.4328, 584.69)(799.4456, 630.36)(800.4473, 305.59)(801.4483, 107.76)	342839.5	223399.3	320956.5	341254.5	6039262	4537443	3030805	2690834	1.86	5.2E-12	1.48E-10	-0.92	0.000573	0.039109
COMPOUND_398_212a9_051908	Rp	-	398.2120	9.05				(511.1938, 670.13)(512.1869, 252.6)(513.2007, 4581.98)(397.2047, 728685.75)(398.2084, 154584.11)(399.2145, 74286.28)(795.4176, 1933.17)(796.4214, 965.96)(797.4327, 11868.54)(798.4355, 5506.51)(799.4477, 25246.61)(800.4511, 11568.13)(801.4499, 4935.67)	157852.9	160128.7	139934	158689.9	603789.8	426374.8	1044924	1424371	1.22	1.56E-06	7.5E-06	0.14	0.611962	0.814985
COMPOUND_398_2129a8_772959	Rp	-	398.2129	8.77				(397.2052, 25211.3)(398.208, 6148.52)(399.2126, 2121.6)(400.2059, 291.75)(795.4168, 3262.22)(796.4201, 1547.14)(797.421, 774.77)(798.4219, 238.1)(799.4172, 59.87)	185079	98777.25	185162	104111.5	1878052	928775.9	1021511	882956.3	1.92	3.28E-13	1.99E-11	-0.55	0.027374	0.226687
COMPOUND_398_2129a8_106329	Rp	-	398.2129	8.11				(397.205, 64228.56)(398.2082, 15404.26)(399.2063, 5017.69)(400.2076, 703.41)(795.4174, 2931.44)(796.4203, 1575.54)(797.4214, 639.39)	602163.8	292010.5	721258.4	650914.6	320121.3	116290.2	267184.5	123842.5	-1.18	3.34E-07	1.9E-06	-0.30	0.216702	0.524744
COMPOUND_398_2293a7_8818645	Rp	-	398.2293	7.88				(397.222, 4177.43)(398.225, 1024.38)(399.2204, 3816.23)(400.2234, 832.16)(401.2208, 321.99)	42119.04	18120.61	40271.15	14391.25	76817	25392.11	48144.27	23714.67	-0.48	0.07197	0.121679	0.54	0.09458	0.36381
COMPOUND_398_2445a9_604185	Rp	+	398.2445	9.60				(421.2332, 18751.93)(422.2357, 3855.46)(423.2444, 671.24)(437.2071, 798.18)(438.2112, 160.32)(439.2005, 185.55)(399.2515, 341585.3)(400.255, 66295.18)(401.2578, 10733.9)(402.2604, 1536.77)(814.5513, 470.21)	101827.5	141082.3	110207.4	213710.7	96475.92	71303.23	83996.52	97720.25	0.20	0.499943	0.600465	0.05	0.895811	0.954308
COMPOUND_398_2513a10_60243	Rp	+	398.2513	10.60				(797.5411, 3171.72)(835.4581, 1035.94)(437.208, 1331.48)(438.2197, 567.99)(439.2062, 2935.27)(440.2128, 719.58)(441.2032, 1466.47)(399.26, 5639.71)(400.264, 1675.22)(401.2589, 1837.39)(402.2641, 633.1)	32203.8	7492.452	33491.54	8284.305	27645.44	6971.124	30448.63	8853.65	-0.08	0.753312	0.821132	0.44	0.168116	0.480454
COMPOUND_399_1832a11_005366	Rp	-	399.1832	11.01				(833.3732, 594.62)(834.3915, 331.01)(512.1608, 1789.02)(843.3808, 737.9)(844.3899, 665.35)(845.3832, 842.07)(398.1758, 23437.02)(399.1812, 7001.04)(400.1791, 2261.14)(401.1916, 265.36)(797.339, 781.47)(798.3584, 561.01)(799.3554, 481.48)(911.2949, 540.2)(912.3169, 437.25)(913.2876, 168.93)	100051	18607.89	109990.1	34770.1	97920.07	9402.353	109210.6	41845.16	-0.09	0.772054	0.833547	-0.14	0.691778	0.857326
COMPOUND_399_3364a10_449236	Rp	+	399.3364	10.45				(422.323, 9364.0)(423.3268, 2085.96)(438.2996, 125355.13)(439.3028, 28192.77)(417.3648, 1731.81)(400.3423, 14244.47)(401.3447, 4361.25)(402.344, 567.15)	290038.8	84106.48	280762	85248.86	128922.8	117522	133388.9	83207.69	-1.55	1.27E-08	1.02E-07	0.38	0.181997	0.495594
COMPOUND_399_3364a9_184375	Rp	+	399.3364	9.18	Palmitoylcarnitine	1	10eV: 400.3445(100), 85.0271(7), 341.2713(4)	(400.343, 53203.54)(401.3464, 13585.63)(402.3489, 3114.25)(403.3462, 463.59)	199278.4	60070.98	186998.8	49497.77	167695.6	53663.97	212192.1	62264.36	-0.59	0.048549	0.085349	1.00	0.005671	0.118614
COMPOUND_400_2277a7_921427	Rp	-	400.2277	7.92				(399.2203, 15890.24)(400.2239, 3807.35)(401.2211, 1224.01)(402.2213, 188.02)	24308.06	9320.807	24818	8641.05	81344.07	22426.16	65369.14	36103.42	1.65	9.73E-11	1.47E-09	-0.34	0.174503	0.488924
COMPOUND_400_2281a8_326689	Rp	-	400.2281	8.33				(435.1993, 1376.51)(436.2007, 561.68)(399.2204, 575529.44)(400.2241, 127425.15)(401.2217, 35794.13)(799.449, 2826.81)(800.4512, 1450.08)(801.4506, 596.85)(802.452, 190.32)	1067164	403522.5	1015878	461732.2	2486519	792380.4	1941831	949854.7	1.46	4.62E-08	3.2E-07	-0.38	0.172426	0.487735
COMPOUND_400_2283a9_027945	Rp	-	400.2283	9.03				(399.2198, 1095795.6)(400.2238, 241989.56)(401.222, 65890.29)(799.4481, 7313.84)(800.4514, 3242.13)(801.4501, 1406.52)(802.4502, 430.33)(803.4486, 101.72)	902656.1	352283.7	993375.8	516136.8	4970717	5008438	3428228	2318867	1.69	2.22E-12	7.71E-11	-0.35	0.117957	0.408047
COMPOUND_400_2819a10_460036	Rp	-	400.2819	10.46				(835.5346, 1745.45)(836.5341, 1009.21)(837.5341, 520.48)(399.2746, 43311.87)(400.2777, 11243.61)(401.2794, 2237.73)(402.2824, 336.33)	20834.62	8826.05	30206.16	25440.63	25041.78	9367.897	35491.84	20320.08	0.18	0.469136	0.572936	0.66	0.026351	0.224785
COMPOUND_400_335a11_224104	Rp	+	400.3350	11.22				(423.3188, 1117.57)(424.3365, 510.04)(401.3425, 22143.68)(402.347, 7065.06)(403.3505, 1623.32)(818.7288, 650.56)	35180.04	16978.41	32364.08	14298.67	16713.67	3838.865	15092.33	4105.087	-1.19	9.51E-08	5.98E-07	0.04	0.870191	0.945212
COMPOUND_400_3354a11_06124	Rp	+	400.3354	11.06				(801.6795, 1615.75)(423.3247, 9291.2)(424.3281, 2746.7)(425.3226, 645.94)(823.6516, 449.7)(418.3729, 1281.12)(401.3428, 11524.58)(402.3498, 4211.73)(403.3536, 996.16)(404.3535, 162.5)(818.6695, 2080.03)	62722.3	37801.64	53681.18	27624.11	65365.43	38906.06	75599.6	52487.03	0.07	0.781615	0.84059	-0.10	0.730694	0.885165
COMPOUND_401_2364a11_1903715	Rp	+	401.2364	11.19				(841.4229, 637.66)(825.4316, 1075.33)(826.424, 732.68)(827.4375, 909.26)(402.2434, 58581.97)(403.2467, 14052.43)(404.2483, 2406.05)	79677.57	16100.7	70006.75	13654.31	168385.1	58127.55	174501.4	49302.31	1.13	3.19E-06	1.41E-05	0.47	0.077779	0.333235
COMPOUND_401_3548a10_693071	Rp	+	401.3548	10.69				(841.6348, 184.0)(402.3639, 13078.68)(403.3672, 3642.6)(404.3609, 1361.26)(405.3562, 227.32)	23441.43	11808.58	13088.6	1188.989	18813.17	7492.618	38018.23	24293.9	0.34	0.073697	0.124132	0.71	0.002582	0.0718
COMPOUND_402_2774a10_427682	Rp	+	402.2774	10.43				(805.5445, 5409.58)(843.5252, 3148.43)(425.2673, 15113.85)(426.27, 4625.59)(427.2723, 985.87)(428.2796, 163.17)(827.5283, 4879.23)(441.2415, 5234.4)(442.2458, 1700.92)(443.2459, 915.95)(420.3129, 218464.72)(421.3162, 58207.2)(422.3192, 9177.46)(423.3232, 1358.61)(403.2858, 66874.23)(404.2895, 17371.54)(405.2923, 3439.25)(406.2953, 472.13)	99907.06	70290.57	132107.8	256820.2	66876.07	55926.41	177473.9	116803.2	0.25	0.283907	0.383309	0.90	0.001352	0.058548
COMPOUND_402_2973a10_628744	Rp	-	402.2973	10.63				(401.2903, 20122.72)(402.2935, 5083.25)(403.2947, 807.4)(404.2954, 118.07)(447.2953, 803.45)(448.2936, 326.78)	19438.68	8354.63	23474.07	19549.87	20851.5	19545.92	29868	16562.92	-0.56	0.039027	0.070763	1.05	0.001326	0.058548
COMPOUND_402_2974a10_687773	Rp	-	402.2974	10.69	no msms			(401.2904, 23365.15)(402.2939, 4853.34)(403.2975, 1186.4)(404.3014, 183.43)(447.2996, 294.89)(448.2902, 136.03)	30869.2	16268.32	36572	29420.74	31972	16863.97	58210.7	32920.03	-0.33	0.182905	0.270454	1.08	0.000456	0.034251
COMPOUND_402_3469a10_517959	Rp	+	402.3469	10.52				(425.3441, 1136.78)(426.3415, 254.36)(827.6715, 847.35)(828.6936, 469.35)(420.3707, 234.88)(441.3169, 3299.82)(442.3195, 861.3)(403.3545, 7036.4)(404.3567, 2185.49)(405.3552, 591.65)(406.3644, 89.94)	46795.57	20224.28	32804.48	13796.1	61530.15	34769.09	71858.2	43318.29	0.68	0.009244	0.019696	0.26	0.404845	0.6912
COMPOUND_402_3522a11_524878	Rp	+	402.3522	11.52				(425.3401, 6066.19)(426.3438, 1646.46)(441.2952, 730.41)(442.2925, 486.7)(420.3784, 1821.22)(421.3778, 776.34)(422.3927, 288.12)(403.3587, 3903.03)(404.362, 1275.36)(405.3712, 363.67)(822.7547, 1305.58)	32035.45	8778.075	31558.88	9094.034	24283	8031.347	22813.53	12237.28	-1.21	4.92E-07	2.67E-06	0.13	0.608551	0.812181

									1st trimester				3rd trimester									
									Control		Pre-eclampsia		Control		Pre-eclampsia		Time			Interaction		
Compound	Column	Ion_mode	Mass	Retention_	Identification	ID_level	MSMS_spectra	CompositeSpectrum	Mean	SD	Mean	SD	Mean	SD	Mean	SD	Value	raw p	FDR_p	Value	raw p	FDR_p
COMPOUND_403_1595a9_920933	Rp	-	403.1595	9.92				(841.2975, 602.82)(842.3086, 359.61)(843.3161, 415.02)(438.1268, 5555.81)(439.1303, 1327.5)(440.136, 3450.43)(441.1397, 1381.45)(442.139, 1028.08)(851.3032, 317.61)(852.3264, 306.69)(853.3096, 454.23)(402.1509, 2661.09)(403.1525, 705.21)(404.1454, 6774.71)(448.1565, 4589.8)(449.1479, 81.41)	34297.47	16709.95	36539.26	16255.11	29869	#DIV/0!	44198.43	10219.56	-1.10	1.77E-06	8.36E-06	-0.06	0.820023	0.922833
COMPOUND_403_2946a10_85513	Rp	+	403.2946	10.86				(421.3091, 1810.86)(404.3018, 19960.03)(405.3051, 4973.4)(406.3029, 307.41)	63991.72	17946.65	58426.22	15494.28	113352.6	24027.32	122140.1	28795.13	1.17	4.42E-07	2.44E-06	0.45	0.071293	0.325554
COMPOUND_404_2153a8_812526	Rp	-	404.2153	8.81				(439.188, 1312.73)(403.2091, 6227.34)(404.2133, 1280.64)(405.2212, 320.51)	25804.94	11728.33	27067.43	9992.113	26605.5	4608.215	26156.33	10854.09	-1.09	1.96E-06	9.14E-06	-0.15	0.554157	0.781442
COMPOUND_404_2185a8_720452	Rp	-	404.2185	8.72				(439.1877, 153.55)(403.2116, 5046.15)(404.2148, 1155.31)(405.225, 621.68)(406.2308, 74.54)	25145.49	9301.792	26038.85	11038.82	16396.5	86.97413	21192	8522.468	-1.31	5.87E-09	5.07E-08	0.12	0.59554	0.804532
COMPOUND_404_3134a10_927403	Rp	-	404.3134	10.93				(517.3056, 2874.93)(518.3082, 697.39)(439.2832, 423.93)(440.2824, 101.57)(403.3059, 36764.26)(404.3093, 8942.75)(405.3118, 1578.71)(406.3144, 235.88)(451.2909, 12333.44)	69639.87	33639.53	83423.82	37353.54	34854.67	25656.69	39821.44	34772.9	-0.99	6.23E-05	0.000203	-0.30	0.280094	0.587858
COMPOUND_405_2892a9_249067	Rp	+	405.2892	9.25				(406.2957, 14165.16)(407.2989, 3961.12)(408.3036, 986.53)(409.3136, 145.99)	#DIV/0!	#DIV/0!	#DIV/0!	#DIV/0!	#DIV/0!	#DIV/0!	#DIV/0!	#DIV/0!	-0.93	8.22E-05	0.000257	0.01	0.966808	0.982565
COMPOUND_406_1982a6_558121	Rp	-	406.1982	6.56		4	10 eV: 329.1735(100), 301.1781(16), 405.1858(8), 129.8673(8)	(405.1915, 12883.21)(406.1944, 3017.74)(407.1986, 539.75)(408.1995, 63.84)(451.1964, 863.71)(452.2017, 225.27)	20064.62	5250.175	21251.16	5105.504	37616.57	6814.944	41416.58	13086.58	1.25	7.21E-06	2.96E-05	-0.28	0.360621	0.666906
COMPOUND_406_2268a11_005156	Rp	+	406.2268	11.01				(813.4362, 2845.04)(851.4516, 5138.57)(429.2132, 335.71)(835.4424, 1933.21)(445.1843, 210.52)(407.2354, 8612.04)(408.2404, 2597.73)(409.2377, 6316.94)(410.2378, 1682.25)(411.2337, 2917.73)(412.2377, 885.55)(413.2242, 1065.05)	48995.57	16542.03	45654.87	18072.65	59595.14	31526.98	54549.82	20583.48	0.53	0.066344	0.11379	0.08	0.813006	0.92097
COMPOUND_406_2277a11_001731	Rp	+	406.2277	11.00				(813.4351, 391.78)(851.4515, 4977.15)(429.2084, 453.8)(835.4406, 1915.92)(445.1899, 222.97)(407.2368, 9846.6)(408.2403, 2628.95)(409.2378, 8282.86)(410.2399, 2297.37)(411.2352, 3498.93)(412.2367, 1213.21)(413.2223, 213.14)	45822.18	17845.26	48529	18175.51	55143.14	30784.56	52472.11	20943.63	0.44	0.13771	0.212996	-0.04	0.907354	0.958319
COMPOUND_406_2347a9_558533	Rp	-	406.2347	9.56				(405.2276, 20771.5)(406.2312, 5453.51)(407.2405, 2382.42)(408.2433, 289.11)	58600.53	33730.41	61153.67	29633.09	53396.25	25568.55	71602.2	28696.27	-1.02	1.3E-05	5.09E-05	-0.19	0.465085	0.732912
COMPOUND_406_2351a9_501248	Rp	-	406.2351	9.50				(405.2277, 15945.5)(406.2311, 4219.3)(407.236, 958.28)(408.2443, 138.84)	42923.72	23753.85	45211.2	21258.5	38360.75	16455.02	53326.5	17786.22	-1.03	1.1E-05	4.37E-05	-0.18	0.486275	0.747178
COMPOUND_406_2353a9_245045	Rp	-	406.2353	9.25				(405.2277, 38597.25)(406.2312, 9852.23)(407.234, 1833.7)(408.2353, 246.61)	52391.81	29207.48	55324.02	26633.16	57345.67	6367.557	64747.8	24397.71	-1.04	1E-05	4.01E-05	-0.15	0.552234	0.780002
COMPOUND_407_1523a10_501683	Rp	-	407.1523	10.50				(849.2845, 427.31)(850.2834, 380.81)(851.3005, 333.05)(520.1504, 460.85)(521.153, 236.49)(522.1511, 1263.9)(523.1447, 678.3)(859.3126, 468.94)(860.3299, 202.55)(861.3235, 272.4)(406.1449, 14157.24)(407.1503, 4640.42)(408.1505, 1347.83)(409.1461, 147.75)(813.3053, 581.9)(814.3061, 528.26)(815.2813, 482.25)(452.1701, 966.3)(453.1812, 455.76)(454.1645, 6394.53)(455.1661, 1723.13)(927.3322, 227.38)(928.3293, 176.42)(929.3132, 273.63)	116957.9	50044.06	126983.7	56644.95	120265	43095.01	141817.2	51876.54	-0.16	0.578874	0.675453	0.12	0.717289	0.876415
COMPOUND_407_2472a11_585079	Rp	+	407.2472	11.59				(853.4761, 2656.4)(854.4609, 849.54)(837.4748, 905.0)(446.2181, 227.37)(408.2563, 9999.27)(409.2595, 3205.27)(410.2575, 4524.43)(411.2555, 1457.45)(412.2517, 1966.46)(413.2605, 661.04)(832.5003, 359.05)	26970.14	8912.843	26129.83	7267.28	31603.43	8447.252	30241.02	9799.798	0.47	0.104978	0.169836	-0.08	0.804999	0.917422
COMPOUND_407_3021a10_822291	Rp	+	407.3021	10.82				(425.3239, 2599.57)(408.3097, 518468.53)(409.3133, 118893.58)(410.3157, 18611.31)(411.3171, 2467.95)	165632.9	123284.2	280184.2	159471	584333.6	488235.5	618532.7	404298.6	1.26	1.73E-06	8.23E-06	-0.37	0.196509	0.507758
COMPOUND_407_3031a10_7575	Rp	+	407.3031	10.76				(408.31, 284984.28)(409.313, 65166.04)(410.3153, 10655.44)(411.3187, 1489.37)(412.3167, 167.92)	300016	166329.1	385606.4	165803.3	583809.7	417515.9	602489.6	352927.8	0.99	0.000421	0.001182	-0.25	0.439241	0.715597
COMPOUND_408_1967a10_444244	Rp	+	408.1967	10.44				(817.3753, 500.44)(818.3445, 518.48)(855.3391, 610.18)(856.3375, 449.77)(431.1869, 465.71)(432.2041, 56.34)(433.1905, 426.54)(839.3718, 287.47)(840.3918, 521.81)(426.2118, 1004.06)(409.2039, 30139.56)(410.2074, 7003.22)(411.2055, 11384.28)(412.2077, 3008.81)(413.2032, 4704.49)(414.2044, 1467.12)(415.2092, 1115.87)(205.1057, 299.91)(205.6049, 48.25)(206.1052, 287.37)(834.4653, 785.68)(835.4462, 690.41)	26116.52	13628.76	31709.09	18836.71	29247.38	12493.11	51710.61	30291.26	0.22	0.352223	0.454039	0.60	0.0353	0.250187
COMPOUND_408_2143a6_7962737	Rp	-	408.2143	6.80		4	20 eV: 331.1919(100), 44.9974(27), 282.1274(15), 125.0615(7)	(407.2072, 86569.58)(408.2106, 21334.57)(409.2141, 3542.4)(410.2187, 499.93)(453.2114, 2474.95)(454.2141, 647.51)(455.2231, 844.83)	88078.04	29996.61	96324.46	35806.68	191484.7	60492.18	155775.9	85486.29	1.45	8.51E-07	4.29E-06	-0.82	0.010769	0.163867
COMPOUND_408_2516a10_857087	Rp	+	408.2516	10.86				(855.4652, 879.08)(431.2541, 993.15)(447.207, 871.38)(409.2576, 22898.01)(410.26, 5714.49)(411.2629, 1621.05)(412.2633, 236.15)(834.5378, 25160.23)(835.5366, 12123.26)(836.5456, 352481.94)	1157785	267362.9	1103329	381120.4	353864.1	504841.6	277933.8	416813.6	-0.62	0.029724	0.055968	0.19	0.564402	0.785799
COMPOUND_409_2484a8_333355	Rp	+	409.2484	8.33				(432.2391, 2317.33)(433.2424, 702.03)(434.249, 262.1)(410.2544, 15392.23)(411.2605, 3762.13)(412.2695, 1011.41)	38102.52	17972.18	32774.61	14092.26	24487	#DIV/0!	22681	15809.39	-1.29	2.47E-09	2.44E-08	0.20	0.361079	0.667063
COMPOUND_410_2115a10_692786	Rp	+	410.2115	10.69	no msms			(821.4467, 869.73)(859.4208, 579.45)(860.4015, 385.13)(861.4114, 523.78)(843.4116, 1240.74)(844.3978, 864.86)(845.4017, 770.42)(428.2356, 423.84)(429.2393, 166.08)(411.2198, 36324.95)(412.2227, 8913.26)(413.221, 14718.33)(414.2235, 2848.36)(415.2164, 5837.38)(416.2197, 1466.55)(417.2214, 882.43)	35808.13	17250.28	35715.5	19126.48	43358.27	17403.81	79627.54	41759.72	0.09	0.704234	0.783952	1.20	4.79E-05	0.008997
COMPOUND_410_2124a10_690761	Rp	+	410.2124	10.69	no msms			(821.4476, 1328.53)(859.4135, 666.39)(860.4153, 336.67)(861.4142, 716.76)(843.4282, 844.72)(844.4369, 290.78)(845.4153, 376.8)(428.2271, 297.74)(429.227, 122.23)(411.2196, 40396.33)(412.2231, 9500.65)(413.2207, 15421.64)(414.223, 3613.58)(415.2151, 6025.9)(416.2193, 1705.22)(417.2205, 1216.51)	36197.93	16500.29	34022.03	14252.87	38082.55	15848.79	71732.07	42615.66	0.09	0.712135	0.788624	1.11	0.000145	0.017228
COMPOUND_410_2376a11_190169	Rp	+	410.2376	11.19				(843.4354, 5525.64)(449.2112, 763.91)(428.2541, 3538.06)(411.2437, 67155.2)(412.2476, 19429.28)(413.254, 3885.69)	123467.8	24253.01	106951.2	21761.42	257305.1	85329.38	271160.9	78996.67	1.13	2.32E-06	1.07E-05	0.52	0.049732	0.282107
COMPOUND_410_3175a11_228687	Rp	-	410.3175	11.23				(409.3103, 5853.23)(410.314, 1867.99)(411.317, 425.87)(933.6213, 480.36)(934.6334, 250.36)	23360.13	2822.246	24523.67	2948.484	19191.43	3639.782	19058.75	3384.971	-1.07	2.54E-05	9.24E-05	-0.27	0.327449	0.634609
COMPOUND_410_4245a11_614339	Rp	+	410.4245	11.61				(821.8348, 215.47)(822.8311, 414.4)(823.8474, 344.51)(859.7935, 264.97)(860.811, 129.33)(861.785, 769.37)(843.8053, 3267.02)(844.793, 435.09)(449.385, 681.05)(411.4314, 74452.07)(412.4347, 21144.25)(413.4379, 2898.76)(414.4352, 456.12)	65309.67	36998.65	68015.53	39663.49	100712	95009.86	99078.89	107352.4	0.45	0.136182	0.211505	-0.08	0.822854	0.922843
COMPOUND_411_1149a10_576438	Rp	-	411.1149	10.58				(857.2086, 512.78)(858.2201, 232.68)(859.2095, 543.63)(524.0815, 748.91)(410.1076, 29475.27)(411.1129, 6342.74)(412.1052, 19394.04)(413.1078, 4242.97)(414.1003, 4303.06)(415.1015, 444.33)(821.2167, 471.63)(822.2163, 442.44)(935.2227, 819.45)(936.2256, 499.0)(937.211, 534.93)	16522.38	6504.652	18002.5	6438.605	21041.88	11963.82	35015.7	21966.37	0.54	0.005318	0.011952	0.60	0.00888	0.149233
COMPOUND_412_1914a6_7693324	Rp	-	412.1914	6.77				(411.1838, 7303.68)(412.187, 1753.46)(413.197, 2289.64)	22919.17	10180.12	23756.47	7402.222	48327.58	16095.78	33707.09	13005.26	1.32	3.14E-06	1.39E-05	-0.86	0.006422	0.125019
COMPOUND_412_2218a10_011734	Rp	-	412.2218	10.01				(525.1951, 1119.49)(526.1963, 443.05)(527.2105, 319.98)(411.2144, 12840.39)(412.2176, 3707.97)(413.2221, 853.64)(414.2282, 143.19)(823.4347, 377.98)(824.4503, 309.82)(825.4132, 334.59)	38175.23	15262.45	43977.67	18895.74	48993.75	19322.92	58855.27	23777.21	-0.85	0.000454	0.00127	-0.31	0.261753	0.572854
COMPOUND_412_2277a11_579544	Rp	-	412.2277	11.58				(859.438, 537.16)(860.4522, 400.16)(447.1958, 2721.08)(448.1983, 1321.12)(449.1938, 2711.77)(450.1942, 1221.53)(451.1908, 1339.21)(452.1924, 626.85)(453.1817, 866.22)(525.2113, 3991.04)(526.2096, 992.78)(527.2091, 3597.61)(528.2068, 1340.39)(529.2069, 1249.39)(530.2075, 595.54)(531.2183, 528.94)(869.4625, 901.32)(870.4615, 453.79)(871.4757, 564.46)(411.2201, 4131.13)(412.2231, 1964.7)(413.2217, 1967.25)(414.2152, 438.2)(415.2231, 234.96)(823.4407, 335.97)(824.4525, 200.58)(825.4306, 303.49)(457.2283, 11137.64)(458.2307, 3194.44)(459.2253, 20425.6)(460.2272, 5786.91)(461.2219, 8185.82)(462.2253, 2201.45)(463.2209, 1618.84)(937.4494, 797.24)(938.4423, 231.55)(939.4259, 387.54)	104478.9	24084.4	112224.9	22933.08	116186.4	31578.34	107224.7	33301.96	0.40	0.184271	0.272116	-0.74	0.041196	0.265944
COMPOUND_412_298a10_453484	Rp	+	412.2980	10.45				(435.2769, 2065.23)(436.2817, 1160.08)(437.2905, 1063.11)(413.3065, 15295.25)(414.3102, 4726.48)(415.317, 2079.53)	48431.02	13141.97	50088.25	15807.51	37368	22109.62	42988.88	25804.45	-0.85	0.000361	0.001025	-0.24	0.362048	0.667758

									1st trimester				3rd trimester									
									Control		Pre-eclampsia		Control		Pre-eclampsia		Time			Interaction		
Compound	Column	Ion_mode	Mass	Retention_	Identification	ID_level	MSMS_spectra	CompositeSpectrum	Mean	SD	Mean	SD	Mean	SD	Mean	SD	Value	raw p	FDR_p	Value	raw p	FDR_p
COMPOUND_413_1796a10_660368	Rp	+	413.1796	10.66				(827.3603, 430.72)(828.3765, 1290.75)(829.363, 359.35)(865.3265, 863.71)(866.3342, 482.66)(436.1741, 462.89)(431.2113, 13277.82)(432.2129, 3509.47)(433.2121, 9714.0)(414.1863, 14904.87)(415.1898, 3533.35)(416.1957, 756.75)(844.3875, 848.34)	#DIV/0!	#DIV/0!	#DIV/0!	#DIV/0!	#DIV/0!	#DIV/0!	#DIV/0!	#DIV/0!	0.57	0.021589	0.042131	0.01	0.970608	0.984052
COMPOUND_413_1973a10_598261	Rp	-	413.1973	10.60				(861.4038, 302.04)(862.4085, 290.97)(863.3807, 474.35)(448.1766, 690.83)(871.3648, 172.11)(872.3815, 381.3)(873.4073, 144.74)(412.1919, 6763.06)(413.1935, 2331.47)(414.1768, 1369.85)(825.3892, 153.68)(826.3561, 429.93)(827.3829, 207.88)(939.4055, 366.46)	16220.87	4189.969	15806.64	4663.607	#DIV/0!	#DIV/0!	12253.5	1981.561	-0.87	0.00011	0.00034	-0.03	0.890197	0.953702
COMPOUND_413_2926a0_9439945	Hilic	+	413.2926	0.94				(827.5944, 761.43)(828.6049, 553.76)(849.5682, 8740.58)(431.311, 292.48)(414.301, 130725.42)(415.3044, 36811.69)(416.3074, 6024.66)(417.3116, 761.58)	112826	54810.32	105091.1	50066.38	118269	107161	106246.6	154465.7	-0.04	0.883173	0.922498	-0.28	0.405451	0.6912
COMPOUND_413_2937a9_0965185	Rp	+	413.2937	9.10				(849.5708, 328.45)(850.5794, 315.71)(414.3011, 389292.78)(415.3048, 108555.97)(416.3099, 21310.46)(417.3149, 3054.45)	235782	171850.8	184936.5	123079.8	282136.9	252735.9	268071	370184.7	0.18	0.520481	0.620038	-0.23	0.46937	0.734042
COMPOUND_413_9736a8_737131	Rp	-	413.9736	8.74				(862.9127, 188.72)(863.9335, 101.31)(526.9765, 140.13)(412.9659, 7745.85)(413.9679, 733.24)(414.9749, 135.5)	15773.38	5963.183	18239.21	6048.873	12706.8	2386.555	14171.86	4751.888	-0.51	0.000312	0.000895	-0.09	0.543022	0.776977
COMPOUND_414_2052a8_545986	Rp	+	414.2052	8.55				(437.1948, 2724.68)(438.1989, 546.02)(439.1904, 296.73)(453.1685, 532.41)(454.1759, 218.06)(455.1773, 157.89)(432.2388, 17564.25)(433.2417, 5117.59)(434.2457, 855.08)(435.2475, 132.16)(415.213, 25194.39)(416.2161, 6330.29)(417.2202, 1287.26)(418.2227, 223.17)	101241.2	59944.81	95157.04	55672.09	136730.5	35880.53	135164.8	39769.24	0.88	0.001793	0.004481	0.11	0.734748	0.888627
COMPOUND_414_2066a7_793913	Rp	-	414.2066	7.79				(413.1996, 524736.0)(414.2032, 118528.36)(827.4075, 5751.37)(828.411, 2743.61)(829.4215, 7975.27)(830.4247, 3546.07)(831.4342, 3451.38)(832.4377, 1569.5)(833.4376, 657.42)	15603.55	5411.732	19756.35	15328.06	371773.6	717894.8	175326.9	194885	1.73	8.42E-18	9.48E-15	-0.28	0.091385	0.359093
COMPOUND_414_2069a6_9546723	Rp	-	414.2069	6.95				(413.1997, 35411.78)(414.2026, 8695.66)(415.2023, 2593.49)(416.2052, 427.16)	27039.67	14059.78	20596.63	8942.226	133181.5	65482.65	88284.28	78846.68	1.52	5.56E-13	2.91E-11	-0.33	0.101424	0.37567
COMPOUND_414_207a7_473809	Rp	-	414.2070	7.47				(413.1997, 36687.21)(414.2026, 8339.87)(415.2125, 11096.33)(416.216, 2570.99)(417.2135, 802.53)(827.4151, 176.47)	199675.5	141824.2	215419.2	79139.81	91804	43502.4	89102.91	45316.78	-0.91	2.03E-05	7.55E-05	-0.59	0.012188	0.168989
COMPOUND_414_2072a8_005874	Rp	-	414.2072	8.01				(413.1998, 21347.25)(414.2028, 5311.82)(415.2073, 2019.67)(416.2015, 321.38)(827.4107, 186.4)(828.4154, 73.49)	21862.46	8162.23	26519.94	13157.74	207146.9	120607	153576.1	114379.5	1.84	1.04E-14	1.95E-12	-0.51	0.018872	0.196411
COMPOUND_414_3135a9_61439	Rp	+	414.3135	9.61				(437.3048, 1322.0)(438.2977, 260.41)(439.3103, 1808.66)(851.6132, 6254.42)(852.6166, 1848.37)(853.6156, 550.61)(415.3205, 7075.87)(416.3256, 2170.99)(417.3331, 1671.09)	88289.29	35549.27	84847.3	35254.96	79466.43	38332.71	83454.3	41204.09	0.02	0.958396	0.97523	0.05	0.897823	0.954626
COMPOUND_414_3155a10_468377	Rp	+	414.3155	10.47				(829.5919, 245.42)(867.5936, 536.07)(868.5806, 340.83)(869.5931, 423.91)(415.3204, 16079.34)(416.3249, 5376.27)(417.3324, 1359.03)	302804	#DIV/0!	173338	41094.22	183091.6	112560.8	128974.6	81173.27	1.64	2.95E-15	7.92E-13	-0.54	0.004309	0.100035
COMPOUND_414_3824a10_219754	Rp	+	414.3824	10.22				(415.3904, 6068.53)(416.3899, 1889.64)(417.394, 469.64)(418.396, 64.77)	23413.37	11177.95	23411.58	18820.14	24523.44	17118.31	26452.28	18317.62	-0.09	0.752987	0.821132	-0.10	0.755008	0.895058
COMPOUND_415_2212a10_60256	Rp	+	415.2212	10.60				(831.491, 1169.11)(832.5149, 774.28)(869.3633, 697.91)(870.3504, 611.39)(871.3591, 293.64)(438.2174, 772.88)(853.4566, 809.03)(454.196, 877.74)(416.2274, 12527.66)(417.2293, 3710.91)(418.235, 616.8)	23159.6	6673.859	23162.47	7810.851	21979.77	8501.781	25100.71	9127.343	-0.25	0.394424	0.498733	0.31	0.374945	0.67723
COMPOUND_415_3577a12_111721	Rp	+	415.3577	12.11				(869.6571, 1240.05)(438.3463, 1691.36)(439.3499, 588.63)(440.3544, 174.57)(853.7413, 1266.88)(854.7333, 660.38)(855.7384, 436.15)(454.324, 515.51)(455.3318, 296.04)(416.3657, 14026.61)(417.3704, 4457.32)(418.3713, 905.15)(419.3793, 104.18)(848.719, 8143.97)(849.7223, 5614.27)	210257.5	122828.6	152880.5	111151.4	166345.9	80123.1	185612.5	118550	-0.29	0.316493	0.418506	0.61	0.085211	0.346484
COMPOUND_415_3678a11_101445	Rp	+	415.3678	11.10				(831.7513, 3753.03)(853.7281, 1123.77)(854.7292, 869.08)(416.374, 10677.23)(417.3762, 2839.15)(418.3758, 1398.95)(848.7646, 3491.44)(849.778, 317.73)	25056.41	11409.94	23795.65	11675.69	36525.31	23718.5	46250.92	20882.44	0.72	0.00273	0.006569	0.50	0.075135	0.329961
COMPOUND_416_2218a7_448072	Rp	-	416.2218	7.45				(415.2157, 81530.55)(416.2187, 19003.73)(417.2167, 6028.96)(418.2169, 1001.52)(831.4394, 117.98)(832.4375, 80.15)(461.2152, 414.88)(462.2232, 112.0)	71413.85	34339.35	74809.2	31695.26	275317.6	130970.8	208154	128660	1.69	5.04E-11	9.14E-10	-0.52	0.041026	0.265944
COMPOUND_416_2222a6_1842346	Rp	-	416.2222	6.18				(867.433, 72.65)(415.2155, 15924.16)(416.2187, 3699.06)(417.2171, 1049.6)(418.2169, 178.21)(831.4458, 71.17)	#DIV/0!	#DIV/0!	#DIV/0!	#DIV/0!	#DIV/0!	#DIV/0!	#DIV/0!	#DIV/0!	1.45	2.98E-15	7.92E-13	0.00	0.995923	0.998139
COMPOUND_416_2222a7_77997	Rp	-	416.2222	7.78				(415.2157, 188867.06)(416.2188, 44725.54)(417.2171, 13219.07)(418.2183, 2261.99)(831.4323, 481.21)(832.4415, 164.87)(833.4375, 200.14)(834.4161, 25.87)	107176.7	151020.7	110530	128826.3	968714.4	917832.7	598299.1	733715.7	1.58	8.48E-09	7.13E-08	-0.66	0.020159	0.199116
COMPOUND_416_2224a7_08239	Rp	-	416.2224	7.08				(415.2151, 19837.45)(416.2186, 5018.33)(417.216, 1401.49)(418.2168, 235.87)	30570.04	17098.65	30082.93	23798.3	120298.6	48849.61	100392.1	81162.08	1.52	1.2E-09	1.29E-08	-0.23	0.356442	0.665596
COMPOUND_416_2224a8_630362	Rp	-	416.2224	8.63				(415.2154, 15773.05)(416.2192, 3929.71)(417.2165, 1154.13)(418.2166, 174.7)	23164.29	10378.3	24891.44	10920.35	76027.14	48041.59	52397.59	37691.39	1.55	8.53E-08	5.46E-07	-0.75	0.016778	0.1918
COMPOUND_416_2225a8_2940235	Rp	-	416.2225	8.29				(415.2157, 47955.06)(416.2188, 10502.98)(417.2166, 3467.44)(418.2185, 582.7)(831.4383, 3710.75)(832.4417, 1547.27)(833.4407, 781.84)	17044.08	6606.232	18554.61	10980	275101.8	667544.4	117750.6	134520.5	1.55	3.45E-14	4.32E-12	-0.20	0.267018	0.575669
COMPOUND_416_2227a7_795285	Rp	-	416.2227	7.80				(415.2157, 188867.06)(416.2188, 44725.54)(417.2171, 13219.07)(418.2183, 2261.99)(831.434, 3095.88)(832.4373, 1423.36)(833.4384, 616.73)(834.4537, 134.04)	112845.5	133861	117890.1	115328.6	783140.7	757629.2	702961.4	877644.5	1.49	1.2E-11	2.9E-10	-0.20	0.323725	0.630999
COMPOUND_416_2227a8_400249	Rp	-	416.2227	8.40				(415.2154, 38361.23)(416.2185, 8659.52)(417.2153, 2899.92)(418.2184, 501.49)(831.4669, 71.36)	20224.61	7935.229	15020.13	4047.682	48410.71	32498.62	48212.47	51886.71	1.39	9.38E-10	1.04E-08	-0.24	0.291588	0.601884
COMPOUND_416_3303a10_157481	Rp	+	416.3303	10.16				(439.3156, 1315.9)(440.3232, 724.88)(441.322, 1198.23)(455.2978, 15397.64)(417.3366, 13701.81)(418.3403, 4050.78)(419.3446, 1086.3)	25022.33	4734.435	#DIV/0!	#DIV/0!	32510.31	12835.23	48485.62	20284.92	1.02	1.12E-10	1.67E-09	0.46	0.004464	0.102583
COMPOUND_416_3623a10_757749	Rp	+	416.3623	10.76				(871.6544, 567.26)(439.3403, 1161.54)(855.6815, 1771.79)(856.7045, 1050.64)(434.386, 1599.39)(455.3334, 1583.57)(456.3307, 584.82)(417.3695, 38582.68)(418.3727, 10545.04)(419.3698, 2518.87)(420.3764, 405.4)	70036.48	31969.53	66292.14	28044.34	80304.5	48254.96	90929.89	39517.78	0.29	0.324067	0.424795	0.35	0.305528	0.613089
COMPOUND_416_3626a10_789158	Rp	+	416.3626	10.79				(871.6578, 811.28)(439.3587, 650.55)(855.7127, 257.7)(856.7136, 2205.67)(455.3355, 1009.9)(456.3328, 447.75)(434.3899, 4972.98)(417.3694, 19166.51)(418.3727, 4928.82)(419.3652, 1109.98)(420.3754, 148.93)	80026.24	35178.93	66609.95	28842.59	108599.7	70584	120948.5	57807	0.32	0.246811	0.342042	0.55	0.094291	0.36381
COMPOUND_418_3098a11_388098	Rp	+	418.3098	11.39				(441.2982, 4187.52)(442.3023, 1303.56)(443.3006, 420.44)(436.3442, 2427.24)(437.3552, 1132.17)(438.3533, 809.06)(419.3167, 39649.76)(420.3202, 11711.42)(421.3235, 2292.74)(422.3255, 391.19)	128711.2	53697.51	113388	80964.42	257469.8	243194.1	211199	93727.88	0.94	0.000354	0.001009	0.38	0.202586	0.5149
COMPOUND_418_3459a11_67529	Rp	+	418.3459	11.68				(837.7028, 990.3)(875.6495, 590.46)(441.334, 1692.99)(442.3332, 639.62)(443.3445, 173.31)(859.6639, 555.51)(457.3119, 453.64)(458.3069, 137.45)(459.3169, 279.82)(436.3786, 7560.39)(437.3835, 2309.3)(438.385, 668.15)(439.404, 103.21)(419.3529, 6597.22)(420.3556, 2188.57)(421.3609, 600.93)(854.7533, 545.57)(855.7321, 1047.49)	87255.17	13732.89	82154.17	13073.21	90954.57	9158.36	90714.11	9297.507	0.29	0.316667	0.418506	0.39	0.25619	0.56678
COMPOUND_418_3461a12_435217	Rp	+	418.3461	12.44				(419.3525, 11564.88)(420.3555, 3730.67)(421.3597, 781.44)(422.3599, 122.51)	86792.51	14615.63	76307.22	15596.31	75655.14	12889.38	78062.19	17013.25	-0.63	0.028022	0.053074	0.76	0.025124	0.219303
COMPOUND_420_1593a9_919672	Rp	-	420.1593	9.92				(875.3025, 310.45)(876.2842, 156.09)(877.2823, 319.77)(885.284, 262.12)(886.2882, 133.08)(887.3001, 304.43)(419.1524, 1637.01)(420.157, 589.2)(421.1541, 1008.54)(422.1579, 158.22)(423.15, 369.86)(839.3122, 609.1)(840.2991, 555.18)(841.2975, 463.6)(467.1576, 8112.35)(468.1607, 1714.87)(469.1558, 3446.72)(953.2692, 252.09)	74104.19	27585.23	79761.09	29005.89	72672.5	35560.63	82464.55	31756.42	-0.96	4.41E-05	0.000148	-0.27	0.296457	0.606376
COMPOUND_420_1727a11_196837	Rp	-	420.1727	11.20				(533.1568, 1169.89)(419.1615, 9823.57)(420.1641, 1863.75)(421.1609, 5981.66)(422.1575, 1482.62)(423.1581, 2839.71)(424.157, 425.34)(425.1534, 1020.87)(465.1693, 1614.54)	40289.91	13845.76	40206.07	15521.25	58627.79	22285.4	58336.28	19792.29	0.93	0.000923	0.002424	0.03	0.925521	0.966461
COMPOUND_420_2443a11_1882715	Rp	+	420.2443	11.19				(841.4929, 682.66)(879.4511, 19205.71)(863.4544, 9259.42)(459.2004, 515.58)(460.2057, 293.55)(421.2529, 52098.49)(422.2557, 14714.22)(423.2535, 56954.89)(424.2563, 15139.87)(425.2497, 22815.54)(426.252, 6793.74)(427.2519, 4166.82)(858.519, 4695.09)(859.5091, 4463.25)	285299.1	49162.76	257072.4	55643.12	457314.1	93655.01	439421.6	88122.77	1.39	1.83E-08	1.42E-07	0.16	0.535125	0.775983

									1st trimester				3rd trimester									
									Control		Pre-eclampsia		Control		Pre-eclampsia		Time			Interaction		
Compound	Column	Ion_mode	Mass	Retention_	Identification	ID_level	MSMS_spectra	CompositeSpectrum	Mean	SD	Mean	SD	Mean	SD	Mean	SD	Value	raw p	FDR_p	Value	raw p	FDR_p
COMPOUND_421_1419a10_575464	Rp	-	421.1419	10.58				(877.2499, 500.89)(878.2481, 325.94)(879.2264, 353.55)(534.1266, 1050.72)(887.2586, 328.48)(888.2737, 280.51)(889.2447, 532.26)(890.2673, 384.78)(891.2575, 388.03)(420.1364, 10259.94)(421.1387, 2701.57)(422.1341, 3944.1)(423.139, 1088.72)(424.1449, 257.52)(841.2713, 411.55)(842.2655, 341.54)(843.2595, 498.58)(466.1566, 3799.78)(955.2117, 409.37)(956.2411, 162.51)	23733.48	8405.882	23532.71	9872.408	31846.33	10214.87	43692.36	16792.85	0.61	0.010104	0.02144	0.52	0.064647	0.307286
COMPOUND_422_2299a8_261905	Rp	-	422.2299	8.26				(421.2226, 56391.05)(422.226, 15376.61)(423.2277, 2969.35)(424.23, 435.2)	134624.9	74437.64	137868.8	62076.94	117583	6748.651	114254.1	46037.46	-1.21	5.88E-08	3.94E-07	-0.15	0.525258	0.768104
COMPOUND_422_23a8_65038	Rp	-	422.2300	8.65				(421.2226, 16602.64)(422.2261, 4321.63)(423.2328, 1413.26)(424.2346, 238.38)	82623.17	42679.51	82484.78	35341.95	90622.67	5087.67	88344	37954.41	-1.09	2.79E-06	1.25E-05	-0.15	0.548044	0.77853
COMPOUND_422_2301a8_484494	Rp	-	422.2301	8.48				(421.2226, 12232.23)(422.2259, 3189.55)(423.2315, 794.63)(424.2378, 118.42)	76150.08	41809.14	75759.49	37042.65	66231.67	4132.667	74187.78	22437.06	-1.13	7.01E-07	3.66E-06	-0.12	0.617675	0.816971
COMPOUND_423_2755a10_812532	Rp	+	423.2755	10.81				(847.555, 34540.17)(885.5486, 6220.86)(462.2481, 833.39)(463.2418, 981.0)(464.2501, 569.05)(424.2836, 19138.72)(425.2864, 5469.79)(426.2842, 2398.35)(864.6022, 2051.78)(865.6045, 2692.48)(212.6382, 60.06)(213.1487, 1132.22)	393176.8	198274.4	263080	224084.9	70836.91	37003.17	70821.98	26255.36	0.37	0.14871	0.226482	-0.07	0.826177	0.923809
COMPOUND_423_2778a10_781187	Rp	+	423.2778	10.78				(847.5553, 34283.1)(885.5375, 2839.68)(462.2496, 638.58)(463.2425, 335.04)(464.2518, 590.27)(424.2835, 22640.89)(425.286, 5746.37)(426.2865, 2559.8)(427.2893, 444.88)(212.6427, 50.3)(213.1484, 1269.42)(864.579, 12856.44)(865.5832, 6965.29)	192400.7	106194.9	267976.3	246010.3	61356.09	29959.82	61044.77	22010.48	0.91	2.9E-05	0.000104	-0.10	0.668864	0.846185
COMPOUND_423_3348a9_032939	Rp	+	423.3348	9.03	ACar 18:2	2	20 eV: 424.3428(100), 85.0278(75), 157.0459(10)	(446.3201, 562.18)(424.3437, 36929.04)(425.3467, 9967.41)(426.3478, 2423.03)(427.3436, 384.02)	113263	42069.25	103441.4	30991.68	105162.7	32652.62	92861.37	29534.16	-0.02	0.934149	0.95693	-0.43	0.206677	0.518302
COMPOUND_425_3519a9_302936	Rp	+	425.3519	9.30	ACar 18:1	2	10 eV: 246.3608(100), 85.0283(18), 239.1203(7)	(426.3587, 100418.5)(427.3616, 28131.3)(428.3647, 4802.42)(429.3714, 708.69)	371843	147464.8	328401.9	112081	326472.4	118922.9	358226.9	125795	-0.33	0.276475	0.375752	0.57	0.116486	0.405455
COMPOUND_425_9744a10_021565	Rp	+	425.9744	10.02				(464.9495, 156.83)(444.0168, 113.7)(426.9807, 5361.34)(427.983, 661.57)(428.9657, 636.16)	22365.5	9567.346	18005.14	7074.333	22549.4	4505.37	25772.06	9146.837	-0.16	0.564556	0.661488	1.06	0.001805	0.061415
COMPOUND_426_3722a11_76912	Rp	+	426.3722	11.77				(853.7254, 1398.92)(854.7066, 479.91)(449.3601, 3644.62)(450.3629, 1646.17)(451.365, 507.02)(875.7425, 683.54)(876.7068, 943.79)(465.3392, 488.09)(466.3412, 229.75)(467.3434, 157.71)(444.4043, 4679.61)(445.407, 1139.86)(446.4262, 97.23)(427.3784, 22176.68)(428.3822, 6264.99)(429.3736, 33173.78)(430.3773, 11020.01)(431.3812, 4585.04)(432.3824, 1024.33)(433.3859, 351.45)	#DIV/0!	#DIV/0!	#DIV/0!	#DIV/0!	#DIV/0!	#DIV/0!	#DIV/0!	#DIV/0!	-1.33	9.56E-08	6E-07	-0.01	0.964463	0.981972
COMPOUND_426_4077a13_627346	Rp	+	426.4077	13.63				(891.7856, 700.12)(892.7925, 346.92)(449.4001, 813.23)(450.4027, 350.38)(451.4107, 79.22)(427.4163, 5391.55)(428.4193, 1687.79)(429.4102, 470.95)(430.4076, 69.92)	38084.19	7593.869	36662.96	6480.603	37927.79	3456.077	38259.32	5666.328	0.05	0.87856	0.919812	0.22	0.542185	0.776977
COMPOUND_427_178a10_57897	Rp	-	427.1780	10.58				(889.2607, 357.84)(890.2839, 305.65)(899.3749, 198.52)(900.3763, 330.34)(426.1704, 5179.33)(427.1747, 2273.12)(428.1663, 1838.37)(429.1541, 192.09)(853.3133, 326.83)(854.3305, 244.2)(855.323, 410.78)	28508.42	9376.799	28415.7	11128.81	22708.25	7984.304	25329.26	8184.528	-0.40	0.124812	0.196558	0.22	0.478036	0.740906
COMPOUND_427_3671a9_576748	Rp	+	427.3671	9.58				(445.4066, 191.57)(466.332, 2125.65)(467.3189, 346.71)(428.3745, 10758.37)(429.3779, 3112.93)(430.3818, 775.11)(431.3822, 109.3)	69161.25	21520.49	65837.72	22700.42	49582.21	15888.3	54266.14	17734.1	-1.02	0.000277	0.000799	0.39	0.207364	0.518872
COMPOUND_427_9748a8_685235	Rp	-	427.9748	8.69				(540.9705, 124.11)(462.9336, 147.72)(900.9746, 193.0)(426.9676, 19413.36)(427.9707, 1956.34)(428.966, 947.03)(429.9655, 146.56)(472.9913, 117.01)	81036.75	7385.623	82621.7	5278.883	80357	4573.439	80579.77	7078.906	-0.07	0.812651	0.867341	-0.26	0.480893	0.743153
COMPOUND_428_166a10_663464	Rp	-	428.1660	10.66				(891.3191, 363.15)(541.1488, 3330.11)(542.1575, 1165.86)(543.1442, 3316.48)(544.1485, 1443.07)(545.1482, 1440.94)(546.1475, 813.86)(547.157, 599.35)(463.1292, 4552.25)(464.1298, 1363.67)(465.1313, 3754.31)(466.1364, 618.97)(467.1346, 2110.46)(468.1484, 782.94)(901.3321, 458.1)(427.1576, 41350.06)(428.1603, 16327.95)(429.1618, 4537.06)(855.3403, 749.23)(473.1614, 10249.65)(474.165, 3231.36)(475.163, 18938.69)(476.1747, 7004.95)(477.1615, 7674.33)(478.1671, 2722.61)(479.1634, 1386.79)(969.3356, 446.48)(970.3332, 133.76)	151058.9	65738.22	175169.2	104423.9	137619.3	89649.95	180523.2	114192.6	-0.41	0.146655	0.223909	0.34	0.308516	0.614305
COMPOUND_428_3664a12_090394	Rp	+	428.3664	12.09				(429.3736, 13646.16)(430.3777, 4600.38)(431.357, 4040.59)	51966.8	20184.64	47505.84	19339.8	40623.33	18687.7	34668.54	17918.06	-0.78	0.001064	0.00276	-0.16	0.548157	0.77853
COMPOUND_428_3673a13_088575	Rp	+	428.3673	13.09				(895.674, 785.11)(896.651, 479.28)(429.3735, 249545.5)(430.3768, 77447.2)(431.3798, 13584.12)(432.3844, 1699.05)(433.3807, 145.02)	974121.7	488898.3	790061.2	452268.8	436570.4	361050.3	353477.2	197560.5	-1.41	2.34E-08	1.76E-07	0.22	0.397118	0.690408
COMPOUND_428_3674a12_592195	Rp	+	428.3674	12.59	Unknown	4	20 eV: 429.3722 (100), 163.1043 (25), 71.0851 (15), 234.1586 (10)	(857.7341, 154.76)(858.7212, 153.74)(895.682, 438.94)(896.6834, 713.64)(897.6828, 514.36)(451.3557, 266.94)(452.3503, 181.92)(429.3738, 15585.32)(430.3828, 6401.77)(431.3885, 2157.45)(432.3853, 317.58)	78685.06	35261.99	62866.22	30180.65	26913.5	10901.47	41093.88	16239.6	-1.82	2.82E-13	1.87E-11	0.85	0.000375	0.030145
COMPOUND_428_3679a11_37579	Rp	+	428.3679	11.38				(857.7714, 1699.96)(879.7483, 1066.73)(880.7458, 784.81)(429.3737, 11574.41)(430.3796, 5887.58)(431.3866, 7506.85)(432.3894, 2473.79)(874.7911, 1638.8)	44976.78	22933.52	39558.9	13622.12	70989.13	23214.89	49675.41	14748.48	0.62	0.0169	0.033982	-0.14	0.650094	0.838541
COMPOUND_428_3968a10_781774	Rp	+	428.3968	10.78				(857.799, 2887.51)(895.7457, 519.74)(896.7333, 164.75)(429.406, 17341.88)(430.4083, 5161.25)(874.8011, 1234.73)	42732.05	19709.02	32397.55	14052.42	41074.23	12432.21	50155.81	22542.27	0.77	0.001072	0.002775	0.39	0.147692	0.456493
COMPOUND_429_3736a12_432636	Rp	+	429.3736	12.43				(452.3622, 2822.22)(453.3667, 974.28)(454.3717, 190.84)(881.7369, 757.56)(882.7436, 416.23)(883.7427, 196.79)(468.3374, 614.71)(469.3448, 221.51)(470.3278, 106.07)(430.3797, 206071.5)(431.386, 101793.91)(432.39, 23002.4)(433.3944, 3221.02)(434.3922, 368.42)	932115.8	300512.1	754370.7	239414.7	1016716	106848.1	1028706	264306.3	-0.08	0.77912	0.838308	1.05	0.00154	0.06002
COMPOUND_430_1733a11_847422	Rp	+	430.1733	11.85				(899.2976, 260.08)(900.2927, 191.74)(453.1686, 250.48)(883.3505, 954.98)(469.1331, 124.73)(431.1795, 65409.94)(432.1823, 17389.01)(433.1808, 4670.27)(878.3922, 693.34)	159784.5	53186.58	135089	36423.19	144110.2	34687.08	157621.3	45823.84	-0.29	0.333552	0.434951	0.74	0.040717	0.265782
COMPOUND_430_2018a10_598564	Rp	-	430.2018	10.60				(543.1802, 1990.07)(465.166, 6254.74)(905.3905, 427.4)(429.1945, 6429.25)(430.1938, 2247.49)(431.1763, 2551.79)(432.1754, 515.31)(859.3716, 229.43)(860.3565, 485.1)(475.1939, 7263.75)(476.1969, 1834.46)(477.1944, 272.83)(973.391, 442.56)(974.3801, 97.71)(975.4008, 198.53)	44926.56	14341.04	44758.11	13345.5	26031.75	7403.458	33560.32	12986.11	-1.33	2.01E-06	9.34E-06	0.52	0.084567	0.346484
COMPOUND_430_3072a9_780315	Rp	-	430.3072	9.78				(543.3006, 336.57)(544.2865, 208.72)(545.2872, 75.51)(465.2921, 1641.38)(466.2827, 268.71)(467.2906, 366.1)(468.3032, 28.88)(429.3004, 9474.79)(430.3034, 2988.92)(431.3126, 1193.49)(432.3139, 199.27)(475.3057, 4690.92)(476.3089, 1457.5)(477.3159, 484.29)	54083.13	17420.02	51071.67	13304.56	59266.93	17900.41	70824.95	20188.54	0.32	0.235414	0.329992	0.64	0.045074	0.273371
COMPOUND_430_3095a9_785857	Rp	+	430.3095	9.79				(899.5774, 255.22)(900.5933, 171.94)(453.2986, 2056.25)(454.2983, 876.6)(455.2898, 55.32)(469.2509, 268.08)(470.2588, 107.34)(431.3165, 30247.2)(432.3191, 10760.04)(433.3209, 7643.12)(434.3232, 1349.46)	129359.8	33363.61	113191.2	29123.27	137420.5	30566.11	168155.9	47968.22	0.28	0.28356	0.383069	0.94	0.004019	0.094399
COMPOUND_430_347a12_106378	Rp	+	430.3470	12.11				(861.7229, 341.64)(862.7389, 719.3)(863.7619, 245.13)(453.3339, 600.67)(454.3327, 338.15)(455.3189, 121.07)(883.6718, 559.89)(884.6323, 670.23)(885.6313, 438.02)(448.3508, 481.06)(449.3597, 202.17)(450.358, 572.15)(431.3531, 12469.41)(432.3558, 4000.17)(433.3608, 809.56)	36878.22	10007.38	34688.77	12823.31	30310.86	9405.984	31392.11	11295.27	-0.34	0.255139	0.351635	0.18	0.606527	0.810674
COMPOUND_430_3797a10_820964	Rp	+	430.3797	10.82				(861.7829, 1311.81)(453.3588, 227.97)(883.7136, 151.76)(469.3307, 5654.21)(470.3324, 2312.97)(448.4094, 957.61)(431.3859, 141786.67)(432.389, 36573.68)(433.3906, 6290.94)(434.3855, 1087.69)(435.4028, 125.92)(878.7314, 199.38)(879.7403, 124.95)(880.7352, 387.32)	391303	303255.3	341578.7	276794.2	887720.1	684596.9	1042650	723568.1	0.91	0.000604	0.001656	0.34	0.264399	0.575338
COMPOUND_430_3798a10_757505	Rp	+	430.3798	10.76				(861.7741, 503.89)(448.4019, 86.97)(469.3318, 1462.04)(470.3352, 613.3)(471.3446, 555.94)(431.3849, 764154.75)(432.3889, 200065.55)(433.3912, 30399.53)(434.3923, 3967.72)(435.3875, 399.99)(878.7761, 514.42)(879.7628, 450.27)(880.7564, 401.09)	690791.4	456766	588298.9	433880.7	1117465	767581.9	1294819	868785.8	0.64	0.019372	0.038437	0.41	0.205556	0.516497
COMPOUND_430_3806a12_4324875	Rp	+	430.3806	12.43				(453.3662, 1089.47)(454.3716, 227.42)(431.3856, 122195.88)(432.3898, 26236.36)(433.3932, 3897.0)(434.3747, 221.54)	349210.6	84738.52	299890.2	72071.76	373341.2	44075.58	388185.9	72466.69	0.35	0.19394	0.282871	0.76	0.017856	0.195199

									1st trimester				3rd trimester									
									Control		Pre-eclampsia		Control		Pre-eclampsia		Time			Interaction		
Compound	Column	Ion_mode	Mass	Retention_	Identification	ID_level	MSMS_spectra	CompositeSpectrum	Mean	SD	Mean	SD	Mean	SD	Mean	SD	Value	raw p	FDR_p	Value	raw p	FDR_p
COMPOUND_430_3816a10_828956	Rp	+	430.3816	10.83				(861.781, 1296.85)(453.3669, 105.25)(454.3447, 876.37)(883.7733, 7907.61)(448.4049, 290.45)(469.3298, 5671.94)(470.3354, 1924.86)(431.3858, 51626.31)(432.3889, 13829.5)(433.3905, 2685.18)(434.3863, 479.44)(878.8174, 28042.58)	431405.5	351993.2	383231.8	378216.6	1011105	948988.5	1219668	980836.8	0.66	0.018731	0.03733	0.34	0.295522	0.606116
COMPOUND_431_3031a0_9478264	Hilic	+	431.3031	0.95				(863.6166, 2784.29)(864.6189, 526.36)(454.2934, 1693.78)(455.297, 702.83)(456.2864, 169.14)(449.3273, 537.53)(432.3117, 246696.78)(433.3149, 69447.63)(434.3178, 11673.19)(435.3206, 1298.55)(436.3211, 158.9)	145681.2	86080.49	131570.2	60981.09	191655.4	150861.2	187732	267133.4	0.29	0.312052	0.413471	-0.35	0.302609	0.610641
COMPOUND_431_3043a9_096739	Rp	+	431.3043	9.10	Chenodeoxycholic acid glycine conju[illegible]	1	10 eV: 414.3021(100), 432.3113(50), 339.266(17), 265.1939(7), 147.0952(6)	(449.338, 4173.45)(432.3117, 472287.0)(433.3152, 136238.45)(434.318, 21336.65)(435.3204, 2865.95)	232074.4	163644.6	181764.3	115194	274572.8	237303.2	260209.8	351930.1	0.19	0.486064	0.587241	-0.25	0.433929	0.712127
COMPOUND_431_3271a11_147078	Rp	+	431.3271	11.15				(863.593, 658.82)(864.6122, 4320.1)(865.6012, 2035.08)(901.6314, 5407.38)(432.333, 22825.46)(433.3361, 6094.13)(434.3458, 1649.79)(880.7003, 2461.59)	31618.04	12030.93	27073.5	9361.337	73329.67	48101.08	82725.55	44704.23	-0.32	0.244383	0.339933	0.18	0.571563	0.789098
COMPOUND_431_8104a9_787293	Rp	+	431.8104	9.79				(864.6256, 19122.69)(865.6286, 9287.06)(866.6302, 3055.3)(867.63, 722.17)(868.6328, 167.62)(432.8185, 10504.87)(433.8218, 1620.84)(434.8238, 538.4)	237625.7	54047.93	208122.5	36743.57	256197.6	43372.05	252709.2	60635.47	0.24	0.396566	0.500317	0.61	0.076122	0.331641
COMPOUND_432_3235a9_673759	Rp	-	432.3235	9.67				(467.2939, 864.39)(468.2991, 288.0)(469.2974, 265.66)(431.3161, 10061.07)(432.3194, 3019.56)(433.328, 1366.65)(434.3334, 241.28)(863.6331, 231.17)(864.6255, 72.19)(477.3234, 470.38)	34413.03	11292.75	39169.52	16880.02	37582.08	10180.5	33597.61	14544.09	0.63	0.017267	0.034626	-0.35	0.248207	0.557845
COMPOUND_433_1201a6_149124	Hilic	+	433.1201	6.15				(434.128, 23234.52)(435.131, 3537.66)(436.1314, 643.65)	18068.9	9878.533	13533.61	7298.18	17557.57	6863.499	29907.05	13422.2	0.10	0.683231	0.768166	1.26	8.73E-05	0.012291
COMPOUND_433_1679a10_686771	Rp	-	433.1679	10.69				(901.3294, 366.63)(911.3609, 267.18)(912.35, 499.45)(913.3617, 429.24)(432.1607, 29669.16)(433.1649, 13292.35)(434.1653, 3824.74)(865.353, 675.1)(866.3491, 560.69)(478.1737, 1974.69)(479.1636, 915.19)(480.1614, 16073.85)	174934	22025.07	177679.9	33508.96	185107.2	50742.6	148476.7	35438.14	-0.49	0.032599	0.060722	-0.56	0.039487	0.261543
COMPOUND_435_2115a6_702896	Rp	+	435.2115	6.70				(436.2178, 6068.63)(437.2224, 1318.9)(438.2299, 461.62)(439.244, 79.32)	22320.23	5481.398	22732.49	4875.551	20496.08	6115.648	22409.5	4983.021	-0.18	0.542353	0.640471	0.15	0.669116	0.846185
COMPOUND_435_336a11_066518	Rp	+	435.3360	11.07				(909.6202, 2150.3)(910.6038, 2612.6)(911.6212, 2222.34)(453.3583, 1070.36)(436.3435, 140071.75)(437.3467, 39011.43)(438.3499, 6996.22)(439.3505, 1241.74)	182179.9	103200.8	141102.4	81855.95	245317.9	67871.9	275010.1	112914.4	0.68	0.011075	0.023137	0.56	0.072481	0.326093
COMPOUND_437_2898a10_448982	Rp	-	437.2898	10.45				(909.5503, 3381.13)(550.2873, 1172.37)(551.288, 598.29)(552.2949, 63.01)(436.2826, 139226.45)(437.2858, 34574.65)(438.2886, 5924.35)(439.2903, 964.85)(873.5331, 10937.14)	247299.2	72730	253543.8	80418.28	83437.86	90669.81	68494.45	70496.87	-1.28	1.74E-07	1.05E-06	-0.20	0.43033	0.707891
COMPOUND_437_2904a1_4497926	Hilic	+	437.2904	1.45				(875.5825, 353.52)(876.599, 299.16)(460.2795, 6907.03)(461.2824, 2058.39)(462.2926, 804.51)(463.2876, 126.95)(897.5484, 399.31)(898.569, 395.77)(899.5733, 147.55)(438.2983, 12030.29)(439.3019, 2940.4)(440.2908, 1033.96)(441.2879, 133.98)	53940.13	16528.48	58279.89	18788.65	28394.5	26413.09	20897.82	20881.52	-1.14	1.86E-06	8.73E-06	-0.24	0.356955	0.665625
COMPOUND_437_2908a10_447945	Rp	+	437.2908	10.45				(875.5737, 11355.81)(913.5591, 5185.14)(460.2804, 6600.42)(461.2818, 1740.19)(462.2919, 584.02)(897.566, 8127.91)(898.5746, 11739.46)(476.2593, 951.26)(477.2516, 575.9)(478.2445, 413.33)(438.2989, 87202.36)(439.3019, 20156.07)(440.3058, 3790.21)(441.3129, 671.19)	#DIV/0!	#DIV/0!	#DIV/0!	#DIV/0!	#DIV/0!	#DIV/0!	#DIV/0!	#DIV/0!	-1.37	5.17E-08	3.54E-07	0.00	0.995273	0.998139
COMPOUND_438_1679a8_226983	Rp	-	438.1679	8.23				(473.1391, 736.94)(437.1612, 5545.66)(438.1629, 1147.58)(439.1625, 433.14)(440.1672, 66.12)	23650.37	10171.31	25664.11	9312.98	16255.3	7186.263	19627.66	8328.65	-0.58	0.010177	0.021539	-0.18	0.469925	0.734155
COMPOUND_438_2308a10_461539	Rp	+	438.2308	10.46				(877.4538, 136.02)(878.4435, 114.16)(879.4653, 300.47)(880.4879, 203.09)(881.4561, 329.29)(915.4216, 725.44)(916.4132, 367.52)(917.431, 560.47)(461.2197, 956.23)(899.4589, 783.31)(477.1822, 109.74)(478.1783, 2334.71)(479.1802, 1156.39)(439.2374, 10668.49)(440.2414, 2558.73)(441.243, 1352.21)(442.2304, 111.11)	40234.62	29571.09	32975.5	14116.68	19279	8599.527	31332.09	15158.47	0.18	0.432834	0.537937	0.71	0.01001	0.153413
COMPOUND_439_3042a10_511937	Rp	-	439.3042	10.51				(913.5444, 1569.37)(552.2895, 524.01)(438.298, 6971.59)(439.2995, 2012.91)(440.3017, 424.98)(441.3068, 76.11)(484.2949, 715.77)(485.2901, 262.07)(991.5599, 1013.96)	28838.65	10672.68	30153.36	14355.89	33359	7545.421	31228.11	7092.894	-0.93	6E-05	0.000196	-0.26	0.311507	0.617667
COMPOUND_439_3074a10_509746	Rp	+	439.3074	10.51				(917.5848, 4687.16)(462.2946, 1656.84)(463.2975, 476.15)(901.5968, 1432.88)(478.258, 641.42)(479.2541, 349.78)(440.314, 25754.2)(441.3174, 6015.8)(442.3201, 1648.36)	51169.38	27220.76	43659.59	24813.8	53774.33	25971.12	39980.38	8125.075	-1.12	1E-06	4.98E-06	0.08	0.759716	0.896853
COMPOUND_440_2145a10_688315	Rp	-	440.2145	10.69				(925.4103, 286.77)(926.4104, 269.51)(927.4138, 306.02)(439.2078, 7333.53)(440.2089, 2307.25)(441.2111, 1724.02)(879.3867, 361.18)(880.385, 348.24)	19057.72	4792.49	20880.25	6610.861	18495.92	5689.36	21426.1	6160.303	-0.06	0.833977	0.884235	-0.04	0.892563	0.953702
COMPOUND_441_1583a10_579119	Rp	-	441.1583	10.58				(927.2968, 187.98)(928.3009, 216.8)(929.3054, 165.07)(440.1496, 4494.43)(441.1498, 1703.67)(442.1536, 827.66)(443.1462, 58.33)(881.3337, 341.76)(882.3418, 181.97)(883.3135, 274.17)(884.3407, 232.72)(486.1438, 570.98)(487.1492, 170.77)(488.1499, 5488.77)(489.1506, 1800.02)(995.3034, 237.49)(996.3258, 146.09)	24046	9172.581	24806.51	10890.67	55163.73	31708.69	47123.36	24917.42	0.61	0.029891	0.056095	0.18	0.580961	0.794368
COMPOUND_442_2422a4_7021737	Rp	+	442.2422	4.70				(465.2317, 2359.26)(466.2345, 574.37)(467.2168, 273.53)(468.2108, 29.12)(907.4814, 84.04)(481.2047, 684.7)(482.1994, 198.89)(483.206, 69.49)(460.2765, 104341.76)(461.2797, 22452.09)(462.2816, 4440.82)(463.2854, 769.94)(464.2883, 92.75)(443.2494, 16494.92)(444.2528, 3776.75)(445.2531, 769.54)(446.258, 142.35)	144487.2	214000.4	115581.7	171290.6	131898.7	183424.3	108890.1	159953.3	0.22	0.460381	0.56531	0.18	0.623601	0.823156
COMPOUND_443_1729a10_855437	Rp	-	443.1729	10.86				(921.3042, 487.41)(922.3327, 325.0)(442.1657, 23276.35)(443.168, 7814.18)(444.1688, 2588.11)(445.1688, 475.54)(885.3307, 664.47)(886.3413, 542.55)(887.3337, 606.88)	133563.3	19086.61	121977.9	22268.39	109798.8	24777.72	107948.3	15392.98	-1.19	1.27E-05	4.98E-05	0.53	0.080258	0.339484
COMPOUND_444_1141a11_330682	Rp	+	444.1141	11.33				(889.2099, 915.02)(467.1012, 405.49)(911.2383, 2037.41)(462.147, 5047.96)(463.1479, 2123.44)(464.1466, 1603.11)(465.1463, 220.49)(445.1192, 2250.26)(446.1205, 1091.13)(447.1224, 625.16)(906.2363, 2098.09)	31805.79	5229.028	31826.78	11102.77	41977.5	7680.535	44074.42	7401.817	1.03	4.48E-05	0.00015	0.39	0.163556	0.47785
COMPOUND_444_3616a11_450504	Rp	+	444.3616	11.45				(889.7006, 1300.37)(890.698, 741.24)(927.6604, 1085.31)(928.6523, 1283.08)(929.6587, 1094.31)(467.3606, 529.13)(468.377, 230.11)(911.6745, 1843.21)(445.3689, 126683.3)(446.3725, 39113.12)(447.3754, 6933.16)(448.378, 1203.09)(906.7938, 583.83)(907.7863, 281.43)(908.7861, 254.73)	222128.3	149900	227093.8	142373.4	88401.3	90130	122868.7	104785.2	-0.97	3.78E-05	0.00013	-0.32	0.229404	0.535521
COMPOUND_444_3617a12_4358635	Rp	+	444.3617	12.44				(462.3977, 514.82)(463.3918, 235.22)(445.3678, 24485.73)(446.371, 7764.98)(447.3752, 1702.27)(448.3817, 290.87)	130653	22462.37	116443.7	17635.12	128634.7	11042.52	131674.4	19668.4	0.10	0.717058	0.792741	0.67	0.037723	0.259794
COMPOUND_444_3621a11_444465	Rp	+	444.3621	11.44				(889.6995, 1407.93)(890.6989, 751.71)(927.688, 1178.98)(928.6692, 1029.11)(929.6829, 941.8)(467.3523, 536.02)(468.3608, 238.88)(911.7141, 581.4)(445.3687, 187967.97)(446.372, 58406.98)(447.375, 10426.29)(448.3793, 1561.79)(906.778, 516.2)(907.7883, 136.26)(908.7549, 606.37)	#DIV/0!	#DIV/0!	#DIV/0!	#DIV/0!	#DIV/0!	#DIV/0!	#DIV/0!	#DIV/0!	-0.96	0.000126	0.000383	0.00	0.999691	0.999832
COMPOUND_444_3931a10_82522	Rp	+	444.3931	10.83				(467.3793, 760.87)(911.7762, 2106.0)(483.3673, 1439.28)(484.3775, 849.77)(485.3538, 379.28)(445.4017, 154659.14)(446.4047, 41910.83)(447.4063, 7415.87)(448.4042, 1073.12)(906.8492, 14338.2)(907.8527, 9041.42)	119179.6	55538.81	137887.5	62043.24	202344.6	145068	214058.5	131621.6	0.81	0.003982	0.009198	-0.07	0.822436	0.922833
COMPOUND_444_3939a10_758215	Rp	+	444.3939	10.76				(889.8027, 827.87)(911.8194, 1173.54)(462.4117, 207.59)(483.3594, 931.36)(484.3602, 1077.64)(485.3612, 658.28)(445.4018, 104334.64)(446.4048, 29261.63)(447.4064, 5404.88)(448.412, 698.24)(449.4139, 84.46)(906.8481, 10545.87)(907.8522, 7270.59)(908.8554, 2629.66)	158077.8	75383.34	180302.9	74749.2	247299.5	172448.5	253745.8	153021.9	0.74	0.010908	0.022872	-0.19	0.565273	0.785799

									1st trimester				3rd trimester									
									Control		Pre-eclampsia		Control		Pre-eclampsia		Time			Interaction		
Compound	Column	Ion_mode	Mass	Retention_	Identification	ID_level	MSMS_spectra	CompositeSpectrum	Mean	SD	Mean	SD	Mean	SD	Mean	SD	Value	raw p	FDR_p	Value	raw p	FDR_p
COMPOUND_445_1025a9_919444	Rp	-	445.1025	9.92				(558.0926, 588.78)(559.0852, 793.96)(480.0687, 6260.76)(481.0739, 1726.62)(482.0666, 5659.47)(483.07, 1733.51)(484.0647, 1938.97)(485.0669, 626.17)(486.0616, 447.51)(935.2325, 1445.08)(444.0926, 13731.19)(445.0972, 4691.19)(446.0918, 8768.16)(447.0968, 4001.13)(448.0954, 1878.01)(889.1787, 319.62)(890.1843, 270.57)(891.1747, 1469.28)(1003.2136, 697.96)	52357.79	24153.89	58206.91	27089	59683	10343.46	56611.17	26673.86	-1.03	1.42E-05	5.5E-05	-0.15	0.560247	0.783425
COMPOUND_445_3677a10_910512	Rp	+	445.3677	10.91				(891.7126, 1363.03)(468.3656, 885.43)(469.3527, 275.07)(470.3477, 152.17)(463.3965, 665.93)(446.3753, 22432.34)(447.3797, 7292.81)(448.3836, 1976.69)(449.3914, 261.63)	41620.91	20909.28	40296	17969.24	129596.5	47788.06	122279.7	40348.36	1.37	6.75E-11	1.1E-09	0.02	0.938276	0.97184
COMPOUND_446_339a10_843731	Rp	-	446.3390	10.84				(559.3352, 1517.07)(481.3085, 418.36)(482.2939, 2612.31)(445.3315, 10011.88)(446.3351, 3152.71)(447.3441, 1305.19)(448.3509, 238.5)(491.3316, 784.45)(492.3417, 565.48)(493.3277, 77.51)(1005.6961, 298.54)(1006.671, 734.49)	74531.92	30643.83	81768.46	30279.15	99142.07	30369.1	86077.3	33043.57	0.78	0.001274	0.00325	-0.69	0.011997	0.168863
COMPOUND_447_2029a10_993175	Rp	-	447.2029	10.99				(929.3517, 614.92)(560.182, 1017.61)(561.1773, 421.49)(562.1751, 2398.64)(563.1681, 982.26)(564.1691, 1480.0)(565.1702, 550.39)(482.1654, 6156.71)(939.3529, 328.89)(940.3711, 236.97)(446.1972, 6937.68)(447.1996, 1579.71)(448.2046, 671.21)(449.2019, 99.61)(492.194, 16519.94)(893.4143, 394.66)(894.4113, 376.02)(895.4223, 439.55)(1007.3465, 197.65)(1008.365, 165.01)(1009.3668, 210.54)	56947.64	31674.83	52370.93	26928.1	60183.33	25565.46	56294.2	26747.46	-0.24	0.423908	0.528595	0.31	0.385724	0.687856
COMPOUND_447_2037a11_014076	Rp	-	447.2037	11.01				(929.3501, 636.6)(560.1794, 786.95)(561.1802, 525.33)(562.178, 2331.33)(563.1738, 1010.07)(564.171, 1799.22)(565.1731, 334.4)(482.1655, 5482.66)(939.3692, 300.1)(940.3982, 283.03)(446.197, 7572.59)(447.1992, 1868.97)(448.1991, 501.47)(449.2046, 79.94)(492.1931, 2725.0)(893.4133, 509.13)(894.4277, 353.43)(895.4393, 303.65)(1007.4456, 199.76)(1008.4242, 248.9)(1009.4058, 179.87)	36475.21	14990.51	39282.5	19535.5	44734.85	22397.12	49170.22	21544.39	0.33	0.259268	0.356019	0.17	0.623966	0.823156
COMPOUND_448_1408a8_923512	Rp	-	448.1408	8.92				(561.1026, 310.53)(562.1076, 205.56)(447.134, 9936.1)(448.1373, 1831.21)(449.1396, 722.05)	36034.44	3302.194	36908.24	4028.59	31466.75	3515.997	30050.79	3060.67	-0.46	0.091577	0.150974	0.15	0.632478	0.828105
COMPOUND_449_3129a8_110696	Rp	-	449.3129	8.11				(484.2819, 2984.0)(485.286, 1120.73)(486.2794, 947.65)(562.2969, 10453.46)(563.2982, 3887.86)(448.3056, 1057400.9)(449.3097, 281169.97)(450.3124, 48557.41)(451.3152, 6504.77)(897.6204, 90366.41)(898.623, 52123.88)(899.627, 16158.52)(900.6278, 3308.23)(901.6285, 611.77)	71804.43	85517.9	50464.24	54596.17	79972.09	181240.7	258178.5	1517567	-0.39	0.116699	0.186408	-0.15	0.608775	0.812181
COMPOUND_449_3136a9_239955	Rp	-	449.3136	9.24				(562.2919, 3858.22)(563.2919, 1334.43)(564.2762, 3402.87)(565.2804, 992.25)(448.3062, 406948.28)(449.3097, 116274.84)(450.3124, 18429.24)(451.3114, 3416.19)(452.3092, 344.47)(897.62, 14728.94)(898.6236, 8476.66)(899.6257, 2891.02)(900.6281, 637.47)	174282.8	135955.4	155921.9	96943.81	197152.9	123549.3	287929.6	419785.6	0.00	0.989436	0.995427	0.17	0.564808	0.785799
COMPOUND_449_3138a9_090611	Rp	-	449.3138	9.09				(562.2956, 1764.88)(563.2945, 485.59)(564.2742, 1097.49)(565.272, 391.94)(566.2739, 104.33)(484.2804, 2923.45)(485.2839, 990.29)(486.2804, 997.79)(487.2809, 197.82)(448.3064, 143047.62)(449.3096, 41263.54)(450.3126, 7430.24)(451.3105, 1027.05)(452.3076, 136.92)(897.6208, 30603.72)(898.6238, 17227.43)(899.6272, 5159.73)(900.6292, 1476.19)(901.6253, 283.77)	433764.2	329882.2	373668.9	234989.2	457001.1	343034.7	441020.1	563393.3	0.06	0.839991	0.888104	-0.20	0.545903	0.77815
COMPOUND_449_3143a0_9564283	Hilic	+	449.3143	0.96				(899.6363, 17919.18)(900.6394, 10658.91)(901.6425, 3291.92)(902.6455, 866.93)(903.6459, 202.13)(472.3035, 31094.47)(473.3072, 9400.9)(474.3093, 1619.66)(475.3111, 316.61)(921.6179, 46035.23)(922.6208, 27115.62)(923.6245, 8525.56)(924.6267, 1956.77)(925.6282, 536.23)(467.3488, 891.74)(468.3516, 337.67)(450.3223, 151678.39)(451.3256, 43684.09)(452.3286, 7386.77)(453.3315, 959.16)(916.6726, 1067.25)	#DIV/0!	#DIV/0!	#DIV/0!	#DIV/0!	#DIV/0!	#DIV/0!	#DIV/0!	#DIV/0!	0.45	0.100727	0.163781	0.00	0.988653	0.99433
COMPOUND_449_3155a9_243959	Rp	+	449.3155	9.24				(899.6366, 29427.3)(900.6395, 17442.19)(901.641, 5738.53)(902.6448, 1176.5)(903.6457, 297.57)(937.5828, 2009.12)(938.5871, 1444.93)(939.5895, 540.29)(472.3034, 7745.2)(473.3055, 2407.59)(474.3007, 748.37)(475.3117, 119.56)(921.618, 21455.2)(922.6218, 12416.98)(923.6222, 4159.39)(924.6212, 1362.09)(925.6176, 318.99)(488.2712, 14410.39)(489.2752, 4482.73)(490.2799, 1358.74)(450.3227, 460478.25)(451.3262, 131858.67)(452.3286, 22545.83)(453.3332, 3170.13)	130347.9	102876.3	110670.3	66646.11	154767.1	98757.64	255214.2	446159.5	0.05	0.841964	0.888937	0.19	0.52371	0.766338
COMPOUND_449_3517a11_066475	Rp	+	449.3517	11.07	ACar 20:3	2	10eV: 60.0812(100), 450.3234(4), 309.1472(2)	(921.7001, 1624.15)(450.3594, 60966.14)(451.3628, 17886.59)(452.3661, 3225.59)(453.3679, 557.82)(916.7305, 3709.03)	136900.5	34593.8	133787.3	36095.61	208134.6	45688.96	208053.4	54463.65	1.35	2.89E-07	1.66E-06	-0.04	0.896049	0.954308
COMPOUND_450_261a9_890633	Rp	-	450.2610	9.89				(563.238, 1544.31)(564.2379, 473.19)(485.2285, 1543.69)(486.2289, 174.89)(487.224, 342.05)(449.2537, 18698.85)(450.2592, 8779.08)(451.263, 1033.59)(452.2683, 180.65)(899.5161, 2488.42)(900.5179, 1519.24)(901.5227, 552.13)(902.5189, 211.79)	130813.4	60785.94	145557.3	57549	276876.8	75637.42	215289.9	114458.7	1.20	3.59E-05	0.000125	-1.01	0.002419	0.068106
COMPOUND_451_2426a6_665682	Rp	+	451.2426	6.67				(490.21, 345.42)(491.2107, 80.35)(469.2731, 1542.11)(470.2743, 398.06)(452.2494, 5158.63)(453.2532, 1266.1)(454.2578, 289.18)(455.2678, 52.32)	21637.2	4684.758	19665.58	4125.23	21959.08	5820.558	23983.63	5842.819	0.32	0.262006	0.358903	0.23	0.491988	0.748114
COMPOUND_451_2716a9_937766	Rp	+	451.2716	9.94				(474.2549, 2959.97)(475.2578, 771.1)(469.3015, 4186.63)(452.2786, 6402.13)(453.294, 2557.0)(454.2845, 1517.54)(455.2716, 213.38)	33739.38	11791.74	42460.88	21365.49	58133	22235.26	54625.69	25975.62	1.01	4.21E-05	0.000143	-0.16	0.552574	0.780002
COMPOUND_452_2981a11_1870985	Rp	-	452.2981	11.19				(939.572, 795.65)(940.5506, 1112.74)(941.5599, 792.45)(451.2908, 13658.45)(452.2932, 4576.05)(453.2908, 2055.69)(454.2919, 364.21)(497.3214, 143.83)(498.2967, 369.4)	77986.29	56435.45	105293.1	81130.23	152099.2	122972.8	126553.2	111257.3	0.82	0.005942	0.01321	-0.41	0.235744	0.541667
COMPOUND_453_1911a10_7157135	Rp	-	453.1911	10.72				(941.327, 274.32)(942.3239, 192.06)(488.1523, 4725.34)(489.1533, 1448.62)(951.4337, 252.05)(952.4445, 163.14)(452.1858, 9113.65)(453.1922, 3331.13)(454.1844, 2243.28)(455.1721, 201.51)(905.3397, 355.18)	46223.8	10771.43	50348.13	14240.85	32331.3	11792.14	31407.39	15064.15	-0.68	0.007016	0.0154	-0.33	0.268258	0.575898
COMPOUND_453_2852a10_221464	Rp	-	453.2852	10.22	LPE 16:0	2	10 eV: 452.2777(100), 255.2327(47), 196.038(4), 78.9588(2)	(566.2635, 357.54)(567.2576, 229.29)(452.2779, 250282.3)(453.2809, 56654.32)(454.2838, 9989.13)(455.2854, 1396.06)(456.2898, 185.08)(905.5425, 1141.37)(906.5345, 2840.27)(907.5375, 1238.75)	520477.6	303973.9	534886.4	395678.4	248873.4	372721.6	324465.8	332258.4	-1.02	0.000548	0.001512	0.30	0.367317	0.669574
COMPOUND_453_2859a1_5489997	Hilic	+	453.2859	1.55				(907.5813, 119.95)(908.6063, 35.22)(909.5627, 127.37)(476.2845, 1985.8)(477.2765, 62.34)(478.2953, 2443.18)(929.5563, 333.29)(930.5692, 407.68)(471.3177, 124.76)(454.2931, 7903.8)(455.2965, 1896.44)(456.2985, 291.51)	27363.25	6214.642	30801.93	10937.52	23796.07	11943.39	23953.33	11566.33	-0.58	0.048105	0.084701	-0.21	0.543026	0.776977
COMPOUND_453_2862a1_7646052	Hilic	+	453.2862	1.76				(907.5783, 1449.51)(908.585, 803.64)(909.583, 300.23)(910.5835, 20.58)(476.2751, 21255.27)(477.2783, 5198.93)(929.5606, 1849.02)(930.5645, 875.93)(931.5775, 937.98)(932.5794, 600.54)(933.5898, 323.65)(454.2933, 12973.41)(455.2965, 3085.44)(456.3004, 738.71)(457.2912, 137.34)	182634.9	42413.19	210557.7	72040.94	145621.4	80477	155325.2	79036.5	-0.82	0.004443	0.010127	-0.10	0.76301	0.899577
COMPOUND_453_2869a10_280065	Rp	+	453.2869	10.28	LPE 16:0	2	10 eV: 454.2935(100), 313.2762(100), 44.049(95)	(907.5735, 1542.61)(908.527, 5226.52)(909.5337, 2836.85)(910.5409, 3367.13)(945.5485, 450.16)(946.5461, 258.75)(476.2758, 7428.87)(477.279, 1914.78)(478.2898, 492.32)(929.553, 442.63)(930.5668, 264.22)(471.3431, 297.22)(472.3272, 207.72)(454.2943, 152926.77)(455.2973, 35300.49)(456.3002, 6094.35)(457.3066, 1091.21)(458.2992, 147.86)	393011.6	84627.98	402405.6	141149.7	423145.5	225436.6	424688.3	163120.3	-0.14	0.633911	0.723918	0.31	0.388451	0.68827
COMPOUND_453_2879a10_172813	Rp	+	453.2879	10.17				(907.5782, 3709.08)(908.5839, 2158.51)(909.5915, 732.14)(910.6174, 216.73)(945.5558, 757.32)(946.5452, 753.06)(476.2746, 6254.8)(477.2772, 1786.63)(478.2879, 340.15)(929.5546, 590.67)(930.5429, 968.58)(471.3439, 1845.91)(472.3487, 595.23)(454.294, 204890.62)(455.2968, 47510.01)(456.2991, 7442.86)(457.3009, 1291.69)	99734.77	25960.39	116559.2	85459.7	198449.1	247717	157824.7	120433.2	0.08	0.770771	0.833358	0.07	0.836596	0.929001
COMPOUND_453_2898a9_770219	Rp	-	453.2898	9.77				(566.2756, 14686.64)(567.2797, 4786.42)(568.2751, 1611.69)(452.2813, 2116.92)(453.2848, 636.21)(454.2886, 153.64)(455.299, 25.32)(498.2887, 457314.28)(1019.5675, 6130.91)(1020.57, 3732.79)(1021.5688, 1906.93)	128791.4	169070.3	117318.4	106269.1	241904.1	248854.1	522374.9	632289.7	0.37	0.173169	0.257751	0.20	0.544485	0.77754

									1st trimester				3rd trimester				Time			Interaction		
									Control		Pre-eclampsia		Control		Pre-eclampsia							
Compound	Column	Ion_mode	Mass	Retention_	Identification	ID_level	MSMS_spectra	CompositeSpectrum	Mean	SD	Mean	SD	Mean	SD	Mean	SD	Value	raw p	FDR_p	Value	raw p	FDR_p
COMPOUND_454_3013a11_232764	Rp	+	454.3013	11.23				(909.6243, 1557.47)(910.6107, 1208.01)(911.6394, 876.46)(477.2571, 286.29)(478.2509, 111.06)(479.2698, 1170.69)(480.2665, 597.96)(931.6299, 1166.6)(455.3063, 12142.07)(456.3084, 3605.22)(457.3085, 868.21)	21163.86	10295.24	25013.43	10579.23	44358.17	22832.73	39124.45	16731.1	1.08	4.35E-05	0.000146	-0.26	0.383948	0.686377
COMPOUND_455_1286a9_921174	Rp	-	455.1286	9.92				(945.2492, 282.12)(946.2456, 175.26)(947.2612, 167.27)(568.109, 1172.95)(569.1076, 215.67)(570.1048, 822.32)(571.1042, 527.57)(572.104, 452.62)(454.1207, 7846.78)(455.1164, 2434.94)(456.1177, 2710.2)	27648.96	8226.84	28602.33	9929.868	36308	2920.933	29210.56	10550.16	-0.90	0.0001	0.00031	-0.25	0.344614	0.651065
COMPOUND_455_1408a10_597347	Rp	-	455.1408	10.60				(568.1216, 1183.89)(569.1274, 481.02)(570.1194, 995.7)(490.1102, 13728.1)(491.113, 3297.87)(492.1075, 12371.17)(493.1112, 3112.75)(494.1058, 3946.55)(495.1103, 971.56)(496.1063, 698.46)(955.2775, 347.98)(956.2836, 299.5)(454.1339, 34633.83)(455.1371, 9083.12)(456.1313, 22448.43)(457.1316, 5750.03)(458.1279, 4127.81)(459.116, 1836.06)(909.2471, 428.85)(910.2724, 149.08)(911.2403, 265.02)(1023.2259, 595.95)	94734.98	40792.3	89847.85	37510.55	112562.4	41090.63	158908	54680.53	0.39	0.121666	0.193088	0.84	0.005846	0.118614
COMPOUND_455_1723a10_50408	Rp	-	455.1723	10.50				(568.15, 814.52)(569.1515, 287.42)(570.1415, 488.73)(490.1468, 5962.15)(491.1423, 2429.89)(955.2977, 233.9)(956.3164, 435.11)(957.326, 239.51)(454.1646, 7515.92)(455.1669, 2301.33)(456.175, 1021.69)(457.1609, 117.62)(909.3474, 292.07)(910.3496, 202.71)(500.1758, 13242.94)(501.1782, 4066.07)	46377.94	18675.09	46867.07	19731.65	100134.2	22082.65	112303.9	32347.95	0.31	0.237059	0.331794	0.74	0.01915	0.196411
COMPOUND_455_3402a10_948466	Rp	+	455.3402	10.95				(949.6566, 2593.06)(950.6517, 2556.01)(478.3303, 5629.57)(479.3363, 1645.93)(480.3431, 1005.12)(456.3492, 6190.17)(457.351, 2170.67)(458.3505, 925.33)(928.6868, 1409.32)	45278.94	33235.79	40158.6	42896.23	41128.77	11099.65	44740.7	12257.37	0.00	0.99522	0.998768	0.27	0.440796	0.716214
COMPOUND_457_1675a10_659327	Rp	-	457.1675	10.66				(456.1604, 13574.57)(457.1672, 6518.55)(458.1662, 2011.93)(459.172, 335.95)(913.3545, 507.62)	112909.8	17401.9	110728	17975.7	92721.08	33250.2	105279.8	30341.54	-0.80	0.004761	0.010754	0.46	0.156859	0.467257
COMPOUND_457_1902a11_017043	Rp	-	457.1902	11.02				(949.3273, 408.21)(950.3328, 268.8)(951.3531, 280.37)(959.3299, 412.72)(960.3369, 384.12)(961.3518, 319.79)(456.1829, 1979.13)(457.1861, 980.56)(458.1963, 14580.56)(459.1996, 4482.5)(913.373, 529.63)(914.3838, 477.95)(915.3855, 325.04)(1027.3038, 283.12)(1028.3337, 284.11)	44750.94	8013.6	43425.35	8040.38	32502.9	16084.75	34482	10592.72	-0.85	0.00115	0.002954	0.05	0.87176	0.945212
COMPOUND_457_2445a10_789214	Rp	-	457.2445	10.79				(959.4797, 3334.21)(456.2363, 14534.56)(457.2379, 6115.97)(458.2377, 2275.65)(459.2386, 357.05)(1027.487, 1711.81)	31580.52	14072.86	32971.29	15456.78	57723.29	21337.7	67673.89	20304.74	1.13	1.15E-06	5.64E-06	0.31	0.218258	0.526249
COMPOUND_458_2359a11_579628	Rp	-	458.2359	11.58				(951.5203, 408.98)(952.4734, 559.47)(953.4772, 383.65)(457.2283, 11137.64)(458.2307, 3194.44)(459.2253, 20425.6)(460.2272, 5786.91)(461.2219, 8185.82)(462.2253, 2201.45)(463.2209, 1618.84)(915.4657, 416.1)(916.4555, 414.99)(917.4655, 698.44)(1029.497, 667.07)(1030.5043, 1267.43)	66015.54	15227.08	70379.85	15232.66	71942.36	21087.6	66501.73	22600.21	0.43	0.124933	0.196611	-0.80	0.018156	0.195632
COMPOUND_458_2437a1_3971556	Hilic	+	458.2437	1.40				(917.5243, 309.66)(481.2325, 3874.78)(482.2474, 1489.83)(483.2504, 744.79)(939.4746, 296.96)(940.5002, 262.9)(476.2798, 1177.52)(477.2891, 449.92)(478.2938, 1012.58)(459.2509, 4518.56)(460.2687, 2070.62)(461.2716, 515.96)(934.5295, 319.49)	61025.43	41172.33	69269.66	37849.84	81611.45	49237.25	80209.56	25332.11	0.50	0.071339	0.120709	-0.17	0.604434	0.810123
COMPOUND_458_4091a11_081561	Rp	+	458.4091	11.08				(917.8398, 1232.28)(918.8344, 809.83)(919.8211, 274.75)(955.7604, 115.75)(481.4111, 347.45)(939.7895, 940.35)(459.4161, 23194.81)(460.4188, 6754.1)(461.4188, 1241.02)(462.4104, 225.15)(934.8894, 967.75)(935.8738, 815.06)(936.8909, 252.71)	44082	25837.03	32195.69	13120.35	73895.25	59952.42	75315.16	37617.03	0.54	0.038954	0.070689	0.46	0.14256	0.448182
COMPOUND_458_4091a11_032743	Rp	+	458.4091	11.03				(917.8356, 3886.7)(918.8293, 1843.93)(919.8083, 530.35)(955.83, 232.6)(481.374, 375.76)(939.8035, 1048.05)(459.4165, 16010.02)(460.4201, 4527.2)(461.4235, 1197.59)(462.4197, 182.63)(934.8324, 185.29)(935.8454, 1081.52)(936.861, 351.2)	38199.26	20773.94	28733.71	12539.22	46860.62	29724.82	51148.41	28356.74	0.46	0.092995	0.153089	0.41	0.203491	0.5149
COMPOUND_459_9957a10_144271	Rp	+	459.9957	10.14				(482.9902, 155.33)(483.9916, 305.09)(498.9674, 90.92)(499.9677, 228.5)(461.0042, 6061.39)(462.0056, 762.2)(462.9907, 1014.57)(464.0032, 119.68)	43910.52	19002.43	34782.5	13550.54	49800	11730.96	53224.09	16914.46	0.23	0.393487	0.497827	0.78	0.020103	0.199116
COMPOUND_463_2766a9_4074135	Rp	+	463.2766	9.41				(464.2838, 22592.01)(465.2875, 6400.58)(466.287, 1962.45)(467.2826, 362.06)	50506.83	37765.59	53623.94	36754.1	274236.9	617776	148436.8	199837.7	0.57	0.025244	0.0483	-0.04	0.896677	0.954308
COMPOUND_463_3036a10_536387	Rp	-	463.3036	10.54				(961.5754, 4481.01)(962.5544, 3271.76)(576.2789, 564.88)(577.2751, 257.28)(462.2981, 72822.43)(463.3014, 18492.07)(464.3077, 4763.66)(925.5556, 3694.96)	239965.7	72517.91	247174.3	82713.7	99067.56	92118.98	107383	89977.73	-1.19	1.27E-07	7.8E-07	-0.23	0.335126	0.63958
COMPOUND_463_3066a1_3849862	Hilic	+	463.3066	1.38				(927.6145, 236.87)(928.6462, 521.98)(486.296, 4285.12)(487.2995, 1123.58)(488.3111, 2825.3)(949.5894, 276.13)(481.3323, 364.52)(464.3141, 12368.54)(465.3179, 3226.68)	47263.45	15111.23	49605.74	18073.72	21616.63	19001.16	17717.86	18327.24	-1.21	1.66E-07	1.01E-06	-0.19	0.426584	0.705867
COMPOUND_463_3083a10_537336	Rp	+	463.3083	10.54				(927.6204, 560.9)(928.6171, 865.94)(929.6275, 700.29)(965.5896, 1036.7)(966.5809, 1179.65)(967.5915, 897.18)(486.2963, 4236.74)(487.2951, 1453.91)(488.3008, 568.0)(949.5746, 1507.81)(950.5701, 1785.3)(951.587, 903.34)(502.294, 591.58)(503.2973, 323.32)(464.3146, 68359.01)(465.3177, 17323.0)(466.3235, 4779.5)(467.3166, 692.57)(944.6288, 542.55)(945.6153, 860.91)	208666.9	67837.42	204597.1	64239.7	81871.75	90674.87	65212.43	76234.07	-1.22	7.38E-07	3.83E-06	-0.12	0.642189	0.834513
COMPOUND_465_1576a9_919288	Rp	-	465.1576	9.92				(464.1503, 11359.13)(465.1535, 3844.21)(466.1581, 1044.58)(510.1435, 402.0)(511.1442, 214.64)(512.148, 221.51)	34855.47	10328.76	35781.46	16516.02	47881.67	11805.18	32558.08	15105.82	-1.00	1.61E-05	6.15E-05	-0.22	0.401011	0.690408
COMPOUND_465_1656a10_594962	Rp	-	465.1656	10.59				(965.253, 392.68)(578.1296, 745.42)(579.1207, 951.36)(580.1141, 654.92)(975.265, 272.6)(976.2896, 259.36)(464.1626, 28229.14)(465.166, 6254.74)(466.1603, 8507.07)(467.1529, 2707.12)(468.1418, 305.75)(929.2801, 274.67)(930.2591, 296.45)(931.2806, 215.43)(1043.3484, 250.41)(1044.3738, 106.02)	63985.44	23061.32	58810.87	19744.68	46084.64	10423.04	55727.46	19746.4	-1.05	0.000272	0.000786	0.92	0.005166	0.111932
COMPOUND_465_1696a10_598275	Rp	-	465.1696	10.60				(965.2708, 368.79)(578.1629, 876.96)(579.149, 74.0)(580.1731, 2785.03)(975.3522, 97.09)(976.3481, 261.17)(464.1626, 28229.14)(465.166, 6254.74)(466.1603, 8507.07)(467.1529, 2707.12)(468.1418, 305.75)(929.3047, 247.24)(930.3148, 233.82)(931.279, 192.9)(1043.2965, 122.87)(1044.2784, 37.76)	65656.58	20765.8	62098.24	18544.62	42674	11268.59	56585	18470.45	-1.16	6.25E-05	0.000203	0.81	0.01305	0.173893
COMPOUND_465_3087a8_598774	Rp	-	465.3087	8.60	Glycocholic acid	1	10 eV: 464.3013(100), 33.827(2), 267.14(2)	(578.2891, 4487.23)(579.2895, 1403.59)(580.27, 5961.9)(464.3006, 855771.94)(465.3046, 229961.73)(466.3072, 39754.06)(467.3098, 5745.21)(468.3129, 614.58)(929.6106, 15694.38)(930.6134, 9083.26)(931.6169, 2978.67)(932.6207, 814.52)(933.6169, 176.25)	174257.6	197812.9	142711.1	96118.75	808732.5	1452310	474640.9	659137.7	1.02	3.89E-05	0.000133	-0.08	0.770221	0.902789
COMPOUND_465_309a8_606094	Rp	+	465.3090	8.61				(931.6281, 385080.12)(932.6318, 221179.42)(933.6336, 66820.21)(934.6356, 15303.1)(935.6389, 2895.6)(936.6382, 499.45)(969.5737, 2572.78)(970.5763, 2006.54)(971.5767, 760.02)(488.2974, 6701.39)(489.3041, 2152.0)(490.2878, 952.19)(491.2742, 113.72)(953.608, 17440.76)(954.6113, 10153.45)(955.6092, 4077.2)(956.6058, 1281.66)(957.6011, 363.3)(504.2667, 15742.83)(505.2689, 5231.96)(506.269, 1381.44)(483.3513, 819.96)(484.3582, 379.18)(466.3172, 73770.11)(467.3202, 20452.21)(468.3235, 3912.56)(469.3241, 720.18)(470.3126, 98.67)(948.6514, 6508.12)(949.654, 3851.83)(950.6489, 1441.33)	100054.5	131888.5	65737.95	46791.57	612528.9	1436408	316524	552559.1	1.06	6.12E-05	0.0002	-0.16	0.579355	0.793617
COMPOUND_465_3202a10_783273	Rp	-	465.3202	10.78				(965.5914, 683.41)(464.3139, 73669.59)(465.3161, 21639.49)(466.3173, 4215.27)(929.6315, 539.23)	157402.5	46861.34	161602.9	55104.66	95709.5	3834.64	100362.3	12894.58	-1.03	1.62E-06	7.78E-06	-0.19	0.419053	0.69956
COMPOUND_465_3207a10_592524	Rp	+	465.3207	10.59				(488.3117, 1041.77)(489.3157, 342.97)(490.3145, 412.46)(953.6422, 868.33)(954.6236, 649.41)(466.3301, 11557.39)(467.3327, 2988.73)(468.3231, 1117.89)	21739.73	9411.86	22262.83	10011.92	24357.33	1081.692	24611.11	6168.088	-0.77	0.000937	0.002458	-0.07	0.783467	0.909997
COMPOUND_465_3241a10_781681	Rp	+	465.3241	10.78				(931.6081, 1067.31)(932.6335, 861.54)(488.3134, 6019.13)(489.3201, 1808.21)(490.3244, 463.74)(953.6498, 1381.24)(954.6284, 1201.51)(504.2916, 1969.99)(483.3636, 1385.93)(466.3299, 87836.08)(467.3334, 22164.67)(468.3369, 4110.92)	166018	52615.5	158808.8	58983.84	51364.69	46836.25	40144.08	31253.81	-1.41	1.27E-09	1.37E-08	-0.01	0.960272	0.981885
COMPOUND_466_2553a8_315677	Rp	-	466.2553	8.32				(465.2487, 3292.4)(466.2515, 1103.42)(467.2523, 234.58)(468.2504, 44.55)	21259.56	8571.036	22393.81	8519.029	18183.09	8366.817	26797.56	26684.77	-0.52	0.027861	0.052859	-0.03	0.925374	0.966461

									1st trimester				3rd trimester									
									Control		Pre-eclampsia		Control		Pre-eclampsia		Time			Interaction		
Compound	Column	Ion_mode	Mass	Retention_	Identification	ID_level	MSMS_spectra	CompositeSpectrum	Mean	SD	Mean	SD	Mean	SD	Mean	SD	Value	raw p	FDR_p	Value	raw p	FDR_p
COMPOUND_467_1709a10_715968	Rp	-	467.1709	10.72				(969.2925, 305.07)(970.274, 236.62)(580.174, 4592.66)(581.1736, 2046.07)(979.3146, 295.5)(466.166, 8121.47)(467.1693, 2704.32)(468.1671, 1044.91)(469.1743, 190.24)(933.3435, 395.52)(934.3408, 234.73)(935.361, 303.46)(936.3526, 270.72)(512.1785, 340.82)(513.1772, 85.69)(514.1658, 2577.94)	60983.09	10925.54	61832.07	18066.8	50566.78	19220.54	56263.03	16644.83	-0.94	0.000778	0.002076	0.27	0.404114	0.6912
COMPOUND_467_3017a1_1443499	Hilic	+	467.3017	1.14	LPC 14:0 isomer	2	10 eV: 468.3079 (100), 184.0733 (80)	(935.6091, 2432.96)(490.2911, 47804.33)(491.2943, 12196.35)(492.305, 4272.44)(957.5901, 1983.39)(468.3092, 52551.93)(469.3124, 13455.88)(470.3147, 2272.53)(471.3197, 431.47)	164880.9	463434.7	129110.4	111653.3	112388.9	35189.9	87577.63	43050.65	0.58	0.042152	0.075398	-1.16	0.000803	0.045795
COMPOUND_467_3017a9_617346	Rp	+	467.3017	9.62	LPC 14:0 isomer	2	10 eV: 468.3131(100), 184.0753(15), 104.1084(11)	(490.2931, 2986.0)(491.2955, 959.48)(492.3023, 265.17)(485.3461, 1038.09)(486.3463, 626.19)(468.3094, 15033.94)(469.3132, 3740.26)(470.3134, 827.35)(471.3131, 158.5)	97803.55	46461.07	128636.6	74630.19	101046.6	41699.43	83402.71	43616.55	0.01	0.962678	0.978317	-0.61	0.056055	0.29488
COMPOUND_467_3025a10_470193	Rp	+	467.3025	10.47				(935.6177, 403.98)(936.6313, 314.46)(937.6366, 539.62)(973.5746, 838.92)(974.5782, 752.21)(490.2948, 757.87)(491.2948, 300.34)(492.295, 394.55)(957.5686, 865.57)(506.273, 519.53)(507.2801, 243.88)(468.3098, 6954.84)(469.3132, 2152.2)(470.3048, 317.65)(952.6614, 1644.74)(953.661, 1339.82)	50088.33	12834.41	51667.9	16061.07	29439.6	11589.57	27133.03	11234.1	-1.02	3.44E-05	0.00012	-0.04	0.877922	0.949606
COMPOUND_467_3026a1_2767569	Hilic	+	467.3026	1.28	LPC 14:0	2	10 eV: 468.3088 (100), 184.0734 (25), 104.1073 (15)	(935.6102, 4465.51)(936.6133, 2455.77)(937.6177, 760.34)(938.5983, 294.97)(490.291, 73432.02)(491.2943, 18967.02)(492.3048, 6841.32)(957.592, 3968.97)(958.5948, 2274.64)(959.5991, 857.8)(960.6159, 211.71)(485.3317, 641.79)(468.3086, 701271.56)(469.3127, 163248.05)(470.3152, 28435.16)(471.3176, 3782.26)(472.3192, 498.36)	1147623	666444.1	1742892	1101313	1159377	510672.8	932919.6	521910.5	0.07	0.783743	0.842477	-1.02	0.002282	0.067469
COMPOUND_467_303a9_7604685	Rp	+	467.3030	9.76	LPC 14:0	2	10eV: 468.3104(100), 104.1076(25), 184.077(17)	(935.6113, 6131.02)(936.6152, 3084.25)(937.6173, 1071.12)(938.5954, 151.75)(490.2909, 9423.74)(491.2941, 2656.38)(492.307, 1023.06)(493.3086, 169.41)(957.6054, 1138.52)(958.587, 557.04)(959.5726, 904.68)(960.573, 451.68)(506.2644, 3146.84)(507.2661, 1183.67)(508.2723, 1194.33)(468.3103, 737854.94)(469.3141, 171333.94)(470.3163, 28110.07)(471.3199, 4429.43)(472.3109, 418.2)	907722.4	499039.5	1293387	799978.1	945585.1	399904.3	769146	447486.1	0.08	0.770044	0.833358	-0.95	0.002714	0.073638
COMPOUND_468_1649a9_920559	Rp	-	468.1649	9.92				(467.1576, 8112.35)(468.1607, 1714.87)(469.1558, 3446.72)(470.1535, 1030.7)(471.1617, 792.67)(513.175, 715.8)(514.1628, 164.34)	26846.57	7750.655	27775.7	7640.995	26865	3433.895	31984.7	9942.689	-1.13	2.34E-06	1.07E-05	-0.04	0.880417	0.950612
COMPOUND_468_2145a9_028728	Rp	-	468.2145	9.03				(467.2075, 20390.52)(468.2108, 4933.65)(469.2114, 1751.87)(470.2102, 281.77)	29299	9888.931	33679.2	15119.39	100275.9	47380.98	83096.4	36756.9	1.71	4.43E-13	2.56E-11	-0.44	0.04669	0.275249
COMPOUND_468_2155a8_328927	Rp	-	468.2155	8.33				(467.2078, 6304.44)(468.2115, 1721.58)(469.2099, 559.57)(470.2137, 96.57)	19330.8	6337.55	19725.05	7534.179	38634.5	10801.23	32294.38	12464.14	1.43	1.76E-07	1.06E-06	-0.51	0.076861	0.332868
COMPOUND_468_3584a11_773093	Rp	+	468.3584	11.77				(975.7167, 794.14)(959.7309, 1211.85)(960.7418, 1248.73)(469.3668, 12206.89)(470.3692, 4328.61)(471.3709, 848.69)(472.3797, 149.11)	52954.3	15137.96	53054.63	13862.92	23692.3	14325.75	21045.97	12929.04	-1.31	4.77E-09	4.23E-08	-0.19	0.409663	0.694731
COMPOUND_469_1018a10_011417	Rp	-	469.1018	10.01				(973.1678, 513.81)(504.0688, 2101.59)(505.0731, 764.43)(506.0662, 2156.68)(507.0678, 692.3)(508.0632, 693.82)(509.0779, 219.28)(983.1933, 417.34)(984.2173, 306.02)(468.092, 9589.48)(469.0973, 3958.86)(470.0896, 6458.42)(471.0952, 2687.32)(472.0911, 1384.63)(514.0844, 355.0)(937.1867, 659.91)(938.1991, 509.69)(939.2092, 488.95)(1051.2279, 425.78)(1052.2241, 418.75)	34970.02	16205.52	39666.33	16131.12	41931.25	19801.39	56005.6	23137.29	-0.85	0.000521	0.00144	-0.20	0.465718	0.732912
COMPOUND_470_0011a9_978315	Rp	+	470.0011	9.98				(492.9948, 183.97)(494.0136, 415.75)(488.0574, 167.03)(508.9833, 160.5)(471.0082, 11155.62)(472.0109, 1168.21)(472.9929, 1725.81)(474.0027, 211.42)(475.004, 550.74)	61593.83	23642.75	51015.26	18486.03	62760.62	11090.56	66216.27	19468.09	0.09	0.74521	0.814666	0.78	0.025023	0.219303
COMPOUND_470_2331a7_9199367	Rp	-	470.2331	7.92				(469.2255, 6065.79)(470.2293, 1969.3)(471.2329, 869.09)	13915.16	3129.989	12709.57	1945.359	18639.31	9726.876	17280.95	7060.772	1.12	2.11E-07	1.23E-06	-0.50	0.02711	0.226687
COMPOUND_471_4299a11_762702	Rp	+	471.4299	11.76				(943.9028, 455.19)(944.909, 405.2)(981.8043, 470.37)(494.412, 770.88)(489.4477, 329.02)(510.4159, 444.02)(472.4367, 28438.21)(473.44, 8866.34)(474.4419, 1793.52)	91678.85	32687.25	74013.93	33032.31	91311.14	23867.08	99401.72	30612.89	0.04	0.88663	0.923966	0.83	0.014278	0.179521
COMPOUND_472_3141a11_110396	Rp	+	472.3141	11.11				(495.3101, 685.52)(473.3197, 7591.19)(474.323, 2239.05)(475.3293, 685.42)(962.6642, 6099.84)(963.6672, 5891.05)	30996.14	9905.527	26589.38	8071.212	45293.4	9624.523	46632.8	14167.93	0.44	0.105433	0.170449	0.36	0.266002	0.575669
COMPOUND_473_2418a9_516804	Rp	+	473.2418	9.52				(474.2494, 5406.14)(475.2527, 1645.91)(476.2546, 362.67)(477.2516, 70.36)	39484.42	8245.141	36050.65	6654.149	36166.71	4265.773	38288.28	7053.684	-0.35	0.242391	0.337578	0.68	0.059651	0.300425
COMPOUND_474_1659a9_920882	Rp	-	474.1659	9.92				(587.1383, 541.07)(588.124, 298.12)(509.1374, 648.34)(510.1356, 238.88)(511.1281, 303.86)(473.1624, 5298.44)(474.1647, 1927.95)(475.1589, 914.76)(476.1716, 107.98)(519.1577, 990.66)(520.1685, 261.55)	18048.22	5786.546	20598.08	6584.556	21937.33	6339.429	24796.33	1932.44	-0.76	0.0004	0.00113	-0.51	0.039467	0.261543
COMPOUND_474_4063a12_528065	Rp	-	474.4063	12.53				(509.3753, 2335.0)(510.3772, 819.51)(511.3742, 957.88)(512.3761, 480.14)(513.3736, 80.32)(993.7929, 103.32)(994.7905, 84.9)(473.3991, 24226.61)(474.4025, 8546.94)(475.4053, 1594.51)(476.4085, 268.73)(477.3953, 37.06)(519.4044, 4131.18)(520.4069, 1389.64)(521.4095, 466.18)(522.4132, 90.14)	126604	40314.33	157849.6	45078.52	151205.3	56808.63	143314.3	46612.28	0.45	0.112897	0.181214	-0.66	0.048748	0.279822
COMPOUND_474_4064a12_427342	Rp	-	474.4064	12.43				(509.3756, 3599.3)(510.3787, 1141.81)(511.3743, 1263.96)(512.3754, 508.77)(513.3755, 71.48)(993.7995, 91.87)(994.8178, 93.38)(473.3989, 60688.42)(474.4027, 20750.52)(475.4057, 3694.89)(476.4087, 589.53)(477.4094, 84.41)(519.4044, 8978.74)(520.4076, 2833.01)(521.4106, 611.87)(522.4105, 115.13)	400979.1	93797.4	457723.8	104540.4	387574.9	80133.57	380158	87355.66	-0.09	0.744111	0.814256	-0.78	0.020479	0.200518
COMPOUND_474_4089a12_433876	Rp	+	474.4089	12.43				(497.3961, 6718.44)(498.4006, 2188.38)(499.4005, 476.49)(500.4102, 80.65)(513.3704, 958.84)(514.3736, 428.01)(515.3701, 654.99)(516.3687, 236.41)(492.442, 93049.78)(493.4452, 31171.89)(494.4484, 5951.79)(495.4504, 1020.79)(496.4576, 142.68)(475.4147, 32715.69)(476.4179, 11244.28)(477.4208, 1947.79)(478.4246, 365.27)	684295.5	142997.1	612463.1	151841.8	598334.4	147154.3	625260.2	146674.3	-0.50	0.0823	0.136743	0.59	0.080008	0.339484
COMPOUND_474_6732a10_116064	Rp	+	474.6732	10.12				(950.3576, 227.09)(951.334, 151.77)(952.3405, 235.31)(953.3321, 103.69)(988.3231, 214.7)(989.3218, 155.64)(497.6427, 241.57)(972.3331, 125.27)(973.3555, 98.59)(974.3331, 277.63)(513.6525, 153.78)(475.6787, 13432.51)(476.681, 1659.95)(477.6644, 1166.25)(478.6735, 150.67)(967.4238, 214.51)	56085.1	24173.01	43880.05	17889.02	61720.92	14939.19	67152.35	20200.42	0.21	0.442045	0.547572	0.90	0.006563	0.125256
COMPOUND_475_0068a10_125416	Rp	+	475.0068	10.13				(497.9895, 495.88)(498.9857, 116.72)(476.0133, 7568.28)(477.0116, 616.06)(477.9969, 634.77)	46403.91	21061.28	35777.08	14371.23	50470	8968.202	53275.74	15651.03	0.38	0.173092	0.257751	0.33	0.327174	0.634609
COMPOUND_475_1985a10_596521	Rp	-	475.1985	10.60				(474.1912, 22951.97)(475.1942, 6312.7)(476.196, 1431.08)(477.2068, 283.34)(949.3523, 283.59)(1063.382, 289.93)(1064.3864, 319.91)	64987.92	21760.8	61744.84	23991.82	35761.93	12416.04	47614.51	17354.91	-1.23	2.89E-05	0.000103	0.69	0.03579	0.252664
COMPOUND_475_1986a10_588313	Rp	-	475.1986	10.59				(474.1914, 19308.13)(475.1946, 5114.73)(476.1969, 1418.0)(477.2, 269.98)(949.3523, 283.59)(1063.3455, 277.44)(1064.3207, 190.71)	48638.17	24333.56	53458.04	22280.14	32123.36	13627.54	42559.63	17397.32	-0.75	0.010477	0.022133	0.33	0.332387	0.636996
COMPOUND_475_269a9_859699	Rp	-	475.2690	9.86				(474.2618, 18320.87)(475.2647, 4736.53)(476.2684, 933.38)(477.2667, 181.92)	26983.35	13719.59	30696.95	19979.26	31036	15043.57	26986.12	11449.39	0.35	0.231531	0.326697	-0.45	0.196014	0.507591
COMPOUND_475_2708a9_863308	Rp	+	475.2708	9.86				(498.2596, 1009.41)(499.2625, 354.0)(514.2222, 336.87)(515.2357, 166.72)(493.3154, 777.02)(476.2788, 10906.55)(477.2822, 2922.66)(478.287, 684.88)(479.275, 110.43)	30913.79	17070.33	34046.12	22072.52	32308.21	14568.26	30054.31	12866.36	0.34	0.233793	0.328448	-0.43	0.211327	0.519294
COMPOUND_477_2847a10_117544	Rp	-	477.2847	10.12	LPE 18:2	2	20 eV: 279.2325(100), 476.2777(13), 196.038(6)	(590.253, 707.94)(591.2624, 325.34)(592.2644, 6398.98)(593.2663, 2244.9)(476.2776, 168821.77)(477.2807, 43225.82)(478.2835, 7537.99)(479.2862, 1124.52)(480.2751, 113.73)(953.5634, 3638.41)(954.5656, 1667.38)(955.5678, 545.14)(956.5722, 145.03)	428611.5	203977.7	483956.5	222502.9	446165.2	138251	454623.3	164710.3	0.16	0.588531	0.683887	-0.24	0.504068	0.754687
COMPOUND_477_2847a10_022529	Rp	-	477.2847	10.02				(590.2536, 551.9)(591.2628, 312.15)(592.2658, 4147.5)(593.2697, 1269.97)(476.2775, 173901.7)(477.2806, 43664.4)(478.2831, 7732.17)(479.2872, 1267.49)(953.564, 2067.11)(954.5668, 1125.29)(955.5751, 340.73)(956.5897, 71.37)	99305.49	94443.43	90557.8	87751.14	107094.9	145935.4	146131.3	153122.7	0.01	0.969723	0.982819	0.34	0.341518	0.646301
COMPOUND_477_2859a1_7269133	Hilic	+	477.2859	1.73				(955.578, 977.84)(956.5808, 459.37)(957.5892, 304.23)(500.2754, 12920.63)(501.2784, 3210.47)(502.2905, 7553.92)(977.5588, 983.72)(978.5637, 532.79)(979.5749, 550.94)(980.5781, 264.95)(495.3033, 338.89)(478.2933, 32560.44)(479.297, 8747.36)(480.3076, 9870.99)	152884.3	75418.4	172797.5	77778.42	146165.1	45901.17	152563.8	60167.32	-0.02	0.946278	0.965573	-0.22	0.549364	0.779073

									1st trimester				3rd trimester									
									Control		Pre-eclampsia		Control		Pre-eclampsia		Time			Interaction		
Compound	Column	Ion_mode	Mass	Retention_	Identification	ID_level	MSMS_spectra	CompositeSpectrum	Mean	SD	Mean	SD	Mean	SD	Mean	SD	Value	raw p	FDR_p	Value	raw p	FDR_p
COMPOUND_477_2871a10_094607	Rp	+	477.2871	10.09	LPE 18:2	2	10eV: 44.0493(100), 337.2768(80), 478.3379(12), 263.2394(12)	(955.5785, 4362.88)(956.5818, 2271.32)(957.5855, 906.4)(958.5834, 278.88)(993.5406, 385.59)(994.534, 343.66)(500.276, 7701.32)(501.2789, 2049.77)(977.5618, 320.15)(978.5597, 268.69)(979.5762, 3175.18)(980.5814, 1813.42)(516.2413, 736.37)(517.2494, 239.03)(518.2315, 96.23)(495.3337, 1931.4)(478.2942, 208917.92)(479.2972, 53403.52)(480.3001, 9458.22)(481.3033, 1415.05)(972.6449, 299.75)	288743.8	231607.6	344715.3	246447.4	494400.1	138325.5	468423.8	197594.6	0.79	0.004584	0.010385	-0.13	0.686694	0.854077
COMPOUND_477_2876a10_098582	Rp	+	477.2876	10.10	LPE 18:2 duplicate			(955.577, 1973.99)(956.5834, 1264.86)(957.5877, 439.37)(958.582, 105.39)(993.5625, 371.81)(994.5686, 53.89)(500.275, 7855.6)(501.2797, 2327.42)(977.5631, 315.62)(978.5523, 191.8)(979.5768, 3525.26)(980.5817, 1774.49)(495.3306, 1865.56)(516.2442, 1373.65)(517.2637, 857.48)(518.2371, 107.93)(478.2941, 209498.39)(479.2974, 51041.89)(480.3005, 9994.51)(481.3017, 1275.59)(972.5928, 374.17)	378153.3	257897.8	371149	234553.4	450575.4	321042.4	519064	281588.5	0.15	0.599542	0.693461	0.29	0.404677	0.6912
COMPOUND_477_3221a1_0574329	Hilic	+	477.3221	1.06				(955.6719, 887.5)(500.2985, 255.88)(501.2889, 20.38)(478.3295, 13545.25)(479.3326, 3555.39)(972.678, 648.33)	41510.64	8026.303	45338.76	7925.469	21249.5	6139.086	21852.88	6610.256	-1.41	2.95E-10	3.97E-09	-0.31	0.160245	0.472345
COMPOUND_479_1411a10_46062	Rp	-	479.1411	10.46	no msms			(993.2132, 295.33)(994.1896, 259.73)(592.1299, 751.84)(593.1232, 151.34)(514.1101, 11156.82)(515.1135, 2995.0)(516.1075, 11309.63)(517.1096, 3373.45)(518.1058, 3587.05)(519.107, 1163.76)(520.1093, 1085.15)(478.1344, 19155.57)(479.1387, 4962.55)(480.1313, 12017.74)(481.1333, 3203.56)(482.129, 3482.05)(483.127, 1036.9)(484.1345, 196.38)(524.1307, 582.84)(525.1211, 294.81)	42366.29	21174.75	46542.38	30212.14	74615.56	26540.76	110294.7	54656.77	0.25	0.264942	0.361825	1.04	0.000223	0.020129
COMPOUND_479_2212a11_580742	Rp	-	479.2212	11.58				(478.2143, 5213.71)(479.2171, 1336.79)(480.2133, 2859.34)(481.2146, 1103.06)(482.2066, 1360.25)(957.4533, 538.06)(958.4701, 283.29)(524.2127, 1051.35)	26914.75	9435.771	25028.52	6060.143	37955.36	16961.63	29912.72	15247.04	0.76	0.012541	0.025958	-0.65	0.071771	0.325554
COMPOUND_479_2997a9_937698	Rp	-	479.2997	9.94				(478.2926, 8500.52)(479.2963, 2471.72)(480.301, 557.86)(481.3056, 85.64)	32570.83	11855.33	38530.55	13827.33	29980.85	7016.233	28729	11980.4	-0.13	0.628887	0.720006	-0.73	0.019673	0.198675
COMPOUND_479_3004a10_379412	Rp	-	479.3004	10.38	LPE 18:1	2	10 eV: 478.2937(100), 281.2476(28), 196.0387(3)	(993.5703, 811.82)(994.5725, 580.1)(995.5734, 299.55)(996.5985, 96.15)(592.2784, 511.15)(593.2733, 297.37)(478.2935, 177998.72)(479.2966, 45400.98)(480.3024, 10076.92)(481.3057, 1912.9)(482.312, 271.25)(957.5654, 1138.13)(958.5403, 2677.11)(959.5407, 1407.54)(960.5455, 1884.27)	523891.2	174914.6	530067.8	157261.7	442316.5	114229.8	496023.6	146930.5	-0.50	0.098051	0.160007	0.28	0.422519	0.702743
COMPOUND_479_3004a10_283007	Rp	-	479.3004	10.28				(993.5754, 587.3)(994.5774, 389.48)(995.5855, 265.08)(996.5976, 63.38)(592.2751, 543.03)(593.2723, 334.46)(478.2932, 143278.5)(479.2965, 36593.35)(480.3037, 9041.72)(481.3088, 1640.14)(957.6084, 2090.41)(958.6278, 385.54)(959.6424, 178.44)(960.6376, 68.61)	89859.91	102180.5	110438.1	148895	73834.64	68402.1	68229.35	63307.23	-0.36	0.234003	0.328538	-0.04	0.912739	0.960508
COMPOUND_479_3021a1_6692221	Hilic	+	479.3021	1.67				(959.6095, 481.19)(960.6113, 230.59)(961.6237, 513.75)(962.6278, 279.16)(502.2927, 25582.78)(503.2965, 6740.24)(981.5901, 806.05)(982.5954, 434.33)(983.6069, 857.06)(984.6096, 445.52)(985.6197, 413.97)(480.3087, 15113.82)(481.3123, 3767.88)(482.3236, 6456.17)	229371.4	72906.44	232174.4	65762.26	169722.4	42753.28	198314.6	58561.44	-0.91	0.002489	0.006067	0.42	0.229475	0.535521
COMPOUND_479_3036a10_383264	Rp	+	479.3036	10.38	LPE 18:1	2	10eV: 480.3121(100), 339.2917(65), 62.0591(25)	(959.6104, 4605.99)(960.6107, 2669.92)(961.6099, 1155.15)(962.5856, 375.31)(997.5841, 642.5)(998.5828, 631.99)(999.5919, 868.34)(502.2913, 9804.21)(503.2957, 2731.68)(504.3042, 1018.16)(981.5848, 1081.06)(982.5861, 1019.03)(983.5879, 936.85)(480.31, 89422.38)(481.3128, 23490.74)(482.3174, 4469.56)(483.3197, 822.29)(484.3148, 118.03)(976.6096, 1224.93)(977.6038, 1031.78)(978.593, 827.77)	454948.2	149436.1	431106.7	136479.7	467509.8	134784.9	525150	154331	0.09	0.748635	0.81722	0.52	0.123661	0.417518
COMPOUND_479_3051a10_349595	Rp	+	479.3051	10.35				(959.6103, 4308.15)(981.6449, 1180.32)(480.3103, 101622.45)(481.3132, 26427.35)(482.3171, 5381.14)(483.324, 1036.93)(976.6096, 1224.93)(977.6038, 1031.78)(978.593, 827.77)	372966.3	234442.8	272321	193008.9	303306.1	231461.5	391290.2	261493.1	-0.28	0.33533	0.436459	0.88	0.014341	0.179521
COMPOUND_479_3386a1_0067562	Hilic	+	479.3386	1.01				(959.6832, 4673.47)(960.6851, 2560.38)(961.6855, 982.84)(962.6777, 330.59)(502.327, 8182.47)(503.3312, 2114.01)(504.343, 2531.51)(505.3478, 678.73)(981.6645, 752.08)(982.6689, 441.0)(983.6677, 224.96)(497.3702, 190.65)(480.3461, 241358.69)(481.349, 63617.67)(482.3521, 10866.65)(483.3552, 1534.82)(484.3516, 211.97)	971891.6	232271.1	1011474	273130.2	296402.8	188265.6	274901.8	173156.8	-1.58	3.53E-12	1.09E-10	-0.04	0.855222	0.938351
COMPOUND_479_3394a10_438019	Rp	+	479.3394	10.44	duplicate of LPE18:1			(959.6779, 3830.33)(960.677, 2474.78)(961.6869, 777.59)(962.679, 310.37)(997.6679, 440.63)(998.6524, 618.82)(999.6308, 455.47)(502.3283, 26972.79)(503.3327, 7834.85)(981.6632, 1041.41)(982.6606, 571.28)(983.6541, 600.95)(480.3453, 59004.98)(481.3486, 15855.63)(482.3601, 20728.22)(483.3638, 5761.19)(484.3641, 1623.63)	1297217	331607.6	1272403	381582.2	309047.6	251653.8	295313.5	215454.5	-1.69	1.69E-12	6.46E-11	0.17	0.459969	0.729472
COMPOUND_479_3404a10_222365	Rp	+	479.3404	10.22				(480.3482, 8976.34)(481.3489, 1467.6)(482.3326, 2834.86)(483.3331, 1209.17)(484.341, 1099.83)	50435.05	13151.67	45447.53	9959.168	51280.33	13223.06	58513.06	20882.85	-0.15	0.58411	0.679802	0.32	0.333879	0.638282
COMPOUND_481_1587a10_686686	Rp	-	481.1587	10.69				(997.301, 221.57)(998.2834, 221.4)(999.2807, 360.26)(594.1527, 3394.28)(595.154, 1647.22)(516.1254, 10222.84)(517.134, 3235.77)(518.1224, 9768.49)(519.1332, 3315.88)(520.1229, 3502.74)(521.1319, 942.7)(522.1253, 479.85)(480.1499, 1584.12)(481.1502, 485.6)(482.1483, 944.9)(483.1576, 439.93)(484.1352, 878.1)(485.1392, 337.34)(486.1393, 661.35)(961.2934, 424.84)(962.2943, 256.84)(963.2897, 292.16)(526.149, 985.56)(527.1513, 819.56)(1075.3397, 74.34)(1076.3479, 28.63)(1077.3472, 64.85)	40019.07	31963.76	38487.11	18286.49	76521.5	31118.88	163785	83591.45	0.63	0.000574	0.001576	0.71	0.001167	0.055917
COMPOUND_481_3157a10_56611	Rp	-	481.3157	10.57				(997.5951, 477.15)(998.6191, 205.74)(480.3089, 85109.38)(481.312, 22155.23)(482.3147, 4124.21)(483.3135, 617.04)(484.3255, 52.78)(961.6231, 746.39)(962.627, 542.99)(963.6056, 839.76)(964.5808, 415.23)	123792.6	149229.2	150974.3	176511.4	83116.5	77979.4	121184.6	158304.1	-0.87	0.00253	0.006154	-0.03	0.928641	0.967445
COMPOUND_481_3159a10_257178	Rp	-	481.3159	10.26				(480.3087, 48573.28)(481.3119, 12398.32)(482.3146, 2239.86)(483.3164, 397.91)(484.3169, 54.22)(526.2941, 4181.18)(527.2973, 1405.5)(961.6361, 789.03)(962.6354, 442.81)	375190.5	37004.21	396915.6	36124.26	311730.6	36336.33	305552.5	39563.42	-1.16	5.23E-07	2.8E-06	-0.50	0.046246	0.273966
COMPOUND_481_316a10_146292	Rp	-	481.3160	10.15				(480.3088, 52579.06)(481.3121, 13021.43)(482.3145, 2354.45)(483.3178, 211.08)(961.6204, 359.23)(962.6047, 146.06)(526.2883, 7492.55)(527.2914, 1102.65)	118233.4	37584.58	120394	16407.09	76741.36	13987.69	77926.7	16007.31	-1.45	9.94E-11	1.49E-09	-0.21	0.329304	0.635357
COMPOUND_481_3162a10_6453085	Rp	-	481.3162	10.65	LPE 18:0	2	10 eV: 480.3079(100), 283.2637(30), 78.9587(1)	(997.6243, 587.84)(998.6519, 299.65)(480.3092, 160373.53)(481.3123, 43772.92)(482.3148, 7177.47)(483.3141, 1462.64)(484.3236, 216.48)(961.5826, 2377.71)(962.5406, 2706.24)(963.5712, 718.92)(964.5239, 1242.18)	478892.6	137549.8	524483.7	144391.8	291586.9	111366.3	302211.7	99430.7	-1.29	3.95E-07	2.22E-06	-0.15	0.571851	0.789098
COMPOUND_481_317a1_6459244	Hilic	+	481.3170	1.65				(963.6428, 423.39)(964.6448, 344.26)(965.6592, 50.6)(504.3066, 10343.25)(505.31, 2629.78)(506.315, 857.05)(507.3233, 122.91)(985.6221, 674.87)(986.6241, 327.34)(987.6175, 54.24)(499.353, 362.89)(482.3245, 18985.23)(483.328, 5252.08)(484.33, 1114.38)(485.3237, 125.03)(980.6668, 224.15)	145835.2	34382.72	157775.2	37956.54	87309.57	31978.38	93924.67	32543.2	-1.29	5.07E-07	2.73E-06	-0.13	0.645749	0.836838
COMPOUND_481_3174a1_2258265	Hilic	+	481.3174	1.23				(963.6423, 10542.88)(504.324, 18686.58)(505.3283, 5216.87)(506.317, 495.7)(482.325, 210759.84)(483.328, 54887.24)(484.3307, 10086.01)(485.3334, 1468.11)(486.3348, 216.27)(980.6902, 1300.52)	671625.7	208343.9	772496.2	252244.8	354509	120466	293807.7	123930.9	-1.13	7.35E-08	4.8E-07	-0.57	0.009473	0.153413
COMPOUND_481_3176a1_0978168	Hilic	+	481.3176	1.10				(504.3065, 995.88)(505.3096, 275.0)(506.3225, 203.17)(482.3248, 31799.39)(483.3282, 9038.21)(484.3308, 1676.26)(485.3315, 175.02)	112919.4	27592.44	123872.3	32298.34	74768.29	20793.54	64411.07	25273.63	-1.04	7.15E-06	2.94E-05	-0.54	0.033005	0.244873
COMPOUND_481_3181a10_028497	Rp	+	481.3181	10.03	LPC 15:0	2	10eV: 482.3257(100), 184.0733(24), 104.106(20), 258.1098(4)	(963.6451, 1177.14)(964.6449, 733.39)(965.6489, 177.9)(504.308, 28913.4)(505.3104, 7482.71)(506.3197, 1920.95)(520.2781, 700.2)(482.3255, 403392.38)(483.3289, 97274.88)(484.3325, 18369.54)(485.337, 2951.58)	877932.8	255472.7	936323.3	320972.4	449817.5	149318	387912.5	157236.1	-1.28	1.12E-09	1.22E-08	-0.36	0.088516	0.352184

									1st trimester				3rd trimester									
									Control		Pre-eclampsia		Control		Pre-eclampsia		Time			Interaction		
Compound	Column	Ion_mode	Mass	Retention_	Identification	ID_level	MSMS_spectra	CompositeSpectrum	Mean	SD	Mean	SD	Mean	SD	Mean	SD	Value	raw p	FDR_p	Value	raw p	FDR_p
COMPOUND_481_3182a10_647905	Rp	+	481.3182	10.65	LPE 18:0	2	20 eV: 341.3091(100), 44.0483(85), 216.0687(15), 421.2673(10)	(963.6374, 2573.37)(964.6361, 1452.37)(965.6124, 1262.27)(1001.6049, 840.45)(1002.6029, 667.95)(504.3068, 8321.37)(505.3109, 2416.95)(506.3109, 406.35)(985.6021, 1407.76)(520.2798, 636.44)(521.2786, 376.76)(482.3256, 150379.14)(483.3287, 38870.39)(484.3314, 7393.67)(485.3401, 1179.78)(980.6254, 905.38)(981.607, 896.16)	344673.5	70155.34	347075.2	91240.45	306478.4	125990	322058	91557.32	-0.67	0.025025	0.047922	0.34	0.329477	0.635357
COMPOUND_481_319a9_913841	Rp	+	481.3190	9.91				(504.3065, 10937.63)(505.3103, 2912.06)(506.3133, 1205.83)(499.371, 10173.13)(482.3252, 111964.23)(483.328, 29664.07)(484.3405, 6598.98)(485.3403, 1186.74)	151077	70882.81	159468.8	66858.19	128768.4	137476.9	183750.1	182845.2	-0.68	0.01634	0.033032	-0.08	0.812556	0.920924
COMPOUND_481_352a1_2328423	Hilic	+	481.3520	1.23				(963.6636, 2709.18)(964.6456, 994.3)(504.3424, 15009.95)(505.3453, 4199.13)(506.3581, 3542.67)(985.6514, 670.91)(986.6418, 543.18)(482.3608, 198765.64)(483.3641, 51656.55)(484.3667, 9010.0)(485.3625, 1668.73)(486.37, 222.56)	1093456	329327.3	1183969	386516.3	318020.1	255319.7	271731.5	199963.2	-1.39	4.03E-10	5.01E-09	-0.29	0.192785	0.505089
COMPOUND_481_3563a10_50593	Rp	+	481.3563	10.51	LPC O-16:0	2	20 eV: 104.1068(100), 482.3619(37), 184.0714(14)	(963.7152, 1884.69)(964.7158, 1353.46)(965.7191, 552.68)(966.7224, 209.03)(1001.7434, 1102.85)(1002.7495, 539.55)(1003.7187, 407.76)(504.3465, 25814.78)(505.3474, 7216.89)(506.3586, 41249.01)(985.6879, 406.13)(986.7012, 226.32)(987.6897, 1520.01)(499.3774, 100.18)(500.3765, 273.28)(482.3624, 266383.88)(483.3653, 65722.41)(484.3671, 11903.25)(485.3664, 2062.82)(486.3639, 306.32)(980.7479, 1005.1)	1614599	540797.6	1565750	519847.4	427676.9	435703.1	333292.8	293355	-1.42	4.23E-10	5.23E-09	-0.12	0.593797	0.804108
COMPOUND_483_1723a10_929026	Rp	-	483.1723	10.93				(1001.3236, 202.35)(1002.3026, 417.15)(518.1401, 2704.64)(519.1404, 837.69)(520.1384, 1937.33)(521.141, 745.35)(522.1369, 435.09)(1011.3098, 326.54)(1012.3183, 188.55)(482.1656, 17829.33)(483.1682, 5523.36)(484.1631, 11731.25)(485.162, 3928.84)(486.1601, 2492.29)	86200.64	29682.98	73101.49	30747.93	95402	27457.19	95951.77	32820.99	0.13	0.620566	0.712655	-0.42	0.168	0.480454
COMPOUND_484_3529a1_4378754	Hilic	+	484.3529	1.44				(485.3592, 6696.9)(486.3615, 2377.64)(487.366, 602.24)(243.1837, 21755.38)(243.6853, 6031.15)(244.1872, 1487.24)(244.6876, 247.64)	54401.43	17598.97	57037.72	23028.2	53681.71	37969.71	48485.35	26882.13	-0.32	0.279917	0.379286	-0.14	0.687233	0.85411
COMPOUND_484_6756a9_969427	Rp	+	484.6756	9.97				(970.3852, 179.72)(971.3898, 136.71)(507.6791, 368.71)(523.6415, 176.52)(485.6837, 10728.71)(486.686, 1562.82)(487.669, 1609.61)(488.6587, 248.55)(489.6793, 713.75)	75539.94	28291.62	62492.12	24240.59	72878.62	12045.36	77526.36	22626.79	0.00	0.988716	0.995346	0.77	0.028273	0.229862
COMPOUND_487_2856a12_53011	Rp	-	487.2856	12.53				(522.2525, 324.43)(523.248, 129.18)(524.245, 43.16)(486.2781, 12400.5)(487.2807, 4614.47)(488.2817, 1284.47)(489.2802, 217.1)(532.257, 2166.61)(533.2435, 2042.23)	54189.09	10797.68	58587.73	10052.06	54628.29	13600.94	49404.18	9542.958	-0.03	0.904959	0.938291	-0.71	0.028907	0.231966
COMPOUND_488_2207a9_978855	Rp	+	488.2207	9.98				(511.2068, 636.67)(512.2117, 306.54)(513.2134, 148.02)(527.1664, 409.27)(506.2536, 1379.6)(507.2571, 450.15)(508.2666, 297.78)(489.228, 5650.92)(490.2316, 2131.67)(491.236, 550.54)	21895.61	9179.286	21727.12	7084.948	12700	#DIV/0!	13488.67	2438.501	-1.20	1.84E-08	1.43E-07	-0.11	0.616569	0.816971
COMPOUND_489_2507a11_579841	Rp	-	489.2507	11.58				(1013.4207, 510.69)(1014.4236, 300.08)(1015.4148, 423.57)(602.2208, 734.1)(603.2269, 477.25)(604.2227, 829.27)(605.2211, 367.53)(606.2236, 358.71)(488.2436, 7955.97)(489.2474, 2094.66)(490.2418, 1713.31)(491.244, 343.21)	36019.85	9781.677	33761.48	8007.475	34235	11195.24	32968.65	7508.961	-0.46	0.126768	0.19922	0.46	0.200493	0.512498
COMPOUND_489_4186a12_100817	Rp	+	489.4186	12.10				(490.4268, 8818.71)(491.4305, 3148.52)(492.4401, 2391.88)	23923.45	8478.968	26201.95	10755.45	24033.07	6561.224	22615.15	7294.035	0.22	0.403072	0.507105	-0.53	0.094451	0.36381
COMPOUND_491_1816a10_687108	Rp	-	491.1816	10.69	no msms			(604.1659, 1004.02)(1027.3918, 218.27)(1028.4075, 216.48)(490.1776, 23162.13)(491.179, 6634.74)(492.1742, 7924.66)(493.1756, 1876.77)(494.1545, 3569.18)(981.3221, 339.32)(982.341, 167.78)	57573.35	17046.67	47960.63	17003.45	47278.5	11688.09	72723.44	32611.35	-0.21	0.371407	0.473886	1.25	3.22E-05	0.007252
COMPOUND_491_4321a11_773435	Rp	+	491.4321	11.77				(983.9017, 250.71)(984.9164, 432.01)(1021.85, 471.12)(1005.8423, 282.75)(509.4432, 432.42)(492.4415, 80084.85)(493.4446, 25613.92)(494.4477, 5065.91)(495.4498, 801.06)	178439.2	91147.68	139518.8	91547.61	96477.5	71491.47	82557.66	48293.78	-1.16	8.54E-06	3.45E-05	0.19	0.506247	0.755512
COMPOUND_493_2006a10_866357	Rp	-	493.2006	10.87				(492.1932, 6461.68)(493.1967, 1688.56)(494.1913, 2142.8)(495.1927, 807.63)(496.1779, 2887.23)(497.1791, 1086.43)(498.1782, 3501.74)(985.3685, 258.35)(986.3685, 155.55)(987.3919, 178.31)	36335.41	13797.09	40416.38	16251.12	24148	10093.33	27043.2	13399.52	-1.13	8.22E-07	4.19E-06	0.01	0.955938	0.980661
COMPOUND_493_2262a11_366556	Rp	+	493.2262	11.37				(987.4649, 1675.61)(988.4622, 1549.11)(516.1869, 441.17)(494.2282, 7225.71)(495.232, 2338.21)(496.2362, 542.09)	38422.62	21427.6	27248.73	9893.487	27544.6	14686.29	21209.93	12106.81	0.31	0.190755	0.279675	0.09	0.741566	0.888965
COMPOUND_493_3177a1_0837613	Hilic	+	493.3177	1.08				(987.6443, 989.34)(516.3071, 2452.68)(517.3101, 656.1)(1009.6216, 262.04)(494.3247, 85389.75)(495.3278, 23202.18)(1004.6885, 1555.2)	179276.6	62596.18	214341	79140.2	178250.4	41123.39	162389.1	76502.16	-0.09	0.729018	0.803204	-0.71	0.022851	0.214418
COMPOUND_493_3188a9_7975	Rp	+	493.3188	9.80				(516.3059, 5845.44)(517.3096, 1552.59)(532.2904, 1149.59)(533.2891, 554.21)(511.3627, 1944.0)(494.3252, 129277.25)(495.3284, 35360.16)(496.3332, 8270.9)(497.3387, 1447.25)	208954.4	89367.13	229561.2	99721.74	169685.8	45986.49	166747.3	79015.3	-0.51	0.03936	0.071195	-0.35	0.21117	0.519294
COMPOUND_493_3191a1_2026542	Hilic	+	493.3191	1.20				(987.6403, 4501.15)(988.6442, 2493.7)(516.3066, 13189.24)(517.3102, 3915.49)(1009.6245, 1032.99)(1010.6302, 744.82)(1011.6391, 6285.4)(494.3241, 531382.4)(495.3281, 132933.17)(1004.7115, 14470.64)	1147603	419477.1	1401814	595115.5	1018645	243328.1	946115.4	460477.2	-0.35	0.163059	0.245296	-0.66	0.025392	0.219937
COMPOUND_493_3198a9_925706	Rp	+	493.3198	9.93	LPC 16:1	2	10eV: 493.3266(100), 104.1047(30), 184.0732(20), 258.1161(3)	(987.6423, 3422.64)(988.6458, 1682.65)(989.6516, 568.19)(1025.6024, 196.66)(516.308, 53597.34)(517.3119, 14621.11)(1009.6221, 679.99)(1010.6223, 334.76)(1011.6122, 631.5)(1012.6101, 475.34)(532.281, 2303.41)(533.2854, 1157.19)(534.2915, 2158.31)(511.3424, 400.1)(512.3363, 7622.08)(513.3423, 2505.93)(494.3261, 618151.7)(495.3302, 157898.69)(496.3329, 30218.62)(497.3409, 5675.5)	1472723	655365.6	1764497	961093.3	1268945	365159.3	1243440	656917.4	-0.38	0.129394	0.20264	-0.39	0.18069	0.493854
COMPOUND_493_354a10_570445	Rp	+	493.3540	10.57				(987.7371, 1279.74)(988.7297, 570.83)(516.3429, 1388.67)(517.3479, 604.14)(518.3233, 5929.88)(494.3616, 29607.63)(495.3646, 8149.66)	123999.7	50477.26	119864.6	63626.99	38279.43	22702.25	49606.47	31171.53	-1.19	2.06E-06	9.52E-06	0.10	0.723129	0.878838
COMPOUND_494_0199a10_220755	Rp	+	494.0199	10.22				(1026.9968, 142.33)(1028.0077, 120.43)(517.0235, 222.82)(518.0278, 451.22)(532.9828, 551.2)(495.027, 10310.16)(496.0301, 1477.55)(497.0117, 1823.56)(498.0143, 241.92)(498.9933, 178.1)(1006.1233, 292.38)	48140.6	15406.18	41313.4	15249.26	65364.38	17514.53	65827.2	21029.1	0.59	0.029807	0.055984	0.52	0.106608	0.38909
COMPOUND_494_2867a8_815159	Rp	-	494.2867	8.82				(607.2659, 128.28)(608.2667, 74.84)(609.2662, 68.7)(493.2794, 1606.97)(494.2809, 541.58)(495.2947, 543.57)(496.2978, 319.67)(987.5641, 587.88)(988.5528, 402.98)(989.5835, 3240.95)	18634.33	11491.74	19419.86	10499.41	126976.9	70375.88	82839.77	55243.59	1.87	9.83E-14	9.22E-12	-0.53	0.02425	0.219303
COMPOUND_494_2871a8_755209	Rp	-	494.2871	8.76				(607.2617, 1004.28)(608.265, 410.26)(609.2567, 748.79)(493.2801, 35029.75)(494.2832, 11238.14)(495.2948, 13979.26)(496.2973, 4172.33)(497.3007, 699.43)(987.5661, 1109.76)(988.5727, 714.52)(989.5804, 217.24)	23377.7	12190.9	30000.57	18326.26	305090.6	154376.4	209633.5	125852.9	1.95	1.49E-16	8.4E-14	-0.56	0.007145	0.131884
COMPOUND_494_2873a9_267705	Rp	-	494.2873	9.27				(493.28, 92364.91)(494.2832, 27799.48)(495.2862, 5791.66)(496.2888, 1062.07)	18924.96	7185.801	20667	16769.82	209183	155191	185511.7	125362.5	1.48	9.3E-11	1.42E-09	-0.49	0.02903	0.231966
COMPOUND_494_4354a12_537136	Rp	+	494.4354	12.54				(495.4419, 13706.83)(496.4451, 4996.42)(497.446, 1285.14)(498.4485, 168.81)	25880.94	21520.02	23457.39	15332.59	16135.09	4594.902	27824.36	18739.15	-0.12	0.672235	0.759214	0.36	0.265357	0.575669
COMPOUND_494_482a11_230958	Rp	+	494.4820	11.23				(517.4862, 1042.3)(1011.9438, 1651.08)(512.5209, 182.15)(495.489, 12011.82)(496.4922, 4378.04)(497.4956, 771.75)	29548.37	10520.9	34055.98	11513.75	43767	20025.34	42008.63	17159.55	0.91	0.001934	0.004818	-0.46	0.175858	0.490747
COMPOUND_495_3328a1_1757683	Hilic	+	495.3328	1.18				(991.6724, 30386.41)(992.6769, 17098.4)(993.6783, 4869.37)(994.6778, 976.57)(995.6787, 265.02)(518.3225, 84636.95)(519.3254, 22284.29)(1013.6535, 7839.34)(1014.6561, 3796.04)(1015.6725, 34145.09)(1016.6748, 20673.4)(1017.6873, 40142.02)(1018.6914, 20528.82)(1019.6998, 14155.05)(513.3634, 2376.5)(496.3399, 1479497.2)(497.3443, 365744.22)(498.3464, 59618.42)(499.3487, 9185.12)(500.3519, 933.31)(1008.6801, 434.83)	70418868	6523333	72443478	7610888	48635714	9223349	50331579	9227427	-1.53	1.27E-11	3.05E-10	-0.09	0.683166	0.853695

									1st trimester				3rd trimester									
									Control		Pre-eclampsia		Control		Pre-eclampsia		Time			Interaction		
Compound	Column	Ion_mode	Mass	Retention_	Identification	ID_level	MSMS_spectra	CompositeSpectrum	Mean	SD	Mean	SD	Mean	SD	Mean	SD	Value	raw p	FDR_p	Value	raw p	FDR_p
COMPOUND_495_3328a1_0589571	Hilic	+	495.3328	1.06				(991.672, 29057.14)(992.6751, 16058.74)(993.6786, 4766.42)(994.6813, 1477.52)(995.6883, 185.61)(518.3227, 63840.67)(519.326, 16340.98)(1013.6534, 3707.72)(1014.654, 1793.31)(1015.6722, 16688.67)(1016.6749, 9354.33)(1017.6875, 41425.62)(1018.6912, 21759.93)(1019.7042, 98936.38)(513.3641, 834.02)(496.3393, 1219908.1)(497.3437, 298859.9)(498.3462, 55659.13)(499.3479, 7692.34)(500.3546, 949.42)(1008.687, 484.12)(1009.6964, 843.41)	14404974	2296360	15290129	2600131	7844737	2233051	8038482	2242212	-1.46	3.19E-11	6.39E-10	-0.22	0.294592	0.604759
COMPOUND_495_3338a10_146823	Rp	+	495.3338	10.15				(991.6732, 36653.44)(992.6765, 18924.78)(993.6795, 5594.03)(994.6797, 1601.03)(995.6792, 428.61)(1029.6393, 1833.15)(518.323, 28452.49)(519.3267, 7517.75)(520.3384, 10211.66)(521.3423, 3219.92)(1013.6577, 3416.5)(1014.655, 2342.23)(1015.6676, 6465.6)(1016.6753, 2936.52)(1017.6731, 1042.37)(1018.6546, 683.1)(534.2974, 6156.16)(513.3746, 7074.21)(496.3409, 381713.9)(497.3446, 96118.66)(498.3471, 17530.64)(499.3555, 3325.97)(1008.7201, 508.24)(1009.7108, 222.72)	17896226	12723343	16075937	9149152	8148847	2474081	10091920	7493636	-1.59	3.38E-10	4.37E-09	0.33	0.191881	0.505089
COMPOUND_495_3344a10_254457	Rp	+	495.3344	10.25	LPC 16:0	2	10eV: 184.0742(100), 496.3416(90), 86.0972(2), 258.1104(1)	(991.6738, 2459.24)(992.6758, 1509.5)(993.6778, 523.5)(994.6754, 42.68)(1029.6075, 109.48)(1030.6252, 95.53)(1031.5989, 253.28)(1032.5836, 191.89)(1033.5847, 157.08)(1034.586, 88.66)(1035.59, 83.13)(518.3239, 29849.04)(519.3269, 8185.76)(520.3396, 14745.57)(521.3449, 4344.97)(1013.6548, 3614.31)(1014.6574, 1704.45)(1015.6329, 3878.15)(1016.643, 1116.06)(534.2968, 1339.1)(513.385, 6356.34)(496.3413, 1492052.9)(497.3461, 375985.0)(498.3486, 65725.84)(499.3507, 8944.04)(1008.7401, 1060.76)(1009.7502, 651.94)	61771698	7075727	60867391	8063637	42671429	8275304	44522807	8432061	-1.58	7.53E-11	1.2E-09	0.21	0.367493	0.669574
COMPOUND_496_2532a5_9693127	Rp	+	496.2532	5.97				(519.2421, 9117.8)(520.2453, 2640.6)(521.2457, 608.51)(522.2419, 97.81)(1015.496, 434.82)(1016.5096, 60.41)(535.2185, 591.48)(536.2237, 245.5)(514.287, 142497.67)(515.2901, 35369.74)(516.2924, 7180.76)(517.2955, 1229.07)(518.3014, 164.35)(497.2603, 13872.17)(498.2629, 4154.7)(499.2658, 799.01)(500.266, 157.25)	284305.3	140646.5	265092.7	150060.3	339459.8	169477.6	285588.6	131160.2	0.36	0.231363	0.326665	-0.07	0.850752	0.936867
COMPOUND_496_3027a8_848101	Rp	-	496.3027	8.85				(609.2806, 2868.97)(610.2816, 1205.85)(531.279, 1703.71)(532.2608, 369.24)(495.2959, 341107.12)(496.2991, 94615.74)(497.3017, 19011.66)(498.3044, 2959.37)(499.3107, 398.12)(991.5994, 9084.15)(992.6027, 5220.3)(993.6065, 1988.97)(994.6086, 517.94)(995.6015, 120.48)	282984.8	98287.45	314625.3	142708.2	1271915	576139.1	1184983	543002.2	1.52	7.37E-12	1.98E-10	-0.04	0.832555	0.926681
COMPOUND_496_3299a12_532405	Rp	-	496.3299	12.53				(531.3, 196.39)(1037.6929, 406.11)(1038.6976, 224.7)(1039.6699, 323.73)(495.3227, 131393.75)(496.3261, 41028.5)(497.3289, 6149.37)(498.3326, 821.74)(499.3271, 117.35)(991.6799, 543.48)(992.6775, 484.88)(541.319, 693.22)(542.3123, 246.15)(543.3242, 37.8)	546963.2	92444.58	581286.2	117767.8	641202.1	119020.3	650651.4	111792.1	0.73	0.00895	0.019159	-0.14	0.657246	0.842058
COMPOUND_497_2501a1_7669948	Hilic	+	497.2501	1.77				(995.5211, 280.67)(520.2431, 508.2)(521.2478, 254.95)(515.2824, 510.34)(498.2568, 7515.74)(499.2604, 1686.27)(500.273, 1172.35)	43939.91	8613.9	50747.96	13787.32	33577.57	13704.3	35977.23	14534.74	-0.82	0.003736	0.008697	-0.19	0.550428	0.7796
COMPOUND_498_6922a10_344574	Rp	+	498.6922	10.34				(998.4319, 813.37)(999.4298, 978.15)(521.6986, 399.03)(1020.4002, 448.02)(1021.4085, 518.31)(516.7411, 194.34)(499.6989, 10528.04)(500.7016, 1496.27)(501.6847, 1356.92)(502.6844, 261.55)	59580.29	24919.13	44857.15	17974.15	53214.31	10283.61	58143.7	17291.68	0.18	0.482411	0.58406	0.76	0.018977	0.196411
COMPOUND_499_0257a10_346504	Rp	+	499.0257	10.35				(999.0896, 390.48)(1000.0769, 530.05)(522.0084, 488.71)(1021.0486, 444.69)(537.9787, 424.22)(500.0332, 8615.83)(501.0276, 508.61)(502.0182, 576.54)(1016.0691, 400.17)	44024.12	16154.15	36096.61	12368.61	44052.85	10281.36	47397.38	14878.45	-0.04	0.890431	0.926471	0.91	0.008503	0.14883
COMPOUND_499_2338a11_58046	Rp	-	499.2338	11.58				(612.2125, 261.18)(498.2265, 5609.38)(499.2288, 1764.08)(500.2321, 667.82)(501.2274, 133.68)(997.4743, 248.98)(998.4908, 333.26)(999.4995, 58.36)(1111.4974, 613.12)(1112.4825, 901.16)(1113.4849, 624.68)(1114.4836, 705.02)	24442.58	3639.254	25381.56	4984.753	23042.2	2788.806	21483.82	5362.455	-0.79	0.000788	0.002098	-0.12	0.662619	0.844492
COMPOUND_499_2692a9_864109	Rp	-	499.2692	9.86	LPE 20:5	2	20 eV: 301.217(100), 498.2841(42), 196.0385(18), 140.0103(4)	(498.2618, 60075.57)(499.265, 17496.03)(500.2683, 3117.58)(501.2709, 621.68)(997.5291, 694.82)(998.5345, 412.44)(999.5411, 283.0)	74139.06	54931.8	67983.27	49940.08	42754.9	17066.23	43161.44	24756.38	-0.79	0.002279	0.005586	0.49	0.095271	0.36381
COMPOUND_499_2706a9_868546	Rp	+	499.2706	9.87				(999.5341, 513.96)(1000.5509, 265.32)(1001.5308, 165.58)(522.2597, 2976.54)(523.2646, 1037.41)(524.2736, 528.06)(538.2284, 1164.74)(539.2304, 568.96)(540.2213, 191.28)(517.3111, 6716.06)(500.2785, 17632.49)(501.2809, 5174.59)(502.2864, 1480.74)(503.2719, 166.01)	76453.06	57244.65	71514.09	48397.25	50440.1	22233.09	46041.71	27139.6	-0.72	0.008646	0.018615	0.09	0.783784	0.909997
COMPOUND_499_2958a9_522447	Rp	-	499.2958	9.52	Taurocholic acid	1	40 eV: 498.2895(100), 106.981(3), 384.5558(2)	(1033.5697, 111.29)(1034.5839, 50.69)(1035.5591, 170.26)(612.2688, 2186.46)(613.2725, 1012.72)(498.2894, 248445.56)(499.2924, 73630.37)(500.2914, 21566.52)(501.2922, 4273.41)(502.2946, 649.06)(997.5858, 22488.22)(998.588, 14260.76)(999.5887, 6354.07)(1000.59, 2466.07)(1001.5907, 609.58)	248089.9	291225.4	246726.7	194094	1145505	2687784	1116845	1640462	0.76	0.003427	0.008038	0.14	0.637335	0.831083
COMPOUND_499_2959a9_756559	Rp	-	499.2959	9.76	duplication of taurocholic acid			(1033.5665, 347.14)(1034.58, 261.52)(1035.5494, 314.77)(612.2681, 966.33)(613.2734, 448.41)(498.2889, 42177.32)(499.2923, 13001.38)(500.2918, 3938.82)(501.293, 822.83)(502.2876, 36.0)(997.5854, 9582.22)(998.5888, 5536.31)(999.5876, 2535.57)(1000.5898, 965.17)	154303.8	190654	182384.7	120650.8	296305.3	267454.9	652998.4	751917	0.60	0.017312	0.034685	0.29	0.322157	0.628989
COMPOUND_499_6875a9_962222	Rp	+	499.6875	9.96				(1038.289, 167.99)(522.6738, 344.59)(523.6993, 418.83)(538.6349, 146.56)(500.6927, 13289.25)(501.6952, 679.13)(502.6786, 936.06)	52833.37	19725.82	45824.14	18344.97	54259.08	9672.222	56493.7	15774.07	0.11	0.704173	0.783952	0.62	0.07725	0.333235
COMPOUND_499_9378a9_278484	Rp	-	499.9378	9.28				(612.9463, 179.79)(498.9301, 15360.33)(499.933, 1425.14)(500.9293, 840.5)(501.9281, 57.82)(544.9557, 145.45)	37258.15	19845.14	43597.67	25397.85	27271	11419.23	32824.19	17169.54	-0.40	0.003257	0.007689	-0.19	0.210875	0.519294
COMPOUND_500_0951a8_790591	Rp	-	500.0951	8.79				(535.0686, 569.1)(536.0647, 171.93)(537.0628, 283.21)(499.0874, 8065.55)(500.0912, 1650.07)(501.085, 2826.85)(502.09, 683.94)(503.0974, 100.05)(545.0958, 358.65)(546.1072, 219.09)	29240.3	2983.167	30154.59	3323.837	24785	2265.338	27187.33	3779.474	-1.24	1.84E-05	6.95E-05	0.42	0.189037	0.504029
COMPOUND_500_149a9_034518	Rp	-	500.1490	9.03				(613.1325, 1882.57)(614.1307, 739.78)(615.1356, 2062.09)(616.1341, 994.71)(617.1341, 1382.18)(535.1175, 1876.0)(536.1262, 534.62)(537.1157, 1809.04)(538.1234, 437.7)(539.1316, 1575.3)(499.1456, 7273.55)(500.1495, 2709.61)(501.1508, 1269.81)(502.1418, 128.89)(545.1447, 7973.79)(546.1496, 2603.98)(547.1475, 5340.26)(548.1569, 1505.54)(549.1462, 2231.16)(550.1474, 859.44)(551.1378, 2903.38)	63378.67	15919.79	82379.13	26339.14	212676.1	138134.1	172076.7	87390.94	1.32	3.22E-08	2.31E-07	-0.33	0.181994	0.495594
COMPOUND_500_3898a12_96994	Rp	+	500.3898	12.97				(523.3808, 1723.08)(524.38, 1037.08)(525.3809, 286.55)(539.3547, 337.02)(540.3512, 140.03)(518.4245, 4840.45)(519.4279, 1705.88)(520.43, 588.81)(521.4352, 89.22)(501.3987, 6214.47)(502.4018, 1978.75)(503.3998, 842.79)(504.4067, 166.24)	57102.51	18996.77	49184.89	17587.77	54432.14	12777.54	60514.47	18767.01	-0.10	0.738212	0.809768	0.73	0.041676	0.265944
COMPOUND_501_2844a10_033219	Rp	-	501.2844	10.03				(614.2624, 2271.91)(615.263, 1076.91)(500.2777, 112531.19)(501.2806, 31047.99)(502.2835, 5936.7)(503.2839, 926.51)(1001.5623, 1939.45)(1002.5658, 1185.69)(1003.5708, 400.32)	174945.8	171084.3	95927.57	95489.64	110139.9	105309.5	102819	97827.73	-0.87	0.003312	0.007802	0.63	0.068426	0.319037
COMPOUND_501_2849a10_113906	Rp	-	501.2849	10.11	LPE 20:4	2	20 eV: 303.2318(100), 500.2763(35), 196.0371(8), 59.0131(5), 418.2086(2)	(500.2777, 114650.91)(501.2806, 31438.31)(502.2835, 5643.52)(503.2839, 1096.98)(1001.5623, 5324.79)(1002.5656, 2961.08)(1003.568, 1046.11)(1004.5776, 333.37)	340047	103750.2	326194.5	104381	211330.4	64378.39	263017.4	94604.12	-1.37	5.01E-06	2.13E-05	0.73	0.028786	0.231966
COMPOUND_501_2849a1_619784	Hilic	+	501.2849	1.62				(1003.5741, 824.59)(1004.5779, 404.54)(1005.5888, 824.5)(1006.5953, 368.29)(1007.6019, 510.28)(524.2747, 11491.11)(525.2776, 3303.45)(1025.5555, 726.89)(1026.559, 455.06)(1027.5735, 907.58)(1028.5782, 586.25)(1029.5897, 524.53)(1030.5938, 283.58)(502.2926, 19177.85)(503.2961, 5619.49)(504.3067, 9152.55)(505.311, 2511.58)(506.3138, 672.01)(507.3268, 84.63)(1020.5796, 66.86)(1021.5412, 261.52)	328899.7	84616.16	344159.6	91906.24	221777.9	66923.48	272526	89265.18	-1.22	3.89E-05	0.000133	0.45	0.176915	0.491826

Compound	Column	Ion_mode	Mass	Retention_	Identification	ID_level	MSMS_spectra	CompositeSpectrum	1st trimester				3rd trimester									
									Control		Pre-eclampsia		Control		Pre-eclampsia		Time			Interaction		
									Mean	SD	Mean	SD	Mean	SD	Mean	SD	Value	raw p	FDR_p	Value	raw p	FDR_p
COMPOUND_501_2875a10_02062	Rp	+	501.2875	10.02				(1003.5721, 262.94)(1004.5721, 194.41)(1005.5563, 212.47)(524.2878, 1586.24)(525.2813, 1799.44)(540.2887, 1182.81)(541.2924, 616.85)(542.2699, 189.23)(502.2949, 26319.2)(503.2976, 7553.41)(504.3072, 15447.37)(505.3116, 3957.92)(506.3174, 788.88)	111159.6	28390.13	112560.6	32063.16	59996.86	15660.22	68552	21812.78	-1.06	5.51E-05	0.000181	-0.04	0.898253	0.954633
COMPOUND_501_2896a10_118062	Rp	+	501.2896	10.12				(1003.5798, 5106.05)(1004.5827, 2432.79)(1005.5949, 1235.58)(1006.6172, 475.15)(524.3006, 14008.0)(525.2996, 4980.8)(1025.5571, 592.09)(540.245, 788.49)(541.2571, 411.69)(502.2957, 131275.44)(503.2992, 34906.9)(504.3021, 6928.46)(505.3013, 1187.77)(1020.6341, 4889.14)	307328.9	66672.27	273361.9	70013.67	228535.2	59571.31	285074.1	95376.03	-1.07	0.000509	0.001416	1.14	0.001656	0.06002
COMPOUND_503_3a10_28832	Rp	-	503.3000	10.29				(538.3175, 632.68)(539.3032, 206.98)(502.293, 24411.81)(503.2968, 6579.35)(504.2975, 1339.55)(505.2966, 227.34)(548.2891, 305.26)(549.2886, 6183.97)(550.2908, 2258.69)	65500.58	26223.47	67788.15	32113.84	53549.21	17482.92	74470.81	31463.98	-0.42	0.165725	0.248975	0.65	0.071992	0.325554
COMPOUND_503_3005a1_2347902	Hilic	+	503.3005	1.23				(504.3069, 11265.45)(505.3097, 2808.13)(506.3088, 233.1)	26323.18	10308.62	28647.43	10681.79	19006	5547.544	14786.25	5505.901	-0.61	0.024268	0.046711	-0.15	0.641503	0.834103
COMPOUND_503_3036a10_293396	Rp	+	503.3036	10.29				(542.2772, 1125.11)(543.2722, 445.11)(544.283, 430.44)(521.343, 6521.31)(504.3095, 29545.69)(505.3131, 7946.14)(506.3156, 1753.56)	69067.08	17108.93	64426.78	18748.09	60248.21	16006.8	74288.7	22327.69	-0.33	0.25758	0.354133	0.98	0.00625	0.124555
COMPOUND_504_0226a10_077409	Rp	+	504.0226	10.08				(1009.0809, 315.79)(527.0177, 536.85)(1031.0457, 249.85)(522.0412, 252.85)(543.0114, 175.75)(505.0309, 8213.95)(506.0342, 1157.61)(507.0161, 1302.53)(508.0156, 181.9)(509.0284, 699.63)	77976.96	30889.64	63657.3	25668.6	83101.85	13326.89	87999.98	25065.94	0.21	0.457062	0.562154	0.80	0.018307	0.196324
COMPOUND_505_1886a10_66283	Rp	-	505.1886	10.66				(504.1804, 10661.45)(505.1834, 3177.96)(506.189, 1026.57)	21411.27	6025.326	19461.61	5884.237	23602.5	9303.247	28391.3	8378.38	0.30	0.244851	0.339954	0.79	0.01174	0.167839
COMPOUND_505_3154a10_109535	Rp	-	505.3154	10.11				(504.3085, 19441.63)(505.3121, 5914.43)(506.3149, 1169.89)(507.3179, 241.59)(550.3176, 178.44)(551.3161, 49.29)(1123.6201, 2372.57)(1124.6262, 1069.74)(1125.6324, 425.29)	137360.2	34443.93	149031.1	28569.74	101014.4	21540.51	91574.82	24346.29	-1.00	2.64E-05	9.55E-05	-0.50	0.057793	0.298567
COMPOUND_505_3163a10_003307	Rp	-	505.3163	10.00				(504.309, 5444.23)(505.3125, 1707.39)(506.3207, 302.56)(507.3267, 62.01)(550.313, 682.44)(551.3197, 177.11)(552.322, 1644.48)(553.3215, 471.68)(1123.6403, 227.77)(1124.6447, 159.7)(1125.6342, 725.08)(1126.6365, 395.96)	31994.4	18318.29	32084.49	9326.166	33132.5	31742.48	25869.57	23445.32	-0.38	0.170694	0.25474	-0.52	0.120473	0.410445
COMPOUND_505_3515a0_9708553	Hilic	+	505.3515	0.97				(1011.7013, 907.03)(1012.7141, 952.18)(1013.721, 753.24)(528.3433, 2245.4)(529.3471, 817.4)(530.3589, 2396.09)(531.3652, 804.76)(1033.7181, 1118.61)(506.3613, 57620.86)(507.3647, 16958.99)(508.3763, 36477.94)(509.38, 10470.07)(510.36, 15474.87)	#DIV/0!	#DIV/0!	#DIV/0!	#DIV/0!	#DIV/0!	#DIV/0!	#DIV/0!	#DIV/0!	-1.11	7.96E-06	3.24E-05	0.00	0.998259	0.999577
COMPOUND_505_3527a10_534728	Rp	+	505.3527	10.53				(528.3502, 2754.64)(529.365, 1646.48)(530.3593, 13128.18)(531.364, 4319.93)(1033.7366, 1861.37)(1034.7391, 529.05)(506.3604, 6547.72)(507.3625, 2437.43)(508.3724, 2146.52)(509.3647, 80.83)	283022.3	83130.61	284796	87821.88	97603.7	93906.3	88344.72	77184.87	-1.38	1.47E-08	1.17E-07	0.04	0.881565	0.950612
COMPOUND_506_129a8_312418	Rp	-	506.1290	8.31				(619.1146, 1593.71)(620.12, 395.13)(621.11, 1414.12)(622.1091, 400.28)(623.1271, 285.84)(541.0988, 1576.48)(542.1094, 551.36)(543.101, 653.93)(544.1091, 321.28)(545.1083, 133.58)(505.1198, 1036.19)(506.1223, 392.81)(507.1074, 155.13)(551.1265, 3883.45)(552.1316, 779.55)(553.1456, 301.94)	27491.29	4826.948	27780.67	5171.314	27592.58	6287.922	27754.81	5289.959	-0.32	0.288027	0.387015	0.17	0.643009	0.835096
COMPOUND_507_3319a10_376232	Rp	-	507.3319	10.38				(1059.662, 922.3)(1060.6674, 572.93)(506.3244, 20397.93)(507.3276, 5787.86)(508.3295, 1104.59)(509.3338, 214.38)(552.3295, 3939.24)(553.3339, 1301.79)	176262.6	27398.13	184445.7	32250.72	108145.3	18574.69	112622.8	25386.28	-1.57	1.86E-12	6.88E-11	-0.02	0.908957	0.958319
COMPOUND_507_3319a10_366358	Rp	+	507.3319	10.37				(530.3198, 3455.17)(531.3201, 1280.04)(546.2868, 617.97)(525.3657, 19072.23)(508.3394, 9082.84)(509.3497, 3058.08)	176978.1	40741.5	168248.6	43066.06	90562.5	30287.11	103042.2	27934.72	-1.25	2.25E-07	1.3E-06	0.33	0.189045	0.504029
COMPOUND_507_3329a1_0469726	Hilic	+	507.3329	1.05				(1015.6742, 57667.95)(1016.6775, 29849.48)(1017.6861, 23661.02)(1018.6906, 9779.13)(530.3571, 949.14)(531.35, 420.93)(532.3383, 3039.83)(533.3411, 1035.92)(1037.6542, 7739.43)(1038.6577, 4797.17)(1039.6693, 12482.79)(1040.6733, 6816.96)(1041.6864, 14641.06)(508.3414, 4988.19)(509.3453, 1336.84)(510.3561, 59062.14)(1032.7078, 1382.89)	474864.2	126423	529554.9	115059.8	161434.6	59060.19	177255.6	106518.2	-1.44	4.85E-10	5.88E-09	-0.24	0.298884	0.60748
COMPOUND_507_3333a1_1561561	Hilic	+	507.3333	1.16				(1015.6723, 68317.91)(1016.6759, 35864.08)(1017.6864, 42490.6)(1018.6897, 20749.91)(530.3579, 1632.26)(531.3617, 698.75)(532.3389, 6958.24)(533.3411, 1871.13)(1037.6559, 24833.93)(1038.6597, 14267.77)(1039.6724, 93591.88)(1040.6761, 51922.94)(1041.6877, 122455.48)(508.3763, 36111.95)(509.3798, 10200.6)(510.3567, 65323.19)(1032.7087, 15489.18)	3132720	1060411	3422158	955536.3	1808703	488481	1755027	600216.3	-1.22	5.89E-07	3.11E-06	-0.28	0.281153	0.588984
COMPOUND_507_3342a10_136346	Rp	+	507.3342	10.14				(1015.6755, 181896.17)(530.3126, 2213.02)(531.3232, 1419.03)(532.3273, 5668.18)(546.2964, 1164.91)(525.3762, 3461.71)(508.3399, 27478.92)(509.3434, 7702.64)(510.3471, 1724.2)(511.3445, 306.18)	690763.4	210602.2	718481.5	234591.1	246942.9	104180.9	259028.7	126763.6	-1.55	2.43E-11	5.21E-10	-0.02	0.923184	0.965307
COMPOUND_507_3683a0_95514464	Hilic	+	507.3683	0.96				(1015.7845, 877.24)(530.3601, 1490.14)(531.3661, 628.39)(532.3748, 2058.0)(533.3802, 716.54)(508.3765, 40444.21)(509.3803, 11595.99)(510.3735, 3849.34)(511.3701, 801.96)	135592.5	41944.33	142484.5	49712.82	49245.92	34262.14	41850.18	25905.11	-0.79	0.002205	0.005427	-0.17	0.573388	0.790246
COMPOUND_507_3688a1_1631838	Hilic	+	507.3688	1.16				(530.3608, 688.49)(531.3616, 171.16)(532.3741, 632.72)(533.38, 221.84)(508.3767, 154340.42)(509.3799, 42090.49)	795767.1	176934.7	811685.8	191410.8	236793.3	54023.76	201317	32276.94	-0.94	0.000207	0.000613	0.13	0.645438	0.836838
COMPOUND_507_3695a10_783912	Rp	+	507.3695	10.78				(530.3649, 3729.5)(531.3719, 1964.88)(532.379, 2110.45)(508.3768, 44672.52)(509.3796, 13194.99)(510.3904, 16924.49)(511.3927, 5313.84)	177587.7	55083.81	170576.5	57795.8	97907.67	63616.02	80446.73	41686.3	-1.34	3.18E-10	4.19E-09	-0.03	0.888851	0.953702
COMPOUND_507_3714a10_592616	Rp	+	507.3714	10.59	LPC O-18:1	2	20eV: 104.1058(100), 508.3774(50), 184.0751(6)	(1015.7421, 3041.73)(1016.7484, 1831.57)(1017.7301, 1494.35)(530.3598, 27657.2)(531.3623, 8100.28)(532.3672, 2407.41)(533.3578, 512.8)(1037.704, 490.88)(1038.7261, 390.51)(508.3772, 536099.0)(509.381, 147956.77)(510.3816, 27576.99)(511.3802, 4392.93)	1590641	492224.1	1556485	502789.9	511513.4	506177.7	407599	344758.1	-1.42	4.99E-09	4.38E-08	-0.06	0.824297	0.923541
COMPOUND_508_6964a10_214169	Rp	+	508.6964	10.21				(1056.3112, 262.48)(531.6823, 275.01)(532.6888, 552.09)(547.6775, 420.54)(509.7021, 9152.34)(510.7061, 1465.79)(511.69, 1482.5)(512.6841, 251.38)(513.6896, 395.29)(514.6813, 43.69)(515.6821, 254.15)	62052.13	22718.25	52151.84	20138.55	71888.92	13653.23	75799.18	24100.33	0.36	0.194778	0.283887	0.66	0.049862	0.282135
COMPOUND_509_0289a10_21308	Rp	+	509.0289	10.21				(1019.0286, 202.79)(532.0112, 747.95)(533.0135, 357.44)(1041.036, 253.95)(527.0433, 351.6)(547.9696, 227.7)(510.0367, 16859.15)(511.0365, 946.9)(512.0209, 978.06)	46422.47	19766.99	36064.81	15403.39	51488.83	12844.81	56787.26	20150.63	0.04	0.88534	0.923801	0.89	0.009747	0.153413
COMPOUND_509_3476a10_651134	Rp	-	509.3476	10.65				(1053.663, 286.95)(1054.6544, 199.16)(1055.6805, 248.33)(1056.7012, 115.8)(1057.6534, 227.91)(622.3603, 810.69)(623.3713, 447.39)(544.3351, 1332.59)(1063.6935, 1921.74)(1064.694, 1438.19)(1065.6971, 1126.11)(1066.691, 493.52)(508.34, 20491.71)(509.3432, 5946.73)(510.3454, 1268.51)(511.3498, 219.42)(1017.6548, 2365.06)(1018.6313, 741.22)(554.3661, 3107.03)(555.3673, 680.42)(1131.6567, 366.6)(1132.6478, 332.0)(1133.6417, 435.15)(1134.6267, 282.09)	230388	27171.14	241531.3	38423.9	137095.3	27580.07	137757.2	31900.57	-0.30	0.268652	0.366447	-0.33	0.309299	0.61532
COMPOUND_509_3483a10_652478	Rp	-	509.3483	10.65				(1053.6694, 398.93)(1054.6906, 165.33)(1055.6669, 249.65)(1056.6722, 269.42)(1057.6392, 236.3)(622.3592, 364.34)(623.3712, 235.02)(544.3351, 1438.42)(1063.6917, 626.03)(1064.6938, 546.47)(1065.6831, 428.25)(1066.6984, 188.06)(1067.7017, 24.15)(508.3399, 22372.19)(509.3436, 5890.98)(510.3444, 1068.79)(511.3522, 214.71)(1017.666, 2157.4)(1018.6602, 863.74)(554.3696, 4509.39)(555.3658, 1057.47)(1131.6732, 465.88)(1132.6833, 293.3)(1133.6517, 479.1)(1134.6337, 187.68)	238774.1	31336.72	251847.4	37994.47	144790.7	26715.49	139189.9	30494.02	-1.40	1.58E-11	3.68E-10	-0.36	0.073269	0.326093

									1st trimester				3rd trimester									
									Control		Pre-eclampsia		Control		Pre-eclampsia		Time			Interaction		
Compound	Column	Ion_mode	Mass	Retention_	Identification	ID_level	MSMS_spectra	CompositeSpectrum	Mean	SD	Mean	SD	Mean	SD	Mean	SD	Value	raw p	FDR_p	Value	raw p	FDR_p
COMPOUND_509_3488a1_0669302	Hilic	+	509.3488	1.07	Unknown	4	10 eV: 508.3764 (100), 104.1077 (20)	(1019.7023, 56398.47)(1020.7065, 27008.62)(1021.7092, 8191.34)(1022.713, 1952.99)(1023.7131, 451.79)(532.339, 3575.2)(533.3414, 941.83)(534.3542, 1540.7)(535.358, 382.95)(1041.6875, 55438.27)(1042.6914, 29650.83)(1043.704, 94675.53)(1044.7074, 52331.37)(1045.7195, 124627.24)(510.3564, 78683.48)(511.3597, 23029.53)(512.3622, 4078.07)(513.3647, 653.29)(514.3665, 88.4)(1036.7258, 437.69)	#DIV/0!	#DIV/0!	#DIV/0!	#DIV/0!	#DIV/0!	#DIV/0!	#DIV/0!	#DIV/0!	-0.01	0.965487	0.980203	0.78	0.017462	0.194676
COMPOUND_509_3498a1_1393627	Hilic	+	509.3498	1.14				(1019.7027, 105944.28)(1020.7065, 54416.98)(1021.7099, 17542.64)(1022.712, 4148.66)(1023.7102, 920.77)(532.3384, 2413.63)(533.3417, 699.53)(534.3546, 765.51)(535.3567, 210.18)(1041.6871, 36900.3)(1042.6907, 19196.09)(1043.7035, 63281.91)(1044.7072, 34448.86)(1045.7197, 124222.78)(510.3568, 299458.94)(511.3607, 83287.84)(512.3637, 15668.71)(513.3663, 2376.67)(514.3706, 370.17)(1036.7281, 982.37)	2913774	485561.6	2941526	521974.1	1319528	368272.3	1183454	332028	-1.49	3.41E-12	1.07E-10	-0.18	0.359405	0.666906
COMPOUND_509_3503a10_413503	Rp	+	509.3503	10.41	LPC 17:0 isomer	2	10 eV: 510.3573(100), 104.1062(30), 184.073(26)	(1019.6951, 6829.97)(1057.658, 4682.74)(532.3388, 20343.31)(533.3427, 10405.6)(534.3474, 4242.4)(1041.6821, 4376.81)(527.3838, 1947.57)(528.3795, 3774.97)(548.3075, 4588.87)(510.3565, 224461.0)(511.3595, 58941.03)(512.363, 10972.67)(513.3662, 1993.14)	1171403	262889.2	1226992	393840.7	387772.8	173770.7	383220.8	153944.1	-1.59	6.31E-13	3.09E-11	-0.06	0.76986	0.902789
COMPOUND_509_3504a10_411854	Rp	+	509.3504	10.41				(1019.6951, 6829.97)(1057.659, 5208.52)(532.3382, 20568.69)(533.3421, 9889.12)(534.3436, 3789.06)(1041.6802, 4599.28)(548.3075, 4588.87)(527.3743, 1107.65)(528.3847, 4247.14)(510.3565, 224461.0)(511.3595, 58941.03)(512.363, 10972.67)(513.3662, 1993.14)	1053869	273351	1103243	382856.9	401870.1	134863.3	381332.8	145494.3	-1.29	1.89E-08	1.46E-07	-0.30	0.199304	0.510852
COMPOUND_509_351a10_466734	Rp	+	509.3510	10.47	LPC 17:0	2	11 eV: 510.3573(100), 104.1062(30), 184.073(26)	(1019.7024, 2502.18)(1020.7055, 1454.3)(1021.7068, 396.68)(532.3393, 34902.28)(533.3431, 10264.2)(534.3571, 3613.63)(535.3502, 437.74)(1041.6772, 2012.39)(1042.6794, 1480.31)(1043.6549, 76.07)(548.3144, 2425.93)(549.3177, 1404.61)(550.3136, 776.05)(527.3768, 1952.13)(510.3566, 384510.47)(511.3601, 101736.05)(512.3638, 21075.21)(513.3679, 3617.81)	1574266	469388.9	1556930	720922.1	419941	160500.3	377145.4	129800.2	-1.57	1.86E-12	6.88E-11	-0.03	0.883794	0.951974
COMPOUND_509_3861a10_855367	Rp	+	509.3861	10.86				(1019.7932, 1229.89)(1020.7712, 1221.6)(532.3752, 14461.31)(533.379, 3925.58)(534.3917, 4998.39)(535.3947, 1631.31)(527.4464, 377.58)(510.3929, 134935.77)(511.3957, 38378.95)(512.3982, 7192.22)(513.4031, 1273.66)(514.4051, 194.41)	489851.4	161113.3	498710	190630.5	160222	165906.7	120712.2	93879.12	-1.22	1.18E-08	9.6E-08	-0.37	0.095314	0.36381
COMPOUND_510_1233a8_790389	Rp	-	510.1233	8.79				(509.1161, 41548.05)(510.1196, 7896.63)(511.1215, 968.04)(512.133, 197.3)	104220.1	9497.517	105948.8	8898.314	98584	8491.806	102639.9	7641.947	-0.64	0.033043	0.061346	0.28	0.437514	0.714538
COMPOUND_510_2907a11_2283945	Rp	+	510.2907	11.23				(549.2592, 726.65)(550.2638, 407.49)(551.2653, 720.84)(511.2959, 6148.11)(512.2989, 2337.22)(513.2927, 1005.75)(514.299, 140.65)	18996.56	12039.27	13968.16	3579.012	28987.25	11082.56	28440.53	10561	1.11	9.24E-07	4.62E-06	0.08	0.744525	0.889952
COMPOUND_510_377a9_43281	Rp	+	510.3770	9.43				(1021.7289, 91.32)(533.3658, 861.86)(534.3519, 113.01)(535.3733, 1290.58)(549.3304, 284.51)(528.4113, 16754.31)(529.412, 5187.15)(530.4144, 1142.94)(531.4093, 234.37)(511.3843, 2856.15)(512.3865, 919.0)(513.3689, 300.42)(256.1826, 433.9)(256.6961, 134.13)	142365.4	34739.07	129294.3	24431.02	154050.3	25477.56	153207.6	37556.81	0.26	0.370363	0.472821	0.50	0.152875	0.465253
COMPOUND_511_3158a9_286957	Rp	+	511.3158	9.29				(512.323, 29314.56)(513.3264, 8829.42)(514.3348, 2078.21)(515.3336, 349.13)	14749.5	2870.798	26845.5	19403.72	83105	41409.07	82792.98	67472.21	1.41	8.88E-12	2.27E-10	-0.36	0.076628	0.332499
COMPOUND_511_3274a8_750298	Rp	+	511.3274	8.75				(534.319, 1252.25)(535.3228, 398.36)(536.3014, 636.68)(537.2891, 151.34)(512.3277, 32142.38)(513.3302, 9692.27)(514.3335, 2476.16)(515.336, 468.92)	130139.2	38899.03	127585.2	44986.44	187372.8	112419.5	147908.7	81170.79	0.29	0.304002	0.404245	-0.67	0.050772	0.283798
COMPOUND_511_3277a8_590921	Rp	+	511.3277	8.59				(534.3292, 6125.13)(535.328, 2002.47)(512.3354, 5213.97)(513.3394, 1616.42)(514.342, 482.59)	19625.76	7484.578	19386.24	6011.914	14065	2317.866	14251.9	3675.458	-1.19	5.97E-08	3.99E-07	-0.08	0.740788	0.888965
COMPOUND_511_3299a9_88206	Rp	+	511.3299	9.88				(534.3185, 2331.58)(535.3241, 676.71)(536.3147, 301.17)(512.3357, 28177.65)(513.3389, 7725.79)(514.3442, 1840.51)	114583.1	29567.04	108621.8	40487.16	52163.25	6688.069	60189.16	28542.07	-1.58	7.65E-13	3.59E-11	0.35	0.09396	0.36381
COMPOUND_512_2974a8_635713	Rp	-	512.2974	8.64				(511.2904, 30274.98)(512.2936, 8649.85)(513.2958, 2062.62)(514.2979, 405.7)	62784.2	25817.84	62804.84	30600.85	213237.1	61633.1	175473	100936.9	1.66	1.3E-10	1.88E-09	-0.36	0.155807	0.466545
COMPOUND_513_2754a9_351753	Rp	-	513.2754	9.35				(1071.5552, 119.73)(1072.5687, 124.23)(512.2682, 11744.09)(513.2717, 3527.03)(514.2713, 1182.03)(515.2678, 192.78)(255.6308, 2720.49)(256.132, 814.23)(256.6317, 296.7)	192051.7	243871.9	181209.9	130189.4	119436.6	108037.8	93382.85	66992.58	-0.51	0.032515	0.060615	-0.44	0.110802	0.396703
COMPOUND_513_2796a5_9541445	Rp	+	513.2796	5.95				(1027.5135, 107.58)(514.2871, 106462.85)(515.2901, 25982.53)(516.2924, 5417.08)(517.2955, 1087.9)	157109.8	122643.8	190166.8	142196	218486.9	156140.8	191681.4	129382.5	0.45	0.141805	0.218132	-0.31	0.396553	0.690408
COMPOUND_513_3054a9_622855	Rp	-	513.3054	9.62	LPC 14:0	1	10 eV: 452.2769(100), 512.2986(42), 227.2012(10), 44.9981(7)	(1071.6064, 140.59)(1072.6204, 105.5)(512.2981, 49025.72)(513.3011, 12178.05)(514.3033, 2407.46)(515.3072, 433.14)(516.3118, 43.61)	93830.12	50198.35	127436.6	66685.46	118443.9	80706.87	86328.15	56360.56	0.42	0.141293	0.217493	-1.14	0.001389	0.059009
COMPOUND_513_3059a9_766879	Rp	-	513.3059	9.77	LPC 14:0 isomer	3	10 eV: 452.2769(100), 512.2986(42), 227.2012(10), 44.9981(7)	(1071.6272, 60.13)(512.2985, 375752.28)(513.302, 92454.06)(514.3042, 16817.23)(515.3069, 2741.51)(516.3078, 303.26)	571460.8	284988.2	801812.9	432757.7	608628.3	215277.1	476258.1	252116.7	0.15	0.56304	0.660055	-1.11	0.000739	0.045795
COMPOUND_513_3317a8_852228	Rp	+	513.3317	8.85				(552.3224, 807.58)(531.3756, 1341.23)(532.3685, 233.98)(514.3386, 14527.38)(515.3416, 4955.11)(516.3459, 1227.01)(517.3452, 252.58)(518.363, 36.73)	82241.49	29502.19	88099.21	38976.69	333685.1	142236	325993.3	161506.1	1.44	1.07E-10	1.59E-09	0.11	0.593036	0.803855
COMPOUND_513_7017a10_334726	Rp	+	513.7017	10.33				(1066.3882, 359.85)(1067.3962, 314.4)(1068.3846, 340.22)(536.6968, 412.97)(537.7156, 317.48)(514.7081, 11679.82)(515.7088, 771.22)(516.6949, 705.05)	48342.88	18641.26	39251.63	14152.79	52078.08	10946.8	53300.85	16477.89	0.25	0.371765	0.474075	0.62	0.067317	0.315172
COMPOUND_514_0258a9_951875	Rp	+	514.0258	9.95				(537.0279, 420.9)(538.031, 759.44)(1051.042, 141.27)(532.0343, 522.31)(515.0346, 8828.17)(516.0368, 1415.48)(517.02, 1760.4)(518.0096, 245.35)(519.0307, 658.53)	73573	26320.92	63133.81	26695.29	72456.85	11386.02	74595.78	18252.63	-0.03	0.904073	0.937804	0.75	0.032307	0.244873
COMPOUND_514_4067a13_4083	Rp	+	514.4067	13.41				(537.3966, 383527.8)(538.3997, 121307.84)(539.3987, 36805.98)(540.3993, 7552.95)(1051.7996, 2930.24)(1052.8044, 2248.6)(1053.8044, 992.47)(1054.8127, 427.3)(1055.809, 127.38)(553.3721, 19544.53)(554.3756, 7042.0)(555.3731, 3983.74)(556.3711, 861.92)(532.4398, 1133238.0)(533.4441, 372903.5)(534.4432, 103336.23)(535.4436, 22214.72)(536.4439, 3485.12)(515.4133, 1189855.5)(516.4179, 379318.5)(517.4168, 105856.15)(518.4167, 22623.58)(519.4182, 3130.08)	55050943	10226918	50721739	8809059	55807143	5942217	56843860	7845404	0.12	0.687005	0.771253	0.54	0.132458	0.431688
COMPOUND_515_1731a10_66196	Rp	-	515.1731	10.66				(550.1548, 815.74)(551.1458, 531.92)(552.1521, 357.93)(514.1656, 6661.06)(515.1677, 2469.29)(516.1673, 1181.05)(517.1799, 242.41)	19489.08	4662.294	18619.57	5500.5	19577.09	6656.883	20041.45	6152.985	-0.49	0.0952	0.156261	0.65	0.058999	0.30009
COMPOUND_515_2909a7_4732437	Rp	-	515.2909	7.47				(514.2835, 51324.06)(515.2871, 16013.7)(516.2858, 5002.46)(517.2861, 960.33)	29564.93	24795.27	27702.93	18983.05	95369.6	106335.4	73494.98	62938.62	0.66	0.001767	0.004425	0.21	0.378258	0.681469
COMPOUND_515_2911a8_730288	Rp	-	515.2911	8.73				(1075.5514, 524.23)(1076.5552, 378.0)(514.2841, 306272.16)(515.2874, 87106.54)(516.2864, 26711.29)(517.2868, 5555.29)(518.2876, 864.57)(1029.5757, 28789.9)(1030.5785, 17006.33)(1031.5784, 7876.75)(1032.5786, 2912.65)(1033.5759, 890.36)	145999.9	458780.4	95037.84	127538.5	2345539	7220578	1498032	2507149	1.10	1.69E-06	8.07E-06	0.26	0.293533	0.603136
COMPOUND_515_3049a1_2238518	Hilic	+	515.3049	1.22				(1031.6315, 923.22)(1032.6498, 514.28)(1053.6196, 655.57)(533.3417, 851.12)(516.3066, 10083.76)(517.31, 3047.47)(1048.6256, 51.92)(1049.6616, 292.54)(1050.6708, 291.0)	59115.74	12808.2	64271.61	17347.88	66259.93	11748.89	63756.38	14205.5	0.77	0.007265	0.015916	-1.06	0.002306	0.067469
COMPOUND_515_4188a11_848802	Rp	+	515.4188	11.85				(1069.8168, 1744.7)(516.4277, 15136.22)(517.4303, 5469.19)(518.4337, 1188.41)(1048.8715, 259.79)	21075.88	5083.084	21532.53	5687.701	18354.73	5024.695	21141.88	12271.71	-0.35	0.204404	0.295398	0.15	0.659575	0.842895
COMPOUND_517_299a9_091623	Rp	-	517.2990	9.09				(516.293, 12883.21)(517.2951, 3627.22)(518.2894, 778.54)(519.302, 160.31)(1033.58, 407.46)(1034.5764, 175.04)(562.2972, 1266.26)(563.2963, 362.78)(564.2758, 1493.1)(565.2779, 806.27)(566.2737, 195.37)	34732.2	19329.57	32826.58	13487.24	30458.64	15709.05	31507.5	28286.71	-0.07	0.790272	0.84749	0.03	0.922142	0.965124
COMPOUND_517_3131a10_156097	Rp	+	517.3131	10.16				(1035.6342, 1397.34)(1036.6289, 914.46)(529.3024, 2929.41)(529.8033, 723.63)(530.3145, 5072.4)(530.798, 1131.51)(1057.6053, 1827.28)(1058.6263, 1930.87)(1059.6268, 1804.18)(556.2737, 994.32)(556.7814, 861.86)(557.2834, 1208.11)(281.6355, 1016.26)(282.1341, 526.43)(518.3232, 143501.31)(519.326, 37923.53)(1052.6566, 1624.87)(259.6598, 4102.79)(260.1608, 1989.2)(260.6593, 991.39)(261.1549, 182.37)	550294.7	236048.3	535093	226553.5	381544.2	66587.46	447637.8	141808	-0.24	0.363118	0.465155	0.63	0.04247	0.268638

									1st trimester				3rd trimester									
									Control		Pre-eclampsia		Control		Pre-eclampsia		Time			Interaction		
Compound	Column	Ion_mode	Mass	Retention_	Identification	ID_level	MSMS_spectra	CompositeSpectrum	Mean	SD	Mean	SD	Mean	SD	Mean	SD	Value	raw p	FDR_p	Value	raw p	FDR_p
COMPOUND_517_3162a1_1540976	Hilic	+	517.3162	1.15				(1035.6368, 629.76)(1036.6412, 481.8)(1037.6552, 11728.55)(540.3075, 1204.05)(541.3119, 452.71)(542.3229, 8465.61)(543.3259, 2835.84)(1057.6355, 480.38)(1058.6322, 504.76)(1059.6366, 418.43)(1060.651, 238.33)(518.3233, 124558.33)(519.3266, 34056.87)	1630681	544386.9	1702967	649648.2	1541913	229976.5	1540287	259118.5	-0.09	0.756506	0.82302	-0.14	0.702676	0.86442
COMPOUND_517_318a9_845915	Rp	+	517.3180	9.85	LPC 18:3	2	10 eV: 518.3241(100), 104.107(18)	(1035.6384, 219.21)(1036.6392, 204.79)(1037.6555, 140.86)(540.3039, 7344.48)(541.3042, 3413.71)(556.2869, 777.25)(518.3256, 124474.67)(519.3289, 35002.93)(520.3333, 7486.69)(521.3389, 854.66)	352557.3	172549.6	403034.1	208796.8	251813.3	93958.06	220031.3	100639	-0.73	0.004247	0.00973	-0.34	0.238832	0.543832
COMPOUND_517_329a10_529172	Rp	-	517.3290	10.53				(1079.6473, 369.66)(1080.665, 351.76)(516.3213, 6802.36)(517.3249, 2087.68)(518.3196, 2534.35)(519.3211, 748.36)(520.3045, 1235.97)	42393.19	8462.973	43648.36	10993.91	24132.75	15487.86	20193.9	9135.36	-1.25	2.46E-08	1.83E-07	-0.22	0.338203	0.642187
COMPOUND_518_2862a8_854052	Rp	+	518.2862	8.85				(541.3004, 420.51)(542.2871, 211.83)(557.2534, 320.26)(558.2555, 74.63)(519.2938, 13178.34)(520.297, 3910.27)(521.2975, 1081.57)(522.3092, 199.78)	#DIV/0!	#DIV/0!	#DIV/0!	#DIV/0!	#DIV/0!	#DIV/0!	#DIV/0!	#DIV/0!	1.50	1.04E-12	4.7E-11	0.00	0.993167	0.997491
COMPOUND_519_3324a1_1471411	Hilic	+	519.3324	1.15				(1039.6727, 21502.4)(1040.676, 12284.19)(1041.6888, 81182.96)(1042.6912, 46807.38)(542.3248, 441055.03)(543.329, 124839.65)(1061.6576, 8610.87)(1062.6602, 5096.96)(537.3769, 1794.46)(520.3397, 869338.75)(521.3441, 236528.58)(522.3552, 3288982.8)	13824656	4909357	14473183	4207194	9239677	2466730	8553470	2707035	-0.98	0.000116	0.000354	-0.33	0.246264	0.555348
COMPOUND_519_333a1_0411083	Hilic	+	519.3330	1.04				(542.3245, 70841.04)(543.3276, 20542.25)(1061.6556, 5886.15)(1062.6586, 3560.5)(520.3396, 754564.06)(521.3437, 203585.92)(1056.7023, 640.31)(1057.6832, 1001.01)	1691489	674244	1830499	572272.1	897008.8	236868.1	828787.7	306399.1	-1.18	5.03E-07	2.72E-06	-0.36	0.156518	0.46686
COMPOUND_519_3341a10_012239	Rp	+	519.3341	10.01				(1039.6724, 36113.35)(1040.6759, 19284.49)(1041.675, 7506.11)(1042.6912, 437.04)(1077.606, 975.01)(1078.6117, 2527.18)(1079.6162, 1674.84)(542.3231, 54205.82)(543.3257, 16910.29)(1061.6501, 8221.14)(1062.6512, 4836.95)(1063.6674, 17986.58)(1064.6726, 9727.92)(1065.6749, 5191.04)(1066.6741, 2825.08)(558.296, 4080.68)(559.3007, 2253.22)(560.3008, 1687.92)(537.3803, 1962.97)(520.3408, 1235781.1)(521.3459, 328856.3)(522.3476, 59266.63)(523.3436, 10527.79)	2943646	3326904	3255308	4524378	1190243	1395285	2870652	4114157	-0.99	0.000345	0.000984	0.13	0.682911	0.853695
COMPOUND_519_3356a10_106271	Rp	+	519.3356	10.11	LPC 18:2	2	10eV: 520.3395(100), 184.0736(90), 104.1083(4), 258.1018(2)	(1039.6685, 5066.64)(1040.6703, 2961.41)(1041.6864, 8704.23)(1042.6907, 4310.57)(1043.6918, 1853.47)(1077.5944, 2826.01)(542.3238, 63893.44)(543.3271, 18710.74)(1061.6501, 8221.14)(1062.6512, 4836.95)(1063.6674, 17986.58)(1064.6726, 9727.92)(1065.6749, 5191.04)(537.3721, 2096.39)(538.3743, 1373.35)(558.2945, 5591.27)(520.3401, 1333381.0)(521.3454, 361394.38)(522.3476, 65406.29)(523.3494, 4707.87)(1056.635, 3074.81)(1057.6375, 950.36)	16365627	5118735	16600003	4672088	10449789	2540909	9942399	3408144	-1.15	3.25E-06	1.44E-05	-0.15	0.556783	0.781554
COMPOUND_519_4676a12_437243	Rp	+	519.4676	12.44				(520.4723, 23735.97)(521.4753, 8806.24)(522.4794, 1636.7)(523.4838, 242.82)	38763.05	29811.58	24846.32	15085.51	27252.44	9346.254	32020.86	20019.77	-0.32	0.219919	0.31355	0.62	0.041462	0.265944
COMPOUND_520_4106a10_754911	Rp	+	520.4106	10.75				(543.3921, 2816.09)(544.3809, 1917.6)(545.3776, 730.03)(538.4246, 864.43)(521.4211, 11852.47)(522.4234, 4295.23)(523.4286, 1506.66)	46607.6	27818.46	45655.56	18094.92	64839.85	40686.09	53090.06	34390.74	0.60	0.030587	0.057307	-0.66	0.041202	0.265944
COMPOUND_520_4489a12_670413	Rp	+	520.4489	12.67				(1041.932, 367.95)(1042.9244, 317.33)(1043.9117, 540.56)(1079.8967, 198.84)(521.4579, 41933.42)(522.4608, 14594.03)(523.4641, 3046.73)(524.4704, 550.41)(1058.9426, 620.4)(1059.9446, 470.61)(1060.9396, 388.63)	59943.57	35211.42	62839.91	37213.28	54778.64	18341.3	90951.68	51647.01	0.11	0.671604	0.758882	0.65	0.045136	0.273371
COMPOUND_521_3482a1_1173073	Hilic	+	521.3482	1.12				(1043.702, 21934.41)(1044.7068, 11460.52)(1045.7202, 82030.55)(1046.724, 46576.91)(1047.7362, 342104.4)(1048.7397, 194042.16)(1049.7423, 61991.49)(1050.7448, 14818.45)(1051.7463, 3565.73)(1052.72, 820.31)(544.3401, 1355353.0)(545.3445, 377928.0)(1065.6865, 25891.92)(1066.6901, 13115.49)(1067.7037, 81354.4)(1068.7074, 46303.08)(1069.717, 57934.67)(1070.7213, 29600.16)(1071.7305, 17605.84)(1072.7349, 6160.95)(1073.7465, 6033.33)(539.3995, 9737.8)(540.3938, 1649.92)(522.3548, 1149114.9)(523.3597, 279626.5)(1060.7646, 2529.13)	23575472	3356790	23597826	3381090	12698823	2414974	13573768	3342102	-1.70	1.22E-14	2.11E-12	0.13	0.51501	0.761025
COMPOUND_521_349a10_368553	Rp	+	521.3490	10.37	LPC 18:1	2	10eV: 522.3558(100), 184.078(50), 104.1087(15), 366.6751(6), 240.0936(4)	(1043.7034, 16613.24)(1044.7063, 10031.07)(1045.7087, 3796.67)(1046.7017, 1270.59)(1047.7046, 410.38)(1081.6136, 730.71)(1082.6215, 503.83)(1083.6224, 634.4)(544.339, 51415.71)(545.3421, 15101.63)(546.3551, 20301.62)(547.3583, 5621.64)(548.3708, 5829.9)(1065.6835, 4365.24)(1066.6876, 2849.35)(1067.6873, 3010.23)(1068.6807, 1507.61)(1069.7002, 515.88)(560.3122, 4181.32)(561.3124, 1595.95)(562.3186, 8262.27)(563.3173, 5589.42)(539.3807, 1579.66)(522.3572, 1229078.4)(523.3617, 328683.1)(524.3636, 58481.22)(525.3664, 8880.36)(526.366, 1432.56)	20258491	3308057	19528261	3940314	10947889	2395487	11900727	3134930	-1.74	1.66E-13	1.25E-11	0.32	0.134363	0.43508
COMPOUND_521_3493a1_0073508	Hilic	+	521.3493	1.01				(1043.7024, 8615.11)(1044.7067, 4637.08)(1045.7188, 16526.84)(1046.7217, 9051.48)(1047.7336, 16827.03)(1048.7382, 8806.6)(1049.74, 2784.53)(1050.7368, 839.43)(1051.7181, 312.97)(544.3404, 157027.1)(545.3437, 48177.14)(546.3556, 138752.39)(547.3592, 43072.89)(548.3678, 15795.34)(1065.6873, 13948.94)(1066.6912, 7208.66)(1067.7034, 17147.85)(1068.7074, 9930.56)(1069.7174, 11133.43)(1070.7214, 5194.06)(1071.7275, 2691.22)(1072.728, 1106.07)(1073.7393, 889.53)(522.3554, 609957.56)(523.3594, 161500.36)(1060.7684, 1014.83)	2738550	592942	2832210	602058.6	1297271	367085.7	1342191	389984	-1.58	4.61E-13	2.6E-11	-0.06	0.780935	0.909341
COMPOUND_521_3501a10_274176	Rp	+	521.3501	10.27				(544.3392, 65696.72)(545.3432, 18387.91)(1065.6855, 10840.7)(1066.6884, 6210.83)(1067.6982, 5367.55)(560.3118, 4078.06)(561.3179, 1352.02)(539.3878, 9978.71)(540.3803, 1461.94)(522.3572, 919051.9)(523.3615, 246616.97)(524.3632, 44236.02)(525.3669, 7855.4)	1473495	2724971	1040784	219719.8	698238.6	82332.39	930347.2	1210631	-1.81	5.49E-12	1.53E-10	0.71	0.005699	0.118614
COMPOUND_521_792a10_213077	Rp	+	521.7920	10.21				(1044.5842, 32684.63)(1045.589, 20023.68)(1046.5958, 7738.09)(1047.6019, 3330.07)(544.7929, 692.72)(1066.6277, 4072.65)(1067.6108, 2434.82)(560.7507, 1038.65)(522.7984, 44536.32)(523.8031, 29192.87)(524.8035, 14621.86)(1061.6508, 6360.56)(1062.6553, 4391.86)	511531.1	212380.8	480643	230132.2	537183.7	261445.7	516045.2	259793.7	0.12	0.68039	0.765736	0.29	0.414013	0.696309
COMPOUND_522_3295a12_530789	Rp	-	522.3295	12.53				(557.313, 177.62)(558.3154, 282.56)(521.3218, 13860.63)(522.3222, 8890.68)(523.3246, 2231.1)(524.3256, 418.52)(567.3468, 246.17)(1043.6166, 460.5)(1044.6187, 342.07)	91937.91	9901.396	88079.24	12556.71	94828.21	8871.161	94008.32	12056.1	0.28	0.32212	0.423801	0.29	0.399547	0.690408
COMPOUND_522_4667a13_280933	Rp	+	522.4667	13.28				(1045.9412, 7322.95)(1046.941, 4343.5)(1047.9431, 1852.9)(1048.9398, 506.48)(523.4732, 19825.12)(524.4762, 7199.14)(525.48, 1606.95)(526.4807, 262.33)	123196.6	60084.04	158168.5	68708.54	156105.4	77745.94	141639.7	66815.93	0.62	0.037176	0.0679	-0.79	0.026777	0.225849
COMPOUND_523_2641a6_903725	Rp	+	523.2641	6.90				(524.2712, 6298.91)(525.2748, 1639.96)(526.2524, 3522.67)(527.2556, 965.91)(528.2577, 296.07)	30659.59	8130.808	30133.81	6399.25	28664.36	9121.776	32525.62	8089.008	-0.04	0.890506	0.926471	0.23	0.500899	0.753664
COMPOUND_523_3634a1_0992484	Hilic	+	523.3634	1.10				(1047.7318, 28528.98)(1048.7362, 13221.49)(1049.7393, 4154.69)(1050.7379, 1246.15)(1051.7214, 270.12)(546.3544, 159531.27)(547.3577, 42156.74)(548.3708, 67822.28)(549.374, 19664.15)(1069.7142, 8625.35)(1070.7194, 3966.51)(1071.7227, 1683.39)(1072.7223, 555.26)(541.4061, 721.69)(524.3704, 1464778.1)(525.3752, 374053.94)(526.3778, 67731.24)(527.3792, 8100.69)(528.3858, 1095.13)(1064.798, 656.19)	25735849	4111573	25869565	3977261	10949717	2651341	10724784	2692486	-1.56	6.74E-14	6.9E-12	-0.07	0.720192	0.876947
COMPOUND_523_3639a0_9970972	Hilic	+	523.3639	1.00				(1047.7333, 10931.65)(1048.7369, 5532.55)(1049.7378, 1860.72)(1050.7245, 784.7)(1051.7522, 81.54)(546.3547, 44676.52)(547.3582, 13109.07)(548.368, 5885.24)(549.3728, 1311.09)(1069.7145, 6600.01)(1070.7189, 3063.48)(1071.7268, 1894.26)(1072.7288, 794.76)(524.3706, 750840.6)(525.375, 204271.27)(526.3772, 36813.25)(527.38, 5937.3)(528.3841, 768.46)	3371288	812641	3477854	696156.3	1243266	364751.5	1163593	333595.9	-1.47	5.94E-13	2.98E-11	-0.20	0.278347	0.585315

									1st trimester				3rd trimester									
									Control		Pre-eclampsia		Control		Pre-eclampsia		Time			Interaction		
Compound	Column	Ion_mode	Mass	Retention_	Identification	ID_level	MSMS_spectra	CompositeSpectrum	Mean	SD	Mean	SD	Mean	SD	Mean	SD	Value	raw p	FDR_p	Value	raw p	FDR_p
COMPOUND_523_3652a10_640335	Rp	+	523.3652	10.64	LPC 18:0	2	10 eV: 524.3745(100), 184.073(70), 104.1067(3)	(1047.74, 18668.87)(1048.744, 10191.32)(1049.7434, 3755.41)(1050.7451, 1269.58)(1051.7399, 389.8)(1085.6648, 647.91)(1086.6654, 423.12)(1087.6859, 464.11)(1088.7122, 515.41)(546.356, 184363.2)(547.3587, 48464.29)(548.3629, 9627.82)(549.3706, 2105.26)(1069.7191, 3020.89)(1070.7155, 1819.39)(1071.7051, 1481.02)(1072.7054, 512.89)(541.3874, 1505.51)(542.4009, 655.55)(543.3706, 6008.54)(562.3281, 6255.94)(563.3327, 2177.15)(564.3326, 1424.4)(565.3209, 242.6)(524.3723, 1472801.6)(525.3767, 402439.5)(526.3791, 65702.59)(527.3812, 11436.44)(1064.724, 668.49)	24650943	4250413	23647826	4484429	10706563	3239046	10564884	3129403	-1.64	9.71E-14	9.22E-12	0.10	0.596494	0.805251
COMPOUND_523_3662a10_548693	Rp	+	523.3662	10.55	LPC 18:0 isomer	2	10 eV: 524.3745(100), 184.073(70), 104.1067(3)	(1047.7385, 11020.18)(1048.741, 6199.9)(1049.7426, 2502.8)(1050.742, 818.39)(1051.7325, 111.43)(1085.6538, 505.47)(1086.6392, 424.35)(546.3557, 131120.62)(547.3587, 37092.9)(548.3625, 8067.74)(1069.7107, 1941.05)(1070.7191, 1325.57)(1071.721, 901.7)(1072.7257, 400.7)(1073.7327, 66.68)(562.3288, 5306.5)(563.3301, 2081.29)(564.3297, 1404.77)(524.3732, 783535.4)(525.3772, 211042.19)(526.3794, 39284.11)(527.3809, 5907.48)(528.3845, 726.78)	3848738	891366.9	4090050	2574789	2280830	3069334	1684168	2859787	-1.06	6.44E-06	2.68E-05	-0.36	0.157546	0.468065
COMPOUND_523_7058a10_204601	Rp	+	523.7058	10.20				(546.691, 473.79)(547.7085, 478.7)(524.7117, 15649.51)(525.7137, 1130.09)(526.6979, 814.47)	55060.31	24182.92	44815.58	21354.51	58988.54	11211.26	62242.22	19304.21	0.23	0.419877	0.524149	0.67	0.048994	0.280039
COMPOUND_525_2845a10_05921	Rp	-	525.2845	10.06				(524.2778, 251396.61)(525.2811, 72879.71)(1049.5616, 4144.25)(1050.563, 2464.92)	273142.4	205735.1	287159.1	250016.9	235648.6	214626.5	219202.6	237372.1	-0.39	0.194888	0.283887	0.10	0.790949	0.911108
COMPOUND_525_2851a1_3956394	Hilic	+	525.2851	1.40				(548.2751, 2352.06)(549.278, 723.5)(550.2895, 372.31)(551.2964, 35.61)(526.2921, 2180.79)(527.2953, 682.01)(528.3019, 4289.5)(529.3062, 1330.71)	26965.9	6878.425	29446.96	9341.161	21991.23	8211.094	27677.22	12252.18	-0.64	0.03303	0.061346	0.36	0.304921	0.613089
COMPOUND_525_2852a10_103805	Rp	-	525.2852	10.10	LPE 22:6	2	10 eV: 524.2775(100), 327.2313(18)	(1085.5209, 415.97)(1086.5138, 372.32)(524.2777, 243590.89)(525.2809, 70133.47)(526.2917, 16357.06)(527.2895, 2377.26)(1049.5616, 4144.25)(1050.563, 2464.92)(1051.5704, 836.8)(1052.576, 203.74)(1163.5862, 1122.63)(1164.5807, 600.3)(1165.6119, 1506.42)	545500.2	137636.2	568914.3	196032.8	434123.3	185830.6	559061.5	242237.5	-0.74	0.014462	0.029634	0.60	0.090948	0.358068
COMPOUND_525_2853a1_586687	Hilic	+	525.2853	1.59				(1051.5754, 942.98)(1052.5809, 592.02)(1053.5894, 268.78)(1054.5936, 115.52)(548.275, 10110.02)(549.2788, 3036.97)(550.2898, 3107.6)(551.2937, 972.56)(1073.5565, 1106.09)(1074.5638, 707.89)(1075.5696, 333.67)(543.3251, 581.54)(526.2931, 14655.7)(527.2963, 4079.57)(528.3061, 1874.34)(529.31, 407.29)	190245.9	48173.66	205790.1	71427.75	141554.6	56353.13	181744.7	78673.7	-0.89	0.002935	0.007008	0.46	0.178081	0.492203
COMPOUND_525_2865a10_022741	Rp	+	525.2865	10.02				(1051.5656, 4598.59)(1052.5752, 2601.52)(1053.5928, 1808.98)(548.2959, 3215.48)(549.2932, 1405.28)(550.2965, 1403.08)(1073.6085, 1558.32)(564.2497, 1708.76)(565.2574, 520.97)(526.2944, 171313.03)(527.2982, 50787.09)(528.301, 11821.77)(529.3002, 1991.9)(1068.5927, 9058.52)(1069.6084, 9193.84)	119410.9	102119.2	119803.7	95606.47	76620.5	29084.05	173013.9	262651.4	-0.80	0.008009	0.017393	0.68	0.053256	0.287606
COMPOUND_525_2878a10_106169	Rp	+	525.2878	10.11	LPE 22:6	2	10eV: 526.2943(100), 35.2722(40), 62.0587(32), 44.0482(22), 198.0479(8)	(1051.5732, 4110.93)(1052.5806, 2803.67)(1053.5944, 2428.1)(548.2849, 990.35)(549.2984, 646.65)(550.3013, 831.58)(1073.6125, 1814.39)(564.2483, 1902.47)(565.2572, 1059.04)(526.2949, 195116.61)(527.2985, 61771.38)(528.3011, 12970.79)(529.3002, 2569.37)(1068.591, 10096.16)(1069.6013, 9016.42)	513694.6	126090.3	474893.7	182535.3	482294	165494.4	619673.5	233355.4	-0.26	0.379329	0.480996	0.90	0.012445	0.169858
COMPOUND_525_3427a10_452606	Rp	-	525.3427	10.45	LPC P-16:0	2	10 eV: 464.3136(100), 524.3348(30), 141.4541(3)	(1085.6787, 506.21)(1086.6794, 382.32)(1095.7026, 574.81)(1096.7212, 503.97)(1097.7172, 450.22)(524.3354, 149773.0)(525.3383, 39419.31)(526.3437, 11083.53)(527.348, 1681.05)(1049.6766, 866.3)(1050.6781, 534.64)(1051.6659, 563.61)(1052.6617, 334.69)(1053.6571, 376.27)(1163.6283, 661.78)(1164.6166, 370.88)	685595.6	131185	707287.3	151431.1	252223.5	143935.4	232295.4	126220.2	-1.69	7.2E-12	1.95E-10	0.28	0.228504	0.535521
COMPOUND_527_3009a10_237866	Rp	-	527.3009	10.24	LPE 22:5	2	20 eV: 329.248(100), 526.2932(37), 196.0365(7), 53.0058(4), 440.0885(4)	(1089.5819, 3181.16)(1090.5854, 1771.54)(1091.5836, 2938.65)(526.2936, 19072.98)(527.2964, 5609.15)(528.3003, 1151.08)(529.3007, 239.46)(1167.6259, 981.61)(1168.6296, 433.72)	86221.6	33458.79	91104.02	38665.74	78929.36	32494.58	88863.11	23469.44	-0.27	0.362601	0.464757	0.20	0.582594	0.796117
COMPOUND_527_301a10_300283	Rp	-	527.3010	10.30				(1089.5807, 2205.98)(1090.5831, 1283.83)(1091.5826, 2106.18)(526.2936, 19072.98)(527.2964, 5609.15)(528.3003, 1151.08)(529.3007, 239.46)(1167.6075, 752.35)(1168.6068, 389.09)	48130.28	28653.4	53682.31	40200.71	45885.15	30324.02	42427.81	20071.66	-0.35	0.244546	0.339945	0.22	0.546392	0.77815
COMPOUND_527_3103a8_223662	Rp	+	527.3103	8.22				(528.3185, 23057.2)(529.3213, 6738.28)(530.3259, 1664.11)	#DIV/0!	#DIV/0!	#DIV/0!	#DIV/0!	44807	37355	27623.95	18921.86	1.33	2.4E-11	5.2E-10	-0.33	0.09665	0.36704
COMPOUND_527_3208a9_9053335	Rp	-	527.3208	9.91				(526.3143, 90461.38)(527.3176, 22764.48)(528.3175, 5519.24)	96060.19	26164.2	118681.5	65630.41	89642.57	118648.9	55920.91	23295.65	-0.84	0.000358	0.001017	-0.75	0.005826	0.118614
COMPOUND_527_3211a10_038401	Rp	-	527.3211	10.04	LPC 15:0	1	10 eV: 466.2927(100), 526.3123(45), 241.217(13)	(526.3144, 124962.4)(527.3174, 32728.1)(528.3158, 8680.55)(529.3123, 1757.21)(530.3092, 233.54)	469972.5	120944.7	516201.5	147295.3	263526.5	77413.79	220212.5	79892.71	-1.22	4.27E-09	3.86E-08	-0.50	0.019664	0.198675
COMPOUND_527_3574a10_531313	Rp	-	527.3574	10.53	LPC O-16:0	2	10eV: 466.3301(100), 526.3495(60), 231.6412(3), 112.9849(3)	(562.3356, 449.41)(526.3509, 162402.23)(527.3542, 45608.22)(528.3566, 8664.5)(529.3617, 1527.37)	693196.1	151674.2	714493.1	194632	267222.5	184021.8	234234.9	139707.1	-1.36	1.71E-09	1.76E-08	-0.22	0.330727	0.636557
COMPOUND_528_0424a10_324152	Rp	+	528.0424	10.32				(551.0315, 355.23)(552.0468, 876.32)(529.0501, 13578.04)(530.053, 2312.98)(531.0368, 2404.91)(532.0264, 218.84)(533.0413, 919.9)	66390.96	26908.96	53049.16	20666.42	75482.92	14527.58	74788.33	22836.46	0.31	0.264282	0.361362	0.62	0.068056	0.317972
COMPOUND_528_4232a13_922085	Rp	+	528.4232	13.92				(551.4111, 2578.58)(552.4166, 1047.87)(553.397, 412.62)(554.4027, 43.46)(567.407, 1012.35)(568.4103, 173.41)(546.4564, 7621.28)(547.4582, 2663.56)(548.459, 645.59)(529.4302, 7804.79)(530.4341, 2544.14)(531.4334, 890.95)(1074.8502, 141.49)	98885.77	18450.5	97001.48	22977.95	102739.4	11937.19	103803.4	14841.2	0.22	0.466098	0.570463	0.25	0.486282	0.747178
COMPOUND_528_702a9_944124	Rp	+	528.7020	9.94				(551.7078, 619.66)(529.7102, 12506.46)(530.7125, 1929.83)(531.6966, 1968.23)(532.6923, 287.04)(533.7051, 964.11)	63202.48	24245.85	54234.53	24174.94	64603.23	13150.15	62985.8	17580.1	0.08	0.770819	0.833358	0.54	0.121374	0.411649
COMPOUND_529_0355a9_946081	Rp	+	529.0355	9.95				(1059.0917, 138.81)(552.019, 764.43)(553.0158, 100.78)(547.0471, 323.17)(567.9837, 184.55)(568.9863, 119.24)(530.0449, 12442.26)(531.0443, 735.83)(532.031, 1294.21)(1076.1566, 584.44)(1077.1298, 375.38)	48554.63	20008.24	43678.05	20751.63	45399.85	12519.55	46360.4	13741.81	-0.11	0.705332	0.784399	0.44	0.211453	0.519294
COMPOUND_529_2701a8_816477	Rp	-	529.2701	8.82				(528.2629, 12002.27)(529.2659, 3725.47)(530.2648, 1127.07)(531.2654, 229.82)(263.628, 791.96)(264.1294, 243.04)(264.6274, 77.23)	102590.6	102800.1	104433.3	83833.42	211879.7	158585.1	126147	93477.14	0.68	0.006815	0.015002	-0.58	0.044551	0.272788
COMPOUND_529_2702a8_639005	Rp	-	529.2702	8.64				(528.263, 14272.67)(529.2663, 4378.11)(530.2647, 1281.82)(531.2564, 194.36)(263.6283, 3389.62)(264.1301, 981.24)(264.6294, 420.52)	35882.77	38164.84	37492.39	32490.23	197426.3	492278.3	54985.5	107674.5	0.83	0.003555	0.008314	-0.66	0.046226	0.273966
COMPOUND_530_1842a9_033124	Rp	-	530.1842	9.03				(1105.3414, 317.47)(1106.3588, 120.92)(1107.3555, 129.41)(529.178, 5879.92)(530.1811, 1548.63)(531.18, 818.92)(532.1806, 158.28)(1059.3572, 261.14)	14740.06	3387.914	16560.7	5758.249	49030.86	33497.44	39126.2	19270.93	1.59	2.39E-12	7.99E-11	-0.27	0.203461	0.5149
COMPOUND_530_1965a11_19501	Rp	+	530.1965	11.20				(553.1864, 559.9)(569.1538, 2548.5)(531.203, 2941.64)(532.2057, 1152.58)(533.1863, 10838.52)(534.188, 3426.94)(1078.4271, 848.27)	32610.29	12358.28	23843.88	8095.714	51327	15321.7	55737.61	17416.71	0.77	0.001581	0.003982	0.74	0.009653	0.153413
COMPOUND_530_4028a11_883773	Rp	+	530.4028	11.88				(1061.7737, 465.2)(1062.7631, 349.84)(1063.7789, 265.33)(1099.8055, 365.3)(1100.7847, 2000.69)(1101.788, 1936.92)(553.3919, 136343.48)(554.3949, 43676.13)(555.3936, 13752.78)(556.3948, 2822.44)(557.3868, 313.02)(1083.792, 36763.55)(1084.7957, 24690.98)(1085.7953, 11938.62)(1086.7961, 4504.12)(1087.7955, 1534.47)(1088.7979, 384.81)(569.3661, 13047.09)(570.3692, 4190.35)(571.3648, 2390.51)(572.3622, 570.92)(548.4115, 382.65)(531.4089, 832401.94)(532.4131, 260824.56)(533.4122, 75112.47)(534.4127, 16504.04)(535.416, 2471.59)(1078.8323, 516.08)	4779299	1428579	4594954	1312184	975225.6	1300041	714101.3	950012	-1.56	5.8E-11	1E-09	0.04	0.849328	0.936215
COMPOUND_530_4689a14_097955	Rp	-	530.4689	14.10				(565.4388, 214.37)(566.4409, 52.3)(567.4362, 125.42)(1105.8927, 133.87)(529.4616, 16768.23)(530.4647, 6709.01)(531.4688, 1358.55)(532.4706, 198.04)(1059.9633, 123.97)(575.4569, 53.36)	112312.7	35718.71	135940.2	38419.76	109686.9	29994.02	109543.8	33989.98	0.00	0.997917	0.999401	-0.79	0.018615	0.196411
COMPOUND_530_4708a14_106094	Rp	+	530.4708	14.11				(553.4601, 52249.25)(554.4632, 20117.25)(555.4666, 4304.29)(556.4693, 792.39)(557.4665, 122.17)(569.4338, 5472.33)(570.4386, 2016.49)(571.4471, 2231.85)(572.4512, 705.75)(548.5049, 358172.88)(549.5082, 133559.95)(550.5111, 26568.04)(551.5132, 4390.74)(552.5164, 558.86)(531.4774, 6682.04)(532.4766, 2288.03)(533.4816, 500.17)(534.4865, 96.62)	4050187	177945.9	3886086	190241.3	4176189	175651.5	4125030	162040.9	0.75	0.005055	0.011395	0.44	0.154366	0.466404

									1st trimester				3rd trimester									
									Control		Pre-eclampsia		Control		Pre-eclampsia		Time			Interaction		
Compound	Column	Ion_mode	Mass	Retention_	Identification	ID_level	MSMS_spectra	CompositeSpectrum	Mean	SD	Mean	SD	Mean	SD	Mean	SD	Value	raw p	FDR_p	Value	raw p	FDR_p
COMPOUND_531_276a12_5302	Rp	-	531.2760	12.53				(644.2597, 1590.6)(645.2632, 607.73)(646.2581, 778.39)(647.2572, 327.7)(648.2531, 346.58)(566.246, 79089.75)(567.2493, 27271.29)(568.2443, 27455.04)(569.2463, 8822.82)(570.2499, 1944.69)(571.2576, 473.6)(530.2679, 3863.73)(531.2685, 1752.99)(532.2751, 878.06)(533.2837, 62.45)(576.2746, 68865.68)(577.2776, 24515.66)(578.2808, 5642.26)(579.2833, 977.96)	724224.2	92660.43	695101.9	108358.3	778002.5	69506.33	764839.2	108356.8	0.64	0.029071	0.054877	0.12	0.730509	0.885165
COMPOUND_531_3678a10_857612	Rp	+	531.3678	10.86				(1101.726, 550.6)(1102.7317, 742.34)(1103.7375, 838.16)(1085.7325, 1092.04)(1086.723, 611.17)(532.3754, 8951.49)(533.38, 2670.45)(534.391, 3114.92)(535.3926, 1291.42)	100761.2	33005.84	113391.5	44983.04	39021.83	30850.47	33471.79	25714.59	-1.07	4.02E-07	2.25E-06	-0.51	0.028159	0.229762
COMPOUND_531_3866a11_108429	Rp	+	531.3866	11.11				(1101.765, 1010.56)(1102.7765, 1092.41)(1103.7605, 663.52)(570.3458, 322.9)(532.3928, 11985.21)(533.3955, 5496.94)(534.3908, 1173.64)(1080.7734, 1179.2)(1081.7982, 564.96)(1082.7664, 875.45)	38627.86	8678.665	34332.11	8451.365	42232.45	8301.785	47123.34	12973.78	0.32	0.221971	0.315181	0.47	0.137592	0.443286
COMPOUND_532_7128a10_43298	Rp	+	532.7128	10.43				(555.7137, 386.33)(533.723, 5623.2)(534.7215, 1191.01)(535.71, 1253.69)(536.7236, 231.52)(537.7105, 432.89)	48251	16488.73	40166.21	14683.58	52171.38	9245.288	52306.69	14145.92	0.21	0.450206	0.555846	0.71	0.036714	0.255977
COMPOUND_533_0477a10_432405	Rp	+	533.0477	10.43				(1067.1024, 312.13)(1105.0792, 222.98)(556.0474, 322.62)(557.0585, 527.92)(571.9969, 355.14)(551.0602, 353.56)(534.0558, 8786.99)(535.0603, 747.95)(536.0417, 946.25)	37122.54	14049.95	28597.03	11988.99	40646.55	7772.888	40263.04	13607.21	0.07	0.793885	0.850538	0.80	0.018961	0.196411
COMPOUND_533_283a10_251471	Rp	+	533.2830	10.25				(556.2771, 980.04)(556.7809, 697.89)(557.2848, 1244.82)(557.7897, 775.77)(558.2787, 816.86)(1067.5914, 853.35)(1105.5509, 1473.38)(556.2933, 1056.49)(557.3131, 230.98)(558.3, 474.05)(545.2847, 376.36)(545.7923, 568.71)(546.2788, 389.74)(1089.5652, 1668.9)(1090.5754, 1083.13)(1091.59, 4059.1)(1092.5939, 3088.9)(1093.582, 1854.58)(564.26, 2519.46)(564.7697, 2681.71)(565.2727, 1979.34)(565.774, 915.97)(566.2605, 330.0)(534.2953, 10181.1)(535.2989, 3315.31)(536.3059, 4632.85)(537.3075, 1971.11)(538.3051, 1823.41)(267.6488, 33273.44)(268.1505, 8966.75)(268.6516, 2135.61)(269.1552, 348.01)	448914.4	110080	408538.9	144166.9	470660.4	45479.31	464670.7	56994.4	-0.09	0.772512	0.833547	0.24	0.50909	0.757224
COMPOUND_533_2861a10_151148	Rp	+	533.2861	10.15				(556.2819, 808.22)(556.7777, 470.47)(557.2842, 866.68)(557.7805, 468.61)(558.2819, 524.08)(1067.631, 6078.28)(1105.5562, 705.0)(545.2845, 239.57)(545.7837, 257.13)(546.2768, 203.58)(556.2947, 3432.1)(564.2609, 1132.78)(564.7708, 1197.14)(565.2655, 1465.11)(565.7792, 710.74)(566.2556, 278.21)(1089.5662, 867.51)(1090.5763, 791.83)(1091.5918, 2554.17)(1092.6056, 2365.02)(1093.5991, 1480.62)(534.2954, 5880.01)(535.2988, 2358.72)(536.3073, 4472.39)(537.3092, 2280.84)(538.3094, 1284.64)(267.6493, 30446.66)(268.1509, 8256.29)(268.652, 1782.24)(269.1534, 366.8)	328545.6	51646.95	321092.1	43493.23	327030.1	116180.6	273343.9	76884.13	-0.39	0.124059	0.195708	-0.66	0.030294	0.234557
COMPOUND_533_2885a1_0548054	Hilic	+	533.2885	1.05				(1068.6038, 401.31)(1069.5807, 94.81)(556.2855, 259.38)(1089.58, 619.97)(1090.5778, 1101.91)(1091.5734, 664.55)(551.3318, 557.56)(534.2963, 10553.75)(535.299, 2822.82)(536.2979, 1280.55)(537.3005, 258.45)(1084.6652, 769.91)	68117.11	7441.158	73009.46	7263.458	63811.36	13063.97	66229.25	9185.799	-0.34	0.23858	0.333581	-0.43	0.219599	0.528917
COMPOUND_533_2896a1_1620592	Hilic	+	533.2896	1.16				(1067.5598, 361.24)(1068.578, 812.73)(1069.6039, 818.3)(556.3005, 667.62)(557.3017, 314.43)(1089.5681, 270.31)(1090.5751, 517.35)(1091.5905, 566.98)(551.328, 398.97)(534.2962, 10210.61)(535.2997, 3015.3)(536.2991, 1267.81)(537.2996, 259.32)(1084.6467, 701.68)	109809.2	20002.55	115580.4	27703.46	109156.4	7665.042	110242.1	8653.991	-0.10	0.733531	0.806204	-0.39	0.264411	0.575338
COMPOUND_533_3075a10_783806	Rp	-	533.3075	10.78				(532.3006, 7855.75)(533.304, 2044.12)(534.3057, 568.49)	19922.32	5427.853	21319	6831.156	13177	1241.68	12618.25	2472.042	-1.33	2.42E-10	3.34E-09	-0.02	0.934202	0.971294
COMPOUND_533_3098a1_3927718	Hilic	+	533.3098	1.39				(1067.5927, 262.98)(556.3021, 5993.7)(557.3051, 1844.89)(558.3181, 5137.98)(534.3183, 35084.64)(535.3216, 10380.87)(536.3338, 13117.82)(537.338, 3905.48)(538.3495, 2664.5)(539.3531, 724.11)(540.3645, 1294.49)(267.665, 925.84)(268.1676, 438.82)(268.6722, 23.65)	169809	157109.7	207830.8	110886.9	86359	#DIV/0!	74209.6	51413.28	-0.92	1.92E-05	7.21E-05	-0.22	0.359218	0.666906
COMPOUND_533_3868a10_702347	Rp	+	533.3868	10.70				(1105.7347, 220.36)(1106.7107, 404.46)(1107.7002, 350.07)(556.3879, 2692.31)(557.3938, 1852.38)(558.3996, 13730.82)(1089.7137, 457.98)(1090.745, 231.97)(534.3888, 7970.38)(535.3889, 3142.97)(536.3874, 5948.64)(537.3904, 2578.79)(1084.7565, 225.12)(1085.7496, 141.69)(1086.7336, 137.34)	46590.69	16020.33	53406.14	26128.5	36442	19555.71	37439.43	17847.1	-0.97	1.65E-05	6.31E-05	-0.15	0.546474	0.77815
COMPOUND_533_7078a10_0650425	Rp	+	533.7078	10.07				(556.6939, 595.85)(557.7019, 332.73)(534.7151, 16560.88)(535.7179, 1210.28)(536.7003, 1120.38)	57692.73	24846.98	47374.4	20902.76	60973.08	11314.49	63254.84	18874.32	0.19	0.499444	0.600186	0.74	0.030401	0.234557
COMPOUND_535_1591a11_6996355	Rp	+	535.1591	11.70				(1071.3276, 707.7)(1072.3239, 830.4)(1073.333, 857.57)(1074.335, 747.82)(1109.2837, 715.67)(1093.3014, 380.17)(1094.3213, 594.73)(536.1659, 38785.71)(537.1663, 19286.2)(538.165, 13224.04)(1088.3312, 958.59)	184821.8	17461.59	180130.6	16549.95	190287.2	21340.35	199013.4	18743.03	0.30	0.280115	0.379326	0.67	0.048832	0.279822
COMPOUND_535_3277a1_4799759	Hilic	+	535.3277	1.48				(558.3179, 1620.36)(559.3215, 498.48)(560.3337, 1058.06)(561.3368, 317.34)(536.3345, 4131.89)(537.3382, 1181.47)(538.3496, 1336.83)(539.354, 376.89)(540.3661, 2267.01)	107331.5	90594.99	136777.9	71630.77	86559.75	30415.68	139560.9	113399.2	-0.96	2.79E-05	0.0001	-0.38	0.139807	0.444883
COMPOUND_535_3278a1_5378143	Hilic	+	535.3278	1.54				(558.3173, 6325.06)(559.3208, 2104.91)(560.3327, 2135.83)(561.3371, 775.75)(536.3349, 7399.81)(537.3382, 2351.96)(538.3502, 2421.67)(539.3528, 669.56)(540.3659, 2204.62)	134400.2	91688.88	162963	94592.11	126829	60579.1	149219.6	72496.95	-0.96	2.39E-05	8.71E-05	-0.41	0.105918	0.387484
COMPOUND_535_3285a8_948308	Rp	+	535.3285	8.95	LPC HODE	2	20 eV: 518.3264(100), 104.1083(80), 184.0737(50), 454.9768(25)	(558.3206, 2564.3)(559.3223, 835.24)(560.3347, 2707.03)(561.3395, 912.58)(562.333, 232.0)(536.3363, 94582.22)(537.3397, 26135.66)(538.3434, 5067.09)(539.3468, 919.97)	129437.3	107060.1	179966.3	115023.7	119562.8	66490.47	150788.6	65783.44	-0.88	0.000105	0.000323	-0.41	0.113829	0.400254
COMPOUND_535_3644a1_0816109	Hilic	+	535.3644	1.08				(1071.7275, 21057.24)(558.3532, 865.02)(559.355, 254.61)(560.3687, 696.2)(561.3701, 336.62)(1093.715, 15727.69)(1094.7205, 7239.11)(1095.7251, 3383.72)(536.3889, 21054.88)(537.3754, 2240.26)(538.3937, 20329.21)(539.3919, 5336.81)(540.3934, 1074.85)(541.3881, 233.67)	234872.1	107384.5	264537.9	88936.16	92873.9	22978.84	100195.6	27504.88	-0.18	0.510219	0.610853	0.08	0.796743	0.91372
COMPOUND_535_3985a10_906938	Rp	+	535.3985	10.91				(558.3984, 11521.09)(559.3938, 3159.03)(560.3996, 11576.8)(536.4092, 24585.94)(537.4121, 7481.89)(538.4086, 2150.19)(539.4081, 312.56)	213778.9	68476.05	206651.9	70813.62	210333.7	12352.43	152105.7	60443.47	-1.06	2.84E-06	1.27E-05	-0.10	0.686826	0.854077
COMPOUND_535_3998a1_0951511	Hilic	+	535.3998	1.10				(558.3865, 790.76)(559.3834, 511.63)(560.3979, 2195.96)(561.4055, 687.42)(553.4389, 80.23)(536.4069, 33507.43)(537.4096, 10497.12)(538.3886, 13553.93)(539.3909, 3903.35)(540.3948, 573.29)	181254.5	44254.34	194726.8	67488.94	88308.33	54673.3	72179.6	39658.27	-1.01	2.04E-05	7.57E-05	-0.04	0.88131	0.950612
COMPOUND_537_3785a1_0742693	Hilic	+	537.3785	1.07				(1075.7529, 1189.84)(1076.7651, 491.96)(1077.7747, 214.0)(560.3934, 2563.72)(561.4015, 745.76)(1097.7543, 498.2)(1098.7369, 591.69)(1099.7625, 280.01)(555.3981, 1396.82)(538.3882, 24422.51)(539.392, 7503.54)(540.3929, 1701.32)(541.3937, 201.6)	106435.8	23629.46	105242.7	23324.72	36061.86	10076.87	34661.08	10202.39	-1.27	9.28E-08	5.87E-07	-0.02	0.949651	0.978323
COMPOUND_537_38a10_808536	Rp	+	537.3800	10.81				(1113.761, 1399.67)(560.3863, 1091.72)(561.3985, 1761.75)(576.3407, 364.08)(538.3875, 16758.73)(539.39, 5694.0)(540.3925, 2250.82)	130821.9	35868.69	128713.6	43546.7	52017.25	15474.62	56428.31	21151.19	-1.38	5.62E-11	9.89E-10	-0.06	0.788656	0.909997
COMPOUND_537_4183a11_136549	Rp	+	537.4183	11.14				(560.4025, 5471.15)(561.4008, 1845.61)(562.4027, 4683.02)(538.4231, 4576.37)(539.4255, 1477.99)(540.4049, 642.62)	91478.22	36714.09	72972.5	28080.45	85426.17	39376.51	86135.73	51766.68	-0.72	0.004334	0.00991	0.41	0.160749	0.473212
COMPOUND_538_0445a10_193908	Rp	+	538.0445	10.19				(1077.123, 951.1)(561.0248, 421.5)(577.004, 441.23)(577.999, 77.59)(539.0547, 25243.05)(540.0569, 4333.35)(541.0391, 4347.3)(542.0274, 505.49)(543.0509, 1538.26)	77936.08	28082.25	66561.79	27039.8	79568.69	19550.75	81918.2	23608.56	0.03	0.906687	0.938328	0.69	0.048821	0.279822
COMPOUND_538_3792a10_193529	Rp	+	538.3792	10.19				(561.3776, 1245.85)(1099.7418, 192.01)(1100.742, 213.24)(539.3892, 23475.66)(540.3819, 2651.26)(541.3731, 2673.97)	54706.68	21185.71	45015.95	20827.7	59762.42	10354.12	61831.37	19109.21	0.11	0.704685	0.784067	0.78	0.022385	0.213113
COMPOUND_538_5459a10_468469	Rp	+	538.5459	10.47				(561.5336, 3351.38)(562.5363, 1256.37)(563.5387, 372.94)(556.5584, 933.02)(539.5525, 12025.39)(540.5548, 5034.12)(541.5594, 980.39)(542.5617, 214.78)	46972	5672.036	45073.5	6069.867	52852.14	4455.899	50341.74	3695.813	1.13	8.1E-05	0.000254	-0.20	0.536595	0.776357

									1st trimester				3rd trimester									
									Control		Pre-eclampsia		Control		Pre-eclampsia		Time			Interaction		
Compound	Column	Ion_mode	Mass	Retention_	Identification	ID_level	MSMS_spectra	CompositeSpectrum	Mean	SD	Mean	SD	Mean	SD	Mean	SD	Value	raw p	FDR_p	Value	raw p	FDR_p
COMPOUND_539_3219a9_802215	Rp	-	539.3219	9.80				(538.3141, 38160.7)(539.3175, 10844.56)(540.324, 3184.95)(541.3287, 603.44)(542.3296, 91.19)	136218.3	50037.57	159695.5	66339.68	118562.9	27439.56	115547.3	53758.74	-0.34	0.190162	0.279169	-0.47	0.116794	0.405455
COMPOUND_539_322a9_936208	Rp	-	539.3220	9.94	LPC 16:1	2	10 eV: 478.2928(100), 538.3157(52), 253.218(10), 44.9987(6)	(538.3145, 330043.12)(539.3179, 88133.05)(540.3217, 18930.29)(541.3251, 3317.2)(542.329, 522.27)	868583	319518.9	1035936	397725.8	803952	172416.7	767833.7	345989.5	-0.26	0.325103	0.425658	-0.60	0.051887	0.285695
COMPOUND_539_3593a1_494732	Hilic	+	539.3593	1.49				(562.3503, 6874.04)(563.3534, 2141.53)(564.3643, 1932.9)(540.3659, 5378.41)(541.369, 1677.98)(542.3728, 312.7)	71896.9	36422.4	90615.61	47470.53	16925.7	8012.141	24031.5	30515.86	-1.31	3.09E-07	1.76E-06	0.06	0.815253	0.92157
COMPOUND_540_256a6_879685	Rp	-	540.2560	6.88				(539.2493, 10108.05)(540.2525, 2969.57)(541.2554, 882.81)(542.2555, 186.6)	29839.81	11851.31	30162.87	10416.43	25091.38	10326.32	30725.3	14197.52	-0.38	0.172544	0.257161	-0.02	0.960956	0.981885
COMPOUND_540_2677a2_2955306	Hilic	+	540.2677	2.30				(541.275, 6258.53)(542.2787, 1924.48)(543.2811, 522.34)(544.2778, 98.43)	9407.14	3470.968	11382.98	4895.084	35220.07	15204.54	33244.79	14158.79	1.59	4.39E-12	1.3E-10	-0.26	0.22642	0.533925
COMPOUND_541_3016a10_103418	Rp	+	541.3016	10.10				(1083.6344, 1810.76)(1084.6335, 952.29)(1085.653, 7927.2)(1086.6566, 5935.23)(1087.6711, 40142.84)(1121.5801, 1215.24)(1122.5913, 4463.17)(561.3006, 2480.0)(561.794, 1761.26)(564.3138, 5473.55)(565.2925, 3844.51)(553.3118, 4056.18)(553.7967, 2666.21)(554.3076, 4494.62)(554.7994, 1228.04)(555.2976, 5617.42)(1105.5735, 2898.7)(1106.5789, 6296.32)(301.6359, 1855.91)(302.1422, 907.87)(580.2907, 6103.56)(580.783, 551.2)(581.2793, 17502.36)(293.6323, 627.56)(294.1325, 289.08)(580.27, 572.67)(581.2791, 17069.09)(582.2821, 6483.93)(1100.5967, 1224.6)(271.6609, 4400.96)(272.1621, 1741.13)(272.662, 1015.86)(273.1625, 115.33)	498225.4	84396.66	477857	81099.39	296108.4	51233.26	306294.4	83698.19	-1.46	1.1E-09	1.21E-08	0.33	0.166758	0.480028
COMPOUND_541_3152a1_0322595	Hilic	+	541.3152	1.03				(1083.6595, 928.46)(1084.6608, 705.97)(1085.6573, 3777.5)(564.3134, 2383.54)(565.3176, 823.86)(559.3576, 685.18)(542.3245, 68152.59)(543.3275, 19867.57)(1100.6364, 900.17)	142757.1	63438.92	145064.5	45419.99	78146.29	28554.89	86414.88	38400.61	-1.30	8.38E-07	4.24E-06	0.04	0.890222	0.953702
COMPOUND_541_317a1_1332275	Hilic	+	541.3170	1.13				(564.3078, 5200.53)(565.3111, 2139.39)(542.3245, 494054.5)(543.3281, 142677.77)	631180.3	360977.3	586225.3	273697.1	364981.4	128797.5	364642.8	145492.8	-1.07	3.25E-05	0.000114	0.08	0.772758	0.904496
COMPOUND_541_3178a9_769402	Rp	+	541.3178	9.77				(1083.6155, 80.88)(564.3072, 16774.8)(565.3114, 5570.23)(566.3185, 1881.97)(580.2694, 1035.79)(581.2639, 308.13)(542.3265, 142151.4)(543.3297, 43445.36)(544.3321, 8017.93)(545.3367, 1526.44)(1100.6532, 153.11)	132335.1	179790.1	204038	293765.2	81776.21	65844.7	74379.32	59224.15	-0.47	0.099594	0.162099	-0.33	0.329503	0.635357
COMPOUND_541_3185a9_84722	Rp	+	541.3185	9.85	LPC 20:5	2	10 eV: 542.3259(100), 104.1087(20), 184.0754(13)	(1083.6375, 3405.19)(1084.6405, 2352.62)(1085.6447, 796.28)(564.3077, 21828.45)(565.3102, 6706.04)(566.3181, 2219.01)(567.3058, 309.83)(1105.6172, 814.57)(1106.6276, 452.19)(1107.6013, 951.79)(1108.6063, 636.64)(1109.612, 266.13)(580.2785, 3157.04)(581.2768, 981.81)(542.3258, 521416.88)(543.3295, 153956.36)(544.3322, 28875.86)(545.3334, 4585.91)(546.3338, 744.97)(1100.6376, 68.63)(271.6583, 499.65)(272.1619, 251.58)(272.6621, 61.87)	693054.5	633877.2	587868.6	418911.4	210467.7	130129.8	231554.2	142991.9	-1.32	5.83E-07	3.08E-06	0.28	0.318505	0.624803
COMPOUND_541_3367a10_146301	Rp	-	541.3367	10.15				(540.3295, 914958.6)(541.3335, 237716.08)(542.3357, 48358.0)(543.3383, 7105.4)(544.3362, 858.57)	4953321	637830.4	5179236	669306.3	3213431	642638.7	3329479	644866.2	-1.50	4.14E-11	7.76E-10	-0.16	0.46607	0.732953
COMPOUND_541_3369a10_256066	Rp	-	541.3369	10.26	LPC 16:0	1	10 eV: 480.3086(100), 540.33(57), 255.2325(13), 44.9983(2)	(1127.6696, 646.81)(1128.6735, 323.22)(1129.688, 1827.46)(540.3294, 1034370.9)(541.3335, 269025.56)(542.3356, 50257.91)(543.3384, 7916.24)(544.3345, 1248.39)	16183019	1179755	16880435	1150385	13000000	1421808	13249285	1601320	-1.46	1.56E-10	2.25E-09	-0.24	0.288347	0.597533
COMPOUND_541_7246a2_894622	Hilic	+	541.7246	2.89				(1084.4597, 460.7)(1085.4883, 494.07)(1086.4655, 374.04)(1106.4604, 430.78)(542.731, 8225.28)(543.7344, 618.87)(544.7296, 4499.21)(545.7334, 415.13)(546.7282, 1116.6)(547.7338, 88.51)(548.7253, 206.47)	34507.62	8580.399	40749.46	10080.9	36474.93	9596.589	35890.47	10680.47	0.31	0.279079	0.378834	-0.61	0.080349	0.339484
COMPOUND_542_7169a10_312872	Rp	+	542.7169	10.31				(560.7331, 854.52)(543.7253, 14219.59)(544.7301, 1637.55)(545.7137, 1741.76)(546.7081, 381.8)(547.7216, 698.76)	58399.06	24805.94	48763.65	20261.59	65140.77	13874.72	66969.18	20758.59	0.29	0.312123	0.413471	0.58	0.089411	0.353871
COMPOUND_543_0519a10_314047	Rp	+	543.0519	10.31				(1087.0785, 546.38)(1125.0988, 316.28)(566.0373, 806.48)(567.0355, 213.27)(582.0081, 639.2)(583.0057, 275.81)(544.059, 16073.09)(545.0622, 1116.9)(546.0452, 965.52)	47613.24	17640.74	41191.46	17446.01	47726.23	10380.72	51483.8	13188.08	0.04	0.885691	0.923801	0.78	0.025025	0.219303
COMPOUND_543_1841a10_601183	Rp	-	543.1841	10.60				(656.1604, 412.7)(657.1601, 233.47)(658.1655, 692.68)(578.1763, 1033.81)(579.1812, 607.09)(1131.4148, 328.09)(1132.4268, 461.66)(1133.4387, 418.83)(542.1788, 6008.06)(543.1808, 1497.62)(544.1692, 949.1)(545.1608, 212.51)(1199.3701, 314.77)(1200.3796, 254.17)(1201.3562, 302.77)	16952.12	4428.088	16622.06	5024.311	15753.64	4857.836	18320.8	5530.848	-0.13	0.626339	0.717454	0.31	0.3162	0.622922
COMPOUND_543_3191a10_370974	Rp	+	543.3191	10.37	PC 20:4e	2	20 eV: 104.1066(100), 485.2637(65), 544.3366(60)	(1087.6656, 1294.14)(1088.6696, 758.48)(1089.6799, 903.59)(1090.685, 516.08)(1091.6704, 702.29)(1125.6204, 783.61)(1126.6263, 4467.06)(1127.63, 3103.91)(566.3145, 2111.11)(567.311, 1315.75)(563.317, 2770.62)(563.8143, 653.44)(564.308, 1301.58)(564.7955, 601.88)(555.3135, 1222.97)(555.796, 975.21)(556.3273, 514.02)(582.2805, 3449.97)(582.8013, 540.43)(583.2826, 1371.65)(583.8001, 365.22)(584.2741, 2545.3)(584.7927, 646.81)(585.28, 715.65)(582.2787, 2787.34)(583.268, 555.19)(584.2731, 2126.8)(544.3387, 147325.17)(545.3418, 40950.2)(1104.7257, 613.45)(1105.7112, 649.93)(272.668, 5209.58)(273.1694, 2501.88)(273.6689, 1010.88)(274.1598, 96.73)	1329145	229745.6	1379280	171195.3	885622.7	168733.6	953983.3	187090.9	-1.59	5.79E-11	1E-09	0.12	0.617455	0.816971
COMPOUND_543_3324a1_0920596	Hilic	+	543.3324	1.09				(1087.672, 10471.39)(1088.6752, 6151.93)(1089.6871, 15729.22)(1090.6903, 9422.43)(1091.7034, 33969.23)(1092.7073, 20287.34)(1093.7151, 14155.35)(1094.7205, 6512.12)(566.3223, 25175.99)(567.3255, 7845.21)(1109.6459, 1935.17)(1110.6527, 1208.49)(1111.6719, 6777.15)(1112.675, 4398.97)(1113.6855, 5414.35)(561.3941, 759.68)(544.3394, 803566.7)(545.3437, 228707.16)(546.3545, 462580.06)(547.3586, 133273.9)(548.3697, 87038.22)(549.3738, 23635.73)(1104.6647, 416.75)	6992772	1488215	6785943	1477341	3919556	774607.3	4480450	1359311	-1.59	3.92E-11	7.62E-10	0.34	0.142326	0.448182
COMPOUND_543_3327a0_99507535	Hilic	+	543.3327	1.00				(1087.672, 3180.81)(1088.6743, 1871.72)(1089.6858, 4635.83)(1090.6917, 2919.04)(1091.7028, 4816.06)(1092.706, 2965.21)(1093.714, 2377.16)(1094.7189, 1187.12)(566.3218, 8903.6)(567.3259, 2870.06)(1109.6416, 617.31)(1110.6499, 477.34)(1111.6685, 1067.62)(1112.6691, 557.78)(1113.6819, 943.37)(1114.685, 616.33)(1115.6967, 514.85)(561.3838, 239.85)(544.3403, 159135.47)(545.3436, 48940.28)(546.3555, 132346.0)(547.3591, 40910.89)(548.3678, 14755.0)(549.3728, 3655.67)	851273.5	201002.7	864881.9	201211.3	393075.8	97725.47	441791.8	153150	-1.58	2.72E-12	8.87E-11	0.05	0.805392	0.917422
COMPOUND_543_3348a10_016662	Rp	+	543.3348	10.02				(1087.674, 108430.22)(1088.6771, 65895.26)(1089.679, 23652.31)(1125.6036, 1109.48)(566.3225, 36627.7)(567.3254, 12078.11)(1109.6498, 1210.16)(1110.649, 860.85)(1111.6508, 1302.58)(1112.6523, 656.53)(1113.6635, 336.76)(1114.6765, 130.79)(582.2866, 5677.43)(544.3418, 143136.66)(545.3444, 43324.78)(546.3521, 7982.11)(547.3516, 1676.1)	1435653	1711625	1190357	1303771	336050.6	559752.5	542827.6	633800.1	-1.70	4.33E-09	3.87E-08	0.79	0.009498	0.153413
COMPOUND_543_335a10_098435	Rp	+	543.3350	10.10	LPC 20:4	1	10eV: 544.3387(100), 184.0743(70), 125.0033(7), 401.6277(5)	(1087.674, 108430.22)(1088.6771, 65895.26)(1089.679, 23652.31)(1125.596, 1704.02)(566.3223, 43215.77)(567.3254, 14118.73)(1109.6532, 4759.12)(1110.6577, 3144.98)(1111.6671, 9428.41)(1112.6719, 5959.38)(1113.6649, 2491.98)(1114.6708, 352.15)(582.2872, 7219.69)(544.3405, 933251.75)(545.3448, 276707.78)(546.3475, 52688.17)(547.345, 8662.69)	4201431	986833.3	3712442	1040892	1858705	442345.8	2248575	901949.6	-1.67	1.6E-12	6.22E-11	0.53	0.017027	0.193655
COMPOUND_543_4428a11_848227	Rp	+	543.4428	11.85				(1087.9457, 1217.28)(566.4325, 1935.87)(561.4829, 1697.89)(562.4537, 2032.36)(544.4584, 10146.73)(545.4622, 3365.46)(546.4613, 895.0)	29703.76	11735.71	25364.59	11065.81	28766.2	8184.698	28743.31	13029.04	0.38	0.137576	0.212935	-0.34	0.266481	0.575669
COMPOUND_544_8708a10_071682	Rp	+	544.8708	10.07				(545.8778, 5441.12)(546.8804, 1297.25)(547.8842, 886.92)	30396.12	12075.41	29744.46	10099.36	46677.31	8770.512	42893.2	10654.12	0.98	0.000345	0.000984	0.05	0.867981	0.9447
COMPOUND_545_2716a10_12051	Rp	-	545.2716	10.12				(658.2552, 783.15)(659.2512, 339.2)(1135.5144, 246.02)(1136.511, 235.29)(544.2643, 5358.88)(545.2688, 1528.09)(546.2681, 333.66)(547.2661, 64.68)	20895	6477.642	22871.07	8363.635	29557.5	7915.47	28607.04	7808.143	1.03	0.000357	0.001016	-0.41	0.208953	0.518913
COMPOUND_545_3231a10_471181	Rp	-	545.3231	10.47				(1125.5765, 450.2)(1126.5818, 246.19)(658.3335, 1530.82)(659.3311, 442.71)(1135.615, 964.81)(1136.6185, 668.06)(1137.6165, 533.68)(544.3165, 6241.08)(545.3195, 2054.89)(546.3127, 4266.5)(547.3153, 1341.16)(548.2948, 382.15)(1089.6095, 303.13)(1090.6163, 246.01)(1091.6348, 142.23)(1203.6182, 559.4)(1204.6171, 308.83)(1205.5865, 291.65)	77264.57	36087.78	85689.5	39580.59	65503.3	20116.36	63644.79	20054.7	-0.34	0.203829	0.294812	0.10	0.755579	0.895058

									1st trimester				3rd trimester									
									Control		Pre-eclampsia		Control		Pre-eclampsia		Time			Interaction		
Compound	Column	Ion_mode	Mass	Retention_	Identification	ID_level	MSMS_spectra	CompositeSpectrum	Mean	SD	Mean	SD	Mean	SD	Mean	SD	Value	raw p	FDR_p	Value	raw p	FDR_p
COMPOUND_545_3504a10_286545	Rp	+	545.3504	10.29	LPC 20:3	2	10 eV: 546.3574(100), 104.1073(18), 184.0727(5)	(1091.7001, 5370.31)(1092.7057, 3017.96)(1093.7015, 1449.51)(1129.6799, 565.92)(568.3391, 13369.4)(569.3415, 4492.85)(1113.6831, 352.69)(1114.6952, 316.42)(1115.7036, 539.7)(1116.7185, 401.08)(1117.6951, 683.56)(584.3076, 561.8)(585.3135, 327.66)(546.3573, 295396.2)(547.3605, 88926.8)(548.3624, 17529.76)(549.365, 3292.65)	1056674	307851.1	1044963	354638	702017.1	198093.3	812065.1	265324.8	-1.21	7.98E-06	3.24E-05	0.44	0.134394	0.43508
COMPOUND_545_3508a10_194364	Rp	+	545.3508	10.19	PC 20:3e	2	10 eV: 184.0748(100), 546.3461(7), 104.1077(5), 377.6575(2)	(1091.7, 2170.25)(1092.705, 1111.37)(1093.7074, 775.63)(1129.6486, 413.17)(568.3401, 15741.98)(569.3442, 5182.36)(1113.6522, 514.5)(1114.6628, 362.14)(1115.6458, 84.57)(584.3235, 18137.4)(585.3252, 10963.33)(546.358, 166193.06)(547.3611, 49286.48)(548.3625, 9944.77)(549.3674, 1525.89)	416535	298481.3	438421.2	326751.3	135788.4	33872.28	217779.1	161254.5	-1.57	3.19E-08	2.3E-07	0.59	0.048722	0.279822
COMPOUND_545_7903a10_102914	Rp	+	545.7903	10.10				(1092.5839, 10957.29)(1093.5883, 6796.05)(1094.5886, 2712.39)(1095.5857, 1172.53)(1096.5983, 161.35)(1114.61, 3686.47)(1115.605, 2723.14)(1116.5797, 9773.59)(1117.5814, 6780.56)(1118.5741, 3795.75)(1119.5743, 2146.22)(584.7374, 3247.55)(585.7367, 868.13)(546.7978, 18383.51)	307895.9	47665.52	289156.2	61179.45	243915.4	52371.76	229813.3	66633.66	-0.80	0.002071	0.005143	0.15	0.606288	0.810674
COMPOUND_546_3793a13_977488	Rp	+	546.3793	13.98				(569.368, 12504.14)(570.3706, 4499.62)(571.3676, 1995.28)(572.3717, 471.19)(585.3478, 1364.2)(586.3509, 584.97)(587.3389, 306.61)(588.3494, 72.72)(564.4122, 19720.77)(565.4157, 6605.36)(566.4136, 3083.17)(567.4146, 691.95)(547.3857, 25014.49)(548.3891, 8766.8)(549.3859, 3827.21)(550.3888, 892.07)	449194.8	89279.71	421242.1	89806.53	442405.9	57222.84	433870.8	68173.65	-0.03	0.921569	0.949393	0.23	0.527756	0.7696
COMPOUND_547_2377a9_927201	Rp	+	547.2377	9.93				(567.2387, 422.6)(567.7361, 484.95)(548.2444, 5925.62)(549.2491, 1929.8)(550.2521, 2200.28)(551.2555, 1035.48)(552.2606, 1032.46)(1112.5095, 196.02)(1113.5176, 143.42)(1114.4904, 224.71)(274.6268, 9306.99)(275.1285, 3139.8)(275.6278, 4692.02)(276.1289, 1383.37)(276.6254, 1746.38)(277.1279, 561.89)(277.6246, 353.06)	64788.68	22870.39	71482	27674.53	61551.29	13159.53	58068.55	26664.37	-0.21	0.424307	0.528799	-0.43	0.152001	0.463831
COMPOUND_547_2878a10_380387	Rp	-	547.2878	10.38				(1139.5836, 483.08)(1140.5781, 494.53)(1141.6106, 1817.88)(546.2807, 8198.28)(547.2833, 2433.91)(548.2876, 622.44)(549.281, 91.01)(1093.5437, 177.9)(1094.5565, 257.44)(1095.5536, 182.4)(592.2729, 722.65)(593.266, 468.62)(594.2574, 642.38)	33186.23	11968.39	36130.16	8732.515	44086.25	10373.67	46062.69	11787.18	0.89	0.00075	0.002008	-0.25	0.409908	0.694731
COMPOUND_547_366a10_495685	Rp	+	547.3660	10.50	LPC 20:2	2	10 eV: 548.3727(100), 184.0712(25),104.104(22), 45.0326(5)	(1095.6895, 119.35)(1096.6508, 726.56)(570.3547, 12781.87)(571.3596, 4405.39)(572.3702, 2515.52)(586.3165, 825.1)(587.3187, 577.84)(588.3338, 683.63)(548.3725, 84888.15)(549.3756, 25983.9)(550.3788, 5985.82)(551.3821, 1273.68)	443262	103551	480971.2	120250.1	178390.9	74470.16	196235.2	83906.63	-1.50	9.38E-11	1.43E-09	-0.04	0.864121	0.943353
COMPOUND_547_8064a10_366444	Rp	+	547.8064	10.37				(1096.6161, 57683.57)(1097.6199, 35738.68)(1098.6211, 13303.88)(1099.6261, 4937.72)(570.8011, 1678.22)(571.7954, 978.58)(1118.6067, 1356.9)(1119.6061, 1318.75)(1120.6011, 4740.15)(1121.6029, 3195.91)(1122.6042, 2613.25)(586.7563, 2417.0)(587.7557, 1737.16)(588.7427, 94.06)(548.8139, 28577.4)(549.8183, 19806.08)(550.8188, 9665.22)(1113.6589, 1196.15)	502801.7	86966.69	462558.3	109496.4	336365.8	71385.52	365007.9	101567.5	-1.50	3E-08	2.18E-07	0.65	0.02112	0.204127
COMPOUND_548_0503a10_059913	Rp	+	548.0503	10.06				(571.0446, 776.03)(549.0577, 10507.56)(550.0612, 1678.43)(551.0431, 1897.11)(552.0391, 419.09)(553.053, 682.34)	70104.12	30855.64	58761.74	31525.11	73942.77	15888.92	76314.36	23157.44	0.14	0.614554	0.707554	0.71	0.038558	0.259981
COMPOUND_548_4874a13_465339	Rp	+	548.4874	13.47				(1097.9579, 120.15)(1135.9542, 113.07)(1119.9587, 650.89)(1120.9534, 501.17)(1121.9526, 227.24)(549.4895, 80047.7)(550.4926, 29111.95)(551.4959, 6148.93)(552.4981, 1062.83)(553.4953, 107.39)	165404.9	67572.39	167164	89711.45	169416.6	43101.95	288688	149622.8	0.23	0.304081	0.404245	0.85	0.001612	0.06002
COMPOUND_549_2385a9_240673	Rp	-	549.2385	9.24				(662.2161, 519.64)(663.2094, 280.16)(664.2159, 645.66)(548.2312, 54012.44)(549.2335, 20564.45)(550.2349, 7045.64)(551.2353, 1455.34)(594.2094, 1431.08)(595.2053, 1271.94)(596.2066, 315.24)	31826.1	20454.58	30362.28	15241.89	37736	20161.49	58647.9	67542.66	0.11	0.642247	0.731954	0.07	0.796912	0.91372
COMPOUND_549_2553a10_246654	Rp	+	549.2553	10.25				(561.2643, 422.8)(561.7676, 275.57)(562.2497, 335.14)(569.2517, 868.88)(569.7466, 880.57)(558.7994, 719.15)(559.2856, 614.84)(577.7422, 3319.05)(578.2629, 1799.17)(578.7432, 3712.09)(550.2638, 2485.27)(551.2653, 1629.65)(552.2738, 2394.23)(553.2724, 1204.17)(554.2801, 1119.74)(275.6352, 18419.43)(276.1367, 5237.13)(276.6351, 7781.75)(277.138, 2043.11)(277.633, 2955.31)(278.1356, 956.55)(278.6335, 618.89)(1116.5464, 1629.49)(1117.555, 1117.21)(1118.5479, 1245.45)(1119.5496, 848.75)(1120.554, 560.64)	233916.7	50166.86	221750.8	51034.81	247141.5	28851.08	251896.7	31562.32	0.05	0.848835	0.894514	0.82	0.017386	0.194676
COMPOUND_549_2573a10_226089	Rp	+	549.2573	10.23				(569.2562, 1200.38)(569.7518, 1528.9)(561.2618, 391.76)(561.7538, 369.11)(562.2754, 1095.51)(550.2656, 1451.12)(550.7679, 522.51)(551.2671, 1378.6)(551.7714, 1297.7)(552.2729, 1498.55)(558.7881, 497.21)(559.2962, 320.7)(577.7498, 3879.1)(578.2667, 3657.53)(578.7746, 675.69)(550.2635, 1954.78)(551.2627, 1086.94)(552.2727, 2204.44)(553.2724, 1338.5)(554.278, 1291.0)(1116.5585, 4608.09)(1117.557, 2860.89)(1118.5469, 3410.15)(1119.5455, 1874.63)(1120.5557, 1170.56)(275.6348, 17454.36)(276.1367, 4147.8)(276.6351, 8432.15)(277.1371, 2054.79)(277.6326, 3233.45)(278.1354, 1025.9)(278.6336, 769.39)	215604.4	97776.14	202113.9	86346.34	233791.4	62718.58	242063.5	52171.19	0.26	0.369224	0.471635	0.29	0.395985	0.690408
COMPOUND_549_303a10_64223	Rp	-	549.3030	10.64				(1143.6008, 529.88)(1144.613, 335.86)(548.2956, 7839.8)(549.2989, 2278.49)(550.3007, 463.6)(551.2968, 85.7)	28678.64	5892.551	30696.78	6939.001	33548.13	5987.447	35515.67	7843.404	-0.12	0.655252	0.743389	0.66	0.051404	0.283822
COMPOUND_549_3795a1_0592326	Hilic	+	549.3795	1.06				(1099.7656, 504.01)(1100.771, 371.97)(1101.7822, 429.1)(572.3707, 29765.92)(573.3743, 9199.81)(574.3819, 3057.73)(575.3866, 730.79)(1121.7439, 599.65)(1122.7491, 214.84)(567.3855, 88.33)(550.3868, 56289.02)(551.3904, 16703.76)(552.4016, 17178.53)(553.4054, 4795.68)(554.3984, 3270.3)(555.3999, 1028.72)	611929.6	136082.8	618746	110767.8	266992	81533.35	272070.9	78567	-1.64	3.22E-12	1.02E-10	0.07	0.763623	0.899577
COMPOUND_549_3798a0_9942051	Hilic	+	549.3798	0.99				(1099.761, 373.8)(1100.7563, 506.04)(1101.7896, 305.7)(572.37, 18412.84)(573.3744, 5781.86)(574.3815, 2989.02)(575.3842, 674.85)(1121.7598, 389.9)(1122.7426, 396.68)(567.431, 1446.7)(550.3872, 43699.61)(551.3906, 12972.23)(552.402, 15611.9)(553.4059, 4381.49)(554.3995, 1947.58)(555.4002, 567.22)	220527.8	252940.3	295038	262830.8	104596.1	127634.8	39685.49	65831.08	-0.95	2.84E-05	0.000102	-0.63	0.013843	0.179521
COMPOUND_549_3805a10_716592	Rp	+	549.3805	10.72	LPC 20:1	2	10 eV: 550.3875(100), 197.135(8), 454.7543(2.5)	(1099.7585, 583.44)(1100.7618, 361.25)(1137.7178, 584.2)(1138.701, 628.79)(1139.6954, 710.77)(572.3724, 8716.54)(573.3767, 2778.02)(574.3868, 2791.67)(575.3973, 1487.38)(588.3455, 1077.94)(589.3448, 499.72)(590.3412, 444.37)(550.3884, 79846.8)(551.391, 26024.06)(552.3983, 9531.82)(553.4017, 2192.81)	558260.8	176577.6	546995.1	157955.8	188290.9	105979.9	180393.5	82448.09	-1.66	9.3E-12	2.35E-10	0.17	0.457932	0.727948
COMPOUND_549_8195a10_640041	Rp	+	549.8195	10.64				(1100.648, 77144.65)(1101.6515, 46453.4)(1102.6526, 17205.19)(1103.6558, 5957.56)(1104.663, 2092.35)(572.8164, 1181.56)(573.8199, 689.72)(574.8074, 465.75)(1122.6417, 691.92)(1123.6497, 1114.39)(1124.66, 1187.96)(1125.6483, 1160.95)(1126.65, 1229.17)(567.8798, 587.77)(588.8003, 1446.95)(589.7938, 813.9)(550.8301, 32312.7)(551.8338, 21981.25)(552.8345, 12124.93)(1117.6656, 467.56)(1118.6702, 516.29)(1119.673, 375.78)(1120.6436, 786.54)	438994.3	84652.56	402532.3	92876	324265.6	75110.82	314126.6	99682.18	-1.07	5.26E-05	0.000174	0.31	0.278171	0.585315
COMPOUND_550_2637a7_0117373	Rp	+	550.2637	7.01				(573.2534, 83318.17)(574.2564, 22075.96)(575.2584, 5035.6)(576.2611, 916.83)(589.2257, 2746.84)(590.2318, 851.51)(591.2291, 516.36)(592.2327, 86.28)(568.2969, 934488.2)(569.3013, 250401.66)(570.3031, 52565.93)(571.3055, 8529.97)(572.3068, 1425.14)(551.2705, 48957.36)(552.2743, 12928.92)(553.2751, 3208.21)(554.2796, 637.49)(555.2843, 105.2)	3023968	1555534	2949196	1287745	2950458	1346818	3274868	1389690	-0.13	0.678095	0.764682	0.46	0.205264	0.516497

Compound	Column	Ion_mode	Mass	Retention_	Identification	ID_level	MSMS_spectra	CompositeSpectrum	1st trimester				3rd trimester									
									Control		Pre-eclampsia		Control		Pre-eclampsia		Time			Interaction		
									Mean	SD	Mean	SD	Mean	SD	Mean	SD	Value	raw p	FDR_p	Value	raw p	FDR_p
COMPOUND_550_417a11_0225315	Rp	+	550.4170	11.02				(1139.7617, 1209.08)(573.4069, 10675.19)(574.3987, 2255.55)(575.4102, 13377.23)(576.406, 5374.66)(577.4094, 9690.87)(551.4467, 102.62)(552.4496, 69.59)(553.457, 987.06)	152980.5	107083	126811.3	85479.7	160336.6	58927.91	153027.9	65610.92	0.51	0.076355	0.128131	0.19	0.56486	0.785799
COMPOUND_550_4976a14_295705	Rp	+	550.4976	14.30	DG 16:0/16:0	1	10eV: 551.5036(100), 81.0666(7), 406.9026(2)	(551.5047, 20581.54)(552.508, 8122.08)(553.5084, 1568.05)(554.5122, 205.64)	37809.31	19121.75	39795.5	25270.93	38435.5	13255.13	74553.07	47084.84	0.40	0.106354	0.171691	0.51	0.085065	0.346484
COMPOUND_551_2798a12_528823	Rp	-	551.2798	12.53				(586.2409, 406.56)(587.224, 29.5)(588.2559, 538.47)(550.2731, 2476.53)(551.2751, 854.64)(552.2664, 30676.07)	136355.7	15262.07	141275.3	13688.56	139355.6	16727.02	131179.1	14935.95	0.23	0.417873	0.522806	-0.87	0.011953	0.168863
COMPOUND_551_3224a8_9443245	Rp	+	551.3224	8.94				(1103.7042, 89.17)(574.3126, 3473.62)(575.316, 1169.26)(576.3283, 1735.52)(552.3307, 26860.33)(553.3336, 8072.81)(554.3364, 1736.9)(555.3387, 279.76)	54570.7	46490.46	66469.11	55127.66	48406.67	22458.93	44884.22	15688.25	-1.11	2.17E-07	1.26E-06	-0.35	0.129181	0.425745
COMPOUND_551_3229a1_386341	Hilic	+	551.3229	1.39				(574.3136, 10288.03)(575.3172, 3312.7)(576.3288, 10322.82)(577.3324, 3136.49)(552.33, 7232.08)(553.3329, 2309.22)(554.3453, 3936.33)(555.349, 1301.27)(556.3589, 1289.57)	93638.57	99331.2	110998	77691.33	66633.75	47727.43	65858.18	37411.47	-1.07	7.6E-07	3.91E-06	-0.40	0.085157	0.346484
COMPOUND_552_134a8_21079	Rp	-	552.1340	8.21				(551.1275, 4649.25)(552.1287, 1083.09)(553.1274, 260.78)(554.1357, 57.09)	14119.49	1720.003	14611.69	1682.277	14630.07	1382.337	14694.74	1338.976	0.34	0.245265	0.340319	-0.23	0.505359	0.754687
COMPOUND_552_3546a13_408602	Rp	+	552.3546	13.41				(564.3585, 272.88)(564.8505, 146.11)(565.355, 227.12)(1127.6923, 155.73)(1128.6888, 190.34)(1129.717, 130.28)(553.372, 30846.66)(554.3754, 10281.57)(555.3717, 5088.5)(556.3716, 720.18)(1122.7103, 371.54)(1123.6936, 571.06)(1124.7002, 440.62)(277.1854, 6360.54)(277.6872, 2091.87)(278.1847, 975.65)(278.686, 222.66)	387531.4	35312.32	397485.8	38232.79	411212.9	21732.68	401032.4	34117.63	0.70	0.021072	0.041336	-0.47	0.191383	0.505089
COMPOUND_552_7225a10_183379	Rp	+	552.7225	10.18				(553.7304, 14554.48)(554.7355, 2637.27)(555.717, 2870.93)(556.7172, 429.91)(557.7295, 1252.9)	70616.06	30393.94	58892.95	28526.39	72544.54	16029.45	76515.42	19893.54	0.08	0.772242	0.833547	0.77	0.025671	0.221494
COMPOUND_553_0545a10_183234	Rp	+	553.0545	10.18				(576.0422, 715.15)(577.0335, 293.0)(554.0644, 6094.07)(555.0663, 462.06)(556.0474, 796.4)(557.0333, 82.27)(558.0583, 495.16)(1124.1644, 224.29)	55758.1	22979.86	45808.4	22395.11	58586.08	12728.96	58942.96	16647.27	0.14	0.623081	0.714806	0.64	0.061096	0.302474
COMPOUND_553_3272a10_105649	Rp	-	553.3272	10.11				(1141.6102, 1710.16)(1142.6088, 1035.21)(1143.6086, 1317.1)(1144.6123, 775.2)(666.3182, 507.49)(667.3211, 168.73)(1151.6497, 10139.19)(1152.6517, 6053.73)(1153.654, 2746.6)(1154.6517, 703.5)(552.3131, 3838.8)(553.317, 1138.64)(554.301, 41585.28)(1107.6625, 73042.99)(1108.6658, 44663.05)	469128.1	99875.78	482884.7	83311.55	280693.5	52910.05	273030	71736.03	-1.39	1.39E-09	1.46E-08	-0.20	0.383476	0.686377
COMPOUND_553_3369a10_175268	Rp	-	553.3369	10.18				(552.3299, 22630.23)(553.3329, 6443.04)(554.3276, 817.82)	120763.4	27323.32	126958.9	29957.57	75283.57	15846.13	78796.59	24262.18	-1.21	4.69E-07	2.56E-06	0.11	0.671643	0.847836
COMPOUND_553_3376a10_353978	Rp	-	553.3376	10.35				(1151.693, 53.36)(1152.7067, 58.61)(1153.6641, 1964.7)(552.3297, 3749.07)(553.3335, 1092.59)(554.3452, 21620.26)(555.3486, 6030.78)(598.3517, 363.83)(599.3423, 216.05)	107592.1	19318.16	135623.7	87110.67	55475.5	13982.77	52945.19	13112.86	-0.75	0.001041	0.002704	-0.16	0.542035	0.776977
COMPOUND_553_3725a10_618265	Rp	-	553.3725	10.62	LPC O-18:1	1	20 eV: 492.3455(100), 44.9972(12), 168.0433(6)	(1141.7274, 490.06)(1142.7346, 237.09)(1143.7186, 1560.17)(666.3254, 1354.05)(588.3452, 910.3)(552.3664, 123964.84)(553.3696, 37666.96)(554.3691, 8022.92)(555.3659, 1721.81)(556.3557, 233.53)(1105.7545, 235.17)(1106.7579, 597.43)(598.37, 511.27)(599.3566, 778.64)(600.3687, 383.57)	521369.6	119190.3	533800.1	136735.5	222476	167376.6	186192.8	120839	-1.25	1.44E-07	8.77E-07	-0.25	0.311576	0.617667
COMPOUND_554_4033a9_407668	Rp	+	554.4033	9.41				(577.3923, 1069.26)(578.3937, 742.44)(579.4016, 870.44)(593.356, 180.19)(594.3502, 129.83)(572.4376, 22120.68)(573.4387, 7155.16)(574.4391, 1760.57)(575.4441, 295.92)(555.4104, 2799.49)(556.412, 1048.87)(557.4069, 331.89)	204149.1	48106.26	181034.2	30474.77	221965.4	39450.25	219320.6	56041.62	0.27	0.338079	0.438821	0.54	0.114104	0.400254
COMPOUND_555_2723a12_528823	Rp	-	555.2723	12.53				(554.2647, 10370.52)(555.267, 3352.24)(556.2682, 160.22)	62754.15	6482.957	64105.09	5927.455	62783.43	8515.524	59506.07	7001.292	-0.02	0.938928	0.960248	-0.59	0.077834	0.333235
COMPOUND_555_308a10_004767	Rp	-	555.3080	10.00				(1145.6086, 2133.07)(1146.6129, 1431.2)(1155.5892, 721.48)(1156.5912, 550.73)(1157.581, 746.09)(1158.5782, 753.38)(1159.574, 920.32)(554.3011, 17699.47)(555.305, 5563.43)(556.2997, 6887.97)(557.3099, 2792.12)(558.3113, 694.03)(559.3215, 165.53)(560.3201, 38.68)	81969.64	27751.94	89595.28	23285.03	78171.67	62498.78	77567.08	67471.31	-0.70	0.014923	0.03044	-0.17	0.602667	0.809307
COMPOUND_555_3523a10_480261	Rp	-	555.3523	10.48	LPC 17:0	2	20 eV: 269.2476(100), 44.9978(8), 78.9592(6)	(1145.6459, 1651.59)(1146.6486, 1159.56)(668.3072, 1252.87)(1155.6796, 7035.19)(554.3458, 150733.58)(555.3489, 42390.2)(556.3491, 9816.44)(1109.6846, 748.66)(1110.6777, 319.38)	759503.3	158041.6	738170.8	156488.7	371506.3	163080.8	339797.1	118383.5	-1.58	8.15E-11	1.28E-09	0.08	0.728385	0.883795
COMPOUND_555_3525a10_392928	Rp	-	555.3525	10.39				(668.3245, 1241.56)(554.3456, 149046.23)(555.3488, 42237.11)(1109.6812, 1900.78)(1110.6794, 1122.36)	209292.8	147598.9	186407.2	154889.6	135524.1	50195.82	138591.4	54332.39	-0.32	0.247958	0.343209	-0.02	0.944853	0.974642
COMPOUND_555_3886a10_904344	Rp	-	555.3886	10.90				(668.3533, 311.47)(554.3817, 23214.23)(555.3851, 7320.38)(556.3881, 1521.4)(557.3887, 287.61)(1109.7821, 881.97)(1110.794, 3868.43)(1111.7977, 2727.95)	191635.2	44807.01	201368.4	60211.55	64963.83	54157.07	47117	35688.21	-1.48	2.07E-11	4.66E-10	-0.16	0.448269	0.721588
COMPOUND_555_4868a12_667192	Rp	+	555.4868	12.67				(1149.935, 196.34)(1150.9408, 360.92)(1151.9482, 307.21)(556.495, 19671.57)(557.4985, 7108.42)(558.5024, 1493.79)(559.5098, 336.46)(1129.0283, 205.87)	29605.43	19691.59	29661.7	18982.27	25039	9888.632	44588	28355.58	0.05	0.838029	0.886862	0.66	0.042626	0.268638
COMPOUND_557_2245a12_524318	Rp	-	557.2245	12.52				(556.2168, 18490.56)(557.2204, 6027.15)(558.2148, 11503.26)(559.2172, 3889.61)(560.2139, 2398.29)(561.2155, 944.8)(562.2164, 212.97)	166695.9	43503.42	148548.5	35199.11	148133.8	34080.87	131598	38857.5	-0.42	0.117725	0.187521	-0.04	0.885909	0.952737
COMPOUND_557_2831a10_034626	Rp	+	557.2831	10.03				(1115.5925, 1675.2)(580.267, 2375.93)(581.2428, 2463.77)(577.2672, 2461.09)(577.7512, 1596.71)(578.2622, 2366.24)(1137.5822, 1859.82)(1138.5732, 1901.47)(1139.5747, 3051.86)(1132.5673, 2731.58)(580.2917, 5480.95)(580.7815, 409.32)(581.2798, 13983.93)(569.2666, 828.34)(569.7873, 705.95)(588.2439, 2769.28)(588.765, 1747.53)(589.2632, 1782.89)(589.768, 940.93)(558.2953, 11911.36)(558.7969, 7879.46)(559.3001, 13569.6)(559.8022, 5431.09)(560.3017, 5723.55)(560.7999, 2457.26)(561.2993, 2230.48)(301.6355, 1280.26)(302.14, 798.09)(302.6298, 488.29)(575.2914, 961.28)(596.2639, 14381.86)(558.2944, 3984.04)(559.2987, 2496.61)(560.3035, 1117.73)(575.3088, 830.86)(575.8046, 952.92)(576.3205, 1034.01)(279.6489, 24213.41)(280.1517, 6155.15)(280.6567, 1625.6)(281.1484, 261.41)	159000.2	124149.3	142937.2	106520	234929.7	166586.6	208498.8	192116.7	0.08	0.789623	0.84749	-0.09	0.787397	0.909997
COMPOUND_557_2881a10_104622	Rp	+	557.2881	10.10				(1115.6082, 3210.68)(580.2683, 560.91)(581.2785, 13935.37)(577.2686, 2379.33)(577.7519, 2056.26)(578.2625, 2854.49)(1137.572, 1726.01)(1138.5819, 1735.89)(1139.5864, 3370.87)(1132.5732, 2541.88)(580.2912, 6099.23)(580.7874, 612.97)(581.2798, 15087.82)(569.2583, 678.98)(569.7747, 639.66)(588.2417, 3606.99)(588.7628, 1614.47)(589.2722, 1415.8)(589.7709, 678.79)(558.2957, 9959.88)(558.7971, 9271.57)(559.2997, 17485.37)(559.8006, 7563.53)(560.3015, 6985.16)(560.8037, 3393.27)(561.3012, 2958.17)(301.6354, 1923.7)(302.1423, 924.21)(302.6335, 337.09)(596.2637, 14528.67)(596.758, 1337.08)(597.2638, 5019.4)(596.2644, 12651.13)(575.3112, 2013.42)(558.295, 9001.48)(575.3093, 1409.74)(575.7997, 1048.57)(576.3205, 1548.28)(279.6485, 21941.39)(280.1518, 5226.93)(280.6518, 1077.63)(281.148, 220.4)	355977.6	239795.1	274304.6	123459.2	326199.3	225485.4	311383.6	206974.2	-0.69	0.015753	0.032018	0.27	0.418434	0.69956
COMPOUND_557_3319a9_889187	Rp	-	557.3319	9.89				(556.3251, 30508.36)(557.3279, 8619.79)(558.3316, 1827.03)(559.333, 394.68)	77353.17	17518.59	73427.26	23472.5	35472.93	8203.208	38499.05	16583.12	-1.57	5.84E-11	1E-09	0.22	0.33492	0.63958
COMPOUND_557_3319a8_742867	Rp	-	557.3319	8.74				(556.3249, 34999.91)(557.3282, 9608.62)(558.3304, 2085.69)(559.3341, 321.58)	93189.09	29562.54	95353.13	35604.3	40125	12768.22	39941.87	14449.62	-1.42	6.69E-11	1.1E-09	-0.16	0.441887	0.717469
COMPOUND_557_3954a10_311741	Rp	+	557.3954	10.31				(1153.7433, 326.62)(596.3838, 2692.46)(558.4041, 8723.4)(559.4014, 1831.56)(560.3901, 1857.34)(561.3951, 329.23)(562.3947, 1562.99)	49505.86	16233.78	45321.84	20480.29	67797	22630.84	65199.77	28214.91	-0.03	0.917104	0.94696	0.75	0.026231	0.224785
COMPOUND_557_7826a10_094246	Rp	+	557.7826	10.09				(1116.5796, 13153.81)(1117.5837, 8901.8)(1118.5756, 4357.61)(1119.588, 2020.16)(580.7711, 934.99)(1138.57, 2118.1)(596.7589, 918.61)(597.7617, 577.71)(558.7977, 11513.18)(559.802, 7951.52)(560.8025, 3998.09)(1133.6475, 2099.76)(1134.6534, 1417.21)	157645	39779.74	130803.3	42375.16	99057.25	16871.21	108966	28413.55	-0.20	0.399358	0.503276	-0.11	0.689632	0.856181
COMPOUND_558_0521a9_926525	Rp	+	558.0521	9.93				(1117.1029, 296.84)(581.0506, 580.37)(582.0659, 644.63)(559.0618, 5574.37)(560.0614, 1081.58)(561.0472, 1172.62)(562.0397, 152.11)(563.059, 628.41)(1134.0988, 161.21)	48363.6	18640.9	39906.4	20012.6	42148.92	11811.33	45063.48	13338.4	-0.44	0.131739	0.205881	0.88	0.013838	0.179521

Compound	Column	Ion_mode	Mass	Retention_	Identification	ID_level	MSMS_spectra	CompositeSpectrum	1st trimester				3rd trimester				Time			Interaction		
									Control		Pre-eclampsia		Control		Pre-eclampsia							
									Mean	SD	Mean	SD	Mean	SD	Mean	SD	Value	raw p	FDR_p	Value	raw p	FDR_p
COMPOUND_558_4636a11_643689	Rp	-	558.4636	11.64	no msms			(593.4212, 82.93)(557.4563, 20682.4)(558.4597, 8405.4)(559.4637, 1784.9)(560.4666, 314.81)	154479.2	62708.28	176010.8	75184.89	213958.6	102257.5	134807.9	66641.39	0.66	0.016577	0.033451	-1.27	0.000145	0.017228
COMPOUND_559_2975a10_365007	Rp	+	559.2975	10.37				(1119.6008, 967.01)(579.2767, 2743.01)(579.7901, 653.19)(580.2869, 5517.12)(580.7897, 359.53)(581.2799, 11112.36)(581.7978, 164.32)(582.2813, 4284.13)(582.2819, 1957.23)(583.2908, 934.93)(584.2793, 1443.89)(571.2995, 645.29)(571.7927, 889.01)(572.293, 897.54)(572.7873, 502.2)(573.2764, 359.05)(1141.5992, 1226.18)(1142.5973, 1081.39)(1143.6165, 2673.41)(1144.6215, 1768.41)(1145.6025, 1256.97)(1146.6077, 769.57)(1147.5983, 789.43)(590.2676, 1365.0)(590.7845, 1327.26)(591.2604, 2904.33)(591.7523, 1405.11)(592.2659, 2950.83)(560.3115, 5612.58)(561.3123, 2169.99)(562.3158, 6546.32)(563.3159, 4169.46)(280.6571, 18887.84)(281.1584, 5650.97)(281.6592, 1195.84)(282.1545, 274.93)	467250.1	104810.9	479868.6	105431.7	322883	48197.92	327483.8	61904.4	-1.55	7.31E-11	1.18E-09	0.04	0.869544	0.945084
COMPOUND_559_3051a1_1042868	Hilic	+	559.3051	1.10				(1119.623, 890.58)(1120.6129, 947.59)(1121.622, 962.57)(1122.6295, 902.67)(1123.6394, 1142.02)(1141.6161, 439.44)(1142.6118, 458.29)(1143.5936, 850.28)(1144.5898, 1026.87)(1145.6182, 794.76)(560.3114, 6698.54)(561.314, 2115.56)(562.3272, 8998.58)(563.3312, 2727.22)(564.3149, 2703.81)	107315.7	17090.3	110407.7	17113.6	125685.3	14334.45	125481.4	12058.21	1.18	6.01E-06	2.51E-05	-0.20	0.480271	0.743153
COMPOUND_559_3272a1_385052	Hilic	+	559.3272	1.39				(582.3175, 6507.34)(583.3212, 2033.48)(577.3359, 320.33)(560.335, 27512.37)(561.3384, 8593.63)(562.3496, 12118.33)(563.3538, 3646.0)(564.3634, 2601.68)(565.3691, 772.65)(566.3801, 1300.97)	161360.7	93561.59	192053.3	110459.1	103738.7	100935.7	138980	107604.5	-0.96	2.97E-05	0.000105	-0.37	0.147901	0.456493
COMPOUND_559_3381a10_560094	Rp	-	559.3381	10.56				(1153.6407, 353.04)(1154.6367, 370.36)(1155.6493, 315.35)(1163.6559, 1015.67)(1164.6569, 780.78)(1165.6504, 455.38)(558.3323, 21197.61)(559.3349, 6640.03)(560.3308, 8082.93)(561.3338, 2132.23)(562.3327, 439.82)(563.3341, 137.09)(604.357, 483.32)(1231.6364, 374.36)(1232.66, 365.8)(1233.6436, 273.21)	103559.2	17577.93	106777.8	21764.28	52310.14	11566.25	54768.28	12229.25	-1.60	2.17E-13	1.48E-11	-0.04	0.823741	0.923377
COMPOUND_559_3391a10_655146	Rp	-	559.3391	10.66				(1153.6475, 763.05)(1154.6384, 353.36)(1155.6367, 381.32)(1156.6414, 274.38)(1157.6509, 621.02)(1158.6672, 318.56)(558.3324, 36189.23)(559.3355, 10675.7)(560.3306, 12775.75)(561.333, 3547.09)(562.3361, 831.64)(563.3389, 145.29)(564.3315, 831.08)	372582.7	39437.12	392132	50323.1	294952.8	33097.85	301399.5	45689.12	-1.30	5.3E-08	3.6E-07	-0.21	0.403759	0.6912
COMPOUND_561_308a12_530645	Rp	-	561.3080	12.53				(560.3007, 4817.84)(561.3044, 1626.55)(562.2953, 75321.38)(606.3173, 684.31)(607.3208, 426.98)(608.323, 226.15)	200937.7	49445.18	234600.7	52859.07	247620.2	80861.7	246118.5	59278.35	0.27	0.304062	0.404245	-0.50	0.108755	0.391238
COMPOUND_561_3202a1_0932081	Hilic	+	561.3202	1.09				(1123.6394, 1146.22)(1124.6439, 739.49)(1125.6543, 1254.54)(1126.6619, 919.04)(1127.6613, 972.0)(584.3077, 1325.7)(585.3065, 307.19)(586.286, 1731.33)(1145.6332, 578.37)(1146.6484, 501.76)(1147.6405, 714.72)(1148.6196, 634.22)(1149.642, 636.08)(562.3275, 9122.47)(563.3316, 2735.17)(564.3118, 4426.82)(565.3126, 1472.6)	80078.44	8665.829	84595.13	11993.81	90221.71	11984.69	90187.66	12664.04	0.88	0.003032	0.007218	-0.51	0.138268	0.44479
COMPOUND_561_3211a1_0057981	Hilic	+	561.3211	1.01				(1123.6538, 1315.14)(1124.6443, 761.79)(1125.6603, 1125.76)(1126.6562, 681.26)(1127.6681, 1245.87)(584.3088, 1229.48)(585.3126, 257.88)(586.312, 612.19)(1145.6305, 434.99)(1146.6625, 193.01)(1147.6403, 549.19)(1148.6425, 572.77)(1149.642, 560.63)(579.368, 130.96)(562.3276, 4753.54)(563.3305, 1295.27)(564.3097, 2635.37)(565.3128, 1033.42)	41413.14	6655.733	44783.02	7763.405	39422.8	34205.22	34045.46	29800.3	-0.63	0.02091	0.04109	-0.21	0.508236	0.756475
COMPOUND_561_3451a9_254577	Rp	+	561.3451	9.25				(1123.6427, 100.08)(584.3359, 4596.34)(585.3398, 1735.62)(586.3523, 1886.42)(587.3581, 666.69)(588.3478, 160.64)(579.3557, 1138.05)(562.3512, 13427.76)(563.3558, 4018.63)(564.3641, 1048.01)	61133.9	40295.43	71993.55	42478.85	93319.67	42170.56	64240.13	46934.23	-0.68	0.003738	0.008697	-0.23	0.393877	0.690408
COMPOUND_561_4155a0_8984425	Hilic	+	561.4155	0.90				(584.4084, 315.22)(585.4045, 146.79)(586.4192, 298.58)(1145.8073, 569.77)(562.4232, 2169.88)(563.4264, 666.83)(564.4363, 889.69)(565.4352, 382.55)(566.4273, 1263.13)	27066.17	11453.42	28025.67	13365.31	10552.44	6775.812	8271.364	4957.171	-1.38	1.73E-10	2.49E-09	-0.15	0.477404	0.740436
COMPOUND_561_4163a1_0634553	Hilic	+	561.4163	1.06				(584.4199, 499.97)(562.4232, 11223.75)(563.4272, 4091.06)(564.4391, 41950.18)(565.4426, 13856.24)	133235.8	48370.88	138273.5	66812.64	38225.29	28732.74	29809.77	22265.57	-1.06	3.79E-06	1.65E-05	-0.51	0.045208	0.273371
COMPOUND_561_5134a14_105435	Rp	+	561.5134	14.11				(562.5197, 21967.05)(563.5229, 8675.02)(564.527, 2009.88)(565.528, 377.77)	164060.9	41012.07	144926	38935.83	163227.4	31558.71	167524.4	38573.93	0.03	0.906137	0.938328	0.68	0.041327	0.265944
COMPOUND_562_0635a10_405621	Rp	+	562.0635	10.41				(1163.0828, 285.28)(585.0661, 440.13)(563.073, 6249.6)(564.0767, 1334.67)(565.0557, 1383.8)(566.0648, 301.76)(567.0703, 681.02)	51874.06	14929.63	44435.24	19062.7	56807.15	9311.64	60055.13	16404.15	0.28	0.322015	0.423801	0.61	0.073087	0.326093
COMPOUND_562_3864a11_393862	Rp	-	562.3864	11.39				(675.3918, 908.3)(597.3328, 121.73)(598.344, 38.53)(599.3159, 820.12)(561.3787, 16870.68)(562.3821, 6455.56)(563.3848, 1452.44)(564.3875, 194.27)(607.3999, 1204.22)	24730.27	35071.3	16103.94	5971.971	40966.62	40844.45	30765.24	28586.46	1.05	6.9E-05	0.000221	-0.20	0.485686	0.747178
COMPOUND_563_3213a9_729128	Rp	-	563.3213	9.73				(562.3141, 22116.88)(563.3175, 6767.33)(564.3238, 2246.99)(565.3287, 492.25)(608.315, 393.18)(609.3099, 166.0)	31834.2	12930.63	43538.74	39150.8	23984.38	8657.021	29107.98	29534.35	-0.52	0.047687	0.084096	-0.40	0.192856	0.505089
COMPOUND_563_3214a9_853186	Rp	-	563.3214	9.85				(562.3143, 78841.45)(563.3178, 22783.57)(564.3212, 5266.0)(565.3239, 971.56)(608.3096, 981.2)(609.3088, 188.64)	240131.9	103370.3	283552.6	137319.9	192887.5	81514.44	166724	67850.66	-0.60	0.024062	0.046353	-0.41	0.184813	0.499463
COMPOUND_563_4228a11_848587	Rp	+	563.4228	11.85				(581.4538, 1144.41)(564.4318, 5601.81)(565.4353, 2011.43)(566.4339, 800.91)(567.4555, 95.03)(1144.8542, 534.33)(1145.8632, 645.58)(1146.8765, 434.01)(1147.8751, 210.05)	45182.62	15638.29	43834	14858.86	25655.8	8046.772	23655.07	5628.688	-1.22	9.87E-08	6.16E-07	-0.06	0.816205	0.92157
COMPOUND_563_4319a1_0573426	Hilic	+	563.4319	1.06				(586.422, 2470.64)(587.4254, 917.23)(588.4382, 1891.79)(589.4408, 923.51)(564.4394, 15374.45)(565.4427, 5356.7)(566.4535, 5392.41)(567.4573, 1787.61)(568.4488, 225.1)	119887.4	44762.88	123976.4	59401.37	33921.14	25432.25	25579.8	19195.65	-1.29	2.83E-09	2.69E-08	-0.34	0.119271	0.408824
COMPOUND_564_1078a8_940244	Rp	-	564.1078	8.94				(677.1057, 845.86)(678.1061, 532.09)(599.0721, 539.07)(600.0749, 295.01)(601.0705, 278.16)(563.0997, 12997.26)(564.1043, 2742.31)(565.0978, 4128.0)(566.1049, 1144.93)(567.1111, 567.42)(609.116, 647.11)(610.1149, 410.01)(1241.2137, 115.75)(1242.2361, 79.77)	90224.13	10179.72	91951.93	10864.53	79968.43	10357.62	76490.28	11075.56	-0.79	0.002718	0.006546	-0.43	0.165055	0.47785
COMPOUND_564_2897a8_848619	Rp	-	564.2897	8.85				(563.2824, 11438.04)(564.2858, 3504.18)(565.2885, 839.72)(566.2909, 148.95)	15104.95	4207.13	18127.84	5980.966	51323.23	18128.04	50831.11	19101.52	1.36	4.6E-10	5.66E-09	0.07	0.758267	0.896744
COMPOUND_564_3059a12_531212	Rp	-	564.3059	12.53				(563.2985, 26107.95)(564.3013, 5136.8)(565.2999, 953.79)(609.2961, 431.72)(610.2862, 279.13)(611.2877, 349.71)	76531.21	17167.12	86143.46	17648.7	91517.29	23479.89	89910.07	19674.51	0.69	0.016213	0.032834	-0.51	0.127333	0.424221
COMPOUND_564_3985a11_675868	Rp	+	564.3985	11.68				(1129.8442, 692.0)(1151.773, 910.97)(1152.764, 641.75)(1153.7601, 285.02)(565.4037, 10050.42)(566.4083, 3988.01)(567.4143, 1458.89)(283.2081, 1184.51)(283.7099, 461.93)(284.2185, 583.12)	17386.59	8180.515	19771.53	6149.384	25289.3	10215.44	23803.85	9902.473	0.78	0.000886	0.002339	0.08	0.775487	0.905077
COMPOUND_565_2332a8_598216	Rp	-	565.2332	8.60				(564.2259, 75421.88)(565.2285, 29248.56)(566.2291, 11001.94)(567.2306, 2548.8)	26120.83	16718.18	16717.12	7427.226	69231.31	122440.8	43761.53	48432.47	1.04	9.84E-06	3.95E-05	-0.25	0.337626	0.642175
COMPOUND_565_3369a10_1097975	Rp	-	565.3369	10.11	LPC 18:2	2	10 eV: 504.3095(100), 564.3299(98),279.2318(17), 44.9983(2)	(564.3295, 940953.8)(565.3336, 266499.3)(566.3359, 52558.07)(567.3387, 8170.51)(568.3394, 1164.55)	5493651	1478750	5907122	1320936	3928852	858066	3604779	970758.8	-1.01	3.86E-05	0.000132	-0.43	0.112536	0.398247
COMPOUND_565_3373a9_995817	Rp	-	565.3373	10.00				(564.3301, 209700.52)(565.3335, 59677.51)(566.3356, 12193.35)(567.3386, 2259.07)(568.3411, 362.96)	1105269	408599.3	1186580	371131.3	658700	174098.3	622179.8	197790.9	-1.13	3.6E-06	1.58E-05	-0.31	0.240049	0.54495
COMPOUND_565_3509a11_236948	Rp	-	565.3509	11.24				(1165.7224, 410.25)(600.3226, 6298.28)(601.3246, 1543.34)(602.3296, 325.47)(564.3432, 42790.03)(565.3478, 16483.47)(566.3507, 3986.0)(567.3607, 877.28)(1129.6757, 324.02)(1130.6833, 432.1)	97831.4	26109.66	113609.2	22990.96	81943.36	23525.52	84639.47	34366.36	-0.72	0.008076	0.017522	-0.51	0.111492	0.397302
COMPOUND_565_4049a11_104023	Rp	+	565.4049	11.10				(1131.7887, 1327.54)(604.3798, 427.39)(566.412, 33986.93)(567.416, 17751.89)(568.4232, 9660.89)	62574.25	17977.64	46553	19429.64	255003.2	82609.98	216364.3	70749.55	1.47	4.76E-12	1.39E-10	-0.25	0.21429	0.521709
COMPOUND_566_2576a10_029896	Rp	+	566.2576	10.03				(567.2645, 7778.43)(568.2678, 2485.73)(569.2629, 706.58)(284.1343, 308.22)(284.6338, 80.6)(285.1346, 459.03)	25190.57	5756.044	25542.73	7968.538	16113.8	4401.748	15254.14	3928.165	-1.23	2.17E-08	1.66E-07	-0.10	0.64982	0.838541
COMPOUND_567_3305a10_305364	Rp	+	567.3305	10.31				(1173.5872, 570.44)(1174.628, 749.49)(1175.6298, 439.99)(590.3296, 2459.33)(1157.6476, 472.63)(606.3066, 6877.37)(568.338, 11888.42)(569.3405, 3726.08)(1152.6852, 453.7)(1153.7094, 810.26)(284.6662, 918.55)(285.1692, 226.8)(285.6636, 107.08)	48767.02	33532.43	52451.57	48396.31	32076.73	16269.45	38176.56	20688.8	-0.85	0.004518	0.010278	0.58	0.102165	0.377172

									1st trimester				3rd trimester									
									Control		Pre-eclampsia		Control		Pre-eclampsia		Time			Interaction		
Compound	Column	Ion_mode	Mass	Retention_	Identification	ID_level	MSMS_spectra	CompositeSpectrum	Mean	SD	Mean	SD	Mean	SD	Mean	SD	Value	raw p	FDR_p	Value	raw p	FDR_p
COMPOUND_567_3324a1_0725617	Hilic	+	567.3324	1.07				(1135.669, 3492.85)(1136.6696, 2116.93)(1137.6674, 2283.52)(1138.685, 1040.2)(1139.6931, 969.5)(1140.7052, 524.24)(1141.7104, 316.67)(590.3222, 9156.96)(591.3251, 2738.72)(592.3363, 3473.34)(593.3409, 1075.84)(1157.612, 830.16)(1158.6052, 932.64)(1159.602, 502.31)(1160.6226, 463.91)(585.3749, 20626.1)(586.378, 7234.58)(568.34, 420406.4)(569.3438, 133996.77)(570.355, 142940.64)(571.3587, 43331.82)(572.3701, 41699.74)	2344067	573313.6	2266273	591252.2	1306386	467429.1	1512970	605753.1	-1.45	2.58E-08	1.89E-07	0.39	0.144435	0.451201
COMPOUND_567_3331a0_9777065	Hilic	+	567.3331	0.98				(1135.6353, 1207.27)(590.3111, 1194.21)(591.3174, 1724.8)(592.3242, 674.56)(593.3321, 1388.81)(594.3303, 702.9)(585.3748, 13201.4)(586.377, 4679.18)(587.3764, 999.98)(568.34, 54732.92)(569.3436, 18233.71)(570.3549, 21359.91)(571.3591, 6330.76)(572.3694, 5678.07)(573.3744, 1810.08)(574.3823, 1485.37)	234301.1	61874.45	235371.7	63585.83	140135.3	52363.72	149806.6	55732.03	-1.31	4.18E-07	2.33E-06	0.11	0.698489	0.862515
COMPOUND_567_334a10_019982	Rp	+	567.3340	10.02				(1135.669, 2564.16)(1136.6741, 1704.3)(1137.6636, 765.45)(1138.679, 378.09)(1173.5846, 577.48)(1174.5767, 832.93)(1175.553, 911.72)(590.3237, 13389.17)(591.3256, 4697.9)(592.3275, 961.96)(593.3455, 179.49)(1157.5702, 641.84)(1158.5865, 731.05)(1159.5782, 774.97)(1160.5789, 886.21)(568.3421, 349818.9)(569.3456, 109262.1)(570.3491, 25282.9)(571.3516, 3995.25)(1152.6687, 116.29)	428227.2	460496.7	687554.4	674013.3	367484.1	494280.1	291431.1	351334.2	-0.91	0.001168	0.002997	-0.12	0.699358	0.862515
COMPOUND_567_3353a10_088961	Rp	+	567.3353	10.09	LPC 22:6	2	10eV: 568.3422(100), 104.1069(14), 184.0732(8), 311.2021(2)	(1135.6699, 8036.09)(1136.6732, 5074.87)(1137.6752, 2215.89)(1138.6825, 616.82)(1173.6049, 493.23)(1174.5643, 1367.34)(1175.5479, 900.27)(590.3225, 24510.55)(591.3259, 8407.89)(592.3276, 1917.66)(593.334, 269.92)(1157.6025, 816.58)(1158.5958, 503.33)(1159.5897, 848.47)(1160.5764, 943.38)(568.3415, 308729.25)(569.3446, 97691.52)(570.3483, 21611.92)(571.3496, 3855.51)(572.3433, 673.41)(1152.6909, 207.82)	1701706	446812.5	1523642	380324.1	908003.4	331576.4	1104965	471984.9	-1.50	1.89E-08	1.46E-07	0.65	0.019106	0.196411
COMPOUND_567_3523a10_376442	Rp	-	567.3523	10.38	LPC 18:1	2	20 eV: 506.3236(100), 281.2477(75), 44.998(5), 168.041(3)	(566.3452, 812793.1)(567.3492, 231045.34)(568.3517, 48095.76)(569.3544, 7251.78)(570.3586, 925.6)(612.3477, 2963.12)(613.3544, 1547.8)(1133.6906, 303.53)(1134.6779, 149.56)(1135.6831, 263.14)(1247.6469, 145.82)(1248.6647, 182.68)	8708403	1155285	8983571	1248465	5615457	832310.2	5891769	1114914	-1.65	1.82E-13	1.28E-11	0.05	0.798141	0.913784
COMPOUND_567_3524a10_27714	Rp	-	567.3524	10.28				(566.3452, 685226.7)(567.3492, 195864.38)(568.3521, 38956.48)(569.3549, 6204.62)(570.3571, 991.6)(1133.6906, 370.45)(1134.6908, 163.0)(1135.6938, 835.1)(1136.6968, 626.5)(1137.7042, 350.28)(612.3298, 1735.83)(613.3341, 570.98)(1248.6956, 153.66)	432419.9	59709.5	831431.2	1881389	304078.4	36971.98	307969.3	48743.76	-1.55	4.44E-11	8.19E-10	0.04	0.857648	0.940019
COMPOUND_567_4207a11_505867	Rp	+	567.4207	11.51				(1173.794, 454.33)(1174.7769, 1454.12)(1175.7762, 1401.7)(1157.8789, 29.45)(1158.8417, 275.24)(585.4542, 1062.78)(568.429, 23271.97)(569.4319, 11263.9)(570.4347, 2814.59)(571.4294, 575.71)	57502.92	28398.73	53375.76	31210.84	123902.1	34276.39	103786.2	46986.49	1.30	1.88E-07	1.11E-06	-0.12	0.650196	0.838541
COMPOUND_567_4213a11_512313	Rp	+	567.4213	11.51				(1173.7972, 489.53)(1174.7759, 1431.59)(1175.7762, 1404.25)(1157.8748, 304.55)(1158.856, 339.77)(585.4495, 472.36)(568.4288, 16106.9)(569.4325, 7321.55)(570.4364, 2215.58)(571.4502, 432.55)	53737.43	25163.06	49363.9	27766.14	114554.1	56425.76	94714.46	50075.26	1.18	3.41E-06	1.5E-05	-0.12	0.670227	0.846999
COMPOUND_567_4214a11_04483	Rp	+	567.4214	11.04				(1173.7911, 306.97)(1174.774, 1265.09)(1175.7882, 1341.29)(590.4196, 5732.92)(606.3748, 664.04)(568.4288, 255825.66)(569.4319, 114242.6)(570.433, 26189.49)(571.419, 6268.25)	237442.7	85517.38	206235.9	105796.2	264768.9	319182.4	415283.2	332609.6	0.26	0.309356	0.41053	0.34	0.25905	0.570391
COMPOUND_567_4215a11_021897	Rp	+	567.4215	11.02				(1173.853, 336.4)(1174.8417, 276.64)(1175.838, 182.86)(590.4227, 6127.24)(606.3802, 858.16)(568.4295, 306772.3)(569.4328, 129000.48)(570.4346, 28659.93)(571.4306, 5628.04)	286936.4	125470.3	273274	102504.1	769091.9	376971.8	682111.7	311220.1	1.53	2.19E-12	7.7E-11	-0.23	0.266655	0.575669
COMPOUND_569_2493a1_5842978	Hilic	+	569.2493	1.58				(570.2569, 4862.8)(571.2605, 1464.6)(572.2677, 741.1)(573.2728, 177.79)	22942.02	5243.09	24599.24	8168.333	18431.93	8300.592	23243.26	9712.053	-0.74	0.012396	0.025705	0.53	0.126526	0.423869
COMPOUND_569_2518a12_529054	Rp	-	569.2518	12.53				(568.2445, 25969.56)(569.2465, 8520.41)(570.2488, 1634.16)(571.2472, 365.09)	126477.6	20197.43	116436.1	19526.67	128724.4	11996.39	123025.1	19874.64	0.21	0.478946	0.581761	0.17	0.631748	0.828008
COMPOUND_569_3493a10_225313	Rp	+	569.3493	10.23	LPC 22:5	2	10 eV: 570.3549(100), 104.1069(17), 303.1917(6)	(1139.7098, 501.45)(1177.6667, 167.48)(592.3391, 6552.65)(593.3419, 2245.11)(594.3475, 718.1)(595.3322, 158.01)(1161.7379, 308.55)(1162.7358, 205.97)(608.2984, 5591.14)(570.3573, 86172.02)(571.3603, 28682.88)(572.3638, 5851.93)(573.3639, 874.6)	259269.1	99749.44	225597.7	95552.17	90823.93	32081.21	125959	55784.05	-1.77	5.96E-13	2.98E-11	0.74	0.001626	0.06002
COMPOUND_569_3494a10_285902	Rp	+	569.3494	10.29				(1139.7098, 501.45)(1177.6469, 586.87)(592.3384, 10070.02)(593.3412, 3457.29)(594.3444, 1182.24)(595.3393, 138.22)(1161.7345, 665.15)(1162.7281, 412.19)(608.3413, 1800.21)(570.3573, 46439.5)(571.3604, 15448.08)(572.3596, 1560.02)(573.3549, 277.75)	166835.8	107110.6	162087.4	96645.14	75558.79	33691.45	104660.1	48043.67	-1.15	0.00014	0.000423	0.57	0.098144	0.36878
COMPOUND_569_3681a10_651824	Rp	-	569.3681	10.65	LPC 18:0	2	10 eV: 508.3402(100), 568.3602(90), 283.2646(6), 44.9982(3)	(1183.7605, 759.21)(1184.762, 1387.05)(1185.7592, 1118.74)(568.3609, 817851.56)(569.365, 235295.88)(570.3673, 45687.98)(571.3697, 6984.99)(572.3715, 935.81)(614.3782, 565.77)	9505479	1154325	9723602	1292382	5141346	800222.8	5069928	971432.7	-1.50	1.67E-13	1.25E-11	-0.19	0.29948	0.607594
COMPOUND_569_3686a10_558576	Rp	-	569.3686	10.56				(1183.7562, 365.96)(1184.7513, 343.63)(1185.7622, 570.55)(1186.7675, 319.47)(1187.7695, 445.96)(568.3611, 422628.9)(569.3648, 120293.59)(570.3673, 25023.74)(571.3705, 3846.7)(572.369, 600.55)(1137.689, 317.15)(614.3631, 876.64)(615.3622, 1230.77)(1251.697, 261.8)(1252.7153, 331.39)(1253.7042, 392.75)	1759625	315060.7	1793996	342235.6	727350.1	157289.4	728200.2	175710.2	-1.55	5.31E-14	5.69E-12	-0.11	0.558125	0.781981
COMPOUND_570_2051a10_029141	Rp	+	570.2051	10.03				(588.2224, 441.74)(589.2493, 1020.24)(590.2472, 741.78)(571.2131, 11748.16)(572.2183, 5719.46)(573.2132, 4825.11)	38883.98	12263.18	40601.04	14966.07	20181.86	7521.111	20048.07	7207.608	-1.20	2.93E-08	2.14E-07	-0.23	0.300424	0.60896
COMPOUND_570_2408a10_505557	Rp	+	570.2408	10.51				(1141.4728, 206.77)(1142.474, 369.1)(1163.4769, 475.81)(571.2505, 9480.98)(572.2557, 5150.67)(573.2505, 4052.41)(1158.5597, 350.94)	37120.8	13354.18	35279.68	11997.72	25665.14	18029.5	21308.52	10281.59	-1.02	1.37E-05	5.33E-05	0.21	0.41737	0.699344
COMPOUND_571_366a10_434301	Rp	+	571.3660	10.43	LPC 22:4	2	10 eV: 572.3744(100), 104.1071(14), 184.0706(7), 425.3099(5)	(1181.6447, 565.31)(594.3522, 6493.05)(595.3563, 2085.03)(610.3133, 1668.91)(572.3736, 12391.75)(573.3785, 3966.25)(574.3852, 1477.18)(575.3743, 222.88)	139250.2	45881.29	123274.8	46345.77	47881.2	29716.42	80664.48	27236.23	-1.14	1.08E-06	5.33E-06	0.31	0.21104	0.519294
COMPOUND_572_0696a10_300524	Rp	+	572.0696	10.30				(1183.0765, 236.5)(1167.1234, 396.17)(573.0763, 5164.93)(574.0804, 1077.98)(575.0646, 1201.9)(576.0701, 206.95)(577.0739, 548.08)(1162.1704, 361.22)	42116.98	19039.67	35565.09	17699.06	51582.54	8301.274	52394.4	16961.75	0.40	0.156603	0.236691	0.55	0.10599	0.387484
COMPOUND_572_3074a6_028568	Hilic	+	572.3074	6.03				(1145.6364, 137.6)(595.2967, 6120.17)(596.2991, 2383.88)(1167.58, 153.84)(573.3148, 93310.12)(574.3176, 31033.24)(575.3197, 6179.95)(576.3226, 864.12)(577.3264, 69.83)(287.1604, 710.35)(287.6627, 243.27)	88798.98	135470.6	151296.2	180866.4	236908	421204.8	45174.14	73279.67	-0.92	6.65E-05	0.000214	0.06	0.800841	0.916038
COMPOUND_572_4807a13_043053	Rp	+	572.4807	13.04				(1145.9335, 139.05)(1146.943, 98.27)(1183.9108, 318.05)(1167.9406, 135.16)(573.4885, 7740.02)(574.4917, 3218.97)(575.4953, 875.48)(576.4915, 92.15)	56785.33	22049.8	52922.45	23338.37	59349.57	21391.66	81925.66	30213.23	0.33	0.186342	0.274455	0.69	0.02358	0.219225
COMPOUND_573_2556a10_071222	Rp	+	573.2556	10.07				(304.6537, 699.47)(305.16, 288.04)(1147.5201, 1422.02)(1148.5391, 1275.37)(1149.5228, 2062.21)(1185.4803, 310.55)(585.237, 2521.36)(585.7525, 440.16)(586.2406, 2417.0)(593.2691, 3060.98)(593.7533, 1636.09)(594.2585, 3045.31)(594.753, 811.98)(595.2583, 10757.65)(1169.5115, 342.32)(1170.5153, 186.76)(1171.5054, 374.7)(1172.5035, 270.49)(1173.5024, 484.17)(601.7582, 803.76)(602.2623, 1678.42)(602.7604, 2188.67)(603.2695, 1867.33)(603.7574, 495.41)(591.2642, 1202.72)(592.2574, 817.78)(593.2466, 1287.12)(574.2598, 6827.17)(575.2667, 2579.76)(576.2662, 3819.47)(577.267, 1744.77)(578.2647, 2037.56)(579.2761, 1455.21)(325.6002, 284.34)(326.0989, 112.36)(287.6352, 17317.12)(288.137, 4787.64)(288.6353, 9340.71)(289.1374, 2710.99)(289.6342, 3524.0)(290.1353, 1439.1)(290.6253, 1098.08)(1164.5754, 1529.38)(1165.5709, 918.3)(1166.5488, 954.55)(1167.5361, 360.38)(1168.5522, 788.2)	333213.9	156156.3	349565.3	131188.8	244505.1	151384.4	202746.9	151254.8	-0.60	0.033473	0.062043	-0.18	0.578456	0.792868

									1st trimester				3rd trimester									
									Control		Pre-eclampsia		Control		Pre-eclampsia		Time			Interaction		
Compound	Column	Ion_mode	Mass	Retention_	Identification	ID_level	MSMS_spectra	CompositeSpectrum	Mean	SD	Mean	SD	Mean	SD	Mean	SD	Value	raw p	FDR_p	Value	raw p	FDR_p
COMPOUND_573_3109a1_0502512	Hilic	+	573.3109	1.05				(1147.6154, 652.75)(1148.6316, 493.49)(1149.6426, 519.96)(1150.64, 457.54)(596.3151, 98.64)(1169.5741, 703.85)(574.3176, 9150.05)(575.3205, 2845.86)(576.3165, 1037.49)(577.3234, 197.04)(1164.618, 431.3)	49102.58	10414.87	48757.6	11072.39	77807.15	12764.19	86181.21	12839.69	1.00	3.59E-06	1.58E-05	0.66	0.00644	0.125019
COMPOUND_573_4807a13_921373	Rp	+	573.4807	13.92				(574.4871, 18366.67)(575.491, 6841.4)(576.4904, 1043.74)(577.4869, 205.21)	119038.3	38534.4	101506.6	39215.73	120071.8	25960.91	123825.2	32885.77	-0.08	0.76918	0.833186	0.39	0.229135	0.535521
COMPOUND_574_1353a9_321006	Rp	-	574.1353	9.32				(1183.2289, 250.88)(1184.213, 80.14)(573.1284, 9138.84)(574.132, 2047.88)(575.1342, 341.66)(1147.2343, 191.38)	50958.47	15536.13	47981.37	4757.905	49147.93	5764.338	49619.39	6420.262	0.06	0.837469	0.886862	0.14	0.693199	0.85806
COMPOUND_574_1356a9_204304	Rp	-	574.1356	9.20				(573.1289, 16321.9)(574.133, 3572.04)(575.1336, 320.35)(576.1204, 48.63)	83712.34	8944.078	83601	7998.938	78710	9558.823	81591.7	8859.027	-0.55	0.070555	0.119735	0.31	0.396398	0.690408
COMPOUND_574_1361a8_940168	Rp	-	574.1361	8.94				(687.1254, 374.97)(688.1075, 257.81)(573.1288, 70382.42)(574.132, 13241.31)(575.1351, 1711.39)(576.134, 381.08)	262570.7	25169.28	264084.3	17522.47	255557.5	17158.46	253024.4	16779.31	-0.31	0.282092	0.381545	-0.30	0.389863	0.689687
COMPOUND_575_271a10_366332	Rp	+	575.2710	10.37				(1151.5974, 572.74)(1152.6003, 699.63)(1189.5437, 811.43)(587.2635, 1206.95)(587.7565, 3021.66)(588.2587, 1586.84)(588.7521, 1186.97)(589.2705, 1037.6)(1173.5608, 894.26)(1174.5662, 655.21)(1175.5405, 779.59)(576.2751, 12295.04)(577.2784, 5223.47)(578.2591, 13699.62)(579.2608, 7214.17)(580.2687, 5362.65)(1168.573, 1717.18)(1169.5756, 1325.38)(1170.5686, 1382.36)(1171.5684, 870.07)(1172.5671, 495.98)(288.6428, 24614.0)(289.1443, 7880.57)(289.6438, 10419.45)(290.1455, 3204.0)(290.6415, 4480.43)(291.143, 1764.95)(291.6403, 854.82)	406294.8	52445.93	398389	55591.1	307596.5	37472.69	315048.7	45013.59	-1.57	2.24E-10	3.14E-09	0.33	0.177599	0.491946
COMPOUND_575_287a12_53095	Rp	-	575.2870	12.53				(574.2799, 4542.82)(575.2835, 1538.52)(576.2746, 68865.68)(620.2934, 763.52)(621.3002, 194.51)	171129.9	30428.66	165647.6	38054.55	204772	34896.56	209435.9	40640.71	0.29	0.29787	0.397867	0.03	0.931121	0.968985
COMPOUND_575_4614a11_883623	Rp	+	575.4614	11.88				(1151.9391, 404.09)(576.4671, 524789.44)(577.4707, 182698.47)(578.4703, 52783.81)(579.4705, 12101.36)(580.4743, 2168.32)	2768946	1342957	2171220	1148124	746660.4	1048920	494488.5	671845.2	-1.50	1.81E-09	1.85E-08	0.22	0.380999	0.683863
COMPOUND_575_5302a14_105693	Rp	+	575.5302	14.11				(576.5357, 470167.5)(577.5394, 183963.9)(578.5421, 37939.93)(579.5446, 5489.85)(580.5481, 863.88)	2324091	1055720	1868278	884406.1	2098360	511174.6	2361468	1001926	0.01	0.983145	0.99151	0.76	0.02457	0.219303
COMPOUND_577_285a10_638372	Rp	+	577.2850	10.64				(1155.6176, 599.92)(597.2768, 1828.79)(597.775, 1714.97)(598.29, 2605.77)(598.7918, 1288.78)(599.2805, 1911.76)(589.3074, 1026.31)(589.815, 1284.09)(590.3117, 902.05)(586.8074, 849.99)(587.2943, 625.68)(587.8073, 2133.09)(588.2952, 849.46)(588.7976, 1006.83)(578.2932, 2509.89)(579.2954, 1738.54)(580.3059, 3163.06)(581.3082, 1822.89)(582.3031, 1631.85)(1172.6097, 1670.6)(1173.6096, 856.87)(1174.6072, 1089.07)(1175.6044, 815.35)(1176.5996, 364.58)(289.6513, 11968.6)(290.1525, 3581.39)(290.6519, 5545.44)(291.1547, 1761.92)(291.6497, 1927.77)(292.151, 842.29)(292.6517, 264.54)	164389	48343.95	164287.3	48996.84	137889.6	29441.25	130252.7	26872.47	-0.72	0.008913	0.019098	0.07	0.824864	0.923716
COMPOUND_577_737a10_04196	Rp	+	577.7370	10.04				(600.7304, 255.79)(601.7492, 707.84)(578.7434, 12129.77)(579.7475, 1394.01)(580.7307, 1395.19)	46841.1	17356.94	43790.85	21931.88	48045.69	9062.49	48182.76	13676.47	0.04	0.886062	0.923801	0.30	0.397194	0.690408
COMPOUND_578_2591a9_931467	Rp	+	578.2591	9.93				(617.2443, 1246.75)(618.2348, 848.28)(579.2636, 10261.53)(580.2668, 3399.83)(581.2741, 129.42)(1174.5258, 155.31)	68449.2	15742.98	74049.33	21612.77	52783.69	11814.45	51640.47	23124.37	-0.07	0.80127	0.857257	-0.34	0.280491	0.588143
COMPOUND_578_2849a12_531047	Rp	-	578.2849	12.53				(577.2776, 27458.91)(578.2807, 6224.9)(579.2834, 1085.61)	60858.83	10062.72	61309.78	14682.66	74373.07	12188.7	75005.4	14369.37	0.89	0.00143	0.003617	0.08	0.796203	0.91372
COMPOUND_579_3095a10_093017	Rp	-	579.3095	10.09				(1193.6031, 1832.79)(1194.6058, 1304.02)(578.3006, 11979.54)(579.3035, 3307.39)(580.2998, 4079.04)(581.3093, 1735.41)(582.3127, 356.56)(583.325, 119.76)(624.2878, 2798.07)	87023.87	18834.36	91009.93	18616.77	58244.64	10092.12	68045.25	16207.41	-1.28	2.48E-07	1.43E-06	0.18	0.481135	0.743153
COMPOUND_579_3096a10_016066	Rp	-	579.3096	10.02	no msms			(1193.6038, 922.06)(1194.6095, 700.3)(578.3008, 7797.33)(579.3039, 2524.23)(580.3013, 3090.36)(581.3096, 1242.72)(582.3173, 585.98)(583.3209, 196.71)(624.3162, 1001.6)	29707.08	12819.57	26022.07	7218.974	18857	17665.73	36365.51	25652.45	-1.51	7.6E-07	3.91E-06	1.50	2.47E-05	0.006177
COMPOUND_579_3158a8_945963	Rp	-	579.3158	8.95				(578.309, 12804.16)(579.3121, 4102.2)(580.3249, 95014.9)	101098.8	79337.74	133956.9	92890.16	98990.67	11005.2	121144.7	33215	-0.84	4.89E-05	0.000162	-0.58	0.013204	0.173893
COMPOUND_580_2727a10_193052	Rp	+	580.2727	10.19				(1161.6006, 28.19)(1162.5645, 231.94)(1163.5826, 246.06)(1164.5468, 286.5)(1165.5283, 129.84)(1199.5153, 1425.89)(1200.519, 875.81)(1201.5232, 820.47)(600.2622, 2717.27)(600.765, 222.58)(601.2501, 1047.14)(603.2721, 1887.33)(1183.5317, 633.24)(1184.5388, 698.29)(1185.544, 735.24)(581.2792, 32230.66)(582.2827, 10414.17)(583.2772, 2215.04)(584.2925, 454.34)(291.1451, 2439.09)(291.645, 915.21)(292.1453, 211.1)(1178.566, 286.17)(1179.5714, 105.25)(1180.5522, 262.92)	121898.3	76035.76	144108.9	83159.53	158373.4	65233.04	154372	76343.78	0.50	0.095992	0.157217	-0.16	0.655792	0.841269
COMPOUND_580_2732a10_27787	Rp	+	580.2732	10.28				(1161.5712, 452.79)(1162.5829, 506.47)(1163.5892, 505.24)(1164.5903, 1080.93)(1165.6025, 1305.47)(1199.5483, 1148.1)(1200.5498, 768.14)(1201.5472, 1319.1)(600.2675, 4892.64)(600.7696, 854.27)(601.2581, 2618.77)(603.2454, 1570.27)(1183.5588, 551.63)(1184.561, 350.09)(1185.5536, 452.38)(581.2794, 25335.68)(582.2819, 7420.59)(583.2779, 1898.9)(584.2712, 350.53)(1178.5653, 492.48)(1179.5648, 615.78)(1180.5507, 322.47)(291.1422, 2423.18)(291.6454, 506.14)(292.1461, 217.52)	215157.4	161810.3	213216.7	160953.9	211724.6	66046.95	215597.5	75933.19	0.15	0.619309	0.711936	0.06	0.864083	0.943353
COMPOUND_581_2457a8_853452	Rp	+	581.2457	8.85				(604.2358, 154.04)(605.2315, 401.23)(606.2431, 205.9)(582.253, 9356.74)(583.2553, 3563.3)(584.258, 1125.43)	14002.77	3336.531	15257.2	4214.689	38850.64	15348.05	37852.66	14020.02	1.55	1.47E-12	5.93E-11	-0.13	0.531424	0.771657
COMPOUND_581_2811a10_091351	Rp	+	581.2811	10.09				(1163.5583, 1572.81)(1201.5413, 1181.23)(1202.5386, 981.64)(1203.5591, 481.99)(601.2631, 2107.67)(601.7606, 803.6)(602.2633, 1613.85)(602.7616, 2103.44)(603.2721, 1614.91)(593.2696, 2788.82)(593.7541, 2261.76)(612.2398, 2152.59)(612.7613, 269.06)(613.2589, 1071.77)(613.7658, 620.14)(1185.5658, 934.69)(1186.5521, 826.91)(1187.5411, 1033.41)(582.2854, 4375.11)(582.797, 1257.41)(583.2962, 2758.09)(583.7993, 829.11)(584.3013, 1537.61)(584.8019, 514.31)(585.298, 440.88)(313.6344, 690.41)(314.1385, 366.76)(314.6346, 123.14)(582.284, 6831.16)(583.2942, 3884.95)(1180.5612, 923.57)(291.649, 6469.1)(292.15, 1787.62)(292.6394, 845.6)(293.1344, 195.24)	219383.7	45723.26	213499.8	42349.14	168278.8	25679.88	178249.3	40648	-1.10	9.62E-05	0.000298	0.44	0.164315	0.47785
COMPOUND_581_293a9_766728	Rp	-	581.2930	9.77				(580.2857, 18263.8)(581.2886, 5357.82)(582.2889, 1369.64)(583.2936, 233.18)	46658.17	20793.8	63008.3	30395.29	48698.29	15782.72	39901.53	18327.48	0.12	0.649587	0.737859	-1.08	0.000956	0.050056
COMPOUND_581_3317a8_946457	Rp	-	581.3317	8.95				(580.3249, 30412.73)(581.3282, 9299.72)(582.3313, 1997.48)(583.3316, 344.67)(584.3373, 51.94)(1161.6785, 157.39)(1162.6741, 39.27)	123570.8	98627.4	166195.3	118234.4	106465.8	52001.16	131680.4	69694.43	-0.97	1.16E-05	4.62E-05	-0.45	0.070864	0.325554
COMPOUND_581_3675a10_571905	Rp	-	581.3675	10.57				(1197.6528, 290.96)(1198.6599, 373.94)(1199.6671, 393.13)(580.3612, 6079.66)(581.3638, 2082.31)(582.3672, 500.4)(583.3747, 97.01)(626.3561, 454.51)	50267.51	10808.66	53219.05	13092.65	27847.13	5874.314	29462.93	7647.221	-1.27	4.3E-07	2.39E-06	0.20	0.446014	0.72091
COMPOUND_581_5029a12_850153	Rp	+	581.5029	12.85				(1201.9419, 223.67)(582.5099, 16554.86)(583.5132, 6304.59)(584.5177, 1578.73)(585.5216, 261.61)(1181.0645, 171.48)	44657.74	21730.48	44317.54	21757.67	46485.14	19998.11	78821.68	38065.96	0.13	0.594921	0.690571	0.97	0.001028	0.051457
COMPOUND_581_7475a10_490611	Rp	+	581.7475	10.49				(1164.5211, 257.41)(1186.501, 173.15)(582.7524, 9651.86)(583.751, 1103.17)(584.7363, 838.6)(1181.5577, 355.18)	32266.58	10176.33	28433.72	9392.561	30905.55	6819.133	31670.66	9233.548	-0.12	0.679122	0.765074	0.62	0.071484	0.325554
COMPOUND_582_0725a10_165926	Rp	+	582.0725	10.17				(1165.1016, 154.77)(1203.076, 115.83)(1187.0844, 146.16)(583.0816, 10363.14)(584.085, 1967.06)(585.0681, 1972.32)(586.0582, 361.68)(587.079, 946.39)(1182.2294, 180.58)	52283.85	23880.22	45263.95	23981.26	55014.31	8037.183	55861.33	15705.97	0.23	0.42644	0.531163	0.56	0.108749	0.391238
COMPOUND_582_2076a9_9266615	Rp	+	582.2076	9.93				(600.2643, 1417.75)(601.2651, 761.72)(602.2453, 292.99)(583.214, 15148.95)(584.2191, 7832.83)(585.2144, 6521.51)	83253.96	24988.73	94210.51	35734.12	60238.57	17116.31	65285.8	34315.69	-0.43	0.082234	0.136743	-0.04	0.886004	0.952737
COMPOUND_582_248a8_187181	Rp	+	582.2480	8.19				(1165.5186, 363.66)(1166.5046, 242.25)(1167.5132, 204.0)(605.235, 549.75)(606.2408, 140.33)(621.2051, 334.23)(622.2266, 62.17)(583.2559, 67910.12)(584.2589, 24313.38)(585.262, 5058.17)(586.2646, 800.69)	62488.03	77413.91	39158.42	31250.13	23116.33	9960.075	30969.17	34470.75	-0.81	0.000187	0.000558	0.05	0.826661	0.923891

									1st trimester				3rd trimester									
									Control		Pre-eclampsia		Control		Pre-eclampsia		Time			Interaction		
Compound	Column	Ion_mode	Mass	Retention_	Identification	ID_level	MSMS_spectra	CompositeSpectrum	Mean	SD	Mean	SD	Mean	SD	Mean	SD	Value	raw p	FDR_p	Value	raw p	FDR_p
COMPOUND_582_249a11_367533	Rp	+	582.2490	11.37	Unknown	4	20 eV: 583.2532(100), 297.1195(25), 478.3266(3)	(1165.5021, 1992.8)(1166.5079, 1458.82)(1167.5187, 8971.12)(605.238, 9088.48)(606.2463, 3992.9)(607.2522, 5784.6)(608.2546, 2469.9)(609.2464, 751.03)(1187.5138, 220.34)(621.2037, 2587.25)(622.2089, 1060.63)(623.219, 2340.62)(600.3008, 2181.26)(583.257, 150212.64)(584.2621, 97779.23)(585.2711, 116165.55)(586.2747, 37597.11)(587.2769, 8747.16)(588.2791, 1621.16)(589.279, 1899.96)(590.2679, 761.27)	495015.1	469934.1	325079.2	237956	690362.1	533419.9	517121	440433.8	0.52	0.05177	0.090237	0.02	0.95717	0.980661
COMPOUND_582_3182a4_5278673	Hilic	+	582.3182	4.53				(605.3063, 1134.48)(606.3136, 382.07)(1187.6501, 269.98)(600.3229, 199.79)(583.3246, 17837.04)(584.3275, 5341.26)(585.3298, 1185.81)	17732.55	8914.625	19006.96	8780.493	11101	4755.373	8470.235	3924.751	-1.32	8.85E-10	9.91E-09	-0.16	0.452067	0.724079
COMPOUND_583_3274a1_3199148	Hilic	+	583.3274	1.32				(606.3168, 2487.39)(607.3203, 878.7)(608.3315, 947.78)(609.334, 399.03)(584.3347, 15728.73)(585.3379, 5498.16)(586.3492, 6462.26)(587.3529, 2161.23)(588.3648, 1819.93)	105010.8	73030.71	130389.1	79521.74	83071	66058.18	78284.59	88980.21	-0.99	4.24E-05	0.000144	-0.29	0.274252	0.583098
COMPOUND_583_5159a12_38974	Rp	-	583.5159	12.39				(582.5093, 7657.5)(583.5131, 2972.73)(584.5143, 586.09)(585.5164, 144.06)	16811.87	5865.533	17734.95	7981.128	21135.17	7861.154	21343.93	8639.133	0.40	0.150963	0.229398	0.08	0.807282	0.917894
COMPOUND_583_5169a13_459112	Rp	+	583.5169	13.46				(584.5257, 32187.71)(585.5289, 12594.86)(586.5327, 3037.49)(587.526, 675.8)	71911.66	33880.32	74643	41700.04	73904.36	28408.36	142854.1	78516.6	0.17	0.480527	0.583053	0.94	0.001225	0.056309
COMPOUND_583_5181a12_667186	Rp	+	583.5181	12.67				(606.5105, 4684.04)(607.5147, 2007.96)(608.522, 608.12)(1190.0555, 1116.58)(584.5253, 9179.22)(585.5286, 3732.82)(586.5305, 1000.83)(587.5393, 168.34)	47015.77	29509.31	43045.91	23491.45	44635.71	22030.47	56930.57	28146.48	-0.38	0.167533	0.251022	0.84	0.011668	0.167839
COMPOUND_584_2216a1_0488341	Hilic	+	584.2216	1.05				(585.229, 3273.15)(586.2336, 1374.25)(587.2276, 1385.33)(588.2321, 732.5)(589.2427, 202.1)(1186.535, 312.41)	15123.6	3523.452	15851.17	2884.691	23427.79	3281.435	24416.48	5042.285	1.43	1.01E-08	8.38E-08	-0.14	0.577734	0.792726
COMPOUND_584_2246a10_30371	Rp	+	584.2246	10.30				(1169.4655, 262.0)(1207.4556, 418.93)(602.2661, 2286.27)(603.2573, 1188.67)(604.2616, 2831.33)(585.2286, 25794.3)(586.2344, 13586.51)(587.2294, 10456.87)(1186.5098, 331.05)(1187.5148, 506.74)(1188.5232, 324.15)(1189.5438, 430.06)	124560.2	39954.67	109412.6	30414.38	139953.2	23089.03	134931.7	32017.74	0.49	0.081401	0.135688	0.34	0.315222	0.621785
COMPOUND_584_2248a10_1615305	Rp	+	584.2248	10.16				(1169.4971, 550.49)(1207.4517, 304.92)(1191.426, 348.29)(602.2616, 1357.98)(585.2286, 33188.7)(586.2346, 17752.76)(587.2299, 13124.5)	145249.5	38488.36	129877.3	38550.41	144928.4	34696.4	149082	37251.67	0.11	0.712283	0.788624	0.44	0.211987	0.519471
COMPOUND_584_2623a6_9321017	Rp	-	584.2623	6.93		4	10 eV: 583.2549(100), 227.1172(6), 74.5504(3)	(583.2556, 20817.44)(584.2585, 7484.91)(585.2608, 1421.13)(586.2566, 255.61)(1167.523, 249.33)(1168.5474, 185.94)(629.2584, 387.04)	48504.57	31258.88	44736.98	26413.63	57035.07	33528.29	41573.35	21537.18	0.25	0.333921	0.43518	-0.31	0.305237	0.613089
COMPOUND_584_2623a5_6700606	Rp	-	584.2623	5.67				(583.2553, 10003.74)(584.2581, 3880.14)(585.2613, 747.49)(586.2537, 115.78)	27648.39	15365.06	25559.84	13409.14	28685.83	17321.25	20088.86	9368.648	-0.09	0.699487	0.780984	-0.11	0.665353	0.844963
COMPOUND_584_2634a5_6885524	Rp	+	584.2634	5.69				(585.2718, 10673.63)(586.2749, 4007.42)(587.276, 975.9)(588.2792, 199.72)(589.2751, 35.86)(293.1407, 1623.54)(293.6415, 610.12)(294.1429, 187.91)	#DIV/0!	#DIV/0!	#DIV/0!	#DIV/0!	#DIV/0!	#DIV/0!	#DIV/0!	#DIV/0!	-0.16	0.533678	0.632881	-0.01	0.96228	0.981922
COMPOUND_584_2644a1_1423072	Hilic	+	584.2644	1.14				(607.2468, 127.66)(608.2399, 85.22)(609.2344, 801.7)(602.2935, 751.65)(603.2973, 404.5)(604.2825, 9482.93)(605.2849, 2670.41)(585.2708, 15896.39)(586.2744, 5928.95)(587.2783, 1357.67)(588.2773, 229.0)(1186.5634, 196.96)(1187.5511, 229.01)(1188.5521, 483.87)	47629.9	38505.78	36198.59	28544.37	67379.36	41035.46	52190.75	30674.26	1.10	2.65E-05	9.59E-05	-0.37	0.196003	0.507591
COMPOUND_584_2658a6_9457154	Rp	+	584.2658	6.95				(1191.5342, 97.81)(602.2967, 233.54)(603.3083, 93.91)(585.2723, 87373.56)(586.2754, 31634.04)(587.2786, 6850.54)(588.2816, 1256.41)(589.2802, 184.44)(293.1379, 756.0)(293.6395, 74.3)	233474.8	145036.8	200775.2	111711	265154.6	142697.7	198622.7	109022	0.26	0.310017	0.411165	-0.24	0.4296	0.707514
COMPOUND_584_3676a1_055654	Hilic	+	584.3676	1.06				(602.3943, 1168.27)(585.3752, 27010.02)(586.3786, 9464.27)(587.3822, 2097.93)(588.3789, 402.78)(293.1919, 6430.72)(293.6941, 2101.61)(294.197, 414.98)	12341.12	7537	14327.77	10100.74	48816.36	39211.74	40538.02	40970.36	1.42	8.29E-10	9.47E-09	-0.19	0.41327	0.696003
COMPOUND_585_2641a0_780989	Hilic	+	585.2641	0.78				(603.2772, 640.88)(586.2712, 215743.84)(587.2741, 56288.03)(588.2769, 14715.48)(589.2802, 2589.76)(590.2823, 424.5)	313674.2	420318	292372.8	402987.2	489121	525946.2	311151.6	438555.3	0.44	0.139498	0.215319	-0.57	0.112648	0.398247
COMPOUND_585_363a9_362009	Rp	-	585.3630	9.36				(584.3558, 5572.9)(585.3592, 1555.23)(586.362, 275.09)	18655.14	5955.441	18494.68	6320.297	#DIV/0!	#DIV/0!	10754	#DIV/0!	-1.37	4.13E-11	7.76E-10	0.10	0.624678	0.823156
COMPOUND_585_4464a9_695286	Rp	+	585.4464	9.70				(586.4532, 16799.29)(587.4536, 6331.22)(588.4545, 1332.28)(589.4569, 227.11)(1188.891, 68.25)	116988.7	31956.1	100508.4	19882.39	125676.5	22051.98	123583.5	31367.77	0.32	0.252488	0.348623	0.46	0.174554	0.488924
COMPOUND_585_5347a13_27537	Rp	+	585.5347	13.28				(608.5253, 4194.5)(609.5284, 1683.38)(624.5084, 852.01)(625.5108, 330.87)(586.541, 12947.62)(587.5443, 5153.32)(588.5465, 1186.87)(589.5502, 238.43)	42321	29667.22	35522.35	24253.1	30121.6	8894.083	55535.55	32664.21	0.15	0.520569	0.620038	0.71	0.014859	0.181233
COMPOUND_587_1693a8_431415	Rp	+	587.1693	8.43				(588.175, 5095.27)(589.18, 1000.0)(590.1945, 181.06)	16795.89	2251.171	16276.63	1953.49	16855.93	1638.52	17509.72	2091.254	0.03	0.910964	0.941916	0.38	0.28511	0.593016
COMPOUND_587_2341a10_154449	Rp	+	587.2341	10.15				(1213.4622, 205.67)(610.2255, 1364.94)(611.2333, 989.54)(612.2301, 805.33)(1197.4967, 337.63)(588.2353, 7432.48)(589.2441, 2633.37)(590.2343, 2518.11)(1192.519, 601.31)(1193.5226, 623.92)(1194.5244, 504.89)	101785.7	36037.71	96809.3	39002.11	76318.73	60648.8	100359.3	60814.1	0.00	0.995081	0.998768	0.38	0.226834	0.53434
COMPOUND_587_3214a9_755528	Rp	-	587.3214	9.76				(586.3142, 7971.35)(587.3173, 2565.93)(588.3226, 819.69)(589.3213, 191.24)(590.3182, 21.64)(1173.5837, 29.13)(1174.5911, 105.46)	98956.02	111276.9	60215.15	49735.99	55268.69	61962.01	33927.21	22365.42	-1.00	0.000405	0.001142	0.20	0.537779	0.776408
COMPOUND_587_3217a9_856026	Rp	-	587.3217	9.86				(586.3143, 328147.72)(587.3177, 101260.8)(588.3204, 20955.01)(589.3229, 3622.94)(590.3235, 568.16)(1173.5865, 161.67)(1174.5813, 78.6)	478975.1	348745	435900.8	253168.7	152963.4	90814.81	177554.1	111135	-1.48	1.86E-09	1.88E-08	0.27	0.276368	0.585315
COMPOUND_587_3601a10_474119	Rp	-	587.3601	10.47				(1209.7517, 258.26)(1210.7406, 364.71)(700.3145, 990.38)(701.3131, 380.28)(622.3328, 10869.27)(623.3344, 3495.51)(624.314, 1920.22)(1219.6475, 189.57)(1220.6833, 307.15)(1221.6691, 377.45)(586.3528, 3608.13)(587.3562, 1497.35)(588.3474, 3202.01)(589.3371, 144.69)(590.3475, 1271.26)(1173.7606, 1464.67)(1174.7667, 905.52)(1175.7698, 463.95)(632.3644, 1292.49)(1287.6884, 280.47)(1288.6862, 281.47)(1289.6781, 311.6)	120974.5	28329.14	130027.2	33314.1	71438.15	17823.72	76455.37	18514.38	-1.23	2.61E-06	1.18E-05	-0.06	0.822223	0.922833
COMPOUND_589_337a10_003053	Rp	-	589.3370	10.00				(588.3299, 74826.82)(589.3332, 24207.17)(590.336, 5210.2)(591.3382, 854.52)(592.3467, 155.33)	290693.2	74275.43	268550.7	75609.03	161018.7	126360.5	171216.5	140589.3	-1.43	5.43E-08	3.67E-07	0.28	0.306199	0.613089
COMPOUND_589_3379a10_09891	Rp	-	589.3379	10.10	LPC 20:4	1	20 eV: 303.2322(100), 528.3089(85),44.998(22)	(588.3298, 467615.78)(589.3334, 149190.55)(590.3361, 31017.83)(591.3384, 5275.85)(592.3394, 965.08)(1291.6294, 302.92)(1292.6422, 299.92)	1565910	340017.2	1487614	356670.4	782209.2	172091	922980.7	282602.4	-1.62	3.16E-13	1.98E-11	0.39	0.057927	0.298567
COMPOUND_589_4475a1_0222793	Hilic	+	589.4475	1.02				(612.4376, 634.94)(613.4311, 467.56)(590.4546, 13461.83)(591.4574, 5314.09)(592.4699, 39351.42)	116276.2	43221.23	118707.9	52814.39	52842.86	20509.15	39810.54	18976.12	-1.45	1.19E-11	2.9E-10	-0.07	0.716552	0.876415
COMPOUND_590_2821a10_148812	Rp	+	590.2821	10.15				(1181.5994, 955.32)(1219.5106, 652.35)(1220.5048, 787.28)(1221.5056, 903.94)(610.2747, 888.02)(610.7599, 567.37)(1203.5392, 980.23)(1198.631, 475.03)(296.1478, 14816.87)(296.6494, 4552.06)(297.1484, 8732.74)(297.6497, 2531.31)(298.1467, 3477.26)(298.6473, 987.58)(299.1445, 753.3)(299.6474, 228.96)	123215.4	34832.15	116407.8	26400.68	90246.36	13613.91	95083.53	28405.44	-1.38	7.64E-08	4.94E-07	0.35	0.186206	0.500401
COMPOUND_590_2823a10_250496	Rp	+	590.2823	10.25				(1181.6033, 770.78)(1219.5126, 296.49)(1220.5114, 115.6)(1221.5386, 263.9)(610.2785, 2008.27)(610.7617, 529.48)(1203.5503, 908.36)(608.3146, 3436.27)(608.8036, 153.09)(1198.5524, 288.56)(296.1482, 21717.01)(296.6498, 6873.42)(297.1493, 11901.6)(297.6505, 3507.96)(298.1469, 4225.62)(298.6482, 1463.93)(299.146, 968.82)(299.646, 359.85)	251618.9	62113.22	265064.7	57781.39	200677.9	17656.14	205175	26276.6	-0.85	0.002836	0.006801	-0.10	0.750256	0.89254
COMPOUND_590_4904a11_674094	Rp	+	590.4904	11.67				(613.4824, 3738.65)(614.484, 1943.68)(615.4989, 16932.81)(616.5026, 7285.6)(629.4756, 2223.97)(630.4727, 1548.44)(608.5029, 1553.72)(609.4918, 288.84)(610.5021, 233.49)(591.4989, 14530.95)(592.4949, 7687.88)(593.4904, 2332.63)(594.4791, 558.47)(595.4709, 72.25)	286826.2	145098.2	291073.1	118528.1	233580	218418.2	234012.4	151353.8	-0.92	6.62E-05	0.000213	-0.20	0.44523	0.720819
COMPOUND_590_4916a13_039617	Rp	+	590.4916	13.04	no msms			(1181.956, 156.85)(613.4806, 6321.32)(614.4836, 2551.8)(615.4895, 855.62)(616.494, 185.96)(629.4565, 727.73)(630.4608, 343.32)(631.4572, 258.88)(608.5254, 24471.01)(609.5286, 9859.98)(610.5337, 2523.41)(611.5398, 619.06)(591.4998, 2448.46)(592.503, 1143.36)(593.5073, 337.67)	103033.2	36336.02	92415.02	43488.04	121047.4	47673.37	186280.3	76109.48	0.32	0.179661	0.266533	1.10	0.000194	0.018868
COMPOUND_590_4917a13_157109	Rp	+	590.4917	13.16				(1181.9685, 260.42)(613.4811, 3313.24)(614.4859, 1406.59)(615.4928, 662.08)(616.4967, 124.0)(629.4514, 503.28)(630.4553, 238.37)(631.4544, 164.24)(608.5258, 11769.76)(609.5278, 5051.54)(610.5329, 1334.37)(611.5349, 299.84)(591.5006, 3058.18)(592.5022, 1283.05)(593.5052, 332.31)	88192.81	37042.24	98670.22	41994.32	96831.71	31709.05	127755.5	61996.02	0.35	0.207065	0.297771	0.20	0.535751	0.776357
COMPOUND_591_2484a8_851654	Rp	+	591.2484	8.85				(592.256, 6139.32)(593.2565, 2168.97)(594.2594, 2510.86)(595.2529, 1040.16)(596.2436, 1668.9)	12867.92	1936.341	13699.24	2159.413	28502.64	11214.86	28901.07	10015.77	1.52	1.03E-13	9.27E-12	-0.20	0.277039	0.585315

									1st trimester				3rd trimester									
									Control		Pre-eclampsia		Control		Pre-eclampsia		Time			Interaction		
Compound	Column	Ion_mode	Mass	Retention_	Identification	ID_level	MSMS_spectra	CompositeSpectrum	Mean	SD	Mean	SD	Mean	SD	Mean	SD	Value	raw p	FDR_p	Value	raw p	FDR_p
COMPOUND_591_3518a10_183858	Rp	-	591.3518	10.18				(704.3142, 488.29)(705.3228, 228.73)(590.3451, 57453.12)(591.3485, 18743.37)(592.3502, 4366.61)(593.3542, 726.7)(1181.7188, 173.37)	116848.7	37703.9	121409.9	44103.88	71700.57	15255.05	91093.51	37301.3	-1.35	8.26E-07	4.2E-06	0.47	0.107032	0.38909
COMPOUND_591_3528a10_293874	Rp	-	591.3528	10.29	LPC 20:3	2	20 eV: 530.3234(100), 305.2479(45)	(590.3459, 24995.48)(591.3487, 7927.77)(592.3494, 2034.03)(593.3532, 422.74)(594.3446, 75.54)	497141.3	161938	507621.1	176593.9	361447.4	76703.73	438419.2	132937	-0.94	0.000949	0.002488	0.49	0.127351	0.424221
COMPOUND_591_4373a13_97843	Rp	+	591.4373	13.98				(592.4444, 34689.73)(593.4473, 12627.48)(594.444, 5460.29)(595.4464, 1358.87)	391886.6	180808.6	305939.5	142099.2	355347.1	99966.45	375796.3	134169.9	-0.01	0.965839	0.980203	0.54	0.129734	0.42589
COMPOUND_591_4631a1_0163624	Hilic	+	591.4631	1.02				(1183.9143, 153.47)(614.4788, 4104.72)(615.4828, 1493.26)(609.4744, 208.77)(592.4702, 4912.35)(593.4735, 1877.79)(594.4855, 2142.07)(595.4891, 795.05)(596.4924, 128.83)	113574.9	45051.7	116067.5	55189.7	42084.64	19112.34	32974.09	16658.79	-1.29	1.42E-09	1.49E-08	-0.33	0.122635	0.414675
COMPOUND_591_4658a11_547967	Rp	+	591.4658	11.55				(614.4467, 2975.33)(615.4414, 1460.08)(1205.9482, 116.94)(609.5182, 970.8)(592.4742, 15962.67)(593.4756, 5823.0)(594.4757, 1412.79)(595.4661, 229.12)	102683.6	43060.83	114697.2	55105.27	47680.8	27437.41	36325.91	17069.61	-0.87	6.78E-05	0.000218	0.14	0.574336	0.790589
COMPOUND_591_7525a10_379943	Rp	+	591.7525	10.38				(1184.5605, 581.82)(1222.4991, 519.02)(609.7656, 948.58)(592.7589, 11971.08)(593.7615, 1670.73)(594.7516, 1816.46)(1201.5507, 2884.1)(1202.5472, 1807.64)(1203.545, 2170.13)	76504.33	18609.01	72246.6	20940.64	72019.43	15357.32	76645.09	17910.81	-0.04	0.899676	0.934535	0.50	0.155537	0.466404
COMPOUND_592_396a11_245408	Rp	-	592.3960	11.25	no msms			(705.3738, 608.01)(706.367, 482.71)(591.3892, 42053.09)(592.3927, 15133.53)(593.3953, 3655.06)(594.3986, 827.1)(1183.7574, 713.73)(1184.7526, 585.21)(637.4184, 266.96)(1297.7604, 348.76)(1298.7543, 387.65)(1299.7316, 360.04)	37848.35	10924.51	44902.02	13672.82	87814.43	51585.78	62345.95	26633.28	1.67	2.83E-09	2.69E-08	-1.04	0.000445	0.034251
COMPOUND_593_2729a10_09851	Rp	-	593.2729	10.10				(592.2648, 7907.44)(593.2673, 3065.43)(594.2783, 994.97)(595.2771, 213.49)(1299.5609, 685.45)(1300.547, 665.61)	24413.8	4765.198	25604.22	5805.366	27539.23	8314.436	31886.23	9209.84	0.22	0.418597	0.52284	0.55	0.086956	0.350026
COMPOUND_593_3316a10_455811	Rp	-	593.3316	10.46				(1221.6112, 296.01)(1222.6356, 376.89)(706.3452, 417.33)(1231.6184, 342.47)(1232.6198, 319.73)(1233.6128, 211.26)(592.3222, 10987.7)(593.3265, 3523.91)(594.3308, 877.21)(595.3287, 136.9)(1185.6139, 517.23)(1186.6112, 651.4)(1187.6136, 760.86)(1188.6187, 515.16)(1189.6108, 550.51)(1299.6531, 409.14)(1300.6753, 148.78)	53680.62	11394.13	56801.88	13714.13	23942	11452.18	24566.39	10182.69	-1.26	3.37E-07	1.91E-06	0.08	0.766467	0.902291
COMPOUND_593_367a10_514657	Rp	-	593.3670	10.51				(1221.6809, 1336.94)(1222.6848, 934.78)(706.3459, 562.32)(707.3364, 225.81)(708.3196, 68.6)(628.3501, 2198.76)(592.3608, 22984.89)(593.3644, 7230.09)(594.3421, 7734.78)(595.3443, 2298.54)(1185.7623, 877.71)(1186.7653, 743.61)(638.3626, 6476.47)(639.3721, 3193.43)(640.3766, 1248.55)(1299.7045, 312.71)(1300.7109, 329.6)	208937.6	57507.03	242074.2	72039.28	124126.3	37494.78	150440.4	54636.89	-1.13	1.21E-05	4.79E-05	-0.17	0.55279	0.780002
COMPOUND_593_3702a0_8402881	Hilic	+	593.3702	0.84				(1187.7671, 170.92)(1188.7401, 176.07)(616.3716, 930.2)(617.3689, 689.08)(618.3712, 1023.83)(619.3863, 639.66)(611.3883, 670.23)(594.3766, 11616.16)(595.38, 3859.86)(596.3813, 963.58)(597.3853, 214.69)	94618.02	35866.16	91408.3	40117.73	35938.13	28290.28	29624.65	23994.83	-1.33	9.61E-09	8.04E-08	-0.07	0.762264	0.899225
COMPOUND_593_3746a10_29375	Rp	+	593.3746	10.29				(1187.7415, 474.64)(1188.7417, 320.38)(1189.7605, 407.49)(1225.7642, 499.25)(1226.7352, 426.84)(616.3591, 759.54)(617.3579, 410.49)(618.3406, 107.95)(1209.7646, 484.05)(1210.7452, 446.35)(1211.7378, 298.84)(611.4277, 2267.79)(612.4334, 1510.72)(594.3772, 12510.53)(595.3866, 4787.51)(596.3867, 1652.67)(597.3738, 130.56)	39120.16	33505.14	34147.44	18804.84	21502.33	8038.721	19811.43	9126.448	-1.09	9.77E-07	4.88E-06	-0.04	0.857789	0.940019
COMPOUND_594_4104a11_399655	Rp	-	594.4104	11.40				(629.3661, 2604.86)(630.3685, 1060.3)(593.4049, 5266.56)(594.4072, 1749.39)(595.4107, 664.02)(596.4154, 117.56)	16042.23	3715.1	18060.22	4280.69	21852.93	7522.033	20745.71	5967.281	1.02	0.000552	0.00152	-0.64	0.059298	0.30009
COMPOUND_594_4851a10_978287	Rp	-	594.4851	10.98				(707.4704, 2762.83)(708.4706, 1590.74)(709.4862, 3700.16)(593.4773, 28451.71)(594.4806, 10523.68)(595.4929, 32767.63)(596.4965, 12445.67)(597.4998, 2839.08)(639.4936, 788.04)	184323.5	116065.7	210606.2	111887.3	216304	129016.9	145946.7	136772.6	0.03	0.920873	0.94911	-0.84	0.014613	0.179825
COMPOUND_595_2387a9_850974	Rp	+	595.2387	9.85				(618.245, 1344.65)(618.7496, 1010.24)(623.7334, 234.95)(624.2475, 640.84)(624.7445, 401.06)(596.2437, 5463.56)(597.2483, 1956.27)(598.252, 2976.56)(599.2494, 1179.37)(600.2538, 1428.46)(336.5837, 168.38)(337.0783, 76.25)(337.5864, 291.05)(1208.5085, 413.75)(1209.5092, 193.45)(298.6276, 9593.21)(299.1281, 3605.49)(299.6277, 4909.18)(300.1286, 1508.28)(300.6258, 1959.1)	#DIV/0!	#DIV/0!	#DIV/0!	#DIV/0!	#DIV/0!	#DIV/0!	#DIV/0!	#DIV/0!	-1.05	2.67E-05	9.62E-05	0.01	0.984414	0.992345
COMPOUND_595_2574a10_244268	Rp	+	595.2574	10.24				(1191.5066, 285.42)(1192.5026, 315.2)(1193.5079, 143.51)(1229.4989, 134.65)(1230.4934, 150.15)(618.2481, 3866.2)(619.2496, 1763.07)(620.2497, 989.41)(1213.4467, 70.92)(1214.4615, 70.92)(1215.4738, 102.3)(1216.4784, 64.42)(1217.4879, 137.79)(634.238, 1030.72)(596.2649, 21636.81)(597.2679, 6184.94)(598.2665, 10398.85)(599.2692, 2780.34)(600.2626, 4068.75)(601.2579, 2003.0)(602.2606, 1334.53)(1208.5345, 258.08)	132978.2	87880.28	131906.9	94052.18	184766.2	68512.07	154381.1	97445.18	0.51	0.060053	0.103715	-0.03	0.915254	0.961085
COMPOUND_595_259a10_259193	Rp	+	595.2590	10.26				(1191.5555, 865.31)(1192.5529, 810.52)(1193.5532, 1067.59)(1229.5138, 397.52)(1230.4875, 215.27)(618.2613, 5047.28)(619.2504, 1048.55)(620.2561, 1926.37)(1213.5092, 297.36)(1214.5197, 177.32)(1215.5306, 435.83)(634.2431, 966.37)(596.2655, 13561.38)(597.2669, 4379.96)(598.2666, 6906.84)(599.2681, 2920.35)(600.2659, 3641.69)(601.2569, 2282.92)(602.2698, 1722.11)(1208.5813, 360.02)	161599.2	96417.33	134938.9	78008.31	188431.9	93526.73	210535.2	95828.61	0.40	0.162613	0.244789	0.41	0.231067	0.537849
COMPOUND_595_2595a10_152486	Rp	+	595.2595	10.15				(1191.5322, 922.82)(1192.5258, 761.83)(1229.51, 469.99)(1230.4987, 501.54)(1231.491, 365.7)(1213.5109, 677.28)(1214.5242, 514.93)(1215.5331, 602.81)(1216.5165, 533.24)(1217.5203, 533.67)(634.235, 3729.54)(613.2645, 828.62)(596.2643, 7318.43)(597.2651, 2966.0)(598.2636, 4704.42)(599.2647, 1550.35)(600.2662, 2421.49)(601.2649, 1313.23)(602.2638, 964.67)	229381.3	50502.39	197837.1	48047.85	205644.6	29575.34	214324.1	41886.63	-0.41	0.144926	0.221721	0.82	0.016302	0.18924
COMPOUND_595_3054a12_4282465	Rp	-	595.3054	12.43				(594.2975, 771.66)(595.299, 271.06)(596.292, 6876.15)	23718.12	4526.616	21341.27	3968.488	25339.21	2510.227	25034.69	3606.349	0.36	0.191423	0.280289	0.26	0.425632	0.705315
COMPOUND_595_3082a10_039574	Rp	-	595.3082	10.04				(594.3011, 10212.9)(595.3051, 2782.65)(596.3017, 692.39)(597.3036, 168.91)	40978.89	10052.55	44764.37	11372.84	25036.79	6181.951	21921.53	6182.952	-1.16	2.15E-08	1.65E-07	-0.55	0.0111	0.165539
COMPOUND_595_3473a10_218753	Rp	-	595.3473	10.22				(1225.6915, 208.26)(708.3186, 206.56)(630.3146, 1040.35)(631.3162, 396.61)(594.3395, 10726.36)(595.3431, 3347.07)(596.3462, 879.62)(597.3488, 164.59)(640.3448, 429.03)(641.3472, 210.44)	28879.89	14674.46	25465.84	13072.42	#DIV/0!	#DIV/0!	10617	#DIV/0!	-1.34	5.82E-10	6.93E-09	0.38	0.082127	0.343638
COMPOUND_595_3483a1_3578442	Hilic	+	595.3483	1.36				(1191.7489, 111.75)(618.3392, 3869.23)(619.3416, 1360.69)(620.3476, 430.32)(596.3565, 31718.48)(597.3601, 9820.91)(598.3624, 2287.68)(599.3673, 508.04)	74659.54	47750.68	66107.15	45631.5	8297	1858.894	8211.538	2618.689	-1.63	2.12E-12	7.57E-11	0.19	0.38307	0.686295
COMPOUND_595_3486a10_529693	Rp	-	595.3486	10.53				(1225.6584, 318.2)(1226.6931, 380.42)(594.3405, 8971.38)(595.3432, 2829.8)(596.3485, 701.04)(597.3657, 94.97)	58026.79	10989.36	63169.5	14544.61	26242.4	16203.07	28343.54	15287.36	-0.97	2.44E-05	8.9E-05	-0.23	0.370567	0.671914
COMPOUND_595_3488a10_2031355	Rp	+	595.3488	10.20	BMP 22:2 or PG 22:2 or PS 20:0	2	20 eV: 184.0742(100), 596.359(70), 89.0579(12), 514.3753(5)	(618.3387, 2298.81)(619.3372, 1204.41)(620.3518, 1122.72)(634.293, 996.57)(613.4094, 1412.27)(596.3573, 33396.77)(597.3607, 10610.19)(598.364, 2848.0)(599.3646, 350.42)	66014.1	40544.26	58222.3	35548.99	#DIV/0!	#DIV/0!	#DIV/0!	#DIV/0!	-1.45	3.88E-12	1.16E-10	0.23	0.247694	0.557807
COMPOUND_595_3836a10_741276	Rp	-	595.3836	10.74				(1225.7418, 287.3)(1226.766, 547.9)(1227.7949, 312.31)(708.3849, 287.62)(709.4031, 457.83)(1235.769, 453.7)(1236.756, 495.44)(594.3767, 43040.92)(595.3799, 14065.34)(596.3876, 5345.42)(597.3894, 1168.45)(1189.774, 103.26)(1190.7418, 259.35)(640.3985, 1039.29)(641.4028, 656.25)(1303.7832, 298.99)(1304.7856, 323.97)(1305.7903, 307.33)	217402.2	58591.33	219351.3	52632.27	102723.2	33506.42	111121.8	28954.31	-1.78	1.04E-12	4.7E-11	0.32	0.176496	0.491826
COMPOUND_595_3849a10_650845	Rp	-	595.3849	10.65				(1225.7347, 118.86)(1226.7407, 359.45)(708.3468, 275.58)(709.3392, 224.33)(1235.8184, 90.19)(1236.7942, 81.89)(594.3769, 32386.25)(595.3798, 10734.8)(596.3869, 3849.45)(597.3919, 942.65)(640.3999, 3287.62)(641.4028, 1361.96)(1189.7694, 337.64)(1190.7719, 264.65)(1303.7927, 131.28)(1304.782, 179.61)(1305.7904, 139.4)	65020.34	76209.58	82533.48	99145.3	31374.71	42122.88	24892.08	29598.26	-0.89	6.37E-05	0.000207	-0.40	0.109522	0.392745

									1st trimester				3rd trimester									
									Control		Pre-eclampsia		Control		Pre-eclampsia		Time			Interaction		
Compound	Column	Ion_mode	Mass	Retention_	Identification	ID_level	MSMS_spectra	CompositeSpectrum	Mean	SD	Mean	SD	Mean	SD	Mean	SD	Value	raw p	FDR_p	Value	raw p	FDR_p
COMPOUND_596_4997a11_114499	Rp	-	596.4997	11.11				(631.4763, 1052.07)(709.4699, 4907.02)(710.4722, 2164.26)(711.4554, 134.73)(595.4932, 30074.08)(596.4966, 11988.68)(597.4995, 3420.29)(598.5044, 719.99)(641.468, 249.13)(1191.9965, 331.02)(1193.0126, 42.62)	129729.7	87613.1	102865.8	30701.71	130202.7	102879.8	108767.3	114957.1	0.10	0.70814	0.787133	-0.28	0.390918	0.690408
COMPOUND_596_5a11_068715	Rp	-	596.5000	11.07				(631.4775, 4188.83)(709.4854, 3554.52)(710.4876, 1608.03)(711.4938, 1655.06)(595.4931, 49065.53)(596.4967, 18760.97)(597.4998, 4339.88)(598.5028, 819.85)(1191.9905, 397.98)(1193.0143, 69.16)(641.5162, 162.69)	142786.1	93257.8	166157.7	95528.95	183497.7	155169.3	148720.6	209830.3	-0.15	0.543599	0.641606	-0.78	0.00887	0.149233
COMPOUND_597_0816a10_156655	Rp	+	597.0816	10.16				(1195.217, 124.29)(1233.1022, 156.51)(620.0817, 285.38)(621.0973, 617.84)(1217.1967, 139.58)(598.0904, 13318.37)(599.0935, 1123.98)(600.0727, 1617.09)(1212.2216, 79.95)(1213.2352, 229.49)	37043.58	15243.51	31928.43	20084.64	40717.85	11551.75	37779.87	11976.98	0.26	0.362362	0.464714	0.46	0.18111	0.494376
COMPOUND_597_3092a1_0271055	Hilic	+	597.3092	1.03				(1195.5789, 990.18)(620.2931, 537.45)(598.3168, 2233.92)(599.3194, 775.48)(600.3322, 5553.55)(601.3357, 1768.18)	30395.74	8056.283	29351.96	8769.487	34250.29	9974.302	32872.51	7507.52	0.53	0.076933	0.129004	-0.05	0.893787	0.954308
COMPOUND_597_3265a8_944314	Rp	-	597.3265	8.94				(596.3199, 59294.0)(597.3232, 18103.47)(598.3253, 3975.96)(599.3274, 876.84)	55528.45	46639.18	65480.67	52749.17	51195	#DIV/0!	43533.57	14030.02	-0.98	4.58E-06	1.96E-05	-0.38	0.107121	0.38909
COMPOUND_598_2383a10_454326	Rp	+	598.2383	10.45				(1235.4622, 251.79)(1219.52, 255.62)(616.2644, 3163.28)(599.2453, 7135.56)(600.2507, 3964.09)(601.25, 4219.6)(1214.5215, 277.45)	71776.47	25382.01	72039.76	33749.05	52335	19246.65	36097.53	9599.59	-0.96	5.1E-05	0.000169	0.19	0.463264	0.731607
COMPOUND_598_4347a10_256556	Rp	+	598.4347	10.26				(621.4364, 2327.44)(622.4416, 1374.94)(637.3931, 3316.39)(599.4396, 26488.04)(600.4436, 9340.71)(601.4428, 17408.6)(602.4365, 8191.26)(603.4295, 1467.72)	178994.2	61922.47	146416.9	60847.07	225823.5	39872.81	227258.3	40887.83	0.73	0.005892	0.013125	0.54	0.084252	0.346484
COMPOUND_598_4978a11_762764	Rp	+	598.4978	11.76				(621.49, 1162.35)(622.4918, 820.16)(1219.9811, 238.71)(1220.9763, 264.31)(1221.9823, 177.87)(637.449, 972.23)(638.4581, 633.55)(616.5157, 1111.98)(599.5044, 8205.58)(600.5088, 3427.17)(601.5082, 1092.36)(602.5061, 211.12)	56745.9	30859.36	56233.43	22985.37	43255.25	25144.51	58148.73	30164.66	-1.12	1.87E-06	8.79E-06	-0.08	0.738715	0.888668
COMPOUND_599_3261a1_0046843	Hilic	+	599.3261	1.00				(1199.649, 254.16)(622.3154, 1542.09)(623.3154, 582.44)(624.3303, 1269.53)(625.3316, 550.54)(617.3742, 260.02)(600.3325, 5924.14)(601.3349, 1877.18)(602.3478, 6075.38)(603.3514, 2001.34)(604.3513, 813.8)(605.3578, 183.69)	58874.88	12934.36	60075.88	12958.59	46973.43	8757.667	51268.53	10434	-0.49	0.095372	0.15643	0.06	0.86585	0.944172
COMPOUND_599_4607a9_955438	Rp	+	599.4607	9.96				(1237.8706, 119.23)(600.4688, 5464.06)(601.4666, 1874.78)	34179.46	9539.243	29402.9	6256.067	36208.85	6893.986	35909.07	9372.902	0.31	0.277487	0.376901	0.48	0.162292	0.475886
COMPOUND_600_259a5_2416496	Rp	+	600.2590	5.24				(601.2663, 5745.87)(602.2697, 2251.81)(603.2717, 632.28)(604.2739, 135.25)(301.1316, 3414.22)(301.6338, 881.18)(302.1316, 828.87)(302.6313, 92.1)	25302.64	11319.7	22256.4	9673.97	16563.4	5107.665	16408.87	6671.785	-0.87	6.53E-05	0.000211	-0.25	0.302436	0.610641
COMPOUND_600_5127a13_950206	Rp	+	600.5127	13.95				(601.5206, 69481.23)(602.5238, 29389.15)(603.526, 7410.7)(604.5261, 1403.47)	228192	69823.59	238703.5	84457.39	263653.4	80171.44	346870.8	165762.4	0.36	0.199408	0.289533	0.39	0.25412	0.566051
COMPOUND_601_2909a6_4889717	Rp	-	601.2909	6.49	no msms			(600.284, 61191.88)(601.2872, 19759.89)(602.2871, 6443.02)(603.2882, 1537.53)(604.2844, 251.81)	33247.18	26115.11	34534.1	14342.62	68350.79	107807.6	38978.25	40910.1	0.68	0.003808	0.008832	-0.92	0.000833	0.045795
COMPOUND_601_3415a1_005049	Hilic	+	601.3415	1.01				(624.3263, 403.76)(625.3222, 154.6)(626.3031, 4050.55)(619.3584, 270.77)(602.3481, 7170.3)(603.3516, 2310.67)(604.3457, 920.83)(605.3606, 123.79)	37475.87	10399.7	39464.51	10860.89	32507.5	8678.239	31952.19	10653.68	-0.48	0.100853	0.163869	-0.17	0.629137	0.827096
COMPOUND_602_1668a9_409308	Rp	-	602.1668	9.41				(637.1414, 27378.18)(638.1448, 5804.33)(639.1473, 862.19)(601.16, 1656.58)(602.1619, 518.23)(603.1521, 154.27)(1203.3563, 148.79)	171416.2	16136.83	175914.6	11449.41	175494.1	12128.69	171066	11260.82	0.37	0.219241	0.313281	-0.75	0.040692	0.265782
COMPOUND_603_3061a7_459699	Rp	-	603.3061	7.46				(602.3, 163911.81)(603.3029, 52615.02)(604.302, 16425.26)(605.3037, 4094.59)(606.3066, 773.78)	15030.21	4886.765	15611.44	4439.775	432388.6	1245718	116741.2	175075.8	1.73	7.34E-15	1.5E-12	-0.62	0.002367	0.067469
COMPOUND_603_3066a6_6695237	Rp	-	603.3066	6.67				(602.2999, 110280.84)(603.3027, 34572.08)(604.3022, 11315.06)(605.3045, 2672.43)(606.3071, 466.19)	19459.16	9641.545	20470	9252.327	141285.3	194228.4	96296.52	135633.3	1.61	4.85E-12	1.4E-10	-0.63	0.004905	0.108286
COMPOUND_603_3077a10_086181	Rp	-	603.3077	10.09				(1241.601, 933.23)(1242.6049, 823.56)(1243.6277, 1105.32)(1244.634, 631.01)(1245.6521, 233.15)(602.3007, 11318.58)(603.3035, 3651.22)(604.299, 4496.57)(605.3081, 1832.27)(606.3089, 796.47)(1319.5433, 569.5)	51277.66	14085.39	50723	10679.98	35754.21	10790.91	40316.88	12607.05	-1.07	7.36E-05	0.000233	0.36	0.221204	0.531023
COMPOUND_604_2729a10_095162	Rp	+	604.2729	10.10				(1209.5626, 682.28)(1210.5529, 712.43)(1211.5394, 680.43)(1247.4988, 625.87)(1248.4938, 410.87)(1249.4974, 496.86)(1250.4806, 531.49)(627.2509, 2748.13)(1231.5227, 322.31)(1232.497, 409.7)(1233.5172, 456.55)(1234.517, 536.48)(643.2524, 1007.55)(644.2535, 642.25)(605.2778, 13770.36)(606.2804, 5437.69)(607.2733, 831.77)(608.2891, 186.27)(1226.5468, 603.02)(1227.5319, 658.7)	121451.3	20104.78	116693	18807.01	125376.4	13212.22	117250.9	20960.9	0.15	0.599297	0.693461	-0.10	0.770498	0.902789
COMPOUND_604_2744a9_9952965	Rp	+	604.2744	10.00				(1209.5647, 361.81)(1210.5529, 225.64)(1211.5468, 240.48)(1247.5123, 355.44)(1248.5, 433.47)(1249.5049, 468.03)(627.2717, 450.35)(1231.565, 206.51)(1232.5374, 138.13)(1233.5775, 176.87)(1234.5754, 105.09)(643.2549, 2709.96)(644.254, 1394.14)(645.2397, 700.67)(605.2795, 10792.86)(606.2829, 4192.95)(607.2714, 1253.14)(608.2873, 143.3)(1226.5608, 196.06)(1227.5488, 120.72)	50340.73	21795.05	51889.11	20190.96	28484.29	7122.797	31174.4	22685.65	-1.11	1.94E-05	7.28E-05	-0.05	0.855433	0.938351
COMPOUND_606_0912a10_353612	Rp	+	606.0912	10.35				(1213.2289, 209.28)(607.0977, 7278.88)(608.0982, 1889.48)(609.0818, 724.02)(610.0919, 125.64)(1230.165, 306.75)	45172.35	18654.84	39603.84	18448.54	43629.46	7914.578	45690.42	12974.95	0.00	0.989681	0.995427	0.55	0.121092	0.411311
COMPOUND_606_287a10_365855	Rp	+	606.2870	10.37				(1213.6577, 392.4)(1214.6615, 505.44)(1215.6453, 340.57)(1251.5488, 394.27)(1252.5334, 427.08)(1253.5496, 281.26)(629.2601, 1162.17)(1235.5587, 337.73)(1236.5286, 586.02)(1237.5558, 417.87)(607.295, 25401.02)(608.2982, 8872.57)(609.276, 3911.92)(610.2594, 813.06)(1230.6476, 400.03)(1231.6476, 294.44)(1232.6232, 632.34)	215677.8	31367.33	200569.6	40025.41	190895.4	22641.94	192911.3	27726.63	-0.68	0.019345	0.038418	0.54	0.111105	0.397157
COMPOUND_606_4113a11_348935	Rp	-	606.4113	11.35	no msms			(719.3878, 608.07)(720.3906, 312.31)(721.3791, 179.87)(1257.7968, 275.59)(1258.8225, 176.93)(1259.772, 231.82)(605.4048, 26773.26)(606.4082, 11139.2)(607.4068, 2729.3)(608.4144, 657.81)(1211.7924, 226.71)(1212.7771, 426.94)(1213.782, 353.24)	52575.04	17078.75	57709.02	19814.58	123030.5	45102.51	82943.56	26707.27	1.67	8.35E-12	2.16E-10	-0.81	0.000745	0.045795
COMPOUND_606_4859a11_18748	Rp	+	606.4859	11.19				(629.4471, 8824.8)(645.4632, 19433.95)(646.4542, 11893.37)(647.4493, 4520.67)(607.498, 14739.75)(608.5012, 6015.9)(609.5116, 1602.98)(610.5196, 383.14)(304.252, 1655.92)(304.7522, 53.4)(305.2481, 518.58)	53215.76	72559.41	30305.59	16198.9	127033.8	38497.16	129058.9	37863.56	0.99	3.78E-05	0.00013	0.23	0.401173	0.690408
COMPOUND_606_7626a10_364663	Rp	+	606.7626	10.36				(1214.5387, 379.42)(1215.5225, 567.95)(1216.528, 412.01)(1217.5287, 470.51)(1218.5276, 371.13)(1252.5243, 344.21)(629.755, 1044.4)(1236.5193, 334.56)(1237.5427, 408.71)(1238.5405, 429.44)(1239.5217, 380.39)(1240.5275, 255.78)(624.7749, 650.06)(607.7682, 7541.93)(608.7539, 2908.25)(609.7448, 550.11)(1231.5328, 311.37)(1232.5454, 442.37)	52095.31	11155.23	54312.47	16600.85	44530	5877.984	46113.69	8619.951	-0.19	0.468451	0.57272	0.29	0.356201	0.665596
COMPOUND_607_3088a9_938235	Rp	-	607.3088	9.94				(606.3018, 16261.07)(607.3054, 4942.17)(608.3086, 1347.14)(609.3108, 204.95)	77731.7	27611.15	93612.89	29704.02	76836.46	12838.81	74280.23	28461.47	0.12	0.678627	0.764899	-0.45	0.180179	0.493854
COMPOUND_607_3286a12_427105	Rp	-	607.3286	12.43				(606.3213, 14758.93)(607.3248, 5601.11)(608.3268, 1287.53)(609.3217, 191.72)	81804.87	13864.55	77899.09	16815.29	93426.93	14560.41	92135.14	13116.88	0.73	0.008582	0.018529	0.19	0.555999	0.781554
COMPOUND_607_3464a9_241442	Rp	-	607.3464	9.24				(606.3396, 5468.15)(607.3433, 1784.22)(608.3461, 424.15)(609.3534, 82.72)(652.3673, 170.17)	20946.43	11330.7	24554.29	13530.29	24695	1061.544	29467.5	8714.823	-0.81	0.000566	0.001559	-0.37	0.171762	0.487538
COMPOUND_607_5166a13_038001	Rp	+	607.5166	13.04	no msms			(1215.9827, 188.52)(1216.9651, 62.83)(1238.003, 1897.52)(608.5255, 24120.87)(609.5286, 10054.89)(610.5327, 2553.35)(611.5374, 533.95)	76370.42	31104.2	64509.48	32863.67	80353.29	36207.48	134253.5	57899.05	0.12	0.605214	0.698229	1.29	1.14E-05	0.005768
COMPOUND_608_2219a9_998976	Rp	+	608.2219	10.00				(1255.4346, 245.06)(1256.451, 326.62)(1239.4297, 266.3)(626.2427, 1374.68)(627.2494, 1014.29)(609.2289, 22850.22)(610.2339, 11404.67)(611.229, 9159.5)	73481.69	25998.71	72235	25053.21	63165.57	46687.95	58780.61	42084.02	-0.50	0.078188	0.130869	-0.14	0.664371	0.844813
COMPOUND_608_2257a10_117158	Rp	+	608.2257	10.12				(1217.5006, 548.28)(1218.5186, 387.8)(1255.4568, 425.13)(1256.4381, 257.94)(647.2131, 1257.7)(648.2056, 651.94)(626.2452, 1323.44)(627.267, 831.17)(628.2474, 351.33)(609.2293, 16387.76)(610.2342, 9245.02)(611.23, 7422.04)	68807.63	37938.04	47749.36	28107.19	116650.4	29478.41	113437.4	37826.96	1.01	7.32E-05	0.000232	0.44	0.12809	0.424828

									1st trimester				3rd trimester									
									Control		Pre-eclampsia		Control		Pre-eclampsia		Time			Interaction		
Compound	Column	Ion_mode	Mass	Retention_	Identification	ID_level	MSMS_spectra	CompositeSpectrum	Mean	SD	Mean	SD	Mean	SD	Mean	SD	Value	raw p	FDR_p	Value	raw p	FDR_p
COMPOUND_608_3721a10_5784855	Rp	-	608.3721	10.58				(643.3316, 261.79)(644.3334, 576.28)(645.34, 201.84)(721.3492, 1635.38)(607.3658, 9427.71)(608.3683, 4004.24)(609.366, 18075.55)(610.3691, 7330.01)(611.3621, 7912.14)(612.3652, 3697.03)(613.3649, 1867.1)(614.3618, 1093.0)(615.3603, 2140.51)(653.3648, 1679.64)(1329.6528, 346.28)(1330.6254, 136.11)(1331.6326, 338.29)	46484.31	31198.25	54551.36	51063.54	51908.83	31710.86	71667.67	42471.42	-0.09	0.718123	0.79314	0.81	0.005799	0.118614
COMPOUND_609_1781a12_093475	Rp	+	609.1781	12.09				(1219.3135, 1588.34)(1220.3116, 1342.51)(610.185, 43769.18)(611.1857, 24975.33)(612.1834, 17824.51)(1236.3606, 637.65)(1237.3866, 315.06)	278611.7	44950.3	249146	44008.72	268749.3	43768.27	274301.4	46709.23	-0.30	0.297546	0.39767	0.72	0.03758	0.259604
COMPOUND_609_3241a10_150996	Rp	-	609.3241	10.15				(608.3173, 46736.48)(609.3205, 13628.42)(610.3221, 2951.0)(611.3247, 528.68)(1217.5789, 609.96)(1218.5544, 271.89)	260367.1	30197.06	277329.3	33104.51	212359.8	30189.63	219256.2	31683.22	-1.20	2.44E-06	1.11E-05	-0.23	0.400943	0.690408
COMPOUND_609_3244a10_274486	Rp	-	609.3244	10.27				(608.317, 56959.8)(609.3205, 15599.15)(610.3225, 3695.19)(611.3283, 681.33)(1217.6484, 2205.68)(1218.6515, 1224.21)(1219.6458, 562.67)	468845.8	99826.8	493242.5	102290.9	530295.9	121597.8	419730.8	170894.1	0.67	0.01972	0.03899	-1.09	0.001679	0.06002
COMPOUND_609_3245a10_26004	Rp	-	609.3245	10.26				(608.3172, 53156.0)(609.3205, 16249.66)(610.3238, 3477.43)(611.3286, 795.73)(1217.6481, 1809.81)(1218.6534, 1088.73)(1219.6484, 447.36)	376782.9	202576.7	313742.8	233595.5	543037.6	132567.1	486932.1	127651.3	0.91	0.002142	0.005295	-0.37	0.278131	0.585315
COMPOUND_609_3643a1_1230482	Hilic	+	609.3643	1.12				(632.3542, 1817.39)(633.3606, 516.25)(634.3699, 482.48)(610.372, 90615.27)(611.3755, 31457.0)(612.3783, 6317.9)(613.3806, 1441.04)	117806.3	95429.98	109312.2	95227.19	10013.44	5009.04	9880.698	3318.17	-1.61	1.65E-11	2.48E-11	0.19	0.402386	0.522902
COMPOUND_609_3651a10_2205	Rp	+	609.3651	10.22				(1219.7352, 666.47)(632.3553, 7566.27)(633.3578, 2692.49)(634.3706, 1553.55)(635.3722, 383.6)(627.4, 395.49)(648.3221, 1941.48)(610.3741, 174823.47)(611.3766, 55633.27)(612.3809, 11685.33)(613.3908, 2550.36)	119690.5	106413.9	113718.3	88966.95	#DIV/0!	#DIV/0!	10756	264.4579	-1.41	2.15E-11	4.74E-10	0.18	0.381105	0.683863
COMPOUND_609_5321a12_532632	Rp	-	609.5321	12.53				(722.512, 33771.75)(723.5155, 14914.84)(724.5175, 4208.95)(725.5192, 1177.34)(726.5177, 247.04)(608.5249, 9443.15)(609.5274, 3991.63)(610.5305, 1028.62)(611.5326, 157.72)(654.4984, 219.73)	344540.8	274171	372276.8	272347.1	465458.6	298572.7	467969.9	246150	0.56	0.034044	0.062893	-0.04	0.892709	0.953702
COMPOUND_609_533a13_662665	Rp	+	609.5330	13.66	Unknown	4	20 eV: 575.5030 (100), 313.2718 (75), 337.2734 (55), 610.5344 (20)	(1242.0602, 599.49)(1243.0623, 660.84)(610.5421, 50728.67)(611.5449, 20979.19)(612.5498, 5143.26)(613.552, 926.93)(614.5543, 121.97)	252139.1	153767.3	244546.9	113688.2	180376.4	89467.38	369196.5	184530.7	-0.53	0.05932	0.10276	1.28	0.000242	0.02094
COMPOUND_609_5347a13_729942	Rp	+	609.5347	13.73	DAG 34:2 (M+NH4)+	2	10 eV: 575.5072(100), 339.2883(32), 83.0836(5)	(1242.063, 289.79)(1243.0474, 297.51)(1244.0656, 235.35)(610.5419, 72649.63)(611.5448, 29355.51)(612.5486, 6996.27)(613.5523, 1133.4)(614.5486, 189.54)(1237.0634, 86.99)	304190.5	119718	314503.9	101526	221094.7	45820.91	334509.9	143043	-0.60	0.043968	0.078211	0.65	0.062893	0.307286
COMPOUND_610_2396a10_369285	Rp	+	610.2396	10.37				(1221.5262, 417.24)(1259.4862, 482.5)(1260.4958, 230.44)(633.2512, 1165.6)(1243.5286, 393.31)(628.2697, 1646.03)(629.2684, 740.94)(630.2722, 1048.16)(611.2446, 33038.63)(612.249, 16520.45)(613.2451, 12977.61)(1238.54, 392.79)(1239.5498, 276.77)(1240.534, 338.61)	130342.6	76550.33	145948.4	69560.31	212172.6	32145.41	210120.3	54355.52	1.01	0.000161	0.000483	-0.06	0.852486	0.937402
COMPOUND_610_7631a10_459865	Rp	+	610.7631	10.46				(1222.5187, 285.73)(1244.5511, 316.13)(611.771, 5232.23)(612.7714, 1793.54)(613.7541, 940.56)(614.7732, 229.49)(1239.544, 288.02)(1240.5497, 145.74)	49178.59	19341.67	50383.21	16166.65	45385.15	15350.48	40753	14410.97	-0.10	0.743484	0.814256	-0.17	0.636319	0.830719
COMPOUND_611_3175a7_1142673	Rp	+	611.3175	7.11				(612.3227, 7142.37)(613.3262, 2165.52)(614.3285, 604.29)(615.3245, 106.4)	25096.98	6207.525	23050.79	5055.134	22301.67	6769.304	25509.12	6165.591	-0.18	0.522442	0.621521	0.53	0.126437	0.423869
COMPOUND_611_55a13_479151	Rp	+	611.5500	13.48				(634.5411, 4162.13)(635.5457, 1821.21)(636.5526, 945.46)(637.5381, 58.45)(650.5127, 510.96)(651.5182, 289.08)(612.5575, 23324.82)(613.5607, 9874.28)(614.564, 2336.47)(615.5687, 358.21)(1241.1774, 1173.43)	46671.98	34766.54	40106.63	25653.56	47008.5	40384.4	78026.71	60776.94	0.13	0.572804	0.669064	0.79	0.00665	0.125854
COMPOUND_612_2534a10_616605	Rp	+	612.2534	10.62				(1225.554, 416.18)(1226.5427, 221.83)(1263.5457, 421.6)(1264.5151, 324.75)(635.2715, 1650.85)(1247.542, 272.27)(651.2497, 1194.01)(630.2883, 1964.42)(631.2843, 691.96)(632.2908, 2284.21)(633.2948, 837.46)(634.2865, 1560.27)(613.2609, 27930.26)(614.2659, 16380.36)(615.2611, 11391.36)(1242.5588, 386.81)	94950.1	55084.59	87200.73	43125.33	175315.5	35236.09	180819.9	54004.43	1.08	2.05E-05	7.62E-05	0.24	0.399352	0.690408
COMPOUND_612_2534a10_551098	Rp	+	612.2534	10.55				(1225.492, 55.8)(1226.4543, 333.31)(1263.5323, 159.22)(1264.5435, 119.33)(635.27, 722.28)(1247.5325, 271.53)(651.2444, 1031.85)(630.2837, 1680.72)(631.2913, 1152.45)(632.2846, 2124.33)(633.2964, 1087.25)(634.2857, 1503.45)(613.2603, 23278.77)(614.2657, 13846.76)(615.2615, 10309.82)(1242.556, 258.28)	70962.1	20384.24	64383.14	21707.86	50357.64	38846.09	63687.11	52384.08	-0.78	0.008871	0.019026	0.46	0.189905	0.505078
COMPOUND_612_2583a11_367208	Rp	+	612.2583	11.37				(1225.5187, 303.62)(635.2452, 275.84)(636.2652, 2106.98)(637.2477, 703.57)(651.2148, 1138.93)(652.235, 564.89)(613.2671, 30985.82)(614.2709, 13490.34)(615.2808, 14061.41)(616.2872, 6553.43)(617.283, 1963.26)	85816.62	72468.93	74610.08	52108.15	108816.7	80274.08	75523.72	50688.18	0.35	0.196398	0.2859	-0.38	0.222575	0.531222
COMPOUND_613_268a10_04665	Rp	-	613.2680	10.05				(648.2294, 699.52)(649.2365, 322.96)(650.218, 306.04)(1271.5405, 336.62)(1272.5247, 337.03)(1273.5544, 257.94)(612.2599, 21354.69)(613.2627, 8026.09)(614.2634, 2599.93)(615.2657, 529.8)(658.2625, 1325.08)(659.2544, 369.66)	53357.94	26034.67	57571.17	18980.32	44356.36	41463.07	38849.65	34548.61	-0.71	0.010498	0.022158	-0.36	0.268592	0.576066
COMPOUND_613_3033a10_530544	Rp	-	613.3033	10.53				(1261.5686, 315.37)(1262.585, 192.96)(1263.5707, 327.7)(726.2743, 964.17)(648.2887, 121.1)(649.3071, 27.95)(650.2978, 1458.43)(1271.6215, 302.04)(612.2965, 6447.93)(613.2976, 2402.67)(614.3006, 1043.36)(615.302, 212.61)(1225.6625, 283.38)(1226.6414, 216.43)(658.316, 862.72)(659.3339, 178.58)(1339.625, 238.25)	86195.24	20772.14	92018.05	22953.26	66012.5	4837.317	35808.43	21162.4	-1.30	2.36E-08	1.77E-07	0.13	0.575104	0.79068
COMPOUND_613_337a9_999344	Rp	-	613.3370	10.00				(726.3075, 663.11)(727.3008, 324.25)(728.3139, 182.76)(612.3301, 13796.83)(613.3333, 5083.97)(614.3353, 1384.52)(615.3435, 297.2)(616.3413, 51.6)	126299.1	32470.23	121001.7	29983.26	68188.86	26043.71	86605.02	47142.86	-1.50	2.52E-08	1.86E-07	0.57	0.042705	0.268638
COMPOUND_613_337a10_089828	Rp	-	613.3370	10.09	LPC 22:6	2	10 eV: 552.3082(100), 612.3302(60), 327.2296(4)	(612.3299, 113538.09)(613.333, 37105.1)(614.3365, 9154.5)(615.3403, 1848.84)(616.3384, 348.37)	715636.7	169731.9	684602.7	160333.5	418095.8	151933.9	490566.3	176178.8	-1.41	3.46E-08	2.46E-07	0.50	0.059209	0.30009
COMPOUND_614_365a8_670418	Rp	-	614.3650	8.67				(727.3231, 261.11)(728.3453, 86.84)(613.3587, 13683.46)(614.3624, 4974.51)(615.3653, 1233.44)(616.3673, 178.3)	29435.92	9606.122	31723.89	9803.905	47424.57	16786.16	44475.86	15956.85	1.16	5.39E-06	2.28E-05	-0.30	0.267562	0.575669
COMPOUND_614_4896a13_731386	Rp	+	614.4896	13.73				(615.4968, 23642.99)(616.5, 9726.36)(617.5041, 2220.3)(618.5126, 541.48)(619.5188, 89.57)	151775.1	56638.46	172911.6	50468.94	119943	21930.28	171130.1	75907.35	-0.54	0.071343	0.120709	0.37	0.292632	0.602481
COMPOUND_614_4914a12_8288	Rp	+	614.4914	12.83				(1230.0096, 122.0)(1267.9083, 167.38)(637.4797, 1133.22)(638.4826, 563.82)(639.4956, 317.32)(632.5247, 5967.46)(633.5279, 2560.81)(634.5339, 774.46)(635.5363, 147.44)(615.499, 1520.46)(616.503, 705.16)(617.5053, 266.91)(1246.9922, 79.6)(1248.01, 196.61)(1249.0062, 237.34)	39120.98	17394.06	39354.11	20066	44872	22495.48	50389.16	26045.37	0.31	0.291206	0.390819	0.18	0.611949	0.814985
COMPOUND_615_2609a10_645	Rp	+	615.2609	10.65				(1231.5591, 257.46)(1232.5311, 361.44)(1233.521, 294.74)(1269.5286, 277.44)(638.2508, 2342.37)(639.2714, 1656.81)(640.2488, 1935.11)(641.2438, 1080.19)(1253.5446, 305.56)(633.2901, 1300.85)(616.2652, 7884.78)(617.2653, 3367.54)(618.265, 2941.22)(1248.5765, 345.71)(1249.5757, 378.56)(1250.58, 344.51)(1251.5529, 348.89)(1252.5708, 203.33)	45346.15	19686.47	42782.5	11144	66027.92	15675.38	61369.24	16975.82	1.11	2.12E-05	7.83E-05	-0.38	0.194231	0.505089
COMPOUND_615_3522a10_325477	Rp	-	615.3522	10.33				(1265.6293, 365.53)(1266.6294, 207.55)(1267.635, 173.26)(614.3458, 5401.97)(615.3491, 1968.66)(616.3601, 4271.71)(617.3639, 1577.94)(618.3614, 574.0)(660.3399, 1121.97)	47498.85	17692.84	43321.37	15521.25	37598.5	16724.04	53570.21	22451.75	-0.49	0.065586	0.112662	0.90	0.004772	0.106402
COMPOUND_615_3525a10_224771	Rp	-	615.3525	10.22				(728.3233, 558.84)(729.328, 308.1)(730.3173, 265.43)(1275.7206, 208.42)(1276.7529, 258.87)(614.3457, 78841.57)(615.3487, 26522.83)(616.3512, 5220.03)(617.3534, 886.86)	126257.4	41220.03	118860	43986.26	56161.86	20298.06	72441.74	27038.59	-1.63	4.11E-11	7.76E-10	0.59	0.014888	0.181233
COMPOUND_615_4555a9_387792	Rp	+	615.4555	9.39				(1231.8833, 73.74)(616.4637, 22539.91)(617.4635, 8154.17)(618.4641, 1965.1)(619.4667, 362.27)(620.4636, 54.07)	205728.4	50353.44	180855.8	34793.79	223560.8	37459.28	220250.3	56076.05	0.27	0.339767	0.440504	0.54	0.11531	0.402603
COMPOUND_616_1458a8_724041	Rp	-	616.1458	8.72				(615.1394, 3242.37)(616.1427, 798.09)(617.1446, 550.1)	15530.67	7485.222	15166.71	1961.5	13885.86	5499.833	16309.9	8202.423	-0.24	0.372705	0.474735	-0.09	0.775665	0.905077
COMPOUND_616_1461a8_427299	Rp	-	616.1461	8.43				(615.1394, 6793.29)(616.1428, 1592.47)(617.1421, 306.03)	21370.62	2774.004	22354.26	2846.536	21927.14	2335.956	22033.84	3098.918	0.19	0.525851	0.624917	-0.33	0.366838	0.669574
COMPOUND_616_1481a8_839612	Rp	-	616.1481	8.84				(615.1394, 3226.85)(616.1432, 784.88)(617.1468, 243.18)	24000.69	5218.977	22621.7	4534.022	21026.86	6375.027	22823.33	6498.809	-0.24	0.41845	0.52284	-0.12	0.73583	0.888668

									1st trimester				3rd trimester				Time			Interaction		
									Control		Pre-eclampsia		Control		Pre-eclampsia							
Compound	Column	Ion_mode	Mass	Retention_	Identification	ID_level	MSMS_spectra	CompositeSpectrum	Mean	SD	Mean	SD	Mean	SD	Mean	SD	Value	raw p	FDR_p	Value	raw p	FDR_p
COMPOUND_616_5072a13_280005	Rp	+	616.5072	13.28				(1233.9976, 115.02)(639.496, 9753.19)(640.5002, 4182.71)(641.5049, 1285.82)(642.5165, 332.3)(655.4717, 1465.76)(656.4733, 744.11)(657.4734, 339.91)(634.5415, 24830.93)(635.5443, 10972.55)(636.5491, 2952.4)(637.5541, 768.47)(638.5648, 145.33)(617.5144, 9760.1)(618.5182, 4030.1)(619.5214, 1119.94)(620.5335, 235.55)(1251.0153, 272.64)(1252.0076, 230.05)(1253.0085, 220.79)	304441.9	92343.48	328823.2	115488.8	370085.4	97453.46	432656.2	198318.5	0.58	0.044702	0.079204	0.02	0.941529	0.973518
COMPOUND_616_508a11_758071	Rp	+	616.5080	11.76				(639.4986, 4977.09)(640.5022, 2445.49)(634.5185, 2767.64)(635.5206, 722.31)(636.5184, 101.95)(617.5155, 41302.39)(618.5186, 17569.72)(619.5209, 4201.34)(620.5221, 923.16)(621.5278, 118.31)	432150.9	165518.8	438101.8	142810.6	365962.3	221813.8	365944.1	271362.9	-1.13	1.74E-06	8.23E-06	-0.08	0.748432	0.891783
COMPOUND_617_3675a10_44986	Rp	-	617.3675	10.45				(730.3531, 1898.93)(731.3574, 862.29)(616.3612, 15519.22)(617.3646, 5243.48)(618.3677, 1376.1)(619.3662, 339.32)	58326.27	19397.13	56141.95	19808.57	32895.29	12736.08	43802.4	15801.94	-1.09	4.25E-05	0.000144	0.65	0.027568	0.226687
COMPOUND_618_0004a11_060905	Rp	+	618.0004	11.06				(1237.0005, 1237.83)(1238.0042, 1333.02)(1239.0023, 1021.7)(656.9589, 4356.45)(636.0188, 8756.74)(619.0079, 9996.46)(619.9996, 1833.82)(621.0094, 11220.2)	124019.7	71037.41	126655	103792.2	187063.9	54172.44	200110.9	101528.6	0.53	0.038606	0.070113	0.48	0.11389	0.400254
COMPOUND_618_2877a12_995973	Rp	+	618.2877	13.00				(1237.608, 108.08)(1275.5156, 206.39)(619.294, 7776.17)(620.2965, 3288.07)(621.2979, 980.25)(622.2963, 235.6)	175287.3	21008.4	175855.1	19860.32	167003.9	23602.36	160042.8	19003.12	-0.37	0.185629	0.273763	-0.37	0.261739	0.572854
COMPOUND_618_4758a0_9611075	Hilic	+	618.4758	0.96				(641.4638, 681.69)(642.4664, 290.95)(643.4788, 160.3)(619.4821, 7439.26)(620.4892, 3502.85)(621.4942, 1137.46)(622.4854, 155.73)	30059.97	8090.009	35605.18	12453.69	26079	8433.082	23316.41	10062.87	0.18	0.501775	0.602344	-0.54	0.094422	0.36381
COMPOUND_618_5231a13_938113	Rp	+	618.5231	13.94				(641.513, 30911.9)(642.5156, 13334.43)(643.5201, 3096.17)(644.5139, 183.15)(657.4869, 3990.95)(658.4922, 1746.25)(659.4905, 749.59)(660.4864, 199.39)(636.5579, 134530.4)(637.5607, 56367.32)(638.5638, 13572.48)(639.5674, 2541.39)(640.5714, 463.43)(619.5301, 3183.46)(620.5341, 1487.18)(621.5347, 403.27)(622.5234, 64.29)	753888.5	220873.6	795635.5	265939.3	883703.1	275422.9	1161166	551971.5	0.41	0.144864	0.221721	0.32	0.345379	0.651869
COMPOUND_620_2683a10_367031	Rp	+	620.2683	10.37				(1241.5142, 408.76)(1242.5237, 320.32)(1243.487, 265.34)(1279.5299, 386.19)(1280.533, 297.44)(1281.5137, 214.89)(1263.4935, 395.95)(1264.5034, 257.49)(1265.5208, 389.61)(1266.5024, 221.58)(621.2745, 13691.68)(622.2769, 6550.9)(623.2647, 2156.5)(1258.5159, 326.05)(1259.5247, 255.5)	88429.91	23863.41	81037.49	30052.92	83529.38	19975.26	84324.82	22374.65	0.06	0.822459	0.874081	0.35	0.297558	0.60748
COMPOUND_621_3597a10_613977	Rp	-	621.3597	10.61				(1277.7365, 289.38)(1278.7611, 487.46)(1279.7352, 287.27)(734.3265, 336.06)(735.3003, 489.22)(736.3246, 76.42)(620.3534, 11833.16)(621.3568, 3826.84)(622.3695, 4141.24)(623.3734, 1216.01)(624.3751, 376.37)(666.3449, 1324.37)(1241.7982, 282.88)(1242.7758, 315.59)(1243.7749, 312.39)	56587.44	13206.32	57582.13	15165.7	41731.5	22084.31	32651.08	16366.75	-1.10	3E-06	1.34E-05	-0.22	0.385961	0.687856
COMPOUND_621_403a0_7456386	Hilic	+	621.4030	0.75				(644.4261, 2599.18)(645.4287, 951.26)(646.4092, 38593.18)(639.4183, 2775.55)(640.4075, 397.2)(622.4087, 9585.14)(623.4115, 3473.71)(624.4112, 1102.1)(625.3978, 179.67)	235055.6	130282.5	280287.8	109813.5	90308	#DIV/0!	91103.57	23058.96	-1.34	8.43E-10	9.56E-09	0.10	0.648878	0.838541
COMPOUND_622_2847a7_1767826	Rp	+	622.2847	7.18				(645.2747, 8292.58)(646.279, 2531.67)(647.2815, 653.5)(648.2822, 107.62)(661.2495, 452.73)(662.2469, 33.09)(663.2276, 133.23)(640.3181, 68228.6)(641.3217, 21389.69)(642.3252, 4766.57)(643.3262, 1199.06)(644.3336, 241.14)(623.2921, 4468.19)(624.2956, 1458.68)(625.2978, 432.26)(626.3005, 110.92)	297453.7	111027.1	281251.6	91896.43	284639.3	95720.68	315061.8	94672.21	-0.12	0.679661	0.765299	0.48	0.177313	0.491826
COMPOUND_622_4341a10_102909	Rp	+	622.4341	10.10				(1245.8652, 570.74)(1246.8788, 213.55)(623.4406, 16143.13)(624.4427, 6157.47)(625.4456, 1300.74)(626.4404, 315.98)	52668.82	26360.03	51160.58	21962.83	21044.8	6730.747	22049.75	10032.58	-1.36	5.32E-09	4.65E-08	0.03	0.90757	0.958319
COMPOUND_623_2876a10_548581	Rp	+	623.2876	10.55				(1248.5631, 127.94)(1249.5856, 111.63)(1250.575, 43.39)(1285.5769, 298.3)(646.2728, 8375.48)(647.278, 3603.01)(648.2798, 1721.11)(1271.5627, 90.44)(1272.5361, 106.75)(1273.5872, 84.64)(662.2553, 1100.94)(663.2448, 766.88)(664.2367, 1970.0)(624.295, 12819.56)(625.2958, 4582.09)(626.2943, 6243.21)(627.2861, 2216.85)(628.2903, 2735.12)(629.2791, 2020.82)(1264.6, 319.05)	75601.74	20962.15	68536.49	19508.58	46504.07	31807	47026.25	33260.64	-1.21	2.4E-06	1.1E-05	0.23	0.393338	0.690408
COMPOUND_623_2887a10_6359	Rp	+	623.2887	10.64				(1247.5706, 383.77)(1248.5845, 472.64)(1249.5729, 361.97)(1250.5636, 325.46)(1285.5182, 398.36)(1286.5396, 201.13)(646.2749, 8897.1)(647.2798, 3486.25)(648.2784, 1693.36)(649.3024, 446.32)(1269.5564, 358.75)(1270.5623, 259.37)(1271.5568, 338.05)(1272.5668, 231.63)(1273.5892, 348.04)(662.2545, 985.39)(663.2435, 281.17)(664.2451, 3202.78)(624.2954, 20271.45)(625.298, 6192.76)(626.296, 11294.93)(627.2954, 3622.09)(628.2931, 4964.85)(629.2821, 3429.45)(630.2889, 2130.12)(1264.6609, 293.21)	190606.1	44615.42	170525	43056.67	200216.1	32322.7	195079.5	41975.29	0.21	0.451632	0.557302	0.40	0.222679	0.531222
COMPOUND_623_3375a10_477456	Rp	-	623.3375	10.48				(1281.6047, 260.41)(1282.5977, 360.38)(1283.6112, 202.99)(736.3106, 339.59)(1291.6512, 371.98)(1292.6674, 330.87)(1293.6497, 180.85)(622.3328, 7366.09)(623.3349, 2299.14)(624.3361, 692.26)(625.3588, 111.8)(1245.6626, 694.48)(1246.6626, 541.18)(1359.7134, 171.43)(1360.7284, 178.24)	92928.38	30467.63	107687.3	33583.93	59834	14797.3	63221.39	15964.72	-1.05	3.63E-05	0.000126	-0.11	0.703973	0.86442
COMPOUND_623_4426a11_3325205	Rp	+	623.4426	11.33				(1247.8627, 256.13)(1285.8021, 890.88)(1286.7812, 745.3)(646.4099, 4690.41)(1269.815, 859.01)(662.3947, 666.02)(624.4485, 18437.32)(625.451, 7513.4)(626.4519, 2836.28)(627.4377, 586.48)	39673.37	15900.32	39162.45	17648.35	72744.93	22709.57	59486.67	18373.58	1.48	8.29E-09	6.99E-08	-0.70	0.008724	0.14883
COMPOUND_625_2888a10_149883	Rp	-	625.2888	10.15				(1285.5111, 351.28)(1286.5234, 370.05)(1287.5106, 462.67)(738.2725, 2618.46)(739.263, 2163.88)(740.2583, 1376.19)(741.2423, 434.77)(660.259, 6272.41)(661.2602, 2593.57)(662.2612, 1690.14)(1295.53, 342.01)(1296.5284, 483.08)(624.284, 4198.9)(625.2885, 1203.54)(626.2758, 46010.46)(670.2868, 10793.37)(671.2787, 7816.81)(672.2791, 3237.99)(673.2793, 737.66)(1249.5472, 477.09)(1250.5562, 483.23)(1251.5492, 870.93)(1252.5511, 624.14)(1253.5605, 441.94)(1363.5264, 311.35)	382758.3	53955.02	400437.7	47866.11	301512.9	47066.99	317871.4	46428.58	-1.19	1.61E-06	7.77E-06	-0.12	0.639119	0.832444
COMPOUND_625_2907a10_2733755	Rp	-	625.2907	10.27				(738.2708, 2373.81)(739.2641, 1990.84)(740.2629, 972.03)(741.248, 275.81)(660.2575, 1725.2)(661.2489, 974.87)(662.2719, 2252.74)(663.2757, 1001.2)(664.2716, 520.13)(624.2849, 4045.94)(625.2885, 1192.14)(626.2757, 58168.11)(670.2875, 13809.35)(671.2796, 8873.32)(672.2826, 2906.54)(673.2822, 774.23)(1249.5272, 461.12)(1250.553, 292.55)(1251.5635, 651.79)(1252.5596, 555.63)(1253.5503, 482.12)	543402.7	209107.5	570115.9	231261.6	593024.2	193664.1	512079.4	216439.7	0.02	0.95011	0.968573	-0.40	0.262879	0.57476
COMPOUND_625_2947a0_9845451	Hilic	+	625.2947	0.98				(648.2804, 359.48)(649.2821, 262.72)(650.2963, 138.8)(626.3031, 2649.98)(627.3051, 892.86)(628.3083, 313.59)(629.3151, 71.41)	17903.49	5341.533	19042.26	5683.192	16359	4745.18	19517.21	4591.756	-0.01	0.978622	0.987833	-0.10	0.754928	0.895058
COMPOUND_626_2603a8_848905	Rp	-	626.2603	8.85				(625.2535, 7956.66)(626.2483, 3912.54)(627.2486, 1540.32)(628.2484, 396.7)(1365.4962, 90.7)(1366.5021, 99.16)	12856	2392.317	14768.08	3426.391	38675.29	15781.27	38254.11	14319.37	1.52	2.19E-14	3.52E-12	-0.11	0.531233	0.771657
COMPOUND_626_4645a10_639316	Rp	+	626.4645	10.64				(1291.8236, 574.97)(649.455, 5692.57)(1275.9225, 508.84)(627.472, 52182.29)(628.4753, 17612.02)(629.4777, 4068.56)(630.4755, 644.17)	133825.8	29938.89	123326	34722.49	38519	18876.75	37736.93	16514.02	-1.64	1.59E-15	5.97E-13	0.32	0.079604	0.339484
COMPOUND_627_325a10_657707	Rp	-	627.3250	10.66				(1289.6685, 348.39)(1290.6375, 261.28)(1291.6385, 231.13)(740.3273, 225.92)(741.3479, 711.97)(1299.7032, 298.05)(1300.6793, 218.36)(1301.6746, 126.91)(626.3188, 6876.4)(627.3212, 2170.89)(628.3177, 2316.23)(629.3181, 725.51)(630.3315, 246.29)(1253.6759, 210.03)(1254.6593, 158.1)(1255.6958, 319.01)(1367.6837, 129.68)(1368.6843, 255.34)	69570.68	15923.57	75360.54	18778	86345.5	13297.92	78684.39	18304.62	0.94	0.002081	0.005161	-0.74	0.038283	0.259981
COMPOUND_627_3255a10_59054	Rp	-	627.3255	10.59				(1289.6326, 374.99)(1290.6453, 371.15)(1291.6318, 246.07)(740.2788, 1029.2)(741.288, 384.69)(1299.6995, 216.91)(1300.6808, 376.33)(1301.6698, 218.9)(626.3188, 6267.44)(627.3216, 1743.45)(628.3175, 2225.18)(629.3211, 768.53)(630.3208, 232.25)(1253.6896, 388.66)(1254.679, 327.83)(1255.6493, 99.63)(1367.6439, 190.41)(1368.6385, 196.13)	42449.87	26022.73	41150.98	15136.95	42534.21	31004.74	58685.12	36252.96	-0.19	0.535695	0.634939	0.35	0.331975	0.636996

									1st trimester				3rd trimester									
									Control		Pre-eclampsia		Control		Pre-eclampsia		Time			Interaction		
Compound	Column	Ion_mode	Mass	Retention_	Identification	ID_level	MSMS_spectra	CompositeSpectrum	Mean	SD	Mean	SD	Mean	SD	Mean	SD	Value	raw p	FDR_p	Value	raw p	FDR_p
COMPOUND_628_1193a9_061208	Rp	-	628.1193	9.06				(741.0756, 535.97)(742.0801, 434.84)(663.078, 666.47)(664.0733, 310.32)(665.0739, 645.24)(627.113, 17516.63)(628.1161, 3663.05)(629.1099, 5745.91)(630.1129, 1106.07)(631.1073, 245.37)(1255.2314, 123.23)(1256.2126, 129.96)(673.0951, 1240.59)(674.1011, 669.37)(675.101, 1595.78)(676.1114, 736.28)(677.1053, 1415.31)	104839.5	10185.83	105468.4	9484.196	95097.14	9783.255	97357.61	10692.24	-0.94	0.001026	0.002671	0.15	0.639954	0.83305
COMPOUND_628_1201a9_296112	Rp	-	628.1201	9.30				(627.1122, 10215.31)(628.116, 2192.65)(629.1093, 3417.05)(630.112, 802.45)(631.116, 193.25)	59796.75	5347.917	59049.76	3675.786	57257.79	4365.68	57542.7	4458.322	-0.55	0.068847	0.117368	0.16	0.658919	0.842895
COMPOUND_628_2742a10_085511	Rp	+	628.2742	10.09				(1257.5173, 411.1)(1258.5002, 392.29)(1295.4783, 433.21)(1296.4727, 350.93)(651.2666, 652.73)(1279.5376, 309.69)(667.2543, 2794.7)(629.2793, 7724.77)(630.2814, 3056.8)(631.2608, 1274.27)(1274.5479, 249.02)	43885.03	15414.07	37484.61	13719.19	28966	5741.462	41093.76	12444.25	-0.91	0.00122	0.003116	1.02	0.002337	0.067469
COMPOUND_628_2844a10_154417	Rp	-	628.2844	10.15				(1291.5294, 505.37)(1292.534, 353.14)(1293.5198, 428.81)(1294.5378, 324.62)(1295.5477, 429.43)(741.2635, 677.87)(742.2748, 2169.05)(663.273, 999.86)(1301.5688, 483.01)(1302.5452, 477.33)(1303.5819, 244.49)(627.278, 13530.3)(628.2797, 4484.82)(629.2822, 845.65)(630.267, 43.36)(1255.5757, 392.42)(1256.5702, 185.13)(1257.5885, 748.0)(1258.5892, 598.36)(1259.583, 303.14)(673.2816, 973.07)(1369.5402, 171.17)(1370.4996, 289.44)	140526.3	31198.66	139356.3	33789.48	119782.1	48744.34	125590.2	46444.78	-0.94	0.00134	0.00341	0.26	0.449699	0.722351
COMPOUND_628_3806a8_966238	Rp	-	628.3806	8.97				(741.3396, 129.12)(627.3743, 6490.65)(628.3777, 2211.7)(629.3797, 588.0)(630.3833, 103.24)(1255.7858, 101.46)(1256.7642, 71.38)	32746.21	13045.64	35595.72	10594.07	46148.64	28750.53	35736.39	16169.85	0.68	0.01305	0.026937	-0.77	0.016504	0.189627
COMPOUND_629_4718a9_663932	Rp	+	629.4718	9.66				(1259.9899, 57.07)(1281.9622, 71.77)(630.4791, 23085.24)(631.4796, 8162.3)(632.4763, 1835.59)(633.4826, 243.04)	183354.9	49543.39	159464.1	30644.51	200937.7	34534.76	199228.7	51756.2	0.09	0.744074	0.814256	0.75	0.029366	0.231966
COMPOUND_631_3713a13_408046	Rp	+	631.3713	13.41				(1301.6873, 152.95)(1302.7052, 106.3)(654.3553, 303.28)(655.3431, 102.61)(656.341, 3513.37)(670.3662, 381.07)(671.3492, 268.96)(632.3781, 12193.22)(633.3813, 4621.72)(634.3797, 1619.1)(635.3686, 172.86)	98558.68	12603.17	98408.2	11595.75	104272.6	5836.654	100953.1	11996.31	0.51	0.096091	0.157266	-0.27	0.455779	0.726923
COMPOUND_632_2471a8_341707	Rp	-	632.2471	8.34				(631.2401, 11598.25)(632.2427, 4165.22)(633.2457, 994.91)(634.2522, 206.92)	20888.31	9860.171	17130.57	7113.454	35990	20679.33	24428.84	14602.39	1.13	2.75E-06	1.24E-05	-0.21	0.410399	0.694731
COMPOUND_632_2479a8_347881	Rp	+	632.2479	8.35				(655.2375, 316.66)(656.2305, 167.52)(633.2572, 33510.64)(634.2607, 12652.3)(635.2618, 3028.49)(636.2677, 656.4)	38035.77	22524.48	27604.33	15290.99	73320.86	42969.64	54255.84	35098.78	1.20	5.2E-06	2.2E-05	-0.17	0.542998	0.776977
COMPOUND_633_3245a10_110965	Rp	-	633.3245	10.11				(632.3169, 28624.94)(633.3206, 8679.49)(634.3227, 2141.8)(635.3257, 484.92)	199888.3	36178.61	219086.8	36925.69	191424	30346.28	172567.9	35375.25	-0.27	0.303023	0.403553	-0.90	0.005169	0.111932
COMPOUND_635_3401a10_379357	Rp	-	635.3401	10.38				(634.3331, 42354.2)(635.3364, 12960.27)(636.3404, 3069.79)(637.3422, 673.1)(638.358, 119.29)	468863.9	68930.54	503956.6	64113.87	391899.3	54369.81	401535.4	65331.28	-1.03	4.89E-05	0.000162	-0.25	0.362721	0.668267
COMPOUND_635_4167a0_7054705	Hilic	+	635.4167	0.71				(658.4204, 1309.92)(659.422, 595.47)(660.4244, 4081.37)(661.429, 1739.99)(662.438, 3069.49)(653.4643, 6418.05)(636.4241, 26944.14)(637.4276, 9809.72)(638.428, 2506.67)(639.4269, 666.61)	398546.7	176504.9	360210.8	166514.6	62280.08	63714.81	53780.33	55044.05	-1.47	4.84E-11	8.86E-10	-0.04	0.842739	0.93246
COMPOUND_635_4198a10_456654	Rp	+	635.4198	10.46				(658.4129, 3482.45)(659.4116, 1192.29)(660.4259, 21101.91)(636.4268, 11596.88)(637.4313, 4689.78)(638.4278, 4744.22)(639.436, 2630.07)	128238.3	56475.52	123203.1	52869.51	86714.25	48602.58	51772.62	33990.61	-1.15	1.34E-06	6.57E-06	0.04	0.890715	0.953702
COMPOUND_635_5493a13_090127	Rp	+	635.5493	13.09				(1310.075, 62.69)(1311.028, 205.86)(658.5401, 3345.45)(659.5434, 1637.98)(660.5566, 4315.58)(661.5602, 2022.09)(1294.0208, 128.26)(636.5566, 19465.95)(637.5601, 8610.1)(638.5632, 2053.83)(639.5696, 399.85)	88492.49	37213.17	95888.36	48601.7	96456.7	48014.79	136459.3	82056.96	0.16	0.522267	0.621521	0.61	0.04427	0.272788
COMPOUND_635_5499a13_93558	Rp	+	635.5499	13.94				(1294.0326, 158.86)(636.5579, 134530.4)(637.5607, 56367.32)(638.5638, 13572.48)(639.5674, 2541.39)(640.5714, 463.43)	495530	153698.3	512197.6	185960.9	574350.4	179786.4	771810.1	381324.3	0.37	0.19322	0.282004	0.43	0.208348	0.518913
COMPOUND_635_5502a13_155785	Rp	+	635.5502	13.16				(1310.0504, 58.05)(658.5411, 17086.98)(659.5446, 7835.27)(660.5501, 2694.92)(661.5565, 742.51)(1294.0977, 188.48)(636.5559, 8712.93)(637.5594, 3581.18)(638.5635, 1088.14)(639.5649, 244.3)	118274.5	55419.45	112092.8	63210.2	145161.9	43649.45	191117.7	98042.2	0.45	0.089549	0.147848	0.59	0.064589	0.307286
COMPOUND_635_7758a10_339983	Rp	+	635.7758	10.34				(1272.5817, 264.16)(1310.4886, 254.85)(658.7882, 391.86)(659.7938, 1161.96)(636.7845, 10996.16)(637.7848, 1262.84)(638.7684, 1431.77)	28083.3	17454.17	27922.48	15211.26	26559.78	5042.852	25395.26	6912.853	-0.11	0.69545	0.77802	-0.02	0.945212	0.974642
COMPOUND_636_2998a10_393211	Rp	+	636.2998	10.39				(1273.6082, 626.17)(1274.6196, 350.47)(1275.5933, 194.65)(1311.5023, 283.39)(1312.5107, 324.99)(659.2877, 11863.67)(660.2928, 5112.41)(661.2859, 1827.87)(662.2713, 411.81)(1295.581, 1953.01)(1296.5897, 1259.38)(1297.6029, 770.91)(1298.576, 330.79)(654.3339, 32672.72)(655.3374, 12720.37)(656.3404, 2818.6)(657.342, 724.46)(637.3073, 56460.81)(638.3098, 19450.78)(639.3132, 4445.51)(640.3166, 1051.01)(641.3095, 193.59)(1290.6824, 416.47)(1291.725, 607.42)	234195.2	85078.72	188720.4	80559.55	280929.4	91292.97	291158.2	109048.4	0.54	0.054443	0.094676	0.50	0.130311	0.425921
COMPOUND_637_2869a10_376145	Rp	-	637.2869	10.38				(1309.5222, 353.03)(1310.5295, 339.37)(1311.5425, 376.11)(1319.5529, 326.96)(1320.5411, 394.42)(1321.558, 275.71)(636.2827, 1346.52)(637.2767, 593.79)(638.276, 19992.51)(1273.5651, 970.56)(1274.5679, 658.55)(1275.5712, 663.04)(1276.5886, 363.28)(1277.5833, 583.69)(1278.5905, 472.47)(1279.575, 460.71)(1387.5345, 285.51)(1388.5399, 203.71)(1389.5358, 294.57)	190867.8	22154.82	196504.3	22812.54	150242.1	16370.88	152515.4	23611.63	-1.31	7.49E-08	4.88E-07	-0.08	0.738034	0.888668
COMPOUND_637_3555a10_62855	Rp	-	637.3555	10.63				(1309.6761, 135.08)(1310.6665, 50.38)(1311.6763, 204.11)(1312.672, 239.73)(1313.6458, 520.03)(750.3196, 562.77)(751.3157, 467.83)(1319.7122, 227.79)(1320.7106, 274.44)(1321.7196, 250.96)(636.3485, 54855.3)(637.3517, 17231.3)(638.3528, 3973.48)(639.357, 818.04)(1273.7301, 303.93)(1274.7046, 122.69)(1275.7378, 337.08)(1276.7511, 327.96)(1277.764, 311.61)(1387.7448, 183.51)(1388.7648, 152.72)(1389.756, 214.31)	523589.2	173499.2	545538.2	207256.1	299722.6	200614.5	333546.7	180303.7	-1.14	1.47E-05	5.69E-05	-0.13	0.655605	0.841269
COMPOUND_637_3559a10_605653	Rp	-	637.3559	10.61				(1309.7144, 145.32)(1310.7249, 318.56)(1311.7292, 334.87)(1312.721, 295.67)(1313.726, 159.28)(750.3378, 619.93)(751.3408, 456.73)(1319.7058, 319.98)(1320.7153, 288.96)(1321.7155, 268.63)(636.3485, 52306.32)(637.3516, 16579.47)(638.3533, 3723.69)(639.3527, 685.77)(1273.7311, 333.73)(1274.758, 233.56)(1275.7677, 421.38)(1276.7664, 314.44)(1277.7595, 427.24)(1387.7183, 175.27)(1388.7351, 215.06)(1389.7449, 186.96)	314202.2	235039.5	306105.7	241236.2	307613.9	214418.4	354739.3	185798.9	-0.52	0.086909	0.1437	0.29	0.422303	0.702743
COMPOUND_637_396a1_0536169	Hilic	+	637.3960	1.05				(660.3859, 3700.71)(661.3984, 1701.63)(662.4031, 13851.36)(655.4257, 165.85)(638.4033, 60589.44)(639.4063, 20437.56)(640.4093, 4889.68)(641.4138, 896.26)	86499.47	72092.99	83276.76	62821.67	6375	35.35534	6451.75	531.3325	-1.46	1.73E-13	1.26E-11	-0.03	0.867294	0.9447
COMPOUND_637_5662a13_674904	Rp	+	637.5662	13.67				(1276.1637, 72.24)(660.5573, 4831.94)(661.5595, 2276.06)(662.5716, 4316.06)(638.5721, 53357.39)(639.5754, 22665.21)(640.5791, 5183.55)(641.5816, 968.34)(642.5857, 181.92)	196039.5	87855.06	209771.2	108764.2	171400.4	73710.97	314168.4	173952.2	-0.25	0.341027	0.441883	1.02	0.00202	0.065137
COMPOUND_637_5663a13_736985	Rp	+	637.5663	13.74				(1276.0898, 53.34)(660.5572, 6084.68)(661.5594, 3030.01)(662.5676, 1732.09)(638.5725, 30207.86)(639.5758, 13138.52)(640.5788, 3244.9)(641.5842, 659.19)(642.5745, 106.75)	256084.4	120949.6	249141	115546.5	191151.6	53002.55	310526.5	169895	-0.49	0.092439	0.152285	0.84	0.015907	0.188539
COMPOUND_638_1482a9_060878	Rp	-	638.1482	9.06				(673.0967, 1499.96)(637.1416, 67667.4)(638.1445, 14993.95)(639.1476, 1986.77)(640.162, 426.76)	303850.2	28681.78	306722.4	20998.68	272919.1	24369.26	286254.2	25046.94	-1.21	3.83E-05	0.000131	0.40	0.2278	0.535498
COMPOUND_638_1483a9_407069	Rp	-	638.1483	9.41				(1311.2484, 280.36)(1312.2366, 83.19)(637.1414, 27378.18)(638.1448, 5804.33)(639.1473, 862.19)(640.1438, 199.13)(683.1476, 374.74)(684.1413, 60.25)(685.1299, 474.26)	159407.5	13912.72	160929.6	11670.22	157919	10169.64	159794.7	12276.94	-0.09	0.773046	0.833547	-0.02	0.960606	0.981885
COMPOUND_638_1487a9_295998	Rp	-	638.1487	9.30				(751.1409, 523.29)(752.1509, 451.18)(753.122, 419.59)(637.1412, 43052.91)(638.1446, 9509.07)(639.1466, 1228.45)(640.1416, 275.07)(1275.3048, 176.54)	193931.4	16123.94	197500	13133.78	190425.1	9363.873	192251	11468.15	-0.18	0.542178	0.640471	-0.23	0.517242	0.763322
COMPOUND_639_3105a1_246787	Hilic	+	639.3105	1.25				(662.3098, 432.46)(657.3357, 245.66)(640.3177, 6285.35)(641.322, 2096.04)(642.3233, 606.87)(643.3277, 70.26)	23643.32	5349.167	26045.28	5877.383	23939	7632.406	25959.15	8164.793	0.29	0.316239	0.418431	-0.04	0.900473	0.955487

									1st trimester				3rd trimester									
									Control		Pre-eclampsia		Control		Pre-eclampsia		Time			Interaction		
Compound	Column	Ion_mode	Mass	Retention_	Identification	ID_level	MSMS_spectra	CompositeSpectrum	Mean	SD	Mean	SD	Mean	SD	Mean	SD	Value	raw p	FDR_p	Value	raw p	FDR_p
COMPOUND_639_3168a10_649515	Rp	-	639.3168	10.65				(674.2783, 1165.32)(675.2682, 506.29)(752.3089, 195.5)(753.2745, 300.51)(754.2992, 218.59)(755.2917, 448.72)(1323.6141, 266.29)(1324.6093, 247.25)(638.3088, 1961.8)(639.3082, 743.44)(640.2918, 16453.08)(1277.5972, 747.73)(1278.5991, 412.23)(1279.6025, 362.07)(684.34, 1537.61)	128032.9	14837.43	128656.9	18242.15	113490.2	12058.12	105329.6	24339.23	-0.88	0.00098	0.002559	-0.21	0.491039	0.748114
COMPOUND_639_3305a12_429419	Rp	-	639.3305	12.43				(674.3081, 221.38)(675.3059, 166.42)(676.3095, 183.0)(677.3075, 140.57)(678.3092, 202.94)(638.3244, 633.43)(639.3219, 266.4)(640.3185, 7169.31)(684.311, 836.55)(685.3094, 409.62)(686.2995, 448.88)	32866.83	4924.137	29726.48	4344.908	33720.21	4898.628	33211.16	5290.533	0.21	0.465334	0.569838	0.47	0.183022	0.496585
COMPOUND_640_2871a10_397091	Rp	-	640.2871	10.40				(1315.5411, 416.82)(1316.5573, 447.18)(1317.5668, 341.81)(1318.5723, 365.03)(1319.5656, 295.06)(753.2747, 884.34)(754.2745, 522.8)(755.2686, 594.42)(1325.5577, 272.32)(1326.5422, 309.85)(1327.5695, 402.58)(1328.559, 296.59)(1329.5686, 140.29)(1330.5247, 291.83)(1331.519, 271.44)(639.2785, 6808.39)(640.2862, 8263.77)(641.2892, 3155.52)(642.286, 585.35)(685.2623, 3109.64)(686.2653, 2561.36)(687.2658, 1188.93)(1279.586, 328.24)(1280.5596, 414.05)(1281.536, 271.57)(1393.558, 142.68)(1394.5698, 124.39)(1395.5388, 210.0)(1396.5214, 189.15)(1397.4978, 313.6)	179087.7	80244.28	180806.4	79441.95	151951.8	36402.22	142909.5	39256.87	0.14	0.579626	0.67598	-0.18	0.548635	0.77853
COMPOUND_640_4301a10_924666	Rp	-	640.4301	10.92				(675.3858, 446.58)(676.4041, 1053.33)(677.3959, 657.17)(753.4051, 1279.58)(1325.8892, 259.53)(1326.897, 368.79)(1327.8778, 383.41)(1328.8894, 382.56)(1329.9099, 320.09)(639.4149, 3703.47)(640.4159, 1411.61)(641.4187, 2175.47)(642.4172, 1181.32)(643.4178, 844.47)(1279.8237, 720.83)(1280.815, 987.95)(1281.8224, 1040.12)(685.4213, 8478.56)(686.4178, 7933.86)(1393.896, 264.03)(1394.859, 346.7)	56413.19	19815.21	64929.95	23512.69	100060.7	41699.35	121862.7	35233.18	0.39	0.079284	0.132426	0.61	0.023655	0.219225
COMPOUND_641_2616a10_1700945	Rp	-	641.2616	10.17				(1317.4768, 79.29)(1318.4911, 226.01)(1319.4976, 104.37)(754.2359, 1220.67)(755.2359, 645.04)(756.2381, 2465.33)(757.2377, 1512.47)(758.2421, 1378.77)(759.2452, 672.53)(760.2526, 1044.63)(676.2324, 1220.89)(677.2316, 613.04)(678.2274, 1594.58)(679.2198, 818.35)(680.2405, 1671.86)(1327.4847, 240.31)(1328.4899, 373.99)(640.2547, 10661.26)(641.2576, 3497.53)(642.2597, 1483.22)(643.2609, 388.93)(1281.5015, 459.53)(1282.5096, 368.98)(1283.5144, 406.09)(686.258, 7324.82)(687.2613, 2401.63)(688.2604, 8799.33)(689.2637, 2626.46)(690.2588, 3854.08)(691.2589, 1376.89)(692.2611, 1194.49)	223508.9	75566.47	211286.2	92490.52	205610.4	50599.3	205271.7	56836.37	-0.40	0.186667	0.274755	0.30	0.405977	0.691573
COMPOUND_641_2624a10_211116	Rp	-	641.2624	10.21				(1317.5281, 336.94)(1318.5327, 236.73)(1319.5255, 393.26)(754.242, 1072.24)(755.2449, 590.38)(756.2402, 2588.25)(757.2384, 1559.57)(758.2442, 1423.14)(759.2417, 604.81)(760.2554, 1244.43)(676.2493, 1591.71)(677.2348, 459.28)(678.232, 1083.37)(679.2423, 461.02)(680.2419, 1754.22)(1327.4965, 279.9)(1328.5145, 241.91)(640.2549, 13408.94)(641.2583, 4853.79)(642.2584, 1594.69)(643.2622, 399.22)(1281.505, 348.33)(1282.5073, 348.44)(1283.5107, 304.63)(686.2589, 5714.86)(687.2605, 1940.42)(688.2589, 9601.57)(689.2619, 3328.98)(690.2585, 3551.54)(691.2615, 1235.43)(692.2653, 1131.23)	170560.9	96379.98	165740.1	95713.9	301790	139478.9	264060.4	137604.2	1.00	0.000573	0.001576	-0.19	0.571159	0.789098
COMPOUND_641_2959a10_507441	Rp	-	641.2959	10.51				(1317.5942, 160.67)(1318.5979, 141.14)(1319.6156, 169.84)(1320.5879, 214.86)(1321.5869, 136.97)(754.2249, 1254.21)(755.2412, 710.4)(676.288, 913.05)(677.2886, 485.95)(678.3041, 1159.13)(1327.5646, 259.25)(1328.5602, 319.05)(1329.5847, 275.61)(1330.5415, 255.95)(640.2908, 10045.78)(641.2929, 3849.65)(642.2935, 1613.45)(643.2986, 388.42)(686.2637, 580.31)(687.2691, 275.84)(688.2805, 910.91)(1281.5895, 352.53)(1282.5938, 313.02)(1283.594, 222.94)(1395.6398, 110.02)(1396.6158, 131.53)(1397.601, 116.49)(1398.6307, 130.53)(1399.6588, 84.89)	97292.73	49993.92	101639.9	48662.11	48736.45	26596.08	38239.06	16511.71	-1.20	7.45E-07	3.85E-06	-0.18	0.501774	0.75374
COMPOUND_641_2971a10_514763	Rp	-	641.2971	10.51				(1317.6144, 131.47)(1318.5908, 133.8)(1319.6039, 98.29)(1320.5817, 85.16)(1321.5807, 85.02)(754.2331, 1097.73)(755.2379, 777.21)(676.2909, 928.33)(677.2863, 376.45)(678.3034, 1126.32)(1327.5696, 316.78)(1328.5768, 347.66)(1329.5891, 201.56)(1330.5928, 167.92)(640.2912, 10717.12)(641.2938, 4304.36)(642.295, 1523.63)(643.2983, 374.4)(644.2966, 68.31)(1281.603, 277.83)(1282.5929, 210.66)(1283.591, 306.0)(686.2634, 499.07)(687.2735, 232.67)(688.2764, 981.87)(1395.6431, 161.71)(1396.5975, 175.91)(1397.574, 144.81)(1398.5789, 134.38)(1399.6115, 81.42)	82451.75	41986.11	88333.84	40811.03	40563.57	20776.23	38721.19	18622.17	-1.07	1.59E-05	6.09E-05	-0.28	0.299479	0.607594
COMPOUND_641_2985a10_65236	Rp	-	641.2985	10.65				(1318.581, 186.02)(1319.5663, 95.53)(1320.586, 138.25)(1321.5797, 182.5)(676.2846, 977.07)(677.2818, 406.01)(678.2734, 579.93)(1327.6132, 193.58)(1328.6132, 128.49)(1329.6083, 247.86)(1330.6298, 182.33)(1331.6047, 371.59)(640.2911, 12120.66)(641.2937, 4674.14)(642.2954, 1713.63)(643.296, 463.36)(644.3116, 71.99)(1281.6053, 244.6)(1282.6409, 227.44)(686.2799, 286.85)(687.2816, 192.93)(688.2873, 944.78)(1395.612, 192.21)(1396.6364, 152.66)(1397.5847, 198.06)	170624.7	16197.22	174154.5	21649.01	151231.3	15922.82	147209.1	21394.05	-0.87	0.001096	0.002831	-0.30	0.323905	0.630999
COMPOUND_641_3025a10_552493	Rp	-	641.3025	10.55				(1317.5886, 125.4)(1318.57, 143.55)(1319.5796, 159.71)(1320.5814, 215.21)(1321.58, 200.6)(754.2364, 1046.95)(755.2392, 692.82)(676.2886, 1126.28)(677.2878, 541.1)(678.3054, 1748.95)(1327.626, 169.21)(1328.6381, 234.33)(1329.6229, 235.76)(1330.6213, 55.45)(640.2919, 12652.96)(641.2939, 5378.59)(642.2949, 1786.56)(643.2974, 479.19)(1281.6295, 222.4)(1282.6132, 302.0)(1283.6321, 167.11)(686.281, 586.75)(687.2625, 352.82)(688.2838, 945.86)(1395.6886, 133.89)(1396.6598, 160.25)(1397.6377, 176.6)(1398.6548, 105.81)(1399.6912, 111.44)	71898.92	18399.41	72467.58	15655.96	40588.36	7059.905	48524.8	37150.78	-1.44	4.04E-08	2.83E-07	0.21	0.449705	0.722351
COMPOUND_641_5245a11_608373	Rp	+	641.5245	11.61				(1321.9551, 127.01)(1322.9895, 37.6)(680.4832, 1402.69)(681.4916, 1949.68)(682.4882, 1484.1)(659.5804, 912.05)(642.5309, 10921.14)(643.5352, 4563.61)(644.5394, 1444.48)(645.5365, 320.1)	74326.11	56638.56	75521.24	41286.43	40785.33	15195.65	40406.13	15236.24	-1.07	2.08E-06	9.6E-06	-0.05	0.831972	0.926681
COMPOUND_642_1857a10_314848	Rp	+	642.1857	10.31				(1285.3422, 268.43)(681.1549, 5486.32)(682.146, 1540.01)(683.1473, 721.61)(643.1901, 11027.72)(644.1964, 3296.93)(645.1871, 6289.26)(646.1907, 2519.11)(647.1949, 2396.88)(648.1969, 913.29)(649.1864, 424.61)	43457.6	11826.17	38763.64	13164.28	60583.14	13383.2	55969.72	13537.55	1.10	3.72E-05	0.000128	0.15	0.604101	0.810123
COMPOUND_642_5223a13_782367	Rp	+	642.5223	13.78				(665.5123, 8118.39)(666.5155, 3687.54)(667.5199, 988.71)(668.5178, 243.53)(681.4879, 848.15)(682.4949, 453.99)(683.4863, 310.16)(660.557, 19341.23)(661.5599, 8632.42)(662.5641, 2470.33)(663.5668, 622.67)(643.5313, 3990.37)(644.5374, 1991.74)(645.5373, 465.71)(646.5478, 97.43)	103122.7	42610.12	110781.3	49833.5	133653.1	67954.86	183684.1	81932.55	0.58	0.021627	0.042167	0.47	0.10824	0.391238
COMPOUND_643_4869a9_914848	Rp	+	643.4869	9.91				(1309.9836, 69.35)(644.495, 12158.0)(645.4961, 4533.27)	81806.54	22779.34	70779.43	13436.16	89901.08	16251.57	89148.49	23278.34	0.27	0.323206	0.424408	0.68	0.043589	0.271169
COMPOUND_644_2833a11_357574	Rp	-	644.2833	11.36				(757.276, 323.9)(758.2833, 174.29)(679.2473, 709.0)(680.2498, 358.99)(681.2478, 452.56)(682.2503, 261.6)(643.2767, 9632.56)(644.2791, 4106.78)(645.287, 1957.07)(646.2943, 501.81)(689.2505, 269.29)(690.2406, 264.56)	30692.23	28714.54	30835	22075.85	61620.08	46047.46	38144.1	29467.11	0.72	0.006089	0.01351	-0.34	0.252974	0.565176
COMPOUND_644_2858a11_367559	Rp	+	644.2858	11.37				(1289.6403, 552.6)(667.2802, 5109.19)(668.2848, 2946.52)(669.2919, 1374.09)(670.2828, 357.79)(662.3138, 2959.88)(663.3001, 1861.84)(664.3294, 1863.17)(683.2395, 910.62)(645.293, 22696.81)(646.2961, 10340.67)(647.2983, 3432.83)(648.2994, 843.86)	128967.8	83462.02	110071.8	67535.66	164780.2	89381.31	130048.1	69293.41	0.41	0.075463	0.126728	-0.29	0.282602	0.591468
COMPOUND_644_4898a0_916681	Hilic	+	644.4898	0.92				(667.4774, 275.8)(668.4738, 169.66)(669.4934, 1303.89)(670.4961, 577.56)(645.4968, 13008.84)(646.4998, 5411.64)(647.5123, 38549.45)(648.5154, 15176.74)	137626.1	37841.29	155833.2	45907.22	141992.3	36027.58	132602	41616.64	0.05	0.848512	0.894514	-0.69	0.024655	0.219303

Compound	Column	Ion_mode	Mass	Retention_	Identification	ID_level	MSMS_spectra	CompositeSpectrum	1st trimester				3rd trimester				Time			Interaction		
									Control		Pre-eclampsia		Control		Pre-eclampsia							
									Mean	SD	Mean	SD	Mean	SD	Mean	SD	Value	raw p	FDR_p	Value	raw p	FDR_p
COMPOUND_645_4017a0_71836036	Hilic	+	645.4017	0.72				(668.3963, 1161.42)(669.3981, 330.98)(670.4032, 461.46)(671.4109, 239.84)(646.4085, 46263.61)(647.412, 16908.5)(648.4204, 8835.16)(649.4239, 2379.76)	200577.4	96596.24	185253.3	88853.72	63681	29637.6	62031.67	22442.01	-1.45	1.08E-11	2.68E-10	0.15	0.472005	0.734155
COMPOUND_645_4017a10_47807	Rp	+	645.4017	10.48				(668.3923, 11369.09)(669.3977, 4456.17)(670.4053, 3648.86)(671.4142, 990.85)(684.3875, 3241.37)(663.4175, 6865.61)(646.4101, 33691.85)(647.414, 12558.78)(648.4233, 12743.35)(649.4326, 4831.2)	338839.2	159438.7	261931.5	134138.6	96537.67	17355.17	105105.8	46400.86	-1.35	2.15E-10	3.02E-09	0.19	0.378565	0.681478
COMPOUND_645_4052a10_856795	Rp	+	645.4052	10.86				(1291.7972, 1387.12)(1329.7812, 734.99)(1330.7706, 914.83)(1331.7892, 1056.78)(1332.7675, 766.12)(1333.7627, 776.58)(668.4026, 384.72)(669.4118, 4616.3)(670.419, 2532.66)(1313.7673, 668.63)(1314.7957, 973.52)(1315.7802, 1002.01)(684.3759, 5320.9)(685.3759, 2904.0)(686.3776, 6869.89)(687.3822, 3335.2)(646.4106, 19650.35)(647.4132, 7526.24)(648.4304, 5921.49)(1308.8217, 612.09)(1309.7711, 705.48)	#DIV/0!	#DIV/0!	#DIV/0!	#DIV/0!	#DIV/0!	#DIV/0!	#DIV/0!	#DIV/0!	-0.03	0.900188	0.934635	0.01	0.964075	0.981972
COMPOUND_646_4198a10_843989	Rp	-	646.4198	10.84				(1327.7659, 736.31)(1328.7782, 624.78)(1329.7699, 927.27)(759.3795, 1039.52)(1337.8843, 266.2)(1338.8657, 295.17)(1339.8442, 263.51)(645.4146, 18097.91)(646.4157, 5810.35)(1291.8107, 431.71)(1292.7845, 447.2)(691.4596, 1177.62)	27029.44	7165.596	30191.68	9951.744	26618.14	4583.083	31398.36	6666.988	-0.67	0.013021	0.026902	0.67	0.032451	0.244873
COMPOUND_646_4537a11_671692	Rp	+	646.4537	11.67				(669.4754, 968.87)(670.4738, 666.56)(685.4322, 1467.28)(686.4351, 774.33)(687.4207, 365.68)(647.4595, 69544.77)(648.4626, 32330.21)(649.4659, 7770.28)(650.468, 1798.96)(651.4511, 177.01)	905607	299841.2	663036	216569.2	1071931	404482.7	1112322	378620.7	0.39	0.137464	0.212908	0.88	0.006026	0.121164
COMPOUND_646_5049a0_916627	Hilic	+	646.5049	0.92				(669.4948, 1962.23)(670.5002, 847.47)(671.5103, 850.2)(664.5323, 393.69)(647.5123, 38549.45)(648.5154, 15176.74)(649.5194, 3698.87)(650.5216, 747.32)(651.5323, 133.78)	135478.7	37454.99	153252.4	46023.56	137897.1	35659.5	128681.1	40851.56	0.00	0.996714	0.999377	-0.68	0.027559	0.226687
COMPOUND_646_5058a11_263703	Rp	+	646.5058	11.26	SM d30:1	2	20 eV: 184.0743(100), 647.5073(10), 89.0603(9)	(669.4752, 5891.05)(670.4743, 4665.07)(671.4798, 8959.94)(647.5138, 29694.6)(648.516, 11731.7)(649.5176, 2797.0)(650.5101, 569.4)	198070.5	52117.68	189297.8	58450.14	181041.3	41094.43	188588.1	44991.26	-0.40	0.13698	0.212305	0.26	0.402158	0.691103
COMPOUND_649_4368a10_522202	Rp	+	649.4368	10.52				(672.4246, 12533.83)(673.4295, 5108.53)(674.4375, 4175.28)(650.4397, 13131.24)(651.4436, 5014.43)(652.4255, 2721.24)(653.4266, 356.35)	300812.4	133891.1	303184.9	126017.9	72985.5	62411.38	37668.67	18657.21	-0.76	0.00039	0.001104	-0.31	0.203442	0.5149
COMPOUND_651_1819a8_603599	Rp	+	651.1819	8.60				(652.1872, 6161.9)(653.1902, 1432.59)(654.1854, 256.79)(1320.4149, 132.9)	18185.08	3527.807	17746.24	3561.262	20095	3732.392	18778.16	3497.315	0.26	0.366875	0.469434	-0.04	0.915146	0.961085
COMPOUND_651_2815a10_040113	Rp	-	651.2815	10.04				(1337.5013, 292.67)(1338.4937, 357.03)(1339.5161, 307.57)(764.2627, 404.04)(765.2571, 154.41)(766.2608, 404.86)(767.2621, 73.88)(686.259, 5546.99)(687.261, 1864.19)(688.2589, 9183.69)(1347.5311, 315.93)(1348.5214, 248.71)(1349.5084, 237.38)(650.2757, 20738.87)(651.2777, 8872.2)(652.2785, 2637.3)(653.2798, 721.57)(1301.5693, 426.41)(1302.5452, 409.14)(1303.5834, 221.61)(696.2733, 2616.36)(697.2719, 840.15)(1415.4854, 232.26)(1416.4403, 207.0)	169796.4	128308.9	216103	137439.8	193617.9	117677.1	162650.6	112602.1	-0.11	0.710548	0.788253	-0.68	0.053067	0.287276
COMPOUND_651_3547a12_427157	Rp	-	651.3547	12.43				(650.3476, 10792.92)(651.351, 4511.67)(652.3538, 1206.89)(653.3561, 296.56)	87915.15	6931.521	83835.98	5841.067	88983.36	7621.055	88722.21	6587.239	0.19	0.498777	0.599888	0.54	0.120231	0.410243
COMPOUND_652_2754a10_083093	Rp	+	652.2754	10.08				(1343.4572, 219.49)(1344.4543, 187.2)(1327.4915, 185.34)(691.2336, 1074.97)(692.2273, 781.48)(693.2295, 181.69)(653.2791, 6033.22)(654.2843, 2154.09)(655.2765, 889.79)(656.2906, 223.98)	22438.67	6555.603	19317.03	3833.98	18517.38	5381.784	22918.44	6006.372	-0.73	0.00887	0.019026	0.73	0.027385	0.226687
COMPOUND_653_3194a10_597453	Rp	-	653.3194	10.60				(1341.6101, 217.83)(1342.5958, 209.56)(1343.5715, 229.71)(688.2804, 1141.39)(689.2767, 828.14)(690.2747, 684.41)(766.3045, 1786.16)(767.2983, 1423.57)(768.2949, 997.49)(769.2958, 194.93)(1351.73, 259.09)(1352.7029, 217.26)(652.3145, 3725.2)(653.3211, 1123.44)(654.3072, 39198.9)(698.3172, 5284.36)(699.309, 4365.81)(700.3073, 1698.62)(701.312, 504.38)(1305.6095, 320.74)(1306.6147, 268.74)(1307.6104, 405.23)(1419.5688, 228.61)	262828.9	148108.8	233004	144706.9	255308	170496.9	237250.8	139679	-0.64	0.030382	0.056969	0.19	0.580069	0.794113
COMPOUND_653_3208a10_647613	Rp	-	653.3208	10.65				(766.3045, 1786.16)(767.2983, 1423.57)(768.2949, 997.49)(769.2958, 194.93)(688.2799, 1043.79)(689.2776, 652.06)(690.2876, 859.77)(1351.6241, 217.33)(1352.6073, 260.44)(652.3165, 3829.95)(653.3181, 1260.33)(654.3071, 46651.78)(655.3097, 18119.99)(698.3184, 6563.41)(699.3097, 5363.67)(700.31, 2097.91)(701.3119, 592.55)(1305.6178, 246.77)(1306.6222, 176.37)(1307.6351, 436.43)(1308.6284, 253.12)	474389.9	48467.69	465951.3	97305.23	369551.6	97956.62	383780.5	59610.67	-1.21	5.12E-06	2.17E-05	0.08	0.76792	0.902789
COMPOUND_653_5592a11_6700115	Rp	+	653.5592	11.67				(1330.156, 88.97)(671.5682, 2666.77)(672.5686, 898.2)(654.5673, 20811.75)(655.5704, 9128.46)(656.5674, 2980.81)(657.5774, 514.58)	143941.1	86927.02	121220.1	75829.63	140204.7	48053.8	93784.17	62516.07	-1.18	5.47E-07	2.92E-06	0.19	0.439253	0.715597
COMPOUND_654_5058a11_672439	Rp	-	654.5058	11.67				(653.4985, 21537.19)(654.5016, 9328.94)(655.5041, 2293.3)(656.5057, 468.6)	63756.15	34742.27	72520.91	30012.92	39430.25	19526.95	53688.36	25661.81	-1.18	7.69E-08	4.97E-07	-0.25	0.284287	0.593016
COMPOUND_655_139a9_063978	Rp	-	655.1390	9.06				(768.1448, 250.64)(769.1407, 3828.7)(770.1437, 1033.83)(690.1124, 224.87)(691.1204, 998.13)(692.1134, 517.34)(654.132, 6775.35)(655.1337, 1476.21)(656.1364, 322.7)	25223.11	27322.73	18234.12	4368.089	19807.5	7055.327	20579.64	4917.659	-0.71	0.006703	0.014784	0.78	0.013001	0.173893
COMPOUND_655_3087a9_856484	Rp	-	655.3087	9.86				(654.3012, 17953.63)(655.3051, 5644.1)(656.3065, 1446.25)(657.3076, 362.47)	38062.28	21979.54	34390.29	17557.97	22592.33	7040.171	25963.07	8458.036	-1.18	4.35E-07	2.41E-06	0.25	0.301058	0.609696
COMPOUND_655_3176a10_095957	Rp	-	655.3176	10.10				(654.3087, 2698.71)(655.313, 1263.56)(656.3162, 8595.67)	69998.11	18731.41	73936.33	15508.37	45905.55	7592.888	53782.8	11836.44	-0.27	0.321974	0.423801	-0.07	0.822056	0.922833
COMPOUND_656_3167a10_65436	Rp	-	656.3167	10.65				(1347.5841, 262.23)(1348.5775, 150.16)(1349.5895, 245.5)(769.2862, 618.07)(770.2789, 498.79)(771.2799, 96.63)(691.2838, 570.28)(1357.68, 287.7)(1358.6558, 242.67)(655.3088, 13960.76)(656.3105, 4891.58)(657.3125, 1097.27)(658.3183, 219.8)(659.314, 28.84)(1311.6564, 247.79)(1312.6826, 159.58)(1313.6428, 567.51)(1314.6466, 529.39)(1315.6292, 377.36)(701.3099, 853.6)(702.3047, 721.06)	188559.1	26698.53	197902.6	31729.41	147354.9	22672.1	148217.5	27049.9	-1.15	3.94E-06	1.71E-05	-0.23	0.394439	0.690408
COMPOUND_656_471a9_638034	Rp	+	656.4710	9.64				(346.2788, 989.51)(346.773, 209.71)(1313.9813, 60.28)(679.4592, 1291.73)(680.4596, 603.82)(681.4575, 404.35)(695.4593, 364.44)(674.5054, 20904.73)(675.5076, 7716.32)(676.4923, 3143.82)(677.5105, 446.33)(678.5174, 78.86)(657.4769, 3173.26)(658.4804, 1287.48)(659.4741, 518.66)(329.246, 3058.73)(329.7387, 50.99)(330.2522, 517.25)	271641.6	67986.5	252091.6	51076.81	291942.3	51663.97	294476.2	72556.57	0.20	0.482366	0.58406	0.44	0.208449	0.518913
COMPOUND_656_4989a11_759014	Rp	+	656.4989	11.76				(1313.9818, 171.61)(674.5079, 5185.12)(657.508, 21569.58)(658.5117, 8990.35)(659.5163, 2498.0)(660.5195, 537.76)	134638.3	54635.09	151552.2	53232.36	114361.3	65631.25	169024.4	54619.7	-0.97	3.02E-05	0.000107	-0.31	0.230558	0.537492
COMPOUND_657_3229a10_032313	Rp	-	657.3229	10.03				(770.2938, 281.34)(1359.6316, 51.98)(1360.6042, 111.47)(656.3168, 7354.64)(657.3174, 2609.87)(658.3216, 856.28)(659.3233, 93.02)	22603.81	16597.53	29321.63	24040.15	36363.91	16694.23	37625.2	18978.52	-0.01	0.973736	0.985552	-0.05	0.880023	0.950612
COMPOUND_657_3237a10_083144	Rp	-	657.3237	10.08				(770.2854, 239.34)(1360.6744, 92.34)(656.3172, 15682.4)(657.3196, 4921.05)(658.3229, 1204.53)(659.3217, 260.49)	67541.91	26484.34	66170.63	27771.17	44109.85	12774.42	54854.6	16654.15	-1.10	0.00025	0.000729	0.50	0.142192	0.448182
COMPOUND_659_3395a10_30282	Rp	-	659.3395	10.30				(658.3325, 7008.41)(659.3353, 2206.22)(660.3397, 885.5)(661.3402, 184.8)	26039.62	8281.057	27551.09	8168.247	32893.93	31610.99	29081.73	8341.729	0.10	0.730068	0.803968	0.08	0.815103	0.92157
COMPOUND_659_4197a10_549629	Rp	+	659.4197	10.55				(682.4062, 17897.64)(683.4108, 7033.72)(684.4191, 4196.56)(685.4337, 975.76)(698.4088, 1969.03)(699.3924, 201.3)(660.4264, 20019.51)(661.432, 7609.99)(662.4392, 9162.62)(663.4466, 4250.7)(1336.882, 8964.86)(1337.8876, 4077.99)	348163.3	151386	274340.4	140335.4	38340.33	36196.17	39919	41470.4	-1.34	1.81E-10	2.59E-09	0.17	0.415196	0.697566
COMPOUND_659_4814a9_375362	Rp	+	659.4814	9.38				(660.4896, 20784.92)(661.4898, 8353.0)(662.492, 1989.96)(663.4963, 409.76)(330.7487, 236.77)(331.2602, 706.92)(331.7501, 42.62)	216351.4	50298.17	189451.3	35939.67	236525.2	41643.57	232143.9	58701.17	0.25	0.374655	0.476411	0.59	0.087764	0.350433
COMPOUND_659_5492a13_779225	Rp	+	659.5492	13.78	DAG 38:5	2	40 eV: 339.2904(100), 81.0701(40), 203.1752(27), 513.4333(19), 660.5201(11)	(1342.0519, 328.37)(660.557, 15987.28)(661.5603, 7489.21)(662.5638, 1980.62)(663.5657, 531.49)	67273.58	30928.92	73567.36	32555.4	82116.14	38879.61	118407.4	54397.17	0.55	0.023248	0.044977	0.48	0.090791	0.358068
COMPOUND_660_5214a11_417529	Rp	+	660.5214	11.42				(683.5114, 1671.7)(684.4974, 981.62)(685.4873, 1072.42)(699.4935, 1168.06)(700.497, 3182.24)(661.5283, 11459.93)(662.5304, 4983.49)(663.5369, 1463.34)	74868.26	34696.04	72941.96	39134.5	61174.64	35196.49	56715.26	36462.69	-0.46	0.097659	0.159599	-0.30	0.361528	0.667345

									1st trimester				3rd trimester									
									Control		Pre-eclampsia		Control		Pre-eclampsia		Time			Interaction		
Compound	Column	Ion_mode	Mass	Retention_	Identification	ID_level	MSMS_spectra	CompositeSpectrum	Mean	SD	Mean	SD	Mean	SD	Mean	SD	Value	raw p	FDR_p	Value	raw p	FDR_p
COMPOUND_660_5218a0_9025708	Hilic	+	660.5218	0.90				(683.5173, 713.06)(684.5128, 298.81)(685.5283, 341.1)(678.5578, 6018.52)(661.5281, 11326.26)(662.5316, 4601.78)(663.5336, 967.97)(664.5351, 243.22)	66336.27	16374.19	72766.8	22973	66664.64	15633.62	61348.14	16240.21	0.00	0.987379	0.994444	-0.62	0.047169	0.27735
COMPOUND_661_3967a1_0089915	Hilic	+	661.3967	1.01				(684.3899, 2825.8)(685.3901, 803.43)(686.4031, 1047.96)(687.4194, 589.64)(662.4032, 22510.94)(663.408, 8233.37)(664.4169, 4105.82)(665.4248, 1156.22)	#DIV/0!	#DIV/0!	#DIV/0!	#DIV/0!	#DIV/0!	#DIV/0!	#DIV/0!	#DIV/0!	-1.39	5.7E-12	1.57E-10	-0.01	0.96453	0.981972
COMPOUND_661_5655a13_280865	Rp	+	661.5655	13.28				(684.5564, 1700.98)(685.5594, 825.65)(686.5722, 10365.06)(662.5724, 35360.2)(663.5755, 16253.5)(664.5783, 4125.1)(665.5823, 804.92)(666.5867, 136.71)	125896.1	65684.05	115605.4	69650.57	141229.2	36095.49	188441.1	112294.5	0.42	0.134079	0.208527	0.53	0.113907	0.400254
COMPOUND_662_4503a12_871078	Rp	+	662.4503	12.87				(1325.8964, 4112.47)(1326.9025, 3740.35)(1327.904, 1823.14)(1328.9048, 702.42)(1329.9103, 210.08)(1363.9434, 353.89)(1364.9438, 262.37)(685.4369, 15036.06)(686.4396, 7387.51)(687.4435, 1766.21)(688.4456, 338.03)(689.4363, 70.63)(1347.8374, 881.59)(1348.8241, 639.09)(1349.8071, 682.06)(1350.81, 634.0)(701.4103, 1445.56)(702.4144, 731.64)(703.4131, 411.74)(680.482, 1260.92)(681.4829, 505.63)(682.4875, 70.43)(663.4561, 177122.25)(664.4589, 81878.22)(665.4619, 18639.52)(666.4643, 3289.35)(667.4661, 645.46)(668.4632, 96.32)(1342.926, 1351.52)(1343.9319, 1201.44)(1344.9347, 568.0)(1345.9473, 294.08)(1346.9417, 118.76)	2831840	862529.2	2489481	515762	2300344	497108.2	2416858	749479.6	-0.66	0.026341	0.050186	0.44	0.211017	0.519294
COMPOUND_663_5814a13_940646	Rp	+	663.5814	13.94				(686.5722, 4281.22)(687.5753, 2054.35)(688.583, 1201.93)(664.5881, 60944.59)(665.5912, 27802.11)(666.5945, 6693.88)(667.5943, 1386.02)(668.5978, 265.48)	246000.9	136052.9	224636.8	142738.5	248943.1	93455.68	383503.7	257729.7	0.24	0.39613	0.500048	0.63	0.068653	0.319436
COMPOUND_665_2605a10_106638	Rp	-	665.2605	10.11				(1365.5002, 323.92)(1366.4836, 198.53)(1367.5173, 222.88)(778.2322, 1055.0)(779.2278, 653.58)(780.2256, 1391.45)(781.2226, 1168.09)(782.2315, 684.81)(783.2233, 546.84)(784.2363, 341.24)(700.2192, 626.08)(701.2219, 319.76)(702.2296, 865.42)(703.2354, 388.87)(704.2439, 1058.58)(1375.4968, 199.21)(1376.4762, 237.76)(1377.4882, 310.21)(664.2546, 5777.17)(665.2575, 2082.42)(666.2691, 1462.62)(667.259, 292.98)(1329.5039, 249.27)(1330.4956, 289.27)(1331.4679, 394.93)(1332.4946, 316.86)(1333.5079, 259.72)(710.2522, 2367.26)(711.254, 744.92)(712.2591, 5164.42)(713.2622, 1734.71)(714.2575, 2296.48)(715.2597, 869.97)(716.2596, 1010.0)(1443.4474, 240.75)(1444.476, 197.13)	210873.8	22735.24	210055.7	37273.55	210072.6	21091.64	192515.2	28435.33	-0.15	0.588852	0.683906	-0.58	0.080919	0.340768
COMPOUND_665_2821a10_3792715	Rp	-	665.2821	10.38				(1365.539, 262.11)(1366.5197, 275.53)(1367.5381, 382.64)(1368.525, 236.43)(1369.5162, 273.7)(778.2324, 1118.07)(1375.6078, 137.26)(1376.5886, 156.62)(1377.6038, 150.76)(664.2683, 943.68)(665.273, 581.43)(666.2703, 12541.6)(1329.5496, 388.47)(1443.4968, 225.56)(1444.4974, 183.81)(1445.4807, 155.29)	115145.4	13455.98	118372.1	14919.07	91718.08	9512.449	98164.15	12456.21	-1.38	4.47E-07	2.46E-06	0.22	0.45118	0.723875
COMPOUND_665_3256a5_558744	Rp	-	665.3256	5.56				(700.2938, 390.37)(701.2941, 149.14)(702.2963, 86.55)(664.3176, 4752.85)(665.3211, 1681.06)(666.3237, 445.05)(667.3223, 52.63)(1443.5835, 108.15)	17877.92	4029.024	17958.65	4994.608	20654.73	6709.355	18145.7	4845.24	0.38	0.180981	0.268138	-0.33	0.332924	0.636996
COMPOUND_665_326a0_84776014	Hilic	+	665.3260	0.85				(688.3166, 1037.07)(689.3185, 399.69)(690.3177, 279.62)(666.3333, 48279.41)(667.3364, 15614.73)(668.344, 7047.74)(669.3491, 1972.76)(670.3526, 403.08)(333.6723, 374.29)(334.175, 193.91)	175357.5	66770.38	185143	79911.73	207142.3	89945.11	183271.4	71894.06	0.37	0.203465	0.294704	-0.42	0.231965	0.537875
COMPOUND_665_33a5_5597057	Rp	+	665.3300	5.56				(688.3151, 1445.12)(689.3175, 461.93)(690.3212, 129.22)(363.6369, 182.49)(364.1428, 72.82)(352.6458, 1651.65)(353.1475, 612.33)(353.6447, 256.42)(344.6649, 6161.27)(345.1671, 2172.35)(345.6667, 722.45)(346.176, 205.35)(704.2891, 134.69)(342.1858, 54017.04)(342.6874, 20641.86)(343.1891, 4677.26)(343.6906, 1089.22)(666.3338, 18414.08)(667.3371, 6271.91)(668.3398, 1732.81)(669.345, 385.05)(333.6727, 1813.15)(334.1752, 908.88)(334.6722, 140.89)	237082.1	95833.92	228826.3	85760.94	276916.5	120284.4	257300.7	107648	0.39	0.185906	0.273992	-0.11	0.759856	0.896853
COMPOUND_665_3573a11_082845	Rp	+	665.3573	11.08				(1369.7261, 414.07)(688.3359, 3719.17)(1353.7106, 152.39)(1354.7219, 271.81)(1355.7274, 288.06)(704.3263, 10302.49)(705.3245, 3481.17)(706.3235, 2314.57)(683.3886, 842.97)(666.3686, 50153.41)(667.3716, 19718.92)(668.3723, 4619.56)(669.3699, 924.52)	111745.3	51255.45	110238.8	53053.51	178112.8	21501.7	185670.9	72978.24	0.95	1.24E-05	4.88E-05	0.22	0.360257	0.666906
COMPOUND_665_4067a10_706241	Rp	-	665.4067	10.71				(1365.7634, 535.15)(1366.7332, 468.86)(1367.7592, 310.7)(778.3765, 1288.35)(779.3936, 1819.39)(780.4021, 822.93)(1375.8785, 563.34)(1376.8574, 74.84)(1377.8679, 570.39)(664.3988, 2964.97)(665.3993, 1414.17)(666.394, 6149.21)(667.398, 4574.72)(668.3997, 1989.22)(1329.7875, 382.45)(1330.7861, 235.07)(1331.8132, 242.33)(1332.828, 334.91)(1333.8344, 605.1)(710.4112, 6295.46)(1443.8156, 463.91)(1444.8206, 299.75)	#DIV/0!	#DIV/0!	#DIV/0!	#DIV/0!	#DIV/0!	#DIV/0!	#DIV/0!	#DIV/0!	-0.69	0.008213	0.017767	0.01	0.968758	0.982722
COMPOUND_665_4237a10_47626	Rp	-	665.4237	10.48				(778.4352, 1095.77)(700.4107, 2021.63)(701.404, 749.66)(702.4043, 2055.64)(1375.9225, 414.86)(1376.9128, 534.46)(1377.883, 854.03)(1378.8798, 672.84)(1379.8861, 1090.39)(664.419, 38658.25)(665.4222, 14373.77)(666.4241, 3791.79)(667.4247, 985.46)(1329.8396, 521.69)(1330.8403, 378.79)(1331.8395, 762.31)(710.4195, 1603.16)(711.4187, 705.67)	97294.59	50678.3	117444.2	74811.24	12594.67	2096.101	24009.75	9791.371	-1.28	2.96E-09	2.79E-08	-0.10	0.660869	0.843694
COMPOUND_666_1793a9_475972	Rp	-	666.1793	9.48				(1367.3328, 159.29)(1368.3423, 76.63)(779.1754, 594.84)(780.1865, 351.6)(781.1638, 582.89)(701.1539, 37055.0)(702.157, 9110.67)(703.1585, 1371.19)(704.1597, 328.18)(665.1775, 2210.88)(666.195, 1676.98)(667.201, 582.3)	243531.9	24865.48	244877.5	21265.96	254153.2	28347.59	246508.3	24604.25	0.43	0.156885	0.236958	-0.45	0.221802	0.531023
COMPOUND_666_523a13_601004	Rp	+	666.5230	13.60				(1334.0464, 73.83)(1335.0754, 52.83)(689.5121, 10581.56)(690.5154, 4507.15)(691.5186, 1187.71)(692.5213, 328.71)(705.4866, 1000.77)(706.4969, 478.97)(707.496, 276.03)(684.5571, 32073.3)(685.5605, 15165.89)(686.5636, 4005.03)(687.5676, 838.6)(667.5327, 2305.23)(668.5352, 1223.07)(669.5476, 452.4)(670.5507, 127.04)	58634.1	31866.3	67459.23	50445.34	104743.6	46614.13	189001.9	118363.2	0.87	0.000113	0.000349	0.51	0.042358	0.268638
COMPOUND_667_2983a10_647927	Rp	-	667.2983	10.65				(1369.5591, 351.18)(1370.5465, 208.31)(1371.5219, 261.06)(780.2558, 833.63)(666.2892, 1001.58)(667.2904, 493.72)(668.286, 13890.82)(1333.5704, 308.52)(1334.5706, 264.71)	82358.59	9002.124	80994.17	14637.15	70499.21	8286.453	69480.91	11817.38	-0.54	0.044014	0.078231	-0.12	0.685538	0.853695
COMPOUND_667_4769a10_860655	Rp	-	667.4769	10.86				(1369.9388, 635.91)(1370.9515, 364.3)(780.4847, 1390.4)(781.491, 819.38)(782.4596, 2638.73)(702.4608, 850.4)(703.4592, 520.79)(704.462, 11351.56)(1379.9253, 656.54)(1380.931, 517.99)(1381.9167, 843.08)(1382.9232, 624.16)(1383.928, 954.39)(666.4697, 8175.77)(667.4723, 4447.23)(668.4705, 1896.47)(669.4718, 231.2)(1333.9087, 423.23)(1334.9443, 192.39)(1447.9388, 443.95)(1448.9524, 481.79)(1449.9454, 487.13)(1450.945, 585.0)(1451.9475, 691.99)	90377.6	16603.63	91059.15	15345.41	114285.1	15926.51	114551.2	12624.85	1.25	2.41E-06	1.1E-05	-0.03	0.915421	0.961085
COMPOUND_668_1981a10_400407	Rp	+	668.1981	10.40				(1337.3989, 208.28)(686.2458, 647.14)(687.2375, 988.63)(707.1875, 2772.11)(669.204, 9776.51)(670.2091, 3412.49)(671.2017, 6361.58)(672.208, 3204.02)(673.2048, 1693.59)(674.205, 697.55)(675.2079, 296.2)(1354.4165, 193.89)	40769.64	23767.59	35853.41	14576.24	88967.57	27692.92	84699.42	34057.12	1.49	5.18E-11	9.34E-10	-0.08	0.703785	0.86442

									1st trimester				3rd trimester									
									Control		Pre-eclampsia		Control		Pre-eclampsia		Time			Interaction		
Compound	Column	Ion_mode	Mass	Retention_	Identification	ID_level	MSMS_spectra	CompositeSpectrum	Mean	SD	Mean	SD	Mean	SD	Mean	SD	Value	raw p	FDR_p	Value	raw p	FDR_p
COMPOUND_669_2917a10_615242	Rp	-	669.2917	10.62				(1373.5986, 220.39)(1374.6218, 218.49)(782.2666, 1033.36)(783.2662, 511.16)(784.2721, 1507.51)(785.2588, 1104.23)(786.2656, 792.02)(787.2644, 420.17)(788.2747, 341.49)(704.2653, 861.66)(705.2706, 452.38)(706.2824, 1574.94)(707.2867, 728.13)(708.2759, 1208.74)(1383.5466, 168.05)(1384.5385, 284.68)(668.2862, 9745.67)(669.2889, 3840.15)(670.2899, 1448.05)(671.2757, 122.99)(1337.5691, 311.71)(1338.5732, 363.22)(1339.5782, 233.94)(714.2892, 5064.96)(715.2891, 1696.2)(716.2913, 5176.29)(717.2949, 1676.34)(718.2857, 2210.51)(719.2886, 933.72)(720.2857, 1110.62)(721.2935, 652.0)	188777.9	82233.21	204495.3	70851.55	150250.1	86103.57	170040.6	81994.06	-0.63	0.033888	0.062656	0.06	0.861682	0.942452
COMPOUND_669_5541a11_606946	Rp	+	669.5541	11.61				(1378.1016, 104.85)(692.5379, 2307.79)(708.5086, 4445.58)(709.511, 3668.41)(710.5045, 2283.33)(670.562, 34856.31)(671.5654, 14622.41)(672.5686, 3930.33)(673.5714, 755.03)	130072.9	92144.07	102611.5	65574.59	99789.67	35771.85	96212.44	42999.59	-1.23	9.62E-08	6.01E-07	0.14	0.568306	0.788601
COMPOUND_670_2151a10_66635	Rp	+	670.2151	10.67				(1379.336, 366.22)(709.1943, 2190.42)(710.1776, 1039.69)(711.1903, 916.81)(688.2807, 1057.49)(671.22, 10099.25)(672.2254, 4277.22)(673.2169, 7388.2)(674.2198, 2391.0)(675.2216, 2036.78)(676.2241, 861.65)(677.2249, 389.34)	31440.6	8046.064	22480.33	8422.645	95274.3	24947.4	85905.39	33088.74	1.08	3.57E-09	3.31E-08	0.26	0.164711	0.47785
COMPOUND_670_5009a11_60244	Rp	-	670.5009	11.60				(783.4829, 583.04)(669.4934, 12835.58)(670.4965, 5267.92)(671.4999, 1410.28)(672.4934, 363.27)(673.5038, 68.2)(715.4882, 625.82)(716.4826, 303.08)(717.5061, 2664.27)(718.5097, 1279.23)	100868.4	54490.18	113067.7	52544.53	57723.67	27162.77	69885.89	18655.99	-1.26	2.57E-09	2.5E-08	-0.21	0.332808	0.636996
COMPOUND_672_2846a10_161604	Rp	-	672.2846	10.16				(707.2647, 1396.26)(671.2781, 10060.74)(672.2798, 3561.05)(673.2836, 807.89)(717.2908, 122.44)(1343.5175, 387.35)(1344.5215, 330.8)	70467.1	29979.23	64022.79	25965.72	60696.21	26881.37	62522.54	28151.65	-0.61	0.040873	0.073578	0.43	0.227378	0.535063
COMPOUND_672_285a10_268594	Rp	-	672.2850	10.27				(707.2677, 1671.17)(671.2779, 12825.76)(672.2809, 4009.04)(673.2822, 1503.12)(1343.5262, 99.79)(1344.5149, 80.33)(717.2697, 1057.06)	119234.7	50848.06	102574.3	54383.39	119943.4	40687.84	120759.4	46720.48	0.02	0.942512	0.963038	0.55	0.116813	0.405455
COMPOUND_672_4958a11_685205	Rp	+	672.4958	11.69				(695.4936, 1801.26)(696.489, 1112.88)(673.5031, 17391.64)(674.5065, 7898.01)(1363.0574, 83.71)	93279.02	50692.61	104832.5	53223.07	58304	47380.94	91112.38	31386.15	-0.96	1.99E-05	7.45E-05	-0.26	0.30464	0.613089
COMPOUND_672_5204a11_339026	Rp	+	672.5204	11.34	SM d32:2	2	10 eV: 673.528(100), 184.072(50), 561.4149(4), 414.042(4), 86.0952(4)	(1345.9983, 215.32)(695.5033, 8065.68)(696.4997, 5748.37)(697.4961, 3766.66)(711.5006, 4436.08)(690.526, 1481.54)(673.5301, 83999.55)(674.5326, 34397.33)(675.5354, 8276.67)(676.5309, 1731.01)(677.5528, 340.94)	418777.3	97773.5	414742.2	134407.4	337609.4	107554.1	324201.1	92552.36	-0.82	0.000114	0.000351	-0.03	0.89272	0.953702
COMPOUND_672_5207a0_8844271	Hilic	+	672.5207	0.88				(695.5104, 2779.22)(696.5133, 1320.85)(697.5257, 8654.32)(698.5292, 4144.41)(699.543, 6984.78)(673.5286, 88253.3)(674.5318, 37185.59)(675.5444, 356427.66)	957721.9	234854	1050988	355962	1088848	238616.5	1035566	266731.1	0.55	0.041429	0.0744	-0.59	0.058464	0.299231
COMPOUND_673_4975a9_63872	Rp	+	673.4975	9.64				(1385.953, 58.14)(1369.9958, 54.59)(346.2702, 900.59)(346.7726, 288.28)(347.2599, 348.88)(674.5052, 19880.7)(675.5076, 7817.14)(676.4995, 2319.22)(677.5102, 394.59)(678.5183, 78.11)(337.7578, 422.97)(338.2658, 1821.53)(338.7642, 24.8)	249748	64750.95	214576	42520.4	268561.8	44541.65	268163	67877.76	0.30	0.285478	0.384886	0.59	0.082221	0.343638
COMPOUND_674_2725a10_392099	Rp	+	674.2725	10.39				(1371.5741, 145.97)(713.2408, 790.22)(714.24, 808.8)(675.2808, 6235.89)(676.2777, 2405.2)(677.2596, 1288.19)(678.2837, 292.15)	34679.57	13175.86	29090.15	12970.14	41551.08	10852.66	37750.46	9586.416	0.87	0.000812	0.002154	0.02	0.952667	0.980085
COMPOUND_674_54a11_606961	Rp	+	674.5400	11.61	SM d32:1	2	10eV: 675.5446(100), 184.0737(35), 388.867(3)	(1350.0846, 710.89)(1351.0935, 588.12)(1352.082, 264.24)(1388.0002, 156.58)(697.5274, 21819.92)(698.5306, 9572.64)(699.5363, 2927.31)(700.5573, 231.35)(1372.0112, 232.74)(713.5016, 1757.86)(714.4983, 1189.02)(715.5121, 1112.26)(692.5535, 2255.93)(693.5555, 1122.95)(675.5459, 310016.28)(676.5492, 125283.59)(677.5515, 26677.19)(678.557, 5112.04)	1510548	404228.4	1607133	684530.7	1488993	354308	1412110	370291.4	0.00	0.991959	0.997273	-0.31	0.322109	0.628989
COMPOUND_675_5249a11_220736	Rp	+	675.5249	11.22				(693.5324, 951.78)(676.5333, 20974.51)(677.536, 9481.15)(678.5343, 14321.73)(679.5351, 6350.34)(680.531, 5763.94)	23836.11	9057.114	31498.6	14588.07	99951.7	44505.02	71912.3	29796.93	1.09	2.35E-06	1.07E-05	0.01	0.957092	0.980661
COMPOUND_675_5406a0_88432986	Hilic	+	675.5406	0.88				(698.5294, 6236.98)(699.5429, 7996.6)(700.5464, 3545.12)(693.5645, 392.61)(676.5479, 112781.45)(677.5513, 26413.68)(678.5555, 4956.43)(679.5589, 973.98)(680.5647, 157.46)	392383.5	97023.87	434429.4	142034.3	448677.7	98007.66	421864.7	109994.3	0.55	0.044449	0.078879	-0.67	0.036127	0.252742
COMPOUND_676_5522a11_792699	Rp	+	676.5522	11.79				(699.5236, 1194.14)(700.5473, 970.22)(694.5993, 2237.45)(695.602, 1490.61)(677.559, 3117.34)(678.5655, 3854.64)(679.5733, 2408.13)(680.5827, 9941.78)	90449.92	38099.3	87788.09	48611.35	43104.6	15637.33	46773.58	19970.66	-0.14	0.60716	0.700115	0.23	0.470222	0.734155
COMPOUND_677_3063a10_304156	Rp	-	677.3063	10.30				(790.2787, 679.15)(791.287, 403.28)(712.2663, 2901.88)(676.2994, 9776.0)(677.3001, 3461.84)(678.3014, 1268.82)(679.292, 336.99)	87129.94	24081.66	84246.96	19593.13	86644.57	16795.16	83258.7	19519.1	0.08	0.80168	0.857257	-0.04	0.906962	0.958319
COMPOUND_677_3082a10_222234	Rp	-	677.3082	10.22				(712.2655, 2567.3)(790.2755, 474.28)(791.2746, 263.14)(676.2999, 7425.59)(677.3029, 2488.91)(678.3035, 1005.06)(679.3025, 269.27)	39675.35	15869.28	42490.25	13664.81	42906.33	30829.36	38361.13	28842.74	0.48	0.083807	0.138877	-0.22	0.512878	0.75937
COMPOUND_677_5013a11_656002	Rp	+	677.5013	11.66	PC 28:0	2	20 eV: 184.074(100), 678.5107(90)	(700.4913, 4493.86)(701.5005, 1493.33)(1377.964, 188.93)(716.469, 471.05)(678.5073, 31683.83)(679.5102, 12618.59)(680.5128, 3237.09)(681.5031, 795.9)	161324	90170.64	177533.9	99085.49	167482.1	84587.18	186676.3	106561.4	0.57	0.023322	0.045082	-0.15	0.593254	0.803855
COMPOUND_677_5598a11_508577	Rp	+	677.5598	11.51				(700.5481, 3956.77)(678.567, 22666.42)(679.5703, 10178.6)(680.576, 4531.2)(681.5659, 843.63)	144243.4	80623.47	142826.3	93472.94	176122.8	119990.5	208413.1	133015.3	-0.70	0.003919	0.009069	-0.19	0.510586	0.757607
COMPOUND_679_5756a11_755592	Rp	+	679.5756	11.76				(1360.1598, 210.11)(1398.155, 143.03)(697.5817, 14308.78)(680.583, 55868.91)(681.5863, 24642.31)(682.5848, 7113.09)(683.5821, 1994.05)	#DIV/0!	#DIV/0!	#DIV/0!	#DIV/0!	#DIV/0!	#DIV/0!	#DIV/0!	#DIV/0!	-1.02	2.22E-05	8.16E-05	-0.01	0.974689	0.986465
COMPOUND_680_1589a8_665838	Rp	-	680.1589	8.67				(679.1525, 3474.99)(680.156, 936.16)(681.1713, 218.96)(682.1542, 61.81)(725.1436, 345.44)(726.1331, 239.21)(727.1438, 372.81)	14706.09	2831.089	15195.07	2766.856	14988.64	3047.039	15377.02	2599.436	0.06	0.855055	0.900226	-0.03	0.937288	0.97184
COMPOUND_680_1594a8_602407	Rp	-	680.1594	8.60				(679.151, 4470.21)(680.1528, 1201.34)(681.1536, 338.82)(682.1419, 45.68)(725.1604, 319.12)(726.1436, 211.05)(727.1444, 331.6)	18298.98	2275.179	18039.59	1915.243	18903.14	3135.381	19144.39	2614.266	0.30	0.318976	0.421063	0.17	0.634244	0.828972
COMPOUND_680_5212a11_757137	Rp	-	680.5212	11.76				(679.5143, 9103.44)(680.5176, 3976.64)(681.5197, 1027.82)(682.5216, 244.29)(683.539, 48.11)(1360.0446, 113.88)(725.5029, 816.41)(726.5039, 372.96)(727.5141, 2821.27)(728.5145, 1379.33)	150371.7	64535.01	170958.5	61637.75	121590.5	75172.1	178936.5	95802.33	-1.06	8.91E-06	3.59E-05	-0.17	0.509654	0.757224
COMPOUND_681_3219a1_1681348	Hilic	+	681.3219	1.17				(704.309, 356.29)(705.3157, 215.56)(682.3282, 9048.2)(683.3322, 3273.78)(684.336, 817.41)(685.3386, 152.22)	19920.61	7299.881	20936.64	9352.378	29496.15	15075.75	28517.42	12774.39	0.73	0.010592	0.022335	-0.15	0.649492	0.838541
COMPOUND_681_3225a10_086524	Rp	-	681.3225	10.09				(680.3165, 12665.25)(681.3201, 4568.78)(682.3222, 1076.37)(683.3287, 296.26)	40418.74	8114.936	39490.3	8160.291	29329.14	9610.092	33601.82	8970.777	-1.13	1.06E-05	4.22E-05	0.56	0.044697	0.272788
COMPOUND_681_3582a10_395896	Rp	+	681.3582	10.40				(1363.7657, 520.03)(1364.7784, 161.39)(1401.65, 317.09)(704.3399, 824.45)(720.326, 656.73)(721.3119, 366.68)(699.4085, 733.64)(682.3641, 78912.15)(683.3673, 30392.42)(684.3704, 6789.34)(685.3756, 1154.36)(686.3805, 246.55)	78223.1	66045.82	53962.58	58479.57	102399.7	52920.66	116347.8	78147.73	0.59	0.02252	0.043718	0.55	0.072698	0.326093
COMPOUND_681_4761a10_928599	Rp	-	681.4761	10.93				(1397.9069, 1592.98)(1398.909, 1273.19)(1399.9196, 1786.1)(794.4573, 750.28)(795.4833, 455.91)(716.446, 6492.6)(717.4442, 2247.01)(1407.8773, 764.48)(1408.8798, 743.69)(680.467, 9328.01)(681.4717, 4405.12)(682.4755, 1177.77)(683.4822, 223.43)(726.4688, 1351.15)(727.4661, 728.64)(728.4446, 11386.25)(729.4474, 5733.0)(1475.9679, 547.48)(1476.9814, 630.12)(1477.9772, 582.7)	87011.69	29134.49	94835.55	59890.54	63164.15	28228.33	74375.82	27654.23	-0.15	0.571822	0.668263	0.14	0.672522	0.848471
COMPOUND_681_5337a13_005921	Rp	+	681.5337	13.01				(1364.024, 74.21)(682.541, 13314.75)(683.544, 6527.75)(684.5485, 1832.71)(685.5554, 362.19)	23227.81	12179.18	22460.7	17246.23	28980.85	11557.51	46586.59	27428.9	0.64	0.006774	0.014928	0.54	0.051392	0.283822
COMPOUND_682_3061a10_389372	Rp	-	682.3061	10.39				(717.2756, 1954.94)(718.2824, 2211.45)(719.2855, 829.75)(681.2993, 9231.94)(682.3049, 4094.84)(683.299, 1964.56)(684.2908, 399.72)	73674.88	10439.14	72032.87	8460.086	69941.77	7233.589	69938.15	14029.6	-0.27	0.351902	0.453885	0.61	0.076283	0.331641
COMPOUND_682_3557a0_8448046	Hilic	+	682.3557	0.84				(705.3635, 446.41)(683.3841, 247.3)(700.4182, 940.27)(700.9165, 82.63)(342.1846, 15321.08)(342.6857, 4945.37)(343.1898, 2505.4)(343.6924, 574.27)(344.1913, 116.92)	58763.83	23939.7	55455.21	21759.33	70533.92	31080.75	66116.81	25662.81	0.42	0.148268	0.226065	-0.06	0.868352	0.9447
COMPOUND_683_1948a12_474742	Rp	+	683.1948	12.47				(1367.407, 292.91)(1368.4072, 341.84)(1369.3904, 349.84)(1389.4283, 312.24)(684.2022, 5904.42)(685.2031, 4012.21)(686.202, 3098.33)(1384.409, 282.95)(1385.4034, 111.39)(1386.4203, 219.98)(1387.4258, 229.37)(1388.3967, 224.33)	54854.81	8595.359	50520.22	9271.957	48389.43	7374.584	48481.6	9221.911	-0.60	0.040654	0.073265	0.42	0.224855	0.532591

									1st trimester				3rd trimester									
									Control		Pre-eclampsia		Control		Pre-eclampsia		Time			Interaction		
Compound	Column	Ion_mode	Mass	Retention_	Identification	ID_level	MSMS_spectra	CompositeSpectrum	Mean	SD	Mean	SD	Mean	SD	Mean	SD	Value	raw p	FDR_p	Value	raw p	FDR_p
COMPOUND_684_5764a0_8690934	Hilic	+	684.5764	0.87				(685.5475, 521.09)(343.2954, 17475.92)(343.7887, 857.01)(344.2991, 3380.95)(344.7913, 90.25)(345.302, 350.28)	41701.93	20231.79	46297.56	17740.09	60661.45	39539.81	45436.26	21603.02	0.41	0.147351	0.224821	-0.08	0.816931	0.92157
COMPOUND_685_4348a10_680474	Rp	+	685.4348	10.68				(1409.8856, 1899.4)(708.4253, 4770.46)(709.437, 2538.3)(710.4374, 6021.82)(711.4449, 2205.37)(712.4458, 4231.71)(703.4679, 7214.48)(686.4414, 16917.18)(687.4538, 8366.79)(688.4556, 19923.99)(689.4618, 8270.16)(690.4494, 10414.12)	347312.5	136628.7	346924.5	121509	#DIV/0!	#DIV/0!	254472	#DIV/0!	-1.07	7.35E-07	3.82E-06	0.32	0.173954	0.488459
COMPOUND_686_5365a0_8701679	Hilic	+	686.5365	0.87				(709.5234, 861.61)(710.5327, 478.45)(711.5416, 4923.99)(712.5436, 2036.73)(687.5439, 10422.59)(688.5453, 4560.33)(689.5597, 111729.45)	297793.4	75895.85	318505	120360.4	330989.6	78384.95	303671.1	77881.79	0.36	0.19691	0.28646	-0.45	0.165421	0.47785
COMPOUND_686_5381a11_517818	Rp	+	686.5381	11.52				(709.5104, 3811.89)(710.5086, 2626.34)(704.5772, 2139.73)(705.5795, 931.53)(687.5453, 7512.12)(688.548, 3470.33)(689.5466, 1157.34)	44416.96	14355.97	52141.97	26079.16	43574.5	18003.31	37513.93	15589.02	-0.45	0.067231	0.115136	-0.21	0.457736	0.727948
COMPOUND_687_3214a10_29665	Rp	+	687.3214	10.30				(1375.6824, 308.65)(726.3059, 872.17)(688.328, 8577.99)(689.3315, 3660.26)(690.3282, 3738.31)(691.3301, 1205.86)(692.319, 345.96)(1392.7479, 379.41)	29435.98	9775.082	24565.95	9485.062	32686.64	12237.18	33374.15	13355.07	0.34	0.232371	0.327267	0.49	0.148192	0.456493
COMPOUND_687_3876a10_863252	Rp	+	687.3876	10.86				(688.3946, 28117.34)(689.3975, 10989.49)(690.3969, 3499.3)(691.3945, 835.74)	11227	#DIV/0!	#DIV/0!	#DIV/0!	77370.64	12190.16	79488.2	16618.45	1.04	1.3E-09	1.39E-08	0.30	0.092029	0.359807
COMPOUND_687_4119a0_9568352	Hilic	+	687.4119	0.96				(710.4127, 1246.91)(711.4176, 455.35)(712.4268, 1900.88)(713.4288, 735.42)(705.4512, 808.34)(688.4196, 17133.05)(689.4233, 6425.55)(690.4347, 14650.79)(691.4384, 5712.42)(692.4429, 2260.56)(693.4503, 781.71)(694.4528, 1042.74)	54401.64	40840.51	59238.57	34787.37	#DIV/0!	#DIV/0!	16711	14584.13	-1.30	3.68E-09	3.39E-08	0.10	0.650503	0.838541
COMPOUND_687_5141a9_882115	Rp	+	687.5141	9.88				(1375.982, 60.23)(1414.0369, 69.09)(353.2721, 1321.24)(353.7755, 67.03)(688.5212, 20793.59)(689.523, 7982.51)(344.7615, 384.37)(345.2685, 410.29)(345.7617, 46.51)	155038.9	42117.97	134849.1	27071.95	167786.4	27226.22	166484.8	43125.96	0.25	0.375652	0.477409	0.57	0.098038	0.36878
COMPOUND_687_5806a13_782784	Rp	+	687.5806	13.78				(688.5883, 11804.9)(689.5911, 5619.4)(690.5951, 1461.88)(691.5986, 272.13)	33561.24	22973.15	31312.51	18889.98	40165.71	13432.82	63707.7	36506.63	0.56	0.029596	0.055774	0.62	0.040467	0.265689
COMPOUND_688_5536a11_812392	Rp	+	688.5536	11.81	SM d33:1	2	10 eV: 689.5641(100), 184.0735(15), 81.0719(3)	(1378.1638, 162.11)(1416.0829, 110.33)(711.5407, 3247.7)(712.5351, 1978.97)(713.5491, 610.58)(714.5534, 49.29)(1400.1448, 180.26)(706.5894, 2245.67)(707.5987, 559.69)(689.5614, 71725.76)(690.5641, 30426.04)(691.5672, 7146.9)(692.5558, 1107.35)(1395.1685, 248.96)	456196.2	124668.7	459250.4	190965	467819.4	118680.4	420165.5	107008.5	0.10	0.712886	0.788904	-0.27	0.409398	0.694731
COMPOUND_689_2666a10_208906	Rp	-	689.2666	10.21				(1413.4645, 162.32)(1414.4478, 244.98)(802.2249, 763.19)(688.2604, 11594.82)(689.264, 3837.89)(690.257, 5470.02)(691.2575, 1684.71)(692.2632, 1501.42)(693.2662, 502.69)(1377.5009, 162.38)(1378.5055, 176.76)(1379.5317, 131.82)(1380.5016, 148.95)(1381.5043, 184.97)(734.2508, 1592.92)(735.2498, 882.59)(736.2575, 2002.06)(737.2596, 937.12)(1491.458, 123.71)	89030.22	58474.49	64651.82	46435.3	146232.5	51276.46	112215.6	54098.57	0.60	0.03441	0.063362	0.13	0.698105	0.862515
COMPOUND_689_2678a10_278288	Rp	-	689.2678	10.28				(1413.451, 147.81)(1414.4779, 192.01)(802.2362, 689.13)(688.2601, 12205.91)(689.2643, 4671.72)(690.2571, 5075.03)(691.2592, 1887.07)(692.261, 1374.22)(693.2674, 512.57)(1377.4868, 151.15)(1378.495, 240.88)(1379.4929, 244.3)(1380.5096, 141.9)(1381.4899, 166.25)(734.2573, 1857.14)(735.2603, 1032.89)(736.2591, 1745.05)(737.2594, 687.27)(1491.526, 153.91)	159948	86809.19	127257.8	86634.97	196234.4	55198.86	140443.3	68583.91	0.42	0.15291	0.231731	-0.27	0.443553	0.71862
COMPOUND_689_4929a9_112516	Rp	+	689.4929	9.11				(720.493, 679.38)(720.9908, 424.39)(354.2662, 2559.64)(354.7706, 416.05)(355.2645, 12697.86)(690.5004, 7740.61)(691.4986, 3665.2)(692.4974, 1087.94)(693.5057, 216.49)(345.7549, 500.72)(346.2549, 1182.7)(346.7559, 21.71)	137695	33970.67	127178.4	37745.96	126752.2	21062.32	123378.6	38117.81	-0.39	0.191408	0.280289	0.43	0.229451	0.535521
COMPOUND_689_4987a12_1908	Rp	-	689.4987	12.19				(688.4915, 9709.51)(689.4946, 3902.25)(690.4979, 1232.48)(691.5021, 235.01)	12210	#DIV/0!	14749.33	5452.56	24127.5	9546.464	38071.29	20842.76	1.01	2.89E-11	6.09E-10	0.45	0.003326	0.082317
COMPOUND_689_5557a0_8700491	Hilic	+	689.5557	0.87				(712.5433, 1055.6)(713.5583, 1738.85)(714.5609, 1026.24)(707.5914, 3332.83)(708.5941, 464.96)(690.5628, 46213.26)(691.5659, 11325.58)(692.5691, 1920.69)(693.5691, 324.82)	138786.6	35666.44	150618	54747.37	154555.5	39215.54	140088.7	36487.1	0.34	0.220554	0.314161	-0.52	0.108689	0.391238
COMPOUND_691_4231a10_704643	Rp	-	691.4231	10.70				(1417.7834, 312.26)(1418.753, 204.17)(804.3781, 964.32)(805.3968, 177.9)(726.4116, 1932.58)(690.4036, 28236.34)(691.4056, 15468.79)(692.4084, 5238.95)(693.4094, 1539.07)(1381.7625, 274.34)(1382.7426, 396.27)(736.4184, 3010.57)(737.4055, 1517.55)(1495.8741, 581.81)(1496.8597, 563.45)	88017.37	30469.29	97170.12	45775.01	79088	35014.44	89161.45	40961.7	-0.75	0.004583	0.010385	0.06	0.833841	0.927314
COMPOUND_692_5062a11_310362	Rp	-	692.5062	11.31				(1429.9694, 196.89)(691.5017, 8041.77)(692.505, 3411.83)(693.5026, 1151.41)(694.5072, 199.04)(1383.9637, 115.0)(737.4969, 1400.09)	83513.33	20237.91	101268.9	29082.37	105642.9	26767.02	111999.5	38371.16	0.86	0.001095	0.002831	-0.07	0.811819	0.920552
COMPOUND_693_489a12_86907	Rp	+	693.4890	12.87				(1387.9474, 344.46)(1425.9443, 263.58)(1426.9309, 306.14)(1427.9523, 268.92)(694.496, 13993.09)(695.4993, 6670.73)(696.5025, 1789.98)(697.506, 418.72)	56140.17	22317.85	45998.18	17215.5	46598.21	14211.43	49369.6	18757.84	-0.41	0.157492	0.237716	0.64	0.064353	0.307286
COMPOUND_695_2695a10_272763	Rp	-	695.2695	10.27				(1425.4901, 182.61)(1426.4784, 205.26)(1427.457, 220.75)(808.2409, 646.06)(809.2524, 387.01)(730.2085, 805.01)(731.2213, 551.56)(732.2077, 208.48)(694.262, 7081.29)(695.2648, 3131.38)(696.2624, 1259.56)(697.2524, 249.04)(1389.5197, 222.74)(740.2927, 1840.25)	58777.92	40784.27	79549.62	46085.21	44775.43	40921.07	62952.94	53563.08	0.38	0.179246	0.266093	-0.50	0.139048	0.444794
COMPOUND_695_3701a10_391698	Rp	+	695.3701	10.39				(1391.791, 613.28)(1429.7736, 389.38)(1413.7523, 454.63)(734.3386, 437.56)(713.4161, 1222.27)(696.3804, 17161.62)(697.3834, 6962.67)(698.3858, 1895.15)(699.3882, 521.35)(1408.7917, 576.02)	41723.34	9407.973	39976.5	9366.083	42027.14	12641.44	41871.02	13007.91	0.27	0.336637	0.437453	0.31	0.356046	0.665596
COMPOUND_695_5708a11_684356	Rp	+	695.5708	11.68				(718.5598, 3500.47)(719.5666, 1808.27)(734.5176, 620.16)(735.522, 1640.57)(736.5208, 1360.99)(713.5915, 892.74)(696.5781, 11635.73)(697.5791, 5635.21)(698.5804, 1650.09)(699.5832, 466.27)(700.5967, 93.77)(1409.1765, 185.62)	162700.7	126893.5	128519.5	97981.99	157918.3	143492.5	109367.9	100551.1	-1.29	1.19E-08	9.65E-08	0.13	0.578	0.792726
COMPOUND_696_5162a11_681318	Rp	-	696.5162	11.68				(809.5339, 789.78)(810.5502, 1363.36)(695.5093, 49707.27)(696.5126, 22736.35)(697.5158, 5799.3)(698.5182, 1190.56)(699.5199, 210.86)	158878.9	89381.86	160669.5	79294.48	107277.3	77385.34	119364.1	68076.25	-1.27	1.01E-08	8.39E-08	-0.08	0.716857	0.876415
COMPOUND_698_2994a10_378443	Rp	-	698.2994	10.38				(733.2599, 737.52)(697.2936, 8166.38)(698.293, 2874.73)(699.2875, 1262.89)(700.27, 247.68)(1395.5353, 325.07)(743.3187, 526.76)(744.3188, 352.16)	96861.79	11946.43	98562.32	11092.28	87479.29	11465.28	85835.58	11889.17	-0.71	0.009322	0.019842	-0.15	0.638268	0.831817
COMPOUND_698_3517a5_478739	Rp	+	698.3517	5.48				(699.3311, 151.43)(350.1835, 8649.64)(350.6846, 3369.63)(351.1848, 875.02)(351.6862, 141.96)	26856.44	10189.64	24016.53	9450.935	35005	12871.58	35985.82	15304.86	0.33	0.244694	0.339945	0.18	0.598336	0.805251
COMPOUND_699_2819a10_087934	Rp	-	699.2819	10.09				(1433.5039, 157.29)(812.2636, 470.43)(813.264, 314.43)(814.2759, 210.94)(734.2467, 1766.7)(735.2461, 1010.91)(736.2536, 1706.04)(737.2564, 662.5)(738.2707, 2664.19)(739.267, 1664.47)(740.2578, 1149.84)(698.276, 7147.15)(699.2769, 3029.62)(700.2961, 2446.86)(1397.4895, 193.72)(1398.5009, 273.24)	89531.58	16811.33	84711.31	22920.16	71019.64	16108.03	76018.75	15402.98	-0.73	0.012454	0.025801	0.38	0.261123	0.572854
COMPOUND_699_3817a7_8125954	Rp	-	699.3817	7.81				(812.3547, 379.33)(698.3752, 22620.21)(699.3781, 9017.83)(700.3802, 2405.22)(701.3827, 498.13)(702.3908, 105.65)	16005.87	4960.95	15521.88	4838.97	58543.71	30792.37	73006.32	53726.24	1.35	3.18E-10	4.19E-09	-0.13	0.538941	0.776408
COMPOUND_699_4492a10_746508	Rp	+	699.4492	10.75				(722.439, 5812.83)(723.4416, 2731.17)(724.4498, 3908.94)(725.4681, 2080.26)(726.4383, 635.74)(700.4578, 17560.8)(701.4631, 8390.67)(702.4712, 24891.42)(703.4754, 11242.73)(704.4725, 6163.97)(705.4731, 2827.69)	281458.4	120399.8	256973.1	101022.2	88604	#DIV/0!	68153.5	43678.69	-1.07	1.02E-06	5.05E-06	0.34	0.147824	0.456493
COMPOUND_699_5184a12_620215	Rp	-	699.5184	12.62				(698.5114, 8914.34)(699.5157, 4114.28)(700.5178, 966.67)(701.5274, 160.81)	45465.67	16006.11	47626.98	18037.23	54188	13038.91	47579.35	19783.9	0.59	0.024868	0.047702	-0.59	0.054619	0.291472
COMPOUND_700_3152a10_646559	Rp	-	700.3152	10.65				(813.2717, 471.4)(735.2744, 839.8)(1445.6451, 113.43)(1446.6451, 136.55)(699.3088, 5004.1)(700.3088, 2178.16)(701.3096, 645.89)(702.2917, 177.42)(1399.582, 168.12)(1400.597, 182.8)(1401.5867, 130.61)	89368.64	12348.79	87090.93	15285.99	71001.71	12971.02	71081	14001.3	-1.12	4.08E-05	0.000139	0.30	0.316993	0.622922
COMPOUND_700_4976a9_614896	Rp	+	700.4976	9.61				(723.4864, 1304.96)(724.4891, 639.69)(725.4899, 289.61)(370.2357, 810.51)(370.7225, 49.48)(739.4877, 251.43)(739.9957, 194.89)(362.2501, 1605.39)(362.7535, 81.25)(363.2511, 488.16)(718.5316, 27904.59)(719.5343, 11193.16)(720.533, 3060.68)(721.5369, 758.31)(359.7707, 2464.21)(360.2684, 1380.4)(360.7732, 253.77)(701.5048, 2943.99)(702.5059, 1306.28)(703.5041, 481.08)(351.2521, 670.35)(351.7542, 86.74)(352.2491, 3411.49)	317438.4	69003.29	280582.4	49242.82	350071.3	56904.25	344158.8	81857.9	0.30	0.285759	0.384886	0.58	0.090132	0.356102

									1st trimester				3rd trimester									
									Control		Pre-eclampsia		Control		Pre-eclampsia		Time			Interaction		
Compound	Column	Ion_mode	Mass	Retention_	Identification	ID_level	MSMS_spectra	CompositeSpectrum	Mean	SD	Mean	SD	Mean	SD	Mean	SD	Value	raw p	FDR_p	Value	raw p	FDR_p
COMPOUND_700_5534a0_85892963	Hilic	+	700.5534	0.86				(723.542, 31714.26)(724.5455, 15006.13)(725.5572, 51306.89)(726.5609, 21731.3)(727.5739, 25477.74)(728.5784, 10674.77)(718.5934, 8382.53)(719.5969, 2479.93)(701.5596, 554417.0)(702.5636, 234702.4)(703.5747, 795642.1)(704.579, 337836.84)(705.5827, 76802.6)(706.5862, 14691.21)(707.5898, 2395.16)(708.5962, 425.62)(351.2837, 487.63)(351.7851, 127.51)(352.2952, 435.22)	7404810	1895624	7871857	2608108	8583046	2216883	8208371	2230865	0.50	0.089172	0.147334	-0.31	0.367119	0.669574
COMPOUND_700_556a11_700257	Rp	+	700.5560	11.70	SM d34:2	2	20 eV: 184.0735(100), 701.5584(25)	(1402.1117, 2461.05)(1403.1143, 2132.1)(1404.1187, 1231.23)(1405.1064, 615.61)(1440.1324, 182.55)(723.5427, 47503.5)(724.5454, 19802.28)(725.5499, 5038.56)(726.5454, 1014.39)(1424.0918, 667.55)(1425.0978, 596.26)(1426.1166, 446.56)(739.5161, 3101.27)(740.5215, 2246.82)(741.5234, 2276.08)(718.5595, 2341.27)(719.5669, 1257.14)(701.5603, 676346.4)(702.5644, 281432.38)(703.5671, 66395.42)(704.5685, 10195.03)(705.5738, 1498.18)	3388843	835956.5	3503888	1275485	3517332	923504.9	3358623	818711.2	0.07	0.790288	0.84749	-0.07	0.832594	0.926681
COMPOUND_701_4618a10_771543	Rp	+	701.4618	10.77				(724.4549, 7192.09)(725.4588, 3236.31)(726.4734, 3040.84)(740.4597, 1513.94)(719.4982, 6531.89)(720.4836, 3794.24)(721.4916, 2165.84)(702.4713, 17836.06)(703.4756, 8841.54)(704.4835, 9779.23)(705.4816, 4864.5)	203382.6	101428.2	174464.4	92422.98	150589.7	66701.45	90778.83	43767.34	-1.19	5.23E-08	3.57E-07	0.08	0.738527	0.888668
COMPOUND_701_5296a10_098768	Rp	+	701.5296	10.10				(702.5367, 6858.37)(703.5361, 2909.02)(351.7638, 70.51)(352.2479, 796.53)	46008.56	11968.22	40455.8	7695.957	53470.15	9706.096	52166.35	12774.94	0.44	0.106784	0.172261	0.55	0.098994	0.368908
COMPOUND_702_1608a9_162468	Rp	-	702.1608	9.16				(815.1586, 374.82)(816.1583, 200.79)(701.1539, 48303.08)(702.1568, 11882.92)(703.1553, 1737.6)(704.152, 424.99)	255684.8	29134.86	258780.4	22524.82	238360.9	16154.44	243050.3	23684.53	-0.68	0.020949	0.041131	0.03	0.935755	0.97184
COMPOUND_702_161a9_473247	Rp	-	702.1610	9.47				(737.1244, 677.85)(701.1533, 40844.41)(702.1571, 10564.34)(703.1584, 1182.52)(704.1613, 354.28)(747.1545, 408.82)(748.1654, 177.64)(749.1359, 561.41)	244656.4	30103.07	242703.6	32992.02	241217.6	28086.19	238965.3	23462.21	-0.11	0.721322	0.796283	-0.13	0.711192	0.871385
COMPOUND_702_161a9_376014	Rp	-	702.1610	9.38				(815.1345, 434.65)(816.1376, 365.57)(817.1287, 538.1)(701.1534, 45202.1)(702.1568, 10713.89)(703.1595, 1562.98)(704.1528, 269.31)(1403.317, 138.84)(747.1379, 592.61)(748.1457, 427.02)(749.1345, 862.59)	223413.6	19668.02	223319.3	18052.03	224694.2	15063.02	219477.6	15479.95	0.09	0.760988	0.827496	-0.30	0.417361	0.699344
COMPOUND_702_5718a12_023964	Rp	+	702.5718	12.02	SM d34:1	2	10 eV: 703.5757(100), 184.0735(27)	(1406.1422, 3154.3)(1407.1462, 2803.78)(1408.1521, 1428.89)(1409.1451, 695.87)(1410.16, 264.09)(1444.1129, 309.26)(1445.1304, 346.47)(1446.1108, 279.98)(725.5598, 38082.6)(726.5625, 16292.11)(727.5682, 4783.21)(728.5686, 1159.18)(1428.1646, 531.62)(1429.1449, 262.81)(1430.1495, 317.59)(1431.1731, 366.52)(1432.1722, 101.09)(741.5357, 5537.61)(742.5411, 2299.74)(743.5402, 1410.19)(703.5762, 747841.2)(704.5803, 306117.4)(705.583, 71694.56)(706.5834, 10203.77)(707.5844, 1484.76)(1423.193, 268.43)(1424.1917, 363.86)(1425.1874, 230.67)	5418515	1675107	5311868	2129967	5401172	994209.6	5138594	1498241	0.09	0.745031	0.814666	-0.09	0.802801	0.916921
COMPOUND_703_3249a10_391383	Rp	-	703.3249	10.39				(816.2822, 298.03)(817.2755, 282.83)(702.3182, 6578.68)(703.3204, 2409.29)(704.3178, 872.81)(705.3238, 191.05)	64867.07	13344.93	67781.98	13678.5	65526.71	10785.53	68938.5	13250.24	0.34	0.236422	0.331108	-0.19	0.588651	0.800992
COMPOUND_703_5082a9_365048	Rp	+	703.5082	9.37				(363.7527, 184.94)(364.2551, 625.77)(364.746, 29.12)(361.2681, 3633.09)(361.7783, 942.09)(362.2668, 1382.12)(704.5161, 17249.73)(705.5162, 7128.07)(706.5176, 2020.19)(707.5258, 350.98)(352.7639, 926.42)(353.2661, 3808.33)(353.7623, 105.78)	237163.7	52068.35	209310	49444.1	257985.5	50525.88	261556.3	73276.71	0.22	0.455052	0.560294	0.54	0.124252	0.418884
COMPOUND_703_5179a11_742987	Rp	+	703.5179	11.74	PC 30:1	2	20 eV: 184.0737(100), 704.5271(60), 499.1125(3)	(1408.0359, 375.66)(1409.0463, 233.28)(1410.0162, 133.5)(726.5058, 12924.29)(727.5099, 5810.29)(728.5221, 5433.41)(1430.0778, 254.99)(742.4852, 1047.29)(743.4943, 420.17)(744.4835, 80.92)(721.5825, 2271.25)(704.5237, 163256.08)(705.5267, 67617.2)(706.5292, 16133.56)(707.5311, 3403.69)	311906.1	148546.4	359848.9	181610.1	545782.9	163396.1	738889.2	402921.3	0.96	2.89E-05	0.000103	0.27	0.27446	0.583098
COMPOUND_704_584a12_243275	Rp	+	704.5840	12.24				(1410.2134, 299.04)(727.5277, 61.17)(728.5569, 1346.74)(1432.2102, 226.64)(705.5906, 22775.95)(706.591, 10078.83)(707.5939, 2322.21)(708.5992, 457.53)(1427.2242, 223.8)(1428.2289, 204.93)(1429.2169, 174.5)	58524.83	20715.07	59800.07	30126.85	53312.71	14010.69	51337.96	16454.18	-0.20	0.470279	0.573399	-0.04	0.908579	0.958319
COMPOUND_705_3442a10_567998	Rp	-	705.3442	10.57				(1445.6733, 182.09)(1446.6447, 154.17)(818.3112, 530.89)(819.2993, 391.06)(704.3345, 6573.55)(705.339, 2456.48)(706.3363, 604.43)(707.3389, 84.1)(1409.7028, 178.4)(1410.6826, 135.84)	27132.46	16192.24	24893.82	6726.496	16948.93	3400.961	33584.28	27866.96	-0.88	0.003174	0.007515	0.45	0.193953	0.505089
COMPOUND_705_4199a10_552607	Rp	-	705.4199	10.55				(1445.8651, 539.06)(818.3668, 512.22)(819.3732, 312.33)(1455.8931, 936.62)(1456.9003, 603.52)(704.4135, 9736.42)(705.4165, 3749.84)(706.4246, 2425.26)(707.4299, 697.6)(1523.8417, 445.99)(1524.8602, 553.72)(1525.8243, 730.6)(1526.8239, 360.16)	78016.71	27386.93	70320.39	29623.24	12359	#DIV/0!	#DIV/0!	#DIV/0!	-1.26	3.71E-09	3.41E-08	0.26	0.234161	0.540298
COMPOUND_705_5347a12_080148	Rp	+	705.5347	12.08	PC 30:0	2	20 eV: 184.0742(100), 706.5421(45)	(1412.079, 625.53)(1413.0729, 504.34)(1414.0846, 218.59)(728.5212, 7547.84)(729.5244, 2496.02)(730.5387, 17209.54)(1434.1039, 369.53)(1435.1118, 299.17)(1436.122, 172.24)(706.5406, 162599.44)(707.5435, 65630.13)(708.5454, 15520.92)(709.5459, 3046.68)(710.5429, 648.38)(1429.1304, 851.2)	711161.5	339273.4	707714.1	341988.1	821733.9	171783.3	952724	363663.3	0.52	0.0313	0.058495	0.17	0.537426	0.776408
COMPOUND_707_5067a12_868767	Rp	+	707.5067	12.87				(1415.9202, 332.56)(1416.9304, 435.09)(1453.9211, 315.79)(708.513, 341311.38)(709.5164, 165648.77)(710.5193, 39356.31)(711.5215, 6693.27)(712.5273, 1225.54)(713.5171, 185.99)(1432.9744, 182.18)	1173202	728799.9	881877.4	611240.2	931654.2	408944.5	1021035	595187.9	-0.27	0.351232	0.453281	0.48	0.161092	0.473602
COMPOUND_709_6457a2_89473	Hilic	+	709.6457	2.89				(710.6531, 3903.79)(711.6556, 509.66)(712.6519, 2934.01)(713.6572, 360.67)(714.6512, 910.58)(715.6515, 105.07)(716.6485, 189.06)	20095.57	4654.196	23843.07	5656.808	20860.86	4531.677	20980.95	5686.464	0.30	0.302962	0.403553	-0.70	0.043194	0.270201
COMPOUND_710_5108a11_688845	Rp	-	710.5108	11.69				(1456.0209, 78.16)(1466.0364, 312.99)(709.5047, 9138.62)(710.5076, 3537.61)(711.5037, 3578.11)(712.5, 1401.56)(713.5049, 362.16)(755.539, 261.34)(756.5133, 1094.14)(757.5283, 781.95)(1534.0665, 322.54)	97224	27745.75	103666.5	36397.55	127376.7	32848.94	127542.9	38518.46	0.25	0.37211	0.474245	-0.37	0.256536	0.56678
COMPOUND_711_5809a13_60106	Rp	+	711.5809	13.60				(712.5886, 17881.63)(713.5923, 8934.46)(714.5948, 2479.46)(715.5921, 734.55)	28711.49	15181.31	27502.09	16620.46	36994.57	17730.42	71824.39	44630.97	0.61	0.008355	0.018057	0.72	0.010014	0.153413
COMPOUND_713_3628a5_4239244	Rp	+	713.3628	5.42				(357.6894, 6069.55)(358.1913, 2114.95)(358.6898, 569.09)(359.1907, 117.91)	17545.09	3712.884	14448.19	3381.014	20520.2	7507.248	27780.4	9121.826	0.29	0.224508	0.318383	0.84	0.004024	0.094399
COMPOUND_713_498a12_015046	Rp	-	713.4980	12.02				(1471.986, 174.24)(1472.987, 147.53)(712.4912, 10304.19)(713.494, 4422.59)(714.4991, 2345.87)(715.5029, 678.7)(1425.9266, 128.53)	20981.97	6188.262	26390.54	16357.09	66571.38	37422.49	59096.28	27981.09	1.45	8.4E-11	1.3E-09	-0.06	0.797274	0.91372
COMPOUND_713_4988a12_034298	Rp	-	713.4988	12.03				(1471.9922, 198.44)(1473.0048, 172.07)(712.4909, 13276.89)(713.4942, 6179.83)(714.4973, 2197.39)(715.4984, 588.25)(716.4963, 73.97)(1425.9183, 110.14)	23635.25	6594.967	33218.59	14823.9	75694.43	31818.03	84409.18	35558.39	1.38	1.24E-10	1.83E-09	-0.04	0.832361	0.926681
COMPOUND_713_5024a12_0111265	Rp	+	713.5024	12.01				(1465.9199, 81.72)(736.4961, 1045.22)(737.4976, 510.48)(738.508, 20044.43)(1449.9257, 102.99)(714.5085, 11307.34)(715.5129, 5198.87)(716.5166, 1507.71)(717.5206, 450.25)	72115	34781.28	55065	18292.34	149010.2	73357.04	180737.6	90594.18	1.23	4.03E-13	2.39E-11	0.22	0.158819	0.468876
COMPOUND_713_5039a11_964554	Rp	+	713.5039	11.96				(1465.935, 253.74)(736.514, 977.86)(737.5061, 489.77)(738.5093, 13640.78)(1449.9316, 126.15)(714.5078, 15125.9)(715.5114, 6719.42)(716.5145, 1791.75)(717.5124, 497.82)	#DIV/0!	#DIV/0!	107515.8	69971.53	56601.18	41937.8	62263.25	62064.05	1.14	2.02E-09	2.03E-08	0.03	0.873639	0.946337
COMPOUND_714_2937a10_646723	Rp	-	714.2937	10.65				(1463.5586, 135.22)(1464.5554, 169.49)(713.2841, 5155.47)(714.2868, 3339.75)(715.2872, 1040.96)(1427.5626, 128.3)(1428.5977, 152.24)(759.3231, 724.57)	50045.27	8126.123	47518.48	9219.662	44922.31	6088.527	43682.13	8985.028	-0.56	0.042799	0.076374	0.29	0.37003	0.671661
COMPOUND_714_566a11_9216795	Rp	+	714.5660	11.92				(1430.1312, 410.67)(737.5313, 1070.12)(1452.0977, 385.42)(715.5757, 8223.74)(716.5784, 4143.6)(717.5816, 1063.18)(1447.1233, 570.03)(1448.1285, 520.13)	72092.78	20549.29	73713.09	25977.93	69054	15936.83	62001.4	19054.46	0.18	0.537767	0.636056	-0.38	0.271944	0.581594
COMPOUND_714_5673a0_84623057	Hilic	+	714.5673	0.85				(737.5571, 816.56)(738.5603, 408.93)(739.5716, 1555.09)(740.5748, 513.34)(741.5857, 463.63)(715.5745, 13026.75)(716.5776, 6049.16)(717.5901, 21453.37)(718.5932, 9272.78)(719.5961, 2623.3)(720.5938, 725.59)(721.5856, 250.53)	150962.9	39419.82	160759	54496.02	151489.9	45622.42	134656.2	36063.55	0.04	0.879839	0.920296	-0.63	0.057315	0.297402
COMPOUND_715_2816a10_377993	Rp	-	715.2816	10.38				(1465.4712, 167.18)(1466.4897, 132.37)(1467.4667, 142.16)(714.2761, 8320.4)(715.279, 2885.04)(716.2717, 4095.88)(717.2733, 1418.27)(718.2785, 1398.07)(719.2803, 690.77)(760.2664, 876.11)(761.2646, 644.64)(762.2534, 1321.32)(1429.4901, 197.05)(1430.5016, 219.9)	134041.5	12625.53	136815.8	14443.26	120941.7	14566.46	122999.3	19136.09	-0.78	0.004719	0.010681	-0.04	0.890483	0.953702

									1st trimester				3rd trimester									
									Control		Pre-eclampsia		Control		Pre-eclampsia		Time			Interaction		
Compound	Column	Ion_mode	Mass	Retention_	Identification	ID_level	MSMS_spectra	CompositeSpectrum	Mean	SD	Mean	SD	Mean	SD	Mean	SD	Value	raw p	FDR_p	Value	raw p	FDR_p
COMPOUND_715_4973a10_8657	Rp	-	715.4973	10.87				(1465.8983, 493.44)(1466.8768, 224.16)(1467.9196, 377.78)(1468.9204, 267.23)(1469.9242, 296.37)(750.4716, 1263.47)(751.4699, 344.01)(752.4865, 4293.9)(753.4885, 2087.93)(754.4972, 4825.28)(1475.9651, 404.67)(1476.9688, 526.81)(1477.9834, 298.35)(1478.9763, 483.09)(1479.9794, 433.56)(714.4909, 5480.19)(715.4936, 2686.21)(716.4929, 908.03)(717.49, 186.62)(1429.9536, 339.49)(1430.9744, 497.5)(1431.9642, 366.26)(1543.9741, 369.78)(1544.9672, 243.52)(1545.977, 321.84)(1546.9857, 263.31)(1547.9834, 330.56)	116898	37695.67	123101.5	37044.28	83854.77	25641.43	84604.56	19958.16	-0.97	0.000681	0.001843	0.05	0.878547	0.949827
COMPOUND_715_5139a12_343616	Rp	-	715.5139	12.34	PE 34:2	2	20 eV: 714.5073(100), 279.2328(42), 434.2677(4)	(1476.0791, 302.8)(714.5071, 107909.09)(715.5105, 47422.35)(716.5133, 11565.56)(717.5152, 2156.28)(718.5186, 345.35)(1429.9418, 34.07)(1430.9739, 75.87)(1544.0833, 630.62)(1545.0723, 689.63)(1546.0837, 2222.43)	74979.62	24523.94	95000.26	43092.66	309433.7	96381.98	399183.3	159105	1.35	2.11E-11	4.71E-10	0.12	0.536762	0.776357
COMPOUND_715_5197a12_3454075	Rp	+	715.5197	12.35	PE 34:2	2	10 eV: 716.5234(100), 575.5043(60), 43.0539(7)	(1432.0249, 158.21)(1433.0347, 163.95)(1434.0396, 66.14)(738.5188, 372.73)(739.5117, 134.06)(740.5241, 11598.35)(741.5268, 5326.76)(716.5259, 43660.29)(717.5295, 18535.76)(718.5322, 4860.35)(719.5371, 919.37)(1449.104, 220.65)	62922.96	33620.06	70378.14	37048.25	180807	53626.73	223226.9	77344.59	1.33	1.6E-09	1.67E-08	0.18	0.405165	0.6912
COMPOUND_716_5838a12_270833	Rp	+	716.5838	12.27				(1472.0872, 777.95)(1473.0739, 419.4)(739.5653, 865.11)(717.5902, 7845.07)(718.5936, 3510.9)(719.5874, 1082.77)	56076.94	18210	54732.96	23456.45	46601.85	7482.646	44939.36	11849.52	-0.60	0.026626	0.050686	0.06	0.851514	0.936901
COMPOUND_717_2988a10_644881	Rp	-	717.2988	10.64				(716.2915, 8555.3)(717.2944, 3108.4)(718.2872, 4034.82)(719.2894, 1337.02)(720.2942, 1259.57)(721.301, 563.86)(1433.5525, 178.5)(762.2915, 817.14)(763.2872, 528.45)(764.2858, 527.2)	94457.06	11976.07	95612.83	15568.04	78878.07	20731.72	78629.37	19403.1	-0.81	0.001112	0.002864	-0.09	0.733927	0.888604
COMPOUND_717_5246a9_619151	Rp	+	717.5246	9.62				(1458.017, 56.59)(370.7593, 250.42)(371.2573, 953.9)(371.7614, 34.59)(387.2545, 276.8)(387.7573, 57.7)(388.2487, 214.08)(368.2843, 3357.13)(368.7865, 1419.47)(369.2834, 1167.55)(369.786, 119.25)(718.5316, 27904.59)(719.5343, 11193.16)(720.533, 3060.68)(721.5369, 758.31)(722.5408, 161.91)(359.7707, 2365.95)(360.2683, 1337.12)(360.7732, 241.08)	307257	75627.83	266788.9	52464.22	335087.1	56285.27	334457.1	84663.06	0.27	0.337057	0.437747	0.63	0.064599	0.307286
COMPOUND_717_5294a12_769517	Rp	-	717.5294	12.77	PE 16:0_18:1	1	10 eV: 716.5214(100), 281.2485(6)	(716.5225, 10132.53)(717.526, 4253.1)(718.5297, 1298.79)(719.5328, 358.23)(720.5326, 65.97)(1434.0535, 51.28)(762.5127, 1066.2)(763.5135, 560.92)	24619.58	8582.44	28905.24	11776.89	73656.86	28953.85	101407.4	38328.46	1.34	3.2E-10	4.19E-09	0.17	0.41194	0.694898
COMPOUND_717_5337a11_952413	Rp	+	717.5337	11.95	PC 31:1	2	20 eV: 184.0735(100), 718.5437(90), 494.0215(5)	(740.5168, 5942.38)(741.5283, 2387.95)(718.539, 40110.43)(719.542, 17345.4)(720.5446, 4494.77)(721.5435, 1052.42)	85071.7	35271.86	92989.96	46583.39	110666.4	30534.76	146327.1	72917.07	0.78	0.000291	0.000837	0.43	0.073932	0.327102
COMPOUND_717_5339a12_766433	Rp	+	717.5339	12.77				(740.5228, 1083.89)(741.5274, 523.71)(735.5742, 14396.8)(736.5765, 3665.19)(737.5806, 833.8)(718.5391, 13070.02)(719.5422, 5838.5)(720.5455, 1812.34)(721.5478, 269.9)	40357	#DIV/0!	47369.8	16307.81	78440.73	21186.28	107568.6	48626.83	0.96	4.96E-10	5.97E-09	0.48	0.0028	0.074044
COMPOUND_717_5671a12_931534	Rp	+	717.5671	12.93				(1474.1708, 92.47)(740.5569, 795.13)(741.5553, 548.7)(742.5725, 3109.6)(718.5752, 15418.84)(719.5791, 6918.07)(720.5809, 1943.33)(721.5815, 377.86)	67749.88	32289.55	57134.33	29358.87	56827	15173.15	62625.6	23208.2	-0.27	0.360402	0.462728	0.10	0.780485	0.909288
COMPOUND_717_5674a12_571076	Rp	+	717.5674	12.57				(1474.064, 167.57)(740.5562, 1833.53)(741.5539, 1394.08)(718.575, 20765.52)(719.5783, 9479.78)(720.5804, 2552.17)(721.5792, 624.43)(1453.2264, 296.12)	149858.5	50866.98	140861	52685.75	134304.9	42081.22	126073.7	47385.58	-0.44	0.078495	0.131233	0.14	0.633405	0.828355
COMPOUND_717_9053a11_05774	Rp	+	717.9053	11.06				(735.9416, 566.07)(736.9649, 67.68)(737.9529, 630.34)(718.9101, 17939.2)(719.9077, 4006.71)(720.9121, 2375.76)	91296.92	57809.11	91837.97	69875.81	86737.54	23951.75	88324.09	23547.98	0.40	0.152624	0.231454	-0.13	0.691616	0.857326
COMPOUND_718_525a11_379731	Rp	-	718.5250	11.38				(831.5098, 778.7)(717.5178, 20135.42)(718.5211, 8800.75)(719.5252, 2397.55)(720.5255, 533.85)(721.5352, 100.12)(1550.0594, 203.07)(1551.0734, 181.39)(1552.0566, 372.74)(1553.0729, 312.02)	213835.9	56163.89	218910.1	60691.96	212635.4	46188.3	231164.7	56871.41	-0.70	0.00579	0.012923	0.06	0.843717	0.93246
COMPOUND_719_1516a9_161084	Rp	-	719.1516	9.16				(718.1446, 5101.83)(719.1467, 1519.8)(720.1409, 351.43)	17832.02	3423.469	17267	3166.995	16009.57	2704.825	17138.86	4282.706	-0.51	0.094291	0.154883	0.35	0.335729	0.639647
COMPOUND_719_5536a12_241971	Rp	+	719.5536	12.24				(1440.1342, 58.04)(1478.1163, 1320.34)(742.5303, 7558.0)(743.5357, 1479.54)(1462.1348, 415.23)(720.5551, 9702.9)(721.5587, 4145.1)(722.5375, 2070.46)(1457.1433, 137.46)	52008.82	27817.67	53754.49	29738.58	67949.55	14826.23	73798.36	25941.96	0.27	0.282295	0.38159	0.49	0.095145	0.36381
COMPOUND_719_5831a13_133488	Rp	+	719.5831	13.13				(1440.2114, 153.86)(742.5709, 2878.21)(743.5739, 1406.09)(720.5902, 9935.82)(721.5936, 4328.3)(722.5957, 1197.68)(723.6016, 267.18)	45569.54	26453.3	49123.13	28336.01	41375.77	17590.64	45660.52	28386.52	-0.11	0.689141	0.773266	0.14	0.667713	0.846185
COMPOUND_720_5402a11_690141	Rp	-	720.5402	11.69				(833.5263, 4220.5)(719.5337, 91220.87)(720.5366, 38033.39)(721.5397, 9964.86)(722.5407, 996.92)(723.5416, 49.99)(1554.0687, 1575.18)(1555.0742, 1399.55)	981455	256322.8	1078396	372634.2	1085899	241661.5	1083365	302282.9	0.40	0.145596	0.222595	-0.36	0.274045	0.583098
COMPOUND_721_5045a12_198856	Rp	-	721.5045	12.20				(756.4966, 511.45)(757.4969, 163.15)(720.4963, 12580.25)(721.5002, 5898.41)(722.5029, 1351.81)(723.5043, 356.81)(724.5198, 74.46)	40495.65	24998.42	38597.04	24407.97	33661.64	15439.93	36898.63	18946.74	-0.16	0.5334	0.632881	0.20	0.506653	0.755617
COMPOUND_721_5228a12_871885	Rp	+	721.5228	12.87				(1443.9922, 160.57)(722.529, 650846.25)(723.5329, 308215.94)(724.5355, 74528.59)(725.5376, 13408.38)(726.5417, 2715.2)	4595190	535556.7	4482359	436826.7	4105132	456796.6	4040573	541576.4	-0.85	0.002423	0.005918	0.04	0.89063	0.953702
COMPOUND_722_1006a9_06127	Rp	-	722.1006	9.06				(835.0736, 339.56)(836.083, 278.64)(721.0926, 1551.27)(722.0887, 578.52)(723.0867, 25730.86)	116912.4	18869.79	127689.5	18202.61	103204.7	12619.83	106271	14075.47	-0.69	0.011405	0.02376	-0.39	0.223049	0.531262
COMPOUND_723_2995a10_647959	Rp	-	723.2995	10.65				(1481.5612, 97.95)(836.2722, 230.05)(837.2916, 61.67)(838.265, 136.33)(758.275, 190.98)(759.28, 121.17)(760.2737, 196.49)(722.2931, 5950.27)(723.296, 2436.99)(724.2906, 1170.44)(725.2896, 350.8)(1445.6132, 79.53)(1446.6401, 97.21)(768.294, 1196.78)(769.2962, 453.95)	88217.3	16559.44	88565.83	17870.41	95963.21	14912.71	92328.88	15784.51	0.47	0.107899	0.173935	-0.24	0.481093	0.743153
COMPOUND_723_5057a11_708447	Rp	-	723.5057	11.71	PC 28:0	2	20 eV: 662.4759(100), 227.2026(22), 722.5332(10)	(1482.0305, 161.89)(836.4983, 296.82)(837.4868, 160.83)(838.5161, 964.35)(758.4858, 157.47)(759.5064, 409.98)(722.4972, 11172.48)(723.5032, 5146.25)(724.5076, 1559.06)(725.5045, 418.63)(1445.9873, 71.27)(1447.0232, 300.8)(1448.0261, 220.26)	77572.16	50888.17	99009.61	74142.89	144303.9	86693.1	156252.7	89449.2	0.57	0.026169	0.0499	0.06	0.841526	0.93246
COMPOUND_723_5191a12_532415	Rp	-	723.5191	12.53	PE 36:5e	2	20 eV: 722.513(100), 303.2328(52), 436.285(7)	(836.4981, 313.78)(837.5013, 270.25)(838.5215, 125.35)(758.4916, 442.12)(759.4944, 228.77)(1492.0677, 127.35)(722.5123, 348922.06)(723.5158, 153524.8)(724.518, 38930.79)(725.5196, 8143.8)(726.5241, 1494.54)(1446.0023, 159.22)(1446.9893, 53.16)(1448.0072, 84.21)	334914.6	269074.2	361978.8	267481.5	452061.1	296055.7	451936	237630.7	0.56	0.033106	0.061413	-0.05	0.871739	0.945212
COMPOUND_723_5206a12_5318985	Rp	+	723.5206	12.53				(1448.0092, 296.65)(1485.9493, 296.89)(746.5107, 1417.32)(747.5122, 1040.0)(748.5275, 1731.8)(1469.9607, 363.56)(1470.9558, 223.8)(762.4776, 416.55)(763.4803, 211.31)(741.5465, 3720.84)(724.528, 28469.11)(725.5344, 14489.46)(726.5361, 3581.65)(727.5473, 1007.5)(1465.0133, 233.36)	126580.6	51753.74	111619.1	47391.72	117128.3	54599.58	112453.7	37056.46	-0.16	0.540598	0.639069	0.20	0.495959	0.751527
COMPOUND_723_9038a11_00311	Rp	+	723.9038	11.00				(746.9236, 3138.41)(762.8614, 5396.36)(763.8581, 3491.21)(764.8621, 1239.26)(741.9082, 4096.21)(724.9119, 18257.61)(725.9082, 7649.01)(726.9041, 2411.86)	71932.9	42129.23	64446.2	50770.2	51866.08	29550.76	68431.9	51930.44	-0.12	0.673401	0.760149	0.26	0.442418	0.717814
COMPOUND_724_0943a9_296961	Rp	-	724.0943	9.30				(759.071, 212.11)(760.0639, 184.54)(1493.2368, 118.53)(723.0875, 10831.49)(724.0892, 3582.32)(725.0904, 996.26)(726.095, 153.23)(1447.1624, 99.29)	69570.64	11152.63	75226.02	10619.14	66929.07	9489.962	67521.32	10563.37	-0.25	0.376458	0.477624	-0.42	0.221903	0.531023
COMPOUND_724_388a10_8601265	Rp	+	724.3880	10.86				(747.403, 8289.9)(742.4026, 2125.24)(743.3999, 1818.4)(725.3986, 5990.24)(726.3998, 3024.64)(727.3932, 10425.22)(728.3941, 5248.48)(729.3808, 3778.71)	23166.23	8574.473	19858.82	7897.725	76685.43	51942.72	87008.8	22121.05	1.25	1.05E-09	1.15E-08	0.06	0.787987	0.909997
COMPOUND_725_0972a9_061598	Rp	-	725.0972	9.06				(1485.1694, 125.8)(760.0672, 390.95)(761.0562, 308.44)(762.0728, 226.24)(724.0896, 7480.25)(725.09, 1930.12)(726.0955, 467.99)(727.0811, 78.15)	50745.49	8788.473	54693.13	8412.752	49242	7756.754	49524.54	6761.588	-0.50	0.09089	0.149952	-0.10	0.775227	0.905077
COMPOUND_725_5345a12_725944	Rp	-	725.5345	12.73				(838.5111, 293.08)(839.5211, 193.93)(724.5275, 11151.45)(725.5317, 5350.51)(726.5351, 1378.76)(727.5378, 392.56)(770.5326, 1580.7)(771.533, 799.1)(772.5272, 880.03)	80989.13	27872.2	87330.2	34219.88	89093.43	19942.38	82124.4	21318.15	0.36	0.205687	0.296557	-0.42	0.212645	0.520001
COMPOUND_725_5381a12_703869	Rp	+	725.5381	12.70				(748.5262, 901.85)(749.5296, 694.04)(1474.0042, 99.86)(764.4728, 272.36)(765.4879, 110.79)(726.544, 8369.26)(727.5478, 3747.26)(728.5527, 1175.75)(729.5434, 194.46)(1469.1357, 358.25)	64818.49	31742.2	58459.46	28918.32	71487.64	21462.69	54479.45	20134.67	0.39	0.109336	0.175875	-0.61	0.031149	0.237791

									1st trimester				3rd trimester									
									Control		Pre-eclampsia		Control		Pre-eclampsia		Time			Interaction		
Compound	Column	Ion_mode	Mass	Retention_	Identification	ID_level	MSMS_spectra	CompositeSpectrum	Mean	SD	Mean	SD	Mean	SD	Mean	SD	Value	raw p	FDR_p	Value	raw p	FDR_p
COMPOUND_726_5682a11_813656	Rp	+	726.5682	11.81				(1454.188, 146.78)(1492.045, 463.71)(749.5317, 1871.16)(750.5429, 1266.26)(751.5463, 1035.8)(744.5719, 1283.04)(745.5787, 803.36)(746.5922, 854.31)(727.5755, 20420.08)(728.5791, 11248.04)(729.5814, 3553.34)(1471.2064, 456.48)	164522.9	48469.36	175957	64406.46	176308.1	61523.76	165618.7	48064.33	0.18	0.483741	0.585062	-0.48	0.111517	0.397302
COMPOUND_727_5157a11_618334	Rp	+	727.5157	11.62				(1456.033, 531.89)(1494.0156, 319.51)(750.5402, 4468.43)(751.5505, 2488.06)(1478.0322, 50.12)(1479.0782, 306.96)(728.5221, 13731.23)(729.5252, 6237.5)(730.5386, 106435.63)(731.5422, 47029.83)(1473.0588, 213.62)	104250.5	58375.21	122422.9	73122.87	207638.1	109854	187362.5	80108.24	1.13	2.28E-05	8.36E-05	-0.26	0.369208	0.671613
COMPOUND_727_5493a13_368302	Rp	-	727.5493	13.37				(840.5301, 715.96)(841.534, 411.46)(842.5355, 799.73)(843.5376, 401.16)(726.5432, 5051.13)(727.5461, 2282.1)(728.5498, 803.71)(729.5522, 201.12)	43735.35	16526.7	50489	22212.8	49193.93	11628.39	44731.34	17937.3	0.47	0.099101	0.161604	-0.63	0.060528	0.302235
COMPOUND_728_5831a0_8363836	Hilic	+	728.5831	0.84				(751.5727, 11965.02)(752.5755, 5816.54)(753.5876, 8783.52)(754.5911, 3968.61)(755.6049, 8038.11)(746.5977, 2826.09)(747.5879, 1503.18)(748.5729, 432.25)(729.5912, 145731.42)(730.5944, 67866.17)(731.605, 68051.16)(732.6085, 27643.09)(733.612, 7373.01)(734.6147, 1410.92)(735.6246, 427.05)(365.2992, 275.14)(365.798, 20.25)	1379251	381935.3	1467393	466881.3	1501340	462471.8	1472812	460912.4	0.25	0.395615	0.499958	-0.25	0.458038	0.727948
COMPOUND_728_5854a12_142221	Rp	+	728.5854	12.14	SM d36:2	2	20 eV: 184.0729(100), 729.5946(28)	(1458.1833, 424.82)(1459.1611, 562.16)(1460.1631, 384.68)(1496.0734, 5401.72)(751.573, 10728.49)(752.5726, 5910.76)(753.5739, 2001.06)(1480.1365, 157.6)(1481.1244, 285.16)(1482.1196, 197.11)(767.5397, 5645.48)(729.5929, 202307.62)(730.5954, 91009.86)(731.5981, 22392.93)(1475.1733, 133.57)(1476.2089, 169.98)(1477.2091, 113.26)	933099.2	285836.4	897211.6	366108.4	892635.7	274777.4	863040.5	280008.6	-0.11	0.703677	0.783952	0.04	0.897205	0.954419
COMPOUND_729_533a11_862355	Rp	+	729.5330	11.86	PC 14:0_18:2	1	20 eV: 184.0737(100), 730.5359(75), 104.1071(3), 451.2971(1)	(1460.0708, 4809.18)(1461.0725, 4429.33)(1462.076, 2094.02)(1463.0789, 818.38)(1464.0867, 379.85)(1498.08, 517.01)(752.5227, 44956.04)(753.5259, 19559.12)(1482.052, 1193.93)(1483.057, 1172.29)(1484.0692, 1971.78)(1485.0775, 1430.76)(1486.0756, 1003.06)(1487.0812, 787.3)(1488.0872, 631.18)(768.5111, 1666.24)(769.5086, 1284.46)(770.5283, 1367.07)(730.5407, 387487.47)(731.5445, 164436.6)(732.547, 39038.51)(733.5488, 7561.62)(734.5517, 1493.77)(735.5558, 266.81)(1477.1459, 191.48)	2062161	742333.5	2414727	857698	3674738	1322215	3678019	1276568	1.27	1.89E-07	1.11E-06	-0.33	0.194471	0.505132
COMPOUND_730_0318a12_024141	Rp	+	730.0318	12.02				(1499.08, 880.95)(748.0358, 548.39)(731.038, 10299.61)(732.0391, 2823.5)(733.0378, 995.32)(734.0269, 162.65)(1478.1075, 770.49)(1479.1123, 906.69)(1480.1135, 708.17)	#DIV/0!	#DIV/0!	#DIV/0!	#DIV/0!	#DIV/0!	#DIV/0!	#DIV/0!	#DIV/0!	-0.10	0.724184	0.798344	0.00	0.993505	0.997491
COMPOUND_730_5978a12_575648	Rp	+	730.5978	12.58	N-Stearoyl-D-sphingomyelin	1	20 eV: 184.0738(100), 731.6159(21), 104.1027(4), 590.5249(3)	(1462.206, 179.16)(1463.2266, 267.1)(1464.2318, 186.05)(1465.2133, 221.68)(1500.1243, 780.48)(1501.1353, 672.83)(1502.1489, 1103.61)(753.5848, 5414.95)(754.5852, 2678.38)(755.5948, 1019.1)(1484.123, 604.13)(1485.122, 560.49)(731.6068, 38584.87)(732.6087, 17978.63)(733.6107, 4507.28)(734.6129, 342.45)(1479.206, 257.59)(1480.2054, 208.15)(1481.1923, 200.01)(1482.1906, 96.04)	341419.9	113208.6	347000.2	141050.3	326137.1	100432.5	322446.3	114598.9	-0.07	0.817885	0.872049	-0.11	0.748274	0.891783
COMPOUND_731_5085a11_177976	Rp	-	731.5085	11.18				(1497.992, 232.75)(1499.0273, 262.77)(766.4652, 5903.32)(767.4678, 2954.71)(768.4698, 1030.39)(1507.9965, 534.62)(1508.998, 660.63)(1510.0013, 474.33)(730.502, 56249.29)(731.505, 24732.77)(732.5062, 7631.18)(733.4984, 2013.24)(1461.9976, 368.51)(1462.9973, 567.44)(1464.0038, 714.58)(1465.0035, 823.19)(1466.0052, 580.71)	103187.9	28107.66	119460.5	41648.58	180205.3	64704.19	224558.1	100724.8	-0.63	0.010662	0.022439	-0.42	0.149132	0.45818
COMPOUND_731_5415a9_853401	Rp	+	731.5415	9.85				(1464.1158, 63.68)(1486.1377, 74.92)(377.7692, 316.65)(378.2676, 1338.33)(378.7781, 66.31)(770.537, 1752.6)(375.2911, 2781.55)(375.793, 1180.14)(376.2861, 2410.21)(376.7963, 76.77)(386.29, 507.37)(386.7955, 127.85)(732.5469, 21003.56)(733.5494, 8500.72)(734.5482, 1106.35)(366.7785, 2081.9)(367.2819, 1130.66)(367.7829, 232.63)	235920.4	64020.62	202242.8	41829.07	272375.8	42167.38	270329.8	70017.29	0.44	0.113225	0.181482	0.54	0.107417	0.38954
COMPOUND_731_5497a12_171762	Rp	+	731.5497	12.17	PC 32:1	2	40eV: 184.0733(100), 86.0963(4)	(1464.1012, 12294.17)(1465.105, 10954.99)(1466.107, 5057.69)(1467.1075, 1982.37)(1468.1073, 537.48)(1469.122, 198.64)(1470.1488, 36.87)(754.538, 17590.37)(755.541, 7661.45)(756.5562, 247497.5)(1486.1024, 1769.04)(1487.116, 1562.33)(1488.1029, 2662.06)(1489.1052, 2024.98)(1490.1141, 1817.13)(1491.1125, 1338.9)(1492.1172, 771.78)(1493.1168, 386.79)(1494.0981, 245.42)(732.5555, 830709.75)(733.5597, 348721.34)(734.5625, 83366.52)(735.5646, 14678.25)(736.5666, 2523.83)(737.561, 433.04)	3786019	1359359	3762709	1927352	6137051	1812338	8278111	3408405	0.89	6.5E-05	0.000211	0.60	0.01762	0.194764
COMPOUND_733_5197a9_113487	Rp	+	733.5197	9.11				(1468.0625, 57.36)(1505.9615, 67.31)(764.5262, 554.73)(765.0149, 305.72)(376.281, 3205.74)(376.7851, 704.61)(377.279, 1294.63)(734.5264, 5876.5)(735.5266, 2689.98)(736.5265, 725.58)(737.5367, 185.34)(367.7686, 987.56)(368.2692, 552.89)(368.7709, 138.54)(1485.1165, 53.82)	97625.87	27370.29	87901.46	22618.48	105096.8	19324.99	99833.16	27130.94	0.17	0.565207	0.661907	0.45	0.192143	0.505089
COMPOUND_733_5645a12_636991	Rp	+	733.5645	12.64	PC 16:0_16:0	1	10 eV: 734.5685(100), 184.074(16)	(1468.142, 518.88)(1469.1357, 418.58)(1470.1539, 349.62)(1471.1361, 176.73)(1506.1434, 1313.43)(756.5535, 16865.63)(757.5569, 7401.85)(1490.1252, 421.86)(734.571, 71559.41)(735.5741, 30629.69)(736.5761, 7517.03)(737.5767, 1676.46)(738.5713, 365.47)(739.5713, 50.42)	800363	325728.3	814631.8	409732.3	795206.6	200400.1	929792.7	346716.8	0.08	0.771264	0.833439	0.41	0.225889	0.533671
COMPOUND_735_5388a12_870843	Rp	+	735.5388	12.87				(1509.991, 156.04)(736.5394, 7865.36)(737.5434, 3830.06)(738.5411, 1793.33)(739.5372, 556.11)	64137.47	9353.328	60828.02	9030.602	55085.29	7475.654	55429.15	8596.102	-0.80	0.004453	0.010139	0.45	0.168038	0.480454
COMPOUND_736_5279a11_778821	Rp	-	736.5279	11.78				(1508.018, 199.92)(1509.0118, 130.35)(1509.9768, 219.41)(1518.0739, 301.13)(1519.1025, 484.17)(1520.1025, 469.09)(735.5245, 14054.51)(736.52, 7991.92)(737.5187, 6488.74)(781.5492, 1136.09)(1586.073, 324.78)(1587.1077, 495.14)	217339.3	57781.27	241380.4	72946.09	257305.8	63385.02	262402.3	61501.83	-0.49	0.063055	0.108729	0.12	0.692104	0.857326
COMPOUND_737_3602a13_429183	Rp	-	737.3602	13.43				(736.3535, 6545.75)(737.3566, 2961.15)(738.3511, 4150.92)(739.354, 1708.92)(740.3504, 1041.92)(741.3499, 343.94)(742.3429, 158.9)(1473.7129, 122.91)	27248.96	11914.37	27906.8	13635.46	27829.07	8545.028	52166.23	25061.34	0.27	0.23878	0.333581	0.87	0.001783	0.061415
COMPOUND_737_5032a11_989126	Rp	+	737.5032	11.99				(1476.0896, 360.73)(1477.0658, 200.66)(760.5006, 598.41)(761.4981, 86.96)(762.518, 321.77)(776.4389, 496.15)(738.5086, 7428.95)(739.5126, 3328.47)(740.5229, 1578.97)(741.5289, 492.45)(1493.0782, 632.36)	38950.1	17712.04	42628.95	22809.41	93206.31	28075.06	112898.1	49723.87	1.19	8.44E-10	9.56E-09	0.29	0.138454	0.44479
COMPOUND_738_5093a0_85949695	Hilic	+	738.5093	0.86				(761.4953, 1366.03)(739.5159, 6201.25)(740.5202, 3349.42)(741.5305, 9835.75)(742.5385, 5159.82)(743.5393, 1892.21)(744.5539, 1297.56)	78591.97	21494.39	90283.91	25783.52	91883.1	18125.93	95995.36	20250.61	0.32	0.219695	0.31355	0.24	0.446933	0.720983
COMPOUND_738_5423a12_0963545	Rp	-	738.5423	12.10				(1512.0677, 279.85)(1513.0886, 376.75)(1514.0793, 394.12)(1522.0833, 950.05)(1523.0825, 589.17)(1524.0706, 363.48)(1525.0396, 121.28)(737.535, 10688.31)(738.5334, 3991.83)(739.5292, 5333.28)(740.5207, 4161.19)(741.5225, 1634.77)(742.5109, 927.12)(743.5166, 368.67)(1476.1368, 445.29)(783.5398, 304.76)(784.5505, 1368.78)(1590.0673, 605.64)(1591.0891, 659.05)(1592.0824, 958.79)	209658.4	53987.73	209194.2	60332.25	240834.3	50782.21	243935.5	47933.55	-0.12	0.660362	0.748056	-0.40	0.221354	0.531023
COMPOUND_739_5143a12_282022	Rp	-	739.5143	12.28				(852.4661, 271.86)(853.4769, 138.29)(854.4745, 168.19)(1524.081, 931.8)(738.5074, 16693.48)(739.5106, 7813.27)(740.5141, 2286.6)(741.515, 516.96)(742.5018, 42.11)(1478.0275, 432.3)(1479.0394, 369.84)(1480.0575, 261.61)	135605.1	39257.84	151750.3	54322.36	435699.4	139734	551810.6	178694.7	1.33	6.68E-11	1.1E-09	0.26	0.185196	0.499463
COMPOUND_739_5174a12_2786	Rp	+	739.5174	12.28	PE 16:0_20:4	1	20 eV: 599.5108(100), 313.2788(7), 740.535(6), 95.0878(4)	(1480.0438, 590.98)(1481.0404, 440.64)(1482.0619, 301.52)(1483.0471, 71.11)(762.5074, 2872.34)(763.5104, 1237.84)(778.4539, 392.01)(779.4625, 355.66)(757.5568, 10231.63)(740.5258, 80403.89)(741.5291, 36202.14)(742.5325, 9172.67)(743.5359, 1964.46)(1497.0763, 3942.52)	93238.83	26753.12	103164.7	41229.96	286070	74939.54	345734.1	93522.65	1.26	5.91E-10	7.01E-09	0.44	0.034661	0.249266
COMPOUND_740_9191a11_136369	Rp	+	740.9191	11.14				(779.8794, 5933.94)(780.8653, 1290.7)(781.8766, 2832.77)(782.8818, 1150.92)(758.9484, 2712.17)(741.9291, 12151.66)(742.9184, 4434.79)(743.9316, 1800.33)(1499.9044, 268.79)	60420.55	52851.75	60084.93	58627.53	71149.64	36309.48	88754.41	33200.94	0.24	0.376276	0.477624	0.13	0.685761	0.853695

									1st trimester				3rd trimester				Time			Interaction		
									Control		Pre-eclampsia		Control		Pre-eclampsia							
Compound	Column	Ion_mode	Mass	Retention_	Identification	ID_level	MSMS_spectra	CompositeSpectrum	Mean	SD	Mean	SD	Mean	SD	Mean	SD	Value	raw p	FDR_p	Value	raw p	FDR_p
COMPOUND_741_4565a0_74391323	Hilic	+	741.4565	0.74				(764.459, 456.12)(765.4618, 246.67)(766.4763, 530.07)(759.4813, 417.16)(742.4668, 13964.59)(743.4705, 6411.64)(744.479, 7601.09)(745.4844, 3178.43)(746.5001, 1908.99)	113944.3	45200.37	105648.3	44985.58	#DIV/0!	#DIV/0!	#DIV/0!	#DIV/0!	-1.25	4.8E-09	4.24E-08	0.12	0.569303	0.788601
COMPOUND_741_5217a10_943338	Rp	-	741.5217	10.94				(1517.9631, 161.76)(1518.9701, 166.56)(1519.9526, 204.88)(1520.9694, 166.3)(1521.9978, 121.93)(854.5196, 7897.19)(855.5234, 3947.43)(776.4873, 1955.49)(777.491, 1101.9)(1527.9801, 163.67)(1528.9911, 177.4)(1529.9797, 208.77)(1530.9877, 232.73)(740.5055, 3526.14)(741.5095, 1824.22)(742.4917, 2064.57)(743.489, 1093.9)(1481.9828, 214.66)(1482.9655, 247.58)(1483.9377, 344.73)(1596.0605, 397.32)	146171.6	52259.02	119530	33446.64	59184.18	4576.772	66953.52	25917.04	0.17	0.511655	0.612246	0.43	0.164565	0.47785
COMPOUND_741_5299a12_466389	Rp	-	741.5299	12.47				(1518.0876, 235.56)(1528.1086, 431.19)(1529.0872, 127.27)(1530.0914, 1296.79)(1531.0975, 1178.14)(1532.1025, 1631.72)(1533.1082, 1118.31)(740.5224, 62852.03)(741.5258, 28007.84)(742.5319, 10680.2)(743.5369, 2914.9)(744.5401, 654.83)(1482.0841, 794.28)(1483.0914, 517.53)(1484.0916, 247.84)(1596.0323, 85.39)(1597.0518, 60.16)	158458.6	64447.21	165200	67433.36	307227.1	78602.56	337022.6	122351.3	1.23	4.35E-08	3.03E-07	0.09	0.683985	0.853695
COMPOUND_741_5331a11_827901	Rp	+	741.5331	11.83				(1484.0762, 1202.82)(1485.0748, 926.82)(1486.082, 1135.5)(1487.0859, 831.05)(1522.0565, 278.86)(764.5191, 3368.56)(765.5232, 1533.55)(1506.0946, 339.96)(1507.0807, 490.83)(742.5391, 10026.49)(743.5418, 4810.33)(744.5441, 1579.14)(745.5257, 416.53)(1501.1027, 195.99)	56844.67	25286.92	69139.24	37812.4	89319.42	45828.82	80434.11	35081.84	0.88	0.000239	0.000702	0.10	0.718561	0.876415
COMPOUND_741_5358a12_454743	Rp	+	741.5358	12.45	PE 36:3	2	10 eV: 742.542(100), 601.5214(42), 412.2839(5), 80.6082(2)	(1484.0807, 417.37)(1485.0726, 234.83)(1486.0717, 234.47)(764.5225, 4473.33)(765.5271, 2122.68)(742.54, 56559.71)(743.543, 25878.83)(744.5474, 8012.49)(745.5525, 2006.63)(1501.1123, 1781.42)(1502.121, 1250.02)(1503.1324, 706.15)	135881.7	32171.24	160873.6	49197.08	316528	83320.56	371625.4	129611.1	1.29	3.1E-13	1.98E-11	0.19	0.239191	0.5441
COMPOUND_741_5698a12_588807	Rp	+	741.5698	12.59	PC 34:3e	2	10 eV: 742.5762(100), 184.0758(12), 601.5229(4)	(1484.1216, 966.14)(1485.12, 1015.89)(1486.131, 641.47)(1487.1416, 352.53)(1522.1718, 19797.21)(1523.1733, 6617.17)(1524.165, 2409.95)(764.5569, 16908.52)(765.5596, 8454.84)(766.5752, 82104.55)(1506.1367, 1044.25)(1507.1218, 280.93)(1508.1172, 1510.57)(742.5758, 276611.78)(743.5791, 126787.34)(744.5822, 34590.27)(745.5855, 7174.06)(1501.1267, 749.2)	2240311	685058.4	2059151	797667.6	1848022	546725.1	1826567	606346.8	-0.49	0.074599	0.125558	0.17	0.597527	0.805251
COMPOUND_743_5453a12_994272	Rp	-	743.5453	12.99				(742.5382, 6802.22)(743.5411, 3083.68)(744.5518, 3563.15)(745.5549, 1946.26)(746.5547, 580.74)(1486.091, 242.44)(1487.0936, 135.51)(1488.1095, 82.87)	120728.8	44162.22	148282.1	59751.55	262888.8	94984.69	319737.6	121302.3	1.32	2.05E-08	1.58E-07	-0.04	0.859495	0.941431
COMPOUND_743_5453a12_921009	Rp	-	743.5453	12.92				(742.5381, 14835.32)(743.5411, 6702.18)(744.5513, 6731.6)(745.5552, 2846.64)(1486.1033, 219.85)	113207.9	71889	130599.2	111708.4	169111.4	147236.7	172181.4	101547.7	0.82	0.001099	0.002836	0.09	0.743586	0.889774
COMPOUND_743_5466a12_34561	Rp	-	743.5466	12.35				(1532.1022, 1326.73)(1533.1086, 1096.1)(742.5383, 15152.77)(743.5413, 7262.89)(744.5444, 1692.66)	177276.7	19462.7	194507.8	21386.35	205209.4	30763.69	195844.5	40815.74	0.90	0.002584	0.006271	-0.88	0.011776	0.167839
COMPOUND_743_5501a12_082787	Rp	+	743.5501	12.08	PC 15:0_18:2	1	10 eV: 744.554(100), 184.0742(15)	(1488.0997, 2398.51)(1489.1044, 1995.41)(1490.1106, 1104.68)(1491.1007, 622.74)(1492.1038, 253.66)(766.5397, 8710.94)(767.542, 4825.79)(768.5548, 60209.89)(1510.13, 5300.95)(1511.1271, 4202.56)(744.556, 139956.17)(745.5589, 60553.45)(746.5612, 15468.46)(747.5625, 3315.35)(748.563, 681.85)(1505.0992, 1797.38)	1627148	382391.5	1626324	532839.8	1861054	495465.9	1627406	429487.5	0.38	0.17878	0.265575	-0.36	0.279738	0.587659
COMPOUND_743_5539a12_984675	Rp	+	743.5539	12.98	PE 36:2	2	20 eV: 603.5355(100), 184.076(32), 744.5828(32), 306.274(5)	(1488.0985, 168.95)(1489.1083, 214.4)(1490.1002, 163.84)(1526.1271, 368.36)(766.5427, 3800.33)(767.5475, 1854.46)(744.5577, 61212.6)(745.5613, 26872.08)(746.5652, 8968.9)(747.5693, 2349.6)(1505.1592, 786.62)	91882.33	6049.352	162881.6	47566.75	255333.7	91662.09	311057.2	122223.5	0.93	4.49E-07	2.47E-06	0.26	0.185547	0.499463
COMPOUND_743_584a12_730444	Rp	+	743.5840	12.73	PC 34:2e	2	20 eV: 184.074(100), 744.5898(65), 423.7637(3)	(1488.1492, 286.63)(1489.1503, 172.37)(1490.1603, 164.28)(766.5761, 8420.09)(767.5793, 4058.03)(1510.1224, 1044.49)(744.5904, 27657.08)(745.5941, 12899.96)(746.5905, 4428.05)(747.5878, 1213.3)(748.5672, 180.55)	612557.2	308401.4	552718.2	194905	582021.7	206778.5	508325.6	221818.1	-0.10	0.686187	0.77072	-0.15	0.598212	0.805251
COMPOUND_743_5866a13_077679	Rp	+	743.5866	13.08	PC 34:2e	2	20 eV: 184.0732(100), 744.5867(70)	(1488.2507, 166.9)(1489.2606, 183.6)(1490.258, 195.92)(1526.2097, 153.22)(1527.2037, 140.3)(766.5671, 4702.47)(767.5717, 2229.47)(768.5865, 5839.69)(769.589, 2663.5)(770.6047, 19008.3)(771.6136, 9886.41)(1510.1852, 132.73)(744.5871, 25231.51)(745.5905, 11634.96)(746.6051, 31169.39)(747.6089, 13658.45)(748.6082, 4102.05)(1505.162, 305.73)	505042.9	317290.8	445444.7	276307.5	421956.5	207731	330340.4	184862.7	-0.64	0.014793	0.030259	-0.08	0.791641	0.911439
COMPOUND_744_1193a11_028072	Rp	+	744.1193	11.03				(767.1271, 3381.78)(783.066, 340.17)(762.1285, 2177.58)(745.1244, 9530.09)(746.1183, 5064.03)(747.104, 2270.03)	66140.32	38425.62	71695.32	53366.3	55201.85	17626.64	67453.46	22459.88	0.08	0.788942	0.847257	0.10	0.767928	0.902789
COMPOUND_744_1705a8_785409	Rp	-	744.1705	8.79				(1533.4058, 69.79)(743.1644, 3973.59)(744.1691, 1231.44)(745.17, 358.48)(1488.3391, 78.69)(790.2031, 150.67)(791.1945, 57.76)	13618.85	1905.094	13500.74	2285.515	14041.25	1934.6	14019.15	2284.153	-0.59	0.041809	0.075022	0.24	0.475918	0.739151
COMPOUND_744_1709a8_742204	Rp	-	744.1709	8.74				(1533.3369, 134.15)(743.1639, 3047.41)(744.1659, 1656.95)(745.1681, 813.61)(1487.4183, 126.91)(1488.4235, 51.51)(789.1479, 256.05)(790.1404, 220.04)(791.1466, 322.32)	13183.92	1999.542	13255.36	2891.092	13411	2056.928	15126.26	5665.965	-0.36	0.22908	0.324051	0.67	0.063462	0.307286
COMPOUND_744_1724a9_074611	Rp	-	744.1724	9.07				(1523.252, 104.2)(1524.2648, 95.64)(857.1748, 210.24)(858.1582, 196.55)(859.1687, 205.31)(1533.2814, 72.59)(743.1641, 3034.56)(744.1672, 950.0)(745.1578, 378.84)(746.1408, 90.31)(789.1006, 487.06)(790.1162, 159.47)	28888.64	8213.995	30909.67	6409.164	27634.21	7945.805	27502.81	8257.289	-0.14	0.631504	0.721883	-0.35	0.322315	0.628989
COMPOUND_745_5551a10_064624	Rp	+	745.5551	10.06				(1492.185, 127.21)(776.5565, 614.0)(777.0363, 489.44)(777.5422, 2012.3)(778.0445, 1397.93)(382.2966, 1711.58)(382.8012, 716.84)(383.292, 804.79)(746.5632, 13012.64)(747.5641, 5474.17)(748.5464, 442.03)(373.7866, 947.78)(374.2881, 699.63)(374.7923, 160.67)	101725.9	34054.11	84905.97	19694.24	110588.3	19997.14	111566.1	30413.83	0.27	0.326643	0.42693	0.65	0.052058	0.285939
COMPOUND_745_56a12_786839	Rp	-	745.5600	12.79				(858.5233, 2117.5)(859.5297, 1042.63)(1536.1354, 885.41)(1537.1361, 739.03)(744.5532, 9549.91)(745.5565, 4555.73)(746.5586, 1169.81)	125462	21525.07	130497.2	26229.76	150153.4	26420	170927.5	28686.73	0.67	0.004362	0.009963	0.52	0.053778	0.288355
COMPOUND_745_5677a12_445017	Rp	+	745.5677	12.45	PC 33:1	2	20 eV: 184.0732(100), 746.5687(65), 482.3166(6)	(1492.1234, 328.24)(1493.1448, 276.86)(1494.1198, 143.81)(1495.0905, 41.46)(768.5787, 13139.44)(769.5786, 4009.27)(770.5804, 1103.7)(1514.1185, 1445.69)(1515.1193, 1417.06)(746.5711, 174925.75)(747.574, 76380.46)(748.5661, 20668.23)(749.5744, 2194.63)(750.5736, 354.11)(1509.1072, 1898.65)	913791.3	297202	822914.2	285355.8	980659	147039.7	1123755	329025.4	0.40	0.095948	0.157217	0.65	0.02149	0.206818
COMPOUND_745_5993a13_282169	Rp	+	745.5993	13.28	PC 34:1e	2	10 eV: 746.6066(100), 184.0723(25)	(1492.2437, 136.59)(768.5886, 5883.64)(769.5916, 2933.61)(770.6047, 24607.68)(771.6093, 11803.67)(1514.2533, 161.75)(746.6063, 53747.46)(747.6097, 24530.16)(748.6125, 6455.42)(749.6136, 1252.56)(750.6277, 215.36)(1509.243, 168.05)(1510.2402, 124.13)	529242	217320.3	474269.8	229661	454537.4	193163.7	419520.7	210703	-0.49	0.069749	0.118546	0.22	0.470784	0.734155
COMPOUND_746_5564a11_781273	Rp	-	746.5564	11.78	SM d34:2	2	20 eV: 685.5281(100), 745.5506(8), 41.0579(2)	(1528.0573, 1496.66)(1529.0609, 1203.3)(1530.0624, 952.54)(859.5299, 3678.16)(1538.0928, 362.19)(1539.0793, 375.1)(1540.0712, 284.67)(745.5493, 115490.57)(746.5524, 50785.64)(747.5553, 12709.24)(748.5544, 1899.35)(749.5557, 337.71)(1492.0392, 437.69)	2195169	478458.2	2303688	704939	2325469	484600	2278689	517604	0.21	0.43874	0.544076	-0.18	0.57079	0.789098
COMPOUND_746_9255a11_077844	Rp	+	746.9255	11.08				(769.9304, 3436.88)(764.9423, 3858.43)(747.9288, 14375.16)(748.9256, 6221.3)(749.9249, 2711.54)	83202.67	54685.42	84814.53	64904.44	70109.62	30692.88	71776.8	24630.34	0.61	0.020639	0.040592	-0.10	0.736708	0.888668
COMPOUND_747_1229a11_073729	Rp	+	747.1229	11.07				(770.1196, 3004.61)(748.1295, 18586.34)(749.1199, 6617.17)(750.117, 2253.02)	61410.1	35797.78	54456.67	41257.52	46607.56	8083.817	57453.56	17894.61	-0.37	0.152504	0.231428	0.61	0.049648	0.282107
COMPOUND_747_5191a12_323417	Rp	-	747.5191	12.32				(746.5119, 36518.02)(747.5156, 17264.94)(748.5191, 5052.0)(749.5231, 1000.33)(1494.0515, 2225.48)	73535.87	59054.04	69234.27	61182.84	107902.9	108116.7	72039.28	80401.44	0.37	0.219986	0.31355	-0.37	0.307695	0.613754
COMPOUND_747_5196a12_4341	Rp	-	747.5196	12.43	PE 38:7e	2	20 eV: 746.5135(100), 327.2332(28), 436.2797(13), 31.9118(4), 229.1934(3)	(746.512, 61227.2)(747.5157, 28899.63)(748.5208, 8569.65)(749.5251, 2125.6)(1494.0519, 321.54)(1495.068, 291.89)	202140.5	66333.03	207920.3	68396.76	290091.6	82969.33	275971.2	72098.07	1.17	2.91E-05	0.000104	-0.27	0.38403	0.686377
COMPOUND_747_5308a12_425919	Rp	+	747.5308	12.43				(1496.079, 523.78)(1497.0669, 475.43)(770.5156, 2469.84)(771.5388, 2186.61)(772.5442, 809.38)(765.5557, 2815.18)(748.5355, 21816.42)(749.5346, 10070.84)(750.5386, 3358.39)(751.5471, 909.1)(752.5247, 205.09)(1513.1061, 690.58)	163485.6	70451.36	150059.1	60736.62	231688.9	64405.52	229894.8	66492.22	0.84	0.00248	0.00605	-0.19	0.543421	0.776977

									1st trimester				3rd trimester									
									Control		Pre-eclampsia		Control		Pre-eclampsia		Time			Interaction		
Compound	Column	Ion_mode	Mass	Retention_	Identification	ID_level	MSMS_spectra	CompositeSpectrum	Mean	SD	Mean	SD	Mean	SD	Mean	SD	Value	raw p	FDR_p	Value	raw p	FDR_p
COMPOUND_747_5355a9_359567	Rp	+	747.5355	9.36				(778.538, 532.59)(779.0193, 157.43)(779.5303, 346.35)(385.7685, 665.77)(386.2697, 450.69)(386.777, 146.29)(383.2912, 3318.28)(383.7918, 1308.36)(384.3015, 603.87)(748.542, 13972.24)(749.544, 6046.18)(750.5453, 1801.9)(751.5507, 422.34)(1513.0688, 66.95)(374.7763, 2360.24)(375.2766, 1526.58)(375.7779, 456.05)	216414.6	50097.46	187509.7	39525.14	225863.9	41292.19	223611.6	58308.93	0.04	0.890622	0.926471	0.68	0.050786	0.283798
COMPOUND_748_3746a5_343451	Hilic	+	748.3746	5.34				(771.3658, 3079.24)(772.3672, 1198.82)(773.3691, 355.67)(749.3832, 10233.13)(750.387, 4404.29)(751.3899, 1119.15)(752.3963, 226.89)(375.1969, 497.43)(375.6994, 178.99)	11679.18	8041.885	14497.16	9593.049	29803.67	9949.195	27378.19	15162.73	1.02	4.83E-06	2.06E-05	0.02	0.934965	0.971639
COMPOUND_748_3749a4_594323	Rp	-	748.3749	4.59				(747.3672, 27452.4)(748.3701, 11338.0)(749.372, 2720.76)(750.3726, 522.7)(751.3639, 81.98)	#DIV/0!	#DIV/0!	#DIV/0!	#DIV/0!	#DIV/0!	#DIV/0!	#DIV/0!	#DIV/0!	1.05	8.1E-06	3.29E-05	0.00	0.997595	0.99937
COMPOUND_748_3761a4_607116	Rp	+	748.3761	4.61				(771.3713, 200.2)(394.1707, 9641.79)(394.6714, 3904.05)(395.1732, 906.34)(395.6719, 250.88)(386.1839, 1137.52)(386.6873, 493.9)(387.1761, 7108.76)(387.6777, 2630.19)(787.3251, 210.43)(749.3847, 54798.9)(750.3879, 22236.04)(751.3881, 5674.61)(752.3922, 1113.77)(753.3955, 263.89)(413.145, 659.46)(413.6434, 200.37)(414.1455, 73.23)(375.1978, 2578.32)(375.698, 999.94)(376.2017, 251.65)(376.6935, 67.37)	73041.81	71753.98	77540.4	76337.25	259096.2	114158.9	245435.2	145509.1	0.93	0.000252	0.000733	0.19	0.505101	0.754687
COMPOUND_748_5726a12_090697	Rp	-	748.5726	12.09	SM d34:1	2	20 eV: 687.543(100), 747.566(14), 399.842(2), 152.1508(1)	(1532.0952, 3942.56)(1533.0941, 3217.87)(747.565, 405617.1)(748.5685, 173234.52)(749.5712, 42389.85)(750.5702, 5811.3)(751.5728, 873.63)	2670060	711880.9	2702743	940262.8	2605528	491948.4	2477125	615124.5	-0.01	0.969727	0.982819	-0.19	0.585736	0.798957
COMPOUND_749_5206a11_796187	Rp	-	749.5206	11.80				(1534.0793, 351.86)(1535.0698, 358.47)(862.4909, 1784.1)(863.4995, 1233.85)(784.4908, 850.34)(785.5052, 1559.78)(748.5126, 66627.53)(749.5158, 27455.67)(750.5188, 7272.59)(751.5212, 1591.03)(752.5272, 266.61)(794.5291, 1441.62)(795.539, 1236.24)(796.5186, 307.59)	183342.6	78362.33	201367.6	102962	336342.3	113564.8	444975.2	239209.3	1.04	7.34E-06	3.01E-05	0.23	0.346413	0.651869
COMPOUND_749_5347a12_651332	Rp	-	749.5347	12.65				(862.4908, 580.4)(863.4748, 221.81)(864.5114, 1127.38)(748.5279, 49183.29)(749.5311, 22916.92)(750.5346, 6159.56)(751.5379, 1314.72)(1498.0781, 387.75)(1499.081, 310.02)(1500.0914, 303.11)(794.5331, 4259.19)(795.5386, 5104.72)(796.5429, 2746.0)	265913.2	104790.3	266148.8	119626.6	270837.7	100226.3	234104	84506.85	0.09	0.721827	0.79645	-0.33	0.275106	0.58392
COMPOUND_749_5389a12_641697	Rp	+	749.5389	12.64	PE 38:6e	2	20 eV: 390.2767(100), 609.5197(28), 137.1271(23), 484.2974(15)	(1500.1339, 1058.19)(1501.1393, 924.98)(1538.0012, 168.71)(1538.9836, 143.65)(788.4941, 479.5)(789.4933, 332.97)(750.5441, 62598.79)(751.5472, 29252.09)(752.551, 8070.21)(753.5654, 2080.08)	293269.9	122272.3	264641.1	99798.61	295396.3	99934.46	258617.5	76434.41	0.17	0.49893	0.599888	-0.13	0.651883	0.83936
COMPOUND_750_122a10_973539	Rp	+	750.1220	10.97				(773.1258, 2408.1)(774.1408, 6875.95)(789.0541, 731.9)(751.127, 11072.71)(752.1212, 4838.64)(753.1103, 2299.99)	66787.16	37619.08	63636	43998.49	68523.11	41227.77	78475.73	46210.14	-0.20	0.453016	0.558703	0.43	0.180672	0.493854
COMPOUND_751_5153a11_583241	Rp	+	751.5153	11.58	PC 34:5	2	20 eV: 184.0728(100), 752.5246(80), 86.0977(5), 287.2077(4), 441.5945(3)	(1503.997, 202.26)(1541.9987, 342.93)(774.4916, 5284.28)(1525.9878, 536.36)(769.5529, 2112.5)(770.5496, 2490.71)(771.5479, 1640.92)(752.5245, 49165.13)(753.5276, 23159.97)(754.5304, 6801.24)(755.5323, 1973.0)	123644.7	78059.67	143203.8	91782.07	105315.8	99160.33	98917.11	70620.55	0.09	0.731639	0.805246	0.06	0.831128	0.926681
COMPOUND_751_5358a12_125399	Rp	-	751.5358	12.13				(1538.0728, 211.86)(1539.086, 415.0)(864.5318, 1419.42)(865.5359, 724.08)(866.5483, 1803.93)(786.508, 428.21)(1548.0729, 1537.21)(1549.0758, 997.44)(750.5287, 55184.12)(751.5316, 24023.35)(752.5338, 6297.49)(753.5355, 1489.79)(754.5166, 341.5)(1502.1023, 477.88)	243826.4	112198.5	263473.3	117438	298913.7	66590.88	345746.9	113750.2	0.60	0.014952	0.030457	0.08	0.788772	0.909997
COMPOUND_751_5516a13_248221	Rp	-	751.5516	13.25				(1538.1427, 600.9)(1539.1523, 311.28)(786.5659, 1280.82)(787.5577, 976.41)(864.5346, 706.41)(865.5298, 390.92)(866.5401, 511.15)(750.543, 27710.7)(751.5463, 13391.32)(752.5498, 4106.92)(753.5509, 1018.16)(796.5677, 2786.65)(797.5653, 2391.7)(798.5605, 1201.52)	#DIV/0!	#DIV/0!	#DIV/0!	#DIV/0!	#DIV/0!	#DIV/0!	#DIV/0!	#DIV/0!	-0.50	0.050743	0.088721	-0.01	0.965914	0.982565
COMPOUND_751_5526a13_230772	Rp	+	751.5526	13.23	PC 35:5e	2	40 eV: 69.0696(100), 361.2729(52), 752.713(12), 572.8602(11)	(774.5428, 1352.23)(775.5487, 594.58)(776.5608, 1219.9)(1526.1188, 105.86)(790.5118, 764.2)(791.4993, 335.34)(792.5079, 37.21)(769.5931, 3487.93)(752.5592, 24766.83)(753.5624, 12265.78)(754.5648, 3654.81)(755.5691, 1037.03)	134471.6	67292	124103.1	75490.61	121506.8	59952.68	113185	47322.93	-0.15	0.537674	0.636056	0.13	0.650438	0.838541
COMPOUND_753_1226a11_019529	Rp	+	753.1226	11.02				(776.1276, 2957.81)(771.1385, 2791.37)(754.1344, 15818.87)(755.124, 6746.13)(756.1151, 2026.51)	65403.02	41790.67	69936.53	45152.08	62510.91	15412.82	59177.45	18310.98	0.03	0.907078	0.938328	0.08	0.810111	0.919078
COMPOUND_753_5327a11_815522	Rp	+	753.5327	11.82	PC 34:4	2	10 eV: 754.5403(100), 184.0738(15), 55.0517(2)	(1508.0669, 1276.07)(1509.0692, 1450.42)(1510.0801, 2167.87)(1511.0869, 1556.54)(1546.0602, 559.14)(776.5228, 17818.56)(777.5274, 8618.16)(778.5412, 75459.88)(779.5435, 35811.36)(780.5569, 179420.17)(781.5597, 82722.81)(1530.0591, 578.83)(1531.0743, 716.03)(1532.0627, 1026.09)(792.4579, 2965.9)(793.4589, 2600.44)(754.5409, 251810.92)(755.5437, 113333.91)(756.5468, 30073.1)(757.5443, 7231.39)	1138011	466549.7	1182417	477681.8	1757745	768476	1657625	575826.6	1.03	0.000176	0.000526	-0.25	0.418873	0.69956
COMPOUND_753_592a13_915185	Rp	+	753.5920	13.92				(754.5997, 8794.68)(755.6031, 4818.47)(756.6001, 1940.1)(757.6233, 574.17)	58613.33	27656.34	51885.52	20051.99	40536.67	1144.432	33237	11801.74	-1.27	4.28E-09	3.86E-08	0.05	0.804179	0.917422
COMPOUND_753_9893a10_244545	Rp	+	753.9893	10.24				(1508.9885, 40680.93)(1509.9911, 33622.91)(1510.9928, 16207.77)(1511.991, 6353.98)(1546.9331, 1228.4)(1547.9062, 1752.04)(1548.9067, 2035.94)(1549.9115, 1378.45)(1550.9156, 1312.93)(1551.9196, 1107.44)(1552.9283, 959.46)(776.948, 3417.53)(777.9391, 1636.8)(1530.9526, 1291.75)(754.9949, 7248.84)(756.0154, 6422.04)(757.0157, 1677.98)(758.0041, 309.16)	285490.8	53767.5	274715.4	43895.18	289039.6	16050.13	298507.6	36728.74	0.11	0.695974	0.77822	0.51	0.144604	0.451201
COMPOUND_755_5479a11_088077	Rp	+	755.5479	11.09				(778.5399, 120993.68)(779.5424, 59183.2)(780.553, 69754.84)(1534.0808, 736.36)(397.7605, 1027.79)(398.2621, 603.07)(398.7686, 699.16)(389.7691, 1251.98)(390.2734, 790.9)(390.7726, 647.03)(756.5571, 477299.34)(757.5611, 212724.83)(758.5618, 58134.77)(759.5594, 13941.22)(416.7359, 5101.96)(417.2388, 3213.81)(378.7822, 1213.19)(379.2977, 984.25)(379.7844, 83.0)	1649371	809010.8	1694209	704801.4	1488923	886681.8	1388438	632521.6	-1.06	2.32E-06	1.07E-05	-0.26	0.292851	0.602481
COMPOUND_755_5492a11_9932575	Rp	+	755.5492	11.99	PC(16:1_18:2) duplicate			(1512.1018, 11629.54)(1513.1046, 10361.32)(1514.1063, 5251.61)(1515.107, 2212.84)(1516.1082, 985.62)(1550.1006, 5532.11)(1551.101, 3987.04)(1552.0929, 2556.76)(778.5371, 25451.26)(779.5407, 12675.13)(780.5555, 672716.56)(1534.0872, 4045.04)(1535.092, 4111.21)(1536.1003, 17769.83)(1537.1038, 16130.84)(1538.1102, 15801.29)(1539.1151, 10615.37)(1540.115, 5228.02)(794.5504, 8645.19)(773.6152, 594.35)(756.5564, 751765.2)(757.5604, 339736.84)(758.5634, 86802.87)(759.5662, 17307.7)(760.5757, 4126.16)(1529.1024, 686.79)(1530.0876, 702.5)	15362032	5866578	15229628	5890754	22635714	5719146	23621053	6255590	0.97	0.000132	0.0004	0.17	0.550093	0.7796
COMPOUND_755_5503a11_170548	Rp	+	755.5503	11.17	PC 34:3	2	20 eV: 756.5538(100), 184.0737(85)	(778.5395, 7390.31)(779.5476, 5291.96)(780.5563, 74930.2)(1534.0996, 785.05)(1535.0989, 502.03)(1536.1237, 190.67)(397.7602, 1931.52)(398.2584, 1341.15)(398.7718, 1131.21)(389.768, 3293.15)(390.2695, 2142.99)(390.772, 1237.13)(756.5549, 719676.9)(757.559, 318297.44)(758.5618, 87530.7)(759.5627, 18177.21)(378.7845, 5670.65)(379.2858, 2633.37)(379.7884, 814.95)	4239682	966992.4	4268790	1024213	3341303	2593144	3647098	1845634	-0.99	1.5E-05	5.78E-05	-0.26	0.304305	0.613089
COMPOUND_755_5507a11_965143	Rp	+	755.5507	11.97	PC 16:1_18:2	1	10 eV: 756.5551(100), 184.0743(10)	(1512.1007, 9956.59)(1513.103, 9262.87)(1514.1053, 4817.16)(1515.1096, 2042.05)(1516.1064, 951.94)(1550.0813, 1839.54)(1551.0797, 1489.68)(1552.0886, 825.27)(778.5369, 23317.08)(779.5398, 11981.6)(780.5552, 519573.22)(1534.0905, 2603.2)(1535.093, 2610.39)(1536.1008, 8751.83)(1537.1025, 8312.71)(1538.1123, 9669.33)(1539.1149, 7106.73)(1540.1167, 3608.0)(773.5504, 1570.53)(794.5499, 6824.95)(756.5559, 777350.8)(757.5605, 346212.47)(758.5631, 90033.48)(759.5659, 17168.6)(760.5753, 4058.2)(1529.112, 1329.28)(1530.0981, 1078.51)	6030459	5990580	5624574	5035051	6039651	5557031	5950080	5840752	0.47	0.093441	0.153711	-0.13	0.698132	0.862515
COMPOUND_756_1442a9_242933	Rp	-	756.1442	9.24				(869.0893, 126.25)(870.1058, 285.89)(871.0889, 278.08)(791.134, 412.34)(792.1455, 151.7)(793.1427, 453.72)(755.1371, 12010.11)(756.1407, 3242.73)(757.1361, 3894.75)(758.1373, 1163.0)(759.1387, 517.46)(760.1274, 249.05)(801.136, 808.24)(802.1526, 378.15)	#DIV/0!	#DIV/0!	#DIV/0!	#DIV/0!	#DIV/0!	#DIV/0!	#DIV/0!	#DIV/0!	-0.57	0.054734	0.095108	0.00	0.999832	0.999832

									1st trimester				3rd trimester									
									Control		Pre-eclampsia		Control		Pre-eclampsia		Time			Interaction		
Compound	Column	Ion_mode	Mass	Retention_	Identification	ID_level	MSMS_spectra	CompositeSpectrum	Mean	SD	Mean	SD	Mean	SD	Mean	SD	Value	raw p	FDR_p	Value	raw p	FDR_p
COMPOUND_757_5605a11_226129	Rp	+	757.5605	11.23				(1554.085, 1020.77)(1555.0707, 700.91)(1556.1051, 1499.25)(1557.1051, 1348.45)(1558.1272, 1928.55)(1559.1296, 1456.76)(1560.1248, 2311.57)(780.5563, 173445.95)(781.5593, 81532.26)(1538.0461, 702.54)(1539.0385, 581.03)(758.5698, 45608.08)(759.5726, 20405.94)(760.584, 22026.92)(761.5872, 8770.57)	1726247	780725.8	1504299	729598.7	1800574	1343115	1839616	986480.5	-0.95	6.35E-05	0.000206	-0.06	0.808527	0.918373
COMPOUND_757_5641a12_321749	Rp	+	757.5641	12.32	PC 16:0_18:2	1	10 eV: 758.57(100), 184.0731(9), 86.1007(1)	(1516.1355, 23609.54)(1517.1406, 21656.95)(1518.1401, 10809.06)(1519.145, 4115.02)(1520.1562, 1292.38)(1521.1552, 422.02)(780.5519, 29431.81)(781.5562, 13746.2)(782.5707, 788420.3)(1538.12, 21362.11)(1539.1232, 20521.71)(1540.1289, 15663.63)(796.5274, 1680.55)(797.5285, 1154.91)(798.5416, 7864.87)(799.5422, 4385.55)(758.5705, 1394080.1)(759.5749, 610906.7)(760.5778, 157150.3)(761.5814, 28391.69)(762.5827, 5027.26)(763.5895, 931.33)(1533.119, 5405.26)(1534.1272, 2762.23)	87701887	11589460	85180435	13312127	97121429	8969451	98256140	13504008	0.76	0.004969	0.011213	0.28	0.360358	0.666906
COMPOUND_759_5408a11_4646635	Rp	-	759.5408	11.46				(1554.0796, 79.59)(1555.0878, 204.78)(1556.0865, 224.11)(1557.0836, 377.31)(1558.0908, 168.46)(1564.0941, 299.18)(1565.0929, 360.17)(1566.0972, 323.64)(1567.1079, 348.45)(1568.1151, 278.83)(1569.1127, 194.6)(1570.1094, 132.82)(758.533, 10252.37)(759.5363, 5282.82)(760.5401, 1361.4)(761.5398, 424.78)(1518.07, 68.76)(1519.101, 363.43)(1520.1038, 285.92)	38866.7	18958.71	48564.29	22477.96	62440	45490.73	66649.67	35983.25	-0.67	0.003876	0.008979	-0.55	0.044404	0.272788
COMPOUND_759_5788a12_747065	Rp	+	759.5788	12.75	PC 34:1	2	10eV: 760.5839(100), 184.0745(10)	(1520.1632, 14861.03)(1521.1665, 13148.03)(1522.1694, 6828.42)(1523.175, 2316.85)(1524.1807, 854.19)(1525.2186, 189.13)(782.5684, 74705.47)(783.5711, 35495.89)(784.5846, 475963.12)(1542.148, 3839.1)(1543.1492, 3275.27)(1544.1564, 9582.53)(1545.1616, 7822.73)(1546.1711, 10916.35)(798.5423, 1756.6)(799.5453, 923.93)(800.5515, 2281.98)(801.5694, 1321.2)(760.5857, 1176076.0)(761.5899, 511765.62)(762.5928, 124819.09)(763.5945, 24120.88)(764.5944, 4004.87)(765.5799, 639.61)	38454717	7608335	36895652	9142671	45464286	6632044	51857895	10625491	0.71	0.002107	0.005219	0.65	0.014285	0.179521
COMPOUND_760_5734a11_967111	Rp	-	760.5734	11.97				(795.5416, 1355.38)(796.5419, 1039.74)(759.5645, 6422.29)(760.5675, 3071.39)(761.5742, 1136.96)(762.5916, 311.18)(805.5636, 1067.01)(806.5371, 1207.76)(807.5271, 709.5)	27228.44	16369.72	37228.45	20768.11	32529.36	10292.69	32089.34	10363.33	0.77	0.001386	0.00352	-0.21	0.454308	0.725699
COMPOUND_761_4078a6_7300625	Rp	+	761.4078	6.73				(400.6931, 144.73)(401.183, 66.92)(392.7006, 955.76)(393.2144, 534.97)(393.715, 112.31)(779.4091, 68.44)(390.2242, 8895.4)(390.7264, 3443.35)(391.2183, 2401.21)(391.7276, 320.38)(762.4122, 5349.72)(763.4154, 2079.69)(764.421, 661.0)(765.4122, 127.6)(381.7124, 1410.24)(382.2133, 632.03)(382.7173, 98.07)(383.2062, 26.23)	#DIV/0!	#DIV/0!	#DIV/0!	#DIV/0!	#DIV/0!	#DIV/0!	#DIV/0!	#DIV/0!	0.41	0.165265	0.248449	-0.01	0.975514	0.986465
COMPOUND_761_5008a11_849198	Rp	-	761.5008	11.85				(1558.0182, 262.32)(1559.0293, 244.94)(874.4771, 1682.31)(796.443, 389.35)(797.4571, 155.85)(1568.0522, 497.85)(1569.0548, 451.17)(760.4915, 13682.44)(761.4949, 6291.07)(762.5096, 3211.43)(763.5082, 563.37)(1522.096, 100.02)(1523.0746, 723.06)(806.4949, 1266.81)(807.5045, 1389.48)(808.5188, 1130.09)	63478.44	27214.31	69761.07	20659.64	111669.7	29776.43	142320.5	49967.61	1.16	3.18E-08	2.3E-07	-0.01	0.957303	0.980661
COMPOUND_761_5513a9_599785	Rp	+	761.5513	9.60				(784.5373, 390.55)(392.7743, 1026.27)(393.2777, 787.85)(393.7767, 209.29)(390.2969, 5906.64)(390.7998, 2107.28)(391.2978, 866.76)(391.8016, 255.8)(409.2787, 314.0)(409.7893, 118.91)(410.2718, 912.15)(762.5581, 18924.81)(763.5608, 8090.96)(764.5602, 2568.21)(765.5624, 565.99)(766.5735, 128.68)(381.7846, 4540.54)(382.2866, 2074.97)(382.7873, 675.68)	364424.7	109352.8	324462.3	69106.35	397472.5	71999.89	389186.1	105846.4	0.21	0.474974	0.578498	0.48	0.167907	0.480454
COMPOUND_761_9283a11_00543	Rp	+	761.9283	11.01				(784.9422, 2252.58)(800.8853, 1180.26)(779.9452, 4009.54)(762.9367, 12221.21)(763.9313, 4924.8)(764.9314, 2629.07)	96880	72330.06	77499.18	53451.34	61007.73	19964.98	60719.38	18407.52	0.59	0.01756	0.035151	-0.19	0.505065	0.754687
COMPOUND_763_3762a13_696421	Rp	-	763.3762	13.70				(762.369, 6179.04)(763.3725, 2578.95)(764.3669, 4223.65)(765.3694, 1865.29)(766.3663, 1020.53)(767.3693, 366.02)(768.3565, 123.7)	55762.08	16665.53	57599.07	15269.92	55314.57	20651.74	83392.07	36298.58	-0.09	0.756152	0.82302	0.92	0.006312	0.12469
COMPOUND_763_5143a12_201316	Rp	-	763.5143	12.20	PE 38:6	2	20 eV: 762.5084(100), 327.2325(38), 59.0131(1)	(876.4975, 365.31)(877.5228, 2079.46)(798.4618, 199.86)(799.4671, 86.63)(762.5077, 283883.9)(763.5109, 129447.1)(764.5142, 33674.28)(765.5182, 6497.2)(1526.0334, 2138.83)(1527.0408, 1976.23)(1528.0468, 1092.06)(1529.0472, 480.43)(1530.0452, 131.97)	474784.7	152684.1	563063.2	222244.5	1390954	414221.7	1685021	472989.1	1.35	7.17E-11	1.16E-09	0.18	0.360292	0.666906
COMPOUND_763_5148a12_525213	Rp	-	763.5148	12.53				(798.5131, 728.56)(799.5292, 59.97)(876.5016, 1393.31)(762.5072, 7471.32)(763.5103, 3889.08)(764.5231, 235256.92)(1525.98, 166.71)(1527.0115, 137.2)(1528.0093, 157.59)(808.5107, 4007.74)(809.5145, 1934.35)	53664.86	20687.8	72061.22	41901.63	177243.9	90751.33	229779.2	143258.8	1.42	5.5E-11	9.83E-10	-0.07	0.746103	0.891365
COMPOUND_763_5204a12_203148	Rp	+	763.5204	12.20	PE 16:0_22:6	1	10 eV: 764.527(100), 623.5025(40), 436.2825(4), 121.107(3)	(1528.0554, 3210.25)(1529.0583, 3107.53)(1530.06, 1670.16)(1531.0669, 773.4)(1532.0735, 366.39)(786.5268, 7034.45)(787.5273, 3432.91)(788.5361, 4181.89)(1550.0162, 413.41)(802.5196, 4142.5)(803.5117, 1956.42)(764.525, 245985.36)(765.528, 111609.7)(766.5314, 28878.7)(767.5344, 6403.61)(1545.102, 2689.04)(1546.0885, 4762.29)(1547.0912, 4444.65)(1548.0985, 4814.09)	465637	133760.7	525662.2	167614.4	1211360	271510.9	1403337	371886.7	1.31	1.35E-11	3.16E-10	0.25	0.182711	0.496338
COMPOUND_764_1579a9_575788	Rp	-	764.1579	9.58				(763.1505, 4792.96)(764.153, 1299.33)(765.1662, 43731.81)(766.1696, 10815.7)	150600.8	42699.38	163183.5	57951.3	170544.6	90585.62	166366.1	68437.44	0.09	0.768387	0.83299	-0.09	0.796333	0.91372
COMPOUND_765_5298a12_529418	Rp	-	765.5298	12.53				(764.5231, 235256.92)(765.5261, 112155.85)(766.5291, 29087.6)(767.5317, 5516.38)	84420.17	55776.98	99917.16	61106.33	240761.7	129508.2	322034	210913.8	1.24	7.29E-08	4.77E-07	0.13	0.580445	0.794145
COMPOUND_765_53a12_392425	Rp	-	765.5300	12.39				(764.5229, 63231.61)(765.5264, 29805.16)(766.5298, 8937.01)(767.533, 2212.91)	96205.98	31568.35	104171.8	39826.22	239051	69456.25	288619.5	92811.74	1.29	4.72E-10	5.74E-09	0.27	0.194035	0.505089
COMPOUND_765_5675a12_500338	Rp	+	765.5675	12.50	PC 36:5e	2	10 eV: 766.5776(100), 184.0733(12), 483.954(1)	(1532.1361, 1146.4)(1533.1292, 976.41)(1534.13, 603.29)(1535.1241, 227.8)(1556.152, 3612.85)(1557.1545, 3592.85)(1558.144, 3761.08)(1559.1405, 2975.76)(1560.1346, 2219.43)(766.5757, 263093.16)(767.5789, 125330.27)(768.5823, 37302.7)(769.5864, 9370.35)	1905792	513369.7	1775173	575094.8	1695816	397808.9	1768704	404567	-0.34	0.206498	0.297335	0.27	0.380999	0.683863
COMPOUND_766_1729a9_243429	Rp	-	766.1729	9.24				(879.1367, 469.36)(880.1151, 274.91)(881.1217, 298.49)(765.1662, 24435.96)(766.1695, 7050.85)(767.1706, 1263.75)(768.1641, 272.03)(769.1889, 61.34)(811.1543, 450.38)(812.1515, 297.59)	172189.7	24686.2	174621.2	22529.02	174682.8	17050.11	169583.7	27078.44	0.16	0.597739	0.692087	-0.42	0.235957	0.541667
COMPOUND_766_1731a9_529215	Rp	-	766.1731	9.53				(801.1388, 802.22)(802.149, 474.9)(803.1217, 414.07)(765.1662, 52526.74)(766.1698, 13612.13)(767.1715, 1992.2)(768.1645, 410.83)	194829.7	35031.19	210330.2	46164.8	193662.2	36783.83	192976.4	42918.68	-0.07	0.8045	0.859456	-0.43	0.235673	0.541667
COMPOUND_766_1731a9_439197	Rp	-	766.1731	9.44				(879.1465, 882.31)(880.1384, 398.81)(765.1662, 45710.95)(766.1693, 11881.3)(767.1677, 1906.32)(768.1714, 258.29)	178560.2	21935.99	189168.9	54093.37	178338.6	23260.47	178262.1	18348.26	-0.03	0.920216	0.94911	-0.23	0.509748	0.757224
COMPOUND_766_5332a12_5289	Rp	-	766.5332	12.53				(765.5261, 112155.85)(766.5291, 29087.6)(767.5317, 5516.38)	30192.93	19039	35095.08	19838.21	77130.5	40975.38	102732.6	67632.2	1.20	7.03E-08	4.62E-07	0.20	0.387945	0.68827
COMPOUND_766_5731a14_229163	Rp	-	766.5731	14.23				(765.5664, 18668.98)(766.5697, 9603.07)(767.5743, 3273.62)(768.5784, 764.74)	77023.26	48478.6	84562.74	43856.24	29392.67	16345.35	49092.11	20813.43	-1.34	3E-10	4E-09	-0.13	0.547789	0.77853
COMPOUND_766_5733a14_3356285	Rp	-	766.5733	14.34				(765.5663, 17362.2)(766.5695, 8989.28)(767.5742, 2735.05)(768.579, 760.21)	60441.13	34409.06	66140.11	33660.2	24350.67	16156.56	29093.25	16018.08	-1.34	6.69E-11	1.1E-09	-0.09	0.655786	0.841269
COMPOUND_767_5333a1_2194676	Hilic	+	767.5333	1.22				(768.5375, 1912.27)(769.5396, 941.28)(770.5422, 361.17)(384.7732, 15072.16)(385.2748, 6838.59)(385.7762, 2000.07)(386.2784, 454.56)	36261.15	22953.63	40138.7	29948.04	52585.5	54145.44	47398.42	32629.36	0.43	0.142563	0.218998	-0.15	0.664164	0.844813
COMPOUND_767_5451a12_906821	Rp	-	767.5451	12.91				(766.5385, 97023.32)(767.5416, 45794.66)(768.5448, 12794.16)(769.5484, 2499.08)(1534.1033, 515.11)(1535.1082, 459.12)(1536.1237, 651.64)(1537.1321, 505.34)(1538.1373, 356.34)	166175.5	59345.54	182422.5	65180.84	324437.2	103607.7	377455.3	153777.1	1.28	1.48E-08	1.17E-07	0.07	0.770173	0.902789
COMPOUND_767_5484a12_00333	Rp	+	767.5484	12.00				(1574.0884, 3418.56)(1575.0732, 3652.94)(1576.07, 4004.27)(790.5433, 3913.54)(791.5477, 3596.26)(792.5567, 50325.04)(793.5584, 25088.95)(1558.0807, 4222.15)(1559.085, 5479.21)(1560.103, 119648.78)(768.5548, 75818.35)(769.557, 36114.42)(770.5585, 10325.16)(771.5515, 3013.42)(1553.0887, 1737.25)	863715.3	415125.9	889683.9	432002.2	1059483	585464.5	1024227	480567.3	0.41	0.166516	0.249829	-0.20	0.570561	0.789098
COMPOUND_767_5546a12_897624	Rp	+	767.5546	12.90	PE 38:4	2	10 eV: 768.5629(100), 627.5364(30), 482.3127(2)	(1536.1191, 664.19)(1537.1206, 548.7)(1538.1375, 549.57)(1539.1469, 395.19)(1540.155, 572.18)(1574.0593, 146.18)(1575.0972, 51.26)(790.5494, 6269.41)(791.5475, 3775.38)(1558.008, 164.05)(1559.0292, 105.41)(768.5591, 90742.13)(769.5621, 42187.6)(1553.1426, 898.69)	206797.5	8647.209	296067	75649.85	382165.5	117290	425803.2	136457.2	1.17	3.06E-11	6.26E-10	0.10	0.541242	0.776977

Compound	Column	Ion_mode	Mass	Retention_	Identification	ID_level	MSMS_spectra	CompositeSpectrum	1st trimester				3rd trimester				Time			Interaction		
									Control		Pre-eclampsia		Control		Pre-eclampsia							
									Mean	SD	Mean	SD	Mean	SD	Mean	SD	Value	raw p	FDR_p	Value	raw p	FDR_p
COMPOUND_767_585a12_670887	Rp	+	767.5850	12.67	PC 36:4e	2	20 eV: 184.0728 (100),768.5919(85), 627.5306(2)	(1536.1624, 601.84)(1537.1556, 683.87)(1538.1431, 890.53)(1539.1418, 1474.83)(1540.1428, 2932.5)(790.571, 14631.37)(791.5696, 8582.43)(792.591, 132273.19)(793.594, 65104.33)(1558.1547, 4102.9)(1559.1475, 3438.33)(768.5909, 398045.16)(769.595, 187165.6)(770.5969, 47953.1)(771.5964, 10127.03)(1553.1562, 5369.44)	4174561	1337156	3976700	1420788	3362093	553668.2	3515920	993992.3	-0.58	0.038311	0.06969	0.25	0.439463	0.715597
COMPOUND_769_4998a12_186076	Rp	+	769.4998	12.19				(1539.9509, 202.11)(808.4636, 682.59)(787.5188, 3679.64)(385.7567, 7525.67)(386.2584, 3368.23)(386.7583, 979.65)(387.2592, 241.5)	40520.08	9386.627	44801.39	14558.47	55483	16653.86	66590.46	19050.44	0.86	0.002014	0.005007	-0.09	0.776547	0.905535
COMPOUND_769_5273a10_669453	Rp	+	769.5273	10.67				(1577.9742, 533.28)(792.5506, 7420.99)(793.5384, 2948.31)(1561.987, 608.64)(770.5312, 22246.0)(771.5348, 9783.15)(772.5441, 11809.5)(773.5475, 5012.68)	112507.8	51417.6	136908.3	77114.57	210488.7	80074.58	183679.6	97264.92	-0.49	0.036293	0.066394	-0.36	0.190638	0.505078
COMPOUND_769_5606a13_25374	Rp	-	769.5606	13.25				(768.5534, 8229.96)(769.5576, 3971.72)(770.5648, 1912.47)(771.567, 624.49)	20332.47	6654.587	25182.29	10897.69	35354.07	19095.53	54364.49	28276.01	0.94	1.27E-05	4.98E-05	0.37	0.118723	0.408824
COMPOUND_769_5619a11_26873	Rp	+	769.5619	11.27				(1578.0804, 1157.81)(1579.0875, 1023.22)(1580.102, 1705.41)(1581.0979, 1346.57)(1582.1138, 1557.87)(1562.1088, 1170.42)(1563.1069, 776.47)(1564.1102, 1082.74)(1565.1014, 867.61)(1566.0854, 961.4)(770.5697, 23710.55)(771.5728, 11078.07)(772.5515, 729494.94)(773.5555, 321151.1)(1557.1117, 1457.89)	917623.7	581993.1	1016352	690802.8	604335.7	131370.9	364753.8	140564.4	-1.28	3.74E-10	4.7E-09	-0.23	0.255712	0.56678
COMPOUND_769_5624a12_270624	Rp	+	769.5624	12.27				(1540.1357, 163646.78)(792.552, 3754.48)(793.5527, 2631.45)(794.57, 30250.69)(1562.1108, 8769.39)(1563.1154, 9628.97)(770.5697, 17929.22)(771.5724, 8581.58)(772.5749, 2416.86)(773.5719, 615.33)(774.5865, 129.94)(1557.1014, 3636.72)(1558.0974, 2014.65)	1718632	402987.6	1661633	585422.3	1983798	533157.7	1803610	797459.9	0.47	0.108981	0.17543	-0.29	0.405228	0.6912
COMPOUND_769_5628a12_2186165	Rp	+	769.5628	12.22	PC 35:3	2	10 eV: 770.5701(100), 184.0741(15), 87.0999(4)	(1540.1274, 87672.99)(792.5577, 3968.85)(793.558, 3159.17)(794.5701, 38088.42)(1562.1104, 8885.06)(1563.1155, 8363.9)(770.5704, 25886.42)(771.5735, 12312.38)(772.5756, 3602.35)(773.5753, 887.33)(774.5932, 120.63)(1557.1077, 1589.83)(1558.1075, 1560.36)	1078535	735736.4	942059	621964.4	953960.1	693705	1286197	865082.9	-0.19	0.528656	0.627588	0.66	0.065157	0.308265
COMPOUND_769_5957a12_867364	Rp	+	769.5957	12.87	PC 36:3e	2	10 eV: 770.6031(100), 184.0733(17), 629.5372(2)	(1540.1404, 1163.92)(1541.1416, 676.33)(1542.1461, 4713.99)(1543.1478, 4635.02)(1544.156, 4622.14)(1545.1622, 3112.56)(1546.175, 7773.28)(1578.1584, 4157.28)(1579.1606, 3818.54)(1580.1843, 4890.29)(1581.188, 4521.82)(1582.1823, 3520.09)(1583.1748, 2514.14)(1584.1697, 2234.85)(792.569, 25355.21)(793.5723, 12791.46)(1562.1613, 772.39)(1563.1469, 809.58)(1564.1423, 1092.88)(770.6057, 89115.32)(771.6089, 40688.12)(772.6062, 13370.63)(773.6025, 3593.03)(1557.1827, 2373.29)(1558.1766, 2312.03)	722362.1	392730.8	805160.6	348208.7	861300.4	351026.7	680053.4	304950.2	0.39	0.139726	0.215522	-0.66	0.034004	0.248347
COMPOUND_771_067a12_736616	Rp	+	771.0670	12.74				(1543.1481, 19181.99)(1544.1582, 24775.61)(1545.1638, 17655.39)(1546.1658, 10568.58)(772.0729, 4966.39)(773.0757, 1855.47)(774.0694, 425.63)	246348.2	51634.92	259007.7	63854.45	305485	44766.44	336972.4	61313.18	0.89	0.000203	0.000605	0.19	0.468621	0.734042
COMPOUND_771_5434a11_08904	Rp	+	771.5434	11.09				(1544.0742, 1228.48)(794.5344, 56573.5)(795.5392, 27781.17)(1566.0355, 397.98)(1567.0405, 405.23)(1568.0735, 1301.76)(1569.0784, 980.7)(1570.0997, 3478.95)(772.5511, 250613.19)(773.5543, 113628.8)(774.5656, 1543081.5)(775.5692, 683105.7)	1445993	615465.2	1378985	653874	2358849	1043287	2162316	1059977	-0.76	0.001868	0.004658	-0.17	0.538494	0.776408
COMPOUND_771_5455a11_323892	Rp	+	771.5455	11.32				(1544.0875, 619.84)(1545.095, 575.52)(1546.0914, 648.18)(1547.0837, 347.66)(1548.1041, 438.46)(794.533, 72513.18)(795.536, 32608.31)(796.5488, 47206.73)(797.5529, 22401.54)(1566.0721, 862.96)(1567.0747, 770.98)(1568.08, 790.04)(1569.0796, 705.88)(1570.1011, 2940.48)(789.5844, 20926.69)(772.552, 702034.9)(773.5558, 309704.94)(774.562, 119315.42)(775.5663, 33402.1)(1561.118, 819.55)(1562.1085, 1254.74)(386.7793, 626.77)(387.2823, 365.61)(387.7773, 60.22)	1900699	1141519	2073596	1316493	1033107	641597.9	724607.5	347457.8	-1.31	8.71E-11	1.34E-09	-0.21	0.296155	0.60631
COMPOUND_771_5747a13_0182085	Rp	-	771.5747	13.02				(1578.1436, 501.74)(1579.1489, 440.46)(1580.1378, 802.75)(1581.141, 611.99)(1582.139, 487.63)(1588.1515, 2883.73)(1589.1548, 2630.78)(1590.1661, 10256.14)(770.5691, 4844.49)(771.5705, 2552.46)(772.5718, 604.56)(773.5791, 171.8)(816.5799, 3579.07)	219148.6	89448.67	230208.8	79238.2	235567.3	85551.73	308053.1	96658.35	0.13	0.646483	0.735665	0.58	0.083955	0.346484
COMPOUND_771_5784a12_63684	Rp	+	771.5784	12.64	PC 17:0_18:2	1	10 eV: 772.5858(100), 184.0748(13), 293,2607(4)	(1544.1555, 2270.18)(1545.1617, 1612.39)(1546.1638, 991.93)(1547.1473, 497.02)(794.604, 32434.24)(795.6049, 16109.65)(796.5919, 13963.3)(1566.1431, 3882.28)(1567.1462, 3834.2)(1568.159, 13270.94)(1569.1627, 11626.77)(772.5867, 137850.16)(773.5896, 63566.58)(774.59, 18022.64)(775.5829, 4748.85)(776.5891, 507.6)(1561.1517, 2546.93)	1584085	410981.6	1573957	618884.3	1581301	506830.7	1315289	566343.5	-0.04	0.895858	0.931428	-0.43	0.213094	0.520485
COMPOUND_771_5798a12_543237	Rp	+	771.5798	12.54				(1544.1539, 15399.08)(1545.1545, 5999.4)(1546.1499, 2384.68)(796.5857, 109028.01)(797.5884, 52784.38)(798.5881, 17270.49)(1566.1432, 15881.93)(1567.1471, 15890.14)(772.5858, 133415.55)(773.589, 61854.75)(774.5707, 23391.93)(775.5874, 2022.78)(1561.148, 718.39)	1517173	434569.2	1642008	595886.5	1565668	579140.2	1287184	406682.6	0.06	0.83794	0.886862	-0.73	0.036138	0.252742
COMPOUND_772_5718a11_902627	Rp	-	772.5718	11.90				(807.5036, 1445.7)(808.5072, 750.05)(771.5645, 14363.99)(772.5673, 6858.49)(773.569, 1868.41)	114370.5	30124.97	119593.8	50074.28	118919.5	36348.02	115768.5	34451.16	-0.04	0.862179	0.906879	-0.27	0.367123	0.669574
COMPOUND_773_5349a12_536672	Rp	-	773.5349	12.54	PE 40:8e	2	20 eV: 772.5273(100), 327.2316(55), 462.2971(11), 59.3957(4)	(808.5127, 3180.0)(809.5147, 1459.04)(772.5284, 202065.95)(773.5315, 100919.99)(774.5344, 28356.93)(775.5381, 6013.56)(1546.123, 820.43)	260259.5	167835.4	291951.3	191195	457577.6	275318.4	406837.7	195434.9	1.00	0.000371	0.001051	-0.25	0.421932	0.702743
COMPOUND_773_5486a12_54078	Rp	+	773.5486	12.54				(774.5673, 15400.8)(775.5554, 5849.71)(776.5537, 1815.37)(777.5389, 476.17)(1565.1362, 5301.46)(1566.143, 12121.6)(1567.1482, 14333.67)	179655.2	52119.41	179182.8	57952.05	235014.9	54897.59	225914.3	46614.01	1.09	2.51E-05	9.12E-05	-0.12	0.677835	0.851748
COMPOUND_773_5572a11_711032	Rp	+	773.5572	11.71				(1548.0989, 214.19)(796.5442, 5524.57)(797.541, 3495.97)(1570.1517, 177.77)(774.5663, 38344.84)(775.5691, 19194.61)(776.5609, 7169.74)(777.5683, 619.22)	81188.62	39952.19	86627.88	35643.68	96421.22	29690.19	94259.6	31362.63	0.48	0.047958	0.084508	0.24	0.402395	0.691103
COMPOUND_773_5596a11_177821	Rp	+	773.5596	11.18	TAG 48:14 (M+NH4)+	2	10 eV: 796.549(100), 184.0744(4)	(1548.1215, 42134.83)(1549.1244, 39312.96)(1550.1268, 18654.2)(1551.1263, 6342.17)(1552.1276, 2224.56)(1553.1489, 450.09)(1586.1012, 14556.21)(1587.1019, 14739.38)(1588.1132, 20715.04)(1589.1176, 15092.19)(1590.1251, 14471.47)(796.5507, 311437.22)(797.5536, 142465.95)(798.5653, 584078.7)(799.57, 263296.62)(1570.0977, 5585.17)(1571.0995, 5300.22)(1572.1143, 14465.02)(1573.1187, 11074.01)(1574.1288, 15505.81)(1575.1324, 12099.69)(1576.1317, 6004.93)(1577.1285, 2759.4)(774.5646, 1097061.1)(775.5686, 480686.78)(776.5722, 125555.26)(777.5748, 25562.05)	17720755	3688306	18063043	3591617	14160335	10353479	9603734	9509513	-1.02	1.25E-05	4.94E-05	-0.23	0.366503	0.669574
COMPOUND_773_593a12_982844	Rp	+	773.5930	12.98				(1548.1841, 1934.32)(1549.188, 844.21)(1586.172, 3071.24)(1570.1743, 9173.99)(774.6005, 20395.34)(775.6034, 9523.97)(776.6079, 3368.57)(777.6086, 937.1)(778.6124, 202.62)(1565.1584, 725.84)(1566.1514, 1071.61)	205509.5	60677.84	214478.8	78357.3	189939	78480.35	204241.5	64846.58	0.00	0.987201	0.994444	-0.17	0.572491	0.789498
COMPOUND_773_5946a13_136387	Rp	+	773.5946	13.14	PC 35:1	2	20 eV: 774.595(100), 184.0738(75), 589.3417(8)	(1548.1812, 1393.96)(1549.1774, 638.68)(1586.1699, 919.09)(1570.1744, 3708.55)(774.6002, 7160.27)(775.6049, 3345.41)(776.6059, 883.01)(777.6129, 240.29)(778.6331, 44.21)(1565.2189, 120.8)(1566.2123, 143.18)	314367	122303.1	303283.2	157703.6	263488	80452.69	301109.8	120561.9	-0.34	0.241242	0.336602	0.45	0.188978	0.504029
COMPOUND_774_5871a12_172709	Rp	-	774.5871	12.17	SM d36:2	2	20 eV: 713.5605(100), 773.5809(14), 57.2381(3), 303.2307(2)	(887.5336, 1299.34)(888.5076, 531.04)(809.5516, 4195.67)(810.5558, 1683.55)(811.5652, 494.35)(773.5808, 70373.66)(774.5834, 32393.76)(775.5868, 9041.68)	350736.2	99221.92	346745.9	123746.6	302026	72821.3	290567.3	86468.91	-0.42	0.106275	0.171687	-0.16	0.604715	0.810123
COMPOUND_775_5351a11_91673	Rp	-	775.5351	11.92	PC 14:0_18:2	1	10 eV: 774.5282(100), 714.5068(28), 279.2306(6)	(1586.0536, 357.36)(1587.0162, 224.31)(1588.0568, 242.96)(1589.0491, 319.13)(888.5114, 3381.55)(889.5128, 2059.49)(890.5126, 3285.87)(891.5042, 1639.31)(1596.0667, 1933.5)(1597.0646, 1305.06)(1598.0643, 814.96)(774.5286, 266096.34)(775.5317, 119055.49)(776.5344, 31527.01)(777.5369, 6574.57)(778.5402, 1531.02)(1550.091, 838.13)(1551.1012, 688.84)	1239672	389440.4	1480314	420057.4	2046325	539033.3	2011385	557945.1	1.37	1.28E-07	7.82E-07	-0.51	0.059186	0.30009
COMPOUND_775_5355a11_413654	Rp	-	775.5355	11.41				(1596.084, 562.28)(774.5284, 16815.96)(775.5314, 7857.09)(776.5344, 2631.37)(777.537, 795.95)	25483.85	14362.17	31750.55	19586.87	34204	26109.82	34205.13	17960.5	-0.85	0.000245	0.000717	-0.30	0.2558	0.56678

									1st trimester				3rd trimester									
									Control		Pre-eclampsia		Control		Pre-eclampsia		Time			Interaction		
Compound	Column	Ion_mode	Mass	Retention_	Identification	ID_level	MSMS_spectra	CompositeSpectrum	Mean	SD	Mean	SD	Mean	SD	Mean	SD	Value	raw p	FDR_p	Value	raw p	FDR_p
COMPOUND_775_5508a13_108693	Rp	-	775.5508	13.11				(1596.1447, 414.47)(774.5428, 16215.62)(775.5467, 8189.79)(776.5493, 3506.05)(777.55, 1150.1)	81038.98	35677.56	83835.65	44493.35	102681.5	45942.34	85529.19	33008.6	0.51	0.065653	0.11269	-0.32	0.316832	0.622922
COMPOUND_775_568a9_826908	Rp	+	775.5680	9.83				(796.1017, 180.81)(796.6013, 131.69)(399.7829, 1034.58)(400.2845, 772.38)(400.7904, 167.13)(397.3052, 6441.29)(397.807, 2583.62)(398.3092, 1079.6)(398.8056, 147.02)(416.2958, 758.12)(416.7924, 140.56)(776.5732, 19880.76)(777.5762, 8685.56)(778.5638, 2947.04)(779.5447, 910.03)(780.5654, 219.8)(388.7922, 4628.99)(389.2932, 2080.88)(389.7942, 609.18)	356720.3	90761.51	309647	64398.71	383174.6	60668.14	383195	99497.7	0.23	0.418276	0.52284	0.59	0.088828	0.352184
COMPOUND_775_5682a11_317031	Rp	+	775.5682	11.32				(1590.1255, 514.37)(1591.13, 563.4)(1592.1157, 598.08)(1574.1295, 1610.11)(1575.133, 978.79)(1576.1313, 528.66)(1577.1154, 226.76)(776.5796, 16659.11)(777.5841, 6684.22)(778.5709, 2069.38)(779.5817, 483.41)	246784.2	100858.6	236272.6	93269.51	157445.7	21870.55	192122.8	43638.14	-1.02	4.25E-06	1.83E-05	-0.14	0.568542	0.788601
COMPOUND_776_1413a11_090051	Rp	+	776.1413	11.09				(799.1483, 1426.65)(777.1495, 13990.97)(778.1424, 5860.36)(779.1313, 2011.57)	48886	30670.06	47710.09	31834.5	57449.55	10291.94	58077.52	19774.02	0.37	0.181743	0.26909	0.07	0.837049	0.929046
COMPOUND_776_6028a12_662439	Rp	-	776.6028	12.66	N-Stearoyl-D-sphingomyelin	1	40 eV: 715.5744(100), 168.0425(37), 279.2326(22), 78.9582(12)	(889.6011, 278.96)(890.587, 1365.76)(891.5915, 647.55)(775.5957, 26065.15)(776.598, 12195.21)(777.6006, 3398.35)	203085.8	65694.47	206193.1	79039.39	204006.2	60123.09	192039.1	58173.5	0.08	0.799881	0.856553	-0.22	0.527076	0.7696
COMPOUND_777_534a11_744952	Rp	+	777.5340	11.74	PC 36:6	2	10 eV: 778.5372(100), 184.0763(13), 585.1288(4)	(1556.0393, 529.81)(1557.0668, 505.45)(1558.0813, 588.07)(1559.081, 501.76)(800.5178, 14184.54)(801.5096, 9588.88)(802.5237, 7083.08)(803.536, 2303.65)(1578.041, 383.7)(816.4896, 1798.09)(817.4824, 1351.06)(818.4991, 1394.89)(778.5403, 104717.09)(779.5431, 49037.13)(780.5536, 60552.5)(781.5574, 24955.78)	528276.4	259159.9	580013	336967.3	740998.5	283798.6	818888	415139.1	0.31	0.190326	0.279227	0.27	0.329273	0.635357
COMPOUND_777_5515a12_213564	Rp	-	777.5515	12.21				(776.5439, 484813.25)(777.5477, 215318.0)(778.5501, 55602.03)(779.5529, 11251.1)(780.5541, 1898.91)(781.5527, 298.75)	1284858	446382.2	1384043	658074.9	1999348	612527.8	2648651	998300.2	0.87	0.000196	0.000585	0.42	0.108008	0.39105
COMPOUND_778_9422a11_136764	Rp	+	778.9422	11.14				(801.9089, 1330.29)(796.9613, 1354.52)(779.9503, 14007.39)(780.9449, 5283.59)(781.9488, 2130.03)	29616.61	26615.07	35332.79	31476.66	46184.8	22729.25	49288.05	22500.02	0.38	0.161682	0.243714	0.06	0.844004	0.93246
COMPOUND_779_1459a11_136102	Rp	+	779.1459	11.14				(802.1548, 985.34)(818.0683, 1293.23)(780.1501, 16921.94)(781.143, 3181.35)(782.1343, 936.83)	38867.12	25196.52	33534.41	28561.61	40311.6	9359.385	44995.09	17622.27	0.04	0.870411	0.912554	0.61	0.047602	0.279163
COMPOUND_779_5084a10_922651	Rp	-	779.5084	10.92				(778.5019, 15678.32)(779.5055, 7546.3)(780.5157, 10811.33)(781.5189, 5067.64)(782.5216, 3420.9)(783.5304, 1075.51)(784.5194, 884.38)(1557.9875, 216.44)(1559.0018, 268.31)(1560.007, 283.12)	139970.3	75405.06	139430.6	62675.36	140676.7	28274.73	142340.6	44338.55	-1.01	1.39E-05	5.4E-05	-0.09	0.735057	0.888627
COMPOUND_779_5098a11_194105	Rp	-	779.5098	11.19				(1593.9368, 174.6)(778.5021, 8832.79)(779.5052, 4305.64)(780.5147, 4307.46)(781.5191, 1905.23)(782.5296, 2259.59)(1558.0624, 283.19)(1559.0826, 249.67)	310177.9	116592.6	375131.9	234922.1	611269.5	173609.5	639118.2	282940.9	-0.64	0.010799	0.022665	-0.35	0.231189	0.537849
COMPOUND_779_5345a12_288433	Rp	+	779.5345	12.29				(1598.1229, 3883.23)(791.5436, 10166.67)(792.0331, 1899.89)(792.5518, 3910.89)(793.0345, 729.92)(409.7412, 1087.58)(410.2378, 748.93)(800.0403, 2890.02)(800.5489, 1809.46)(801.0415, 13863.15)(818.5058, 823.69)(819.0125, 446.12)(818.5396, 1289.71)(780.5521, 72005.9)(781.5552, 33811.2)(782.5703, 1098514.1)(783.5743, 506261.75)(1577.132, 4204.65)(390.7767, 6980.09)(391.2773, 4444.33)(391.7772, 3136.3)(392.2724, 963.97)(392.7734, 158.74)	19511214	4080319	15655826	6966814	8933607	9265509	12210064	9634828	-0.71	0.016931	0.034014	0.63	0.073255	0.326093
COMPOUND_779_5478a11_878703	Rp	+	779.5478	11.88	PC 16:1_20:4	1	10 eV: 780.5553(100), 184.0729(10), 494.0954(3)	(1560.0961, 9639.33)(1561.1, 8245.0)(1562.1108, 9476.21)(1563.1149, 6439.66)(1564.1115, 3107.91)(1565.105, 1551.79)(1598.0374, 1397.99)(1599.0468, 1675.92)(802.5379, 6127.32)(803.5369, 3258.42)(804.5571, 146720.44)(1582.069, 2797.75)(1583.073, 2500.68)(1584.0868, 2632.0)(1585.0919, 2420.06)(818.5162, 4562.53)(819.5176, 2962.44)(820.5293, 3129.27)(797.5471, 4160.73)(780.555, 992129.9)(781.5593, 465964.88)(782.5662, 203582.08)(783.5704, 60975.91)(1577.0771, 2059.4)	7188953	8996697	2920316	4510985	8071244	9008944	5237687	7961148	0.28	0.284481	0.383853	0.25	0.428701	0.707279
COMPOUND_779_5488a11_980451	Rp	+	779.5488	11.98	PC(16:1_20:4) duplicate			(1560.097, 17668.04)(1561.1001, 16317.86)(1562.1108, 21588.36)(1563.1143, 16509.61)(1564.1138, 9003.92)(1565.1068, 4066.67)(1598.0554, 2409.82)(1599.0562, 2266.53)(802.5365, 40018.91)(803.5369, 19739.46)(804.5539, 47337.24)(805.5568, 22349.94)(1582.0717, 4059.56)(1583.0767, 3478.14)(1584.0918, 5384.03)(1585.0925, 5235.28)(797.5483, 4958.11)(818.5109, 4112.02)(819.511, 3544.16)(820.5248, 3927.44)(780.5566, 388594.97)(781.5602, 180066.66)(782.5699, 216934.73)(783.5744, 89331.72)(784.5785, 26334.7)(785.5813, 6673.65)(1577.076, 2483.09)	12643831	6643172	11387092	5683450	13631398	5996660	13779005	6554713	-0.01	0.974675	0.98606	0.42	0.189996	0.505078
COMPOUND_779_5516a11_206111	Rp	+	779.5516	11.21				(1560.1, 2152.57)(1582.122, 75069.05)(780.5552, 86810.25)(781.5581, 41152.35)(782.571, 180078.83)(783.574, 82190.7)(784.5827, 62104.36)(785.5854, 24755.95)(786.5794, 13350.07)(1577.1042, 2942.36)	2977510	1374666	2835822	1550505	3686868	1849743	2639746	1553208	-0.95	3.33E-05	0.000117	-0.08	0.747636	0.891783
COMPOUND_779_5658a12_689466	Rp	-	779.5658	12.69	PC 16:0_16:0	1	10 eV: 778.5577(100), 255.2323(6), 23.7679(2)	(778.5592, 66019.84)(779.5621, 30991.03)(780.5642, 8808.21)(781.5639, 2864.11)(1558.1257, 2066.04)(1559.145, 2128.29)(1560.1359, 2103.28)(824.5701, 6938.04)(825.5758, 3476.34)	382831.8	133023.8	397585.1	156155.6	345168.6	100819.1	381927	162739.2	-0.32	0.246333	0.341589	0.26	0.429787	0.707514
COMPOUND_781_5515a12_730271	Rp	+	781.5515	12.73				(802.0546, 1768.46)(802.5579, 2012.48)(803.0557, 12008.11)(782.5684, 130418.77)(783.5713, 59567.62)(1581.1733, 2676.39)(391.7831, 5057.99)(392.2844, 2902.44)(392.7843, 1531.02)(393.2848, 452.56)(393.7792, 91.69)	1336533	309123.2	1364066	280569.2	1509853	308422.4	1365399	306867.2	0.55	0.070523	0.119735	-0.66	0.07062	0.325554
COMPOUND_781_5619a11_260242	Rp	+	781.5619	11.26	PC 36:4	2	20 eV: 782.5784(100), 184.0736(65), 338.2386(4)	(1564.1177, 20372.01)(804.5576, 96837.49)(805.5601, 48000.33)(1586.0994, 6822.34)(1587.1011, 6775.34)(1588.1122, 10366.58)(1589.1158, 7826.63)(1590.124, 7954.45)(782.5703, 110902.2)(783.5735, 50056.48)(784.5859, 104388.0)(785.5894, 46141.52)(786.5928, 22223.45)(787.5934, 7438.69)(391.7908, 416.08)(392.2895, 3598.95)(392.7951, 72.51)	2758260	1353608	2175217	1306743	3015929	2690905	1871654	1397726	-1.04	5.09E-06	2.16E-05	0.10	0.674185	0.849616
COMPOUND_781_5627a12_260051	Rp	+	781.5627	12.26	PC 36:4	2	20 eV: 782.5729(100), 184.0737(85), 502.3351(1)	(1564.1317, 36288.83)(1565.138, 33906.4)(1566.1326, 41399.55)(1567.1318, 28858.98)(1568.1305, 14294.63)(1569.1208, 5831.79)(1570.1208, 2697.27)(804.5511, 49176.46)(805.5546, 24956.89)(1586.1093, 3888.67)(1587.1106, 3371.77)(1588.1304, 19553.95)(1589.1345, 19285.18)(1590.1426, 29942.8)(1591.1486, 24591.18)(1592.149, 12509.81)(1593.1414, 5236.18)(1594.1206, 2624.61)(799.5609, 1123.63)(800.5259, 2729.16)(820.5574, 3470.99)(821.5816, 616.86)(822.5538, 4120.34)(782.5691, 1415447.8)(783.5731, 669907.44)(784.5768, 174998.72)(785.579, 30857.43)(786.5824, 5478.22)	32060377	6803657	28950000	6844649	33214286	4671494	35242105	7546616	0.44	0.072678	0.122508	0.45	0.116847	0.405455
COMPOUND_781_5642a12_085624	Rp	+	781.5642	12.09				(1564.1305, 11869.55)(1565.1344, 10807.01)(1566.1366, 5750.96)(1567.138, 2369.86)(1568.1184, 1073.47)(804.5537, 42300.05)(805.5556, 21088.63)(806.5731, 489678.28)(1586.1134, 8569.1)(1587.118, 8383.96)(1588.128, 11623.68)(1589.1327, 8858.68)(1590.1329, 4759.93)(1591.1265, 2167.2)(1592.1042, 1313.25)(1593.0898, 1250.66)(1594.0878, 1672.31)(820.5351, 3775.67)(821.5346, 2923.48)(822.5538, 3578.48)(823.5434, 2335.69)(782.571, 1052432.4)(783.5752, 485193.84)(784.5781, 124764.13)(785.5802, 23593.98)(786.5822, 4766.05)(787.5904, 545.82)(1581.0857, 1066.62)(1582.0784, 2038.21)	9736373	3096082	9349826	3467017	9657695	2753727	8941926	3673717	-0.04	0.901289	0.935347	-0.11	0.747568	0.891783
COMPOUND_782_1479a11_030399	Rp	+	782.1479	11.03				(805.1448, 2119.99)(806.1595, 3508.35)(800.2177, 2315.85)(783.1537, 9281.18)(784.1434, 4715.41)(785.1366, 2376.96)	95947.84	53712.83	83111.34	57912.63	104330.8	40075.56	76939.2	36436.25	0.21	0.457534	0.562427	-0.08	0.816812	0.92157
COMPOUND_782_568a13_892386	Rp	-	782.5680	13.89				(781.5618, 14135.55)(782.5648, 7242.05)(783.5689, 2435.53)(784.5719, 687.12)(785.5813, 151.46)(827.5641, 2608.49)	77598.83	49376.42	73020.71	37737	33175.33	4497.657	29613.11	14012.31	-1.41	3.04E-11	6.26E-10	0.03	0.881525	0.950612

									1st trimester				3rd trimester									
									Control		Pre-eclampsia		Control		Pre-eclampsia		Time			Interaction		
Compound	Column	Ion_mode	Mass	Retention_	Identification	ID_level	MSMS_spectra	CompositeSpectrum	Mean	SD	Mean	SD	Mean	SD	Mean	SD	Value	raw p	FDR_p	Value	raw p	FDR_p
COMPOUND_782_5688a14_010123	Rp	-	782.5688	14.01				(781.5611, 13787.71)(782.5648, 7127.21)(783.5691, 2449.86)(784.5726, 642.42)(785.5737, 116.69)(827.5629, 1804.28)	73715.28	47032.39	73153.3	36493.17	35816	914.0804	40766.14	13392.9	-1.25	5.38E-09	4.68E-08	-0.09	0.680483	0.853001
COMPOUND_783_1643a9_241846	Rp	-	783.1643	9.24				(782.1558, 5568.78)(783.1567, 1387.68)(784.1541, 544.88)	17075.65	2987.297	15804.43	3067.72	15817.29	1442.8	16547.48	3501.936	-0.29	0.322367	0.423801	0.48	0.175802	0.490747
COMPOUND_783_5771a11_348161	Rp	+	783.5771	11.35	PC 36:3	2	40 eV: 184.0725(100), 86.0952(8), 524.3809(1)	(1568.1046, 1807.45)(806.5701, 47893.04)(807.5732, 22706.62)(808.5853, 53846.89)(809.5887, 25914.29)(810.5884, 13662.83)(795.5563, 2306.14)(796.0605, 612.88)(1590.1272, 88.74)(422.7497, 645.51)(423.2541, 8610.38)(411.774, 1195.43)(412.2739, 657.14)(412.7833, 677.66)(413.2904, 262.47)(413.7797, 175.22)(403.7842, 914.01)(404.2886, 829.41)(404.7865, 567.54)(784.5881, 163490.72)(785.5911, 75326.05)(786.5923, 31548.52)(787.5925, 9929.67)(430.7584, 1297.97)(431.2646, 1160.25)(431.7655, 2102.42)(432.2719, 1175.88)(1585.1199, 1651.42)(392.8006, 441.27)(393.2987, 499.95)(393.7978, 86.09)	1048162	434972.8	1020556	492590.9	986512.3	536335.8	1144413	550933.7	-1.07	6.54E-06	2.71E-05	-0.08	0.768095	0.902789
COMPOUND_783_5789a12_453448	Rp	+	783.5789	12.45				(1568.1556, 92621.88)(1569.148, 37749.98)(1570.1388, 11969.64)(1571.133, 3729.89)(806.5702, 110439.18)(807.573, 53346.88)(808.5861, 1138609.9)(809.5902, 554917.5)(1590.1414, 9545.5)(1591.1426, 9218.74)(1592.1523, 9567.62)(1593.153, 7418.65)(1594.1532, 6082.66)(1595.156, 3855.36)(1596.1486, 2283.03)(1597.129, 1990.44)(1598.1266, 2426.06)(822.582, 6922.56)(823.589, 2995.13)(824.568, 3714.02)(825.5579, 1956.88)(784.587, 673403.6)(785.5911, 320515.03)(786.594, 78664.95)(787.5958, 15341.71)	22971698	12625149	25328261	11072702	20007143	3361882	30567904	21654933	-0.07	0.82357	0.874436	0.08	0.818036	0.92157
COMPOUND_783_5797a11_452839	Rp	+	783.5797	11.45				(1568.1255, 326.95)(795.5598, 3936.08)(796.056, 1185.29)(806.5707, 11776.91)(807.5725, 5992.23)(808.5857, 28301.7)(809.5887, 14531.0)(810.6004, 25875.01)(1590.142, 1716.91)(422.765, 980.48)(423.2621, 908.57)(423.7666, 1390.89)(424.2679, 965.61)(411.7733, 1697.72)(412.2776, 1181.56)(412.7829, 1398.37)(413.2869, 690.64)(413.7839, 368.6)(403.784, 1724.56)(404.288, 1348.21)(404.7901, 731.11)(784.588, 267058.2)(785.5911, 124430.77)(786.5931, 54029.5)(787.5949, 16592.32)(430.7586, 3217.32)(431.2608, 1890.27)(431.7665, 2828.63)(432.268, 1552.85)(1585.142, 109.17)(392.799, 1539.2)(393.2998, 775.63)(393.7884, 129.38)	1827091	679945.1	1873837	730887.4	1245792	742685	1042510	524416.1	-1.25	4.14E-09	3.78E-08	-0.20	0.363649	0.668521
COMPOUND_783_5803a12_51953	Rp	+	783.5803	12.52	PC 36:3	2	10 eV: 784.5882(100), 184.0719(10)	(1568.1611, 5351.38)(1569.1676, 4876.37)(1570.1666, 2842.3)(1571.1691, 1518.32)(1572.154, 750.13)(806.5707, 89378.13)(807.5737, 44626.38)(808.5871, 256320.98)(809.5913, 123317.89)(810.6029, 338680.44)(811.6068, 157238.28)(1590.146, 3654.79)(1591.1431, 3691.39)(1592.157, 5243.59)(1593.1599, 4424.36)(1594.1758, 10063.21)(1595.1805, 10017.89)(1596.1815, 4873.76)(1597.1716, 2709.0)(1598.1449, 1403.34)(822.5813, 8676.88)(823.5883, 4139.44)(824.5927, 4982.72)(825.6077, 2217.18)(784.5854, 1228777.0)(785.5895, 566754.5)(786.5927, 149374.53)(787.5948, 27926.74)	36052830	10404319	35776087	11185987	41542857	6782411	50052632	13005083	0.64	0.008645	0.018615	0.51	0.070148	0.324382
COMPOUND_784_6445a0_8248375	Hilic	+	784.6445	0.82				(807.6353, 13118.09)(808.6379, 6776.35)(809.6525, 16426.62)(810.6551, 8657.6)(811.6692, 60824.63)(802.6712, 1675.74)(803.6657, 1416.0)(785.653, 46400.39)(786.6535, 22304.42)(787.6622, 11150.1)(788.6653, 3493.76)(789.6707, 793.78)(790.6787, 138.95)(393.3243, 321.43)(393.8292, 27.34)	495413.9	190207.9	541693.8	215655.8	367758.8	138194.6	410167.4	152035.1	-0.74	0.011965	0.024858	0.08	0.817438	0.92157
COMPOUND_784_6452a0_7917568	Hilic	+	784.6452	0.79				(807.6354, 22786.05)(808.6357, 11534.36)(809.6521, 24468.64)(810.653, 12163.02)(811.669, 83287.57)(802.683, 1609.44)(803.6864, 307.75)(785.6531, 78870.36)(786.654, 40907.4)(787.6597, 15329.31)(788.6628, 4103.68)(789.6665, 560.78)(393.3221, 249.83)(393.8308, 89.82)	588455.4	299260	707953.3	359701.5	410053.1	161386	413748.4	176885.1	-0.65	0.018315	0.036565	-0.36	0.254614	0.566592
COMPOUND_784_9497a11_077688	Rp	+	784.9497	11.08				(1570.9233, 336.56)(1571.9218, 182.51)(1572.9121, 215.67)(807.9529, 2700.87)(823.9048, 3363.8)(824.905, 1001.69)(825.9145, 2064.82)(802.9592, 2809.66)(785.953, 13243.06)(786.9458, 5449.06)(787.9413, 1831.74)	80973.12	61397.66	77898.95	77158.59	87654.63	28667.72	73653.98	28137.4	0.28	0.286786	0.385808	0.20	0.520694	0.764408
COMPOUND_785_1398a11_079318	Rp	+	785.1398	11.08				(808.1572, 2248.68)(824.078, 8458.89)(825.0762, 4261.94)(803.161, 3068.08)(786.1535, 14328.22)(787.1435, 4953.34)(788.1332, 2497.02)	87854.47	60549.64	82691.22	64132.36	76863.11	24221.63	78975.07	23015.98	-0.01	0.976596	0.987116	0.23	0.491564	0.748114
COMPOUND_785_472a12_178252	Rp	+	785.4720	12.18				(1571.8972, 170.68)(805.4364, 283.39)(805.9324, 585.7)(1593.872, 183.43)(1594.9082, 204.13)(393.7441, 5221.61)(394.2459, 2351.5)(394.7478, 818.84)(395.2425, 217.85)	17947.03	5156.04	20262.2	7889.815	26860.69	7989.537	32744.57	9272.643	0.84	0.001149	0.002954	0.02	0.95771	0.980661
COMPOUND_785_5946a12_972127	Rp	+	785.5946	12.97	PC 36:2	2	10 eV: 786.6024(100), 184.0735(10)	(1572.1913, 9160.99)(1573.1956, 8058.97)(1574.1963, 4315.29)(1575.2001, 1678.47)(1576.1898, 716.92)(1577.216, 287.57)(808.5836, 51725.89)(809.5868, 25793.8)(810.6016, 617767.2)(1594.1736, 5261.08)(1595.1758, 5451.08)(1596.1896, 17772.86)(1597.1956, 15660.71)(1598.1964, 8956.83)(1599.1968, 4119.13)(824.5794, 6477.07)(825.5688, 4626.43)(826.5709, 3501.96)(803.6064, 117.93)(804.5646, 3032.88)(786.6031, 701191.6)(787.607, 325981.8)(788.6093, 83791.08)(789.6114, 15774.23)(790.6035, 3241.25)(791.6148, 611.54)(1589.1613, 971.48)	32233962	8136195	34967391	10374693	32657143	5867999	32063158	10663131	0.09	0.743604	0.814256	-0.47	0.158644	0.468876
COMPOUND_787_5381a11_02854	Rp	+	787.5381	11.03				(1576.0942, 1637.76)(1577.0883, 1138.07)(810.5351, 30193.9)(811.5362, 14993.31)(812.5473, 122482.17)(813.5506, 58175.16)(814.563, 417321.22)(788.5462, 82442.23)(789.5488, 39127.08)(790.5614, 140565.67)(791.5642, 61307.83)(792.5601, 20629.04)(793.5525, 6093.2)(794.5358, 48810.22)	1844093	1063453	1942637	966363.4	1325593	1142049	1492476	808482.6	-0.94	1.99E-05	7.44E-05	-0.37	0.133467	0.434347
COMPOUND_787_5721a12_641872	Rp	-	787.5721	12.64				(900.5294, 1823.12)(901.5328, 1319.98)(786.5646, 84869.68)(787.5679, 39888.03)(788.5715, 12046.71)(789.5739, 3152.07)(1574.1451, 2263.33)(1575.1422, 1598.87)	709765.3	170049.5	724033.1	201291.2	545571.6	141905.2	532897	130208.7	-1.02	8.19E-05	0.000256	0.07	0.81452	0.92157
COMPOUND_787_61a13_585232	Rp	+	787.6100	13.59	PC 18:0_18:1	1	10 eV: 788.614(100), 184.0717(5)	(1576.2241, 1095.58)(1577.2288, 986.55)(1578.2292, 677.82)(1579.2303, 227.57)(810.5996, 30455.28)(811.6039, 14406.38)(812.6167, 34618.48)(813.6207, 16961.26)(1598.2047, 337.98)(1599.2261, 401.42)(826.6073, 2581.75)(827.5922, 723.1)(828.5782, 1196.77)(788.6183, 218199.19)(789.6212, 104121.55)(790.6239, 27335.47)(791.6268, 6011.3)(792.6196, 1486.21)(793.6203, 285.98)(1593.2726, 99.56)(394.8079, 203.69)(395.3148, 103.52)	1889318	979640.6	2001226	1372223	1648839	739787.3	2200653	1123317	-0.19	0.463518	0.568542	0.54	0.084827	0.346484
COMPOUND_788_146a10_979037	Rp	+	788.1460	10.98				(811.1523, 2444.52)(806.1533, 1811.89)(789.1515, 8108.01)(790.1447, 3442.61)(791.1359, 1461.3)	42169.79	28116.19	40915.47	33220.83	37211.5	11609.16	36338.49	14512.09	-0.36	0.203537	0.294704	0.47	0.166053	0.47881
COMPOUND_788_1517a11_122728	Rp	+	788.1517	11.12				(811.1471, 1922.44)(806.1698, 453.18)(789.1613, 10755.96)(790.1453, 4057.19)(791.1567, 558.64)	37763.5	18581.48	40793.27	39714.82	31230	9521.058	40173.28	12189.3	0.22	0.338422	0.439013	0.14	0.599068	0.805434
COMPOUND_789_3917a13_932123	Rp	-	789.3917	13.93				(788.3843, 8288.85)(789.3875, 3772.74)(790.3828, 5967.38)(791.385, 2515.35)(792.3825, 1312.74)(793.3823, 579.3)(794.3831, 184.86)	59421.79	15472.42	61886	18045.18	69133.14	19833.59	88163.68	36375.41	0.38	0.171355	0.255557	0.42	0.207178	0.518872
COMPOUND_789_5305a12_300298	Rp	-	789.5305	12.30				(788.5226, 47366.86)(789.526, 23633.83)(790.5305, 7022.79)(791.5361, 1830.75)(834.5598, 1732.67)(835.5662, 972.5)	42622.89	17323.73	52827.38	22601.88	199129.1	70595.66	252287.4	90838.12	1.31	2.96E-10	3.97E-09	0.31	0.141355	0.447724
COMPOUND_789_548a12_124758	Rp	-	789.5480	12.12	PC 18:2_15:0	1	20 eV: 728.5216(100), 788.5414(27), 279.2317(13), 44.9981(4), 431.2307(3)	(788.5438, 85184.38)(789.5471, 38191.77)(790.535, 17302.87)(834.5217, 4600.52)(835.5286, 2079.11)(1578.0981, 220.67)(1579.1332, 2210.81)	446416.8	90730	502214.4	111855.1	609469.6	146278.5	507148.6	106986.1	0.06	0.829808	0.880229	-0.55	0.076225	0.331641
COMPOUND_789_5534a11_143794	Rp	+	789.5534	11.14	PC 38:7e	2	20 eV: 790.5613(100), 184.0714(60)	(1580.109, 20192.86)(1581.1138, 18781.15)(1582.1115, 10444.14)(1583.0988, 6811.81)(812.5409, 96216.7)(813.5435, 46228.94)(814.559, 130489.13)(815.5628, 61994.29)(816.5756, 428771.75)(790.5609, 781496.3)(791.5654, 350220.84)(792.5674, 101465.34)(793.5652, 22941.04)	10070348	3802946	10178659	3978602	5643174	5357180	4824664	3233161	-1.23	6.31E-09	5.41E-08	-0.22	0.316776	0.622922

									1st trimester				3rd trimester									
									Control		Pre-eclampsia		Control		Pre-eclampsia		Time			Interaction		
Compound	Column	Ion_mode	Mass	Retention_	Identification	ID_level	MSMS_spectra	CompositeSpectrum	Mean	SD	Mean	SD	Mean	SD	Mean	SD	Value	raw p	FDR_p	Value	raw p	FDR_p
COMPOUND_789_5655a12_40603	Rp	+	789.5655	12.41				(812.5593, 10053.52)(813.5527, 3990.45)(814.5524, 6246.6)(790.5748, 56600.09)(791.5779, 27737.52)(792.5909, 72185.19)(793.594, 34001.44)(794.5921, 10899.14)(795.5823, 4266.09)(796.5854, 22221.28)	661771.2	229214.9	591519.9	177724.9	658516.1	154366.3	718389.2	156640.7	0.13	0.615634	0.708436	0.51	0.094008	0.36381
COMPOUND_789_5832a10_038973	Rp	+	789.5832	10.04				(812.5429, 619.01)(813.0577, 616.63)(813.5598, 1024.97)(1580.1956, 83.73)(406.7935, 720.18)(407.298, 664.23)(407.7912, 155.83)(828.5557, 1027.0)(829.5667, 846.14)(807.6484, 195.24)(423.2752, 389.79)(423.7871, 50.15)(424.2626, 551.39)(404.3113, 7186.26)(404.8151, 2562.17)(405.312, 1242.87)(405.8187, 173.08)(790.589, 16392.5)(791.5915, 7182.02)(792.5834, 1262.64)(793.5916, 194.04)(395.7994, 2985.94)(396.3011, 1677.05)(396.8036, 490.1)	182562.9	56442.33	162125.2	44068.84	198321	35206.88	199646.2	55051.81	0.63	0.015782	0.032048	0.29	0.346486	0.651869
COMPOUND_789_5869a13_163428	Rp	-	789.5869	13.16				(824.55, 3336.2)(825.5507, 1590.78)(788.5795, 18142.78)(789.5835, 8720.51)(790.5843, 4185.0)(791.5915, 1367.03)(1578.1458, 879.88)(1579.1478, 747.33)(1580.1458, 1033.98)	202173.4	73219.02	206264.5	80499.06	134269.4	64040.22	125869.7	49403.2	-1.04	0.00011	0.000338	0.33	0.266257	0.575669
COMPOUND_790_9459a11_021012	Rp	+	790.9459	11.02				(813.9518, 2536.3)(829.905, 1127.66)(808.963, 2318.55)(791.9531, 9763.2)(792.9483, 4797.73)(793.9366, 1568.62)	48284.97	28607.75	43564.78	35361.25	42520.92	14432.01	46878.57	15520.89	0.28	0.307956	0.408913	0.23	0.487964	0.748057
COMPOUND_791_1564a11_02265	Rp	+	791.1564	11.02				(814.157, 650.71)(815.1727, 5898.55)(816.1707, 3418.99)(809.1683, 4806.29)(792.1573, 16567.94)(793.1465, 5175.89)(794.1449, 1814.4)	86277.58	56332.6	71713.14	58842.09	94177.23	41544.74	86221.91	45630.58	0.39	0.140338	0.216188	-0.28	0.36587	0.669574
COMPOUND_791_5324a10_973565	Rp	-	791.5324	10.97				(790.5174, 14510.29)(791.5207, 9329.75)(792.518, 9408.52)(793.5177, 4072.6)(794.5191, 1956.14)(1582.0681, 281.32)(1583.071, 226.21)(1584.0758, 256.31)	132452.7	44402.75	154252.7	48971.42	80143.67	64537.93	81902.87	65923.35	-0.56	0.034112	0.062967	0.20	0.515656	0.761481
COMPOUND_791_5456a11_954262	Rp	+	791.5456	11.95	PC 37:6	2	10 eV: 792.5549(100), 184.0727(11), 463.4809(4)	(1584.091, 8603.26)(814.5301, 4587.05)(815.5285, 2561.8)(816.5165, 1654.53)(817.4988, 447.65)(792.5549, 67741.52)(793.5577, 33511.42)(794.5559, 10365.23)(795.5539, 1069.88)	526808.8	153033.5	442195.6	157477.6	502119.7	175495.2	469217.9	158160.9	0.65	0.014274	0.029303	-0.03	0.926979	0.966461
COMPOUND_791_5461a12_792073	Rp	-	791.5461	12.79				(790.5382, 53994.85)(791.5413, 26547.45)(792.5451, 8268.0)(793.5484, 2112.56)(1582.1462, 867.19)(1583.1497, 732.84)	99071.44	42931.04	126933.7	49018.02	228122.6	91397.97	280680.9	123064.9	1.24	6.55E-08	4.37E-07	0.10	0.683153	0.853695
COMPOUND_791_5549a12_78796	Rp	+	791.5549	12.79	PC 37:6	2	20 eV: 651.5388(100), 792.5743(19), 184.0734(13)	(814.5424, 4251.93)(815.5456, 2871.43)(792.5571, 71108.27)(793.5599, 34746.49)	205588	3016.518	220694	82414.6	303796.5	90847.95	372174.4	112805	0.90	1.79E-07	1.07E-06	0.39	0.033056	0.244873
COMPOUND_791_5832a12_577082	Rp	+	791.5832	12.58	PC 38:6e	2	10 eV: 792.5884(100), 184.0733(12), 644.2719(1)	(1584.1375, 3568.22)(1585.1382, 4064.14)(1586.1375, 3825.15)(814.5728, 15589.18)(815.5691, 10219.4)(816.5843, 13469.11)(792.5921, 177094.97)(793.5951, 85871.67)(794.5933, 30697.11)(795.5874, 9503.99)	1670060	665426.6	1443174	620766.1	1826664	597496.6	1907686	626622.1	0.22	0.406855	0.510438	0.04	0.890389	0.953702
COMPOUND_791_6024a13_421187	Rp	-	791.6024	13.42				(904.6086, 398.62)(905.6095, 246.31)(790.5954, 17057.15)(791.5988, 8049.72)(792.5997, 2321.55)(793.5988, 705.22)	287325.3	97755.54	304435.1	129285.7	260547.1	70677.5	233943.9	80782.65	-0.30	0.261012	0.35776	-0.34	0.273223	0.583098
COMPOUND_793_5387a12_398204	Rp	-	793.5387	12.40				(792.5312, 41583.51)(793.534, 19366.97)(794.5304, 16748.29)	733119.8	104808	753265.2	133403.9	718598.7	87528.69	664651	88793.93	-0.07	0.811707	0.866745	-0.68	0.053594	0.288052
COMPOUND_793_5633a12_2282915	Rp	+	793.5633	12.23				(1588.1338, 107374.44)(1589.1373, 101669.52)(816.5391, 3274.47)(817.5529, 3045.77)(811.5887, 2321.23)(812.6009, 403.22)(813.6191, 69.34)(794.5705, 21892.5)(795.5731, 10793.69)(796.5581, 4492.65)(797.5758, 511.55)	605653.4	300873.4	578824.1	319361.8	737706	446035.5	684964.1	298830.9	0.35	0.232743	0.327385	0.14	0.684839	0.853695
COMPOUND_793_599a12_7826185	Rp	+	793.5990	12.78	PC 38:5e	2	40 eV: 184.0734(100), 86.0966(5), 478.3244(1)	(1588.1493, 2568.0)(816.5811, 13595.86)(817.5824, 7935.82)(794.6076, 256538.0)(795.6105, 127614.98)(796.6121, 34654.71)(797.611, 7350.52)	2380827	685267.9	2135413	697407.8	2225368	342187	2109981	487480.2	-0.15	0.585378	0.680925	0.21	0.511387	0.757661
COMPOUND_793_9485a11_066964	Rp	+	793.9485	11.07				(816.9172, 1234.21)(832.8935, 823.76)(811.9962, 2522.81)(794.9571, 12795.73)(795.9506, 4876.3)(796.9347, 1037.14)	63513.61	35197.6	45401.16	32968.7	42236.38	21179.32	44122.18	20798.22	-0.15	0.554135	0.651651	0.55	0.082247	0.343638
COMPOUND_794_2038a9_581989	Rp	-	794.2038	9.58				(907.174, 605.26)(908.1531, 176.27)(909.1773, 563.16)(829.1787, 68254.48)(830.1818, 18894.55)(831.1797, 2983.14)(832.1747, 451.1)(793.1974, 2368.05)(794.2004, 894.08)(795.1862, 458.37)(796.179, 148.54)(1587.3474, 110.7)	442228.8	51879.9	450866.5	52618.14	435458.9	45479.2	433723.7	55302.29	-0.11	0.712247	0.788624	-0.24	0.504601	0.754687
COMPOUND_794_2042a9_751734	Rp	-	794.2042	9.75				(907.1916, 589.35)(908.1858, 304.28)(909.1701, 458.06)(829.1784, 27087.46)(793.1965, 2579.2)(794.1978, 960.14)(795.2086, 346.79)(1587.4265, 71.74)(1588.428, 76.93)(839.1872, 375.55)(840.1797, 279.84)(841.1774, 247.81)	273357.4	61351.44	272337.2	67005.76	236867.7	67996.99	259117.7	60286.99	-0.07	0.819931	0.872719	-0.03	0.928782	0.967445
COMPOUND_795_5073a11_178007	Rp	-	795.5073	11.18				(794.4978, 8944.86)(795.501, 4457.38)(796.5115, 4890.53)(797.5161, 2482.91)(798.5264, 5779.39)(799.5307, 2608.22)(1590.076, 635.84)(1591.0842, 487.9)	80095.53	46518.62	106081.9	64672.25	196299.7	40999.28	180370.9	49550.99	-0.75	0.001775	0.00444	-0.38	0.17216	0.487678
COMPOUND_795_5145a12_317778	Rp	+	795.5145	12.32				(796.5325, 4159.41)(797.0274, 2303.69)(813.5447, 4813.4)(813.5427, 5304.83)(814.0423, 4453.81)(796.5315, 4827.28)(797.5328, 4173.0)(798.5413, 5941.65)(398.7647, 26708.61)(399.2663, 12727.46)(399.7672, 3192.75)(400.2709, 737.25)	548457.1	160164.4	514264.9	204022.9	410084	194791.3	490148.9	228195.2	-0.40	0.174465	0.259507	0.61	0.086576	0.349408
COMPOUND_795_5423a10_858591	Rp	+	795.5423	10.86				(796.5501, 128940.63)(797.5533, 61800.51)(798.5641, 88244.2)(799.5675, 38826.4)(800.5709, 15196.42)(801.571, 5684.79)(802.5459, 9125.74)(803.5484, 4834.07)(804.547, 9929.72)(398.7789, 705.75)(399.2775, 566.08)(399.7872, 305.0)	2181420	760739.5	2081170	920135.5	1968901	1254353	1651152	1003305	-1.12	7.41E-07	3.84E-06	-0.12	0.627288	0.825147
COMPOUND_795_5536a12_791491	Rp	-	795.5536	12.79				(908.5246, 984.96)(909.5312, 720.57)(910.5441, 1164.76)(911.5476, 623.88)(794.5464, 39362.27)(795.5492, 19215.86)(796.5458, 16593.36)	955863.8	152239.8	1002605	172695.1	1062907	151442.1	1083022	163273.5	0.63	0.032974	0.061346	-0.15	0.663908	0.844813
COMPOUND_795_5779a12_466937	Rp	+	795.5779	12.47				(1592.1608, 50933.05)(1593.165, 42620.79)(1594.1628, 22950.71)(818.5746, 3543.62)(819.5681, 2490.85)(820.5856, 55220.88)(821.5885, 28226.56)(796.5859, 33885.52)(797.5884, 16677.62)(798.5748, 6382.16)(799.5841, 1023.82)(800.5916, 254.57)	1128274	499848.3	1021179	483175.7	1344021	790089.6	1377769	632939.4	-0.15	0.602271	0.695546	0.22	0.507816	0.75635
COMPOUND_795_5815a12_551982	Rp	+	795.5815	12.55	PC 17:0_20:4	1	40eV: 184.0716(100), 86.0936(12), 528.3431(4), 796.6245(1)	(1592.1593, 34662.71)(1593.1635, 27934.74)(1594.1615, 15906.12)(818.5978, 5723.86)(819.5939, 3512.7)(820.5865, 21135.05)(821.5881, 11010.72)(796.5858, 45292.1)(797.5882, 22245.62)(798.5851, 7655.5)(799.5809, 1750.87)(800.5854, 430.38)	536854.4	171551.5	502282.1	233632	439227.9	138084.5	465852.9	380476.1	-0.36	0.205772	0.296557	-0.07	0.843424	0.93246
COMPOUND_795_6143a13_479647	Rp	+	795.6143	13.48	PC 38:4e	2	10 eV: 796.6251(100), 184.0731(13), 485.4863(2)	(1592.2279, 150.73)(818.6033, 4100.63)(819.6056, 2827.19)(820.6193, 4858.17)(821.6217, 2602.03)(796.6226, 39196.94)(797.6267, 19901.48)(798.6298, 6416.77)(799.6285, 1926.51)	322808.2	175302.6	338666.8	176290.9	406869.9	161756.7	317820.3	170037.2	0.55	0.042592	0.076064	-0.83	0.009597	0.153413
COMPOUND_796_6438a0_7802489	Hilic	+	796.6438	0.78				(819.615, 1804.18)(820.6021, 3108.05)(821.6112, 2006.69)(814.6869, 33082.42)(815.6915, 11014.74)(816.694, 3029.43)(817.7004, 767.59)(797.6526, 5038.28)(798.6519, 3015.45)(799.6686, 10844.53)(800.6719, 5567.64)(801.6773, 2766.1)(802.68, 1318.17)(803.6953, 406.89)	187578.7	77013.42	209444.3	89841.91	150199.8	48038.1	165036.6	63383.86	-0.45	0.126582	0.199066	-0.08	0.817975	0.92157
COMPOUND_797_1547a10_968443	Rp	+	797.1547	10.97				(820.1549, 1930.41)(815.1624, 1282.48)(798.161, 10967.92)(799.1476, 4611.75)(800.1382, 1717.0)	35708.22	22856.57	32968.43	27542.14	31342.13	7831.216	35044.4	12187.92	-0.20	0.473851	0.577441	0.67	0.042856	0.268832
COMPOUND_797_5583a11_058923	Rp	+	797.5583	11.06	TAG 48:13 (M+NH4)+	2	10 eV: 798.5624(100), 184.0743(13), 284.7125(2), 86.0934(2)	(1596.1003, 2033.47)(1597.0692, 2141.68)(1598.0889, 1333.6)(1599.0883, 701.47)(820.551, 54265.33)(821.5548, 27040.72)(822.5652, 70366.56)(823.5685, 33928.26)(824.5805, 69078.09)(798.5656, 340116.22)(799.5693, 156448.47)(800.5719, 48892.57)(801.5746, 12683.64)	2464964	828903.5	2669035	932949.8	2181083	551748.4	2190499	918530.5	-1.20	5.29E-08	3.6E-07	-0.18	0.428599	0.707279
COMPOUND_797_5626a11_364023	Rp	+	797.5626	11.36				(1596.1204, 4137.89)(1597.1238, 3560.49)(1598.1324, 3741.3)(1599.1364, 2781.8)(820.5575, 80261.3)(821.5543, 37501.9)(822.5664, 250665.73)(823.5697, 122196.8)(824.5809, 199310.75)(798.5673, 313521.5)(799.5709, 145090.66)(800.5821, 240227.47)(801.586, 102502.74)(802.5981, 520492.16)(803.6023, 234110.88)(399.7761, 102.63)(400.2492, 708.37)	7546809	2825825	7894938	2620745	6155785	1088471	5988852	2029684	-0.92	7.54E-05	0.000238	-0.03	0.922268	0.965124
COMPOUND_797_5894a12_716313	Rp	+	797.5894	12.72				(798.6008, 33998.72)(799.603, 16569.27)(800.6019, 5548.45)(801.6075, 1377.28)	169090	39946.73	192780	57268.25	162950.9	47804.24	163198.1	47849.45	-0.54	0.041881	0.075047	0.26	0.401154	0.690408
COMPOUND_799_538a11_857125	Rp	-	799.5380	11.86				(912.5591, 3853.57)(798.5284, 78174.3)(799.5317, 36991.8)(800.5434, 54297.82)(801.5466, 26936.35)(1598.075, 511.5)(1599.0269, 550.36)	493057.2	204604.8	547414.9	217010.6	758409.5	260839.2	772064.8	274609.1	1.02	6.58E-05	0.000212	-0.12	0.654743	0.841269
COMPOUND_801_5517a12_060341	Rp	-	801.5517	12.06	PC 16:1_18:2	1	10 eV: 800.5442(100), 740.5197(30), 376.2128(1), 71.8725(1)	(800.5441, 445299.34)(801.5476, 210481.8)(802.5508, 59037.24)(803.554, 12597.76)(804.5604, 2996.0)	3249167	1042031	3685460	1217289	5234855	1229550	4960507	1452624	1.34	8.8E-07	4.42E-06	-0.51	0.082761	0.345146

									1st trimester				3rd trimester									
									Control		Pre-eclampsia		Control		Pre-eclampsia		Time			Interaction		
Compound	Column	Ion_mode	Mass	Retention_	Identification	ID_level	MSMS_spectra	CompositeSpectrum	Mean	SD	Mean	SD	Mean	SD	Mean	SD	Value	raw p	FDR_p	Value	raw p	FDR_p
COMPOUND_801_5916a11_457064	Rp	+	801.5916	11.46				(824.5819, 170840.44)(825.5848, 82887.73)(826.5969, 146394.58)(827.6007, 65906.66)(802.598, 753303.8)(803.6018, 350087.78)(804.6046, 90901.58)(805.6054, 18587.0)	5278350	1979765	5606918	2056242	3574470	2141549	3206084	1573134	-1.22	7.44E-09	6.33E-08	-0.26	0.23951	0.544276
COMPOUND_803_5349a12_255897	Rp	+	803.5349	12.26	PC 38:7	2	40 eV: 146.9805(100), 621.4828(65), 745.4794(10), 502.3124(13)	(815.5353, 2982.2)(816.0318, 1909.51)(816.5406, 1614.28)(817.0421, 1022.57)(824.0408, 1536.29)(824.5388, 1450.44)(825.04, 7113.81)(825.5423, 10130.99)(804.5527, 96227.03)(805.556, 46849.9)(402.7753, 3377.54)(403.2762, 2321.25)(403.7767, 1167.86)(404.2791, 388.28)(404.7722, 95.33)	821350	117834.1	766902.4	104254.6	755783	103578.6	741381.6	112072.9	-0.45	0.099495	0.162099	0.23	0.471616	0.734155
COMPOUND_803_5474a11_831466	Rp	+	803.5474	11.83	PC 18:2_20:5	1	40 eV: 184.0756(100), 124.9984(6), 437.1869(2), 804.5533(1)	(826.536, 5635.78)(827.5344, 3131.94)(828.5446, 1451.38)(829.5394, 404.81)(842.4857, 1865.27)(821.5569, 1658.28)(804.5547, 146578.02)(805.5578, 70205.84)(806.5616, 21833.16)(807.5636, 5120.09)	795199.9	466866.5	817518.7	396650.5	1078540	543982.9	1310827	636632.3	0.62	0.011003	0.023007	0.40	0.154078	0.466404
COMPOUND_803_5483a11_835721	Rp	+	803.5483	11.84				(826.5586, 15824.46)(827.5619, 8580.84)(828.5752, 4775.75)(829.5971, 1528.45)(821.5565, 1706.52)(842.4929, 2695.08)(804.5544, 59677.08)(805.5575, 28910.47)(806.569, 37453.32)(807.5717, 16671.41)(808.5769, 6801.45)	902590	348499.8	884833.5	311409.7	1237540	595049.1	1340124	562643.7	0.78	0.001133	0.002916	0.27	0.329625	0.635357
COMPOUND_803_5511a11_19134	Rp	+	803.5511	11.19				(804.5584, 96629.78)(805.5606, 48872.27)(806.5678, 50200.71)(402.7797, 1406.22)(403.2775, 683.9)	966300.6	280005.8	897852.8	359074.7	678911.5	566634.8	829092.5	597761.8	-0.75	0.001426	0.003613	-0.15	0.587047	0.799293
COMPOUND_803_5668a12_344101	Rp	-	803.5668	12.34	PC 16:0_18:2	2	10 eV: 802.5593(100), 279.2329(3)	(802.5591, 780535.0)(803.563, 359118.0)(804.566, 94938.71)(805.5687, 19080.78)(806.57, 3340.83)(807.5736, 635.99)	24769811	2528346	26919565	2457426	27450000	1935001	26901754	2889482	0.90	0.001017	0.002652	-0.92	0.00381	0.093266
COMPOUND_803_5991a10_217415	Rp	+	803.5991	10.22				(842.5788, 991.5)(843.0888, 470.54)(843.5889, 6115.16)(411.3178, 1922.39)(411.8226, 592.25)(412.3228, 525.76)(412.8252, 48.27)(804.6047, 3883.48)(805.6058, 1753.32)(806.5993, 819.81)(807.6027, 100.68)(422.3147, 981.82)(422.8076, 61.51)(423.3114, 496.47)(402.8037, 491.38)(403.2886, 1105.07)(403.7922, 103.5)	69427.86	24451.36	60721.52	22096.46	83807.92	19488.24	84528.89	22579.69	0.49	0.078178	0.130869	0.43	0.197401	0.508252
COMPOUND_803_6023a10_707655	Rp	-	803.6023	10.71				(802.595, 17728.34)(803.5977, 9312.33)(804.5983, 2535.52)(805.5965, 613.83)(806.5962, 152.94)	48935.32	27850.17	60386.44	34336.37	50822.56	23730.63	50145.73	25546.64	-0.08	0.767305	0.832356	-0.41	0.20429	0.516343
COMPOUND_805_5482a11_072999	Rp	-	805.5482	11.07				(804.539, 13815.38)(805.5421, 6410.18)(806.5261, 6497.0)(807.5258, 3034.17)(850.5426, 7000.87)(851.5453, 3578.36)(852.5397, 7279.53)(853.5419, 3727.33)	129805.3	42554.08	136832.8	46374.74	141980	46336.65	121089.3	26959.39	-1.11	2.96E-07	1.69E-06	-0.29	0.214916	0.521996
COMPOUND_805_5626a12_181954	Rp	+	805.5626	12.18	PC 16:0_22:6	1	10 eV: 806.5684(100), 184.074(12), 86.0942(1)	(828.5539, 56676.38)(829.5558, 29112.66)(830.5606, 11347.54)(831.5591, 3468.65)(823.6028, 3217.87)(824.6039, 1378.04)(844.5245, 5603.15)(845.525, 2978.73)(846.5365, 2503.22)(806.5709, 1273137.2)(807.5747, 633189.75)(808.5812, 222204.48)(809.5839, 60083.6)	27277358	6224074	24628261	6234658	28221429	4755916	29092982	5945672	0.19	0.494946	0.596053	0.56	0.103883	0.381877
COMPOUND_805_5658a12_035259	Rp	+	805.5658	12.04				(828.5505, 11121.51)(829.5481, 6356.61)(830.5699, 41800.48)(831.5736, 20772.05)(823.6224, 2083.41)(806.5712, 366537.38)(807.5745, 184610.11)(808.5768, 51209.79)(809.5759, 11049.31)(810.5735, 2542.01)(811.5935, 442.01)	3284945	1095565	3015891	951562.4	2996978	792800.4	3090031	680516.6	-0.23	0.413082	0.517387	0.39	0.243186	0.550407
COMPOUND_805_5784a9_587811	Rp	+	805.5784	9.59				(414.7896, 1535.67)(415.2841, 1353.72)(415.7934, 338.06)(412.3103, 15044.86)(412.8122, 6648.35)(413.3103, 2416.62)(413.8134, 604.41)(806.5843, 11449.46)(807.5867, 5259.83)(808.5888, 1538.19)(809.591, 472.4)(810.5772, 117.34)(423.2918, 352.51)(423.8, 133.8)(424.3029, 905.66)(403.7971, 5073.21)(404.2961, 3111.87)(404.8025, 755.37)	407003.1	108947.4	356083.2	68887.13	425602.6	69719.25	430639.9	110623.1	0.15	0.597071	0.691669	0.60	0.086036	0.348516
COMPOUND_805_5821a12_793038	Rp	-	805.5821	12.79	PC 34:1	2	20 eV: 744.5547(100), 804.5764(30), 281.2487(10), 44.9998(4), 480.309(1)	(804.5747, 840188.3)(805.5788, 393290.88)(806.5816, 99340.09)(807.5843, 19944.06)(808.5851, 3548.98)(809.5846, 613.74)	16481132	2661120	17186957	3174664	19335714	2430767	21200000	2808533	0.86	0.000198	0.000591	0.26	0.305317	0.613089
COMPOUND_806_6259a0_7969206	Hilic	+	806.6259	0.80				(829.6211, 3373.51)(830.6236, 1784.76)(831.6365, 7460.25)(832.6376, 4449.45)(833.6501, 8206.34)(824.6295, 3779.95)(807.6357, 21292.9)(808.6363, 10831.37)(809.652, 24555.1)(810.6522, 13467.68)(811.6689, 87503.69)(404.3232, 1372.97)(404.824, 687.28)(405.3318, 1301.27)(405.8309, 537.69)(406.3341, 255.31)	412257.3	130539.5	446832.3	136913	465967	211999.2	422644.3	174983.7	0.30	0.291571	0.390827	-0.23	0.496013	0.751527
COMPOUND_807_5611a10_627024	Rp	+	807.5611	10.63				(830.556, 122831.57)(831.5588, 56107.81)(832.5699, 82065.22)(833.5735, 34886.83)(808.56, 3324.11)(809.5596, 1509.14)(810.5408, 2308.17)(811.5405, 1333.38)	521163.6	337188.5	501894.7	258146.1	301976.6	261383.5	317317	216371.9	-1.03	6.25E-06	2.6E-05	-0.10	0.680276	0.853001
COMPOUND_807_5798a12_375857	Rp	+	807.5798	12.38	PC 38:5	2	10 eV: 808.5857(100),184.0758(10)	(830.5668, 25291.13)(831.5705, 13168.28)(832.5835, 23087.27)(833.5878, 11313.86)(834.6001, 31714.34)(846.5265, 3583.93)(847.5254, 2225.38)(808.5871, 716585.3)(809.591, 352037.56)(810.5935, 94814.47)(811.5906, 20258.47)(812.5776, 4126.2)(813.599, 460.64)	6769973	1965862	5730694	1658790	5557741	893049	6227971	1463985	-0.52	0.048664	0.085485	0.92	0.00323	0.081732
COMPOUND_807_5818a12_500268	Rp	+	807.5818	12.50				(830.5674, 17935.56)(831.5707, 9215.15)(832.584, 15292.02)(833.5885, 7831.64)(846.5336, 4507.27)(808.5864, 713420.8)(809.5903, 345694.22)(810.5944, 106151.98)(811.5918, 27011.77)	4441667	1800302	3786734	1489509	3712439	1005470	4362628	1439769	-0.38	0.149179	0.226993	0.80	0.011414	0.166978
COMPOUND_808_6454a0_7934525	Hilic	+	808.6454	0.79				(831.6365, 6177.56)(832.6373, 3743.83)(833.6512, 9585.27)(834.6484, 4765.35)(835.6631, 5006.98)(826.6506, 6853.56)(809.6524, 22511.88)(810.6537, 10253.37)(811.6691, 65469.05)(812.6721, 34287.64)(813.6833, 37830.06)(405.3311, 1277.32)(405.8311, 656.67)(406.3371, 317.04)	617268.5	225006	666514.5	266007.2	516080	178635.2	553829.4	243455.1	-0.23	0.426942	0.531495	-0.14	0.678524	0.851748
COMPOUND_809_4733a11_166188	Rp	+	809.4733	11.17				(837.947, 282.03)(838.4205, 526.45)(405.743, 15297.81)(406.2452, 8188.39)	108300.6	27441.99	109666.4	29088.43	104847.5	40648.4	111796.9	36544.84	-0.99	1.66E-05	6.35E-05	-0.20	0.426592	0.705867
COMPOUND_809_5331a11_179161	Rp	-	809.5331	11.18				(808.5259, 20224.93)(809.5297, 8990.91)(810.5245, 9096.98)(811.5265, 3528.03)(812.525, 1616.08)(854.5216, 7488.35)	213578.2	77130.4	239064.8	90794.24	241186.7	11592.74	253655.1	74357.69	-0.94	2.23E-05	8.18E-05	-0.31	0.209232	0.518933
COMPOUND_809_5559a12_19905	Rp	-	809.5559	12.20				(844.5322, 17790.44)(845.5352, 7915.89)(846.5477, 3231.56)(847.5721, 835.7)(808.5481, 6901.29)(809.5514, 3593.02)(810.5553, 1455.96)(811.5661, 450.11)	138575.3	26295.49	139314.3	32146.77	134976.3	32574.42	156108.1	35423.94	-0.13	0.649722	0.737859	0.62	0.083666	0.346484
COMPOUND_809_5951a12_8862915	Rp	+	809.5951	12.89	PC 18:0_20:4	1	10 eV: 810.6015(100), 184.0735(8)	(832.583, 44462.23)(833.5867, 22232.24)(834.6018, 92460.52)(835.605, 45325.59)(836.6153, 58411.31)(848.5538, 4700.25)(849.5531, 2845.73)(850.5569, 3288.22)(827.6411, 417.93)(810.6011, 781902.7)(811.6051, 386191.47)(812.6081, 103516.84)(813.5953, 25177.91)(814.6077, 3148.33)(815.6131, 438.62)	12568897	3512020	11415812	3793958	10396884	2662643	10606833	3324973	-0.53	0.038551	0.07007	0.28	0.33564	0.639647
COMPOUND_809_5959a12_640725	Rp	+	809.5959	12.64	PC 18:1_20:3	1	10 eV: 810.6038(100), 184.0716(8), 522.3501(1)	(832.5841, 23141.67)(833.5866, 12320.6)(834.6012, 34660.84)(835.6052, 17610.52)(836.605, 5616.98)(827.6414, 357.3)(810.6016, 486332.6)(811.6056, 235236.88)(812.6079, 63556.43)(813.6094, 12829.68)	2878208	1043306	2851766	1107611	3634284	1100444	4223037	1484344	0.48	0.047555	0.083973	0.53	0.063348	0.307286
COMPOUND_810_6609a13_636442	Rp	+	810.6609	13.64	SM d42:3	2	10 eV: 811.6664(100), 184.0711(15), 458.2399(1)	(833.6336, 10426.67)(834.6229, 3011.95)(835.6118, 1100.79)(811.6694, 58748.79)(812.6709, 31085.18)(813.6724, 8606.25)(814.6658, 1240.29)(815.6755, 111.8)(406.3459, 389.2)(406.8439, 79.7)(407.3502, 161.99)	1084407	461677.4	1205156	494301.6	757650	276685.4	786873.8	352280.1	-0.46	0.071006	0.12032	0.35	0.249795	0.559739
COMPOUND_810_6614a0_78531355	Hilic	+	810.6614	0.79				(833.6514, 9140.24)(834.6518, 4764.4)(835.6644, 5858.0)(836.6671, 2410.59)(837.6753, 1146.13)(828.6692, 3957.46)(829.6727, 3163.1)(811.6695, 138088.38)(812.6724, 72649.15)(813.6834, 76592.25)(814.6875, 35158.29)(815.6926, 12063.62)(816.6967, 3150.09)(817.7009, 947.1)(406.3364, 273.64)(406.8383, 150.21)(407.3373, 367.21)	673805	272801	721278.3	299062.2	513734.9	173837.8	544408.1	231921.4	-0.65	0.021223	0.04156	-0.02	0.955593	0.980661
COMPOUND_811_4871a12_33389	Rp	+	811.4871	12.33				(406.7499, 14004.93)(407.2517, 6565.37)(407.7513, 3474.75)(408.2522, 1189.05)	183094.9	17362.45	178726.6	20148.11	193603.7	20922.78	186780.9	17117.81	0.62	0.039095	0.070831	-0.28	0.423883	0.704491
COMPOUND_811_5064a11_165831	Rp	+	811.5064	11.17				(850.4639, 1810.39)(851.4779, 1238.64)(406.7608, 10883.28)(407.2623, 5427.27)(407.7639, 2020.9)(408.2559, 654.34)	100664.2	24012.02	113347.7	26944.62	101167.8	10211.1	95066.25	44506.07	-0.94	3.12E-05	0.00011	-0.45	0.075407	0.329961
COMPOUND_811_5725a12_527865	Rp	-	811.5725	12.53	PC 36:5e	2	20 eV: 750.5433(100), 810.5645(28), 303.2329(12), 464.317(3), 44.9993(2)	(924.5391, 1338.49)(925.5575, 552.05)(926.521, 983.19)(927.5254, 448.53)(810.5645, 38032.39)(811.5677, 18587.97)(812.5777, 20035.86)(813.5793, 8861.9)(814.5727, 3406.23)	746552.9	159350.9	749521.8	191847.8	630618	179226.6	655929.5	149799.3	-0.17	0.519013	0.619167	-0.02	0.952272	0.980085

									1st trimester				3rd trimester									
									Control		Pre-eclampsia		Control		Pre-eclampsia		Time			Interaction		
Compound	Column	Ion_mode	Mass	Retention_	Identification	ID_level	MSMS_spectra	CompositeSpectrum	Mean	SD	Mean	SD	Mean	SD	Mean	SD	Value	raw p	FDR_p	Value	raw p	FDR_p
COMPOUND_811_6117a13_244986	Rp	+	811.6117	13.24	PC 18:0_20:3	1	10eV: 812.6133(100), 184.0732(16), 310.348(1)	(834.5997, 38355.43)(835.6031, 19358.69)(836.6169, 56407.12)(837.6208, 27587.21)(838.6246, 9948.93)(839.6076, 3625.89)(840.6336, 480.42)(850.5748, 3131.69)(851.5743, 2371.38)(852.573, 1649.26)(829.6169, 338.65)(830.6366, 866.58)(831.6556, 179.65)(812.6186, 317822.75)(813.622, 157377.39)(814.6243, 41904.05)(815.6265, 8807.96)(816.6141, 1936.42)(817.633, 166.37)	4199728	2273510	4067581	1871355	3872576	1645490	4860263	2431549	-0.20	0.45436	0.560053	0.52	0.098803	0.368908
COMPOUND_813_5493a12_0586405	Rp	-	813.5493	12.06				(926.5021, 1129.49)(927.4941, 919.07)(812.5438, 29748.08)(813.5469, 15489.9)(814.52, 17415.14)(858.5067, 4195.9)	201296.4	50363.78	178954.5	69867.52	202147.9	47379.6	191381.4	52266.29	0.11	0.696358	0.778262	0.17	0.604357	0.810123
COMPOUND_813_5508a9_927079	Rp	-	813.5508	9.93				(926.5213, 683.92)(927.5299, 391.33)(928.5045, 606.87)(929.5197, 217.51)(930.5317, 325.28)(812.5444, 79233.59)(813.5474, 39179.5)(814.5524, 12646.22)(815.5566, 2953.84)(816.5613, 746.99)	65275.3	34110.95	85342.85	60840.48	76004.75	45014.55	74945.2	44159.58	-0.99	4.59E-06	1.96E-05	-0.38	0.106967	0.38909
COMPOUND_813_5561a11_23919	Rp	+	813.5561	11.24				(836.5419, 42397.38)(837.5442, 21234.7)(838.5602, 141386.94)(839.563, 66532.3)(814.5623, 214146.45)(815.5649, 101460.88)(816.5767, 274427.0)(817.5801, 123498.23)(818.5893, 107215.16)	4281641	1786020	4332267	2107547	4342261	4167380	3416063	1970627	-0.71	0.002145	0.005297	-0.25	0.346337	0.651869
COMPOUND_813_5874a12_718801	Rp	-	813.5874	12.72	PC 36:4e	2	20 eV: 752.5582(100), 812.5812(40), 303.2318(13), 44.9986(4)	(926.5322, 1887.85)(812.5807, 149352.84)(813.5837, 72358.63)(814.5865, 20523.76)(815.5883, 4021.67)(858.5863, 993.02)(859.5654, 501.38)(860.583, 171.9)	1052377	264331	1048928	314290.1	909985.3	168979.8	877705.2	192954.6	-0.52	0.037311	0.068092	-0.08	0.786519	0.909997
COMPOUND_814_542a11_783751	Rp	-	814.5420	11.78				(813.537, 25170.07)(814.5382, 12081.66)(815.5392, 3609.64)(859.5299, 3678.16)	250388.6	65313.07	259889.3	103245.6	280105.4	74532.05	275525.3	68199.84	0.75	0.002836	0.006801	-0.18	0.523679	0.766338
COMPOUND_814_5568a10_857167	Rp	+	814.5568	10.86				(837.5473, 94675.16)(815.5645, 130106.38)(816.5719, 66325.13)(817.576, 22662.44)	1791534	477262	1706405	594200	1969205	389498.6	1355709	655616.4	-1.03	5.75E-06	2.41E-05	-0.17	0.490692	0.748114
COMPOUND_815_4156a7_49421	Rp	+	815.4156	7.49				(838.4176, 112.87)(838.9306, 62.72)(839.421, 160.63)(835.4034, 230.27)(835.8987, 26.44)(427.6758, 358.75)(428.1633, 237.1)(419.7085, 5831.51)(420.2099, 2484.57)(420.709, 791.82)(421.2026, 265.21)(417.2301, 40351.89)(417.7317, 17450.98)(418.2338, 5109.34)(418.7349, 1295.71)(419.2359, 274.08)(816.4225, 23440.42)(817.4271, 9230.44)(818.4283, 2962.88)(819.431, 795.04)(820.4374, 206.73)(408.7175, 1695.51)(409.2123, 1087.87)(409.72, 262.73)	289591.5	125220.8	270890.5	97462.96	255968.8	118485.2	299302.3	115858.6	-0.20	0.504384	0.604832	0.55	0.12102	0.411311
COMPOUND_815_5666a12_244778	Rp	-	815.5666	12.24				(814.5588, 15490.13)(815.5599, 10589.22)(860.5915, 1508.76)(861.5954, 896.67)(862.5768, 1044.12)	107093.8	38129.25	111719.9	42090.81	124663.5	42860.77	109010.4	31887.83	0.40	0.144327	0.221256	-0.24	0.464171	0.732257
COMPOUND_815_5766a12_533583	Rp	+	815.5766	12.53				(838.5596, 1963.58)(839.5572, 1872.79)(840.5481, 2119.79)(841.5447, 2003.56)(842.531, 3681.57)(816.5882, 28038.14)(817.592, 13697.78)(818.5916, 13868.91)(819.5907, 6473.85)(820.5858, 16382.99)	201979.9	57854.22	183344	73771.47	199218.5	78966.62	216752	65373.75	-0.02	0.933611	0.95693	0.19	0.536122	0.776357
COMPOUND_815_6011a12_916941	Rp	-	815.6011	12.92				(814.5953, 19225.41)(815.5991, 9562.5)(816.5921, 3234.32)(817.5863, 1102.95)	240846.9	100210.6	253804.9	101287.4	252160.4	63082.25	256493.6	98175.52	0.36	0.168105	0.251544	-0.32	0.297896	0.60748
COMPOUND_817_5a11_98166	Rp	+	817.5000	11.98				(827.0369, 1131.78)(827.5355, 3311.09)(828.0305, 601.92)(838.027, 2084.45)(838.5096, 1194.98)(839.0194, 1469.36)(835.522, 2093.5)(437.2387, 211.84)(437.7353, 177.19)(835.5069, 1566.04)(836.0247, 1626.14)(836.5172, 2078.51)(837.0143, 1920.15)(837.5073, 2051.38)(838.0088, 1692.72)(838.5049, 1864.7)(839.0057, 1451.77)(839.5024, 1169.25)(818.511, 9028.8)(819.5124, 5160.78)(820.5258, 4573.91)(409.756, 13012.07)(410.2579, 6285.33)(410.7589, 1890.51)(411.245, 218.83)	274114	105007.1	238393	71194.81	322030.7	180285.3	362388.5	201183.8	-0.01	0.960528	0.976573	0.45	0.188222	0.504013
COMPOUND_817_5442a11_094098	Rp	-	817.5442	11.09				(930.5168, 2607.76)(931.5158, 1396.68)(816.5387, 72192.52)(817.5419, 32912.48)(818.5542, 119539.96)(819.5576, 54013.21)(820.5464, 28124.41)(821.5433, 10524.25)	860761.3	271580.6	956846.2	338345.9	1104397	498964.8	1033231	718424.4	-0.83	0.000618	0.00169	-0.33	0.235144	0.541455
COMPOUND_817_5456a11_323749	Rp	-	817.5456	11.32				(930.544, 18454.35)(931.5453, 8938.16)(932.5351, 4415.7)(816.5393, 199318.89)(817.5424, 91943.16)(818.5536, 154064.05)(819.5573, 66263.33)(820.563, 22473.28)	609302.9	253434.1	706220.8	226577.9	437550.5	25808.69	425996	152393.9	-1.16	4.37E-08	3.04E-07	-0.18	0.430792	0.708135
COMPOUND_817_5811a11_319503	Rp	+	817.5811	11.32				(840.5747, 122685.97)(841.5787, 53043.07)(818.593, 576618.56)(819.5968, 275583.1)	1755671	1018789	1691806	1095638	1424605	1396018	1103060	439309.1	-1.23	7.64E-09	6.47E-08	-0.13	0.548464	0.77853
COMPOUND_817_5818a12_677716	Rp	-	817.5818	12.68				(930.5763, 1662.33)(931.5666, 1040.81)(816.5751, 81552.77)(817.5785, 38582.41)(818.5607, 17672.8)(862.5948, 15315.4)(863.5985, 8251.9)	967133.5	262128.9	1030982	358333.5	920517.4	126885	824789.2	163198.2	0.27	0.238705	0.333581	-0.06	0.832861	0.926681
COMPOUND_817_5841a11_419728	Rp	+	817.5841	11.42				(840.5753, 87328.65)(841.578, 43904.13)(842.5909, 89357.38)(843.5961, 41477.06)(844.59, 33865.07)(856.5588, 9741.06)(857.5605, 5257.42)(858.572, 6545.43)(818.5919, 755353.94)(819.5957, 358122.25)(820.5946, 103918.57)(821.5897, 22160.71)	4362272	2389116	4704741	2337041	2100707	1575192	1403329	1123330	-1.28	4.01E-10	5.01E-09	-0.22	0.283092	0.591944
COMPOUND_817_5923a13_075347	Rp	+	817.5923	13.08				(840.5816, 1568.96)(841.5754, 1459.82)(842.5683, 2072.84)(818.6043, 14823.06)(819.6081, 7957.08)(820.6199, 11992.83)(821.6232, 6012.43)(822.6184, 2472.6)	127646.8	75573.08	155878.7	123501.3	117600.4	62409.39	116982.2	68722.24	0.36	0.208391	0.299487	-0.30	0.376378	0.678626
COMPOUND_817_598a12_667346	Rp	+	817.5980	12.67	PC 40:7e	2	10 eV: 818.6086(100), 184.0724(10), 502.3357(1)	(840.5851, 7936.13)(841.5869, 4265.08)(842.5741, 2009.46)(843.5812, 676.51)(818.608, 122063.43)(819.6108, 64435.84)(820.6123, 19112.28)(821.6045, 4302.22)	652747.7	226878	575437.3	209045.8	783495.5	235278.3	760750.8	242919.8	0.61	0.025702	0.049092	0.18	0.573735	0.790246
COMPOUND_819_5134a12_24219	Rp	+	819.5134	12.24				(427.7837, 357.27)(428.2786, 309.7)(839.5329, 1931.28)(840.0298, 1625.34)(840.5294, 1250.59)(841.0281, 704.03)(831.4783, 171.35)(831.9649, 53.2)(842.5225, 2863.64)(843.5278, 2600.92)(844.5256, 4563.04)(845.5268, 2124.48)(846.5354, 1851.63)(820.5281, 6279.9)(821.0386, 1596.34)(821.5325, 9222.66)(822.0345, 6317.85)(822.5374, 6259.41)(848.0355, 929.94)(848.5354, 1117.34)(840.0343, 1094.44)(840.524, 1403.19)(841.0284, 793.53)(837.5374, 4914.91)(410.7641, 24008.21)(411.266, 11745.35)(411.7668, 3287.74)(412.2652, 1053.48)	307153.6	59896.03	269892.2	68384.5	278439.6	34265.54	279469.7	62348.34	-0.04	0.871118	0.91287	0.30	0.358907	0.666906
COMPOUND_819_5438a10_837796	Rp	+	819.5438	10.84				(440.7478, 327.78)(441.2422, 2328.19)(441.7387, 1271.03)(421.7419, 554.38)(422.2438, 429.09)(422.7528, 1048.16)(820.5502, 131462.34)(821.5535, 65467.13)(822.565, 77319.15)(823.569, 33049.17)(824.575, 27575.78)(410.78, 1395.63)(411.2857, 885.68)(411.7856, 653.44)	1410195	518734.9	1246255	504508.3	1293648	784437	1244569	693224.8	-1.11	1.7E-06	8.12E-06	-0.02	0.938657	0.97184
COMPOUND_819_5542a12_535168	Rp	-	819.5542	12.54				(818.5472, 44526.77)(819.5499, 22296.55)(820.5464, 18957.68)	1150659	112495.5	1201137	96691.82	1239264	91913.75	1239531	104818.1	0.70	0.007909	0.017193	-0.37	0.224636	0.532591
COMPOUND_819_56a11_121188	Rp	-	819.5600	11.12				(818.5548, 342638.66)(819.5584, 155717.14)(820.5609, 42408.7)(821.5629, 8690.7)(864.5445, 1347.98)(865.5467, 792.57)	497549.6	191774	622527	552027	202564.1	208299.5	211946.2	225662	-1.20	6.28E-07	3.3E-06	-0.22	0.396073	0.690408
COMPOUND_819_5614a11_190397	Rp	-	819.5614	11.19	PC 16:0_HODE	1	10 eV: 818.553(100), 758.5311(30), 295.2285(2)	(818.5541, 486499.88)(819.5581, 223002.61)(820.5607, 60852.2)(821.5632, 12273.88)(822.5547, 2661.39)(823.5613, 415.56)(864.5429, 3760.12)(865.5448, 2054.21)	5713077	1147957	6229103	1319915	1820508	2795350	1221215	2486766	-1.07	1.71E-05	6.5E-05	-0.38	0.172652	0.487735
COMPOUND_819_576a12_380157	Rp	+	819.5760	12.38	duplicate of PC39:6			(837.5778, 2707.34)(838.5562, 1342.73)(820.585, 20964.87)(821.5884, 11389.41)(822.577, 3854.71)(823.5794, 559.05)(824.5749, 113.94)	169275	91198.34	152177.7	50896.43	134631.3	59745.57	138665.7	72474.41	-0.05	0.854374	0.89993	0.19	0.56113	0.783425
COMPOUND_819_5786a12_398953	Rp	+	819.5786	12.40	PC 39:6	2	20 eV: 820.5784(100), 184.0733(80), 504.2071(3)	(837.6058, 3373.15)(838.6215, 460.19)(820.5871, 27382.19)(821.59, 14504.56)(822.5843, 4407.46)(823.5811, 864.16)	171329.5	109568.7	141823.2	81773.93	115060.4	46807.93	133020.9	89624.22	-0.55	0.039457	0.071314	0.46	0.139873	0.444883
COMPOUND_819_5932a9_808102	Rp	+	819.5932	9.81				(421.7983, 1292.42)(422.3047, 1260.47)(422.7952, 263.07)(419.3181, 18867.05)(419.8197, 8572.4)(420.3231, 2373.12)(420.8243, 681.05)(820.6003, 20186.13)(821.6027, 9210.48)(822.6003, 2975.88)(823.5992, 870.38)(824.611, 223.92)(410.8057, 4238.6)(411.3069, 1580.25)(411.8081, 642.32)(412.2984, 192.26)(412.7881, 24.26)	476614.6	126799	408738.8	85864.68	513634	90982.04	514902.5	133473.4	0.23	0.40543	0.509502	0.64	0.060866	0.302474
COMPOUND_819_6108a12_898511	Rp	+	819.6108	12.90	PC 40:6e	2	10 eV: 820.6216(100), 184.0707(13), 60.0798(5)	(858.5969, 10471.71)(859.599, 5692.12)(820.6195, 27517.85)(821.6217, 14588.72)(822.6161, 5923.47)(823.6018, 2012.44)	368803.9	109787	353589.7	83778.08	349120.2	63447.4	345760.9	122503.2	0.16	0.583247	0.679416	-0.10	0.775211	0.905077
COMPOUND_820_155a9_752432	Rp	-	820.1550	9.75				(933.1595, 208.0)(934.166, 181.02)(935.1376, 243.37)(855.1305, 297.43)(856.1136, 231.2)(857.1388, 73.86)(819.1502, 8200.0)(820.1533, 2378.19)(821.1498, 2947.42)(822.1515, 905.69)(823.1544, 384.53)(865.1408, 660.66)(866.1482, 376.25)(867.1256, 288.38)	108007.9	37832.82	98503.89	28107.88	95245.71	27361.9	96352.91	37776.74	-0.33	0.269354	0.367182	0.10	0.784995	0.909997

									1st trimester				3rd trimester									
									Control		Pre-eclampsia		Control		Pre-eclampsia		Time			Interaction		
Compound	Column	Ion_mode	Mass	Retention_	Identification	ID_level	MSMS_spectra	CompositeSpectrum	Mean	SD	Mean	SD	Mean	SD	Mean	SD	Value	raw p	FDR_p	Value	raw p	FDR_p
COMPOUND_820_1584a9_309281	Rp	-	820.1584	9.31				(819.15, 7657.01)(820.1528, 2254.58)(821.1484, 2557.89)(822.1491, 823.56)(823.164, 325.07)(865.1605, 527.33)(866.1573, 225.62)(867.1379, 371.69)	62871.87	11228.62	63520.26	9549.255	60350.57	12383.98	60667.67	12061.02	-0.25	0.400789	0.504797	-0.04	0.91513	0.961085
COMPOUND_821_5294a12_543394	Rp	+	821.5294	12.54				(844.5394, 4440.38)(845.5406, 2220.07)(846.5408, 2351.7)(847.5402, 1426.23)(848.5477, 2464.96)(842.0408, 1031.99)(842.5245, 5029.74)(843.0363, 923.38)(430.7552, 447.54)(431.2611, 323.94)(431.7592, 1495.58)(839.5497, 2191.71)(840.0458, 1317.38)(840.5364, 2110.2)(841.0372, 2085.1)(841.5354, 2028.2)(842.0376, 1279.02)(842.5279, 5315.06)(843.0344, 1246.6)(843.5298, 3265.34)(822.5725, 12264.78)(823.5969, 2568.53)(824.5559, 4402.94)(825.5563, 2270.38)(411.7714, 7715.57)(412.2717, 4039.48)(412.7791, 8196.72)(413.2809, 3790.25)(413.7701, 343.19)	750656.7	159014.7	679612.6	119280.1	808118.7	282751.5	897225.9	333028.4	0.59	0.022539	0.043718	-0.25	0.398797	0.690408
COMPOUND_821_5692a13_040231	Rp	-	821.5692	13.04	PC 18:0_18:2	1	10 eV: 820.5637(100), 417.1003(5)	(820.5624, 35251.39)(821.5654, 17312.54)(822.5618, 14973.74)	1269711	192797.5	1386188	202349.3	1336690	136252.5	1282157	202150.6	0.18	0.512159	0.612524	-0.72	0.028912	0.231966
COMPOUND_822_5555a12_53386	Rp	-	822.5555	12.53				(857.5178, 4967.62)(821.5492, 10338.53)(822.5533, 3570.35)(823.5603, 1200.38)	114688.6	24000.92	108972.9	21582.47	106437.9	23138.39	110779.3	20829.33	-0.41	0.138175	0.213422	0.47	0.147713	0.456493
COMPOUND_825_4188a0_89871585	Hilic	+	825.4188	0.90				(848.4222, 419.48)(849.4166, 318.5)(850.4338, 675.19)(843.4623, 443.3)(826.4261, 3836.89)(827.4297, 1965.65)(828.4369, 8579.11)(829.44, 4086.98)(830.4378, 5262.95)(831.4406, 2051.55)(832.4374, 1400.55)(833.4368, 463.36)	63475.85	18972.29	64877.73	20669.54	100021.5	20665.55	108449.5	22771.76	0.94	9.73E-05	0.000301	0.50	0.065663	0.309933
COMPOUND_825_5297a11_150297	Rp	-	825.5297	11.15				(824.5211, 16338.22)(825.524, 7392.56)(826.5218, 7675.22)(827.5253, 3176.62)(828.537, 2884.79)	209193.6	48752.43	211711.1	56470.52	180064.5	7149.557	156932.9	43793.38	-1.22	1.61E-08	1.26E-07	-0.14	0.543395	0.776977
COMPOUND_825_5529a12_011763	Rp	-	825.5529	12.01	PC 16:1_20:4	1	20 eV: 764.5209(100), 824.5395(37), 44.9981(11), 303.2318(5)	(824.5441, 228025.05)(825.5474, 106813.27)(826.5574, 107511.66)(827.5617, 44390.11)(828.5659, 13004.87)(829.569, 3202.28)	6365642	2265955	6134436	2361568	6361894	1941607	6275328	2560039	0.13	0.613084	0.706223	0.25	0.403119	0.691103
COMPOUND_826_1739a10_981858	Rp	+	826.1739	10.98				(849.1773, 2139.84)(844.1776, 1193.58)(827.1835, 7132.12)(828.1696, 3377.31)(829.1597, 1584.6)	35776.84	28219.45	38753.82	31975.68	30061.33	10425.52	29702.97	11142.67	0.20	0.4651	0.569838	-0.03	0.937771	0.97184
COMPOUND_827_5359a12_180136	Rp	+	827.5359	12.18	PC 40:9	2	40 eV: 645.4863(100), 146.9819(60), 478.34(3)	(839.528, 3096.71)(840.0272, 2029.85)(840.5214, 2397.47)(858.4855, 3103.39)(859.0066, 792.81)(855.9923, 368.03)(856.4876, 5352.73)(857.0101, 883.0)(848.0447, 1358.4)(848.538, 1450.94)(849.0408, 7676.29)(828.553, 112791.29)(829.5557, 56788.36)(830.5596, 17082.13)(831.5609, 4747.28)(414.7762, 3397.04)(415.2772, 2404.72)(415.7748, 1253.51)(416.2737, 358.89)(416.7612, 86.22)	973030.3	115258.2	936226.8	138293.1	885714.8	118260.1	847250.2	128913.8	-0.62	0.02436	0.046848	-0.01	0.962474	0.981922
COMPOUND_827_5665a12_278189	Rp	-	827.5665	12.28	duplicate of PC36:4			(826.5591, 886736.75)(827.563, 427021.16)(828.5662, 114750.37)(829.5687, 23190.27)(830.5722, 4432.67)(831.5744, 874.55)	11003604	1677448	10754653	1788782	10622571	1079152	11052675	1649305	0.09	0.691125	0.774808	0.14	0.587038	0.799293
COMPOUND_827_5673a12_128742	Rp	-	827.5673	12.13	PC 18:2/18:2	2	10 eV: 826.5593(100), 279.236(1)	(826.5597, 391094.9)(827.5633, 183349.25)(828.5659, 50689.39)(829.5689, 10737.18)(830.5729, 2492.89)(831.584, 463.74)	3387273	874955.2	3729767	1033170	3553017	1001525	3541768	1062017	-0.01	0.971751	0.983985	-0.19	0.537863	0.776408
COMPOUND_827_6023a10_685225	Rp	-	827.6023	10.69				(826.5961, 78262.14)(827.599, 38688.81)(828.602, 11490.13)(829.6045, 2499.39)	114554.1	50065.7	151294.4	68286.27	79565.36	30959.67	86289.19	42752.25	-0.72	0.002672	0.006456	-0.41	0.131323	0.428608
COMPOUND_827_6033a11_5870495	Rp	+	827.6033	11.59				(850.5962, 57091.14)(851.5994, 29618.08)(852.6096, 27148.98)(828.612, 87676.74)(829.6155, 38502.21)(830.6187, 13892.97)(831.6204, 3662.94)	1683177	824683.1	1525578	857699.2	1243959	467101.8	1277836	1065185	-1.21	7.52E-08	4.88E-07	-0.03	0.907757	0.958319
COMPOUND_829_5622a11_951857	Rp	+	829.5622	11.95				(852.5488, 4795.12)(853.5515, 2601.85)(854.5703, 39661.95)(830.5711, 24834.12)(831.5748, 12920.52)(832.5783, 4242.5)(833.5774, 1301.65)(834.5717, 226.13)	520665.2	180127.9	494881.7	188520.8	744727.2	241016.4	834904.4	244149.5	0.90	0.000345	0.000984	0.44	0.126671	0.423869
COMPOUND_829_5631a12_53796	Rp	+	829.5631	12.54	PC 40:8	2	40 eV: 647.5054(100), 146.9854(43), 771.4955(17), 830.6186(6)	(841.5355, 2541.04)(842.0388, 1672.94)(852.5567, 5174.45)(853.5575, 3555.94)(854.5613, 4625.28)(855.5523, 2253.73)(849.5453, 2112.89)(850.0485, 1285.4)(434.7595, 32.8)(435.2668, 1033.01)(435.7666, 759.07)(847.581, 2641.39)(848.6032, 604.42)(830.5675, 21243.01)(831.5706, 10988.22)(832.5838, 14979.0)(833.5885, 7575.52)(415.7756, 1621.82)(416.2755, 870.01)(416.7848, 719.0)(417.2713, 28.08)	322027.6	128058.8	292117.4	90479.58	297221.6	77644.97	325266.8	97811.02	0.02	0.950509	0.968573	0.29	0.3944	0.690408
COMPOUND_829_5822a12_538663	Rp	-	829.5822	12.54				(828.5749, 853537.56)(829.579, 416709.06)(830.5817, 111795.34)(831.5839, 21996.28)(832.5845, 3990.32)(833.5776, 792.08)	15407105	3996384	17279191	3263828	19300000	3421426	20042105	3447771	0.97	0.000239	0.000701	-0.25	0.388231	0.68827
COMPOUND_830_1854a9_308799	Rp	-	830.1854	9.31				(943.1742, 111.83)(944.1327, 285.12)(829.1786, 20829.03)(830.182, 6291.21)(831.1829, 1033.18)(832.1745, 204.08)(875.1704, 298.34)(876.159, 229.59)(877.1588, 648.69)	145329.1	28887.2	142790.1	20337.74	135608.9	14855.31	136806.4	22855.75	-0.39	0.197615	0.287116	0.12	0.738661	0.888668
COMPOUND_830_1855a9_74798	Rp	-	830.1855	9.75				(829.1787, 27599.75)(830.1819, 8060.73)(831.1789, 1309.05)(832.1806, 290.88)	277607.6	65251.02	269822.6	51626.78	272132.6	41356.14	279897	63541.01	-0.09	0.773758	0.833547	0.19	0.598001	0.805251
COMPOUND_830_186a9_494234	Rp	-	830.1860	9.49				(943.1413, 786.5)(944.1429, 359.85)(945.1511, 467.51)(829.1789, 34899.66)(830.182, 10042.59)(831.1733, 1728.23)(832.1626, 383.5)	175626.1	25240.25	169595.7	26195.45	173318.5	17257.92	170995.4	23297.01	-0.16	0.605187	0.698229	0.19	0.606565	0.810674
COMPOUND_830_1861a9_581605	Rp	-	830.1861	9.58				(865.1352, 1044.45)(866.1351, 563.07)(829.179, 70581.77)(830.1822, 19783.88)(831.179, 3276.78)(832.1837, 466.67)(875.1707, 478.46)(876.1715, 204.58)(877.1591, 688.18)	341946.6	39617.31	357142	31415.6	345033.7	43078.53	342159	37158.2	0.10	0.747872	0.816784	-0.48	0.188121	0.504013
COMPOUND_831_504a12_204472	Rp	-	831.5040	12.20				(830.4943, 7486.29)(831.4993, 3468.98)(832.5014, 1062.97)(876.4939, 710.1)(877.5185, 1281.05)(878.5196, 1113.38)	20922	6114.909	27948.4	9605.667	45195.3	23412.82	43656.69	11373.16	0.98	8.31E-07	4.22E-06	0.19	0.372035	0.674033
COMPOUND_831_5784a12_152266	Rp	+	831.5784	12.15				(854.5672, 2692.92)(855.5663, 1557.43)(856.5854, 14265.81)(857.589, 8078.43)(870.5135, 1972.54)(871.5035, 1779.89)(832.5838, 8818.51)(833.5822, 3134.95)(834.5867, 1188.17)(835.595, 356.76)(836.5758, 88.62)	102400.9	67683.2	81088.7	40379.07	184420.5	72750.72	221073	125052.9	0.86	0.000619	0.001691	0.32	0.266343	0.575669
COMPOUND_831_579a12_280395	Rp	+	831.5790	12.28	PC 40:7	2	20 eV: 832.5886(100), 184.0757(70), 526.3304(1)	(854.5667, 6661.08)(855.5704, 3952.14)(856.5737, 1617.89)(857.5652, 288.63)(832.5856, 175783.92)(833.5881, 90610.39)(834.5906, 25593.7)(835.5905, 5476.87)(836.5857, 1102.63)(837.5959, 155.16)	911135.2	269545	839703.6	288863.1	1131202	296580.5	1248196	344083.5	0.56	0.027265	0.051815	0.62	0.034866	0.249266
COMPOUND_831_5983a13_031429	Rp	-	831.5983	13.03	PC 18:1_18:1	1	10 eV: 830.5915(100), 129.8537(1)	(830.5906, 656541.4)(831.5945, 319159.53)(832.5974, 87568.98)(833.5998, 17446.6)(834.6, 3246.99)(835.5999, 585.29)	16532075	2950865	18152174	3978595	17314286	2586610	16729342	3429937	0.14	0.608371	0.701152	-0.56	0.092566	0.360654
COMPOUND_833_54a11_007691	Rp	-	833.5400	11.01				(832.5336, 69023.11)(833.5367, 32024.01)(834.5487, 88173.75)(835.5524, 38617.56)(836.5549, 12424.98)(837.5556, 3501.73)(878.5387, 5073.91)(879.5344, 2533.45)(880.533, 9556.14)(881.535, 4955.64)(882.5486, 34226.4)	392251.3	217103.1	430354.2	203125.6	574868.3	222418	353184.7	95227.61	-0.87	0.000122	0.000371	-0.04	0.861242	0.942428
COMPOUND_833_5987a12_77569	Rp	+	833.5987	12.78	PC 40:6	2	20 eV: 834.6035(100), 184.0745(70)	(856.5828, 23340.16)(857.5853, 12760.42)(858.5806, 4897.04)(859.5867, 1425.19)(851.6275, 383.18)(852.6428, 1365.23)(834.6017, 610261.56)(835.6058, 310496.22)(836.6093, 94786.84)(837.6121, 21835.19)(838.613, 4721.11)(839.6112, 952.45)	4613007	1485995	4096511	1250114	4305461	1281807	4427254	1449803	-0.08	0.76159	0.827751	0.36	0.236779	0.542473
COMPOUND_833_6104a10_008281	Rp	+	833.6104	10.01				(845.5835, 300.59)(846.0697, 281.78)(846.5596, 527.94)(864.6205, 258.75)(865.11, 222.53)(865.5986, 1802.97)(866.1031, 1525.18)(866.5994, 1150.26)(447.7757, 42.68)(448.2769, 464.95)(428.8034, 1626.87)(429.3026, 1285.16)(429.8052, 318.97)(426.3268, 10875.03)(426.8288, 5699.68)(427.3223, 3501.88)(427.8298, 543.11)(445.2931, 5554.59)(445.7801, 692.27)(872.5658, 1331.97)(873.5792, 963.09)(874.5763, 1239.44)(834.6156, 14979.03)(835.6186, 7335.46)(836.6001, 3294.73)(837.5857, 1141.93)(838.6032, 244.1)(417.8128, 6108.87)(418.3149, 3870.4)(418.8157, 885.43)	311540.7	81268.22	267391.9	63365.94	357339.5	59081.92	357156.9	94252.08	0.37	0.175824	0.261357	0.65	0.051421	0.283822
COMPOUND_835_5349a12_906634	Rp	-	835.5349	12.91				(834.5258, 5905.01)(835.53, 2918.98)(836.532, 773.95)(837.5315, 183.4)	15915	7034.581	26352.6	4676.498	28207.5	8458.043	31382.53	9537.65	1.10	4.34E-09	3.87E-08	0.18	0.341169	0.646185
COMPOUND_835_5568a11_1571455	Rp	-	835.5568	11.16				(834.5489, 664335.44)(835.5529, 305854.4)(836.5556, 85495.5)(837.5582, 17772.52)(838.5498, 4161.19)(839.5278, 455.33)(880.5427, 4779.23)(881.5383, 2409.65)	4219985	1542341	4580985	1946887	1522225	1746085	980926.8	1320236	-1.32	1.7E-09	1.76E-08	-0.21	0.339499	0.643562
COMPOUND_835_5706a12_436599	Rp	-	835.5706	12.44				(834.5645, 15042.87)(835.5673, 7828.43)(836.5793, 18975.79)(837.5821, 8903.93)(838.5814, 3122.84)(839.5785, 906.06)(840.5726, 7841.65)	179175	64787	166476.8	56980.27	166795	47762.92	176163.1	53804.34	-0.11	0.647879	0.736881	0.40	0.155381	0.466404
COMPOUND_835_6102a13_016415	Rp	+	835.6102	13.02	PC 40:5	2	40 eV: 184.0735(100), 86.0946(5), 601.2265(2), 836.6168(1)	(858.597, 12668.02)(859.5986, 6874.6)(860.6054, 2962.44)(861.6051, 1076.72)(836.6176, 216215.86)(837.6207, 111657.25)(838.6239, 34164.57)(839.6253, 8262.01)(840.6213, 1713.03)(841.6298, 169.99)	1094125	536638.9	941098.5	364146.4	908615.4	274863.4	1051138	339652.9	-0.26	0.321078	0.423341	0.61	0.052346	0.286125

									1st trimester				3rd trimester									
									Control		Pre-eclampsia		Control		Pre-eclampsia		Time			Interaction		
Compound	Column	Ion_mode	Mass	Retention_	Identification	ID_level	MSMS_spectra	CompositeSpectrum	Mean	SD	Mean	SD	Mean	SD	Mean	SD	Value	raw p	FDR_p	Value	raw p	FDR_p
COMPOUND_837_5021a12_498895	Rp	+	837.5021	12.50				(430.757, 378.34)(431.2558, 315.4)(431.7586, 1534.73)(432.2617, 955.61)(419.7582, 6603.31)(420.2599, 3354.51)(420.7596, 1904.23)(421.2583, 569.11)	183900	28540.84	176756	28562.75	212161	28944.67	210302.6	34922.67	0.80	0.002689	0.006491	0.19	0.520208	0.764408
COMPOUND_837_5506a11_985389	Rp	-	837.5506	11.99				(950.5295, 476.07)(836.5437, 32130.73)(837.5472, 16858.51)(838.5454, 5644.38)(839.544, 1688.51)	187957.2	57487.78	181838.8	66687.74	215062.4	95351.38	195125.8	65290.21	0.29	0.291381	0.390822	-0.08	0.790424	0.91097
COMPOUND_837_5549a10_855005	Rp	+	837.5549	10.86				(860.5452, 132054.03)(861.5478, 64274.79)(862.5573, 64692.66)(863.5613, 27184.33)(864.5748, 39702.54)(865.5789, 19612.3)(866.5769, 15403.27)(838.5626, 280632.5)(839.5661, 133280.89)(840.5756, 145597.3)(841.579, 61393.86)(842.5838, 45433.48)(843.5856, 18807.21)(419.777, 1082.0)(420.2814, 683.58)	6645907	2064785	6251599	2260026	4679524	3999423	5598147	3503666	-1.08	3.43E-06	1.51E-05	-0.10	0.69873	0.862515
COMPOUND_837_5621a11_493071	Rp	-	837.5621	11.49				(836.5553, 7866.89)(837.5598, 3619.13)(838.5559, 3658.61)(839.5577, 1414.14)(840.5475, 2648.39)(882.5559, 3599.92)(883.5505, 2255.0)(884.5615, 12080.28)(885.5649, 6469.42)	212199.6	138130.9	242615.3	148099.2	319164.7	63394.92	306739.9	108163.1	-0.63	0.007618	0.016607	-0.22	0.434776	0.712127
COMPOUND_837_5871a12_651116	Rp	-	837.5871	12.65				(872.5635, 3500.71)(873.5758, 1408.05)(874.5836, 478.82)(950.5019, 2205.05)(951.5125, 1257.66)(952.516, 1636.76)(836.5801, 53792.73)(837.5834, 28835.99)(838.5867, 9286.66)(839.5895, 2365.0)(882.5732, 1362.22)(883.5616, 375.86)	438520.9	125731.6	424145.9	136751.4	426574.9	123716.7	427441.6	109523.4	-0.02	0.938265	0.960006	0.27	0.421538	0.702667
COMPOUND_839_5174a12_975627	Rp	+	839.5174	12.98				(840.5051, 237.58)(420.7655, 7106.43)(421.2671, 3835.82)(421.7676, 1799.18)(422.27, 644.58)	154742	23972.77	158831.8	32957.39	167772.3	20600.12	156592.4	24895.52	0.47	0.104869	0.169781	-0.53	0.128704	0.425125
COMPOUND_839_6025a12_830119	Rp	-	839.6025	12.83				(874.5696, 6198.27)(875.5703, 1499.08)(876.579, 559.74)(838.5958, 63200.59)(839.5992, 31762.14)(840.6005, 9558.31)(841.6001, 2294.57)	891316.5	186112.4	877433.1	204628.5	865890.8	146327.7	774753.7	166041.7	-0.12	0.658034	0.745794	-0.41	0.190426	0.505078
COMPOUND_840_6351a0_8306668	Hilic	+	840.6351	0.83				(863.6162, 3292.77)(864.6185, 498.54)(865.635, 2115.41)(858.6744, 742.72)(841.643, 29166.79)(842.6451, 15601.36)(843.6582, 37325.41)(844.6608, 20379.7)(845.6679, 9049.09)(846.6715, 4129.02)(847.676, 1466.55)	219159.8	118464.4	239060.9	143443.8	92929.8	64385.88	73186.63	55353.65	-1.31	3.53E-10	4.47E-09	-0.21	0.326174	0.633587
COMPOUND_841_5387a12_21047	Rp	-	841.5387	12.21				(840.5316, 12929.77)(841.5375, 6490.76)(842.5357, 7739.24)(843.5399, 3689.78)(844.5365, 6498.16)	425455.4	63257.31	416863.8	66969.13	395830.9	53671.69	363245.4	77089.9	-0.45	0.083714	0.138825	-0.25	0.40105	0.690408
COMPOUND_841_5463a11_111553	Rp	-	841.5463	11.11				(840.5392, 157625.58)(841.5422, 75717.93)(842.5533, 118932.28)(843.5573, 51879.09)(844.5641, 24727.83)	1435265	461075.3	1525346	469578.2	1590718	248183	1615442	390554.8	-1.05	2.68E-06	1.21E-05	-0.16	0.499897	0.753664
COMPOUND_841_5824a12_602464	Rp	-	841.5824	12.60				(876.5722, 2181.46)(877.5757, 1155.11)(840.5744, 14422.15)(841.5776, 7199.01)(842.555, 7566.97)(843.5606, 4208.61)	211307.7	79084.89	215800.5	91977	138492.1	49490.64	160872.1	42889.67	-0.67	0.012657	0.026173	-0.21	0.507669	0.75635
COMPOUND_843_5089a12_177403	Rp	+	843.5089	12.18				(863.4988, 5635.0)(864.0132, 1165.49)(864.5028, 3661.96)(865.016, 821.09)(865.5057, 1612.18)(866.0142, 963.7)(855.5065, 2049.25)(856.0202, 1539.72)(853.0107, 805.85)(853.5202, 1200.96)(871.9999, 755.59)(872.4751, 2236.88)(861.5061, 1588.66)(862.009, 1151.41)(844.5215, 4802.28)(845.523, 2743.51)(846.5205, 2144.41)(847.4984, 666.76)(422.7646, 22262.95)(423.2667, 11727.59)(423.7682, 3952.95)(424.2689, 1085.1)	262062.5	71253.53	280847.1	92031.44	299297.4	94027.72	265440.9	84790.3	0.36	0.223561	0.31724	-0.67	0.056798	0.296083
COMPOUND_843_5233a11_918069	Rp	-	843.5233	11.92				(956.4877, 1858.02)(957.4897, 1111.55)(958.4861, 1222.69)(959.4784, 992.84)(878.4758, 1442.31)(842.5161, 15421.51)(843.5192, 6976.63)(844.5374, 2930.06)(845.5176, 654.3)(846.5348, 109.97)(888.5141, 3554.3)(889.5136, 2223.36)	119269.9	42689.53	141762	47272.44	197370.5	59157.61	187565.4	62760.25	1.38	5.71E-08	3.85E-07	-0.52	0.048035	0.279822
COMPOUND_843_5448a11_193773	Rp	+	843.5448	11.19				(861.5992, 7292.52)(882.5095, 2918.47)(844.5482, 244878.12)(845.551, 120780.64)(846.5571, 47056.94)(847.563, 14779.55)	896802.4	284914.7	818996.3	339878.6	865036	504626.8	1000700	591399.9	-0.57	0.016603	0.033474	-0.10	0.727753	0.883503
COMPOUND_843_5544a12_41593	Rp	-	843.5544	12.42				(842.5464, 9807.26)(843.5487, 5000.24)(844.544, 5153.56)(845.5468, 2251.03)	77755.66	27096.7	64867.83	17722.65	73814.79	25576.69	80605.2	23209.89	0.19	0.476484	0.580023	0.77	0.018669	0.196411
COMPOUND_843_5617a11_3645315	Rp	-	843.5617	11.36				(842.5549, 138360.3)(843.558, 67369.73)(844.5681, 73688.38)(845.5723, 30952.59)(846.586, 242580.2)(847.5893, 114349.02)(848.5918, 32012.82)(849.5938, 7254.95)	2364827	1714728	2602692	1466770	1856893	2964121	1221505	2468429	-0.52	0.031068	0.058111	-0.20	0.467769	0.734042
COMPOUND_843_5637a11_075828	Rp	-	843.5637	11.08	PC 17:0_20:4	1	20 eV: 782.533(100), 842.555(37), 295.2257(6)	(878.5645, 9810.22)(879.5692, 4210.36)(880.5415, 5152.79)(881.5398, 2400.51)(842.5532, 87912.63)(843.5568, 40084.68)(844.5634, 17794.39)(845.5676, 5789.32)(846.5649, 930.11)	887995.9	306850.2	999519	303545.7	410953	378851.7	351502.2	472710.8	-1.24	2.49E-08	1.85E-07	-0.23	0.31531	0.621785
COMPOUND_843_5976a12_88655	Rp	-	843.5976	12.89				(956.6061, 212.42)(957.5932, 206.83)(842.5908, 12985.37)(843.5935, 6415.49)(888.61, 1626.03)(889.6127, 1031.0)(890.6145, 387.86)	114461.6	24417.07	113635.3	34409.66	100680.4	21592.73	104962	22457.79	-0.41	0.169042	0.252441	0.27	0.446567	0.72091
COMPOUND_845_5402a12_228107	Rp	-	845.5402	12.23				(844.5321, 18620.79)(845.5348, 8395.05)(846.5389, 2444.2)(847.5447, 753.99)(890.5245, 1725.29)(891.5266, 1135.33)	76498.16	25772	86535.26	38296.54	88700.57	31409.08	104533.6	38635.38	0.46	0.101508	0.164813	0.14	0.681416	0.853001
COMPOUND_845_5697a12_936705	Rp	-	845.5697	12.94				(844.5631, 7445.44)(845.5668, 3775.37)(846.5662, 4609.89)(847.572, 2688.53)(848.5768, 1410.37)(849.5802, 603.36)	340657.4	67088.85	317190	80641.94	284792.3	50282.74	263615.5	63052.38	-0.55	0.009234	0.019693	-0.14	0.560744	0.783425
COMPOUND_845_57a12_689569	Rp	-	845.5700	12.69				(844.564, 11263.72)(845.5654, 6217.92)(846.5549, 9120.13)(847.5569, 4275.37)(848.5712, 2135.95)(849.5786, 888.01)	113308.2	30298.72	112093.8	25321.01	118864	19898.31	126667.8	22424.14	0.14	0.629375	0.7202	0.43	0.205213	0.516497
COMPOUND_847_1737a9_568081	Rp	-	847.1737	9.57				(960.1473, 594.48)(846.1685, 3284.04)(847.1741, 1543.67)(848.1782, 413.6)(892.1787, 744.53)	38608.53	9259.682	36617.13	10885.88	38887.54	5501.294	39507.42	12177.07	-0.18	0.553542	0.651294	0.43	0.232813	0.538289
COMPOUND_847_5844a13_380447	Rp	-	847.5844	13.38				(846.5776, 17851.84)(847.5806, 9345.02)(848.5772, 7992.6)	368580.1	141493.1	392426.2	120748.8	363507.8	91220.24	397294.4	116410.3	0.09	0.746612	0.815803	-0.10	0.739796	0.888787
COMPOUND_847_5929a11_493024	Rp	-	847.5929	11.49	PC 18:0_HODE	1	10 eV: 846.5862(100), 786.563(25), 131.4722(2)	(882.5545, 6499.76)(846.586, 403208.3)(847.5896, 193135.11)(848.5921, 56020.31)(849.5948, 12015.87)(850.5769, 2988.23)	2565134	902716.9	2870553	987657.1	1011715	1117313	663626.2	1008531	-1.28	1.04E-08	8.59E-08	-0.26	0.259107	0.570391
COMPOUND_847_6225a10_199461	Rp	+	847.6225	10.20				(870.587, 475.49)(871.0964, 253.23)(871.6019, 574.58)(870.5927, 152.96)(871.5695, 764.35)(878.5903, 648.24)(879.1041, 620.9)(879.6055, 956.07)(876.1117, 351.67)(876.6233, 481.89)(868.0923, 429.12)(868.586, 372.96)(886.6066, 558.98)(887.1143, 582.67)(887.6152, 3988.65)(435.8108, 286.84)(436.2919, 979.27)(436.8019, 100.77)(437.2975, 375.52)(433.3343, 8486.05)(433.8353, 2929.59)(434.337, 1038.59)(434.8275, 223.24)(452.2833, 528.26)(452.7917, 60.96)(453.2985, 1259.7)(444.3312, 852.87)(444.8238, 50.13)(445.3252, 531.3)(848.6306, 6752.53)(849.6337, 3269.59)(850.6051, 3420.3)(851.5964, 1871.53)(424.821, 2770.1)(425.3216, 1318.35)(425.825, 555.55)	158331	58580	131501.9	38648.28	154732.2	30778.03	150978.2	50752.83	-0.03	0.906257	0.938328	0.53	0.134658	0.43508
COMPOUND_849_5531a11_884156	Rp	-	849.5531	11.88				(962.5049, 2142.81)(963.5114, 1418.6)(848.5441, 57258.01)(849.5472, 29906.15)(850.5572, 36012.08)(851.5617, 16036.13)(852.5655, 4917.92)(894.559, 7134.01)	657891.2	285796.8	752723	276178.9	911857.2	362546.9	999048.4	295063.8	0.67	0.007661	0.016685	-0.03	0.907398	0.958319
COMPOUND_849_5893a11_479956	Rp	+	849.5893	11.48				(872.5779, 12642.25)(873.5809, 6947.27)(874.5873, 7394.11)(875.5905, 3850.51)(888.567, 6910.12)(889.5728, 3720.78)(850.5963, 51051.54)(851.5994, 25612.62)(852.6095, 26785.88)(853.6131, 12023.21)(854.6142, 5941.21)	963155.8	514470.2	1023460	443831.7	806899.3	561867.9	939969	331245.8	-1.09	1.15E-06	5.64E-06	-0.20	0.397464	0.690408
COMPOUND_849_6068a9_576319	Rp	+	849.6068	9.58				(436.8014, 2295.38)(437.3046, 1280.97)(437.8062, 476.35)(438.3104, 179.2)(434.3248, 8657.83)(434.8265, 4253.12)(435.3308, 1233.97)(435.8206, 227.05)(867.6018, 222.99)(868.0876, 146.47)(850.6108, 11206.13)(851.613, 5256.82)(852.6148, 1676.23)(853.6156, 468.38)(854.6137, 99.42)(463.7573, 174.14)(464.2469, 125.65)(425.8115, 4247.73)(426.3124, 2003.5)(426.8142, 797.34)(427.3, 229.8)	433306.3	111949.2	368191	95754.95	464200.6	79208.19	459338.9	114009	0.22	0.442797	0.548202	0.60	0.084099	0.346484
COMPOUND_851_5664a12_071053	Rp	-	851.5664	12.07	PC 38:6	1	10 eV: 850.5583(100), 790.5399(25), 44.9979(2)	(850.5596, 186801.19)(851.5627, 96029.96)(852.5655, 28309.42)(853.5688, 6264.31)(854.5726, 1602.28)	1220419	365841.6	1176006	321891.6	1059140	329277.7	1134953	291300.2	-0.46	0.118646	0.188828	0.41	0.232155	0.537875
COMPOUND_851_5664a12_202712	Rp	-	851.5664	12.20	duplicate of PC 38:6			(850.5592, 999121.8)(851.563, 514154.6)(852.5669, 150589.47)(853.5698, 32998.07)(854.5742, 6297.01)(855.578, 1126.74)	10453910	1618109	10353963	1802363	10739770	2217858	10781411	1797030	0.21	0.457837	0.562492	0.07	0.831684	0.926681
COMPOUND_851_6019a10_662118	Rp	-	851.6019	10.66				(964.5508, 1757.0)(965.5441, 1233.22)(886.5709, 3114.74)(887.5764, 1421.07)(850.596, 88194.23)(851.5992, 47509.23)(852.5998, 14878.25)(853.5876, 4433.66)(854.5924, 1020.76)(855.5917, 200.94)	233645.8	72958.87	286270.7	154490	164450.5	81417.32	198332.6	87244.52	-0.62	0.022396	0.043517	0.13	0.683691	0.853695

									1st trimester				3rd trimester									
									Control		Pre-eclampsia		Control		Pre-eclampsia		Time			Interaction		
Compound	Column	Ion_mode	Mass	Retention_	Identification	ID_level	MSMS_spectra	CompositeSpectrum	Mean	SD	Mean	SD	Mean	SD	Mean	SD	Value	raw p	FDR_p	Value	raw p	FDR_p
COMPOUND_853_1224a9_243353	Rp	-	853.1224	9.24				(888.0863, 257.88)(889.0962, 290.25)(890.0971, 98.64)(852.1149, 11710.53)(853.1143, 3143.33)(854.1184, 671.04)(855.1392, 129.9)	56583.87	11129.95	62383.15	11670.84	54252.57	9922.074	53810.68	9888.124	-0.20	0.481825	0.583999	-0.50	0.150267	0.460409
COMPOUND_853_5644a11_888902	Rp	+	853.5644	11.89	PC 42:10	2	10 eV: 854.5741(100), 184.0707(15), 308.9499(5)	(876.5483, 5511.58)(877.5547, 3105.33)(878.5661, 3196.29)(879.5709, 1776.32)(854.5707, 50089.03)(855.5741, 27265.34)(856.5764, 8257.41)(857.5795, 1935.89)(858.5912, 519.67)	112991.6	42104.23	101166.4	41463.25	250838	106989.9	284874.9	132818.7	1.34	2.91E-09	2.75E-08	0.17	0.459546	0.729315
COMPOUND_853_5679a10_619556	Rp	-	853.5679	10.62				(852.5613, 36155.73)(853.564, 17475.78)(854.564, 5787.77)(855.5674, 1374.1)	104249.6	59975.47	124140.3	90262.18	61760.75	37317.29	45632	25508.38	-0.81	0.000137	0.000414	-0.56	0.021021	0.204049
COMPOUND_853_5824a12_488253	Rp	-	853.5824	12.49				(852.5756, 182937.38)(853.5787, 93563.52)(854.5813, 27139.1)(855.5841, 6024.84)(856.5859, 1389.09)(857.5958, 256.71)	2213187	706405.2	2191848	663083.7	1895173	347820	2096032	520902.9	-0.46	0.119143	0.189485	0.26	0.454644	0.725699
COMPOUND_853_5825a12_405809	Rp	-	853.5825	12.41				(852.5753, 385136.03)(853.5789, 194315.25)(854.5817, 55112.57)(855.5838, 11677.96)(856.5848, 2346.59)(857.5844, 412.47)	2572910	638087.2	2350576	565459.7	2147142	408088.8	2354627	530459.8	-0.60	0.022668	0.043932	0.66	0.029916	0.234557
COMPOUND_853_6569a10_853576	Rp	-	853.6569	10.85				(966.6678, 359.82)(967.6604, 2719.36)(968.6703, 1536.22)(888.5882, 2683.45)(852.65, 32592.36)(853.6534, 18394.81)(854.6559, 5698.44)(855.6583, 1990.65)(856.6652, 373.9)(898.6794, 637.37)(899.6781, 3525.79)	97375.77	20839.58	104889.1	18277.08	114915.3	24150.44	119300.9	24190.98	0.78	0.00701	0.0154	-0.21	0.539555	0.776408
COMPOUND_855_4734a12_004098	Rp	+	855.4734	12.00				(878.471, 7446.8)(879.4713, 6944.16)(873.5007, 5725.57)(874.4921, 4060.42)(875.493, 3434.38)(856.4843, 6461.08)(857.4902, 3517.68)(858.4861, 3529.13)(859.4933, 2125.74)(860.4888, 2277.36)(428.7468, 89.15)(429.2323, 71.21)(429.7465, 539.79)	291678.4	66628.31	273586.8	70583.47	314920.2	140314.5	333860.1	132360.3	0.42	0.138149	0.213422	0.48	0.153475	0.465803
COMPOUND_855_5737a12_795058	Rp	+	855.5737	12.80	PC 42:9	2	40 eV: 673.5219(100), 86.0965(45), 504.3401(9), 797.5095(18), 856.5798(15)	(878.551, 2808.02)(879.5238, 3138.55)(880.5135, 2122.49)(894.5348, 6062.56)(895.0384, 1684.35)(894.531, 4920.75)(895.5354, 3061.72)(896.529, 3136.49)(897.5282, 2010.92)(898.5246, 2444.24)(856.5824, 15269.99)(857.5854, 7943.64)(858.5778, 3423.29)(859.586, 309.54)(428.7857, 600.43)(429.2877, 463.72)(429.7906, 498.34)(430.2922, 283.9)	749492.3	276242.1	775141	251352	542181.5	197617	613668.3	218392.7	-0.66	0.02044	0.040237	0.18	0.595448	0.804532
COMPOUND_855_5781a12_068871	Rp	+	855.5781	12.07				(878.5646, 1332.38)(879.5682, 812.49)(880.5853, 1366.04)(881.6067, 1181.81)(856.586, 16187.36)(857.5896, 9569.68)(858.5924, 2911.69)(859.5901, 924.66)	59531.6	27650.77	50818.41	19528.46	98108.29	23919.67	125558.5	49637.96	1.03	6.06E-06	2.53E-05	0.55	0.031021	0.237615
COMPOUND_855_5972a12_688698	Rp	-	855.5972	12.69	PC 18:1_20:3	1	10 eV: 854.5909(100), 293.1318(4), 112.9855(2)	(854.5906, 136689.77)(855.5939, 68488.06)(856.5966, 19761.73)(857.5985, 4490.92)(858.5961, 1054.44)(859.6026, 163.81)	1574187	523990.4	1566756	424338.6	1629485	429566.6	1889253	398687.3	0.08	0.776736	0.836143	0.63	0.04967	0.282107
COMPOUND_855_5988a12_93301	Rp	-	855.5988	12.93	duplicate of PC38:3			(854.5907, 505598.25)(855.5949, 254650.9)(856.5975, 72126.91)(857.5998, 15288.09)(858.6015, 2907.63)(859.5997, 620.06)	5548839	1382421	5295836	1546768	4446272	1160565	4591829	1251005	-0.71	0.003485	0.008158	0.23	0.395701	0.690408
COMPOUND_857_54a10_777037	Rp	-	857.5400	10.78				(970.5227, 3586.53)(856.5334, 40229.61)(857.5365, 20344.39)(858.5486, 67221.84)(859.552, 31603.2)(860.5615, 25529.21)(861.5654, 10264.87)(862.5632, 3631.53)(863.5671, 847.59)	704765.5	376638.8	674808.4	217443.2	863226.3	104992.9	675937	326530.5	-0.81	0.000624	0.001698	-0.15	0.576937	0.792233
COMPOUND_857_5899a12_4159565	Rp	+	857.5899	12.42				(858.6018, 6323.54)(859.6084, 3734.54)(860.6046, 1128.06)(861.6064, 182.46)	33639.1	11929.76	31584.16	12857.23	37888.86	14033.62	45815	15453.46	0.60	0.020289	0.039974	0.37	0.221441	0.531023
COMPOUND_859_5564a10_864677	Rp	-	859.5564	10.86				(972.5487, 1782.32)(973.5339, 1081.56)(974.523, 3602.46)(975.5279, 2253.3)(858.5493, 144964.89)(859.5526, 69863.63)(860.5634, 91865.18)(861.5671, 40783.84)(862.5701, 12104.31)(863.5738, 2820.85)	1567131	475264.9	1522367	477643.4	346341.2	557829	278938.8	528573.6	-1.46	4.45E-09	3.97E-08	0.05	0.835867	0.929001
COMPOUND_859_6094a12_872848	Rp	+	859.6094	12.87				(882.5651, 1342.87)(883.5839, 799.6)(884.6046, 177.66)(860.6156, 13095.83)(861.6209, 6749.96)(862.6244, 3054.75)(863.6259, 819.61)(864.632, 213.3)	80894.52	36788.07	82381.75	37149.36	99623.14	47774.72	104670.3	39594.45	0.50	0.085652	0.141726	0.17	0.625453	0.823696
COMPOUND_861_5714a11_229768	Rp	-	861.5714	11.23				(860.5634, 67023.38)(861.5671, 31447.31)(862.5784, 34358.9)(863.583, 14592.94)(864.5735, 5244.32)(865.5892, 1162.74)	1379092	995115.8	1489446	962592.3	703181.7	434974.5	920649.3	833367.4	-1.27	3.87E-09	3.55E-08	-0.07	0.74324	0.889774
COMPOUND_861_6166a11_682631	Rp	+	861.6166	11.68				(884.608, 3850.15)(885.6114, 2330.58)(886.613, 1122.6)(887.6106, 412.89)(900.613, 644.56)(901.5987, 338.56)(902.6251, 631.62)(879.6271, 445.89)(880.6474, 2172.4)(881.6551, 1503.86)(862.6293, 15256.46)(863.6311, 7778.65)(864.6273, 3218.52)(865.6397, 921.9)	49004.17	17627.67	46304.88	15662.63	32766.69	7850.787	31351.47	15261.35	-0.66	0.010923	0.022883	0.33	0.272998	0.583098
COMPOUND_863_5663a10_005866	Rp	-	863.5663	10.01				(976.5429, 448.67)(977.5579, 344.57)(978.5438, 591.29)(862.5591, 25712.38)(863.5621, 13396.9)(864.5666, 4046.72)(865.5693, 1076.69)(866.5769, 276.84)	40249.88	21067.44	48068.59	21308.5	32755.25	13515.59	50158.8	22550.7	-1.02	6.64E-06	2.74E-05	-0.32	0.199021	0.510852
COMPOUND_863_5883a11_446689	Rp	-	863.5883	11.45				(862.5807, 624020.4)(863.5846, 296275.94)(864.5875, 85130.76)(865.5892, 18242.36)	2423648	1193054	2625359	1361449	1414964	537617.4	1018539	490826	-1.34	6.17E-11	1.05E-09	-0.18	0.380816	0.683863
COMPOUND_863_6009a12_710474	Rp	-	863.6009	12.71				(898.5674, 7236.8)(862.5953, 42912.13)(863.5988, 23039.66)(864.6003, 7263.45)(865.6018, 1668.78)	194246.4	72841.85	189240.9	60555.75	258110.7	74410.82	240557.3	69562.23	0.60	0.016233	0.032846	0.12	0.66252	0.844492
COMPOUND_865_5827a12_497206	Rp	-	865.5827	12.50				(978.5699, 140.67)(900.5828, 558.8)(901.5427, 1598.24)(864.5751, 15173.69)(865.5783, 7924.33)(866.5805, 3217.39)(867.5829, 1046.6)(910.5595, 1983.01)(911.5364, 676.72)	131925.4	52833.79	123870.2	57668.6	124102.1	69080	115013.8	52159.05	-0.22	0.454779	0.560264	0.04	0.916643	0.961472
COMPOUND_867_5289a0_7175961	Hilic	+	867.5289	0.72				(868.536, 33778.23)(869.5388, 17485.6)(870.5492, 109314.16)(871.5527, 54297.96)(872.5636, 75841.38)(434.7724, 631.35)(435.2684, 624.13)(435.7752, 648.68)	2038900	707149.5	1891954	701534.7	1841865	#DIV/0!	1057227	689261.4	-1.26	3.37E-08	2.41E-07	0.35	0.139968	0.444883
COMPOUND_867_562a11_215339	Rp	-	867.5620	11.22				(866.5548, 158620.58)(867.5579, 79788.14)(868.5679, 89358.26)(869.5723, 39040.52)(870.5775, 14810.35)	2045736	676103.2	2020121	906974.2	2316922	234333.9	2313902	960247.6	-0.97	3.65E-05	0.000126	-0.19	0.456343	0.727307
COMPOUND_869_5384a12_06106	Rp	-	869.5384	12.06				(868.5316, 32468.06)(869.5347, 16376.55)(870.5473, 6258.69)(871.5591, 1961.23)(872.5543, 98.94)	348594.2	87588.7	359589.1	104039.1	422118.2	104880.9	409429.1	111942.2	0.82	0.004428	0.010103	-0.17	0.619178	0.817823
COMPOUND_869_5443a12_989213	Rp	+	869.5443	12.99	no msms			(908.5166, 6033.34)(870.5525, 10087.8)(871.5553, 5439.46)(872.5492, 2372.47)	340572.7	35205.61	344387.4	39221.48	369250.9	34606.87	331597.6	49211.02	0.82	0.005835	0.01301	-1.20	0.000801	0.045795
COMPOUND_871_5529a12_403818	Rp	-	871.5529	12.40				(984.5138, 3995.88)(985.5143, 2349.84)(906.5119, 703.04)(907.5146, 561.2)(908.5178, 681.38)(870.5471, 52322.24)(871.5506, 25619.41)(872.5539, 7743.86)(873.5581, 1634.74)	872208.9	217061.9	937102.7	258289.4	787595.8	164895.5	739233.4	162437.7	-0.31	0.259017	0.355891	-0.48	0.142694	0.448182
COMPOUND_873_5699a12_794237	Rp	-	873.5699	12.79				(872.5623, 46346.39)(873.5658, 22435.4)(874.5687, 6775.8)(875.57, 1521.27)(876.5851, 413.05)	1134887	179976.8	1195510	185016.8	1242948	150055.9	1215792	239713	0.57	0.04454	0.07898	-0.43	0.196094	0.507591
COMPOUND_873_6066a11_643519	Rp	-	873.6066	11.64				(872.5993, 59703.85)(873.6033, 27122.22)(874.6029, 8776.44)(875.6085, 2292.37)(918.5993, 479.41)(919.5903, 569.74)	836478.3	289082.2	961039.7	322166.8	693475.5	322653.6	704122.5	350277.4	-1.00	6.21E-06	2.59E-05	-0.34	0.16387	0.47785
COMPOUND_875_5362a10_856782	Rp	+	875.5362	10.86				(914.4878, 2608.98)(915.4867, 2521.7)(916.4926, 3315.51)(917.4848, 2625.24)(918.507, 3098.17)(893.5886, 2947.03)(876.5418, 59543.59)(877.5443, 29995.61)(878.5533, 34677.95)(879.5568, 16494.52)(880.5692, 23791.25)(881.5721, 11930.88)(882.5832, 21955.71)(438.7904, 63.42)(439.3085, 234.78)(439.7988, 192.76)	920748.9	382633.9	870248.6	408475.7	810641.3	365388.5	906410.8	330772	-0.79	0.000699	0.001888	0.03	0.920854	0.964541
COMPOUND_875_567a11_979936	Rp	-	875.5670	11.98				(874.5594, 35032.57)(875.5623, 19119.71)(876.5643, 6410.59)(877.5634, 1489.55)	254488.2	59673.07	258911.6	66685.19	314909.5	78316.51	337952.4	67735.95	0.79	0.00314	0.007444	0.26	0.395579	0.690408
COMPOUND_877_4441a12_5313635	Rp	-	877.4441	12.53				(876.4374, 10111.48)(877.4409, 4291.51)(878.4386, 6376.93)(879.4379, 3560.59)(880.4361, 4731.32)(881.4388, 2122.08)(882.4404, 792.31)(883.435, 304.15)(884.4273, 638.62)(922.454, 3341.32)(923.4555, 1621.44)	34212.7	16783.03	35778.53	20102.67	43498.64	21054.61	39681.84	15053.41	0.68	0.014067	0.02893	-0.36	0.260621	0.572854
COMPOUND_877_5588a11_824242	Rp	+	877.5588	11.82				(900.5363, 1236.46)(901.5493, 761.75)(902.5669, 228.47)(878.5687, 7903.45)(879.5737, 4646.77)(880.5741, 1700.38)(881.5875, 557.64)	#DIV/0!	#DIV/0!	#DIV/0!	#DIV/0!	#DIV/0!	#DIV/0!	#DIV/0!	#DIV/0!	1.09	1.76E-06	8.32E-06	0.01	0.966858	0.982565
COMPOUND_877_58a12_184313	Rp	-	877.5800	12.18				(876.5753, 27360.66)(877.5789, 14345.13)(878.5817, 4978.93)(879.5818, 1263.56)(880.5739, 278.24)(881.5978, 44.31)	133079	146928.4	96154	115665.6	79435.29	102671.6	73452.33	50977.47	-0.19	0.484448	0.585603	0.99	0.002828	0.074044
COMPOUND_877_5821a12_295855	Rp	-	877.5821	12.30				(876.5752, 80287.02)(877.5784, 42952.64)(878.5809, 13000.82)(879.5836, 3164.67)(880.583, 678.24)(881.5973, 143.32)	412320.8	86060.23	414992.4	88612.83	485798.7	142702.3	512945	139658.5	0.47	0.069425	0.118263	0.32	0.288421	0.597533

									1st trimester				3rd trimester									
									Control		Pre-eclampsia		Control		Pre-eclampsia		Time			Interaction		
Compound	Column	Ion_mode	Mass	Retention_	Identification	ID_level	MSMS_spectra	CompositeSpectrum	Mean	SD	Mean	SD	Mean	SD	Mean	SD	Value	raw p	FDR_p	Value	raw p	FDR_p
COMPOUND_877_6384a9_982376	Rp	+	877.6384	9.98				(897.6023, 321.55)(898.1133, 108.3)(889.6105, 117.03)(890.1113, 159.47)(890.6018, 295.86)(906.1191, 178.75)(906.639, 397.22)(907.1318, 162.04)(916.6289, 401.69)(917.1293, 277.55)(917.6197, 406.1)(450.817, 3083.42)(451.3211, 2159.51)(451.8158, 660.37)(916.6174, 532.08)(917.6237, 273.69)(448.3401, 20932.01)(448.8407, 10944.47)(449.3435, 3607.41)(449.8421, 1033.67)(450.3423, 288.9)(878.6417, 16077.99)(879.6449, 7689.43)(880.6339, 2953.61)(881.6383, 696.72)(882.6482, 117.91)(439.8263, 9235.6)(440.3276, 4658.19)(440.8293, 1553.74)	463928	125658.7	396042.5	83952.69	514945.5	83915.47	514964.2	129762.6	0.29	0.296057	0.396151	0.68	0.045482	0.273371
COMPOUND_878_5307a12_098638	Rp	-	878.5307	12.10				(877.5222, 8412.8)(878.5182, 6831.33)(879.5174, 3065.41)	88700.03	18471.64	90934.29	22261.73	84751	10277.35	78439.95	15814.81	0.03	0.923033	0.950466	-0.47	0.156467	0.46686
COMPOUND_879_5981a12_817843	Rp	-	879.5981	12.82				(878.5912, 168541.11)(879.5943, 89959.1)(880.5977, 29492.33)(881.6009, 7851.69)(882.5984, 2170.23)(924.5823, 4041.15)	2023915	556989.4	1971626	508965.1	1907878	642211.1	1974150	619831.7	-0.14	0.58824	0.683887	0.21	0.47205	0.734155
COMPOUND_881_489a12_250776	Rp	+	881.4890	12.25				(899.523, 5651.88)(900.5009, 939.74)(901.5177, 1960.77)(902.5027, 1367.77)(882.4955, 22374.69)(883.4994, 11990.29)(884.497, 16945.2)(885.5002, 8274.24)(886.4954, 7542.45)(887.498, 3894.02)(888.4969, 2476.76)	209625.1	57174.67	170458.5	42218.44	179080.9	40667.16	191052.8	50658.45	-0.58	0.048728	0.085503	0.96	0.007258	0.132885
COMPOUND_881_5915a10_865179	Rp	-	881.5915	10.87				(880.5873, 13788.63)(881.5918, 7163.23)	35215	19581.95	41335.26	25072.6	16619	797.6164	17343.8	3956.935	-1.11	6.74E-08	4.47E-07	-0.34	0.115199	0.402603
COMPOUND_883_5567a10_840087	Rp	-	883.5567	10.84				(996.5294, 2137.47)(997.5313, 1238.26)(998.5302, 874.19)(999.5435, 234.96)(882.5497, 109292.85)(883.5528, 54177.65)(884.5626, 58550.98)(885.5668, 25211.99)(886.5753, 19730.6)(887.5791, 8356.82)	2725266	847508.2	2688474	760442.4	2285760	1243093	2180059	1585163	-1.13	2.23E-06	1.03E-05	-0.04	0.873494	0.946337
COMPOUND_889_5578a13_074443	Rp	-	889.5578	13.07				(888.5495, 6976.01)(889.5523, 3654.57)(890.5413, 3749.53)(891.5415, 1814.07)(892.5388, 926.91)(893.5371, 348.83)(894.5497, 1041.26)(895.5643, 640.06)(896.5625, 1926.88)(897.5667, 1062.48)	211839.5	71866.59	222843.3	73584.51	243939	71084.53	224649.5	64686.26	0.36	0.204774	0.29561	-0.71	0.034706	0.249266
COMPOUND_889_6013a11_607544	Rp	-	889.6013	11.61				(924.5597, 602.06)(925.555, 502.77)(926.5351, 1450.49)(888.5959, 50135.07)(889.5996, 24614.44)(890.6051, 10061.99)(891.6096, 3353.93)(892.6118, 577.11)	528832.4	262733.3	503227	219526.5	257459.3	147759.5	305291	154986.6	-1.33	3.31E-10	4.3E-09	-0.04	0.865373	0.944172
COMPOUND_891_649a10_170948	Rp	+	891.6490	10.17				(911.6021, 176.97)(912.1275, 218.77)(922.6463, 463.15)(923.1315, 593.05)(923.6352, 872.49)(930.5511, 934.05)(931.0673, 58.97)(457.8254, 1117.72)(458.337, 591.98)(458.8256, 339.08)(455.3465, 15239.83)(455.8487, 7845.38)(456.3475, 2745.44)(456.8493, 598.0)(930.5747, 327.87)(892.6567, 9875.08)(893.6602, 5047.94)(894.6522, 2072.91)(895.6415, 659.64)(446.8344, 4609.3)(447.3353, 3267.82)(447.8357, 788.13)	247244.4	60807.96	211653.6	47135.13	277628.5	46371.09	277614.3	63707.1	0.42	0.128419	0.201673	0.62	0.061559	0.303349
COMPOUND_893_5765a11_273696	Rp	-	893.5765	11.27				(928.5396, 10092.85)(929.5382, 5371.49)(930.5496, 17115.15)(931.5501, 9735.66)(932.5352, 6014.14)(933.532, 3166.45)(934.5389, 8715.35)(892.5696, 10318.9)(893.5731, 5803.1)(894.5845, 19181.92)	343466.6	53871.24	374664.6	56108.02	421611.3	72098.6	459885.8	134475.4	-0.66	0.007557	0.01649	-0.37	0.196685	0.507758
COMPOUND_893_6332a9_570584	Rp	+	893.6332	9.57				(466.7625, 146.72)(467.2653, 1467.1)(467.7825, 311.92)(458.8152, 1493.44)(459.3267, 5306.9)(459.8199, 276.97)(460.3319, 1030.8)(456.3374, 13402.72)(456.8395, 6551.44)(457.3391, 2576.4)(457.8433, 586.51)(458.3425, 159.45)(894.6358, 7786.54)(895.6397, 3958.09)(896.6417, 1387.64)(897.6436, 331.26)(485.7973, 274.55)(486.2935, 100.68)(447.824, 5301.19)(448.3268, 2672.19)(448.8258, 1083.97)(449.3135, 104.06)	441258.8	112116.6	387192.7	83135.28	478367.5	75095.92	472050.8	119569	0.28	0.320082	0.422275	0.52	0.127345	0.424221
COMPOUND_894_198a9_36771	Rp	-	894.1980	9.37				(1007.188, 68.72)(1008.1517, 259.23)(1009.1497, 231.27)(893.1906, 16254.78)(894.1943, 5038.86)(895.1872, 1053.42)(896.1681, 228.12)	99390.45	19802.42	101281.1	16366.54	95794.79	18397.92	95743.7	14877.06	-0.20	0.4958	0.596762	-0.16	0.65175	0.83936
COMPOUND_894_1982a9_601177	Rp	-	894.1982	9.60				(929.1535, 689.54)(930.1559, 233.75)(931.1781, 116.25)(893.1916, 49612.45)(894.1947, 15166.77)(895.189, 2594.41)(896.1909, 425.9)(939.1847, 442.81)(940.1873, 171.43)(941.171, 588.3)(942.1549, 245.24)	435860	62463.41	456282.8	53584.43	445469.1	75562.39	444507.8	61298.3	0.14	0.644327	0.733582	-0.34	0.347179	0.652628
COMPOUND_895_553a12_135263	Rp	-	895.5530	12.14				(894.5462, 22440.07)(895.5488, 11389.73)(896.5526, 3764.92)(897.5523, 1014.25)(898.5531, 252.6)	276875	65064.81	307974.8	72464.45	267027.2	53129.91	257246.5	66785.84	-0.28	0.252334	0.348623	-0.44	0.12635	0.423869
COMPOUND_895_5538a12_271391	Rp	-	895.5538	12.27				(894.5467, 43811.0)(895.5497, 21827.82)(896.5534, 6413.34)(897.5567, 1646.05)(898.5634, 443.1)	375929.7	50902.68	375969.7	49352.73	325185.9	36284.19	312153.4	38689.73	-0.90	0.000404	0.00114	-0.31	0.278159	0.585315
COMPOUND_898_1732a9_573771	Rp	-	898.1732	9.57				(897.1666, 5020.6)(898.1702, 1538.96)(899.1703, 453.02)(900.1721, 107.06)	33209.23	4581.528	34135.07	4924.677	33494.21	6557.933	32330.74	4535.858	-0.03	0.914173	0.944366	-0.32	0.376247	0.678626
COMPOUND_899_5665a11_92443	Rp	-	899.5665	11.92				(1012.5221, 452.65)(1013.4799, 711.62)(1014.4615, 712.8)(898.5591, 31560.48)(899.5625, 17280.23)(900.5654, 6019.47)(901.5609, 1534.85)(902.5658, 319.98)	68053.83	27645.48	67449.36	30240.02	158888.5	59810.08	180752.3	76227.6	1.34	1.05E-08	8.64E-08	0.18	0.464327	0.732257
COMPOUND_899_5852a13_039803	Rp	-	899.5852	13.04				(898.5782, 41874.7)(899.5812, 20610.94)(900.5842, 6536.5)(901.5816, 1668.43)(902.5885, 436.88)(903.5704, 90.56)	1559499	254725.4	1729365	261670	1607628	212940.3	1553018	260302.8	0.09	0.731944	0.805246	-0.75	0.01796	0.195392
COMPOUND_903_4644a4_8528066	Rp	+	903.4644	4.85				(463.7295, 936.27)(464.2278, 527.74)(464.7201, 2938.53)(465.22, 1725.26)(921.4823, 71.67)(904.4676, 2539.88)(905.4719, 1283.39)(906.4742, 387.59)(907.4844, 120.7)(452.7395, 85923.87)(453.2409, 45526.49)(453.7419, 13922.13)(454.2424, 3041.78)(454.7438, 634.47)(455.24, 139.59)	189838.4	316024.4	185812.9	237063.5	55037.9	83216.77	54250.82	46149.14	-1.02	4.3E-05	0.000145	0.03	0.899521	0.955487
COMPOUND_904_4797a3_2558017	Rp	+	904.4797	3.26				(472.2208, 390.92)(472.7206, 113.73)(905.4788, 139.37)(906.4928, 98.15)(453.2469, 15486.71)(453.7486, 7784.79)(454.2503, 2361.68)(454.7524, 551.96)(455.2505, 101.12)	#DIV/0!	#DIV/0!	#DIV/0!	#DIV/0!	#DIV/0!	#DIV/0!	#DIV/0!	#DIV/0!	1.27	1.4E-09	1.48E-08	0.00	0.984112	0.992345
COMPOUND_904_48a3_5165477	Rp	+	904.4800	3.52				(472.2245, 404.35)(472.7171, 382.96)(905.4819, 372.21)(906.4873, 203.98)(453.2469, 19354.23)(453.7483, 9874.79)(454.2496, 2565.94)(454.7502, 617.34)(455.2509, 171.83)	20074	8417.811	15210.71	3855.661	58713.91	48802.32	48808.98	31754.25	1.18	2.24E-08	1.7E-07	-0.11	0.631534	0.828008
COMPOUND_905_4823a12_187387	Rp	+	905.4823	12.19				(923.5099, 6233.35)(924.5069, 4207.27)(925.5046, 2259.75)(926.5056, 699.04)(906.4948, 16727.14)(907.4978, 9855.23)(908.4962, 12735.04)(909.4978, 6093.85)(910.4943, 5760.36)(911.497, 3307.46)(912.498, 2046.64)	347077.5	83684.11	286070.6	78830.11	305436.6	75833.92	328772.5	95065.54	-0.55	0.060055	0.103715	0.92	0.009814	0.153413
COMPOUND_905_5132a11_185645	Rp	-	905.5132	11.19				(940.4681, 3910.69)(941.4782, 2404.09)(904.5027, 15973.27)(905.5045, 8846.51)(906.5052, 3656.38)(907.5076, 1419.05)	149502.6	26404.41	155434.8	38777.61	163479.5	55713.52	167096.5	36982.75	-0.51	0.028379	0.053706	-0.47	0.087351	0.350026
COMPOUND_905_5216a10_583235	Rp	-	905.5216	10.58				(904.5161, 9087.81)(905.5196, 4604.88)(906.5327, 13418.11)(907.5365, 7065.51)(908.5481, 9691.09)(909.5515, 4670.76)(910.5513, 4080.46)(950.5337, 3060.92)(951.5389, 2165.07)(952.5387, 2543.29)(953.5384, 1275.72)(954.5193, 1731.32)	166887	80360.92	159711.8	77739.91	60780	45283.8	76360.14	38617.5	-1.38	6.24E-11	1.06E-09	0.01	0.95557	0.980661
COMPOUND_905_6039a12_530823	Rp	-	905.6039	12.53				(940.5953, 370.46)(1018.6027, 504.26)(1019.6084, 295.25)(1020.6168, 412.65)(904.5977, 1900.55)(905.6019, 1052.34)(906.5946, 23870.64)(950.5916, 597.15)(951.5843, 403.79)(952.5644, 455.4)	49634.33	13417.48	55494.27	14289.45	56108	21590.51	50343.32	14602.04	0.52	0.068525	0.116907	-0.90	0.008689	0.14883
COMPOUND_905_6875a10_929913	Rp	-	905.6875	10.93				(1018.6479, 1298.64)(940.6833, 2073.8)(904.6791, 7735.18)(905.678, 4248.78)(906.6767, 1518.37)	30146.6	14786.8	32306.28	20031.42	22973.64	10684.67	30087.87	12489.23	-0.13	0.635845	0.725759	0.32	0.307045	0.613089
COMPOUND_907_5935a12_848889	Rp	-	907.5935	12.85				(1020.5579, 225.99)(1021.5567, 299.4)(906.5866, 6029.31)(907.5896, 3036.67)(908.5886, 1205.16)(909.593, 262.11)	58263.88	13718.23	56352.39	14547.5	46675.17	10261.64	40251.17	11817.44	-0.88	0.000325	0.00093	-0.04	0.895295	0.954308
COMPOUND_907_6489a9_774024	Rp	+	907.6489	9.77				(473.8233, 490.12)(474.3069, 1003.13)(465.8235, 2636.89)(466.3278, 1494.67)(466.8253, 566.0)(463.3445, 38302.29)(463.8461, 19093.35)(464.3473, 6057.7)(464.8486, 1840.21)(465.3444, 508.92)(908.6517, 10704.59)(909.6543, 5236.63)(910.6567, 1764.21)(911.6549, 539.07)(912.661, 119.78)(454.8316, 11584.35)(455.3334, 6573.13)(455.8348, 2031.25)	674462.7	180464.8	563786.3	146638.7	728468.8	111445.9	732059.6	182669.6	0.28	0.325299	0.425667	0.64	0.059131	0.30009
COMPOUND_911_5819a11_052364	Rp	-	911.5819	11.05				(1024.5507, 704.37)(1025.5684, 462.35)(946.5843, 4303.24)(947.5933, 1836.81)(910.5753, 22519.39)(911.5792, 11873.56)(912.5908, 12042.12)(913.5945, 5716.27)(914.5867, 2253.19)(956.5642, 2799.28)(957.5699, 1681.82)	412478.9	154209	453924.4	168347.1	394315	122706.3	465356.8	158563.2	-1.05	8.72E-06	3.52E-05	0.02	0.936812	0.97184
COMPOUND_912_5031a12_096236	Rp	+	912.5031	12.10				(935.4832, 2223.15)(936.4675, 1129.02)(913.513, 14499.73)(914.5135, 8972.76)(915.5114, 3663.37)	122082.7	44645.12	113393	36992.68	102578.2	33991.88	111912.5	46969.2	0.48	0.04758	0.083973	-0.12	0.659868	0.842895

									1st trimester				3rd trimester									
									Control		Pre-eclampsia		Control		Pre-eclampsia		Time			Interaction		
Compound	Column	Ion_mode	Mass	Retention_	Identification	ID_level	MSMS_spectra	CompositeSpectrum	Mean	SD	Mean	SD	Mean	SD	Mean	SD	Value	raw p	FDR_p	Value	raw p	FDR_p
COMPOUND_912_5049a12_247652	Rp	+	912.5049	12.25				(935.5116, 947.07)(936.5111, 567.26)(913.5127, 15671.73)(914.5132, 8686.17)(915.516, 3123.28)	95520.05	28662.73	82724.46	29297.58	81165.69	19949.23	79038.93	25881.16	0.00	0.993205	0.99808	0.57	0.07221	0.325884
COMPOUND_913_5148a12_269906	Rp	-	913.5148	12.27				(1026.503, 679.94)(1027.5068, 4367.13)(948.4898, 4985.22)(912.5063, 13215.81)(913.5086, 7397.49)(914.5113, 3162.36)(915.514, 1058.05)	93472.21	32916.76	93760.43	28319	90736.38	30665.89	79307.68	30318.5	0.17	0.559703	0.656827	-0.47	0.168987	0.48172
COMPOUND_914_1366a9_309307	Rp	-	914.1366	9.31				(913.1279, 1334.18)(914.129, 428.72)(915.1242, 20246.82)(959.1566, 700.39)(960.1395, 261.16)	95853.96	20568.9	104574.5	21877.02	92917.79	20957.06	87190.75	18062.15	-0.17	0.53668	0.635772	-0.65	0.043384	0.270638
COMPOUND_915_529a12_559266	Rp	-	915.5290	12.56				(1028.5164, 889.45)(1029.5219, 2851.55)(1030.5216, 1781.24)(950.4932, 4530.12)(951.4983, 2190.62)(914.5222, 6855.21)(915.5245, 4271.03)(916.5344, 4480.81)(917.5385, 2575.4)(960.5202, 2756.69)(961.5094, 1063.26)(962.4946, 179.44)	287578.5	83001.84	244871.2	87031.05	317357	87277.14	293796.6	77012.99	0.41	0.149455	0.22726	0.18	0.584763	0.798113
COMPOUND_915_5799a11_493208	Rp	-	915.5799	11.49				(914.574, 11941.05)(915.577, 6151.0)(916.5765, 2340.69)(917.5757, 764.68)(918.6014, 161.53)	265355.9	91768.39	321957.4	102218	210488.8	108082.7	223934	112560.6	-1.06	4.89E-07	2.66E-06	-0.48	0.037481	0.259604
COMPOUND_915_6362a12_531209	Rp	-	915.6362	12.53				(1028.6108, 307.85)(1029.6234, 241.75)(950.5921, 739.46)(951.6013, 440.52)(914.6272, 2174.44)(915.6315, 1407.96)(916.6238, 31133.38)(960.6775, 896.6)(961.696, 3766.21)(962.6985, 2199.0)	45578.49	16656.19	54227.46	20027.25	66077.93	26675.58	63177.68	22338.57	0.86	0.002151	0.005305	-0.50	0.121637	0.411918
COMPOUND_916_5338a12_9877825	Rp	+	916.5338	12.99				(939.5324, 2097.15)(940.5283, 1516.15)(917.543, 15312.62)(918.5433, 9280.5)(919.5415, 4046.52)(920.5265, 1455.17)	323387.4	102756.1	301198.4	104226.5	314065.9	88369.9	339743.4	100685.9	0.04	0.883924	0.922855	0.67	0.048737	0.279822
COMPOUND_917_1344a9_309351	Rp	-	917.1344	9.31				(952.1052, 221.19)(953.114, 207.93)(916.1275, 8028.36)(917.1285, 2397.19)(918.1361, 537.37)(919.1581, 155.26)	44334.53	9640.836	48489.04	11451.58	41895.86	8166.263	39814.95	9999.81	-0.25	0.376402	0.477624	-0.55	0.10308	0.379929
COMPOUND_918_6339a12_530645	Rp	-	918.6339	12.53				(917.6265, 17429.23)(918.6293, 5848.5)(919.6328, 1735.7)(920.6414, 294.58)	37210.55	13686.03	44357.2	16821.71	54464.36	21820.69	51431.07	18346.35	0.82	0.003991	0.009208	-0.45	0.168791	0.48172
COMPOUND_919_553a12_113661	Rp	-	919.5530	12.11				(918.5464, 48548.1)(919.5497, 26086.08)(920.5519, 8323.51)(921.5536, 2086.78)(922.5638, 603.9)	306827.2	237822.7	248235.6	217198.4	146494.1	125350.3	152232.6	144208.2	-0.66	0.017017	0.034155	0.44	0.173221	0.487961
COMPOUND_919_5536a12_211285	Rp	-	919.5536	12.21				(918.5464, 48548.1)(919.5497, 26086.08)(920.5519, 8323.51)(921.5536, 2086.78)(922.5638, 603.9)	537185.5	66336.24	548511.6	97302.73	374408.4	130733.8	338079.4	106921.9	-1.25	1.77E-07	1.06E-06	-0.28	0.274269	0.583098
COMPOUND_921_469a11_911196	Rp	-	921.4690	11.91				(1034.4365, 863.53)(1035.4424, 696.56)(1036.446, 1218.64)(920.4588, 5839.12)(921.4634, 2964.73)(922.4619, 3447.23)(923.4651, 1724.66)(924.4603, 1688.38)(925.4696, 1085.09)(926.4778, 1063.37)	92185	20583.55	84465.59	32355.13	111431.5	32223.41	116761.3	32860.89	0.89	8.86E-05	0.000276	0.03	0.916312	0.961472
COMPOUND_921_5693a12_415492	Rp	-	921.5693	12.42				(920.5618, 19618.55)(921.5653, 10859.47)(922.5643, 3443.05)(923.5721, 1051.99)	187459.8	88687.44	160468.1	88547.39	122647.5	82063.37	133953.7	79119.83	-0.50	0.04976	0.087138	0.40	0.177337	0.491826
COMPOUND_921_6658a9_963869	Rp	+	921.6658	9.96				(472.8303, 4124.2)(473.3312, 2785.37)(473.8314, 1014.2)(470.3525, 38680.61)(470.8541, 19269.33)(471.3478, 19453.51)(471.8513, 1979.2)(472.3498, 2579.58)(489.3428, 1104.14)(489.8476, 925.77)(481.3439, 1987.1)(481.8278, 513.62)(922.6677, 13839.85)(923.6704, 6817.66)(924.6696, 2471.99)(925.6705, 735.17)(926.6814, 189.99)(461.8404, 4773.75)(462.3441, 2527.63)(462.8434, 967.04)(463.3285, 91.01)	634286.2	167638	539681.3	109985.3	689690.9	106143.9	693499.1	173598.1	0.26	0.356125	0.458544	0.68	0.044618	0.272788
COMPOUND_923_5829a12_6915455	Rp	-	923.5829	12.69				(922.5774, 15796.63)(923.5811, 8190.74)(924.5823, 2941.8)(925.5771, 728.34)	#DIV/0!	#DIV/0!	#DIV/0!	#DIV/0!	#DIV/0!	#DIV/0!	#DIV/0!	#DIV/0!	0.24	0.360613	0.462735	0.01	0.982715	0.99152
COMPOUND_923_585a12_936366	Rp	-	923.5850	12.94				(922.5781, 20685.83)(923.5816, 11346.83)(924.5837, 3792.66)(925.5841, 1171.09)(926.5622, 224.62)	458105.2	101138.2	447293.8	123031.1	374819.1	86227.32	364483.8	82523.94	-0.69	0.005316	0.011952	-0.07	0.809897	0.919078
COMPOUND_925_7311a10_862317	Rp	-	925.7311	10.86				(924.7239, 5252.72)(925.7261, 3420.68)(926.7277, 1683.01)(927.7277, 639.23)(970.7322, 260.61)(971.7333, 237.99)(972.7098, 886.76)	31370.39	4824.686	33910.09	4786.042	24793.14	2568.129	24952.76	4647.074	-0.34	0.227189	0.321983	-0.14	0.677089	0.851683
COMPOUND_927_575a11_035155	Rp	-	927.5750	11.04				(1040.5254, 1703.56)(1041.5111, 1110.74)(1042.5006, 778.47)(962.5143, 1871.0)(963.4952, 1074.93)(964.4981, 1339.06)(926.569, 21174.84)(927.5725, 10846.02)(928.5834, 12837.66)(929.588, 5823.7)(930.5824, 4035.45)(931.5807, 1808.13)(972.5424, 5037.89)(973.5209, 2361.78)(974.5112, 2575.63)	372178.1	155871.3	329120.6	173366.4	283109	100245.9	307651.7	64130.92	-1.17	2.2E-07	1.28E-06	0.27	0.256034	0.56678
COMPOUND_929_2315a9_578919	Rp	+	929.2315	9.58				(947.2752, 80.09)(930.2399, 5911.61)(931.2431, 1834.93)(932.2489, 295.22)(933.2593, 65.51)	41453.06	13671.97	43615.78	11985.92	48293.43	9163.936	44822.02	11388.78	0.60	0.048369	0.085098	-0.48	0.1807	0.493854
COMPOUND_929_2324a9_597884	Rp	+	929.2324	9.60				(947.2635, 78.78)(930.2389, 5197.16)(931.2429, 1796.66)(932.2451, 383.56)(933.2677, 52.2)	40573.13	14896.16	40097.78	13882.95	50050	10423.63	42534.44	15287.8	0.72	0.018235	0.036439	-0.53	0.140063	0.444883
COMPOUND_930_4808a0_8089563	Hilic	+	930.4808	0.81				(953.5101, 2883.78)(953.9858, 1318.66)(953.4723, 456.26)(954.4648, 375.28)(955.4772, 309.92)(948.51, 3845.94)(949.5035, 2172.32)(950.5138, 2528.69)(931.4971, 3623.49)(466.2479, 5797.99)(466.7499, 2741.59)(467.2509, 1107.98)(467.7556, 329.89)	60203.15	16340.16	62237.38	13009.82	49767.69	22781.42	42683.54	12010.56	0.14	0.617404	0.710109	-0.39	0.248728	0.55846
COMPOUND_933_524a12_4057045	Rp	-	933.5240	12.41				(932.5172, 14085.96)(933.5207, 6955.51)(934.5244, 2634.4)(935.5274, 813.58)	215452.8	54276.35	239324.2	77017.58	201509.9	59015.88	173093.3	39680.45	-0.29	0.27121	0.369265	-0.62	0.050563	0.283798
COMPOUND_935_539a12_808606	Rp	-	935.5390	12.81				(934.532, 15838.18)(935.5357, 7498.59)(936.5381, 3037.75)(937.5391, 923.38)	301652.9	45378.31	326694.1	50651.34	321576.8	55843.14	315877.2	69692.23	0.35	0.24185	0.337243	-0.34	0.349981	0.656798
COMPOUND_935_5503a11_240658	Rp	-	935.5503	11.24				(1048.51, 891.09)(1049.5109, 528.12)(1050.5245, 650.45)(934.5413, 14351.48)(935.5447, 7417.71)(936.5544, 7909.42)(937.559, 3724.35)(938.5695, 3439.36)	273096.1	93313.9	318455.4	164499.7	359771	118448.2	350351.3	147092.4	-0.74	0.001816	0.004534	-0.38	0.167383	0.480454
COMPOUND_935_6762a10_148822	Rp	+	935.6762	10.15				(479.836, 1703.93)(480.3468, 6998.45)(480.8377, 494.48)(477.3599, 14708.01)(477.8613, 7830.78)(478.3546, 1551.19)(478.8449, 520.57)(974.6298, 2461.23)(936.6833, 7362.33)(937.686, 3828.02)(938.6794, 1579.13)(939.6671, 617.15)(940.6708, 73.34)(488.3415, 513.4)(488.8439, 156.25)(489.3492, 2556.53)(468.8471, 5854.11)(469.3489, 3425.15)(469.8495, 1245.09)	382448.6	105051.3	327295.3	69900.7	440015.6	70933.57	436123.1	114222	0.42	0.132057	0.206093	0.60	0.071898	0.325554
COMPOUND_935_7423a13_247795	Rp	+	935.7423	13.25				(958.7715, 1071.4)(959.7628, 797.34)(960.7558, 1207.78)(961.7534, 723.41)(953.748, 480.37)(954.7672, 575.81)(955.7777, 453.91)(936.751, 32641.45)(937.7537, 19773.7)(938.7563, 7209.69)(939.7464, 2235.91)(940.7419, 644.2)	96519.1	62693.42	104134.9	91949.35	52164	17472.61	28528.38	14994.02	-1.28	9.85E-10	1.09E-08	-0.15	0.490322	0.748114
COMPOUND_936_5026a12_179933	Rp	+	936.5026	12.18				(959.4828, 1701.01)(960.4778, 1315.33)(975.4839, 2350.42)(937.5126, 17159.91)(938.5157, 9598.12)(939.5164, 3640.06)(940.5133, 1411.63)	127808.9	52798.59	106894.7	56532.95	120220.2	25352.81	135961	51786.75	-0.15	0.597012	0.691669	0.37	0.270446	0.57949
COMPOUND_937_6605a9_559114	Rp	+	937.6605	9.56				(499.8081, 75.83)(500.3052, 4759.49)(500.7982, 128.25)(480.8275, 2472.23)(481.3344, 2264.4)(481.8317, 713.62)(482.3309, 283.24)(478.35, 21293.18)(478.8512, 10997.86)(479.3528, 3644.33)(479.8518, 1126.25)(480.3477, 199.0)(938.663, 4753.09)(939.6649, 2531.03)(940.6671, 890.45)(941.6731, 299.23)(507.792, 220.53)(508.3047, 102.86)(469.8366, 8728.23)(470.3387, 4890.52)(470.8408, 1785.28)	413760.3	110504.3	355619.5	70839.87	438953.7	71157.87	455624.9	103904.5	0.18	0.519089	0.619167	0.79	0.020144	0.199116
COMPOUND_941_4652a4_883823	Rp	+	941.4652	4.88				(964.4519, 85.85)(965.4321, 43.44)(966.4324, 215.12)(942.4731, 474.52)(942.9925, 40.87)(943.4767, 196.61)(943.9956, 208.69)(944.4874, 129.95)(501.6987, 416.86)(502.1896, 133.5)(502.6965, 409.39)(961.9465, 96.36)(962.4526, 61.34)(490.714, 4800.06)(491.2138, 2483.73)(491.7168, 788.75)(492.2101, 161.66)(493.7202, 234.84)(494.2254, 114.33)(494.7059, 397.63)(482.7366, 261.66)(483.2311, 36.51)(483.7176, 830.15)(484.2308, 630.23)(484.7323, 172.62)(959.5191, 104.24)(980.4167, 234.46)(942.4678, 7976.82)(943.4719, 4545.23)(944.4729, 1300.92)(945.4837, 498.68)(946.4813, 81.66)(509.6862, 511.41)(510.1871, 293.35)(510.687, 375.16)(471.7397, 146815.16)(472.2411, 78058.12)(472.7423, 23740.98)(473.2429, 5491.58)(473.7474, 1317.07)(474.248, 279.15)	269278.1	309211.2	444870	501378.7	311868.1	488774.7	152380.8	251345.3	-0.46	0.064899	0.111567	-0.67	0.025028	0.219303
COMPOUND_941_5448a12_252949	Rp	-	941.5448	12.25				(940.5393, 5621.23)(941.5409, 3703.0)(942.5406, 2092.94)(943.5401, 729.2)	28403	8095.023	29130.27	4905.244	24390.5	5255.374	22729.59	4834.905	-0.21	0.46989	0.573399	-0.16	0.650079	0.838541
COMPOUND_947_5201a11_449079	Rp	-	947.5201	11.45				(1060.5028, 1627.31)(1061.5092, 2084.16)(1062.5007, 1846.03)(946.5189, 8282.37)(947.521, 4468.37)(948.5253, 14745.98)(949.5275, 8983.41)(950.5285, 4106.38)(951.5229, 1381.45)(992.5258, 6243.27)(993.5249, 8923.11)(994.5219, 6702.69)(995.5232, 2908.55)	274700.1	131925	329890	198031.6	211092	62817.95	161449	44342.55	-1.17	1.2E-08	9.65E-08	-0.30	0.15339	0.465803
COMPOUND_947_5843a12_83804	Rp	-	947.5843	12.84				(946.5769, 8343.88)(947.5801, 4589.52)(948.585, 2522.69)(949.5883, 908.55)	178220	44436.2	186027.3	50206.51	178122.4	51089.16	164746.4	53599.63	-0.14	0.619708	0.712032	-0.28	0.392988	0.690408

									1st trimester				3rd trimester									
									Control		Pre-eclampsia		Control		Pre-eclampsia		Time			Interaction		
Compound	Column	Ion_mode	Mass	Retention_	Identification	ID_level	MSMS_spectra	CompositeSpectrum	Mean	SD	Mean	SD	Mean	SD	Mean	SD	Value	raw p	FDR_p	Value	raw p	FDR_p
COMPOUND_948_6168a10_193423	Rp	-	948.6168	10.19				(947.6099, 74510.8)(948.6129, 36445.68)(949.6159, 11252.29)(950.6185, 2696.49)(951.6257, 595.03)	72221.19	77375.15	94174.89	108399.1	80019.07	123368.6	104039.8	115789.7	-0.59	0.051469	0.089781	0.26	0.461146	0.729797
COMPOUND_948_617a10_273766	Rp	-	948.6170	10.27				(947.6101, 60998.27)(948.6132, 29220.2)(949.6161, 9226.11)(950.619, 2023.71)(951.6245, 439.88)	186228.8	36448.33	204273.1	61762.77	184256.9	86605.8	188251.7	78597.97	-0.45	0.133459	0.207993	0.03	0.93651	0.97184
COMPOUND_948_6197a10_273863	Rp	+	948.6197	10.27				(971.6075, 6351.67)(972.6099, 3367.35)(973.6173, 1361.83)(974.6216, 412.43)(987.5647, 1098.46)(988.5488, 958.66)(989.5404, 1179.04)(949.6267, 42760.6)(950.63, 20649.35)(951.6346, 6248.35)(952.638, 1611.97)(953.6432, 447.77)	98104.06	22124.84	104185.7	34578.31	85337.93	49485.49	87204.81	39471.06	-0.69	0.021135	0.041425	0.09	0.785857	0.909997
COMPOUND_949_498a12_408281	Rp	-	949.4980	12.41				(1062.4736, 761.1)(1063.4758, 547.18)(1064.4745, 576.11)(948.4904, 11262.56)(949.4931, 6076.05)(950.4911, 6508.06)(951.4955, 3017.78)(952.4895, 2720.6)(953.4924, 1307.56)(954.4983, 1066.25)(994.5172, 548.92)(995.5005, 532.43)	192432.3	37813.91	200304.9	49616.88	181653.2	35456.06	171007.3	38699.95	-0.20	0.482654	0.58406	-0.46	0.17283	0.487735
COMPOUND_949_5325a11_446433	Rp	-	949.5325	11.45				(984.5247, 2840.94)(985.5014, 909.1)(948.5253, 14817.97)(949.5274, 9068.72)(950.5279, 4154.48)(951.5231, 1429.86)	88664.94	30090.41	92481.3	45062.05	72192.5	5714.13	49006.14	9946.424	-1.26	6.37E-10	7.52E-09	-0.22	0.278362	0.585315
COMPOUND_949_6942a10_31658	Rp	+	949.6942	10.32				(980.6749, 358.15)(981.1743, 302.56)(988.5789, 517.03)(989.0996, 289.2)(486.842, 1012.13)(487.3353, 1836.34)(487.8362, 173.76)(967.6841, 281.46)(503.3411, 2521.28)(503.8581, 1348.53)(484.3675, 9150.38)(484.8698, 4691.55)(485.3587, 8632.67)(485.8464, 564.89)(486.3543, 1008.47)(950.6983, 3151.34)(951.7013, 1872.04)(952.6934, 692.17)(953.7071, 226.51)(495.3599, 5659.01)(495.8675, 1193.68)(475.8545, 2634.92)(476.3555, 1752.86)(476.8585, 490.87)	171722	54951.78	144578.6	43142.57	234727.5	50588.97	253043.5	78766.69	0.65	0.011667	0.024261	0.65	0.03481	0.249266
COMPOUND_951_6769a9_75777	Rp	+	951.6769	9.76				(487.8356, 2843.7)(488.3382, 1656.35)(488.838, 662.78)(489.3264, 252.56)(485.359, 21907.79)(485.8608, 11792.12)(486.361, 4136.36)(486.8607, 1250.77)(487.3589, 347.35)(504.3484, 788.27)(504.8565, 610.24)(952.6783, 7984.13)(953.6807, 4210.1)(954.6834, 1353.32)(955.6859, 416.91)(956.684, 45.31)(476.845, 12712.91)(477.3468, 7754.02)(477.8486, 2897.43)	681616.3	176561.8	588766.9	120576.1	744510.4	115321.5	737630.4	188774.3	0.30	0.279853	0.379286	0.58	0.088803	0.352184
COMPOUND_957_5185a12_268805	Rp	-	957.5185	12.27				(994.4793, 420.07)(956.5153, 13866.8)(957.5179, 7210.72)(958.5118, 2932.66)(959.4934, 631.76)	80764.15	16872.88	81237.41	11446.5	66630.23	11922.39	62790.54	11279.76	-0.74	0.004029	0.009288	-0.34	0.255985	0.56678
COMPOUND_957_5199a12_141909	Rp	-	957.5199	12.14				(992.4856, 905.03)(993.4795, 825.06)(994.4757, 1291.74)(956.5142, 12237.02)(957.5176, 6414.78)(958.51, 2582.38)(959.518, 582.54)	60482.7	15647.6	66436.03	17706.07	63902	11660.07	65788.82	18777.58	-0.27	0.296604	0.396646	0.13	0.668885	0.846185
COMPOUND_958_2103a9_640239	Rp	-	958.2103	9.64				(1071.1797, 1290.9)(957.2039, 35559.72)(958.2075, 12148.84)(959.1922, 2881.62)(960.2099, 458.51)(1003.2007, 379.63)(1004.1908, 141.05)(1005.182, 438.0)(1006.1899, 94.4)(1007.1644, 1071.08)(1008.1393, 352.26)(1009.1637, 663.33)	190114.4	45561.55	203671	52315.32	176522.1	43242.93	196151.3	75019.23	-0.23	0.44609	0.551672	-0.02	0.957643	0.980661
COMPOUND_958_2109a9_585631	Rp	-	958.2109	9.59				(1071.1779, 1229.26)(957.2039, 44596.16)(958.2071, 14620.02)(959.2034, 2771.07)(960.2135, 537.81)(1003.1972, 476.18)(1004.1885, 277.61)(1005.1855, 505.33)(1006.182, 233.68)(1007.1661, 1593.56)(1008.1556, 592.5)(1009.1646, 1258.06)	149026	43705.1	158872.5	30471.39	130216.7	25633.01	145712.9	29560.98	-0.60	0.04241	0.075799	0.20	0.554675	0.781554
COMPOUND_961_515a12_025444	Rp	-	961.5150	12.03				(1074.4583, 961.49)(1075.4469, 487.92)(996.4867, 2774.78)(997.4949, 2613.92)(998.4924, 3641.15)(999.4962, 2233.47)(1000.4969, 1538.06)(960.5167, 5874.93)(961.5181, 3461.45)(962.5261, 2135.19)(1006.498, 1615.15)(1007.5005, 1103.48)(1008.4944, 1240.12)	90414.26	31597.79	100574.5	36846.67	78579.6	21151.13	120176.4	62281.03	-0.82	0.001507	0.003801	0.22	0.448171	0.721588
COMPOUND_961_5525a13_040233	Rp	-	961.5525	13.04				(1074.5222, 789.97)(960.547, 13321.85)(961.5506, 7015.57)(962.5464, 2735.73)(963.544, 834.81)	453071	76115.21	497721.5	78049.46	464500	55643.14	446483.9	71415.56	0.00	0.999965	0.999965	-0.70	0.022517	0.213113
COMPOUND_962_6334a1_1547581	Hilic	+	962.6334	1.15	no msms			(985.6299, 711.89)(986.6484, 284.9)(987.6425, 5300.76)(988.6459, 2995.08)(963.6422, 9920.42)(964.6447, 5338.22)(965.6485, 1797.7)(966.6528, 493.62)	16741.63	8005.605	24460.07	13148.19	19631.79	7412.439	16006.16	7785.645	0.38	0.168697	0.252094	-1.23	0.00029	0.024157
COMPOUND_963_774a13_243681	Rp	+	963.7740	13.24				(986.7711, 969.43)(987.7756, 685.69)(988.7812, 1073.02)(964.7812, 26109.66)(965.784, 16128.44)(966.7869, 5767.14)(967.7876, 1473.23)(968.7759, 380.48)(482.8858, 169.69)(483.3866, 100.6)	73018.91	53671.27	75473.38	62277.13	41779.67	15673.42	26698	9084.847	-1.13	1.8E-07	1.07E-06	-0.11	0.62724	0.825147
COMPOUND_965_69a9_930252	Rp	+	965.6900	9.93				(502.8282, 489.1)(503.3221, 345.66)(494.844, 5291.34)(495.3316, 20008.38)(492.3664, 24970.55)(492.8685, 13063.78)(493.369, 4560.55)(493.8711, 1458.39)(1004.6671, 657.79)(511.3514, 676.23)(511.8584, 415.95)(512.3364, 8213.24)(966.6933, 9848.48)(967.6965, 5213.08)(968.6972, 1876.3)(969.6978, 567.17)(970.7029, 151.12)(503.3442, 395.72)(503.8565, 195.94)(483.8523, 15103.78)(484.3529, 9374.71)(484.8563, 3064.89)	1227567	243750.1	1151574	289225.5	1160777	183496.1	1138928	283432.3	-0.33	0.231905	0.326815	0.22	0.498686	0.753329
COMPOUND_968_5249a12_560808	Rp	+	968.5249	12.56				(991.5037, 1681.39)(992.4979, 1247.33)(993.5196, 1474.4)(994.5123, 934.46)(995.4906, 1153.22)(996.4653, 655.17)(997.486, 1890.09)(1007.5061, 1821.04)(1008.5023, 1241.37)(1009.5206, 2311.9)(969.533, 5458.99)(970.5339, 3174.5)(971.5162, 2090.22)(972.4935, 672.19)	133221.6	53793.31	100672.5	38046.45	146654.6	57858.17	124716.1	51625.68	-0.02	0.943361	0.963469	0.54	0.119695	0.409655
COMPOUND_972_6176a10_133184	Rp	-	972.6176	10.13				(1085.5842, 266.06)(1086.5778, 126.99)(1087.5944, 1650.32)(971.6101, 10112.13)(972.6132, 5421.9)(973.6157, 1756.66)(974.6155, 497.29)(975.6203, 66.19)	#DIV/0!	#DIV/0!	#DIV/0!	#DIV/0!	#DIV/0!	#DIV/0!	#DIV/0!	#DIV/0!	-0.51	0.082069	0.136701	0.00	0.994829	0.998139
COMPOUND_972_6185a10_130318	Rp	+	972.6185	10.13				(995.6088, 2566.88)(996.6121, 1333.3)(997.6271, 24282.12)(1011.5508, 1011.54)(1012.5532, 899.63)(990.6739, 865.55)(973.6263, 11929.35)(974.6292, 6216.96)(975.6357, 2104.17)(976.6161, 152.37)(487.3362, 685.01)(487.8297, 135.53)	63169	26969	66021.75	29854.9	39708.54	15520.83	44492.33	18175.64	-0.89	0.001746	0.004388	0.10	0.755277	0.895058
COMPOUND_973_4935a12_144854	Rp	-	973.4935	12.14				(1086.4645, 1030.96)(1087.4666, 622.5)(1088.4569, 563.9)(972.4895, 11185.91)(973.4922, 5810.42)(974.4912, 6492.58)(975.497, 3237.81)(976.4916, 2741.19)	87691.7	20533.89	94441.88	26795.85	89971.46	25272.57	85122.89	27489.2	-0.01	0.984679	0.992613	-0.15	0.641149	0.834103
COMPOUND_973_4963a12_2684145	Rp	-	973.4963	12.27				(1086.4706, 844.44)(1087.4647, 507.43)(972.4898, 11957.61)(973.4927, 6569.45)(974.4905, 7159.16)(975.4955, 3598.87)(976.4926, 2967.77)(977.496, 1573.68)(978.4924, 1159.88)	93688.49	11607.52	89383.48	12586.52	81559.36	15227.57	79206.02	12888.74	-0.83	0.002976	0.0071	0.09	0.770256	0.902789
COMPOUND_975_5116a12_538409	Rp	-	975.5116	12.54				(1088.4822, 1141.99)(1089.4839, 826.13)(974.5055, 11379.93)(975.5082, 6647.8)(976.5069, 6924.15)(977.5097, 3497.75)(978.5056, 3194.21)(1020.4907, 531.81)(1021.4927, 408.88)(1022.502, 572.66)(1023.5051, 498.04)(1024.4974, 997.12)	225510.6	119836.5	200343.2	116223.3	246532.1	142001.9	298781.5	119533.8	0.38	0.151849	0.230589	0.49	0.112356	0.398247
COMPOUND_977_5268a13_042122	Rp	-	977.5268	13.04				(1090.4998, 1286.27)(1091.5015, 877.29)(1092.4934, 692.13)(976.5203, 10288.67)(977.5237, 5755.41)(978.5222, 5857.88)(979.5235, 2999.63)(980.5207, 2797.49)(981.5237, 2641.25)(982.5283, 1948.43)	425072.5	56227.27	431974.2	65803.31	437115.9	44215.55	413829.8	64259.78	0.23	0.446936	0.552415	-0.53	0.14235	0.448182
COMPOUND_977_67a1_217333	Hilic	+	977.6700	1.22				(1000.6696, 2269.68)(1001.6826, 2291.65)(1002.6857, 1266.82)(978.6821, 9137.06)(979.6825, 2997.54)(980.6914, 792.39)(981.6789, 131.0)	43085.82	13453.8	55019	16431.84	24564.5	5193.699	24297.75	4363.578	-0.75	0.000486	0.001358	-0.41	0.100853	0.374169
COMPOUND_979_7052a10_132949	Rp	+	979.7052	10.13				(501.8517, 3490.67)(502.3648, 3636.72)(502.8543, 938.46)(997.7876, 201.17)(499.3728, 32162.57)(499.876, 16325.82)(500.374, 6979.08)(500.8735, 2014.94)(501.3755, 414.13)(980.7093, 6182.65)(981.7118, 3459.8)(982.7126, 1207.89)(983.7108, 452.77)(984.7254, 85.96)(490.8605, 10321.18)(491.3615, 7455.28)(491.8657, 2512.41)	511539.7	131547.6	440327.8	83756.14	573746.8	88169.4	573369.3	137628.9	0.36	0.193184	0.282004	0.67	0.045521	0.273371
COMPOUND_980_1447a9_558185	Rp	-	980.1447	9.56				(1015.1276, 441.03)(1016.1222, 208.28)(979.1374, 30286.7)(980.1401, 12501.75)(981.1398, 3571.29)(982.1427, 720.39)(983.1639, 121.79)	198000.4	115097.9	176173.7	77356.85	179701.2	100566.6	191747.1	120977.6	-0.03	0.929663	0.955546	-0.19	0.575522	0.790773

									1st trimester				3rd trimester									
									Control		Pre-eclampsia		Control		Pre-eclampsia		Time			Interaction		
Compound	Column	Ion_mode	Mass	Retention_	Identification	ID_level	MSMS_spectra	CompositeSpectrum	Mean	SD	Mean	SD	Mean	SD	Mean	SD	Value	raw p	FDR_p	Value	raw p	FDR_p
COMPOUND_980_5134a12_426218	Rp	-	980.5134	12.43				(1093.4685, 440.79)(1094.4702, 837.1)(1095.4792, 653.4)(1096.487, 377.11)(1097.502, 383.56)(979.5075, 7223.02)(980.5095, 4626.61)(981.5096, 2289.07)(1025.5128, 1061.92)(1026.5155, 2247.66)	42435.54	17030.72	34717.52	19483.95	65290	45711.7	53616.39	28337.04	0.02	0.931047	0.956096	0.67	0.032896	0.244873
COMPOUND_980_6074a9_766103	Rp	-	980.6074	9.77				(979.6002, 19190.13)(980.6025, 9544.59)(981.6063, 3191.99)(982.6105, 744.56)(983.6146, 173.79)	23351.58	12522.32	33295.58	24921.96	19456.75	11214.52	20346.55	8983.811	0.01	0.951878	0.96951	-0.73	0.009286	0.153413
COMPOUND_990_3552a9_064262	Rp	-	990.3552	9.06				(1103.3616, 731.52)(1025.3315, 762.57)(1026.3243, 464.83)(989.3482, 13401.98)(990.3516, 5793.31)(991.3623, 10019.32)(992.3654, 4121.26)(993.3669, 1519.8)(994.3635, 487.45)	17389	6428.867	19196.31	4826.99	120085.6	79817.11	65442.2	50302.01	1.81	3.16E-15	7.92E-13	-0.65	0.002025	0.065137
COMPOUND_990_5354a12_2677145	Rp	-	990.5354	12.27				(1025.5077, 1826.55)(1026.498, 986.43)(989.5286, 6364.43)(990.5312, 3886.56)(991.5325, 1517.74)(992.538, 452.06)(993.5475, 105.5)	37024.04	4938.511	34931.39	8798.07	32581.36	7195.816	32806.68	8039.762	-0.67	0.024052	0.046353	0.44	0.214873	0.521996
COMPOUND_995_7027a9_744923	Rp	+	995.7027	9.74				(509.8495, 3718.13)(510.3583, 3981.75)(510.8522, 889.55)(511.3458, 272.11)(526.3597, 667.86)(526.8662, 576.07)(1034.6754, 101.1)(996.7044, 4466.34)(997.7071, 2585.8)(998.7095, 888.78)(999.7108, 272.91)(498.8582, 12416.31)(499.3602, 7517.24)(499.862, 2735.28)	723248.6	195329.9	626149.5	139798.8	779848	134845.1	779017.5	194616.8	0.23	0.408696	0.512463	0.61	0.075258	0.329961
COMPOUND_996_6177a10_121729	Rp	+	996.6177	10.12				(1019.6216, 3868.1)(1020.617, 2505.17)(1021.6256, 12277.01)(1035.6207, 1479.9)(1036.619, 1025.69)(997.6271, 24282.12)(998.6297, 13915.19)(999.6346, 4270.97)(1000.634, 1146.46)	116784.9	39260.91	124821.4	41009.2	58848	22639.24	67427.39	29168.44	-1.28	1.13E-06	5.53E-06	0.09	0.739998	0.888787
COMPOUND_996_7274a10_134578	Rp	+	996.7274	10.13				(1019.6891, 436.22)(1020.2083, 217.8)(1017.2995, 168.88)(1017.789, 157.67)(1014.8, 150.07)(997.6897, 183.2)(998.6761, 148.21)(499.3728, 32162.57)(499.876, 16325.82)(500.374, 6979.08)(500.8735, 2014.94)(501.3755, 414.13)	341201.1	105446.6	272741.9	69657.62	364529.2	65424.96	374743.9	99540.63	0.52	0.051093	0.089195	0.43	0.178128	0.492203
COMPOUND_997_4946a12_208357	Rp	-	997.4946	12.21				(1110.4624, 1132.24)(1111.4587, 785.1)(996.4891, 13036.41)(997.4928, 7150.63)(998.491, 7765.71)(999.4959, 4027.41)(1000.4914, 3253.93)(1001.4939, 1858.11)(1002.4951, 1281.28)	147499.6	29836.89	141326.8	24456.51	139776.9	49869.14	122253.6	63849.39	-0.41	0.155839	0.235695	-0.12	0.720402	0.876947
COMPOUND_998_6333a10_2881	Rp	-	998.6333	10.29				(997.6259, 9826.39)(998.6297, 5197.04)(999.6337, 1841.88)(1000.6354, 647.43)(1001.6572, 164.79)	19955.64	6587.849	20658	8709.31	18611.08	5362.498	22597.6	8546.567	-0.35	0.231771	0.326815	0.54	0.11901	0.408824
COMPOUND_999_5143a12_421767	Rp	-	999.5143	12.42				(998.5038, 5490.17)(999.5084, 3441.4)(1000.506, 4855.77)(1001.5127, 2771.68)(1002.5084, 1904.36)(1003.5109, 1858.33)(1004.5047, 1262.98)	46919.4	15904.9	39839.58	13346.92	40256.7	17190.28	57395.23	17461.78	-0.25	0.364003	0.466024	0.73	0.027757	0.227301
COMPOUND_1000_6486a10_379304	Rp	-	1000.6486	10.38				(999.6412, 51982.82)(1000.6445, 27133.7)(1001.6473, 9000.53)(1002.6509, 2044.62)(1003.6519, 525.74)(1004.6479, 123.74)	166473.8	60489.2	166963.8	50853.35	128556.1	38391.22	148647.8	52460.12	-0.72	0.015992	0.032444	0.36	0.307089	0.613089
COMPOUND_1000_6509a10_3721895	Rp	+	1000.6509	10.37				(1023.638, 6253.57)(1024.638, 4109.68)(1025.6373, 1952.32)(1026.6534, 666.19)(1039.6033, 115.87)(1040.5927, 4372.56)(1001.6566, 9898.21)(1002.6596, 4896.66)(1003.6669, 1781.8)(1004.6806, 671.11)(1005.6681, 176.92)	76298.04	26414.45	76395.48	27243.1	39634.15	15432.81	51399.94	22118.96	-1.13	8.56E-05	0.000267	0.42	0.188073	0.504013
COMPOUND_1004_6794a10_647412	Rp	-	1004.6794	10.65				(1003.6723, 41139.26)(1004.6757, 22964.71)(1005.6783, 7804.82)(1006.6793, 1817.54)(1007.6905, 517.35)	139241.6	29490.76	147706.4	34557.15	76033.79	36802.44	79997.88	31468.18	-1.35	5.8E-08	3.9E-07	-0.12	0.637282	0.831083
COMPOUND_1009_7204a9_927808	Rp	+	1009.7204	9.93				(1019.1796, 101.34)(1019.6879, 141.55)(524.8574, 265.63)(525.3698, 1911.16)(525.8648, 121.4)(526.3668, 1417.6)(1038.2471, 180.25)(1038.7307, 163.4)(1039.2444, 113.16)(516.8569, 5475.77)(517.3571, 3617.61)(517.86, 1192.81)(533.3692, 1361.74)(533.8734, 735.55)(514.3788, 43606.86)(514.8804, 23998.54)(515.3767, 12924.97)(515.8816, 2410.32)(516.3756, 782.85)(525.3712, 1903.09)(525.8571, 466.82)(526.3581, 1367.57)(1010.7196, 4550.29)(1011.7223, 2659.82)(1012.7228, 944.78)(1013.7235, 335.92)(1014.7082, 72.47)(505.8657, 15683.75)(506.368, 8917.06)(506.8691, 3174.5)	787687.6	212104.2	679467.6	148957.2	855091.3	144107.5	872955.7	223716.7	0.23	0.404854	0.509063	0.69	0.044246	0.272788
COMPOUND_1014_6712a10_116928	Rp	+	1014.6712	10.12				(1037.6547, 9672.28)(1038.6573, 7175.09)(1039.6749, 563440.56)(530.3097, 3882.91)(530.7972, 1186.35)(531.3245, 3353.02)(1032.696, 93.27)(1033.7023, 252.27)(1053.6226, 2136.9)(1015.6738, 73053.36)(1016.6767, 37753.26)(1017.6779, 11807.49)(1018.6598, 4870.87)(1019.6701, 420.76)(508.3388, 12813.95)(508.8311, 1120.15)(509.3449, 3612.88)(509.8477, 738.9)(510.36, 3026.26)	1886662	886419.8	2053240	846065.9	611636.1	274983.5	639546.1	377686.9	-1.38	8.71E-10	9.81E-09	-0.18	0.410757	0.694731
COMPOUND_1016_6807a10_3650675	Rp	+	1016.6807	10.37				(1039.6683, 5456.83)(1040.6716, 3140.27)(1041.6761, 5915.1)(1042.6818, 4384.6)(1034.6987, 1179.54)(1035.688, 706.77)(1017.6903, 46877.29)(1018.6935, 25412.14)(1019.6953, 8583.36)(1020.6992, 2146.06)(1021.6894, 601.0)(509.3497, 3183.05)(509.8501, 1568.43)	226461	61637.27	242495	62157.08	63317.25	32060.99	80562.21	37163.19	-1.24	4.32E-08	3.02E-07	-0.14	0.545976	0.77815
COMPOUND_1020_6175a10_113955	Rp	-	1020.6175	10.11				(1019.6102, 40933.0)(1020.6133, 22641.46)(1021.6165, 7721.65)(1022.6225, 1889.1)	114153.8	33642.56	113552.8	33014.26	73848.86	24843.94	86616.58	32801.14	-1.21	3.56E-05	0.000124	0.41	0.215103	0.521996
COMPOUND_1020_6177a10_137846	Rp	+	1020.6177	10.14				(1043.6053, 1525.32)(1044.586, 17486.55)(1032.6365, 1031.69)(1033.1246, 995.04)(1033.6216, 910.5)(1034.1287, 715.57)(533.3103, 392.91)(533.8219, 120.93)(534.296, 9060.22)(1059.5961, 1355.85)(1038.6573, 8735.42)(1021.6273, 16253.2)(1022.6327, 10728.72)(1023.6399, 4271.8)(1024.638, 1681.64)(511.3123, 2625.2)(511.8158, 1065.26)(512.313, 338.57)	222510.1	33007.31	217314.2	36671.38	133872.9	34121.91	143045.1	48491.95	-1.64	3.23E-11	6.39E-10	0.45	0.056586	0.295663
COMPOUND_1022_2229a9_673863	Rp	-	1022.2229	9.67				(1021.2161, 23571.47)(1022.2195, 7992.21)(1023.2077, 1856.88)(1024.2231, 280.65)(1067.2083, 436.9)(1068.2094, 200.2)(1069.1823, 482.92)	230566.5	33960.02	240353.7	38009.14	213537.1	43803.94	233723.2	34529.05	-0.39	0.188062	0.276446	0.19	0.586233	0.79915
COMPOUND_1023_7316a10_095456	Rp	+	1023.7316	10.10				(1043.6929, 223.38)(1044.2104, 140.46)(1044.7086, 59.43)(523.8644, 3476.63)(524.3733, 12819.21)(524.8648, 831.67)(540.3781, 1418.58)(540.882, 837.83)(541.3714, 1085.62)(541.8755, 235.45)(1024.7358, 6759.5)(1025.7382, 3800.85)(1026.7384, 1488.67)(1027.7363, 357.51)(512.8736, 11037.23)(513.3737, 6295.56)(513.8784, 2467.03)	226596.6	74146.67	196918.4	51565	286446	35260.9	297558.6	70624.63	0.73	0.006568	0.014514	0.39	0.217347	0.524815
COMPOUND_1024_7574a10_434175	Rp	+	1024.7574	10.43				(1025.726, 196.0)(513.3885, 7839.62)(513.8909, 4133.34)(514.3775, 4867.84)(514.8772, 911.57)(515.3693, 1307.36)	109335.6	37043.36	97929.59	29466.19	130585.5	39092.4	124431.2	34074.59	-0.97	0.000214	0.000631	0.57	0.056174	0.29488
COMPOUND_1026_5424a10_198213	Rp	+	1026.5424	10.20				(1049.5684, 922.38)(1038.6113, 825.49)(1039.0958, 321.55)(1057.4962, 642.8)(1057.975, 42.34)(1027.5498, 1863.33)(1028.0491, 229.04)(1028.5566, 1032.36)(1029.0496, 95.85)(1029.5435, 775.5)(1030.0402, 91.72)(1030.5457, 297.19)(1036.1531, 1402.89)(1036.6462, 1820.74)(1037.1324, 478.84)(1055.0695, 453.07)(1055.5464, 389.98)(1065.4843, 265.25)(1066.4792, 144.93)(1044.5853, 12106.71)(1045.602, 11369.0)(1046.6094, 5616.31)(1047.611, 1779.69)(1048.6096, 558.62)(1027.5522, 2187.47)(1028.5583, 1140.38)(1029.5465, 846.36)(1030.5458, 341.93)(1031.5629, 43.92)(514.2775, 2163.95)(514.7788, 1036.77)(515.2798, 276.35)(515.7751, 99.56)	130272.8	63725.45	126359	81598.11	173780.4	103586.2	212756.7	120440.1	0.26	0.342945	0.443857	0.26	0.411224	0.694731
COMPOUND_1026_6403a10_160824	Rp	-	1026.6403	10.16				(1025.6335, 16209.99)(1026.6367, 8679.38)(1027.6333, 7517.2)(1028.6345, 3206.82)(1029.6372, 1009.49)(1030.6361, 198.24)	87532.68	46265.52	80482.8	38490.45	60747.29	46984.79	60604.18	46950.38	-1.15	1.88E-05	7.07E-05	-0.02	0.954304	0.980661
COMPOUND_1030_696a10_383612	Rp	+	1030.6960	10.38				(1053.6836, 2616.91)(1031.7035, 9476.59)(1032.7056, 5526.6)(1033.7065, 2213.97)(1034.6927, 867.64)(1035.7133, 103.43)(516.3585, 2618.07)(516.8548, 291.45)(517.3615, 890.35)	80799.43	26145.52	83833.11	31992.67	26451.5	10075.06	25310.28	9470.864	-1.42	2.85E-12	9.15E-11	-0.16	0.393714	0.690408
COMPOUND_1030_7301a10_632225	Rp	+	1030.7301	10.63				(1053.7043, 4520.94)(1054.7091, 2394.14)(1055.7194, 1149.83)(1056.7318, 377.0)(1031.7383, 15154.26)(1032.7417, 8546.65)(1033.7406, 3678.15)(1034.7448, 1070.02)(1035.7363, 265.66)	52112.04	19277.08	56185.02	20190.44	23958	6007.281	17763.07	6641.928	-1.45	1.08E-12	3.24E-12	-0.12	0.522902	0.522902
COMPOUND_1037_742a10_255291	Rp	+	1037.7420	10.26				(530.8735, 2444.79)(531.3713, 2082.63)(531.8714, 712.05)(528.3941, 20638.12)(528.8957, 12373.01)(529.3878, 10171.7)(529.8912, 1469.88)(1076.6724, 864.37)(1077.6757, 460.44)(1038.7502, 3503.48)(1039.7533, 2209.78)(1040.7507, 945.92)(1041.7561, 77.9)(519.8814, 8412.21)(520.381, 1643.57)(520.8846, 1694.09)	145106.2	51058.61	124162	38495.72	289198.8	72237.51	288046.5	101976	1.01	5.09E-05	0.000169	0.44	0.117873	0.408047

									1st trimester				3rd trimester									
									Control		Pre-eclampsia		Control		Pre-eclampsia		Time			Interaction		
Compound	Column	Ion_mode	Mass	Retention_	Identification	ID_level	MSMS_spectra	CompositeSpectrum	Mean	SD	Mean	SD	Mean	SD	Mean	SD	Value	raw p	FDR_p	Value	raw p	FDR_p
COMPOUND_1042_6993a10_368553	Rp	+	1042.6993	10.37				(1065.6876, 47545.77)(1066.6909, 27842.24)(1067.6976, 29238.47)(1068.7, 13694.47)(1069.6885, 6023.95)(1043.7069, 451348.6)(1044.7112, 250218.55)(1045.7129, 81457.39)(1046.7144, 18016.57)(1047.7147, 3975.75)(1048.7047, 1014.55)	2060465	694679.8	2086819	777796.3	486975.2	247440.9	623123	368265.9	-1.72	2.5E-14	3.75E-12	0.18	0.367028	0.669574
COMPOUND_1043_5793a10_248927	Rp	+	1043.5793	10.25				(1061.6074, 1342.32)(1062.5963, 1083.6)(1044.5854, 70259.16)(1045.5887, 40444.18)(1046.5914, 14053.05)	493434.5	96858	436981.9	89360.55	542759.5	74748.01	549982.5	80941.06	0.51	0.047493	0.083952	0.66	0.032001	0.243467
COMPOUND_1044_5898a10_241842	Rp	+	1044.5898	10.24				(1064.5604, 1804.85)(1065.0771, 553.61)(1065.5712, 1058.54)(1066.09, 541.78)(1066.5721, 1034.61)(1056.6189, 2804.61)(1057.1423, 728.55)(1057.6052, 2155.95)(1058.1108, 542.83)(1058.5994, 1272.56)(1059.0958, 459.26)(1059.5948, 1843.95)(1054.1318, 716.52)(1054.6047, 1005.05)(1055.1313, 1012.4)(1055.6322, 2558.74)(1056.1403, 1526.75)(1065.0807, 605.8)(1065.5905, 678.0)(1066.0865, 569.99)(542.2787, 1508.98)(542.7731, 688.88)(543.2765, 1101.64)(543.7775, 348.71)(1062.5925, 433.13)(1063.5781, 437.12)(1064.583, 417.07)(1045.5876, 40071.02)(1046.5905, 14493.35)(523.3026, 68383.26)(523.8028, 33677.92)(524.3034, 35749.46)(524.8049, 18152.76)(525.303, 15786.26)(525.8031, 7713.09)(526.3017, 4791.31)(526.8015, 1595.24)(527.2999, 5615.76)	914721.3	133846.8	829703.5	146873.4	898389.6	84320.05	909216.4	90442.38	-0.25	0.372957	0.474788	0.87	0.009976	0.153413
COMPOUND_1044_5939a10_125637	Rp	+	1044.5939	10.13				(1064.5928, 154.5)(1065.1143, 842.31)(1056.6208, 2300.63)(1057.1167, 1373.02)(1057.6102, 2196.99)(1058.1113, 1621.98)(1075.5836, 3297.66)(1076.0771, 300.35)(1073.1086, 812.03)(1073.6096, 1382.92)(1074.1337, 761.17)(1065.1134, 628.74)(1065.5919, 138.44)(1066.1302, 11646.09)(1083.5662, 2032.78)(1084.0813, 451.46)(1084.602, 2072.88)(534.2959, 8601.04)(534.7981, 7009.51)(535.3001, 11056.33)(535.8012, 4638.18)(536.3009, 5002.43)(536.8037, 2038.24)(537.2998, 2263.15)(1083.5669, 5700.34)(1045.6111, 19849.83)(1046.6196, 10176.84)(1047.622, 4294.8)(1048.6188, 1673.42)(561.2973, 1558.35)(561.786, 1237.99)(562.2816, 2036.97)(523.3022, 42805.41)(523.8033, 19916.34)(524.3038, 25051.48)(524.8044, 11850.21)(525.303, 11366.72)(525.8034, 5332.93)(526.3016, 3961.29)(526.8029, 2011.92)	856840.4	107760.4	813041	132309.7	639385.2	158485.2	767009.7	217410.6	-0.79	0.005414	0.012156	0.80	0.016204	0.189073
COMPOUND_1044_6176a10_105027	Rp	-	1044.6176	10.11				(1157.5953, 1886.14)(1079.5605, 577.37)(1080.5643, 325.45)(1043.6101, 19019.02)(1044.6129, 11736.02)(1045.6168, 3870.94)(1046.6217, 1233.85)(1047.6309, 415.92)(1048.6438, 107.96)	125370	29809.24	131767.9	39289.35	95103.79	38699.83	111509.6	48436.78	-0.80	0.007372	0.016119	0.25	0.469079	0.734042
COMPOUND_1046_727a10_64033	Rp	+	1046.7270	10.64				(1069.7178, 82444.88)(1070.7212, 48369.3)(1071.7166, 19190.94)(1072.71, 6680.41)(1047.7346, 993780.06)(1048.7384, 564931.06)(1049.7418, 178479.23)(1050.7433, 41029.23)(1051.7433, 8177.72)(1052.7393, 1909.09)	3700068	1240544	3671931	1119289	469053.3	360520	427876.8	315734.5	-1.55	1.44E-13	1.25E-11	-0.08	0.685239	0.853695
COMPOUND_1050_721a10_554148	Rp	-	1050.7210	10.55				(1085.7153, 77.75)(1086.7045, 260.08)(1087.701, 243.52)(1049.7137, 7253.72)(1050.7166, 4058.13)(1051.721, 1570.63)(1052.7158, 561.09)(1053.7217, 89.74)(1095.7354, 896.12)(1096.7372, 273.2)	28060.78	11445.53	29162	11155.03	12356.5	3322.695	12757.8	1804.714	-1.09	1.05E-07	6.48E-07	-0.27	0.199707	0.511069
COMPOUND_1051_763a10_412446	Rp	+	1051.7630	10.41				(1052.7661, 977.92)(1053.2467, 135.13)(1053.77, 552.68)(1054.2896, 84.65)(1072.3185, 301.3)(1072.8335, 94.57)(537.878, 1312.17)(538.3885, 3892.82)(538.876, 434.32)(535.4011, 7274.02)(535.9043, 4111.08)(536.386, 4216.11)(536.8826, 443.82)(537.3794, 1610.32)(1069.8131, 275.41)(554.3917, 1876.36)(554.8928, 2066.57)(555.3933, 2530.08)(1052.7687, 1324.96)(1053.7711, 811.45)(1054.763, 313.92)(1055.749, 52.59)(526.8873, 2469.13)(527.3828, 2233.59)(527.8878, 806.52)	177453	49126.95	145832.9	33158.44	208517.3	41328.42	202185.4	63118.6	0.26	0.346317	0.447707	0.64	0.056127	0.29488
COMPOUND_1052_5844a10_237459	Rp	+	1052.5844	10.24				(1064.559, 1800.46)(1065.0684, 518.83)(1065.5667, 1114.81)(1066.0856, 485.02)(1066.5671, 1072.19)(1053.5935, 1242.37)(1054.1108, 665.62)(1054.606, 878.95)(1055.1143, 665.23)(1062.1088, 417.49)(1062.623, 338.15)(1063.0952, 216.85)(1063.5769, 97.79)(546.2837, 557.73)(546.7993, 467.67)(1053.59, 1214.14)(1054.5994, 835.93)(1055.6097, 2490.27)(527.3001, 4832.76)(527.8008, 2314.72)(528.3094, 11250.4)(528.8055, 1845.57)(529.3089, 4372.18)(529.7969, 896.15)(530.3121, 1485.88)(530.807, 315.57)	151308.5	41530.93	152105.2	20768.26	152973.7	36327.08	159431.2	28950.66	-0.24	0.389666	0.493547	0.87	0.01271	0.172426
COMPOUND_1053_7465a9_911521	Rp	+	1053.7465	9.91				(1054.7465, 1691.7)(1055.237, 69.3)(546.8599, 272.66)(547.3547, 743.28)(547.8451, 644.91)(548.3514, 514.56)(548.8377, 277.6)(1082.225, 87.51)(1082.7491, 170.21)(538.8701, 4154.19)(539.3743, 3605.82)(539.8723, 1094.42)(540.3566, 184.25)(555.3827, 1287.85)(555.8858, 770.88)(556.3798, 797.19)(1054.7454, 3631.63)(1055.7485, 2107.43)(1056.7512, 871.26)(1057.7493, 281.09)(527.8795, 12147.13)(528.3814, 7481.44)(528.8836, 2970.22)	846679.3	218980.5	737788	154824	913433.1	148725.4	922636.7	227497.5	0.24	0.393361	0.497827	0.64	0.063493	0.307286
COMPOUND_1054_3676a9_157458	Rp	-	1054.3676	9.16				(1089.3416, 641.42)(1090.3436, 293.13)(1053.361, 44080.02)(1054.3647, 19966.87)(1055.369, 10003.46)(1056.3717, 3017.87)(1057.372, 748.26)(1058.3688, 185.66)	19415.89	8016.267	24051.54	12663.03	94038.08	85176.27	58749.82	43654.87	1.54	3.14E-09	2.95E-08	-0.88	0.001427	0.059516
COMPOUND_1054_5461a5_432122	Rp	+	1054.5461	5.43				(547.2589, 905.62)(547.7567, 575.47)(548.2626, 126.58)(539.2732, 273.89)(539.7646, 158.41)(1055.5527, 339.29)(1056.5533, 247.96)(528.2818, 15551.89)(528.7836, 9313.14)(529.2849, 3320.43)(529.7836, 1026.59)(530.2855, 315.4)(530.7824, 63.37)	48990.91	44962.71	71509.97	70186.03	12816	#DIV/0!	13201	2789.776	-0.83	6.7E-05	0.000215	-0.41	0.079756	0.339484
COMPOUND_1054_7773a10_289623	Rp	+	1054.7773	10.29				(1077.737, 163.85)(1078.2217, 140.66)(1078.7164, 176.12)(1077.729, 340.75)(539.3868, 1498.95)(539.8766, 520.76)(540.369, 5360.99)(540.8842, 879.85)(1072.8127, 105.5)(555.8911, 579.32)(556.3942, 1134.85)(1055.7295, 188.92)(528.3945, 12408.25)(528.8957, 7114.96)(529.3849, 16280.99)(529.8837, 2090.68)(530.3686, 273.94)	162064.5	65970.21	119934	40484.28	162868.1	44391.39	189558.7	66381.57	-0.23	0.406366	0.510394	0.58	0.086046	0.348516
COMPOUND_1060_6418a10_100553	Rp	+	1060.6418	10.10				(1083.6375, 4373.85)(1084.1332, 2044.28)(1084.6278, 2283.36)(1085.1204, 965.09)(1085.6523, 16583.19)(1086.1298, 1135.46)(1072.6309, 2541.53)(1073.1285, 1146.07)(1073.6357, 1838.21)(1074.1307, 1525.41)(1083.6343, 3564.6)(1084.6337, 1904.58)(1085.653, 14986.12)(1086.6562, 11254.09)(1087.6718, 79635.05)(1088.6747, 46553.86)(1089.6758, 16388.35)(1080.5879, 3717.17)(1081.1127, 1496.95)(1081.5898, 2894.64)(1082.117, 1244.76)(1082.6245, 1711.94)(1091.6349, 12364.53)(1092.132, 6236.81)(561.3012, 4710.66)(561.7961, 3245.35)(562.2899, 3044.9)(562.787, 1510.61)(1089.1152, 1041.53)(1089.617, 223.32)(1090.1257, 3493.54)(553.3013, 1837.49)(553.7961, 1157.46)(1061.6552, 24615.28)(1062.6584, 20607.58)(1063.6757, 439261.56)(531.3256, 7293.39)(531.8279, 4388.07)(532.3271, 4541.58)(532.8282, 2211.12)(533.3289, 1069.5)(533.8272, 384.6)	1002624	367506	991184.5	316743.6	295526.1	127747.2	349238.4	207650.2	-1.53	3.85E-11	7.54E-10	0.03	0.887399	0.953446
COMPOUND_1060_669a10_133247	Rp	-	1060.6690	10.13				(1059.6625, 77633.11)(1060.6656, 43884.13)(1061.6683, 14900.43)(1062.6704, 3472.79)(1063.6727, 1007.99)(1064.6759, 194.64)	311983.4	78428.12	349574.7	69720.59	170189	42703.95	166681.1	56800.62	-1.31	4.16E-09	3.78E-08	-0.40	0.080955	0.340768
COMPOUND_1063_67a10_101033	Rp	+	1063.6700	10.10				(1086.6573, 13021.73)(1102.6285, 7586.99)(1103.1335, 6130.7)(1103.6351, 5516.27)(1104.1356, 2600.76)(1104.6075, 3151.87)(1102.6302, 12656.31)(1103.634, 7664.79)(1104.6256, 2347.22)(1064.6708, 3064.72)(1065.6761, 1117.38)(1066.6731, 173.32)(1067.6807, 53.34)(532.8254, 1809.21)(533.3357, 1081.19)(533.8306, 195.73)	416342.2	171568	408402.6	144432.2	128955.8	52348.97	150753.7	89217.51	-1.57	3.71E-12	1.13E-10	0.13	0.556443	0.781554

									1st trimester				3rd trimester									
									Control		Pre-eclampsia		Control		Pre-eclampsia		Time			Interaction		
Compound	Column	Ion_mode	Mass	Retention_	Identification	ID_level	MSMS_spectra	CompositeSpectrum	Mean	SD	Mean	SD	Mean	SD	Mean	SD	Value	raw p	FDR_p	Value	raw p	FDR_p
COMPOUND_1067_7596a10_07238	Rp	+	1067.7596	10.07				(553.8711, 408.72)(554.3957, 7050.24)(1106.7122, 319.75)(1107.2314, 88.8)(545.8784, 3817.31)(546.3893, 895.95)(546.8797, 1091.74)(547.3906, 466.55)(543.3998, 39804.82)(543.9023, 21537.88)(544.3972, 4364.65)(544.9001, 1569.49)(1085.7598, 119.59)(1086.742, 113.6)(562.3917, 1583.2)(562.8952, 1203.42)(1085.7719, 165.49)(1086.2906, 257.74)(1068.7618, 3928.47)(1069.7653, 2303.33)(1070.7668, 1033.85)(1071.7676, 363.6)(554.3953, 5319.88)(554.8779, 677.12)(534.8865, 13066.96)(535.388, 9703.53)(535.8899, 2967.3)	721025.4	221903.3	603945.5	136739.3	863539.3	129468.5	868073.9	205594.3	0.35	0.211831	0.304237	0.60	0.071857	0.325554
COMPOUND_1068_6165a10_101867	Rp	-	1068.6165	10.10				(1103.5648, 533.76)(1104.5417, 483.08)(1105.5557, 134.02)(1067.6099, 7699.94)(1068.6129, 4799.97)(1069.617, 1845.86)(1070.6191, 565.17)(1071.6403, 177.47)	36648.09	11476.9	35634.87	15357.05	24143.92	11060.18	32585.57	16726.74	-1.05	0.000206	0.000613	0.75	0.019188	0.196411
COMPOUND_1073_5898a10_282955	Rp	+	1073.5898	10.28				(1096.5927, 1751.88)(1074.5951, 8465.68)(1075.5974, 6251.72)(1076.5986, 2644.55)(1077.5992, 1022.75)(537.8092, 894.93)(538.3066, 2114.92)	70582.35	8609.056	67513.64	8044.893	72707.38	4483.605	73651.65	5558.954	0.19	0.469022	0.572936	0.58	0.064138	0.307286
COMPOUND_1073_8217a11_1704855	Rp	-	1073.8217	11.17				(1072.815, 36494.11)(1073.8185, 23919.27)(1074.8214, 8665.76)(1075.8254, 2271.5)(1076.8254, 680.36)(1077.8145, 132.28)(1118.804, 354.06)(1119.8195, 208.13)(535.9018, 536.4)(536.3867, 188.84)(536.8988, 277.96)	93755.37	43566.73	117349.5	57246.75	52553	#DIV/0!	65978.22	36157.98	-1.06	7.62E-07	3.91E-06	-0.34	0.146475	0.455609
COMPOUND_1076_2094a9_696656	Rp	-	1076.2094	9.70				(1189.1545, 208.83)(1190.1405, 239.81)(1111.1934, 253.44)(1112.1841, 123.03)(1075.1998, 6477.11)(1076.2023, 2471.08)(1077.2008, 2547.79)(1078.2018, 916.75)(1079.2098, 343.84)(1080.208, 312.71)(1081.1904, 182.79)	91400.7	21362.18	91348.59	18494.01	85233.5	20045.14	81081.4	16650.32	-0.28	0.326115	0.426487	-0.28	0.411538	0.694741
COMPOUND_1076_6108a10_100987	Rp	+	1076.6108	10.10				(1099.5715, 2902.96)(1100.0836, 618.09)(1096.5962, 2033.91)(1097.106, 870.64)(1097.5867, 1712.5)(1098.097, 670.52)(1107.5848, 3375.67)(1108.096, 958.12)(1077.623, 2898.34)(1078.128, 8540.2)(1078.6295, 13577.36)(1079.1316, 10996.87)(1079.6339, 6880.16)(1105.0936, 950.25)(1105.5709, 4684.42)(1106.0825, 717.02)(558.2953, 8024.97)(558.7973, 8731.9)(559.2994, 15751.36)(559.8018, 6369.38)(560.3017, 7108.34)(560.8028, 3341.48)(561.3008, 2711.92)(1115.5972, 2658.93)(1116.1263, 684.26)(1116.5786, 14182.69)(561.3011, 1719.29)(561.7918, 870.68)(562.31, 6778.45)(562.8086, 4206.62)(563.3114, 4849.26)(550.2921, 4113.49)(550.7913, 2295.34)(551.309, 6676.01)(551.8098, 4083.42)(552.3139, 8424.76)(1077.6176, 2613.58)(539.3132, 14304.0)(539.8176, 7625.98)(540.3099, 5245.74)(540.8068, 2018.03)(541.3012, 3568.06)	583686	96197.27	572225.8	88351.41	347159.4	70039.94	345593.9	105892.7	-1.57	1.06E-11	2.65E-10	0.12	0.594847	0.804532
COMPOUND_1076_7372a10_636591	Rp	-	1076.7372	10.64				(1075.7295, 11208.55)(1076.7336, 6977.42)(1077.734, 2470.33)(1078.7323, 632.68)(1079.7443, 156.82)(1121.7177, 117.6)(1122.6818, 839.15)	51851.4	12970.39	57024.26	18056.25	23675.67	12100.2	19910.5	9235.262	-1.46	4.98E-13	2.74E-11	-0.15	0.41538	0.697566
COMPOUND_1080_559a10_1455345	Rp	+	1080.5590	10.15				(1098.5948, 4693.71)(1081.5653, 19196.84)(1082.5627, 15154.54)	108722.3	18266.51	101736.5	23198.25	73633.92	25980.12	78202.94	20345.15	-0.37	0.165898	0.249069	0.53	0.098418	0.36878
COMPOUND_1081_7751a10_223258	Rp	+	1081.7751	10.22				(560.8721, 270.86)(561.3791, 1128.92)(561.8911, 294.08)(552.8857, 3707.96)(553.3887, 3472.49)(553.8883, 885.63)(569.3958, 1457.21)(569.9009, 415.35)(1099.7684, 177.85)(550.4089, 28097.02)(550.9106, 17241.8)(551.4097, 7099.86)(551.909, 2222.31)(552.4079, 736.4)(1120.705, 238.79)(1082.778, 3491.83)(1083.7806, 2034.01)(1084.7784, 886.92)(1085.7695, 313.99)(541.8948, 9498.85)(542.3962, 6597.29)(542.8989, 2191.82)	371377.1	95107.54	315124.8	73104.42	463706.3	72512.79	465181.5	110690.6	0.61	0.019253	0.038302	0.66	0.034076	0.248347
COMPOUND_1082_7032a10_653781	Rp	-	1082.7032	10.65				(1081.696, 12669.65)(1082.6993, 7036.84)(1083.696, 5977.67)(1084.6984, 2801.55)(1085.699, 798.44)	92881.15	12196.01	95056.15	17827.6	51156.43	10609.84	51141.56	13790.83	-1.51	1.32E-12	5.62E-11	-0.15	0.437544	0.714538
COMPOUND_1083_7548a9_725081	Rp	+	1083.7548	9.73				(562.8549, 404.23)(561.8796, 569.84)(562.3659, 430.24)(553.876, 2534.34)(554.3842, 3004.87)(554.8763, 755.32)(555.3596, 196.72)(551.3989, 13280.33)(551.9, 7758.32)(552.3994, 3269.71)(552.8978, 1050.79)(570.386, 578.68)(570.8894, 451.41)(1101.7623, 84.04)(1102.2771, 333.74)(562.3753, 566.22)(562.8637, 364.41)(1084.7559, 1318.01)(1085.7588, 839.28)(1086.757, 313.45)(1087.7611, 109.74)(542.8848, 9060.38)(543.3878, 5867.67)(543.8877, 2253.51)	556319	148498.5	492304.1	101562.1	591025.8	99477.29	593397	146529.3	0.22	0.447596	0.552927	0.57	0.0975	0.368406
COMPOUND_1084_6696a10_110284	Rp	-	1084.6696	10.11				(1083.6633, 202087.77)(1084.6666, 117553.05)(1085.669, 38922.19)(1086.6715, 9253.99)(1087.6731, 2197.4)(1088.6715, 363.67)	536806.5	229930.9	588977.6	210629.7	277744	102781.7	247698	108811.4	-1.14	1.72E-06	8.21E-06	-0.37	0.144511	0.451201
COMPOUND_1092_5872a10_101798	Rp	+	1092.5872	10.10				(1102.1315, 3630.56)(1102.6305, 5194.77)(1103.1317, 4040.44)(1103.6302, 3113.48)(1113.0988, 431.05)(1113.6288, 826.26)(1114.102, 717.25)(1121.0801, 763.91)(1121.5743, 1423.93)(1122.0739, 685.62)(1131.5667, 4405.65)(1132.079, 772.67)(1132.5734, 2046.08)(1133.0854, 579.07)(1133.5712, 1393.12)(1134.0919, 347.49)(1134.564, 1045.2)(558.2953, 8024.97)(558.7973, 8731.9)(559.2994, 15751.36)(559.8018, 6369.38)(560.3017, 7108.34)(560.8028, 3341.48)(561.3008, 2711.92)(561.7967, 1687.43)(1131.5647, 8025.87)(1132.5634, 3374.53)(1133.5623, 1544.67)(555.7964, 4881.4)(556.2977, 3827.84)(556.7949, 1782.45)(1093.5883, 6796.05)(1094.5886, 2712.39)(1095.5857, 1172.53)(1096.5983, 161.35)(585.2347, 5479.09)(585.7362, 576.33)(547.302, 29909.7)(547.8034, 14633.33)(548.302, 14773.35)(548.8034, 6654.05)(549.3012, 6329.88)(549.7957, 3678.69)(550.2866, 3832.53)	598592.3	107880.1	569187.6	107242.3	439398	95824.86	413361.7	127153.1	-1.24	4.54E-07	2.49E-06	0.10	0.69069	0.856988
COMPOUND_1092_7332a10_652321	Rp	-	1092.7332	10.65				(1205.7405, 1016.5)(1206.7592, 481.81)(1207.7681, 508.3)(1091.7257, 190107.83)(1092.7288, 109704.0)(1093.7313, 36525.8)(1094.7344, 9033.92)(1095.7384, 2018.13)(1096.7408, 538.49)(1097.724, 115.08)	1170852	240470.1	1166130	284686.9	352864.7	112468.4	340387.9	125129.1	-1.54	4.13E-14	4.89E-12	-0.10	0.562999	0.785548
COMPOUND_1094_7701a10_057778	Rp	+	1094.7701	10.06				(1117.7344, 383.33)(1118.7404, 212.94)(1119.7411, 171.64)(1115.2618, 189.43)(1115.78, 250.31)(559.3946, 3476.41)(559.888, 712.36)(560.3772, 626.26)(575.9122, 172.94)(576.3863, 631.02)(576.8773, 642.74)(1112.787, 2533.23)(1113.7906, 1674.34)(1114.79, 670.32)(1115.7974, 228.14)(567.8937, 2918.73)(568.4, 3463.46)(568.8939, 519.76)(569.3793, 61.06)(1095.7667, 293.32)(1096.7583, 203.49)(548.3859, 4401.73)(548.8874, 1552.54)(549.3912, 9760.56)(549.8918, 1021.49)(550.4031, 6344.51)	909216.2	257404.1	780178.7	178248.6	986437.2	169061.9	992926.3	246040.3	0.28	0.322185	0.423801	0.61	0.070931	0.325554
COMPOUND_1097_7708a9_898185	Rp	+	1097.7708	9.90				(1128.7645, 139.16)(1129.246, 270.76)(568.8448, 136.52)(569.3448, 5448.52)(560.8847, 3556.76)(561.3843, 2822.82)(561.8843, 1156.22)(562.3769, 463.35)(577.3977, 1390.71)(577.899, 828.24)(569.3948, 1546.53)(569.8793, 365.44)(570.3933, 1213.89)(570.8996, 833.47)(571.3848, 1132.71)(1098.7704, 2174.99)(1099.773, 1405.55)(1100.7765, 543.18)(1101.7872, 157.43)(549.8928, 5640.34)(550.3947, 4038.03)(550.8965, 1664.8)(551.3813, 240.1)	819029.8	212133.8	712792.8	154891.6	852830.3	158252.7	864609.3	216123.7	0.11	0.693543	0.777045	0.66	0.059521	0.300425
COMPOUND_1112_8156a10_392269	Rp	+	1112.8156	10.39				(1135.7767, 202.01)(1133.2784, 113.81)(1133.7513, 121.76)(576.4033, 2144.11)(576.907, 1785.36)(577.407, 2153.92)(568.4076, 3009.69)(568.8919, 646.91)(569.399, 2302.51)(569.9039, 1296.49)(570.3963, 1176.62)(576.9074, 1216.05)(577.4065, 1326.45)(1113.772, 202.56)(557.4142, 15260.35)(557.9164, 8724.96)(558.4049, 9150.74)(558.9073, 1895.11)(559.3983, 2228.09)	174609.8	50426.29	123864.2	32112.4	182619	61394.49	186823.8	65935.3	0.12	0.664625	0.752129	0.36	0.256344	0.56678
COMPOUND_1125_8018a10_2129755	Rp	+	1125.8018	10.21				(580.9132, 749.48)(581.4029, 869.82)(574.8989, 4749.52)(575.3991, 3372.29)(575.899, 1182.1)(576.3821, 234.01)(572.4217, 16814.04)(572.9243, 10806.01)(573.4209, 5439.7)(573.9219, 1640.04)(574.418, 370.69)(591.4175, 1866.29)(591.9118, 888.23)(583.4142, 6371.76)(583.9034, 525.44)(584.4101, 2429.62)(584.9101, 983.25)(1126.8038, 2314.96)(1127.8053, 1482.08)(1128.8074, 691.01)(1129.801, 201.19)(1130.8136, 41.68)(601.8511, 78.76)(602.326, 279.79)(563.9079, 12193.92)(564.4105, 8066.71)(564.9114, 2906.45)	514236.3	133717.9	442890.6	99873.74	597568.2	93278.93	590937.8	137688.5	0.42	0.123656	0.195283	0.64	0.052669	0.2865

									1st trimester				3rd trimester									
									Control		Pre-eclampsia		Control		Pre-eclampsia		Time			Interaction		
Compound	Column	Ion_mode	Mass	Retention_	Identification	ID_level	MSMS_spectra	CompositeSpectrum	Mean	SD	Mean	SD	Mean	SD	Mean	SD	Value	raw p	FDR_p	Value	raw p	FDR_p
COMPOUND_1135_618a10_640722	Rp	+	1135.6180	10.64				(1158.6073, 5359.03)(1159.6107, 4493.61)(1160.6086, 3544.94)(1174.6038, 1564.32)(1153.6445, 1265.47)(1154.641, 877.53)(1155.6536, 389.09)(1136.6241, 12167.77)(1137.6304, 8170.6)(1138.6254, 7202.92)(1139.627, 3439.31)(1140.63, 1162.42)	120221.4	32875.35	107679.3	28744.9	114646.3	24804.53	114926.2	40237.71	0.06	0.826559	0.877196	0.31	0.355709	0.665596
COMPOUND_1139_8173a10_349059	Rp	+	1139.8173	10.35				(589.9019, 471.03)(590.3955, 1413.58)(590.9121, 1139.2)(581.907, 2267.04)(582.4121, 2759.03)(582.9068, 699.86)(583.3902, 658.65)(579.4255, 10886.18)(579.9286, 6591.55)(580.4186, 4284.01)(580.921, 1834.89)(581.4061, 1851.74)(598.4163, 1877.53)(598.9178, 1909.34)(1140.8195, 1305.2)(1141.8248, 983.31)(1142.821, 516.49)(570.9137, 3118.54)(571.4134, 2930.65)(571.9162, 1130.5)	308681.7	86199.11	244989.3	64473.16	331206.7	54955.86	319804.2	85051.64	0.39	0.168598	0.252094	0.43	0.203328	0.5149
COMPOUND_1154_5446a10_36111	Rp	+	1154.5446	10.36				(1177.545, 624.31)(1178.0344, 77.94)(1178.556, 510.87)(1177.5481, 588.38)(1178.5577, 500.63)(1179.5614, 553.87)(1185.5674, 507.87)(1186.0498, 62.64)(600.2574, 3648.8)(600.7514, 463.46)(601.2545, 3340.36)(589.2682, 1078.0)(589.7702, 706.71)(590.2758, 1525.67)(590.7855, 1899.1)(591.2713, 2003.17)(591.7709, 1288.26)(1172.5641, 577.26)(1173.0483, 342.53)(1173.5588, 591.42)(1174.0701, 236.43)(1174.5397, 275.52)(1175.0441, 219.24)(1155.5796, 2098.6)(578.282, 6155.0)(578.7826, 2043.11)(579.2846, 3103.0)(579.7949, 1319.72)(580.2853, 3786.11)(580.7884, 606.74)(581.2788, 6485.33)(581.771, 307.91)(582.2822, 2576.89)	216308.6	42864.62	208567	52443.97	139050.6	14067.04	138835.6	21397.38	-0.15	0.581683	0.678028	-0.02	0.94447	0.974642
COMPOUND_1155_8156a10_045544	Rp	+	1155.8156	10.05				(597.8999, 318.72)(598.4212, 2560.46)(589.9068, 4501.87)(590.4055, 3619.3)(590.9076, 1117.93)(591.4004, 448.51)(606.4133, 1756.01)(606.9207, 931.25)(607.4234, 1643.83)(1156.8132, 1718.11)(1157.817, 1284.87)(1158.8204, 613.14)(1159.8015, 149.59)(598.4237, 5518.46)(598.9139, 288.89)(578.9128, 12407.64)(579.4141, 11908.36)(579.9177, 3632.77)	803967	221970.5	681474.7	164885.6	893279.8	155693.7	891624	218532.9	0.32	0.256236	0.352716	0.66	0.050463	0.283798
COMPOUND_1169_8315a10_19566	Rp	+	1169.8315	10.20				(1198.3396, 71.81)(1198.8466, 79.58)(604.91, 238.48)(605.4003, 1366.28)(605.8941, 532.79)(596.9132, 5402.94)(597.4151, 4440.27)(597.9148, 1514.18)(594.4347, 29400.79)(594.9364, 18793.95)(595.4363, 7963.57)(595.9364, 2667.37)(596.4317, 947.21)(1208.7393, 270.52)(613.4303, 3221.18)(613.9255, 598.33)(614.4215, 2633.11)(614.9282, 384.01)(615.4315, 1444.74)(1170.8298, 1899.79)(1171.8336, 1230.54)(1172.8328, 583.6)(1173.8323, 217.69)(585.9219, 10477.1)(586.4236, 7273.27)(586.9259, 2806.01)	566638.2	155890.6	479553.9	108583.6	646033.6	112773.8	638014.1	161511.6	0.36	0.187928	0.27643	0.65	0.052504	0.286293
COMPOUND_1170_8593a10_492284	Rp	+	1170.8593	10.49				(597.4275, 7652.67)(597.9165, 1094.25)(598.4064, 4611.57)(598.9242, 2375.38)(613.9326, 1506.34)(614.4277, 1992.0)(614.9235, 797.14)(1171.7983, 155.84)(586.4361, 14807.61)(586.938, 9199.98)(587.4321, 5651.45)(587.9258, 2304.84)	110801.4	31829.81	91129.32	25444.52	124195	26801.78	120069.1	33408.53	0.41	0.114775	0.183836	0.57	0.066707	0.313621
COMPOUND_1185_279a9_739681	Rp	+	1185.2790	9.74				(1208.2289, 409.23)(1186.289, 6912.78)(1187.2909, 2907.3)(1188.297, 715.75)(1189.3033, 137.45)	74950.3	13891.02	70200.65	13748.68	78099.93	10306.86	75789.96	18387.84	0.23	0.43537	0.540195	0.15	0.680752	0.853001
COMPOUND_1197_8564a10_469222	Rp	+	1197.8564	10.47				(1209.8568, 98.58)(1210.3401, 75.16)(1198.8555, 480.05)(1199.362, 56.35)(1199.8634, 453.31)(618.9232, 505.29)(619.4175, 1296.3)(1236.7979, 117.54)(1237.2968, 104.15)(610.9269, 1905.82)(611.4286, 2407.73)(611.9191, 764.48)(608.4492, 11746.91)(608.9518, 7671.42)(609.4459, 4092.41)(609.9421, 1827.04)(610.4444, 307.61)(1198.861, 523.01)(1199.8667, 459.77)(1200.8691, 215.83)(599.9352, 3426.26)(600.4304, 3271.9)(600.9356, 1190.42)	148884.4	38233.61	129892.2	27483.85	177960.5	38360.54	177035	46809.84	0.54	0.043004	0.076617	0.56	0.077534	0.333235

Legend: ACar, acetylcarnitine; FA, fatty acid; MAG, monoacylglyceride; PC, phosphatidylcholine; PE, phosphatidylethanolamine; p-values are from linear mixed model (both raw and FDR corrected are shown)
